# ‘Second-generation’ 1,2,3-triazole-based inhibitors of *Porphyromonas gingivalis* adherence to oral streptococci and biofilm formation†
†Electronic supplementary information (ESI) available. See DOI: 10.1039/c8md00405f


**DOI:** 10.1039/c8md00405f

**Published:** 2019-01-15

**Authors:** Pravin C. Patil, Jinlian Tan, Donald R. Demuth, Frederick A. Luzzio

**Affiliations:** a Department of Chemistry , University of Louisville , 2320 South Brook Street , Louisville , Kentucky 40292 , USA; b Department of Oral Immunology and Infectious Diseases , University of Louisville , School of Dentistry , 501 S. Preston St. , Louisville , Kentucky 40292 , USA . Email: drdemu01@louisville.edu

## Abstract

Several ‘second-generation’ click inhibitors of the multi-species biofilm propagated by the adherence of the oral pathogen *Porphyromonas gingivalis* to *Streptococcus gordonii* were synthesized and evaluated. The design of the structures was based on the results obtained with the first-generation diphenyloxazole ‘click’ inhibitors which bear suitable hydrophobic and polar groups within a dual scaffold molecule bearing a 1,2,3-triazole spacer. The structures of the synthetic targets reported herein now consist of a triazolyl(phenylsulfonylmethyl) and a triazolyl(phenylsulfinylmethyl) spacer which joins a 4,5-diphenyloxazole with both phenyl rings bearing lipophilic substituents. The triazolyl “linker” group is formed by a click reaction between the 4-azido(phenylsulfonyl/sulfinylmethyl) oxazoles and acetylenic components having aryl groups bearing hydrophobic substituents. The 1,3,5-trisubstituted-2,4,6-triazine scaffold of the most active click compounds were modeled after the structural motif termed the VXXLL nuclear receptor (NR) box. When substituted at the 3- and 5-positions with 2- and 4-fluorophenylamino and *N*,*N*-diethylamino units, the candidates bearing the 1,3,5-trisubstituted-2,4,6-triazine scaffold formed a substantial subset of the second-generation click candidates. Four of the click products, compounds **95**, **111**, **115** and **122** showed inhibition of the adherence of *P. gingivalis* to *S. gordonii* with an IC_50_ range of 2.3–4.3 μM and only **111** exhibited cytotoxic activity against telomerase immortalized gingival keratinocytes at 60 μM. These results suggest that compounds **95**, **115**, **122**, and possibly **111** represent the most suitable compounds to evaluate for activity *in vivo*.

## Abstract 2

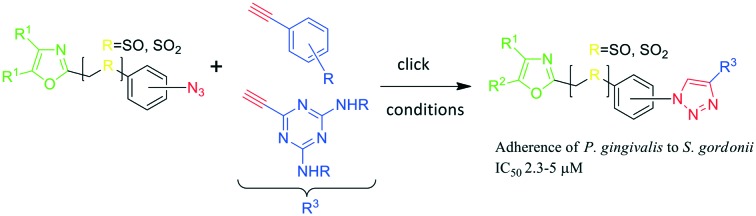
This study details the design, synthesis and bioassay of ‘click’ peptidomimetic compounds which inhibit the adherence of *P. gingivalis* to *S. gordonii*, a primary step toward pathogenic colonization of the subgingival pocket.

## 1. Introduction

**Fig. 1:**
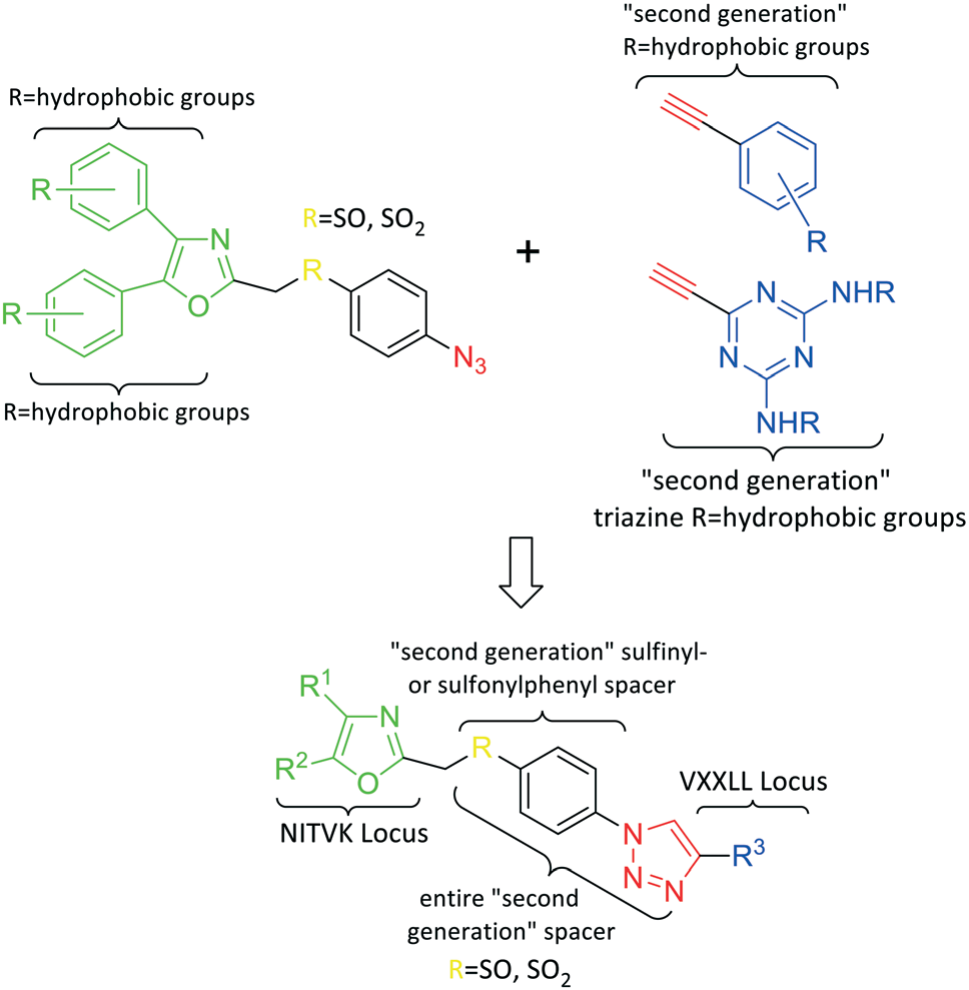
General azide/alkyne scaffold construction of second-generation inhibitors *via* a ‘click’ reaction. The entire linker modality is a sulfinyl- or sulfonylphenyltriazole. NITVK locus = green; VXXLL locus = blue; yellow R = sulfinyl/sulfonyl.

In the human oral cavity, a consortium of anaerobic bacteria including *Porphyromonas gingivalis*, *Tannerella forsythensis* and *Treponema denticola* colonizes the subgingival pocket and has been designated as the ‘red complex’ that is strongly associated with chronic adult periodontitis.^1^*P. gingivalis* is considered a key periodontal pathogen that may function to shape the overall microbial community leading to dysbiosis and tissue damage.^2^–^5^ Current methods to treat periodontitis involves scaling and root planing and surgery may be required to reduce subgingival pocket depth in more severe cases. Therapeutic approaches that specifically target periodontal pathogens like *P. gingivalis* are lacking. In addition, therapies that prevent or limit re-colonization of the oral cavity by *P. gingivalis* after treatment procedures are not available. Although the primary niche for *P. gingivalis* is a mixed community of bacterial species in the subgingival pocket, upon initial entry into the oral cavity it first colonizes supragingival plaque^6^ and the interaction of *P. gingivalis* with oral streptococci is important for this early colonization event.^7^,^8^ Thus, adherence of *P. gingivalis* with commensal streptococci represents an ideal point for therapeutic intervention to control colonization (or re-colonization) of the oral cavity by *P. gingivalis*. Adherence to streptococci is mediated by a protein–protein interaction that occurs between the minor fimbrial antigen (Mfa) of *P. gingivalis* and the antigen I/II (Ag I/II) polypeptide of streptococci.^9^–^11^ Daep *et al.* identified a discrete domain in Ag I/II protein that is required for interaction with Mfa and showed that this region resembles the eukaryotic nuclear receptor (NR) box protein–protein interaction domain.^9^,^10^ Daep *et al.* also showed that the NR box is composed of two functional peptide motifs, VXXLL and NITVK, and a synthetic peptide encompassing both motifs functioned as a potent inhibitor of *P. gingivalis* adherence to streptococci and significantly reduced *P. gingivalis* virulence *in vivo*.^11^ These studies suggested that *P. gingivalis* colonization of the oral cavity can be controlled by preventing its initial association with streptococci and that inhibitors of the Mfa–Ag I/II interaction may represent potential therapeutic agents to control periodontal disease. However, the use of peptides as therapeutic agents has limitations arising from the relatively high cost of peptide synthesis and their susceptibility to degradation by proteases expressed by oral organisms, including *P. gingivalis* itself. The development of small-molecule peptidomimetics is one approach to generate hydrolytically stable inhibitory analogues of the inhibitory peptide and at a decreased cost of synthesis. To this end, we previously reported the synthesis and testing of small-molecule inhibitors based on a non-peptide backbone that mimic the natural peptide substrate recognized by Mfa.^12^,^13^ A 2,4,5-trisubstituted oxazole framework was utilized to mimic the NITVK motif, and mono-, di- and trisubstituted aryl rings having hydrophobic substituents represented VXXLL mimics. These two synthetic small-molecule scaffolds were subsequently joined *via* the ‘click’ reaction.^14^–^22^ In this report, we describe the syntheses and click reactions of similar 2,4,5-trisubstituted oxazole NITVK mimics.^23^–^26^ These reacting components are now used in conjunction with sulfinylaryl- and sulfonylaryl-1,2,3-triazole linkers, albeit with the mono- and disubstituted aryl groups acting as the VXXLL structural mimic. Similar to the previous ‘first-generation’ compounds, the newly formed 1,2,3-triazole linker arising from the click reaction functions as the polar, slightly basic section of the entire peptidomimetic with the phenylsulfinyl or phenylsulfonyl portion allowing several more degrees of conformational freedom in the molecule's overall topology. These second-generation compounds are divided into three groups (**Groups I**, **II**, and **III**) based on the linker motifs joining the two scaffolds, the substitutions on the phenyl rings of the 4,5-diaryloxazole, the hydrophobic substituents on the distal (VXXLL) aryl ring and those in which the 3,5-(arylamino)-substituted 2,4,6-triazine unit represents the distal VXXLL locus (Fig. 1). Further, the 2,4,6-triazines all bear hydrophobic substituents such as 2- and 4-fluorophenylamino and required installation of the acetylenic component so that the resulting 6-acetylenic-2,4-di-*N*-substituted 1,3,5-triazines could react with the NITVK-mimic co-reactant azides in the click reaction.

## 2. Results and discussion

### 2.1. Design and chemistry

**Scheme 1:**
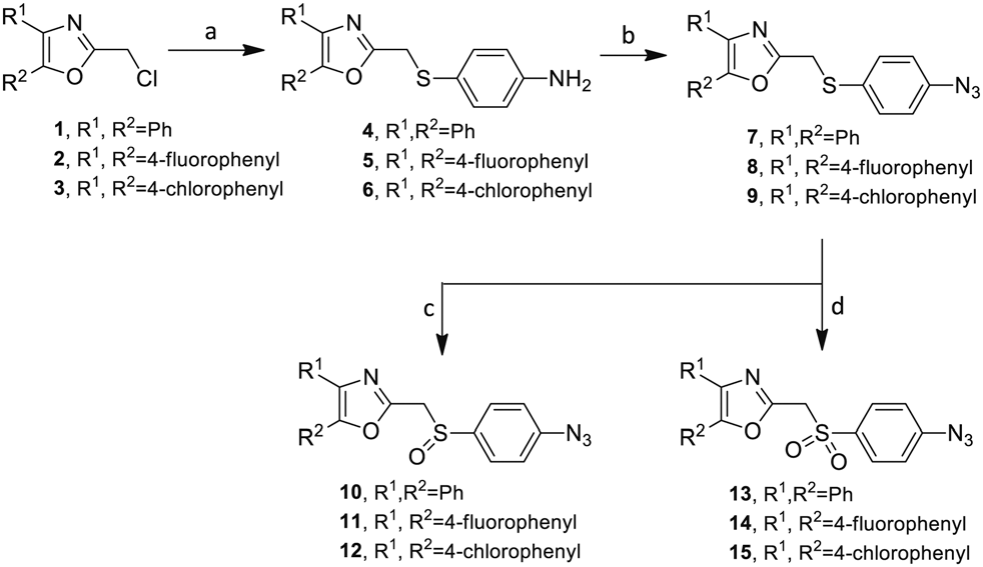
Synthesis of azidophenylsulfinyl and azidophenylsulfonyl click partners **10–15**. Reagents/conditions: (a) NaH/4-aminothiophenol/THF/0–5 °C to rt/16 h (66–85%). (b) NaNO_2_/10% HCl/NaN_3_/0–5 °C to rt/16 h (61–98%). (c) MCPBA (1.2 equiv.)/DCM/rt/16 h (86–95%). (d) MCPBA (3 eq.)/DCM/rt/16 h (86–93%).

**Scheme 2:**
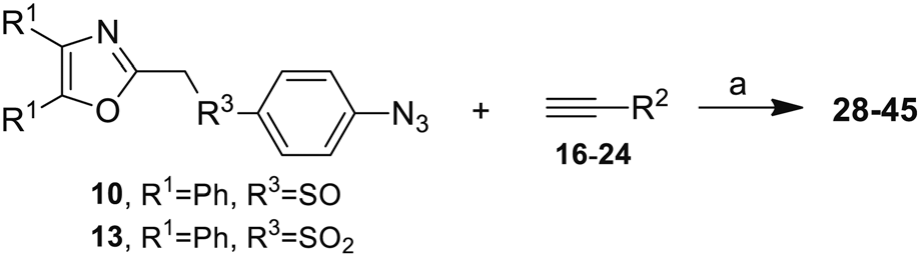
Click reactions of azidophenylsulfoxides **10** and azidophenyl-sulfones **13** with selected arylacetylenes **16–24** to give **Group I** triazoles **28–45**. ^a^Reagents/conditions: CuSO_4_·5H_2_O/Na ascorbate/THF–H_2_O (2 : 1).

Our approach toward the design of the second-generation inhibitors is modelled after the previously reported individual VXXLL and NITVK-peptidomimetic motifs which encompasses both the trisubstituted oxazole (NITVK) and mono- and disubstituted aryl (VXXLL) segments of the original inhibitory polypeptide designed in these laboratories.^12^ The separate motifs were each functionalized with azide and acetylene reactant groups to facilitate the formation of the “click” triazole linker which provided the completed scaffold. Within the substituted aryl groups of the VXXLL segment bearing acetylenic reacting components is the subgroup of *meta*-disposed trisubstituted triazines which bear two fluorophenylamino groups. The alkyldiaminotriazine scaffold possesses nonpolar hydrophobic *N*-alkyl groups which mimic the one valine and two leucine groups in the inhibitory helical peptide. For the hydrophobic substituents on the benzenoid rings of the VXXLL locus, we chose the halogens (F, Cl, Br), methoxy (OCH_3_) and trifluoromethyl (CF_3_) groups, all of which were situated on aromatic rings. In terms of hydrophobicity trends, F > Cl > Br, with the methoxy group being more polar and less hydrophobic than bromine and the trifluoromethyl group being more hydrophobic than a single fluorine. The planarity of the substituted oxazole ring excludes any stereochemical considerations thereby simplifying the initial design of the inhibitory NITVK scaffolds. The NITVK scaffolds were designed with the azide function of the click coupling partner positioned with at least a one-carbon spacer or a phenyl-ring spacer between the azide moiety and the oxazole torus with the azidoalkyl or azidoaryl group located at the 2-position of the oxazole. Given that the naphthalene core has been implicated in mimicking the double leucine motif in earlier studies, our selection included an alkynylnaphthalene bearing a hydrophobic methoxy group (Table 1). It should be noted that our initial selections of simple aryl-substituted VXXLL mimics constituted only aryl rings which were singly substituted with hydrophobic groups. Moreover, the newly formed 1,2,3-triazole or otherwise “linker” ring resulting from the click reaction may function as a polar, slightly basic backbone of the complete peptidomimetic. It may also be noted that triazole rings can function as mild hydrogen bond acceptors as well as π-stacking structures. We chose a sulfinyl/sulfonyl group in tandem with a *para*-substituted azidophenyl group to now provide an extended linker which should have unique interaction properties. There is a dual character of the weakly polar sulfonyl group as a hydrogen bond acceptor and as a hydrophobic group.^27^ Furthermore, the sulfonyl group is capable of forming van der Waals interactions with nonpolar atoms together with weak hydrogen bonds involving hydrogens α to electron-withdrawing groups. A very similar issue characterizes the sulfinyl group whereby the sulfoxide (S=O) itself is also a very weak hydrogen bond acceptor.^28^ The **Group I** inhibitor synthesis begins with the preparation of the individual azide and acetylene click reacting partners (Scheme 1). 2-Chloromethyl-4,5-diphenyloxazoles **1–3**, versatile synthetic intermediates prepared in multi-gram quantities and in modest to good yields by methods previously reported from these laboratories, are the starting points.^23^–^26^ The chloromethyl oxazoles **1–3** are reacted with 4-aminothiophenol in the presence of sodium hydride in THF to afford the 4-(aminothiophenyl)oxazoles **4–6** in yields ranging from 66% to 85%. Treatment of the (aminothiophenyl) oxazoles **4–6** with sodium nitrite in 10% aqueous HCl and sodium azide (5 °C to rt, 16 h) gave the corresponding 4-azidophenyl(oxazolyl)sulfides **7–9** (61–98%) with no interference from the sulfide group. Oxidation of the sulfide moiety of **7–9** could provide either the corresponding sulfoxides **10–12** or the corresponding sulfones **13–15** depending on the equivalents of oxidant employed. Thus, treatment of the sulphides **7–9** with *m*-chloroperbenzoic acid (1.2 equivalents) in dichloromethane (16 h, rt) gave the corresponding sulfoxides **10–12** (86–95%), while treating **7–9** with three equivalents of the oxidant provided the corresponding sulfones **13–15** (86–93%) in excellent yield. Both the oxidations of the sulfides to the sulfoxides and sulfones were optimized and these compounds were obtained as crystalline solids which were easily purified by column chromatography on silica gel. Interestingly, ^1^H NMR spectra revealed a distinct non-equivalence between the enantiotopic methylene protons at the 2-position of the oxazole ring in sulfoxides **10–12**, while the same set of protons in the corresponding sulfones **13–15** were equivalent. Using the percarboxylic acid oxidation protocol, the sulfoxide products are presumed to be racemic at sulfur and were bioassayed as the racemic mixtures. The click reactions of azidophenylsulfoxides **10–12** and azidophenylsulfones **13–15** with the acetylenic partners **16–24** from Table 1 to give the click triazole products **28–45** (Scheme 2) were conducted under standard conditions (CuSO_4_/sodium ascorbate/THF/H_2_O) developed in these laboratories for our previously reported candidates. The yields of the triazole click products **28–45** are presented in Table 2.

**Table 1:** Arylalkynyl click partners **16–27**^*a*^

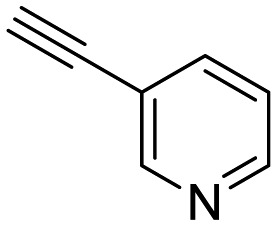 **16**	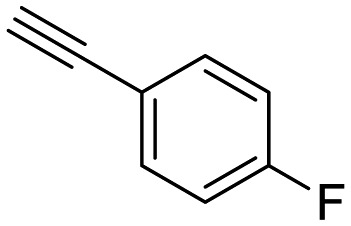 **17**	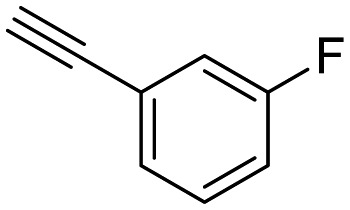 **18**
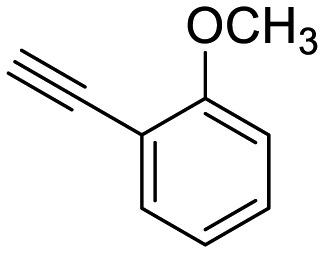 **19**	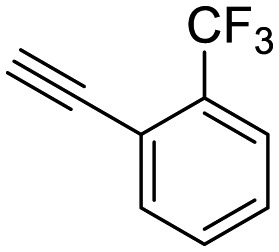 **20**	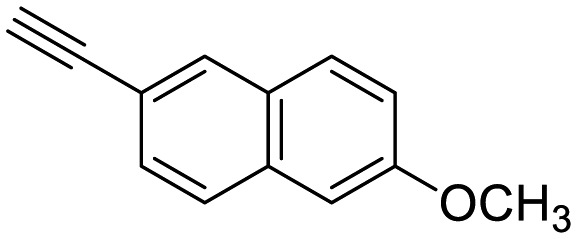 **21**
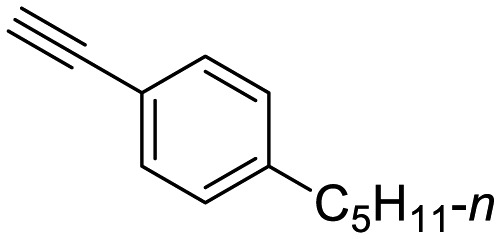 **22**	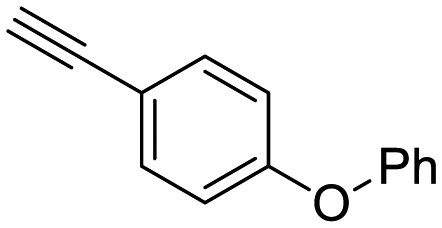 **23**	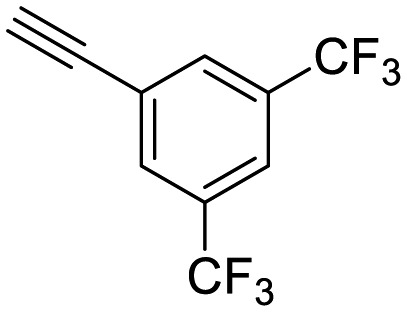 **24**
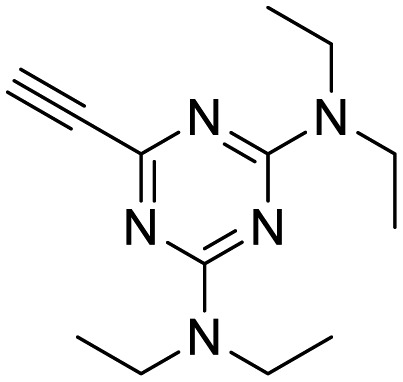 **25**	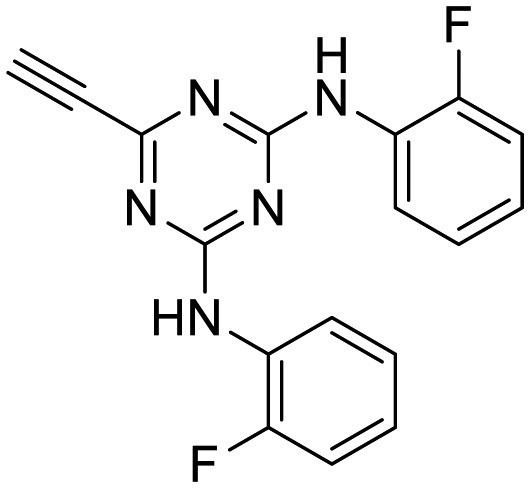 **26**	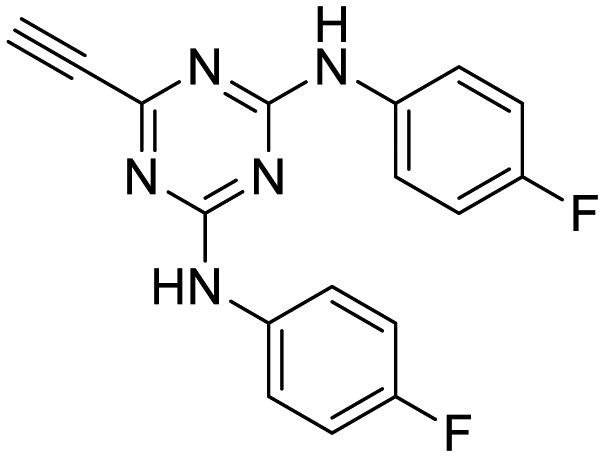 **27**

^*a*^Arranged in order of increasing molecular weight.

**Table 2:** **Group I** click products of azidophenylsulfoxides **10** and azidophenyl sulfones **13** with selected arylacetylenes **16–24**

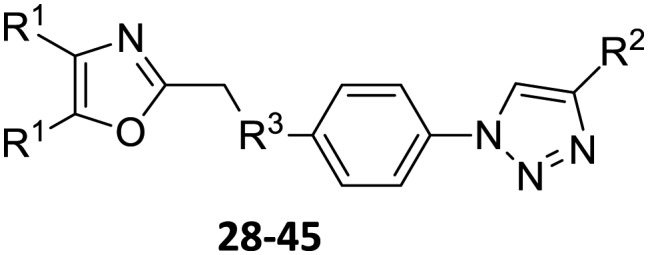	
Compound	Yield^*a*^ (%)
**28**, R^1^ = Ph, R^2^ = 3-pyridyl, R^3^ = SO	46
**29**, R^1^ = Ph, R^2^ = 4-fluorophenyl, R^3^ = SO	86
**30**, R^1^ = Ph, R^2^ = 3-fluorophenyl, R^3^ = SO	97
**31**, R^1^ = Ph, R^2^ = 2-methoxyphenyl, R^3^ = SO	95
**32**, R^1^ = Ph, R^2^ = 2-(trifluoromethyl)phenyl, R^3^ = SO	60
**33**, R^1^ = Ph, R^2^ = 6-methoxynaphthalene-2-yl, R^3^ = SO	58
**34**, R^1^ = Ph, R^2^ = 4-(*n*-pentyl)phenyl, R^3^ = SO	39
**35**, R^1^ = Ph, R^2^ = 4-(phenoxy)phenyl, R^3^ = SO	79
**36**, R^1^ = Ph, R^2^ = 3,5-di-(trifluoromethyl)phenyl, R^3^ = SO	89
**37**, R^1^ = Ph, R^2^ = 3-pyridyl, R^3^ = SO_2_	73
**38**, R^1^ = Ph, R^2^ = 4-fluorophenyl, R^3^ = SO_2_	63
**39**, R^1^ = Ph, R^2^ = 3-fluorophenyl, R^3^ = SO_2_	69
**40**, R^1^ = Ph, R^2^ = 2-methoxyphenyl, R^3^ = SO_2_	98
**41**, R^1^ = Ph, R^2^ = 2-(trifluoromethyl)phenyl, R^3^ = SO_2_	54
**42**, R^1^ = Ph, R^2^ = 6-methoxynaphthalene-2-yl, R^3^ = SO_2_	97
**43**, R^1^ = Ph, R^2^ = 4-(*n*-pentyl)phenyl, R^3^ = SO_2_	77
**44**, R^1^ = Ph, R^2^ = 4-(phenoxy)phenyl, R^3^ = SO_2_	97
**45**, R^1^ = Ph, R^2^ = 3,5-di-(trifluoromethyl)phenyl, R^3^ = SO_2_	63

^*a*^Yields are for isolated pure compounds.

**Scheme 3:**
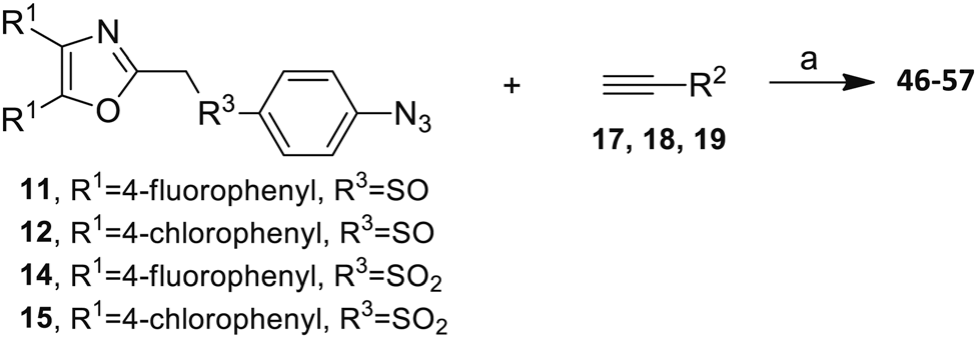
Click reactions of halogenated azidomethylsulfoxides **11** and **12** and azidophenylsulfones **14** and **15** with selected arylacetylenes **17–19** to give **Group II** click products **46–57**. ^a^Reagents/conditions: CuSO_4_·5H_2_O/Na ascorbate/THF–H_2_O (2 : 1).

The 2-substituted-4,5-diphenyloxazole azide units containing the 4,5-di-(4-chlorophenyl) and 4,5-di-(4-fluorophenyl) groups (**11**, **14** and **12**, **15**) offered hydrophobic interactions at both aryl positions in the NITVK-trisubstituted oxazole-associated locus and comprised the next group (**Group II**) of triazole-linked phenyl sulfoxides and sulfones (Scheme 3). Therefore, the combination of azides **11**, **12**, **14**, **15** and arylacetylenes **17–19** (Table 1) gave click products which carried the complete array of halogenated (F, Cl) substituents on both the aryl rings of the 4,5-diaryloxazole (NITVK) and the aryltriazole (VXXLL) loci. The 4,5-bis(4-fluorophenyl)oxazole sulfoxide **11** and the azido 4,5-bis(fluorophenyl) oxazole sulfone **14** together with the corresponding azido bis-(4-chlorophenyl) sulfoxide and sulfone **12**, **15** (from Scheme 1) were reacted with arylacetylenes **17–19** to afford the sulfinyl and sulfonyl-linked triazole click products **46–57** in yields ranging from 52% to 93% (Scheme 3). The click reaction between azides **11**, **12**, **14** and **15** and the group of arylacetylenes **17–19** were conducted under the same conditions as those detailed in Scheme 2. The yields of the triazole click products **46–57** are presented in Table 3.

**Table 3:** **Group II** click products **46–57** of halogenated azidomethylsulfoxides **11**, **12** and azidophenyl sulfones **14**, **15** with selected arylacetylenes **17–19**

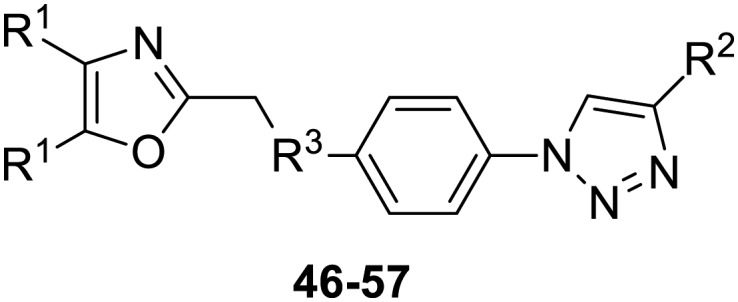	
Compound	Yield^*a*^ (%)
**46**, R^1^ = 4-fluorophenyl, R^2^ = 3-fluorophenyl, R^3^ = SO	78
**47**, R^1^ = 4-fluorophenyl, R^2^ = 4-fluorophenyl, R^3^ = SO	83
**48**, R^1^ = 4-fluorophenyl, R^2^ = 2-methoxypheny, R^3^ = SO	92
**49**, R^1^ = 4-chlororophenyl, R^2^ = 3-fluorophenyl, R^3^ = SO	52
**50**, R^1^ = 4-chlorophenyl, R^2^ = 4-fluorophenyl, R^3^ = SO	93
**51**, R^1^ = 4-chlorophenyl, R^2^ = 2-methoxyphenyl, R^3^ = SO	76
**52**, R^1^ = 4-fluorophenyl, R^2^ = 3-fluorophenyl, R^3^ = SO_2_	79
**53**, R^1^ = 4-fluorophenyl, R^2^ = 4-fluorophenyl, R^3^ = SO_2_	83
**54**, R^1^ = 4-fluorophenyl, R^2^ = 2-methoxyphenyl, R^3^ = SO_2_	95
**55**, R^1^ = 4-chlororophenyl, R^2^ = 3-fluorophenyl, R^3^ = SO_2_	84
**56**, R^1^ = 4-chlorophenyl, R^2^ = 4-fluorophenyl, R^3^ = SO_2_	93
**57**, R^1^ = 4-chlorophenyl, R^2^ = 2-methoxyphenyl, R^3^ = SO_2_	81

^*a*^Yields are for isolated pure compounds.

**Scheme 4:**
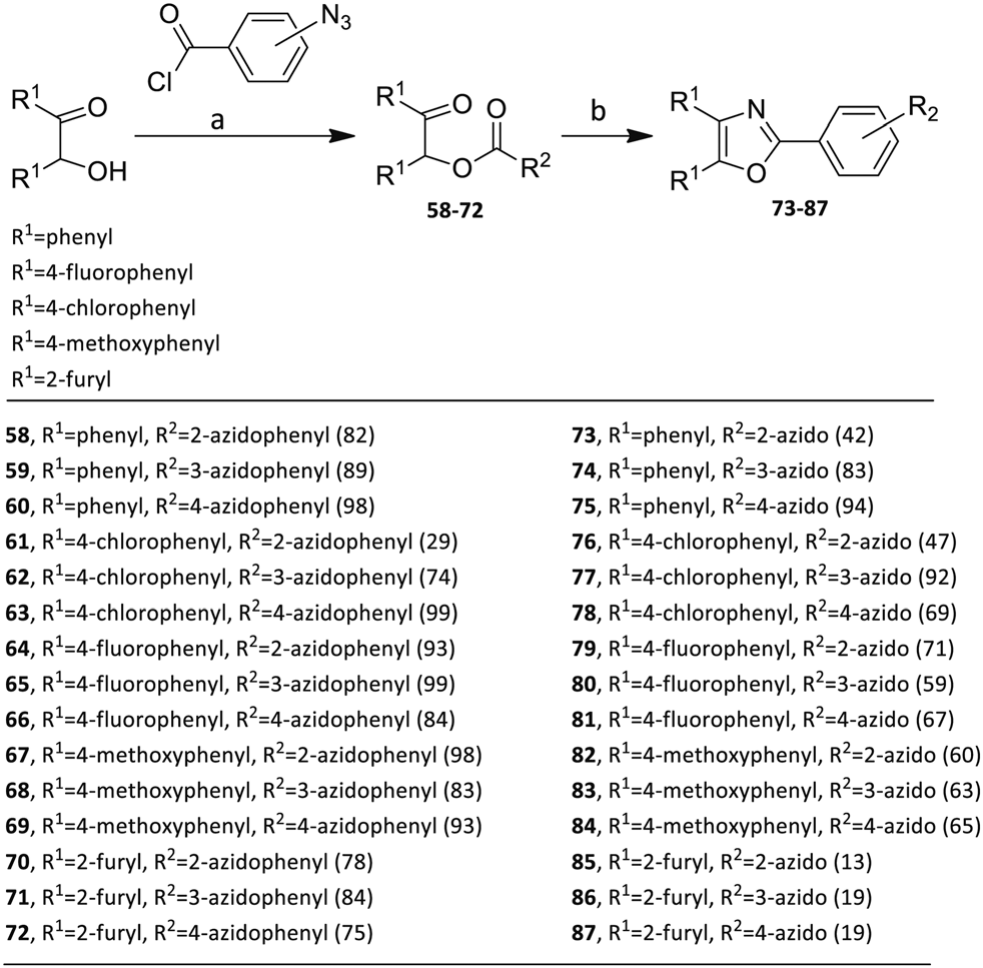
Synthesis of 2-(2-azidophenyl)-, 2-(3-azidophenyl)- and 2-(4-azidophenyl)-4,5-diaryloxazole click partners **73–87** through benzoin esters **58–72**. Reagents/conditions: (a) DMAP/DCM/16 h. (b) NH_4_OAc/HOAc/115–120 °C/3–4 h. Yields are in parentheses.

**Scheme 5:**
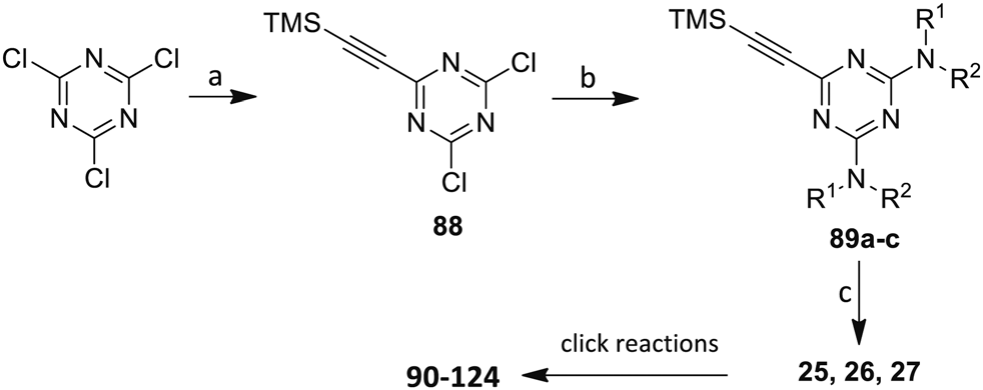
Synthesis of the acetylenic triazine click partners **25–27**. Reagents/conditions: (a) ethynyltrimethylsilane/*n*-BuLi/THF/0 °C/1 h; (b) 4-fluoroaniline, 2-fluoroaniline or diethylamine/THF/0 °C to rt/16 h; (c) TBAF/THF/0 °C to rt/2 h.

**Scheme 6:**
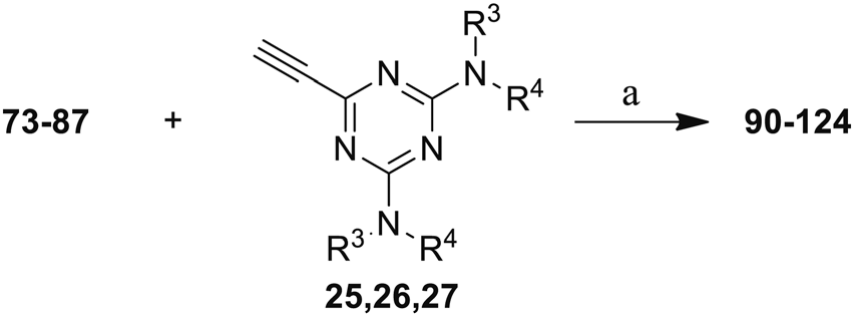
Click reactions of azides **73–87** with acetylenes **25–27** to give **Group III** triazole products **90–124**. ^a^Conditions/reagents: CuSO_4_·5H_2_O/Na ascorbate/THF–H_2_O (2 : 1).

The third group (**Group III**) of second-generation inhibitors includes a more varied array of the VXXLL sectors which are based on the 1,3,5-trisubstituted-2,4,6-triazine motif put forth by Katzenellenbogen and others^29^ in their work on mimicking helical peptide NR boxes containing suitably juxtaposed leucine residues. Thus, the acetylenic triazine click partners **25–27** (from Table 1) are constructs which have the moderately hydrophobic dialkylamino as well as the highly hydrophobic fluorophenylamino groups at positions 3 and 5 of the 2,4,6-triazine.^30^–^33^ Furthermore, the utilization of halogens and halogen isosteres as substituents on the aryl rings of the NITVK/oxazole framework has been commented on in our previous *in vitro* and *in vivo* studies,^12^,^13^ and as a result we decided on the array of azide coupling partners bearing halogenated and methoxy substitution **73–87** (Scheme 4) to be coupled with the acetylenic triazines **25–27**. The preparation of the azidophenyloxazole-based click partners **73–87** which were reacted with the acetylenic triazines **25–27** is detailed in Scheme 4. The appropriate benzoin, where R^1^ = phenyl, 4-fluorophenyl, 4-chlorophenyl, 4-methoxyphenyl or 2-furyl, was reacted with 2-azido-, 3-azido- or 4-azidobenzoyl chloride in the presence of 4-dimethylaminopyridine to give the corresponding benzoin esters **58–72**. The yields ranged from 29% to 99% after purification by column chromatography on silica gel. Cyclization of the benzoin esters **58–72** to the corresponding 4,5-diaryl-2-azidophenyloxazoles **73–87** was accomplished by heating the benzoin esters with ammonium acetate in acetic acid (115–120 °C). The 4,5-diaryloxazoles **73–87** having 2-azidophenyl, 3-azidophenyl or 4-azidophenyl substituents at the 2-position of the oxazole ring were purified by silica gel column chromatography and provided crystalline products in yields ranging from 13% to 94% with the cyclization of the 2-furyl esters giving the lowest yields. The acetylenic triazine click partners **25–27** which comprised the VXXLL scaffold portion of click products **90–124** (Table 4) were prepared from 2,4,6-trichloro-1,3,5-triazine (cyanuric chloride) (Scheme 5). Lithioethynyltrimethylsilane, formed from ethynyltrimethylsilane and *n*-butyllithium (THF/0–5 °C), was added to cyanuric chloride to give 2,4-dichloro-6-((trimethylsilyl)ethynyl)-1,3,5-triazine **88** (0 °C → rt/3 h). Direct addition of excess amine (diethylamine, 2-fluoroaniline or 4-fluoroaniline) to **88** followed by stirring (0 °C → rt/16 h) gave the TMS-acetylenic 3,5-diaminotriazines **89a–c** which were purified by column chromatography. Removal of the trimethylsilyl group was accomplished with tetra-*n*-butylammonium fluoride (TBAF/THF/0 °C → rt) which afforded the acetylenic triazines **25–27**. The click reactions of azides **73–87** with the arylacetylenic substrates **25–27** were conducted using the same copper(ii) sulfate/sodium ascorbate protocol with tetrahydrofuran/water as a solvent system (Scheme 6). The regiochemistry of the dipolar cycloaddition ‘click’ reaction affords products in which the substitution could be 1,4- or 1,5- on the triazole ring depending on the reagents and the conditions used. The click reactions afforded the corresponding 4,5-diaryloxazolyl-1,2,3-triazoles **90–124** as solid compounds with the expected 1,4-regiochemistry. Included with our **Group III** candidates are a new set of compounds **98**, **99**, **108**, **109**, and **122–124** (Table 4) where both phenyl rings at the 4,5-positions of the oxazole were substituted with furan rings. Replacement of benzene with furan is not an uncommon example of effective bioisosteric replacement in medicinal chemistry.^34^ The furan system is not only π-electron rich but also carries a mild hydrogen-bonding component along with its aromatic structure. The yields of the triazole click products **90–124** are listed in Table 4. The oxazole and triazole motifs in the backbone of our peptidomimetic inhibitor candidates offer rigidity as well as the potential for hydrogen bonding. Since the introduction of click chemistry, the 1,2,3-triazole has been effectively deployed as an easily formed peptide bond isostere thereby linking complex polypeptides as well as sectors of less complex peptidomimetics.^35^,^36^ A similar case is made for the oxazole/NITVK scaffold whereby its conferred rigidity influences the disposition of the distal 4,5-(halogenated) diphenyl residues.^37^ All of the click products described herein (Tables 2–4) were formed as a result of copper(i) catalysis which gave exclusively the 1,4- or “anti”-substituted 1,2,3-triazole.^21^

**Table 4:** **Group III** click products of azides **73–87** and acetylenes **25–27**

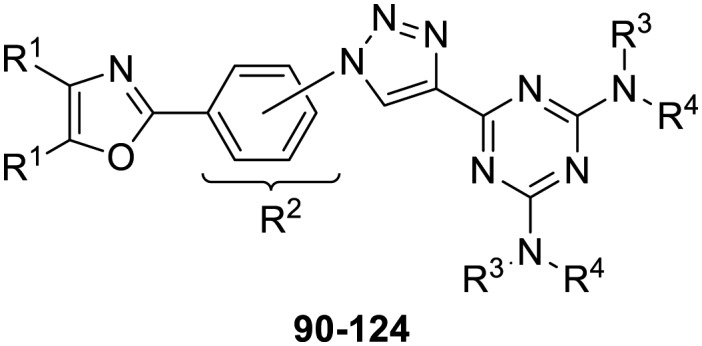	
Compound	Yield^*a*^ (%)
**90**, R^1^ = phenyl, R^2^ = 3-triazolyl, R^3^, R^4^ = ethyl	76
**91**, R^1^ = phenyl, R^2^ = 4-triazolyl, R^3^, R^4^ = ethyl	90
**92**, R^1^ = 4-fluorophenyl, R^2^ = 3-triazolyl, R^3^, R^4^ = ethyl	48
**93**, R^1^ = 4-fluorophenyl, R^2^ = 4-triazolyl, R^3^, R^4^ = ethyl	45
**94**, R^1^ = 4-chlorophenyl, R^2^ = 3-triazolyl, R^3^, R^4^ = ethyl	52
**95**, R^1^ = 4-chlorophenyl, R^2^ = 4-triazolyl, R^3^, R^4^ = ethyl	57
**96**, R^1^ = 4-methoxyphenyl, R^2^ = 3-triazolyl, R^3^, R^4^ = ethyl	53
**97**, R^1^ = 4-methoxyphenyl, R^2^ = 4-triazolyl, R^3^, R^4^ = ethyl	63
**98**, R^1^ = 2-furyl, R^2^ = 3-triazolyl, R^3^, R^4^ = ethyl	52
**99**, R^1^ = 2-furyl, R^2^ = 4-triazolyl, R^3^, R^4^ = ethyl	46
**100**, R^1^ = phenyl, R^2^ = 3-triazolyl, R^3^ = 2-fluorophenyl, R^4^ = H	80
**101**, R^1^ = phenyl, R^2^ = 4-triazolyl, R^3^ = 2-fluorophenyl, R^4^ = H	65
**102**, R^1^ = 4-fluorophenyl, R^2^ = 3-triazolyl, R^3^ = 2-fluorophenyl, R^4^ = H	95
**103**, R^1^ = 4-fluorophenyl, R^2^ = 4-triazolyl, R^3^ = 2-fluorophenyl, R^4^ = H	77
**104**, R^1^ = 4-chlorophenyl, R^2^ = 3-triazolyl, R^3^ = 2-fluorophenyl, R^4^ = H	70
**105**, R^1^ = 4-chlorophenyl, R^2^ = 4-triazolyl, R^3^ = 2-fluorophenyl, R^4^ = H	69
**106**, R^1^ = 4-methoxyphenyl, R^2^ = 3-triazolyl, R^3^ = 2-fluorophenyl, R^4^ = H	66
**107**, R^1^ = 4-methoxyphenyl, R^2^ = 4-triazolyl, R^3^ = 2-fluorophenyl, R^4^ = H	75
**108**, R^1^ = 2-furyl, R^2^ = 3-triazolyl, R^3^ = 2-fluorophenyl, R^4^ = H	69
**109**, R^1^ = 2-furyl, R^2^ = 4-triazolyl, R^3^ = 2-fluorophenyl, R^4^ = H	83
**110**, R^1^ = phenyl, R^2^ = 2-triazolyl, R^3^ = 4-fluorophenyl, R^4^ = H	30
**111**, R^1^ = phenyl, R^2^ = 3-triazolyl, R^3^ = 4-fluorophenyl, R^4^ = H	82
**112**, R^1^ = phenyl, R^2^ = 4-triazolyl, R^3^ = 4-fluorophenyl, R^4^ = H	48
**113**, R^1^ = 4-fluorophenyl, R^2^ = 2-triazolyl, R^3^ = 4-fluorophenyl, R^4^ = H	77
**114**, R^1^ = 4-fluorophenyl, R^2^ = 3-triazolyl, R^3^ = 4-fluorophenyl, R^4^ = H	87
**115**, R^1^ = 4-fluorophenyl, R^2^ = 4-triazolyl, R^3^ = 4-fluorophenyl, R^4^ = H	26
**116**, R^1^ = 4-chlorophenyl, R^2^ = 2-triazolyl, R^3^ = 4-fluorophenyl, R^4^ = H	83
**117**, R^1^ = 4-chlorophenyl, R^2^ = 3-triazolyl, R^3^ = 4-fluorophenyl, R^4^ = H	55
**118**, R^1^ = 4-chlorophenyl, R^2^ = 4-triazolyl, R^3^ = 4-fluorophenyl, R^4^ = H	51
**119**, R^1^ = 4-methoxyphenyl, R^2^ = 2-triazolyl, R^3^ = 4-fluorophenyl, R^4^ = H	43
**120**, R^1^ = 4-methoxyphenyl, R^2^ = 3-triazolyl, R^3^ = 4-fluorophenyl, R^4^ = H	77
**121**, R^1^ = 4-methoxyphenyl, R^2^ = 4-triazolyl, R^3^ = 4-fluorophenyl, R^4^ = H	67
**122**, R^1^ = 2-furyl, R^2^ = 2-triazolyl, R^3^ = 4-fluorophenyl, R^4^ = H	49
**123**, R^1^ = 2-furyl, R^2^ = 3-triazolyl, R^3^ = 4-fluorophenyl, R^4^ = H	69
**124**, R^1^ = 2-furyl, R^2^ = 4-triazolyl, R^3^ = 4-fluorophenyl, R^4^ = H	68

^*a*^Yields are for isolated purified compounds.

### 2.2. Bioassay results and discussion

**Fig. 2:**
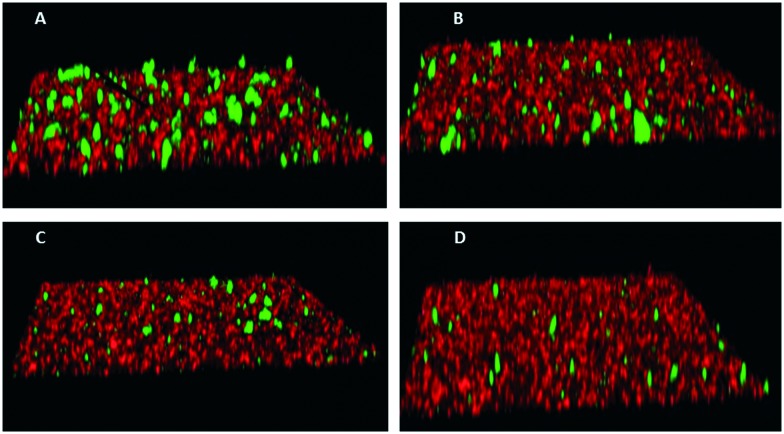
Inhibition of *P. gingivalis* (green) adherence to *S. gordonii* (red) by compound **111**. Biofilms were formed in the presence of PBS/0.1% DMSO (A), or 5 μM (B), 20 μM (C), or 40 μM (D) compound **111**.

To assess the functional activity of the click products, compounds **28–57** (**Groups I** and **II**) and **90–124** (**Group III**) were examined for inhibition of *P. gingivalis* adherence to *S. gordonii* using an established biofilm model system as described in section 4.2.1.^38^ A representative dose response series of inhibition of *P. gingivalis* adherence for compound **111** is shown in Fig. 2. For each compound tested, an IC_50_ value for adherence inhibition was approximated after quantifying the ratio of green to red fluorescence and these values are shown in Table 5. Eleven compounds exhibited IC_50_ values <10 μM and the most potent of these exhibited IC_50_ values that were approximately 2.5-fold lower than the most potent of the previously reported generation 1 compounds.^12^,^13^ Compounds **54**, **56**, **93**, **95**, **102**, **103**, **111**, **115** and **122** were also tested to determine if they affect the planktonic growth of *P. gingivalis* or *S. gordonii*. As shown in Table 6, little effect on planktonic growth of *P. gingivalis* or *S. gordonii* cultures was observed for any of the compounds tested when cultures were incubated overnight in the presence of 40 μM compound. For the four most potent compounds (**95**, **111**, **115** and **122**), this concentration is approximately 10- to 20-fold greater than the IC_50_ values determined for inhibition of adherence. This indicates that the compounds do not function as antibiotics but rather specifically inhibit *P. gingivalis* adherence to streptococci. Indeed, our goal is to develop targeted approaches to prevent colonization of the oral cavity by the periodontal pathogen *P. gingivalis*. The limited distribution of the Mfa protein in other oral bacteria will prevent these compounds from acting on other commensal oral bacteria and compounds that target Mfa function will likely be highly selective agents. In comparing and contrasting the six compounds which exhibit the highest inhibitory activity, five (**95**, **102**, **111**, **115**, and **122**) are in **Group III** and one (**46**) is in **Group II**. Compound **46** (IC_50_, 5.3 μM) is the only candidate which possesses a sulfoxide group as part of the backbone as well as an *m*-fluorophenyl group on the triazole linker which mimics the VXXLL motif. Similar to two other highly active compounds **102** (5.0 μM) and **115** (2.3 μM), the NITVK-mimic sector is the 4,5-di-(4-fluorophenyl)oxazole. The sulfoxide group does impart asymmetry to the molecule; however, the mixture of enantiomers, assuming the peracid oxidation was racemic, was not separated and bioassayed. The **Group III** active compounds (**95–117**) all contain the 1,3,5-trisubstituted-2,4,6-triazine modeled after the Rodriguez *et al.* rendering of an active motif whereby the key leucine residues which mimicked the VXXLL receptor box lie in a triangular arrangement.^29^ While the original iteration of the Katzenellenbogen VXXLL mimics were the hydrophobic 3-disubstituted-di-*N*-alkylamino-triazine motifs, we introduced the hydrophobic 2- and 4-(fluorophenyl)amino groups at positions 3 and 5 of the 2,4,6-triazine (see compounds **102**, **111**, **115**, and **122**). Compound **95**, however (IC_50_ = 3.7 μM), contains the more closely related, nonaromatic lipid-like diethylamino groups at positions 3 and 5 of the 2,4,6-triazine motif. The deployment of furan rings at the 4,5-positions of the oxazole in compounds **98**, **99**, **108**, **109**, and **122–124** led to only one compound **122** (IC_50_ 2.4 μM) of significant activity. Interestingly, along with the di-furyloxazole arrangement, candidate **122** possessed the 3,5-di-(4-fluorophenyl)aminotriazine, and was also the compound which exhibited the most bent conformation by nature of the *ortho* disposition of the oxazole to the triazole linker.

**Table 5:** IC_50_ values for **Group I** compounds (**28–45**), **Group II** compounds (**46–57**) and **Group III** compounds (**90–124**)

**Group I**	IC_50_^*a*^ (μM)	**Group II**	IC_50_^*a*^ (μM)	**Group III**	IC_50_^*a*^ (μM)
**28**	NA	**46**	5.30	**90**	20.7
**29**	NA	**47**	35.9	**91**	NA
**30**	47.3	**48**	7.20	**92**	NA
**31**	NA	**49**	15.1	**93**	9.4
**32**	NA	**50**	39.8	**94**	16.0
**33**	NA	**51**	10.2	**95**	3.7
**34**	NA	**52**	40.0	**96**	NA
**35**	NA	**53**	40.0	**97**	36.6
**36**	NA	**54**	21.2	**98**	NA
**37**	NA	**55**	18.2	**99**	NA
**38**	15.7	**56**	6.3	**100**	9.6
**39**	33.6	**57**	20.0	**101**	NA
**40**	13.1			**102**	5.00
**41**	NA			**103**	12.3
**42**	NA			**104**	33.8
**43**	36.6			**105**	NA
**44**	16.5			**106**	NA
**45**	38.5			**107**	40.0
				**108**	NA
				**109**	NA
				**110**	NA
				**111**	4.3
				**112**	NA
				**113**	49.2
				**114**	NA
				**115**	2.3
				**116**	>40.0
				**117**	27.8
				**118**	13.3
				**119**	NA
				**120**	NA
				**121**	NA
				**122**	2.4
				**123**	8.00
				**124**	7.8

^*a*^NA = not active.

**Table 6:** Inhibition of planktonic growth

Compound	IC_50_ (μM)	Percent inhibition of:	
		*P. gingivalis*	*S. gordonii*
**Tet** ^*a*^		95.7 ± 1.2	94.2 ± 1.7
**95**	3.7	0.4 ± 6.1	–0.8 ± 2.8
**111**	4.3	8.3 ± 3.6	2.9 ± 3.9
**115**	2.3	–2.8 ± 6.4	–0.6 ± 5.8
**122**	2.4	2.5 ± 5.4	–0.6 ± 3.9
**54**	21.2	–3.4 ± 10.4	–3.3 ± 6.0
**56**	6.3	–2.7 ± 6.6	6.2 ± 8.8
**93**	9.4	2.0 ± 6.1	–2.0 ± 8.9
**102**	5.0	–8.3 ± 5.2	–0.6 ± 0.8
**103**	12.3	–1.3 ± 14.2	–8.6 ± 2.0

^*a*^Inhibition of planktonic growth by 10 μg ml^–1^ tetracycline.

#### 2.2.1. Cytotoxicity of compounds **95**, **111**, **115** and **122**

**Fig. 3:**
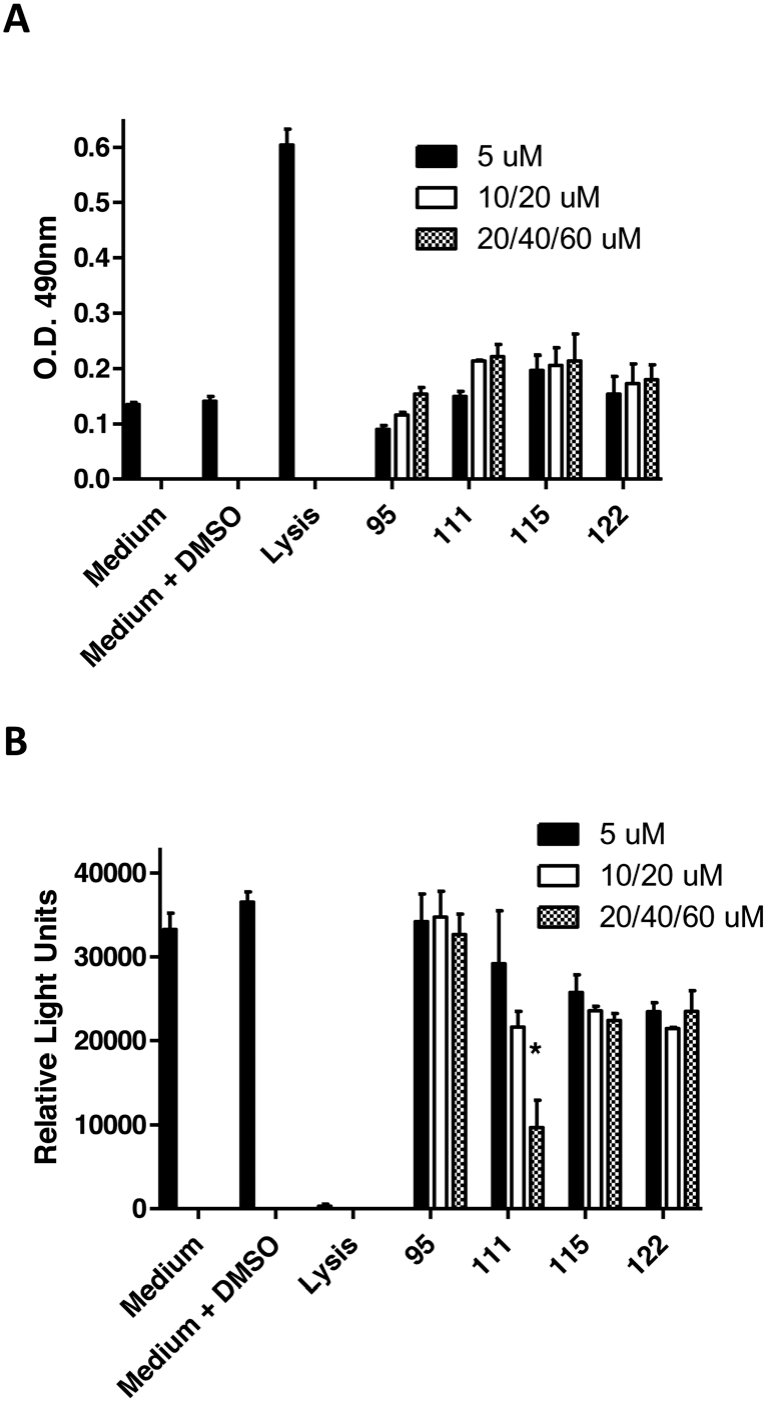
Release of lactate dehydrogenase (LDH) activity (A) and measurement of ATP levels (B) in TIGK cells after 18 h exposure to peptidomimetic compounds. Concentrations of compounds used are as follows: **95** (5, 20 and 40 μM), **111** (5, 20, 60 μM), **115** (5, 10, 20 μM) and **122** (5, 20, 60 μM). The asterisk indicates a significant decrease in ATP relative to cells exposed to medium containing 0.1% DMSO; *p < 0.05.

To initially assess the cytotoxicity of the most active compounds, telomerase immortalized gingival keratinocytes (TIGK cells) were incubated with each compound and the release of lactate dehydrogenase (LDH) into the culture medium was followed as a measure of cell lysis. In addition, the level of cellular metabolism was determined by measuring ATP levels. As shown in Fig. 3A and B, lysis of TIGK cells results in a significant increase in LDH release relative to the medium only or medium/DMSO controls. None of the compounds induced a significant increase in LDH release at any of the tested concentrations. Lysis of TIGK cells also resulted in a significant decrease in cellular ATP levels (Fig. 3B). Incubation of cells with compounds **95**, **115** or **122** did not significantly reduce the levels of ATP. In contrast, incubation of TIGK cells with compound **111** appeared to result in a dose-dependent reduction of ATP levels; however, only the reduction observed at 60 μM was statistically significant. These results suggest that with the possible exception of compound **111**, the four most potent inhibitors of *P. gingivalis* adherence to oral streptococci exhibit little cytotoxic activity against gingival epithelial cells. It should also be noted that the cytotoxic activity of compound **111** was only observed at a concentration that is approximately 15-fold greater than its IC_50_ for inhibition of *P. gingivalis* adherence.

## 3. Conclusions

We have prepared a number of newer “second-generation” click compounds which are effective inhibitors of *P. gingivalis* adherence to oral streptococci. While we noted previously that lead optimization of our first-generation inhibitors could occur through adjustment/positioning of the hydrophobic groups of the mono-aryl and diaryloxazole scaffolds, we instead opted to change the nature of the linkers conjoining the two scaffolds and keep the aryl substitution constant. The new linkers comprised both sulfonyl and sulfoxide groups. Through exploring the diaminotriazine motif, changes were also made to optimize the VXXLL sectors of the general inhibitor scaffold. Four of these “second-generation” compounds (**95**, **111**, **115** and **122**) were shown to be more potent inhibitors of *P. gingivalis* adherence than our “first-generation” compounds. Of these, only compound **111** exhibited cytotoxicity against TIGK cells and this was observed only at the highest concentration tested. As in the development of the first-generation compounds, the evaluation of this new series of candidates in pertinent *in vivo* animal studies will be reported in due course.

## 4. Experimental

### 4.1. Chemistry

Unless otherwise specified, all solvents and reagents were ACS grade and were used as supplied. Solvents were removed from reaction mixtures and extracts using standard Büchi rotary evaporators under a water aspirator vacuum. Alkynes **16–24** were commercially available and were used as supplied. All melting points (mp) were determined using a Thomas-Hoover apparatus. Infrared spectra were recorded on a Perkin-Elmer Spectrum Version 10.02.00 instrument and absorptions are reported as reciprocal centimeters (cm^–1^). Proton (^1^H) and carbon (^13^C) NMR spectra were recorded on a Varian INOVA instrument (400 MHz, 100 MHz for ^13^C) or a Bruker instrument (500 MHz, 125 MHz for ^13^C). High-resolution mass spectra (HRMS) were recorded using electrospray ionization (ESI). All air- and moisture-sensitive reactions were run in oven-dried glassware under an atmosphere of dry nitrogen. Gravity-column chromatography was performed on silica gel 60 (E. Merck, 7734, 70–230 mesh). Thin-layer chromatography (TLC) was performed with glass-backed plates (E. Merck, 5715, silica gel 60 F_254_ 2.5 mm thickness) and visualized using 2% anisaldehyde in ethanol, 2.5% phosphomolybdic acid in ethanol or 10% sulfuric acid in ethanol.

#### 4.1.1. General procedure for the synthesis of aminophenylsulfides **4–6**

To a suspension of freshly washed sodium hydride (1.2 equiv.) in dry THF (15 mL) was added 4-aminothiophenol (1.0 equiv.) under a nitrogen atmosphere at 0–5 °C followed by stirring for 30 min. To this mixture was added dropwise a solution of compounds **1–3** (1.1 equiv.) dissolved in THF (15 mL) at 0–5 °C. After the addition was complete, the reaction temperature was gradually raised to room temperature and allowed to stir (16 h). After completion of the reaction, as indicated by TLC (hexane/ethyl acetate, 3 : 1), the reaction mixture was quenched with cold water (25 mL). The resulting reaction mixture was extracted into dichloromethane (2 × 25 mL) and the organic layers were combined, dried over anhydrous sodium sulfate, filtered and evaporated. The obtained off-white crude residue was submitted to silica gel column chromatography (hexanes/ethyl acetate, 3 : 1) to give aminophenylsulfides **4–6** as pure off-white products.

##### 4.1.1.1. 4-(((4,5-Diphenyloxazol-2-yl)methyl)thio)aniline (**4**)

Light yellow solid; yield 55%; mp = 131–133 °C; *R*_f_ = 0.17 (hexane/ethyl acetate, 7 : 3); FT-IR: 2125, 2090, 1591, 1487, 1291 cm^–1^; ^1^H NMR (500 MHz, CDCl_3_): *δ* 7.61 (dd, *J* = 1.5 Hz, 3.5 Hz, 2H), 7.53 (dd, *J* = 2.0 Hz, 4.0 Hz, 2H), 7.37–7.29 (m, 8H), 6.60 (dd, *J* = 2.0 Hz, 6.5 Hz, 2H), 4.07 (s, 2H), 3.75 (s, 2H) ppm; ^13^C NMR (125 MHz, CDCl_3_): *δ* 160.1, 147.0, 145.8, 135.5 (overlap), 132.3, 128.5 (overlap), 128.0 (overlap), 127.9 (overlap), 126.4 (overlap), 121.2, 115.4 (overlap), 33.7 ppm; HRMS (+ESI): calcd for [C_22_H_18_N_2_O_5_] 359.1218, found 359.1267 ([M + H]^+^).

#### 4.1.2. General procedure for the synthesis of azidophenylsulfides **7–9**

To a pre-chilled solution of compounds **4–6** (1.0 equiv.) in aqueous HCl (4 N) was added dropwise a chilled aqueous solution of sodium nitrite (1.1 equiv./10 mL/H_2_O). The resulting yellowish reaction mixture was allowed to stir at 0–5 °C (1 h). To this clear solution, was added dropwise an aqueous solution of sodium azide (1.05 equiv./10 mL/H_2_O) and stirring was continued (16 h) at room temperature. The progress of the reaction was monitored by TLC (hexane/ethyl acetate). After completion of the reaction, the reaction mixture was extracted into dichloromethane (2 × 25 mL) and organic layers were combined. The organic layer was washed with saturated NaHCO_3_ solution (30 mL), separated, dried over anhydrous sodium sulfate and filtered. Concentration of the filtrate gave the crude residue of the corresponding products which was submitted to column chromatography (hexane/ethyl acetate, 3 : 1) to give the pure azidophenyl sulfides **7–9**.

##### 4.1.2.1. 2-(((4-Azidophenyl)thio)methyl)-4,5-diphenyloxazole (**7**)

Light brown solid; yield 87%; mp = 64–65 °C; *R*_f_ = 0.5 (hexane/ethyl acetate, 7.5 : 2.5); FT-IR: 2125, 2090, 1591, 1487, 1291 cm^–1^; ^1^H NMR (400 MHz, CDCl_3_): *δ* 7.59 (d, *J* = 8.0 Hz, 2H), 7.53–7.50 (m, 3H), 7.48 (s, 1H), 7.38–7.34 (m, 6H), 6.98 (d, *J* = 8.4 Hz, 2H), 4.19 (s, 2H) ppm; ^13^C NMR (125 MHz, CDCl_3_): *δ* 159.3, 145.9, 139.6, 135.3, 133.3, 132.0, 130.4, 128.5, 128.4, 128.1, 127.8, 126.3, 119.5, 31.9 ppm; HRMS (+ESI) *m*/*z* calcd for [C_22_H_16_N_4_OS]^+^ 385.1123, found 385.1133 ([M + H]^+^).

#### 4.1.3. General procedure for the synthesis of azidophenyl sulfoxides and sulfones **10–15**

To a clear solution of compounds **7–9** (1.0 equiv.) in dichloromethane (30 mL) was added *m*-CPBA (1.2 equiv. for synthesis of sulfoxides **10–12** and 3.0 equiv. for sulfones **13–15**) and the resulting solution was stirred for 16 h at room temperature. The reaction progress was monitored by TLC (hexane/ethyl acetate). After completion of the reaction, the reaction mixture was washed with saturated aqueous NaHCO_3_ solution and the organic layer was separated and dried over anhydrous sodium sulfate. Concentration of the organic layer provided an off-white residue of the corresponding sulfoxides and sulfones which were submitted to column chromatography (hexane/ethyl acetate) to afford the pure azidophenyl sulfoxides and sulfones **10–15**.

##### 4.1.3.1. 2-(((4-Azidophenyl)sulfinyl)methyl)-4,5-diphenyloxazole (**10**)

Colorless oil; yield 86%; *R*_f_ = 0.1 (hexane/ethyl acetate, 3 : 1); FT-IR: 3055, 2125, 2090, 1586, 1051 cm^–1^; ^1^H NMR (400 MHz, CDCl_3_): *δ* 7.60 (d, *J* = 8.4 Hz, 2H), 7.57–7.55 (m, 2H), 7.47–7.45 (m, 2H), 7.39–7.34 (m, 6H), 7.14 (d, *J* = 8.4 Hz, 2H), 4.41 (d, *J* = 13.2 Hz, 1H), 4.21 (d, *J* = 13.2 Hz, 1H) ppm; ^13^C NMR (175 MHz, CDCl_3_): *δ* 153.1, 147.1, 144.0, 138.8, 136.1, 131.7, 129.0, 128.7, 128.6, 128.4, 126.1, 127.8, 126.5, 126.1, 119.8, 56.2 ppm; HRMS (+ESI) *m*/*z* calcd for [C_22_H_16_N_4_O_2_S]^+^ 401.1072, found 401.1119 ([M + H]^+^).

#### 4.1.4. General procedure for the synthesis of acetylenic triazines **25–27**

To a solution of ethynyltrimethylsilane (1.0 equiv.) in dry THF (5.0 mL) was added *n*-butyllithium (1.6 M solution in hexane, 1.0 equiv.) by syringe at 0 °C under argon while stirring. After stirring for one hour at 0 °C, the resulting solution of lithioethynyltrimethylsilane was cannulated dropwise onto a solution of cyanuric chloride (1.0 equiv.) in dry THF. The resulting viscous red-brown suspension was stirred at 0 °C (2 h) before the corresponding amines (diethylamine, 2-fluoroaniline or 4-fluoroaniline, 2.0 equiv.) were added dropwise at 0 °C. The reaction temperature was gradually increased to room temperature and the mixture was allowed to stir for 48 h. After completion of the reaction, as monitored by TLC, the reaction mixture was quenched with cold water and extracted into dichloromethane (2 × 25 mL). The organic layers were combined, dried over anhydrous sodium sulfate and evaporated to give a crude brownish residue which was further purified by using column chromatography to obtain the pure TMS acetylenic triazines **89a–89c**. To a prechilled solution of the corresponding trimethylsilyl triazines **89a–89c** (1.0 equiv.) in dry THF (25 mL) was slowly added tetra-*n*-butylammonium fluoride (TBAF, 1.0 equiv.) under a nitrogen atmosphere. The resulting brown solution was stirred at 5–10 °C (1 h). The progress of the reaction was monitored by TLC (hexane/ethyl acetate, 9 : 1). After completion of the reaction, cold water (25 mL) was added to the reaction mixture followed by extraction into dichloromethane (2 × 25 mL). The combined organic layers were dried over anhydrous sodium sulfate, filtered and evaporated to obtain a crude residue which was submitted to silica gel column chromatography (hexane/ethyl acetate, 9 : 1) to give the pure acetylenic diaminotriazines **25–27** as solid materials.

##### 4.1.4.1. *N* ^2^,*N*^2^,*N*^4^,*N*^4^-Tetraethyl-6-((trimethylsilyl)ethynyl)-1,3,5-triazine-2,4-diamine (**89a**)

Pale yellow solid; 50% yield; mp = 102–103 °C; *R*_f_ = 0.56 (hexane/ethyl acetate, 3 : 1); FT-IR: 2981, 2964, 2932, 1535, 1493, 1359, 1249, 1079 cm^–1^; ^1^H NMR (400 MHz, CDCl_3_): *δ* 3.60 (s, 4H), 3.53 (d, *J* = 6.4 Hz, 4H), 1.15 (t, *J* = 7.2 Hz, 12H), 0.26 (s, 9H) ppm; ^13^C NMR (125 MHz, CDCl_3_): *δ* 163.7, 158.3, 110.0, 103.6, 90.6, 40.9, 13.5, 12.8, –0.28 ppm; LRMS (+ESI) for [C_16_H_29_N_5_Si], found 319.

##### 4.1.4.2. *N* ^2^,*N*^2^,*N*^4^,*N*^4^-Tetraethyl-6-ethynyl-1,3,5-triazine-2,4-diamine (**25**)

Off-white solid; 59% yield; mp = 47–49 °C; *R*_f_ = 0.33 (hexane/ethyl acetate, 9 : 1); FT-IR: 3228, 2977, 2933, 2112, 1539, 1492, 1355, 1084 cm^–1^; ^1^H NMR (400 MHz, CDCl_3_): *δ* 3.59 (d, *J* = 6.0 Hz, 4H), 3.53 (d, *J* = 6.0 Hz, 4H), 2.87 (s, 1H), 1.15 (t, *J* = 7.2 Hz, 12H) ppm; ^13^C NMR (125 MHz, CDCl_3_): *δ* 163.6, 157.9, 82.7, 72.9, 41.1, 13.5, 12.8 ppm; LRMS (+ESI) for [C_13_H_21_N_5_], found 247.

#### 4.1.5. General procedure for the synthesis of benzoin esters **58–72**

To a clear pre-chilled solution of benzoin, the halogenated benzoins or furoin (1.0 equiv.) in dichloromethane (50 mL) was added DMAP (1.5 equiv.) and stirring was continued (30 min). To the reaction mixture was added dropwise a solution of 2-, 3- or 4-azidobenzoyl chloride (prepared by reacting the corresponding azidocarboxylic acids (1.3 equiv.) with thionyl chloride at 85–90 °C for 2 h) dissolved in dichloromethane (20 mL) under a nitrogen atmosphere. The progress of the reaction was monitored by TLC (hexane/ethyl acetate). After completion of the reaction, the reaction mixture was washed with aqueous HCl (5%, 2 × 50 mL) followed by aqueous NaHCO_3_ (5%). The organic layer was separated, dried over anhydrous sodium sulfate, filtered and evaporated to obtain the crude residue of corresponding azidobenzoyl esters which were purified by column chromatography on gravity silica gel (hexane/ethyl acetate) to give the pure 2-, 3-, 4-azidobenzoyl esters **58–72**.

##### 4.1.5.1. 2-Oxo-1,2-diphenylethyl 2-azidobenzoate (**58**)

Colorless oil; yield 95%; *R*_f_ = 0.4 (hexane/ethyl acetate, 3 : 1); FT-IR: 3065, 2118, 2090, 1714, 1686, 1596, 1487, 1235 cm^–1^; ^1^H NMR (400 MHz, CDCl_3_) *δ* 8.08 (dd, *J* = 1.2 Hz, 7.6 Hz, 1H), 7.98 (dd, *J* = 1.2 Hz, 8.2 Hz, 2H), 7.57–7.51 (m, 4H), 7.44–7.35 (m, 5H), 7.24–7.17 (m, 1H), 7.08 (s, 1H) ppm; ^13^C NMR (100 MHz, CDCl_3_): *δ* 193.6, 164.3, 140.6, 134.7, 133.7, 133.5, 133.4, 132.4, 129.4, 129.2, 128.9, 128.7, 128.6, 124.5, 121.4, 119.8, 78.1 ppm; LRMS for [C_21_H_15_N_3_O_5_], found 358 (M + H).

#### 4.1.6. General procedure for the synthesis of 4,5-diaryl-2-azidophenyloxazoles **73–87**

The azidobenzoyl esters **58–72** (1.0 equiv.) were dissolved in glacial acetic acid (100 mL) at room temperature. To the clear solution was added ammonium acetate (15 equiv.) under a nitrogen atmosphere. The resulting reaction mixture was heated at 115 °C (oil bath temperature) and the temperature was maintained for 3 h. After completion of the reaction, as indicated by TLC, the reaction mixture was cooled to room temperature. Cold water (150 mL) was added to the reaction mixture which was then slowly neutralized with saturated NaHCO_3_ solution. The crude product was extracted with dichloromethane (2 × 50 mL) and the organic extracts were combined, dried over anhydrous sodium sulfate and filtered. Concentration of the dried extracts provided the corresponding crude azido oxazoles which were submitted to silica gel column chromatography (hexane/ethyl acetate) and afforded the pure 4,5-diaryl-2-azidophenyloxazoles **73–87**.

##### 4.1.6.1. 2-(2-Azidophenyl)-4,5-diphenyloxazole (**73**)

Pale yellow solid; 84% yield; *R*_f_ = 0.22 (hexane/ethyl acetate, 9 : 1); FT-IR: 3059, 2121, 2089, 1581, 1501, 1291, 1070 cm^–1^; ^1^H NMR (400 MHz, CDCl_3_) *δ* 8.32 (d, *J* = 7.6 Hz, 1H), 7.94 (dd, *J* = 8.0 Hz, 18.4 Hz, 4H), 7.70 (t, *J* = 7.6 Hz, 1H), 7.65–7.52 (m, 7H), 7.47 (t, *J* = 8.0 Hz, 1H) ppm; ^13^C NMR (100 MHz, CDCl_3_) *δ* 157.8, 145.9, 138.2, 136.7, 134.9, 132.5, 131.4, 130.7, 129.9, 129.0, 128.9, 128.7, 128.69, 128.64, 128.3, 126.6, 124.9, 119.8, 119.2 ppm; HRMS (ESI) *m*/*z* calcd for [C_21_H_15_N_4_O] 339.1240, found 339.1242.

#### 4.1.7. General procedure for the synthesis of the click triazole products **28–57** and **90–124**

To a solution of the group of the azidophenylsulfoxide and sulfone click partners **10–15** and the azidophenyl click partners **73–87** (1.0 equiv.) in anhydrous THF (2.0 mL) were added the requisite acetylenic click partners **16–27** (1.1 equiv.) followed by addition of solid copper sulfate pentahydrate (0.1 equiv.) at room temperature. To the reaction mixture was then added a freshly prepared clear solution of sodium ascorbate (0.5 equiv.) in water (1 mL). The resulting reaction mixture was stirred at room temperature (5–16 h). The progress of the reaction was monitored by TLC using the mobile phases hexane/ethyl acetate and/or chloroform/methanol. After completion of the reaction, the reaction mixture was concentrated and the crude residue was partitioned between dichloromethane (15 mL) and water (10 mL). The organic layer was separated, dried over anhydrous sodium sulfate and concentrated to obtain the crude residue of the click products which were submitted to silica gel column chromatography (hexanes/ethyl acetate, or chloroform/methanol) to give the corresponding pure click product triazoles **28–57** and **90–124**.

##### 4.1.7.1. 4,5-Diphenyl-2-(((4-(4-(pyridin-3-yl)-1*H*-1,2,3-triazol-1-yl)phenyl)sulfinyl)methyl) oxazole (**28**)

Light yellow solid; yield 48%; mp = 193–195 °C; *R*_f_ = 0.26 (methanol/chloroform, 1 : 9); FT-IR (neat): 3085, 3038, 2986, 2929, 1593, 1507, 1404, 1238, 1049, 687 cm^–1^; ^1^H NMR (400 MHz, CDCl_3_): *δ* 9.07 (s, 1H), 8.65 (s, 1H), 8.29 (d, *J* = 8.4 Hz, 1H), 8.19 (s, 1H), 7.96 (d, *J* = 8.4 Hz, 2H), 7.82 (d, *J* = 8.0 Hz, 2H), 7.57–7.55 (m, 1H), 7.47–7.42 (m, 3H), 7.37–7.29 (m, 6H), 4.40 (dd, *J* = 14.0 Hz, 68.4 Hz, 2H) ppm; ^13^C NMR (175 MHz, CDCl_3_): *δ* 152.7, 149.8, 147.2, 147.1, 145.8, 143.7, 139.2, 136.2, 133.2, 131.6, 129.0, 128.72, 128.66, 128.51, 128.0, 127.8, 126.5, 126.1, 126.0, 123.9, 121.1, 117.7, 56.1 ppm; HRMS (+ESI) *m*/*z* calcd for [C_29_H_21_N_5_O_2_S]^+^ 504.1494, found 504.1516 ([M + H]^+^).

### 4.2. Biology

#### 4.2.1. Bacterial strains and culture conditions

*P. gingivalis* ATCC33277 was grown in reduced trypticase soy broth (Difco) supplemented with 0.5 percent yeast extract, 1 μg mL^–1^ menadione, and 5 μg mL^–1^ hemin. Twenty five milliliters of medium were reduced for 24 h under anaerobic conditions by equilibrating in an atmosphere consisting of 10% CO_2_, 10% H_2_, and 80% N_2_. Following equilibration, *P. gingivalis* was inoculated in the medium and grown for 48 h at 37 °C under anaerobic conditions. *S. gordonii* DL-1 (1) was cultured aerobically without shaking in brain heart infusion (BHI) broth supplemented with 1 percent yeast extract for 16 hours at 37 °C. For some compounds that inhibited *P. gingivalis* adherence to *S. gordonii*, we determined their effect on planktonic growth of *P. gingivalis* and *S. gordonii*. The appropriate growth medium (10 ml) was supplemented with 10 μl of a 40 mM compound stock solution in DMSO. After inoculation, broth cultures were incubated as described above and compared to cultures grown in medium that was supplemented only with 0.1% DMSO. The cell density of each culture was determined by measuring the optical density at 600 nm (O.D._600nm_) and the percent growth inhibition/stimulation was calculated using the following equation: [(O.D._600nm_ of the control/O.D._600nm_ of the culture grown in the presence of compound) – 1] × 100.

#### 4.2.2. Biofilm model for *in vitro* analysis of *P. gingivalis* adherence to *S. gordonii*

Two species biofilms were formed essentially as previously described.^39^ To prepare bacterial cells for biofilm culture, 10 ml of an overnight *S. gordonii* culture was centrifuged at 5600 rpm for 5 min and the cell pellet was suspended in 1 mL sterile PBS. Subsequently, 20 μL of 5 mg ml^–1^ hexidium iodide (Molecular Probes) was added to the cell suspension and incubated for 15 min with gentle shaking at room temperature in the dark. The labeled cells were centrifuged as described above, washed with phosphate-buffered saline (10 mM Na_2_HPO_4_, 18 mM KH_2_PO_4_, 1.37 M NaCl, and 2.7 mM KCl, pH 7.2; [PBS]) and the cell pellet was suspended in PBS at a final O.D._600nm_ of 0.8. Similarly, 10 mL of a 48 h culture of *P. gingivalis* was centrifuged at 5600 rpm for 15 min, the cell pellet was suspended in 1 mL PBS and 20 μL of 5(6)-carboxyfluorescein *N*-hydroxysuccinimide ester (4 mg ml^–1^, Molecular Probes, Inc.) was added. After incubation for 30 min with gentle shaking at room temperature in the dark, the suspension was centrifuged and washed as described above and suspended in PBS at a final O.D._600nm_ of 0.4. For biofilm cultures, 1 mL of labeled *S. gordonii* cells was added to each well of a 12-well microtiter plate (Greiner Bio-one) containing a circular coverslip (Fisher brand) and incubated in an anaerobic chamber with rotary shaking for 24 h at 37 °C. Unbound cells were removed by aspiration and 1 ml of labeled *P. gingivalis* cells containing the desired concentration of test compound was added and incubated under anaerobic conditions for 22 h at 37 °C. Test compounds were dissolved in dimethyl sulfoxide (DMSO) to generate 1000× stock solutions and were routinely tested over a final concentration range of 0–60 μM. 1 μL of the appropriate stock solution was added to each 1 mL aliquot of labeled *P. gingivalis* cells and incubated for 30 min at room temperature prior to adding the suspension to the microtiter plate wells. For control biofilms, 1 μL of DMSO was added to 1 mL of labeled *P. gingivalis* and incubated as described above.

#### 4.2.3. Visualization of two species biofilms

To visualize *P. gingivalis*/*S. gordonii* biofilms, unbound *P. gingivalis* cells were removed by aspiration and coverslips were washed once with PBS. Biofilms were fixed by incubating the coverslips with 1 mL of 4% paraformaldehyde for 5 min followed by two washes with PBS. The coverslips were then removed, placed face down on a glass microscope slide containing a drop of antifade reagent (Life Technology) and sealed with nail polish. Visualization of biofilms was carried out by laser scanning confocal microscopy using a Leica SP8 confocal microscope (Leica Microsystems Inc., Buffalo Grove, IL) using a 488 nm laser to detect labeled *P. gingivalis* and a 552 nm laser to detect *S. gordonii*. *Z*-plane scans of 25 μm in depth were collected at three randomly chosen frames on each coverslip using a *z*-step thickness of 0.7 μm. Background noise was minimized using software provided with the Leica SP8 and three-dimensional reconstruction of the *Z*-plane scans and quantification of total green and red fluorescence was conducted using Volocity 6.3 image analysis software (Perkin Elmer, Akron, Ohio). Data were expressed as the ratio of total green (*P. gingivalis*) to red (*S. gordonii*) fluorescence and the IC_50_ for each compound was defined as the concentration that reduced the ratio of green to red fluorescence by 50%. Experiments were carried out in triplicate for each concentration of test compound and three independent experiments were conducted for each compound. GraphPad InStat3 software was used for data analysis and statistical significance was defined as *p* < 0.05.

#### 4.2.4. Cell culture

Human telomerase immortalized gingival keratinocytes (TIGKs) were provided by Dr. Richard Lamont (University of Louisville) and were authenticated by comparison to primary gingival epithelial cells for cell morphology, growth, cytokeratin expression and the expression of toll-like receptors. TIGKs were cultured at 37 °C in an atmosphere of 5% CO_2_ in Basal Medium Dermalife K complete kit with Supplements (LifeLine, Frederick, MD). Cultures were incubated for 5 days and attained >95% confluence.

#### 4.2.5. Measurement of lactate dehydrogenase (LDH) activity

Lactate dehydrogenase (LDH) activity was determined using the CytoTox 96 non-radioactive Cytotoxicity Assay (Promega). TIGK cells were inoculated in a 96-well microtiter plate at a density of 4000 cells per well and grown for 24 h. The medium was then removed and replaced with fresh medium containing the desired concentration of peptidomimetic compound. The cells were further cultured for 18 h in the presence of the peptidomimetic compounds, centrifuged for 4 min at 250*g* and 50 μl of supernatant was transferred to a fresh 96-well microtiter plate. Subsequently, 50 μl of LDH substrate was added per well and plates were incubated at room temperature for 30 min. Reactions were terminated by the addition of 50 μl of stop solution provided in the CytoTox 96 kit. LDH activity was determined by measuring the optical density at a wavelength of 490 nm. For positive control reactions, 15 μl of lysis buffer provided in the CytoTox 96 kit was added to the cells and incubated for 1 h. Negative control reactions comprised cells that were incubated with medium alone. All samples were assayed in triplicate.

#### 4.2.6. Measurement of cellular ATP levels

Cell metabolic activity was assessed by quantifying total ATP levels in cell culture samples using CellTiterGlo reagent (Promega). TIGK and J774A.1 cells were cultured and incubated with compounds as described above, washed three times with sterile PBS and incubated with 100 μl of CellTiterGlo substrate for 2 min with shaking and for an additional 10 min without shaking. Total light production was measured using a Victor 3 multi-label plate reader (PerkinElmer) in luminometer mode. All samples were assayed in triplicate.

## Conflicts of interest

There are no conflicts to declare.

## Supplementary Material

Supplementary information
