# Supplementary material for: ‘Second-generation’ 1,2,3-triazole-based inhibitors of Porphyromonas gingivalis adherence to oral streptococci and biofilm formation
Source: Medchemcomm. 2019 Jan 15;10(2):268–79. doi: 10.1039/c8md00405f (PMC6390472; doi:10.1039/c8md00405f)
Supplement: Supplementary file 1 [file MD-010-C8MD00405F-s001.pdf]

Electronic Supplementary Material (ESI) for MedChemComm.
This journal is © The Royal Society of Chemistry 2018

Supporting Information

'Second-Generation' 1,2,3-Triazole-Based Inhibitors of *Porphyromonas gingivalis* Adherence to Oral Streptococci and Biofilm Formation

Pravin C. Patil[a], Jinlian Tan[b], Donald R. Demuth[b] *, Frederick A. Luzzio[a],*

[a]*Department of Chemistry, University of Louisville, 2320 South Brook Street, Louisville, KY 40292, USA*

[b]*Department of Oral Immunology and Infectious Diseases, University of Louisville School of Dentistry, 501 S. Preston St. Louisville, KY 40292, USA*

Content	Page No.
Analytical data for compounds **2-15**	S2-S8
Analytical data for compounds **25-27**	S8-S9
Analytical data for compounds **28-45**	S9-S21
Analytical data for compounds **46-57**	S21-S28
Analytical data for compounds **58-87**	S28-S36
Analytical data for compounds **89a-89c**	S37-S38
Analytical data for compounds **90-124**	S38-S59
^{1}H and ^{13}C NMR copies for compounds **2-15**	S60-S87
^{1}H and ^{13}C NMR copies for compounds **25-27**	S88-S93
^{1}H and ^{13}C NMR copies for compounds **28-45**	S89-S129
^{1}H and ^{13}C NMR copies for compounds **46-57**	S130-S154
^{1}H and ^{13}C NMR copies for compounds **58-87**	S155-S181
^{1}H and ^{13}C NMR copies for compounds **89a-89c**	S182-S187
^{1}H and ^{13}C NMR copies for compounds **90-124**	S188-S257
References	S258

Compounds 1-3 were synthesized by following the procedure mentioned in Ref. 1.

Analytical data for compounds 1-3:

*2-(chloromethyl)-4,5-diphenyloxazole **(1)**:* See Ref. 1

*2-(chloromethyl)-4,5-bis(4-fluorophenyl)oxazole **(2)**:*

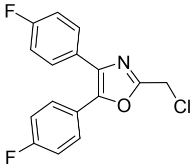

Off-white solid; yield 47%; mp = 78-81°C, R_f = 0.54 (hexane/ethyl acetate, 3:1); FT-IR: 3016, 1664, 1599, 1496, 1222, 835 cm^{-1}; 1H NMR (400 MHz, $CDCl_3$) δ 7.60-7.54 (m, 4H), 7.10-7.05 (m, 4H), 4.68 (s, 2H) ppm; ^{13}C NMR (100 MHz, $CDCl_3$) δ 163.0 (d, J = 248.8 Hz), 162.8 (d, J = 247.1 Hz), 157.7, 146.0, 134.9, 129.7 (d, J = 8.62 Hz), 128.8 (d, J = 8.5 Hz), 127.8, 124.5, 116 (d, J = 21 Hz), 115.7 (d, J = 21.8 Hz), 35.8 ppm; ; LRMS (+ESI) for $C_{10}H_{11}ClF_2NO$ was found 306 (M+1).

*2-(chloromethyl)-4,5-bis(4-chlorophenyl)oxazole **(3)**:*

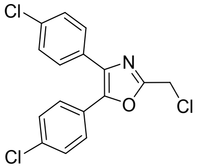

Pale yellow solid; yield 53%; mp = 62-64°C; R_f = 0.56 (hexane/ethyl acetate, 3:1); FT-IR: 3052, 1573, 1497, 1400, 1209, 824 cm^{-1}; ^{1}H NMR (400 MHz, $CDCl_3$) δ 7.56-7.51 (m, 4H), 7.38-7.35 (m, 4H), 4.68 (s, 2H) ppm; ^{13}C NMR (125 MHz, $CDCl_3$) δ 158.0, 146.2, 135.23, 135.18, 134.5, 130.0, 129.19, 129.16, 129.0, 128.0, 126.5, 35.8 ppm; LRMS (+ESI) for $C_{10}H_{11}Cl_3NO$ was found 338.

Analytical data for compounds 4-6:

*4-(((4,5-diphenyloxazol-2-yl)methyl)thio)aniline **(4)**:*

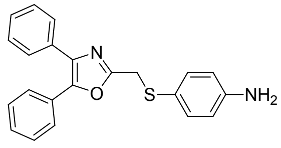

Light yellow solid; yield 55%; mp = 131-133°C; R_f = 0.17 (hexane/ethyl acetate, 7:3); FT-IR: 2125, 2090, 1591, 1487, 1291 cm^{-1}; ^{1}H NMR (500 MHz, $CDCl_3$): δ 7.61 (dd, *J* = 1.5 Hz, 3.5 Hz, 2H), 7.53 (dd, *J* = 2.0 Hz, 4.0 Hz, 2H), 7.37-7.29 (m, 8H), 6.60 (dd, *J* = 2.0 Hz, 6.5 Hz, 2H), 4.07 (s, 2H), 3.75 (s, 2H) ppm; ^{13}C NMR (125 MHz, $CDCl_3$): δ 160.2, 147.1, 145.8, 135.6 (overlap), 132.4, 128.5 (overlap), 128.1 (overlap), 128.0 (overlap), 126.5 (overlap), 121.3, 115.5 (overlap), 33.7 ppm; HRMS (+ESI) m/z calcd for [$C_{22}H_{18}N_2O_5$] 359.1218, found 359.1267 ($[M+H]^+$).

4-(((4,5-bis(4-fluorophenyl)oxazol-2-yl)methyl)thio)aniline ***(5)****:*

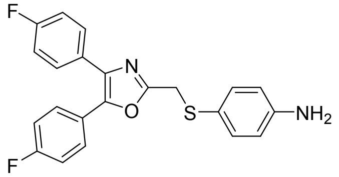

Light yellow soild; yield 85%, mp = 99-101°C; R_f = 0.35 (hexane/ethyl acetate, 1:1); FT-IR: 3460, 3353, 3238, 1648, 1596, 1496, 838, 756 cm^{-1}; ^{1}H NMR (400 MHz, $CDCl_3$): δ 7.54 (t, *J* = 6.8 Hz, 2H), 7.49 (t, *J* = 6.8 Hz, 2H), 7.28 (t, *J* = 8.0 Hz, 2H), 7.04 (t, *J* = 8.0 Hz, 4H), 6.57 (d, *J* = 8.0 Hz, 2H), 4.05 (s, 2H), 3.80 (s, 2H, NH_2) ppm; ^{13}C NMR (100 MHz, $CDCl_3$): δ 162.7 (s, *J* = 248.65 Hz), 162.6 (d, *J* = 246.8 Hz), 160.2, 147.2, 144.9, 135.6, 134.3, 129.7 (d, *J* = 8.4 Hz), 128.4 (d, *J* = 7.6 Hz), 128.2 (d, *J* = 3.1 Hz), 124.8 (d, *J* = 3.0 Hz), 121.0, 115.8 (d, *J* = 22.0 Hz), 115.6 (d, *J* = 22 Hz), 115.4 ppm; LRMS (+ESI) for $C_{22}H_{16}F_2N_2OS$ was found 394.

4-(((4,5-bis(4-chlorophenyl)oxazol-2-yl)methyl)thio)aniline ***(6)****:*

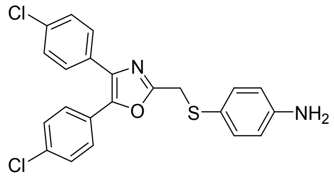

Light yellow soild; 79% yield; mp = 77-79°C; R_f = 0.26 (hexane/ethyl acetate, 3:1); FT-IR: 3334, 1619, 1596, 1495, 1091, 1055, 1012, 825 cm^{-1}; ^{1}H NMR (400 MHz, $CDCl_3$): δ 7.43 (d, J = 8.0 Hz, 2H), 7.34 (d, J = 8.0 Hz, 2H), 7.23-7.19 (m, 6H), 6.50 (d, J = 7.2 Hz, 2H), 3.97 (s, 2H), 3.70 (s, 2H) ppm; ^{13}C NMR (125 MHz, $CDCl_3$): δ 160.6, 147.1, 145.1, 135.6, 134.8, 134.6, 134.2, 130.6, 129.2, 129.0, 128.9, 127.7, 127.1, 121.1, 115.5, 33.6 ppm; LRMS (+ESI) for $C_{22}H_{16}Cl_2N_2OS$ was found 426.

Analytical data for compounds 7-9:

2-(((4-azidophenyl)thio)methyl)-4,5-diphenyloxazole ***(7)****:*

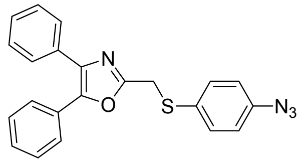

Light brown solid; yield 87%; mp = 64-65°C; R_f = 0.5 (hexane/ethyl acetate, 7.5:2.5); FT-IR: 2125, 2090, 1591, 1487, 1291 cm^{-1}; ^{1}H NMR (400 MHz, $CDCl_3$): δ 7.59 (d, J = 8.0 Hz, 2H), 7.53-7.50 (m, 3H), 7.48 (s, 1H), 7.38-7.34 (m, 6H), 6.98 (d, J = 8.4 Hz, 2H), 4.19 (s, 2H) ppm; ^{13}C NMR (125 MHz, $CDCl_3$): δ 159.3, 145.9, 139.6, 135.3, 133.3, 132.0, 130.4, 128.5, 128.4, 128.1, 127.8, 126.3, 119.5, 31.9 ppm; HRMS (+ESI) m/z calcd for $[C_{22}H_{16}N_4OS]^+$ 385.1123, found 385.1133 ($[M+H]^+$).

2-(((4-azidophenyl)thio)methyl)-4,5-bis(4-fluorophenyl)oxazole ***(8)****:*

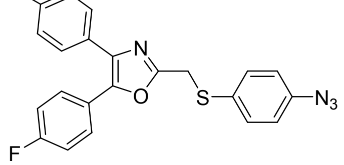

Light brown soild; yield 61%; mp = 108-109°C; R_f = 0.46 (hexane/ethyl acetate, 3:1); FT-IR: 2129, 2090, 1591, 1514, 1489, 1295, 1224, 835 cm^{-1}; ^1H NMR (400 MHz, $CDCl_3$): δ 7.53 (t, *J* = 6.8 Hz, 2H), 7.48-7.45 (m, 4H), 7.06 (t, *J* = 8.8 Hz, 4H), 6.97 (d, *J* = 7.6 Hz, 2H), 4.17 (s, 2H) ppm; ^{13}C NMR (100 MHz, $CDCl_3$): δ 162.8 (s, *J* = 249.1 Hz), 162.6 (d, *J* = 246.8 Hz), 159.8, 145.1, 139.8, 134.4, 133.5, 130.4, 129.6 (d, *J* = 8.4 Hz), 128.4 (d, *J* = 7.6 Hz), 128.0, 124.7, 119.7, 115.9 (d, *J* = 22.0 Hz), 115.7 (d, *J* = 22 Hz), 32.1 ppm; LRMS (+ ESI) for $C_{22}H_{14}F_2N_4OS$ was found 420.

2-(((4-azidophenyl)thio)methyl)-4,5-bis(4-chlorophenyl)oxazole ***(9)****:*

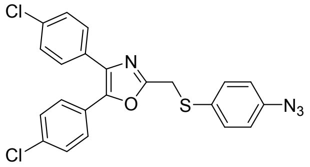

Light brown soild; yield 69%; mp = 104-106°C; R_f = 0.48 (hexane/ethyl acetate, 3/1); FT-IR: 2120, 2090, 1585, 1489, 1406, 1295, 1091, 1012, 821, 736 cm^{-1}; ^1H NMR (400 MHz, $CDCl_3$): δ 7.48 (m, 4H), 7.42 (d, *J* = 7.6 Hz, 2H), 7.35-7.33 (m, 4H), 6.97 (d, *J* = 7.6 Hz, 2H), 4.17 (s, 2H) ppm; ^{13}C NMR (125 MHz, $CDCl_3$): δ 159.9, 145.3, 139.9, 134.8, 134.3, 133.5, 130.3, 129.15, 129.09, 128.9, 127.7, 126.8, 119.7, 32.1 ppm; LRMS (+ESI) for $C_{22}H_{14}Cl_2N_4OS$ was found 452.

Analytical data for compounds 10-15:

2-(((4-azidophenyl)sulfinyl)methyl)-4,5-diphenyloxazole ***(10)****:*

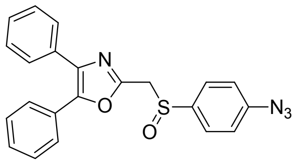

Colorless oil; yield 86%; R_f = 0.1 (ethyl acetate/hexane, 1:3); FT-IR: 3055, 2125, 2090, 1586, 1051 cm^{-1}; ^1H NMR (400 MHz, $CDCl_3$): δ 7.60 (d, *J* = 8.4 Hz, 2H), 7.57-7.55 (m, 2H), 7.47-7.45 (m, 2H), 7.39-7.34 (m, 6H), 7.14 (d, *J* = 8.4 Hz, 2H), 4.41 (d, *J* = 13.2 Hz, 1H), 4.21 (d, *J* = 13.2

Hz, 1H) ppm; ^{13}C NMR (175 MHz, $CDCl_3$): δ 153.1, 147.1, 144.0, 138.8, 136.1, 131.7, 129.0, 128.7, 128.6, 128.4, 126.1, 127.8, 126.5, 126.1, 119.8, 56.2 ppm; HRMS (+ESI) m/z calcd for $[C_{22}H_{16}N_4O_2S]^+$ 401.1072, found 401.1119 ($[M+H]^+$).

2-(((4-azidophenyl)sulfinyl)methyl)-4,5-bis(4-fluorophenyl)oxazole ***(11)***:

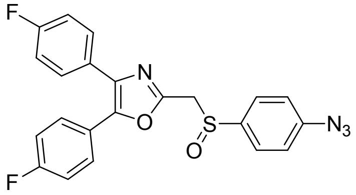

Brown oil; yield 95%; R_f = 0.34 (hexane/ethyl acetate, 1:3); FT-IR: 3058, 2126, 2092, 1586, 1489, 1223, 1051, 834 cm^{-1}; ^{1}H NMR (400 MHz, $CDCl_3$): δ 7.61 (d, J = 8.0 Hz, 2H), 7.53-7.49 (m, 2H), 7.44-7.41 (m, 2H), 7.15 (d, J = 8.0 Hz, 2H), 7.06 (d, J = 8.0 Hz, 4H), 4.35 (d, J = 13.2 Hz, 1H), 4.21 (d, J = 13.2 Hz, 1H) ppm; ^{13}C NMR (125 MHz, $CDCl_3$): δ 162.8 (d, J = 248.8 Hz), 162.7 (d, J = 247.0 Hz), 153.4, 146.0, 143.9, 138.8, 135.0, 129.6 (d, J = 7.6 Hz), 128.5 (d, J = 8.5 Hz), 127.6, 126.0, 124.2, 119.8, 115.9 (d, J = 22.0 Hz), 115.7 (d, J = 22.0 Hz), 55.9 ppm; LRMS for $C_{22}H_{14}F_2N_4O_2S$ (Pos Ion ES) was found 437 (M+H).

2-(((4-azidophenyl)sulfinyl)methyl)-4,5-bis(4-chlorophenyl)oxazole ***(12)***:

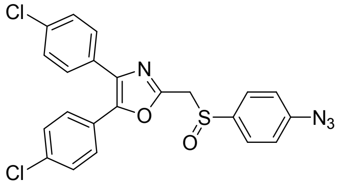

Light brown soild; yield 92%; mp = 99-101°C; R_f = 0.1 (hexane/ethyl acetate, 3:1); FT-IR: 2988, 2927, 2129, 2096, 1588, 1490, 1283, 1092, 1040, 816 cm^{-1}; ^{1}H NMR (400 MHz, $CDCl_3$): δ 7.61 (d, J = 7.2 Hz, 2H), 7.48 (d, J = 6.8 Hz, 2H), 7.40-7.35 (m, 6H), 7.15 (d, J = 7.6 Hz, 2H), 4.35 (d, J = 13.6 Hz, 1H), 4.21 (d, J = 13.6 Hz, 1H) ppm; ^{13}C NMR (125 MHz, $CDCl_3$): δ 153.7,

146.3, 144.1, 138.9, 135.5, 135.2, 134.6, 129.9, 129.1, 127.8, 126.4, 126.0, 119.9, 56.1 ppm; LRMS for $C_{22}H_{14}Cl_2N_4O_2S$ was found 468; Pos Ion ES: 469 (M+1), 491 (M+Na).

*2-(((4-azidophenyl)sulfonyl)methyl)-4,5-diphenyloxazole **(13)**:*

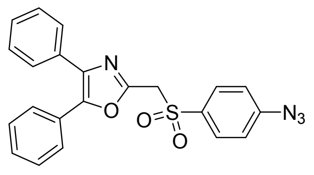

Light yellow solid; yield 93%; mp = 118-119°C; R_f = 0.29 (ethyl acetate/hexane, 1:3); FT-IR: 3054, 2934, 2125, 2097, 1586, 1316, 1290, 963 cm^{-1}; ^{1}H NMR (400 MHz, $CDCl_3$): δ 7.84 (d, J = 8.8 Hz, 2H), 7.54-7.50 (m, 4H), 7.37-7.35 (m, 6H), 7.14 (d, J = 8.4 Hz, 2H), 4.64 (s, 2H) ppm; ^{13}C NMR (175 MHz, $CDCl_3$): δ 151.4, 147.6, 146.6, 136.2, 134.0, 131.5, 130.7, 129.1, 128.7, 128.6, 128.5, 127.9, 127.8, 126.6, 119.5, 56.0 ppm; HRMS (+ESI) m/z calcd for $[C_{22}H_{16}N_4O_3S]^+$ 417.1021, found m/z 417.1000 ($[M+H]^+$).

2-(((4-azidophenyl)sulfonyl)methyl)-4,5-bis(4-fluorophenyl)oxazole ***(14)***:

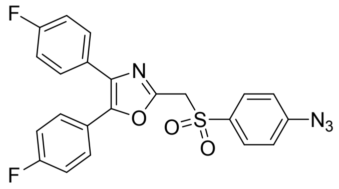

Light yellow soild; yield 93%; mp = 142-144°C; R_f = 0.32 (hexane/ethyl acetate, 3:1); FT-IR: 2953, 2124, 2100, 1587, 1498, 1312, 1291, 1237, 1155, 834 cm^{-1}; ^{1}H NMR (400 MHz, $CDCl_3$): δ 7.83 (d, J = 8.0 Hz, 2H), 7.47 (dd, J = 5.2 Hz, 12.8 Hz, 4H), 7.14 (d, J = 8.4 Hz, 2H), 7.05 (dd, J = 7.2 Hz, 15.2 Hz, 4H), 4.63 (s, 2H) ppm; ^{13}C NMR (175 MHz, $CDCl_3$): δ 163.0 (d, J = 249.1 Hz), 162.7 (d, J = 247.6 Hz), 151.5, 146.6, 135.1, 134.1, 130.7, 129.6 (d, J = 8.3 Hz), 128.6 (d, J = 8.4 Hz), 127.4, 124.0, 119.5, 116.0 (d, J = 22.0 Hz), 115.8 (d, J = 21.3 Hz), 55.9 ppm; LRMS (+ESI) for $C_{22}H_{14}F_2N_4O_3S$ was found 452.

2-*(((4-azidophenyl)sulfonyl)methyl)-4,5-bis(4-chlorophenyl)oxazole* ***(15)***:

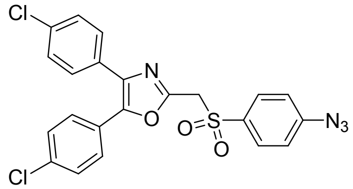

Light brown soild; yield 86%; mp = 143-144°C; R_f = 0.4 (hexane/ethyl acetate, 3:1); FT-IR: 2988, 2893, 21119, 2097, 1586, 1484, 1312, 1288, 1153, 1095, 827 cm^{-1}; ^{1}H NMR (500 MHz, $CDCl_3$): δ 8.16 (d, *J* = 2.12 Hz, 2H), 7.78-7.76 (m, 4H), 7.68-7.65 (m, 4H), 7.47 (d, *J* = 2.2 Hz, 2H), 4.91 (s, 2H) ppm; ^{13}C NMR (125 MHz, $CDCl_3$): δ 151.9, 146.7, 135.4, 134.7, 134.1, 130.7, 129.7, 129.2, 129.0, 127.9, 126.2, 119.5, 55.9 ppm; LRMS for $C_{22}H_{14}Cl_2N_4O_3S$ was found 484.

Analytical data for compounds 25-27:

N2,N2,N4,N4-tetraethyl-6-ethynyl-1,3,5-triazine-2,4-diamine ***(25)*****:**

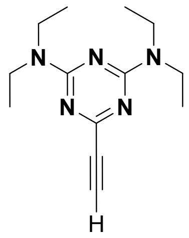

Off-white solid; yield 59%; mp = 47-49°C; R_f = 0.33 (hexane/ethyl acetate, 9:1; FT-IR: 3228, 2977, 2933, 2112, 1539, 1492, 1355, 1084 cm^{-1}; ^{1}H NMR (400 MHz, $CDCl_3$): δ 3.59 (d, *J* = 6.0 Hz, 4H), 3.53 (d, *J* = 6.0 Hz, 4H), 2.87 (s, 1H), 1.15 (t, *J* = 7.2 Hz, 12H) ppm; ^{13}C NMR (125 MHz, $CDCl_3$): δ 163.6, 157.9, 82.7, 72.9, 41.1, 13.5, 12.8 ppm; LRMS (+ESI) for [$C_{13}H_{21}N_5$] found 247.

6-ethynyl-N2,N4-bis(2-fluorophenyl)-1,3,5-triazine-2,4-diamine ***(26)*****:**

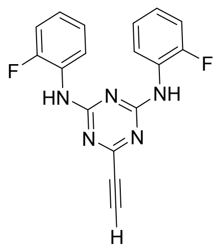

Off-white solid; yield 69%; mp = 208-210°C; R_f = 0.31 (hexane/ethyl acetate, 7.5:2.5); FT-IR: 3289, 3216, 3060, 2126, 1608, 1532, 1495, 1455, 1409, 1257, 814 cm^{-1}; ^{1}H NMR (400 MHz, $CDCl_3$): δ 8.21 (s, 2H), 7.41 (s, 2H), 7.15-7.06 (m, 6H), 3.09 (s, 1H) ppm; ^{13}C NMR (125 MHz, $CDCl_3$): δ 164.1, 159.2, 153.5 (d, J = 242.1 Hz), 126.0 (d, J = 10.5 Hz), 124.6, 124.1, 123.1, 115.1 (d, J = 20.0 Hz), 109.9, 80.8 ppm; LRMS (+ESI) for [$C_{17}H_{11}F_2N_5$] found 323.

*6-ethynyl-N_2,N_4-bis(4-fluorophenyl)-1,3,5-triazine-2,4-diamine **(27)***:

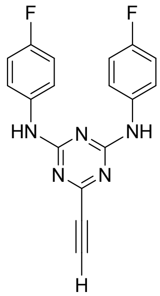

Off-white solid; yield 88 %; mp = 205-207°C; R_f = 0.27 (hexane/ethyl acetate, 3:1); FT-IR: 3416, 3389, 3301, 2126, 1621, 1591, 1566, 1496, 1403, 1206 cm^{-1}; ^{1}H NMR (400 MHz, $CDCl_3$): δ 7.46 (s, 4H), 7.04 (s, 2H), 7.02 (t, J = 7.6 Hz, 4H), 3.05 (s, 1H) ppm; ^{13}C NMR (125 MHz, $CDCl_3$): δ 164.1, 160.6, 159.1, 158.7, 113.5, 123.1, 115.6, 115.5 (d, J = 21.8 Hz), 115.2, 110.0, 76.2 ppm; LRMS (+ESI) for [$C_{17}H_{11}F_2N_5O$] found 323.

Analytical data for compounds 28-57:

*4,5-diphenyl-2-(((4-(4-(pyridin-3-yl)-1H-1,2,3-triazol-1-yl)phenyl)sulfinyl) methyl)oxazole **(28):***

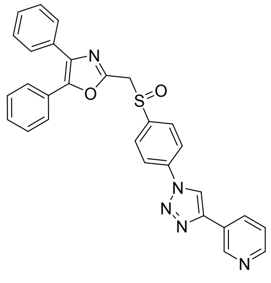

Light yellow solid; yield 48%; mp = 193-195°C; R_f = 0.26 (methanol/chloroform, 1:9); FT-IR (neat): 3085, 3038, 2986, 2929, 1593, 1507, 1404, 1238, 1049, 687 cm^{-1}; ^{1}H NMR (400 MHz, $CDCl_3$): δ 9.07 (s, 1H), 8.65 (s, 1H), 8.29 (d, *J* = 8.4 Hz, 1H), 8.19 (s, 1H), 7.96 (d, *J* = 8.4 Hz, 2H), 7.82 (d, *J* = 8.0 Hz, 2H), 7.57-7.55 (m, 1H), 7.47-7.42 (m, 3H), 7.37-7.29 (m, 6H), 4.40 (dd, *J* = 14.0 Hz, 68.4 Hz, 2H) ppm; ^{13}C NMR (175 MHz, $CDCl_3$): δ 152.7, 149.8, 147.2, 147.1, 145.8, 143.7, 139.2, 136.2, 133.2, 131.6, 129.0, 128.7, 128.6, 128.5, 128.0, 127.8, 126.5, 126.1, 126.0, 123.9, 121.1, 117.7, 56.1 ppm; HRMS (+ESI) m/z calcd for $[C_{29}H_{21}N_5O_2S]^+$ 504.1494, found 504.1516 ($[M+H]^+$).

2-(((4-(4-(4-fluorophenyl)-1H-1,2,3-triazol-1-yl)phenyl)sulfinyl)methyl)-4,5-diphenyloxazole ***(29):***

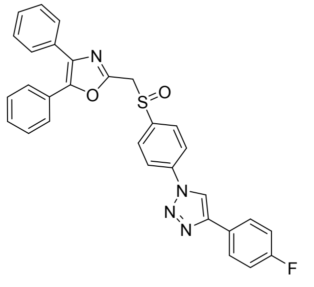

Light yellow solid; yield 89%; mp = 193-195°C; R_f = 0.20 (hexane/ethyl acetate, 3:7); FT-IR (neat): 3133, 1592, 1493, 1233, 1213, 1054, 827 cm^{-1}; ^{1}H NMR (400 MHz, $CDCl_3$): δ 8.09 (s, 1H), 7.95 (d, *J* = 8.4 Hz, 2H), 7.88 (dd, *J* = 5.2 Hz, 8.8 Hz, 2H), 7.80 (d, *J* = 8.4 Hz, 2H), 7.57-7.55 (m, 2H), 7.46-7.44 (m, 2H), 7.35-7.29 (m, 6H), 7.17 (t, *J* = 8.8 Hz, 2H), 4.47 (d, *J* = 6.1 Hz, 1H), 4.31(d, *J* = 5.8 Hz, 1H) ppm; ^{13}C NMR (175 MHz, $CDCl_3$): δ 163.0 (d, *J* = 246.6 Hz),

152.8, 147.9, 147.2, 143.4, 139.4, 136.2, 131.6, 129.0, 128.7, 128.6, 128.5, 128.0, 127.8, 127.6 (d, J = 8.05 Hz), 126.5, 126.1, 126.0 (d, J = 3.5 Hz), 120.9, 117.1, 116.0 (d, J = 21.7 Hz), 56.1 ppm; HRMS (+ESI) m/z calcd for $[C_{30}H_{21}FN_4O_2S]^+$ 521.1447, found 521.1407.

2-(((4-(4-(3-fluorophenyl)-1H-1,2,3-triazol-1-yl)phenyl)sulfinyl)methyl)-4,5-diphenyloxazole ***(30):***

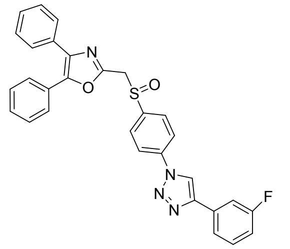

White solid; yield 99%; mp = 177-179°C; R_f = 0.20 (hexane/ethyl acetate, 3:7); FT-IR (neat): 3070, 3009, 1588, 1488, 1229, 1053, 987, 761 cm^{-1}; ^1H NMR (400 MHz, $CDCl_3$): δ 8.12 (s, 1H), 7.95 (d, J = 8.4 Hz, 2H), 7.81 (d, J = 8.4 Hz, 2H), 7.68-7.62 (m,2H), 7.59-7.55 (m, 2H), 7.45-7.42 (m, 3H), 7.35-7.29 (m, 6H), 7.11-7.07 (m, 1H), 4.47 (d, J = 5.8 Hz, 1H), 4.31(d, J = 5.8 Hz, 1H) ppm; ^{13}C NMR (175 MHz, $CDCl_3$): δ 163.2 (d, J = 245.2 Hz), 152.7, 147.7 (d, J = 2.62 Hz), 147.2, 143.5, 139.3, 136.2, 131.9 (d, J = 8.05 Hz), 131.6, 130.6 (d, J = 8.05 Hz), 129.0, 128.7, 128.6, 128.5, 128.0, 127.8, 126.5, 126.1, 121.47, 121.46, 121.0, 119.8, 117.7, 115.5 (d, J = 21.0 Hz), 112.9 (d, J = 22.9 Hz), 56.1 ppm; HRMS (+ESI) m/z calcd for $[C_{30}H_{21}FN_4O_2S]^+$ 521.1447, found m/z 521.1528($[M+H]^+$).

2-(((4-(4-(2-methoxyphenyl)-1H-1,2,3-triazol-1-yl)phenyl)sulfinyl)methyl)-4,5-diphenyloxazole ***(31):***

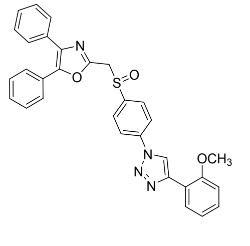

Light yellow solid; yield 98%; mp = 131-133°C; R_f = 0.26 (hexane/ethyl acetate, 3:7); FT-IR (neat): 3145, 2995, 2928, 1712, 1489, 1251, 1029, 742 cm^{-1}; ^1H NMR (400 MHz, $CDCl_3$): δ 8.47 (s, 1H), 8.43 (dd, *J* = 1.6 Hz, 7.6 Hz, 1H), 8.00 (d, *J* = 9.2 Hz, 2H), 7.80 (d, *J* = 8.4 Hz, 2H), 7.57-7.55 (m, 2H), 7.46-7.43 (m, 2H), 7.40-7.34 (m, 5H), 7.31-7.29 (m, 2H), 7.14 (t, *J* = 7.2 Hz, 1H), 7.03 (t, *J* = 8.0 Hz, 1H), 4.47 (d, *J* = 5.9 Hz, 1H), 4.32 (d, *J* = 5.9 Hz, 1H), 3.99 (s, 3H) ppm; ^{13}C NMR (175 MHz, $CDCl_3$): δ 155.8, 152.9, 147.2, 144.2, 142.9, 139.7, 136.2, 131.6, 129.5, 129.0, 128.7, 128.6, 128.4, 128.0, 127.91, 127.90, 127.8, 126.5, 125.9, 121.2, 120.9, 120.54, 120.53, 118.6, 110.8, 56.1, 55.5 ppm; HRMS (+ESI) m/z calcd for $[C_{31}H_{24}N_4O_3S]^+$ 533.1647, found 533.1632($[M+H]^+$).

4,5-diphenyl-2-(((4-(4-(2-(trifluoromethyl)phenyl)-1H-1,2,3-triazol-1-yl)phenyl)sulfinyl)methyl)oxazole ***(32):***

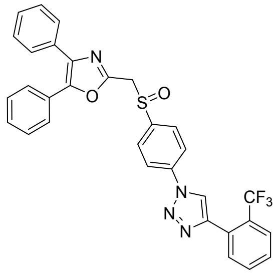

White solid; yield 61%; mp = 134-136°C; R_f = 0.29 (hexane/ethyl acetate, 3:7); FT-IR (neat): 2986, 2925, 1583, 1503, 1399, 1318, 1172, 1039, 757 cm^{-1}; ^1H NMR (400 MHz, $CDCl_3$): δ 8.18 (s, 1H), 8.04 (d, *J* = 7.6 Hz, 1H), 7.98 (d, *J* = 8.4 Hz, 2H), 7.81 (d, *J* = 8.4 Hz, 2H), 7.69 (t, *J* = 7.2 Hz, 1H), 7.57-7.53 (m, 3H), 7.46-7.44 (m, 2H), 7.37-7.29 (m, 6H), 4.47 (d, *J* = 5.9 Hz, 1H), 4.32 (d, *J* = 5.8 Hz, 1H) ppm; ^{13}C NMR (175 MHz, $CDCl_3$): δ 152.8, 147.2, 145.3, 143.5, 139.2, 136.2, 132.2, 131.7, 131.6, 129.0, 128.8, 128.69, 128.67, 128.64, 128.5, 128.0, 127.8, 127.4 (q, *J* = 21.0 Hz), 126.5, 126.3 (q, *J* = 5.4 Hz), 126.1, 124.0 (q, *J* = 271.6 Hz), 121.1, 120.6 (q, *J* = 3.5

Hz), 56.1 ppm; HRMS (+ESI) m/z calcd for $[C_{31}H_{21}F_3N_4O_2S]^+$ 571.1416, found m/z 571.1493 ($[M+H]^+$).

2-(((4-(4-(6-methoxynaphthalen-2-yl)-1H-1,2,3-triazol-1-yl)phenyl)sulfinyl)methyl)-4,5-diphenyloxazole ***(33):***

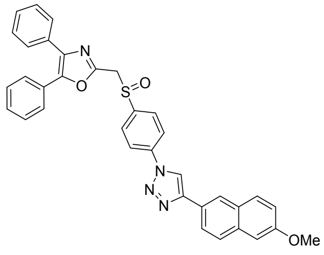

White solid; yield 59%; mp = 197-200°C; R_f = 0.17 (hexane/ethyl acetate, 3:7); FT-IR (neat): 3123, 2989, 2925, 1611, 1503, 1399, 1271, 1039, 742 cm^{-1}; ^{1}H NMR (400 MHz, $CDCl_3$): δ 8.35 (s, 1H), 8.20 (s, 1H), 8.00-7.94 (m, 3H), 7.85-7.81 (m, 4H), 7.58-7.56 (m, 2H), 7.47-7.45 (m, 2H), 7.36-7.30 (m, 6H), 7.22-7.18 (m, 2H), 4.49 (d, *J* = 5.8 Hz, 1H), 4.43 (d, *J* = 5.9 Hz, 1H), 3.96 (s, 3H) ppm; ^{13}C NMR (175 MHz, $CDCl_3$): δ 158.2, 152.8, 149.0, 147.2, 143.2, 139.5, 136.2, 134.6, 131.6, 129.8, 129.0, 128.9, 128.7, 128.6, 128.5, 128.0, 127.8, 127.6, 126.5, 126.1, 124.9, 124.73, 124.72, 124.2, 120.9, 119.5, 117.1, 117.0, 105.8, 56.1, 55.3 ppm; HRMS (+ESI) m/z calcd for $[C_{35}H_{26}N_4O_3S]^+$ 583.1804, found m/z 583.1885 ($[M+H]^+$).

2-(((4-(4-(4-pentylphenyl)-1H-1,2,3-triazol-1-yl)phenyl)sulfinyl)methyl)-4,5-diphenyloxazole ***(34):***

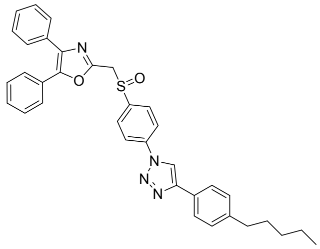

White solid; yield 40%; mp = 182-184°C; R_f = 0.29 (methanol/chloroform, 1:9); FT-IR (neat): 3053, 2929, 2859, 1592, 1490, 1224, 1039, 761 cm^{-1}; ^{1}H NMR (400 MHz, $CDCl_3$): δ 8.09 (d, *J* =

1.2 Hz, 1H), 7.96 (d, *J* = 7.6 Hz, 2H), 7.82-7.79 (m, 4H), 7.57-7.55 (m, 2H), 7.46-7.44 (m, 2H), 7.37-7.34 (m, 3H), 7.31-7.29 (m, 5H), 4.48 (d, *J* = 5.9 Hz, 1H), 4.31(d, *J* = 5.9 Hz, 1H), 2.66 (t, *J* = 7.6 Hz, 2H), 1.68-1.65 (m, 2H), 1.37-1.35 (m, 4H), 0.91 (t, *J* = 6.0 Hz, 3H) ppm; ^{13}C NMR (175 MHz, $CDCl_3$): δ 152.8, 148.9, 147.2, 143.8, 143.2, 139.5, 136.2, 131.6, 129.0, 128.7, 128.6, 128.0, 127.8, 127.1, 126.5, 126.3, 126.0, 125.8, 120.9, 116.8, 56.1, 35.7, 31.5, 31.0, 22.5, 14.0 ppm; HRMS (+ESI) m/z calcd for $[C_{35}H_{32}N_4O_2S]^+$ 573.2319m found m/z 573.2309 ($[M+H]^+$).

2-(((4-(4-(4-phenoxyphenyl)-1H-1,2,3-triazol-1-yl)phenyl)sulfinyl)methyl)-4,5-diphenyloxazole ***(35):***

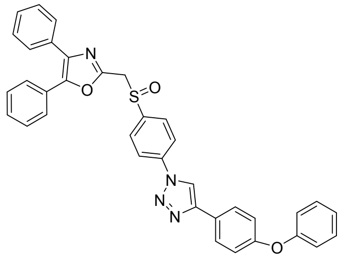

Light yellow solid; yield 81%; mp = 197-199°C; R_f = 0.24 (methanol/chloroform, 1:9); FT-IR (neat): 3064, 1590, 1559, 1485, 1404, 1228, 1054, 758 cm^{-1}; ^1H NMR (400 MHz, $CDCl_3$): δ 8.08 (s, 1H), 7.96 (d, *J* = 8.4 Hz, 2H), 7.86 (d, *J* = 8.4 Hz, 2H), 7.81 (d, *J* = 8.4 Hz, 2H), 7.56 (d, *J* = 7.6 H, 2H), 7.46-7.44 (m, 2H), 7.40-7.29 (m, 8H), 7.17-7.07 (m, 5H), 4.48 (d, *J* = 5.9 Hz, 1H), 4.31(d, *J* = 5.8 Hz, 1H) ppm; ^{13}C NMR (175 MHz, $CDCl_3$): δ 157.9, 156.7, 152.8, 148.4, 147.2, 143.3, 139.5, 136.2, 131.6, 129.9, 129.1, 128.74, 128.67, 128.5, 128.1, 127.8, 127.4, 126.5, 126.1, 124.8, 123.7, 120.9, 119.3, 119.0, 116.8, 56.2 ppm; HRMS (+ESI) m/z calcd for $[C_{36}H_{26}N_4O_3S]^+$ 595.1798, found 595.1796 ($[M+H]^+$).

2-(((4-(4-(3,5-bis(trifluoromethyl)phenyl)-1H-1,2,3-triazol-1-yl)phenyl)sulfinyl)methyl)-4,5-diphenyloxazole ***(36):***

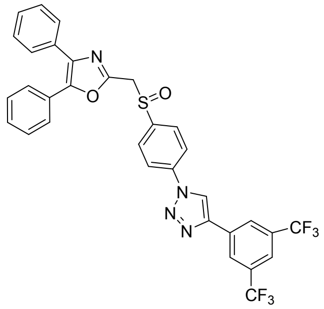

White solid; yield 91%; mp = 199-201°C; R_f = 0.33 (hexane/ethyl acetate, 3:7); FT-IR (neat): 3124, 3099, 1593, 1502, 1375, 1276, 1172, 1129, 1054, 680 cm^{-1}; ^{1}H NMR (400 MHz, $CDCl_3$): δ 8.36 (s, 1H), 8.22 (s, 1H), 7.95 (d, *J* = 8.0 Hz, 2H), 7.90 (s, 1H), 7.82 (d, *J* = 8.0 H, 2H), 7.57-7.55 (m, 2H), 7.47-7.45 (m, 2H), 7.37-7.30 (m, 6H), 4.50 (d, *J* = 5.8 Hz, 1H), 4.32 (d, *J* = 5.8 Hz, 1H) ppm; ^{13}C NMR (175 MHz, $CDCl_3$): δ 152.7, 147.2, 146.0, 144.0, 139.0, 136.2, 132.5 (q, *J* = 33.2 Hz), 131.9, 131.6, 129.0, 128.73, 128.67, 128.5, 128.0, 127.8, 126.5, 126.3, 126.2, 125.7 (q, *J* = 2.8 Hz), 123.1 (q, *J* = 271.7 Hz), 122.1 (q, *J* = 4.02 Hz), 121.2, 118.6, 56.1 ppm; HRMS (+ESI) m/z calcd for $[C_{32}H_{20}F_6N_4O_2S]^+$ 639.1284, found m/z 639.1275 ($[M+H]^+$).

4,5-diphenyl-2-(((4-(4-(pyridin-3-yl)-1H-1,2,3-triazol-1-yl)phenyl)sulfonyl)methyl)oxazole ***(37):***

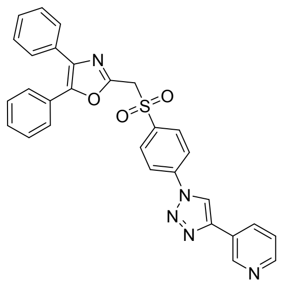

White solid; yield 73%; mp = 127-128°C; R_f = 0.37 (methanol/chloroform, 1:9); FT-IR (neat): 3121, 3078, 2987, 1597, 1328, 1233, 1158, 986 cm^{-1}; ^{1}H NMR (400 MHz, $CDCl_3$): δ 9.09 (s, 1H), 8.66 (s, 1H), 8.31-8.28 (m, 2H), 8.05 (dd, *J* = 8.4 Hz, 24.0 Hz, 4H), 7.52-7.50 (m, 4H), 7.45 (dd, *J* = 4.8 Hz, 7.6 Hz, 1H), 7.34-7.33 (m, 6H), 4.73 (s, 2H) ppm; ^{13}C NMR (175 MHz, $CDCl_3$): δ 151.1, 149.9, 147.8, 147.1, 146.1, 140.8, 138.2, 136.3, 133.3, 131.4, 130.9, 129.2, 128.8, 128.7,

128.6, 127.9, 127.8, 126.6, 126.2, 123.9, 120.5, 117.6, 56.0 ppm; HRMS (+ESI) m/z calcd for $[C_{29}H_{21}N_5O_3S]^+$ 520.1443, found m/z 520.1428 ($[M+H]^+$).

2-(((4-(4-(4-fluorophenyl)-1H-1,2,3-triazol-1-yl)phenyl)sulfonyl)methyl)-4,5-diphenyloxazole ***(38):***

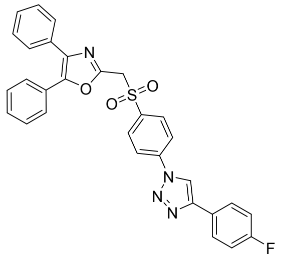

Light yellow solid; yield 63%; mp = 203-205°C; R_f = 0.48 (hexane/ethyl acetate, 1:1); FT-IR (neat): 3145, 3050, 1597, 1494, 1323, 1228, 1150, 1039, 993, 756 cm^{-1}; ^1H NMR (400 MHz, $CDCl_3$): δ 8.17 (s, 1H), 8.03 (d, J = 3.8 Hz, 2H), 8.00 (d, J = 3.8 Hz, 2H), 7.88 (dd, J = 5.6 Hz, 9.2 Hz, 2H), 7.53-7.49 (m, 4H), 7.34-7.33 (m, 6H), 7.18 (t, J = 8.8 Hz, 2H), 4.72 (s, 2H) ppm; ^{13}C NMR (175 MHz, $CDCl_3$): δ 163.06 (d, J = 247.2 Hz), 151.1, 148.2, 147.8, 141.1, 137.9, 136.3, 131.4, 130.8, 129.2, 128.8, 128.6, 128.5, 127.9, 127.77, 127.74 (d, J = 8.9 Hz), 126.6, 125.76 (d, J = 3.5 Hz), 120.4, 116.9, 116.0 (d, J = 21.52 Hz), 56.0 ppm; HRMS (+ESI) m/z calcd for $[C_{30}H_{21}FN_4O_3S]^+$ 537.1397, found m/z 537.1417 ($[M+H]^+$).

2-(((4-(4-(3-fluorophenyl)-1H-1,2,3-triazol-1-yl)phenyl)sulfonyl)methyl)-4,5-diphenyloxazole ***(39):***

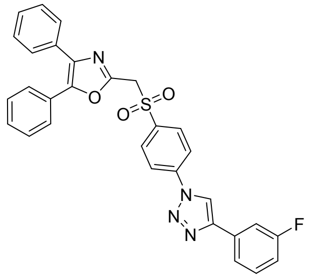

White solid; yield 69%; mp = 187-189°C; R_f = 0.58 (hexane/ethyl acetate, 1:1); FT-IR (neat): 3155, 3059, 2925, 1594, 1403, 1318, 1299, 1153, 860 cm^{-1}; ^1H NMR (400 MHz, $CDCl_3$): δ 8.21 (s, 1H), 8.03 (dd, J = 8.4 Hz, 24.8 Hz, 4H), 7.68-7.62 (m, 2H), 7.51-7.42 (m, 5H), 7.33-7.32 (m, 6H), 7.10 (t, J = 6.8 Hz, 1H), 4.72 (s, 2H) ppm; ^{13}C NMR (175 MHz, $CDCl_3$): δ 163.2 (d, J = 245.7 Hz), 151.1, 148.0, 147.8, 140.9, 138.0, 136.3, 131.6 (d, J = 8.7 Hz), 131.4, 130.9, 130.7 (d, J = 8.2 Hz), 129.2, 128.8, 128.7, 128.6, 127.9, 127.8, 126.6, 121.5 (d, J = 2.8 Hz), 120.4, 117.6, 115.7 (d, J = 21.0 Hz), 112.9 (d, J = 23.1 Hz), 56.0 ppm; HRMS (+ESI) m/z calcd for $[C_{30}H_{21}FN_4O_3S]^+$ 537.1397, found m/z 537.1399 ($[M+H]^+$).

2-(((4-(4-(2-methoxyphenyl)-1H-1,2,3-triazol-1-yl)phenyl)sulfonyl)methyl)-4,5-diphenyloxazole ***(40):***

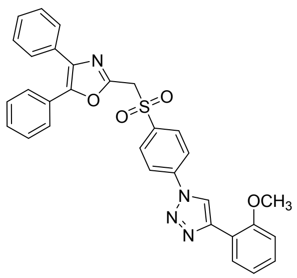

Pale yellow soild; yield 98%; mp = 188-190°C; R_f = 0.33 (hexane/ethyl acetate, 1:1); FT-IR (neat): 3184, 3052, 3024, 1596, 1493, 1323, 1253, 1148, 1032, 751 cm^{-1}; ^1H NMR (400 MHz, $CDCl_3$): δ 8.51 (s, 1H), 8.43 (dd, J = 1.6 Hz, 7.2 Hz, 1H), 8.05 (s, 4H), 7.53-4.89 (m, 4H), 7.41-7.37 (m, 1H), 7.36-7.31 (m, 6H), 7.14 (ddd, J = 0.8 Hz, 7.2 Hz, 14.8 Hz, 1H), 7.04 (d, J = 8.0 Hz, 1H), 4.72 (s, 2H), 4.00 (s, 3H) ppm; ^{13}C NMR (175 MHz, $CDCl_3$): δ 155.9, 151.2, 147.7, 144.5, 141.4, 137.5, 136.3, 131.4, 130.7, 129.6, 129.2, 128.7, 128.6, 128.5, 127.94, 127.89, 127.79, 126.6, 121.2, 120.39, 120.37, 118.4, 110.9, 56.0, 55.5 ppm; HRMS (+ESI) m/z calcd for $[C_{31}H_{24}N_4O_4S]^+$ 549.1591, found m/z 549.1600 ($[M+H]^+$).

4,5-diphenyl-2-(((4-(4-(2-(trifluoromethyl)phenyl)-1H-1,2,3-triazol-1-yl)phenyl)sulfonyl)methyl)oxazole ***(41):***

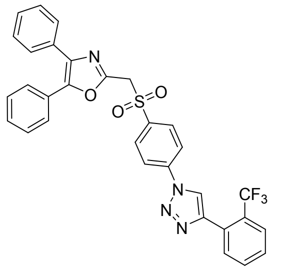

White solid; yield 54%; mp = 124-126°C; R_f = 0.45 (hexane/ethyl acetate, 1:1); FT-IR (neat): 3054, 3007, 2939, 1710, 1592, 1502, 1404, 1313, 1228, 1148, 1025 cm^{-1}; ^{1}H NMR (400 MHz, $CDCl_3$): δ 8.23 (s, 1H), 8.08-8.00 (m, 5H), 7.81 (d, *J* = 7.6 Hz, 1H), 7.69 (t, *J* = 7.6 Hz, 1H), 7.58-7.48 (m, 5H), 7.36-7.32 (m, 6H), 4.73 (s, 2H) ppm; ^{13}C NMR (175 MHz, $CDCl_3$): δ 151.1, 147.7, 145.6, 140.9, 138.1, 136.3, 132.2, 131.7, 130.9, 129.2, 128.9, 128.7, 128.6, 128.56, 128.4, 127.9, 127.8, 127.4 (q, *J* = 30.62 Hz), 126.6, 126.3 (q, *J* = 5.42 Hz), 124.1 (q, *J* = 271.6 Hz), 120.6, 120.5 (q, *J* = 5.42 Hz), 56.0 ppm; HRMS (+ESI) m/z calcd for $[C_{31}H_{21}F_3N_4O_3S]^+$ 587.1365, found m/z 587.1416 ($[M+H]^+$).

2-(((4-(4-(6-methoxynaphthalen-2-yl)-1H-1,2,3-triazol-1-yl)phenyl)sulfonyl)methyl)-4,5-diphenyloxazole ***(42):***

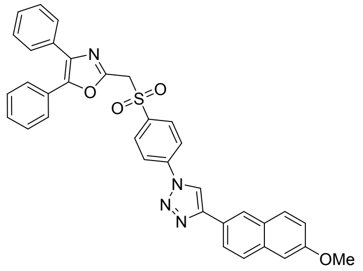

Pale yellow solid; yield 97%; mp = 246-247°C; R_f = 0.46 (ethyl acetate/hexane, 7:3); FT-IR (neat): 3126, 3054, 3000, 1592, 1502, 1394, 1323, 1149, 1033, 756 cm^{-1}; ^{1}H NMR (400 MHz, $CDCl_3$): δ 8.36 (s, 1H), 8.28 (s, 1H), 8.06 (dd, *J* = 9.6 Hz, 15.2 Hz, 4H), 7.95 (dd, *J* = 1.6 Hz, 8.4

Hz, 1H), 7.84 (t, *J* = 8.0 Hz, 2H), 7.54-7.49 (m, 4H), 7.37-7.33 (m, 6H), 7.22-7.18 (m, 2H), 4.73 (s, 2H), 3.96 (s, 3H) ppm; ^{13}C NMR (175 MHz, $CDCl_3$): δ 158.2, 152.7, 148.5, 147.0, 141.0, 138.3, 135.8, 134.7, 133.7, 131.7, 131.0, 130.1, 129.8, 129.4, 129.2, 128.97, 128.93, 128.1, 128.0, 127.8, 126.9, 125.5, 124.5, 124.4, 120.6, 120.2, 119.8, 106.6, 55.7, 54.9 ppm; HRMS (+ESI) m/z calcd for $C_{35}H_{26}N_4O_4S]^+$ 599.1753, found m/z 599.1759 ($[M+H]^+$).

[*2-(((4-(4-(4-pentylphenyl)-1H-1,2,3-triazol-1-yl)phenyl)sulfonyl)methyl)-4,5-diphenyloxazole* ***(43):***

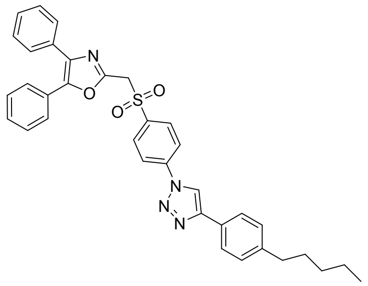

Pale yellow solid; yield 77%; mp = 179-181°C; R_f = 0.57 (hexane/ethyl acetate, 1:1); FT-IR (neat): 3131, 3054, 3007, 2925, 1592, 1328, 1304, 1162, 1036, 992 cm^{-1}; ^{1}H NMR (400 MHz, $CDCl_3$): δ 8.17 (s, 1H), 8.01 (dd, *J* = 8.8 Hz, 20.4 Hz, 4H), 7.81 (d, *J* = 8.0 Hz, 2H), 7.53-7.48 (m, 4H), 7.36-7.29 (m, 8H), 4.72 (s, 2H), 2.66 (t, *J* = 7.6 Hz, 2H), 1.68-1.62 (m, 2H), 1.37-1.31 (m, 4H), 0.91 (t, *J* = 6.8 Hz, 3H) ppm; ^{13}C NMR (125 MHz, $CDCl_3$): δ 151.2, 149.2, 147.7, 143.9, 141.1, 137.8, 136.3, 131.4, 130.8, 129.2, 129.1, 128.8, 128.7, 128.6, 127.9, 127.8, 126.9, 126.6, 125.9, 120.3, 116.8, 56.0, 35.8, 31.5, 31.1, 22.5, 14.0 ppm; HRMS (+ESI) m/z calcd for $[C_{35}H_{32}N_4O_3S]^+$ 589.2273, found m/z 589.2313 ($[M+H]^+$).

2-(((4-(4-(4-phenoxyphenyl)-1H-1,2,3-triazol-1-yl)phenyl)sulfonyl)methyl)-4,5-diphenyloxazole ***(44):***

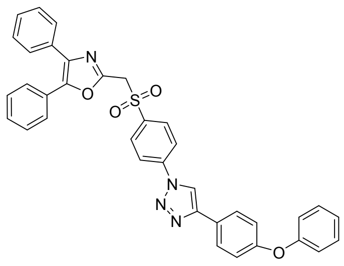

White solid; yield 97%; mp = 201-203°C; R_f = 0.52 (hexane/ethyl acetate, 1:1); FT-IR (neat): 3131, 2930, 1592, 1488, 1329, 1243, 1162, 1035, 822, 761 cm^{-1}; ^{1}H NMR (400 MHz, $CDCl_3$): δ 8.15 (s, 1H), 8.03 (dd, J = 8.4 Hz, 21.2 Hz, 4H), 7.87 (d, J = 8.8 Hz, 2H), 7.53-7.49 (m, 4H), 7.40-7.36 (m, 2H), 7.34-7.32 (m, 6H), 7.18-7.14 (m, 1H), 7.12-7.06 (m, 4H), 4.72 (s, 2H) ppm; ^{13}C NMR (125 MHz, $CDCl_3$): δ 158.1, 156.6, 151.1, 148.7, 147.8, 141.1, 137.8, 136.3, 131.4, 130.8, 129.9, 129.2, 128.8, 128.65, 128.56, 127.9, 127.8, 127.5, 126.6, 124.4, 123.8, 120.3, 119.3, 119.0, 116.6, 56.0 ppm; HRMS (+ESI) m/z calcd for $[C_{36}H_{26}N_4O_4S]^+$ 611.1753, found 611.1758 ($[M+H]^+$).

2-(((4-(4-(3,5-bis(trifluoromethyl)phenyl)-1H-1,2,3-triazol-1-yl)phenyl)sulfonyl)methyl)-4,5-diphenyloxazole ***(45):***

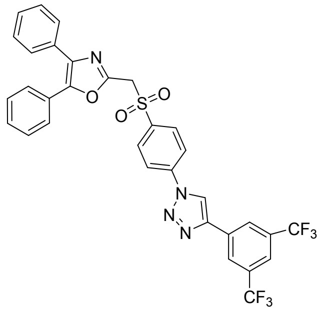

White solid; yield 63%; mp = 128-131°C; R_f = 0.45 (hexane/ethyl acetate, 1:1); FT-IR (neat): 3144, 3052, 3005, 2929, 1597, 1507, 1370, 1323, 1285, 1129, 1087 cm^{-1}; ^{1}H NMR (400 MHz, $CDCl_3$): δ 8.38 (s, 3H), 8.05 (dd, J = 8.4 Hz, 26.4 Hz, 4H), 7.94 (s, 1H), 7.52-7.49 (m, 4H), 7.34-7.32 (m, 6H), 4.73 (s, 2H) ppm; ^{13}C NMR (175 MHz, $CDCl_3$): δ 151.1, 147.8, 146.3, 140.7, 138.4, 136.3, 132.5 (q, J = 33.7 Hz), 131.8, 131.4, 130.9, 129.3, 128.8, 128.7, 128.60, 128.58,

127.8, 127.75, 126.6, 125.8 (q, *J* = 2.62 Hz), 125.4, 123.2 (q, *J* = 270.9 Hz), 122.2 (q, *J* = 4.02 Hz), 120.6, 118.6, 56.0 ppm; HRMS (+ESI) m/z calcd for $[C_{32}H_{20}F_6N_4O_3S]^+$ 655.1239, found m/z 655.1287 ($[M+H]^+$).

4,5-bis(4-fluorophenyl)-2-(((4-(4-(3-fluorophenyl)-1H-1,2,3-triazol-1-yl)phenyl)sulfinyl)methyl)oxazole ***(46):***

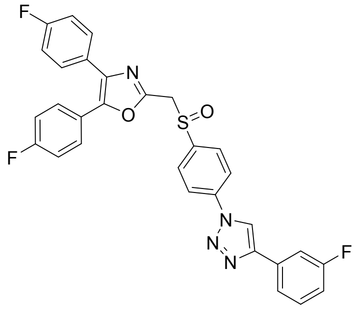

White solid; yield 78%; mp = 205-208°C; R_f = 0.53 (methanol/chloroform, 1:9); FT-IR: 3110, 2927, 1591, 1494, 1236, 1157, 1045, 988, 831 cm^{-1}; ^{1}H NMR (400 MHz, $CDCl_3$): δ 8.19 (s, 1H), 7.98 (d, *J* = 8.0 Hz, 2H), 7.83 (d, *J* = 8.8 Hz, 2H), 7.69-7.63 (m, 2H), 7.53-7.50 (m, 2H), 7.44 (s, 3H), 7.12-7.01 (m, 5H), 4.36 (dd, *J* = 14.0 Hz, 42.0 Hz, 2H) ppm; ^{13}C NMR (125 MHz, THF, 45 °C): δ 165.2 (d, *J* = 243.1 Hz), 164.8 (d, *J* = 247.0 Hz), 164.5 (d, *J* = 246.0 Hz), 156.1, 148.9, 147.6, 146.5, 141.2, 136.8, 135.0 (d, *J* = 8.5 Hz), 132.3 (d, *J* = 7.6 Hz), 131.3 (d, *J* = 8.6 Hz), 130.7 (d, *J* = 8.5 Hz), 130.2, 127.8, 126.8, 123.1, 122.2, 120.8, 117.4 (d, *J* = 21.8 Hz), 117.0 (d, *J* = 21.8 Hz), 116.4 (d, *J* = 20.8 Hz), 114.0 (d, *J* = 22.9 Hz), 57.0 ppm; HRMS (+ESI) m/z calcd for $[C_{30}H_{19}F_3N_4O_2S]$ 557.1254, found 557.1261.

4,5-bis(4-fluorophenyl)-2-(((4-(4-(4-fluorophenyl)-1H-1,2,3-triazol-1-yl)phenyl)sulfinyl)methyl)oxazole ***(47):***

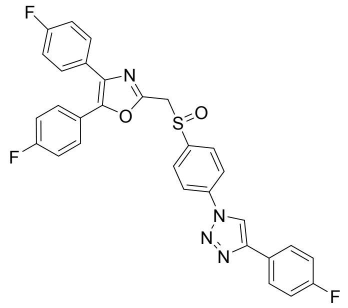

White solid; yield 83%; mp = 199-201°C; R_f = 0.54 (methanol/chloroform, 1:9); FT-IR: 3110, 2932, 1568, 1516, 1494, 1404, 1225, 1053, 1039, 825 cm^{-1}; ^{1}H NMR (400 MHz, $CDCl_3$): δ 8.15 (s, 1H), 7.97 (d, *J* = 8.0 Hz, 2H), 7.88 (d, *J* = 6.4 Hz, 2H), 7.82 (d, *J* = 7.6 Hz, 2H), 7.53-7.49 (m, 2H), 7.44-7.41 (m, 2H), 7.17 (t, *J* = 8.4 Hz, 2H), 7.04 (dd, *J* = 8.4 Hz, 17.6 Hz, 4H), 4.37 (dd, *J* = 13.2 Hz, 41.2 Hz, 2H) ppm; ^{13}C NMR (125 MHz, $CDCl_3$): δ 162.9 (d, *J* = 250.9 Hz), 162.7 (d, *J* = 247.0 Hz), 152.9, 148.1, 146.3, 143.4, 139.4, 135.1, 129.6, 128.6, 127.7, 127.5, 126.1, 124.1, 120.9, 116.9, 116.2, 115.9, 115.8 ppm; HRMS (+ESI) m/z calcd for [$C_{30}H_{19}F_3N_4O_2S$] 557.1254, found 557.1245.

*4,5-bis(4-fluorophenyl)-2-(((4-(4-(2-methoxyphenyl)-1H-1,2,3-triazol-1-yl)phenyl)sulfinyl)methyl)oxazole (**48**):*

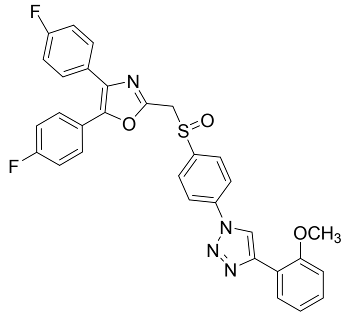

White solid; yield 92%; mp = 184-186°C; R_f = 0.64 (methanol/chloroform, 1:9); FT-IR: 3138, 2997, 1596, 1489, 1252, 1048, 1027, 827 cm^{-1}; ^{1}H NMR (400 MHz, $CDCl_3$): δ 8.48 (s, 1H), 8.43 (d, *J* = 7.2 Hz, 1H), 8.01 (d, *J* = 8.0 Hz, 2H), 7.81 (d, *J* = 7.6 Hz, 2H), 7.52-7.49 (m, 2H), 7.43-7.36 (m, 3H), 7.14 (t, *J* = 8.0 Hz, 1H), 7.03 (dd, *J* = 8.8 Hz, 17.6 Hz, 5H), 4.35 (dd, *J* = 13.6 Hz,

43.2 Hz, 2H), 3.99 (s, 3H) ppm; ^{13}C NMR (125 MHz, $CDCl_3$): δ 162.9 (d, J = 249.8 Hz), 162.7 (d, J = 248.0 Hz), 155.8, 153.1, 146.3, 144.3, 142.8, 139.8, 135.1, 129.7, 129.6 (d, J = 8.5 Hz), 129.56, 128.6 (d, J = 8.6 Hz), 127.9, 127.5, 125.9, 124.1, 121.2, 121.0, 120.5, 118.6, 115.9 (d, J = 22.0 Hz), 115.8 (d, J = 22.0 Hz), 110.9, 56.0, 55.5 ppm; HRMS (+ESI) m/z calcd for [$C_{31}H_{22}F_2N_4O_3S$] 569.1453, found 569.1448.

*4,5-bis(4-chlorophenyl)-2-(((4-(4-(3-fluorophenyl)-1H-1,2,3-triazol-1-yl)phenyl)sulfinyl)methyl)oxazole (**49**):*

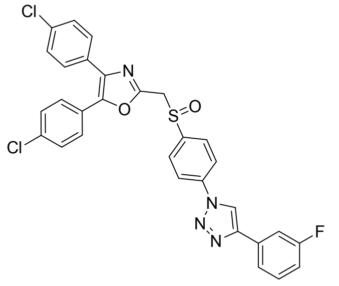

White solid; yield 52%; mp = 219-222°C; R_f = 0.56 (methanol/chloroform, 1:9); FT-IR: 2997, 2932, 1592, 1497, 1399, 1094, 1037, 1015, 823 cm^{-1}; ^{1}H NMR (500 MHz, THF, 45 °C): δ 8.81 (s, 1H), 8.09 (d, J = 8.0 Hz, 2H), 7.87 (d, J = 8.5 Hz), 7.78 (d, J = 7.0 Hz, 1H), 7.71 (d, J = 10.0 Hz, 1H), 7.55 (d, J = 7.5 Hz, 2H), 7.48-7.43 (m, 3H), 7.36 (t, J = 7.5 Hz, 4H), 7.08 (t, J = 9.0 Hz, 1H), 4.44 (s, 2H) ppm; ^{13}C NMR (125 MHz, THF, 45 °C): δ 154.6, 144.8, 139.4, 135.4, 130.3, 129.0, 128.8, 128.5, 128.0, 127.0, 125.8, 121.2, 120.3, 119.5, 118.5, 114.6, 112.3, 110.0 ppm; HRMS (+ESI) m/z calcd for [$C_{30}H_{19}Cl_2FN_4O_2S$] 589.0663, found 589.0670.

*4,5-bis(4-chlorophenyl)-2-(((4-(4-(4-fluorophenyl)-1H-1,2,3-triazol-1-yl)phenyl)sulfinyl)methyl)oxazole (**50**):*

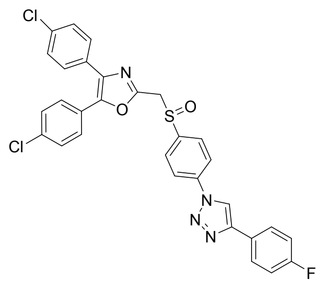

White solid; yield 93%; mp = 212-214°C; R_f = 0.56 (methanol/chloroform, 1:9); FT-IR: 2997, 2937, 1493, 1403, 1228, 1093, 1040, 817 cm^{-1}; ^1H NMR (400 MHz, $CDCl_3$): δ 9.34 (s, 1H), 8.14 (d, *J* = 5.2 Hz, 2H), 7.99 (s, 2H), 7.91 (d, *J* = 5.6 Hz, 2H), 7.47-7.45 (m, 4H), 7.41-7.31 (m, 6H), 4.77 (d, *J* = 11.2 Hz, 1H), 4.63 (d, *J* = 11.2 Hz, 1H) ppm; ^{13}C NMR (125 MHz, THF, 45 °C): δ 162.8 (d, *J* = 248.5 Hz), 154.6, 147.3, 145.9, 144.6, 139.5, 135.4, 134.7, 133.9, 130.7, 129.0, 128.9, 128.5, 128.1, 127.3, 125.8, 120.3, 117.7, 115.4 (d, *J* = 21.8 Hz), 55.2 ppm; HRMS (+ESI) m/z calcd for [$C_{30}H_{19}Cl_2FN_4O_2S$] 589.0663, found 589.0668.

*4,5-bis(4-chlorophenyl)-2-(((4-(4-(2-methoxyphenyl)-1H-1,2,3-triazol-1-yl)phenyl)sulfinyl)methyl)oxazole (**51**):*

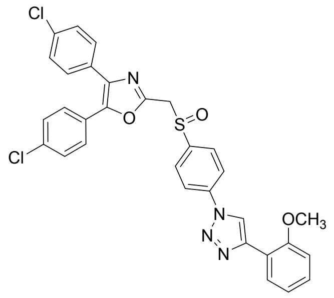

White solid; yield 78%; mp = 188-191°C; R_f = 0.61 (methanol/chloroform, 1:9); FT-IR: 3077, 1591, 1490, 1400, 1248, 1091, 1026, 1053, 829 cm^{-1}; ^1H NMR (400 MHz, $CDCl_3$): δ 8.48 (s, 1H), 8.43 (d, *J* = 7.6 Hz, 1H), 8.01 (d, *J* = 8.4 Hz, 2H), 7.81 (d, *J* = 8.4 Hz, 2H), 7.48 (d, *J* = 8.0 Hz, 2H), 7.39-7.29 (m, 7H), 7.16-7.12 (m, 1H), 7.04 (d, *J* = 8.0 Hz, 1H), 4.36 (dd, *J* = 13.6, 37.2 Hz, 2H), 4.00 (s, 3H) ppm; ^{13}C NMR (125 MHz, $CDCl_3$): δ 155.8, 153.5, 146.4, 144.4, 142.8, 139.8, 135.5, 135.2, 134.6, 129.8, 129.6, 129.2, 129.1, 129.0, 127.9, 127.8, 126.3, 126.0, 125.9,

121.2, 120.9, 120.5, 119.9, 118.5, 110.9, 55.9, 55.5 ppm; HRMS (+ESI) m/z calcd for [$C_{31}H_{22}Cl_2N_4O_3S$] 601.0862, found 601.0864.

*4,5-bis(4-fluorophenyl)-2-(((4-(4-(3-fluorophenyl)-1H-1,2,3-triazol-1-yl)phenyl)sulfonyl)methyl)oxazole (**52**):*

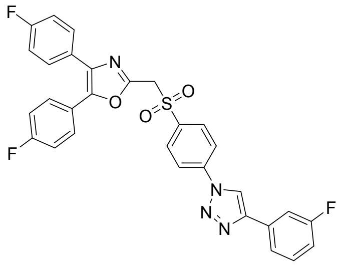

White solid; yield 79%; mp = 255-256°C; R_f = 0.40 (methanol/chloroform, 1:9); FT-IR: 3156, 2927, 1594, 1494, 1315, 1298, 1233, 1148, 1041, 831 cm^{-1}; ^{1}H NMR (400 MHz, $CDCl_3$): δ 8.25 (s, 1H), 8.08 (d, *J* = 8.8 Hz, 2H), 8.03 (d, *J* = 8.8 Hz, 2H), 7.69-7.63 (m, 2H), 7.48 (s, 5H), 7.11-7.02 (m, 5H), 4.70 (s, 2H) ppm; ^{13}C NMR (125 MHz, THF, 45 °C): δ 163.3 (d, *J* = 244.1 Hz), 163.1 (d, *J* = 248.0 Hz), 162.7 (d, *J* = 246.1 Hz), 152.6, 147.3, 146.1, 141.1, 138.9, 135.1, 132.9 (d, *J* = 8.6 Hz), 130.6, 130.5 (d, *J* = 7.6 Hz), 129.4 (d, *J* = 8.6 Hz), 128.9 (d, *J* = 7.6 Hz), 128.1, 124.7, 121.2, 119.9, 118.7, 115.7 (d, *J* = 22.0 Hz), 115.2 (d, *J* = 21.0 Hz), 114.7 (d, *J* = 22.0 Hz), 112.1 (d, *J* = 23.0 Hz), 54.9 ppm; HRMS (+ESI) m/z calcd for [$C_{30}H_{19}F_3N_4O_3S$] 573.1203, found 573.1192.

*4,5-bis(4-fluorophenyl)-2-(((4-(4-(4-fluorophenyl)-1H-1,2,3-triazol-1-yl)phenyl)sulfonyl)methyl)oxazole (**53**):*

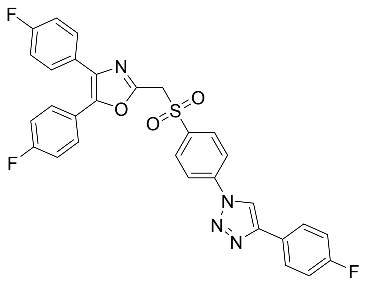

White solid; yield 83%; mp = 235-237°C; R_f = 0.72 (methanol/chloroform, 1:9); FT-IR: 3147, 1596, 1493, 1321, 1225, 1149, 1035, 835 cm^{-1}; ^{1}H NMR (400 MHz, $CDCl_3$): δ 8.20 (s, 1H), 8.05 (dd, *J* = 8.4 Hz, 21.2 Hz, 4H), 7.91-7.87 (m, 2H), 7.49-7.46 (m, 4H), 7.18 (t, *J* = 8.8 Hz, 2H), 7.04 (dd, *J* = 8.4 Hz, 16.8 Hz, 4H), 4.70 (s, 2H) ppm; ^{13}C NMR (125 MHz, $CDCl_3$): δ 163.1 (d, *J* = 247.0 Hz), 162.8 (d, *J* = 248.0 Hz), 151.2, 148.3, 146.8, 141.1, 138.0, 135.3, 130.8, 130.2, 129.5 (d, *J* = 8.62 Hz), 128.7 (d, *J* = 8.5 Hz), 127.8 (d, *J* = 8.5 Hz), 127.3, 125.7, 125.3, 123.9, 120.4, 116.9, 116.1 (d, *J* = 21.8 Hz), 115.8 (d, *J* = 21.8 Hz), 55.9 ppm; HRMS (+ESI) m/z calcd for [$C_{30}H_{19}F_3N_4O_3S$] 573.1203; found 573.1205.

*4,5-bis(4-fluorophenyl)-2-(((4-(4-(2-methoxyphenyl)-1H-1,2,3-triazol-1-yl)phenyl)sulfonyl)methyl)oxazole (**54**):*

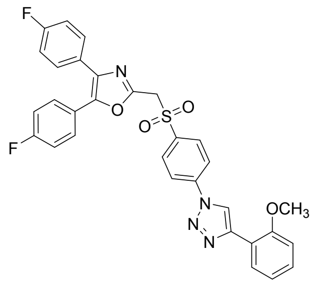

White solid; yield 95%; mp = 200-203°C; R_f = 0.56 (methanol/chloroform, 1:9); FT-IR: 1596, 1515, 1497, 1322, 1253, 1152, 1032, 837, 744 cm^{-1}; ^{1}H NMR (400 MHz, $CDCl_3$): δ 8.52 (s, 1H), 8.42 (d, *J* = 8.0 Hz, 1H), 8.06 (s, 4H), 7. 47 (m, 4H), 7.39 (t, *J* = 8.0 Hz, 1H), 7.14 (t, *J* = 7.2 Hz, 1H), 7.04 (m, 5H), 4.69 (s, 2H), 4.00 (s, 3H) ppm; ^{13}C NMR (125 MHz, $CDCl_3$): δ 163.07 (d, *J* = 249.8 Hz), 162.8 (d, *J* = 248.0 Hz), 155.9, 151.3, 146.8, 144.6, 141.4, 137.5, 135.2, 130.7, 129.7 (d, *J* = 8.6 Hz), 129.6, 128.7 (d, *J* = 8.6 Hz), 127.9, 127.3, 124.0, 121.2, 120.4, 118.3, 116.1, (d, *J* = 22.0 Hz), 115.8 (d, *J* = 22.0 Hz), 110.9, 55.9, 55.5 ppm; HRMS (+ESI) m/z calcd for [$C_{31}H_{22}F_2N_4O_4S$] 585.1403, found 585.1402.

*4,5-bis(4-chlorophenyl)-2-(((4-(4-(3-fluorophenyl)-1H-1,2,3-triazol-1-yl)phenyl)sulfonyl)methyl)oxazole (**55**):*

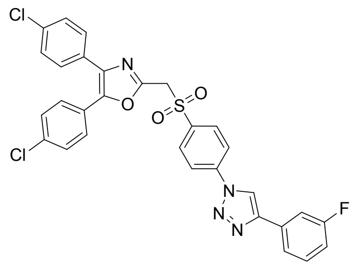

Off-white solid; yield 65%; mp = 270-272°C; R_f = 0.64 (methanol/chloroform, 1:9); FT-IR: 2937, 1588, 1480, 1310, 1286, 1161, 1150, 1088, 826 cm^{-1}; ^1H NMR (500 MHz, THF, 45 °C): δ 8.90 (s, 1H), 8.14 (dd, J = 8.5 Hz, 26.5 Hz, 4H), 7.79 (d, J = 8.0 Hz, 1H), 7.71 (d, J = 10.0 Hz, 1H), 7.52 (d, J = 8.0 Hz, 2H), 7.49-7.44 (m, 3H), 7.39 (d, J = 8.5 Hz, 2H), 7.34 (d, J = 8.5 Hz, 2H), 7.09 (t, J = 8.0 Hz, 1H), 4.91 (s, 2H) ppm; ^{13}C NMR (125 MHz, THF, 45 °C): δ 163.3 (d, J = 244.1 Hz), 152.9, 147.4, 146.3, 141.1, 139.0, 135.5, 134.9, 134.0, 132.9, 130.6, 130.5, 130.4, 128.99, 128.96, 128.6, 128.1, 126.9, 121.3, 119.9, 118.7, 114.7 (d, J = 21.8 Hz), 112.2 (d, J = 23.8 Hz), 54.9 ppm; HRMS (+ESI) m/z calcd for [$C_{30}H_{19}F_3N_4O_2S$] 605.0612, found 605.0613.

*4,5-bis(4-chlorophenyl)-2-(((4-(4-(4-fluorophenyl)-1H-1,2,3-triazol-1-yl)phenyl)sulfonyl)methyl)oxazole (**56**):*

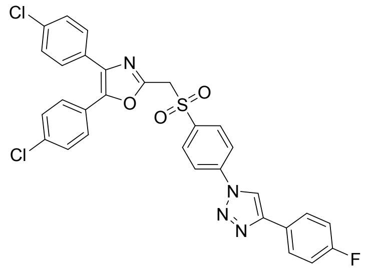

White solid; yield 81%; mp = 245-247°C; R_f = 0.63 (methanol/chloroform, 1:9); FT-IR: 3142, 1596, 1493, 1304, 1231, 1149, 1093, 829 cm^{-1}; ^1H NMR (400 MHz, $CDCl_3$): δ 8.19 (s, 1H), 8.04 (dd, J = 8.0 Hz, 21.2 Hz, 4H), 7.89 (t, J = 6.4 Hz, 2H), 7.44 (d, J = 8.0 Hz, 4H), 7.35-7.31 (m,

4H), 7.18 (t, *J* = 8.0 Hz, 2H), 4.70 (s, 2H) ppm; ^{13}C NMR (125 MHz, THF, 45 °C): δ 162.8 (d, *J* = 245.1 Hz), 153.0, 147.6, 146.3, 141.2, 138.8, 135.5, 134.9, 134.0, 130.6, 130.4, 129.0 (d, *J* = 3.8 Hz), 128.6, 128.1, 127.4 (d, *J* = 8.6 Hz), 126.9, 126.8, 119.8, 117.9, 115.5 (d, *J* = 22.0 Hz), 54.9 ppm; HRMS (+ESI) m/z calcd for [$C_{30}H_{19}Cl_2FN_4O_3S$] 605.0612, found 605.0615.

4,5-bis(4-chlorophenyl)-2-(((4-(4-(2-methoxyphenyl)-1H-1,2,3-triazol-1-yl)phenyl)sulfonyl)methyl)oxazole ***(57)****:*

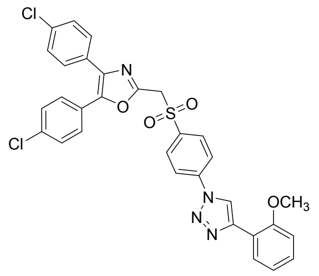

White solid; yield 52%; mp = 236-238°C; R_f = 0.65 (methanol/chloroform, 1:9); FT-IR: 1596, 1498, 1403, 1322, 1251, 1150, 1092, 827 cm^{-1}; ^{1}H NMR (400 MHz, $CDCl_3$): δ 8.51 (s, 1H), 8.41 (d, *J* = 8.0 Hz, 1H), 8.04 (s, 4H), 7.44-7.41 (m, 4H), 7.37 (d, *J* = 8.0 Hz, 1H), 7.33-7.32 (m, 4H), 7.13 (t, *J* = 8.0 Hz, 1H), 7.03 (d, *J* = 8.4 Hz, 1H), 4.68 (s, 2H), 3.99 (s, 3H) ppm; ^{13}C NMR (125 MHz, $CDCl_3$): δ 155.9, 151.6, 146.9, 144.6, 141.5, 137.5, 135.6, 135.5, 134.7, 130.7, 129.7, 129.6, 129.2, 129.0, 128.0, 127.9, 126.1, 121.2, 120.4, 120.3, 118.3, 110.9, 55.9, 55.5 ppm; HRMS (+ESI) m/z calcd for [$C_{31}H_{22}Cl_2N_4O_4S$] 617.0812, found 617.0818.

Analytical data for compounds 58-72:

2-oxo-1,2-diphenylethyl 2-azidobenzoate ***(58):***

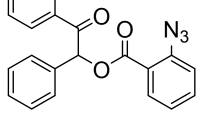

White solid; yield 95%; mp = 113-115°C; R_f = 0.4 (hexane/ethyl acetate, 3:1); FT-IR: 3065, 2118, 2090, 1714, 1686, 1596, 1487, 1235 cm^{-1}; ^1H NMR (400 MHz, $CDCl_3$) δ 8.08 (dd, *J* = 1.2 Hz, 7.6 Hz, 1H), 7.98 (dd, *J* = 1.2 Hz, 8.2 Hz, 2H), 7.57-7.51 (m, 4H), 7.44-7.35 (m, 5H), 7.24-7.17 (m. 1H), 7.08 (s, 1H) ppm; ^{13}C NMR (100 MHz, $CDCl_3$) 193.6, 164.3, 140.6, 134.7, 133.7, 133.5, 133.4, 132.4, 129.4, 129.2, 128.9, 128.7, 128.6, 124.5, 121.4, 119.8, 78.1 ppm; LRMS for [$C_{21}H_{15}N_3O_5$] found 358 (M+H).

2-oxo-1,2-diphenylethyl 3-azidobenzoate ***(59)*****:** See: Ref.1

*2-oxo-1,2-diphenylethyl 4-azidobenzoate****(60)*****:** See: Ref. 1

1,2-bis(4-chlorophenyl)-2-oxoethyl 2-azidobenzoate ***(61)*****:**

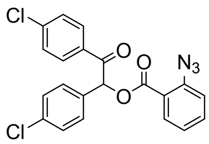

Yellow oil; yield 84%; R_f = 0.32 (hexane/ethyl acetate, 9:1); FT-IR: 3069, 2970, 2120, 1724, 1693, 1587, 1488, 1446, 1238, 1071 cm^{-1}; ^1H NMR (400 MHz, $CDCl_3$) δ 7.90 (d, *J* = 8.8 Hz, 2H), 7.86 (d, *J* = 8.0 Hz, 1H), 7.73 (s, 1H), 7.49-7.38 (m, 7H), 7.23 (s, 1H), 6.98 (s, 1H) ppm; ^{13}C NMR (100 MHz, $CDCl_3$) δ 192.0, 165.0, 140.7, 140.4, 135.8, 132.6, 131.6, 130.8, 130.1, 129.9, 129.6, 129.2, 126.3, 123.9, 120.3, 77.3 ppm; LRMS for [$C_{21}H_{13}Cl_2N_3O_3$] found 448 (M+Na).

1,2-bis(4-chlorophenyl)-2-oxoethyl 3-azidobenzoate ***(62)***[2]**:** See Ref. 2.

1,2-bis(4-chlorophenyl)-2-oxoethyl 4-azidobenzoate ***(63)***[2]**:** See Ref. 2.

1,2-bis(4-fluorophenyl)-2-oxoethyl 2-azidobenzoate ***(64)*****:**

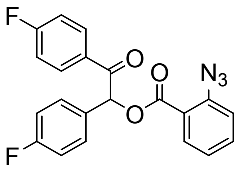

Yellow oil; yield 93%; R_f = 0.34 (hexane/ethyl acetate, 9:1); FT-IR: 3083, 2121, 1725, 1692, 1595, 1508, 1224 cm^{-1}; ^{1}H NMR (400 MHz, $CDCl_3$) δ 8.04-7.99 (m, 3H), 7.57-7.52 (m, 3H), 7.24-7.18 (m, 2H), 7.12-7.06 (m, 4H), 7.01 (s, 1H) ppm; ^{13}C NMR (100 MHz, $CDCl_3$) δ 192.0, 165.9 (d, *J* = 255.1 Hz), 164.3, 163.3 (d, *J* = 248.3 Hz), 140.7, 133.8, 132.3, 131.5 (d, *J* = 9.1 Hz), 130.9, 130.5 (d, *J* = 6.9 Hz), 129.2, 124.5, 121.2, 119.8, 116.2 (d, *J* = 22.8 Hz), 115.9 (d, *J* = 22.7 Hz), 77.1 ppm; LRMS for [$C_{21}H_{13}F_2N_3O_3$] found 393.

1,2-bis(4-fluorophenyl)-2-oxoethyl 3-azidobenzoate ***(65)*****[2]:** See Ref. 2.

1,2-bis(4-fluorophenyl)-2-oxoethyl 4-azidobenzoate ***(66)*****[2]:** See Ref. 2.

1,2-bis(4-methoxyphenyl)-2-oxoethyl 2-azidobenzoate ***(67)*****:**

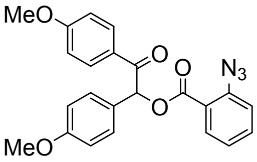

Off-white solid; yield 98%; mp = 83-85°C; R_f = 0.30 (hexane/ethyl acetate, 3:1); FT-IR: 2119, 1714, 1690, 1674, 1596, 1511, 1233 cm^{-1}; ^{1}H NMR (400 MHz, $CDCl_3$) δ 8.06 (d, *J* = 7.6 Hz, 1H), 7.97 (dd, *J* = 1.6 Hz, 8.8 Hz, 2H), 7.53 (t, *J* = 7.6 Hz, 1H), 7.47 (dd, *J* = 1.6 Hz, 8.4 Hz, 2H), 7.25-7.16 (m, 2H), 7.02 (s, 1H), 6.91-6.87 (m, 4H), 3.83 (s, 3H), 3.78 (s, 3H) ppm; ^{13}C

NMR (100 MHz, $CDCl_3$) δ 191.9, 164.4, 163.7, 160.3, 133.5, 132.4, 131.2, 130.2, 127.5, 125.9, 124.5, 119.7, 114.5, 113.9, 77.5, 55.4, 55.3 ppm; HRMS (ESI) m/z calcd for [$C_{23}H_{19}N_3O_5Li$] 424.1485, found 424.1479.

1,2-bis(4-methoxyphenyl)-2-oxoethyl 3-azidobenzoate ***(68)[2]:*** See Ref. 2.

1,2-bis(4-methoxyphenyl)-2-oxoethyl 4-azidobenzoate ***(69)[2]*****:** See Ref. 2.

1,2-di(furan-2-yl)-2-oxoethyl 2-azidobenzoate ***(70)*****:**

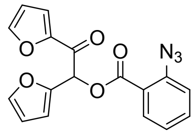

Pale yellow solid; yield 61%; mp = 81-84°C; R_f = 0.50 (hexane/ethyl acetate, 3:1); FT-IR: 3147, 3127, 2103, 2129, 1728, 1678, 1580, 1253cm^{-1}; ^{1}H NMR (400 MHz, $CDCl_3$) δ 8.03 (dd, *J* = 1.6 Hz, 8.0 Hz, 1H), 7.58 (d, *J* = 1.6 Hz, 1H), 7.52 (td, 1.6 Hz, 8.0 Hz, 1H), 7.46 (d, *J* = 2.0 Hz, 1H), 7.29 (d, *J* = 3.6 Hz, 1H), 7.21 (d, *J* = 8.0 Hz, 1H), 7.16 (td, *J* = 0.8 Hz, 7.6 Hz, 1H), 6.97 (s, 1H), 6.57 (d, *J* = 2.8 Hz, 1H), 6.52-6.51 (m, 1H), 6.39-6.38 (m, 1H) ppm; ^{13}C NMR (125 MHz, $CDCl_3$) δ 179.4, 163.9, 150.3, 147.3, 146.4, 144.3, 140.7, 133.8, 132.4, 124.5, 121.1, 119.8, 119.3, 112.5, 111.9, 111.1, 70.9 ppm; LRMS for [$C_{17}H_{11}N_3O_5$] found 337.

1,2-di(furan-2-yl)-2-oxoethyl 3-azidobenzoate ***(71)*****:**

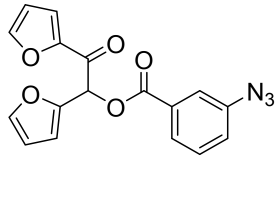

Off-white solid; yield 79%; mp = 78-81°C; R_f = 0.53 (hexane/ethyl acetate, 3:1); FT-IR: 3127, 3097, 2114, 1720, 1673, 1582, 1463, 1232 cm^{-1}; ^1H NMR (400 MHz, $CDCl_3$) δ 7.88 (d, *J* = 7.2 Hz, 1H), 7.75 (s, 1H), 7.59 (d, *J* = 0.8 Hz, 1H), 7.49 (s, 1H), 7.43 (t, *J* = 8.0 Hz, 1H), 7.31 (d, *J* = 3.6 Hz, 1H), 7.23-7.21 (m, 1H), 6.97 (s, 1H), 6.58 (d, *J* = 3.2 Hz, 1H), 6.54 (s, 1H), 6.42 (s, 1H) ppm; ^{13}C NMR (125 MHz, $CDCl_3$) δ 179.30, 164.8, 150.2, 147.4, 146.4, 144.4, 140.6, 130.8, 129.9, 126.4, 123.9, 120.3, 119.4, 112.6, 112.0, 111.2, 71.1 ppm; LRMS for [$C_{17}H_{11}N_3O_5$] found 337.

*1,2-di(furan-2-yl)-2-oxoethyl 4-azidobenzoate **(72)***:

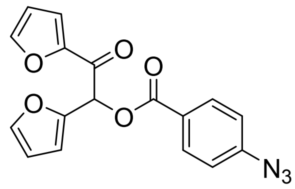

Pale yellow solid; yield 45%; mp = 93-96°C; R_f = 0.53 (hexane/ethyl acetate, 3:1); FT-IR: 2140, 2111, 1719, 1689, 1599, 1501, 1461, 1254 cm^{-1}; ^1H NMR (400 MHz, $CDCl_3$) δ 8.09 (d, *J* = 2.4 Hz, 6.8 Hz, 1H), 7.59 (t, *J* = 0.8 Hz, 1H), 7.48 (t, *J* = 0.8 Hz, 1H), 7.31 (dd, *J* = 0.8 Hz, 3.2 Hz, 1H), 7.06 (dd, *J* = 2.0 Hz, 6.8 Hz, 2H), 6.97 (s, 1H), 6.56 (d, *J* = 3.6 Hz, 1H), 6.54 (dd, *J* = 1.6 Hz, 6.8 Hz, 1H), 6.41 (q, *J* = 1.6 Hz, 1H) ppm; ^{13}C NMR (125 MHz, $CDCl_3$) δ 179.5, 164.8, 150.2, 147.3, 146.5, 145.3, 144.3, 131.8, 125.5, 119.3, 118.9, 112.6, 111.9, 111.1, 70.9 ppm; LRMS for [$C_{17}H_{11}N_3O_5$] found 337.

Analytical data for compounds 73-87:

2-(2-azidophenyl)-4,5-diphenyloxazole ***(73)*****:**

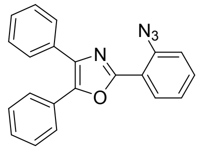

Pale yellow soild; yield 84%; mp = 83-85°C; R_f = 0.22 (hexane/ethyl acetate, 9:1); FT-IR: 3059, 2121, 2089, 1581, 1501, 1291, 1070 cm^{-1}; ^{1}H NMR (400 MHz, $CDCl_3$) δ 8.32 (d, *J* = 7.6 Hz, 1H), 7.94 (dd, *J* = 8.0 Hz, 18.4 Hz, 4H), 7.70 (t, *J* = 7.6 Hz, 1H), 7.65-7.52 (m, 7H), 7.47 (t, *J* = 8.0 Hz, 1H) ppm; ^{13}C NMR (100 MHz, $CDCl_3$) δ 157.8, 145.9, 138.2, 136.7, 134.9, 132.5, 131.4, 130.7, 129.9, 129.0, 128.9, 128.7, 128.69, 128.64, 128.3, 126.6, 124.9, 119.8, 119.2 ppm; HRMS (ESI) m/z calcd for [$C_{21}H_{15}N_4O$] 339.1240, found 339.1242.

2-(3-azidophenyl)-4,5-diphenyloxazole ***(74)*****:** See: Ref. 1

2-(4-azidophenyl)-4,5-diphenyloxazole ***(75)*****:** See: Ref. 1

2-(2-azidophenyl)-4,5-bis(4-chlorophenyl)oxazole ***(76)*****:**

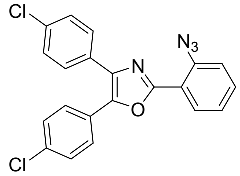

Pale yellow solid; yield 41%; mp = 105-107°C; R_f = 0.29 (hexane/ethyl acetate, 9:1); FT-IR: 3069, 2117, 2091, 1582, 1479, 1292, 1091, 828 cm^{-1}; ^{1}H NMR (400 MHz, $CDCl_3$) δ 7.90 (d, *J* = 7.6 Hz, 1H), 7.78 (s, 1H), 7.64 (d, *J* = 8.8 Hz, 2H), 7.58 (d, *J* = 8.0 Hz, 2H), 7.48 (t, *J* = 8.0 Hz,

1H), 7.40-7.38 (m, 4H), 7.14 (d, J = 8.4 Hz, 1H) ppm; ^{13}C NMR (100 MHz, $CDCl_3$) δ 158.3, 145.1, 138.4, 136.1, 134.9, 134.5, 131.8, 131.4, 130.8, 129.6, 129.4, 129.3, 129.1, 127.9, 127.2, 125.1, 119.9, 118.9 ppm; HRMS (ESI) m/z calcd for [$C_{21}H_{13}Cl_2N_4O$] 407.0461, found 407.0458.

2-(3-azidophenyl)-4,5-bis(4-chlorophenyl)oxazole ***(77)***[2]**:** See Ref. 2.

2-(4-azidophenyl)-4,5-bis(4-chlorophenyl)oxazole ***(78)***[2]**:** See Ref. 2.

2-(2-azidophenyl)-4,5-bis(4-fluorophenyl)oxazole ***(79)*****:**

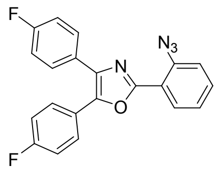

Pale yellow solid; yield 71%; mp = 100-101°C; R_f = 0.24 (hexane/ethyl acetate, 9:1); FT-IR: 2116, 2084, 1580, 1513, 1495, 1223, 1154 cm^{-1}; ^{1}H NMR (400 MHz, $CDCl_3$) δ 8.07 (dd, J = 1.2 Hz, 8.0 Hz, 1H), 7.69-7.61 (m, 4H), 7.52-7.48 (m, 1H), 7.32 (d, J = 8.4 Hz, 1H), 7.28-7.24 (m, 1H), 7.1 (t, J = 8.8 Hz, 4H) ppm; ^{13}C NMR (100 MHz, $CDCl_3$) δ 162.6 (d, J = 240.7 Hz), 157.7, 144.7, 138.0, 135.3, 131.3, 130.4, 129.8 (d, J = 8.3 Hz), 128.4 (d, J = 7.6 Hz), 128.2, 124.7, 119.7, 118.8, 115.8 (d, J = 22.7 Hz), 115.5 (d, J = 24.3 Hz) ppm; LRMS for $C_{21}H_{13}F_2N_4O$ found 374.

2-(3-azidophenyl)-4,5-bis(4-fluorophenyl)oxazole ***(80)***[2]**:** See Ref. 2.

2-(4-azidophenyl)-4,5-bis(4-fluorophenyl)oxazole ***(81)***[2]**:** See Ref. 2.

2-(2-azidophenyl)-4,5-bis(4-methoxyphenyl)oxazole ***(82)*****:**

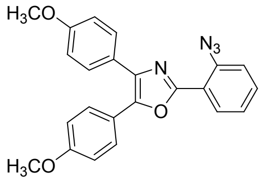

Pale yellow solid; yield 60%; mp = 87-89°C; R_f = 0.28 (hexane/ethyl acetate, 9:1); FT-IR: 3051, 2949, 2118, 2087, 1583, 1497, 1245, 1173 cm^{-1}; ^1H NMR (400 MHz, $CDCl_3$) δ 8.06 (d, *J* = 8.4 Hz, 1H), 7.62 (dd, *J* = 8.4 Hz, 15.2 Hz, 4H), 7.47 (t, *J* = 7.6 Hz, 1H), 7.31-7.22 (m, 2H), 6.93-6.90 (m, 4H), 3.84 (s, 6H) ppm; ^{13}C NMR (100 MHz, $CDCl_3$) δ 159.9, 159.6, 157.3, 145.3, 138.0, 135.3, 131.2, 130.6, 129.5, 128.2, 125.1, 124.9, 121.7, 119.9, 119.5, 114.2, 114.1, 55.4 ppm; HRMS (ESI) m/z calcd for [$C_{23}H_{17}N_4O_3$] 399.1374, found 399.1379.

2-(3-azidophenyl)-4,5-bis(4-methoxyphenyl)oxazole ***(83)***[2]**:** See Ref. 2.

2-(4-azidophenyl)-4,5-bis(4-methoxyphenyl)oxazole ***(84)***[2]**:** See Ref. 2.

2-(2-azidophenyl)-4,5-di(furan-2-yl)oxazole ***(85)*****:**

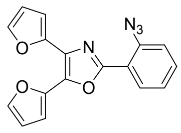

Pale yellow soild; yield 17%; mp = 113-116°C; R_f = 0.33 (hexane/ethyl acetate, 9:1); FT-IR: 3134, 2179, 2125, 1615, 1445, 1301 cm^{-1}; ^1H NMR (400 MHz, $CDCl_3$) δ 8.07 (d, *J* = 8.0 Hz, 1H), 7.57 (d, *J* = 12.0 Hz, 2H), 7.49 (t, *J* = 7.6 Hz, 1H), 7.30 (d, *J* = 8.0 Hz, 1H), 7.27-7.23 (m, 2H), 7.07-7.05 (m, 2H), 6.55 (dt, *J* = 1.6 Hz, 13.2 Hz, 2H) ppm; ^{13}C NMR (125 MHz, $CDCl_3$) δ

160.2, 148.3, 143.0, 142.4, 140.6, 131.6, 129.4, 127.8, 126.7, 126.4, 116.8, 116.2, 112.9, 111.8, 111.5, 109.7, 109.2 ppm; LRMS for $C_{17}H_{10}N_4O_3$ found 318.

2-(3-azidophenyl)-4,5-di(furan-2-yl)oxazole ***(86)*****:**

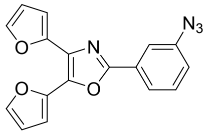

Pale yellow soild; yield 18%; mp = 105-106°C; R_f = 0.41 (hexane/ethyl acetate, 9:1); FT-IR: 3138, 2924, 2141, 2114, 2096, 1612, 1556, 1486 cm^{-1}; ^1H NMR (400 MHz, $CDCl_3$) δ 7.91 (d, *J* = 7.6 Hz, 1H), 7.80 (s, 1H), 7.58 (dd, *J* = 1.2 Hz, 10.0 Hz, 2H), 7.45 (t, *J* = 7.6 Hz, 1H), 7.12-7.10 (m, 1H), 7.05 (dd, *J* = 3.6 Hz, 8.0 Hz, 2H), 6.58 (q, *J* = 1.6 Hz, 1H), 6.55 (q, *J* = 1.6 Hz, 1H) ppm; ^{13}C NMR (125 MHz, $CDCl_3$) δ 159.0, 146.4, 143.3, 142.7, 140.9, 137.6, 130.2, 128.3, 123.2, 121.2, 117.1, 111.9, 111.6, 110.1, 109.6 ppm; LRMS for $C_{17}H_{10}N_4O_3$ found 318.

2-(4-azidophenyl)-4,5-di(furan-2-yl)oxazole ***(87)*****:**

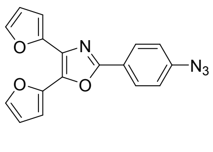

Pale yellow soild; yield 19%; mp = 89-90°C; R_f = 0.29 (hexane/ethyl acetate, 9:1); FT-IR: 3139, 2142, 2109, 2098, 1610, 1559 cm^{-1}; ^1H NMR (400 MHz, $CDCl_3$) δ 8.13 (d, *J* = 6.8 Hz, 2H), 7.57 (d, *J* = 8.0 Hz, 2H), 7.11 (d, *J* = 6.8 Hz, 2H), 7.03 (s, 2H), 6.56 (d, *J* = 11.6 Hz, 2H) ppm; ^{13}C NMR (125 MHz, $CDCl_3$) δ 159.4, 146.5, 143.4, 143.1, 142.6, 142.3, 137.2, 128.3, 123.4, 119.3, 111.8, 111.6, 109.8, 109.5 ppm; LRMS for $C_{17}H_{10}N_4O_3$ found 318.

Analytical data for compounds 89a-89c:

N2, N2, N4, N4-tetraethyl-6-((trimethylsilyl)ethynyl)-1,3,5-triazine-2,4-diamine ***(89a)*****:**

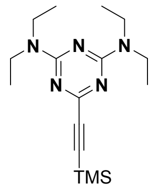

Pale yellow solid; yield 50%; mp = 102-103°C; R_f = 0.56 (hexane/ethyl acetate, 3:1); FT-IR: 2981, 2964, 2932, 1535, 1493, 1359, 1249, 1079 cm^{-1}; ^1H NMR (400 MHz, $CDCl_3$): δ 3.60 (s, 4H), 3.53 (d, J = 6.4 Hz, 4H), 1.15 (t, J = 7.2 Hz, 12H), 0.26 (s, 9H) ppm; ^{13}C NMR (125 MHz, $CDCl_3$): δ 163.7, 158.3, 103.6, 90.6, 40.9, 13.5, 12.8, -0.28 ppm; LRMS (+ESI) for [$C_{16}H_{29}N_5Si$] found 319.

N_2,N_4-bis(2-fluorophenyl)-6-((trimethylsilyl)ethynyl)-1,3,5-triazine-2,4-diamine: ***(89b)*****:**

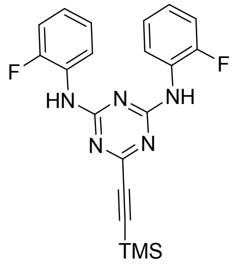

Off-white solid; yield 42%; mp = 244-246 °C; R_f = 0.52 (hexane/ethyl acetate, 7.5:2.5); FT-IR: 3212, 3072, 2964, 1609, 1624, 1514, 1412, 1258, 1191, 842 cm^{-1}; ^1H NMR (400 MHz, $CDCl_3$): δ 8.22 (s, 2H), 7.41 (s, 2H), 7.14-7.04 (m, 6H), 0.30 (s, 9H) ppm; ^{13}C NMR (125 MHz, $CDCl_3$): δ 163.9, 159.3, 153.3 (d, J = 247.0 Hz), 125.9, 124.5, 124.1, 123.0, 115.1 (d, J = 19.1 Hz), 100.9, 95.9, -0.57 (TMS-carbon) ppm; LRMS (+ESI) for [$C_{20}H_{19}F_2N_5Si$] found 395.

N_2,N_4-bis(4-fluorophenyl)-6-((trimethylsilyl)ethynyl)-1,3,5-triazine-2,4-diamine ***(89c):***

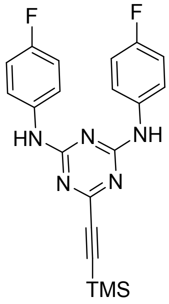

Off-white solid; yield 53%; mp = 226-227°C; R_f = 0.54 (hexane/ethyl acetate, 3:1); FT-IR: 3417, 3390, 3301, 3258, 2126, 1621, 1591, 1565, 1494, 1403, 1206, 828 cm^{-1}; ^{1}H NMR (400 MHz, $CDCl_3$): δ 7.63 (s, 2H), 7.46 (s, 4H), 7.00 (t, J = 8.4 Hz, 4H) ppm.

^{13}C NMR (125 MHz, $CDCl_3$): δ 163.9, 159.3, 153.3 (d, J = 239.4 Hz), 125.9, 124.5, 124.1, 122.9, 1115.1 (d, J = 19.1 Hz), 100.9, 95.9, -0.50 ppm; LRMS (+ESI) for [$C_{20}H_{19}F_2N_5Si$] found 395.

Analytical data for compounds 90-124:

6-(1-(3-(4,5-diphenyloxazol-2-yl)phenyl)-1H-1,2,3-triazol-4-yl)-N2,N2,N4,N4-tetraethyl-1,3,5-triazine-2,4-diamine ***(90)*****:**

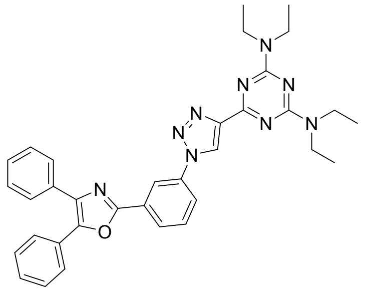

Off-white solid, yield 76%; mp = 96-98°C; R_f = 0.36 (hexane/ethyl acetate, 3:1); FT-IR: 3108, 2973, 2930, 1591, 1540, 1502, 1492, 1374, 816 cm^{-1}; ^{1}H NMR (400 MHz, $CDCl_3$): δ 8.67 (s, 1H), 8.53 (s, 1H), 8.23 (d, J = 8.4 Hz, 1H), 8.02 (d, J = 8.4 Hz, 1H), 7.75-7.66 (m, 5H), 7.45-7.37 (m, 6H), 3.77 (s, 4H), 3.63 (s, 4H), 1.23 (s, 12H) ppm; ^{13}C NMR (125 MHz, $CDCl_3$): δ

164.4, 164.2, 158.7, 148.9, 146.3, 137.6, 137.0, 132.2, 130.4, 128.9, 128.8, 128.53, 128.48, 128.1, 126.6, 126.4, 123.4, 122.6, 118.1, 41.1, 13.7, 13.1 ppm; HRMS (+ESI) m/z calcd for [$C_{34}H_{36}N_9O$] 586.3037, found 586.3034.

*6-(1-(4-(4,5-diphenyloxazol-2-yl)phenyl)-1H-1,2,3-triazol-4-yl)-N2,N2,N4,N4-tetraethyl-1,3,5-triazine-2,4-diamine **(91)***:

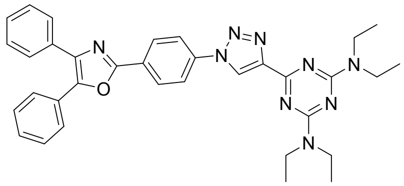

Off-white solid; yield 90%; mp = 115-117°C; R_f = 0.44 (hexane/ethyl acetate, 3:1); FT-IR: 2970, 2930, 1562, 1537, 1501, 1432, 1372, 1021 cm^{-1}; ^{1}H NMR (400 MHz, $CDCl_3$): δ 8.61 (s, 1H), 8.32 (d, *J* = 8.0 Hz, 2H), 7.96 (d, *J* = 8.0 Hz, 2H), 7.75-7.69 (m, 4H), 7.43-7.38 (m, 6H), 3.76 (s, 4H), 3.63 (s, 4H), 1.23 (s, 12H) ppm; ^{13}C NMR (125 MHz, $CDCl_3$): δ 164.4, 164.1, 158.8, 148.9, 146.1, 138.1, 137.1, 132.3, 128.8, 128.76, 128.68, 128.4, 128.1, 127.8, 127.6, 126.6, 123.0, 120.8, 41.1, 13.7, 13.1 ppm; HRMS (+ESI) m/z calcd for [$C_{34}H_{36}N_9O$] 586.3037, found 586.3036.

*6-(1-(3-(4,5-bis(4-fluorophenyl)oxazol-2-yl)phenyl)-1H-1,2,3-triazol-4-yl)-N2,N2,N4,N4-tetraethyl-1,3,5-triazine-2,4-diamine **(92)***:

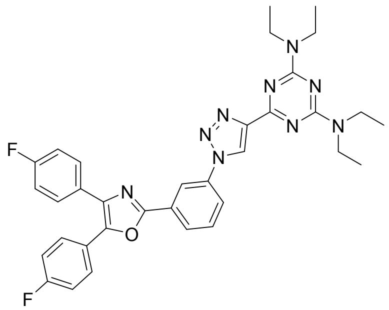

Yellow solid; yield 48%; mp = 111-113°C; R_f = 0.44 (hexane/ethyl acetate, 3:1); FT-IR: 2971, 2931, 1590, 1558, 1537, 1501, 1495, 1430, 1374, 1224, 1023, 807 cm^{-1}; ^{1}H NMR (400 MHz,

$CDCl_3$): δ 8.66 (s, 1H), 8.52 (s, 1H), 8.21 (d, *J* = 7.6 Hz, 1H), 7.99 (d, *J* = 8.0 Hz, 1H), 7.70-7.763 (m, 5H), 7.15-7.10 (m, 4H), 3.77 (s, 4H), 3.63 (s, 4H), 1.24 (t, *J* = 6.4 Hz, 12H) ppm; ^{13}C NMR (125 MHz, $CDCl_3$): δ 164.4, 164.1, 162.9 (d, *J* = 249.0 Hz), 162.8 (d, *J* = 247.0 Hz), 158.8, 148.9, 145.3, 137.7, 135.9, 130.4, 129.9 (d, *J* = 7.6 Hz), 128.6 (d, *J* = 8.6 Hz), 128.1, 126.4, 124.6, 123.4, 122.7, 118.2, 116.0 (d, *J* = 23.8 Hz), 115.8 (d, *J* = 22.8 Hz), 41.1, 13.6, 13.0 ppm; HRMS (+ESI) m/z calcd for [$C_{34}H_{33}F_2N_9O$] 622.2849, found 622.2851.

6-(1-(4-(4,5-bis(4-fluorophenyl)oxazol-2-yl)phenyl)-1H-1,2,3-triazol-4-yl)-N2,N2,N4,N4-tetraethyl-1,3,5-triazine-2,4-diamine ***(93)*****:**

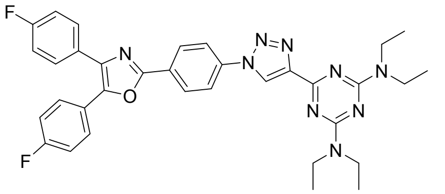

Yellow solid; yield 45%; mp = 234-235°C; R_f = 0.52 (hexane/ethyl acetate, 3:1); FT-IR: 2972, 2934, 1558, 1544, 1505, 1495, 1371, 1216, 804 cm^{-1}; ^1H NMR (400 MHz, $CDCl_3$): δ 8.61 (s, 1H), 8.30 (d, *J* = 8.4 Hz, 2H), 7.97 (d, *J* = 8.4 Hz, 2H), 7.71-7.763 (m, 4H), 7.12 (t, *J* = 8.4 Hz, 4H), 3.76 (s, 4H), 3.63 (s, 4H), 1.22 (t, *J* = 6.4 Hz, 12H) ppm; ^{13}C NMR (125 MHz, $CDCl_3$): δ 164.4, 164.1, 162.9 (d, *J* = 249.0 Hz), 162.8 (d, *J* = 248.0 Hz), 158.9, 149.0, 145.2, 138.2, 135.9, 129.8 (d, *J* = 7.6 Hz), 128.7 (d, *J* = 7.6 Hz), 128.1, 127.8, 127.4, 124.7, 122.9, 120.8, 116.0 (d, *J* = 22.8 Hz), 115.8 (d, *J* = 22.0 Hz), 41.1, 13.6, 13.0 ppm; HRMS (+ESI) m/z calcd for [$C_{34}H_{33}F_2N_9O$] 622.2849, found 622.2843.

6-(1-(3-(4,5-bis(4-chlorophenyl)oxazol-2-yl)phenyl)-1H-1,2,3-triazol-4-yl)-N2,N2,N4,N4-tetraethyl-1,3,5-triazine-2,4-diamine ***(94):***

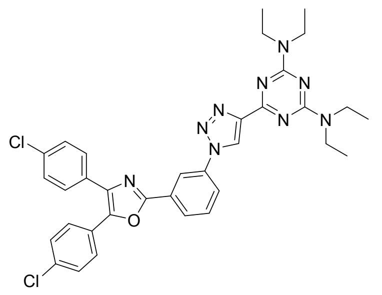

Pale yellow solid; yield 52%; mp = 188-190°C; R_f = 0.16 (hexane/ethyl acetate, 3:1); FT-IR: 2976, 2930, 1557, 1538, 1494, 1430, 1372, 807 cm^{-1}; ^{1}H NMR (400 MHz, $CDCl_3$): δ 8.65 (s, 1H), 8.51 (s, 1H), 8.20 (d, *J* = 8.0 Hz, 1H), 7.98 (d, *J* = 7.6 Hz, 1H), 7.69-7.59 (m, 5H), 7.42-7.38 (m, 4H), 3.75 (s, 4H), 3.62 (s, 4H), 1.22 (t, *J* = 6.8 Hz, 12H) ppm; ^{13}C NMR (125 MHz, $CDCl_3$): δ 166.4, 164.1, 159.1, 148.9, 145.4, 137.7, 136.4, 135.1, 134.6, 130.4, 129.4, 129.2, 129.1, 128.6, 127.9, 126.8, 126.5, 123.4, 122.8, 118.3, 41.1, 13.6, 13.0 ppm; HRMS (+ESI) m/z calcd for [$C_{34}H_{34}Cl_2N_9O$] 654.2258, found 654.2260.

6-(1-(4-(4,5-bis(4-chlorophenyl)oxazol-2-yl)phenyl)-1H-1,2,3-triazol-4-yl)-N2,N2,N4,N4-tetraethyl-1,3,5-triazine-2,4-diamine ***(95):***

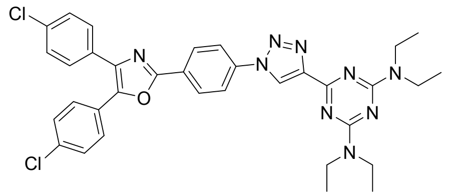

Pale yellow solid; yield 57%; mp = 242-245°C; R_f = 0.16 (hexane/ethyl acetate, 3:1); FT-IR: 2966, 2927, 1563, 1542, 1497, 1429, 1370, 804 cm^{-1}; ^{1}H NMR (400 MHz, $CDCl_3$): δ 8.60 (s, 1H), 8.30 (d, *J* = 8.4 Hz, 2H), 7.97 (d, *J* = 9.2 Hz, 2H), 7.63 (dd, *J* = 8.0 Hz, 20.8 Hz, 4H), 7.40 (d, *J* = 8.4 Hz, 4H), 3.75 (s, 4H), 3.63 (s, 4H), 1.23 (s, 12H) ppm; ^{13}C NMR (125 MHz, $CDCl_3$): δ 164.4, 164.0, 159.2, 149.0, 145.3, 138.3, 136.4, 134.9, 134.5, 130.5, 129.3, 129.2, 129.0, 127.9, 127.8, 127.2, 126.9, 122.9, 120.8, 41.1, 13.6, 13.0 ppm; HRMS (+ESI) m/z calcd for [$C_{34}H_{34}Cl_2N_9O$] 654.2258, found 654.2254.

6-(1-(3-(4,5-bis(4-methoxyphenyl)oxazol-2-yl)phenyl)-1H-1,2,3-triazol-4-yl)-N2,N2,N4,N4-tetraethyl-1,3,5-triazine-2,4-diamine ***(96):***

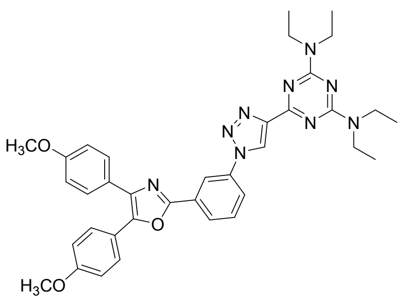

Off-white solid; yield 53%; mp = 95-97°C; R_f = 0.2 (hexane/ethyl acetate, 3:1); FT-IR: 2970, 2930, 1555, 1536, 1496, 1430, 1247, 1021, 809 cm^{-1}; ^{1}H NMR (400 MHz, $CDCl_3$): δ 8.66 (s, 1H), 8.49 (s, 1H), 8.20 (d, *J* = 8.0 Hz, 1H), 7.98 (d, *J* = 8.0 Hz, 1H), 7.88 (d, *J* = 8.4 Hz, 1H), 7.67-7.61 (m, 4H), 6.96-6.93 (m, 4H), 3.86 (s, 6H), 3.76 (s, 4H), 3.62 (s, 4H), 1.23 (s, 12H) ppm; ^{13}C NMR (125 MHz, $CDCl_3$): δ 164.4,164.2, 159.9, 159.6, 158.1, 148.9, 145.7, 137.6, 135.7, 130.3, 129.3, 129.1, 128.1, 126.3, 124.8, 123.4, 122.4, 121.3, 117.9, 114.23, 114.16, 55.3, 41.1, 13.7, 13.0 ppm; HRMS (+ESI) m/z calcd for [$C_{36}H_{39}N_9O_3$] 646.3249, found 646.3251.

6-(1-(4-(4,5-bis(4-methoxyphenyl)oxazol-2-yl)phenyl)-1H-1,2,3-triazol-4-yl)-N2,N2,N4,N4-tetraethyl-1,3,5-triazine-2,4-diamine ***(97):***

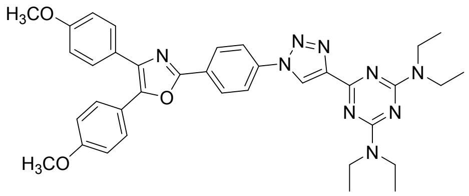

Off-white solid; yield 63%; mp = 195-197°C; R_f = 0.24 (hexane/ethyl acetate, 3:1); FT-IR: 2971, 2932, 1598, 1557, 1536, 1497, 1430, 1247, 1021, 805 cm^{-1}; ^{1}H NMR (400 MHz, $CDCl_3$): δ 8.60 (s, 1H), 8.29 (d, *J* = 8.4 Hz, 2H), 7.94 (d, *J* = 8.4 Hz, 2H), 7.63 (dd, *J* = 8.8 Hz, 14.8 Hz, 4H), 6.96-6.93 (m, 4H), 3.86 (s, 6H), 3.75 (s, 4H), 3.62 (s, 4H), 1.23 (s, 12H) ppm; ^{13}C NMR (125 MHz, $CDCl_3$): δ 164.4, 164.1, 159.9, 159.6, 158.3, 148.9, 145.6, 137.9, 135.7, 129.3, 128.2,

127.9, 127.6, 124.9, 123.0, 121.5, 120.8, 114.2, 114.1, 55.3, 41.1, 13.6, 13.0 ppm; HRMS (+ESI) m/z calcd for [$C_{36}H_{39}N_9O_3$] 646.3249, found 646.3246.

6-(1-(3-(4,5-di(furan-2-yl)oxazol-2-yl)phenyl)-1H-1,2,3-triazol-4-yl)-N2,N2,N4,N4-tetraethyl-1,3,5-triazine-2,4-diamine ***(98):***

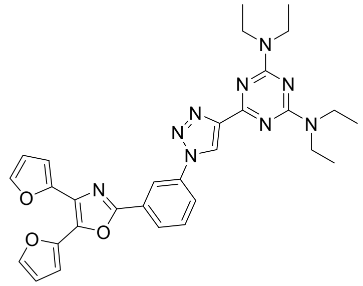

Pale brown solid; yield 52%; mp = 99-101°C; R_f = 0.2 (hexane/ethyl acetate, 3:1); FT-IR: 3117, 2971, 2931, 1557, 1537, 1495, 1430, 1022, 730 cm^{-1}; ^{1}H NMR (400 MHz, $CDCl_3$): δ 8.75 (s, 1H), 8.57 (s, 1H), 8.27 (d, *J* = 8.0 Hz, 1H), 8.07 (d, *J* = 8.4 Hz, 1H), 7.74-7.65 (m, 3H), 7.14 (dd, *J* = 4.0 Hz, 6.0 Hz, 2H), 6.64 (dq, *J* = 1.6 Hz, 2.8 Hz, 2H), 3.84 (s, 4H), 3.61 (s, 4H), 1.30 (s, 12H) ppm; ^{13}C NMR (125 MHz, $CDCl_3$): δ 164.4, 164.1, 158.6, 148.9, 146.2, 143.4, 143.1, 142.8, 137.8, 137.6, 130.4, 128.3, 128.2, 126.7, 123.4, 122.9, 118.3, 111.9, 111.6, 110.3, 109.7, 41.1, 13.6, 13.0 ppm; HRMS (+ESI) m/z calcd for [$C_{30}H_{32}N_9O_3$] 566.2623, found 566.2601.

6-(1-(4-(4,5-di(furan-2-yl)oxazol-2-yl)phenyl)-1H-1,2,3-triazol-4-yl)-N2,N2,N4,N4-tetraethyl-1,3,5-triazine-2,4-diamine ***(99):***

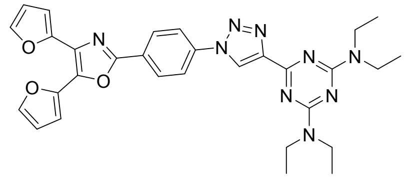

Pale brown solid; yield 46%; mp = 228-230°C; R_f = 0.27 (hexane/ethyl acetate, 3:1); FT-IR: 3113, 2967, 2929, 1541, 1499, 1429, 809, 733 cm^{-1}; ^{1}H NMR (400 MHz, $CDCl_3$): δ 8.60 (s, 1H), 8.31 (d, *J* = 8.4 Hz, 2H), 7.95 (d, *J* = 10.8 Hz, 2H), 7.60 (d, *J* = 10.8 Hz, 2H), 7.07 (dd, *J* = 2.8

Hz, 10.4 Hz, 2H), 6.60-6.57 (m, 2H), 3.75 (s, 4H), 3.62 (s, 4H), 1.23 (s, 12H) ppm; ^{13}C NMR (125 MHz, $CDCl_3$): δ 164.4, 164.1, 158.7, 148.9, 146.4, 143.3, 142.7, 138.3, 137.7, 128.4, 128.0, 126.8, 122.9, 120.6, 111.9, 111.6, 110.2, 109.6, 41.1, 13.6, 13.1 ppm; HRMS (+ESI) m/z calcd for [$C_{30}H_{32}N_9O_3$**]** 566.2623, found 566.2613.

6-(1-(3-(4,5-diphenyloxazol-2-yl)phenyl)-1H-1,2,3-triazol-4-yl)-N2,N4-bis(2-fluorophenyl)-1,3,5-triazine-2,4-diamine ***(100):***

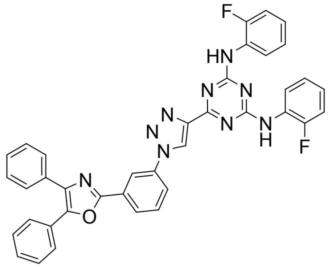

Off-white solid; yield 80 %; mp = 210-213°C (decomposed); R_f = 0.43 (hexane/ethyl acetate, 1:1); FT-IR: 3431, 3260, 1604, 1572, 1509, 1454, 1249, 749 cm^{-1}; ^{1}H NMR (400 MHz, $CDCl_3$): δ 8.80 (s, 1H), 8.55 (s, 1H), 8.35 (s br, 2H), 8.25 (d, *J* = 7.6 Hz, 2H), 7.98 (d, *J* = 7.6 Hz, 1H), 7.75-7.66 (m, 5H), 7.53 (s br, 2H), 7.45-7.36 (m, 6H), 7.16-7.04 (m, 6H) ppm; ^{13}C NMR (125 MHz, $CDCl_3$): δ 165.6, 164.5, 158.5, 153.27 (d, *J* = 243.1 Hz), 146.8, 146.4, 137.2, 137.1, 132.2, 130.5, 129.1, 128.9, 128.8, 128.7, 128.5, 128.4, 128.1, 126.7, 126.5, 124.2, 122.9, 122.2, 118.1, 115.0 (d, *J* = 19.12 Hz) ppm; HRMS (+ESI) m/z calcd for [$C_{38}H_{26}F_2N_9O$] 662.2223, found 662.2229.

6-(1-(4-(4,5-diphenyloxazol-2-yl)phenyl)-1H-1,2,3-triazol-4-yl)-N2,N4-bis(2-fluorophenyl)-1,3,5-triazine-2,4-diamine ***(101)*****:**

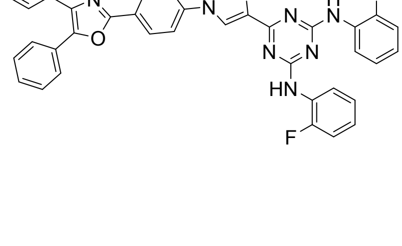

Pale yellow solid; yield 65%; mp = 132-134°C; R_f = 0.46 (hexane/ethyl acetate, 1:1); FT-IR: 3425, 3258, 1598, 1569, 1509, 1451, 1248, 1176, 1031 cm^{-1}; ^1H NMR (400 MHz, $CDCl_3$): δ 8.79 (s, 1H), 8.54 (s, 1H), 8.35 (s br, 2H), 8.23 (d, *J* = 8.0 Hz, 1H), 7.97 (d, *J* = 8.0 Hz, 1H), 7.70-7.67 (m, 5H), 7.52 (s br, 2H), 7.16-7.05 (m, 12H) ppm; ^{13}C NMR (175 MHz, $CDCl_3$): δ 165.6, 164.5, 158.5, 153.2 (d, *J* = 242.2 Hz), 146.8, 145.4, 137.2, 135.9, 130.6, 129.8, 128.8, 128.0, 126.8, 126.4, 124.6, 124.2, 122.8, 122.4, 118.2, 116.2, 116.0, 115.7, 115.13 (d, *J* = 18. 1 Hz) ppm; HRMS (+ESI) m/z calcd for [$C_{38}H_{26}F_2N_9O$] 662.2233, found 6662.2227.

6-(1-(3-(4,5-bis(4-fluorophenyl)oxazol-2-yl)phenyl)-1H-1,2,3-triazol-4-yl)-N2,N4-bis(2-fluorophenyl)-1,3,5-triazine-2,4-diamine ***(102)***:

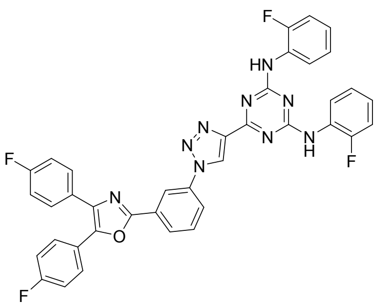

Off-white solid; yield 95%; mp = 238-240°C (decomposed); R_f = 0.43 (hexane/ethyl acetate, 1:1); FT-IR: 3426, 3246, 1625, 1571, 1510, 1453, 1221, 750 cm^{-1}; ^1H NMR (400 MHz, $CDCl_3$): δ 8.74 (s, 1H), 8.34 (d, *J* = 8.8 Hz, 3H), 7.97 (d, *J* = 8.4 Hz, 2H), 7.72 (dd, *J* = 7.2 Hz, 16.0 Hz, 4H), 7.52 (s, 2H), 7.45-7.38 (m, 6H), 7.17-7.08 (m, 6H) ppm; ^{13}C NMR (125 MHz, $CDCl_3$): δ 165.6, 164.5, 158.7, 153.3 (d, *J* = 243.12 Hz), 146.8, 146.3, 137.6, 137.2, 132.2, 129.2, 128.9, 128.8, 128.70, 128.4, 128.1, 127.9, 126.7, 126.4, 124.2, 123.8, 122.8, 120.8, 115.1 (d, *J* = 19.12 Hz) ppm; HRMS (+ESI) m/z calcd for [$C_{38}H_{24}F_4N_9O$] 698.2034, found 698.2041.

6-(1-(4-(4,5-bis(4-fluorophenyl)oxazol-2-yl)phenyl)-1H-1,2,3-triazol-4-yl)-N2,N4-bis(2-fluorophenyl)-1,3,5-triazine-2,4-diamine ***(103)*****:**

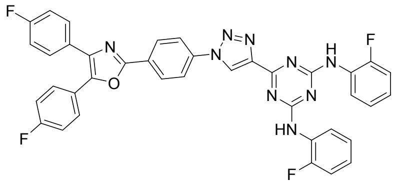

Off-white solid; yield 77%; mp = 239-241°C; R_f = 0.42 (hexane/ethyl acetate, 1:1); FT-IR: 3411, 3066, 1626, 1567, 1508, 1452, 1410, 1226, 741 cm^{-1}; ^1H NMR (400 MHz, $CDCl_3$): δ 8.77 (s, 1H), 8.31 (d, *J* = 8.4 Hz, 3H), 7.97 (d, *J* = 8.0 Hz, 2H), 7.70-7.63 (m, 4H), 7.52 (s, 2H), 7.17-7.08 (m, 11H) ppm; ^{13}C NMR (125 MHz, $CDCl_3$): δ 165.5, 164.4, 162.9 (d, *J* = 248.8 Hz), 162.80 (d, *J* = 248.0 Hz), 158.6, 153.2 (d, *J* = 243.1 Hz), 146.8, 145.2, 137.6, 136.0, 129.8 (d, *J* = 7.6 Hz), 128.6 (d, *J* = 7.6 Hz), 128.1, 127.8, 126.4, 124.6, 124.1, 123.7, 122.8, 120.7, 116.05 (d, *J* = 21.8 Hz), 115.8 (d, *J* = 21.8 Hz), 115.1 (d, *J* = 19.00 Hz) ppm; HRMS (+ESI) m/z calcd for [$C_{38}H_{24}F_4N_9O$] 698.2034, found 698.2038.

6-(1-(3-(4,5-bis(4-chlorophenyl)oxazol-2-yl)phenyl)-1H-1,2,3-triazol-4-yl)-N2,N4-bis(2-fluorophenyl)-1,3,5-triazine-2,4-diamine ***(104)*****:**

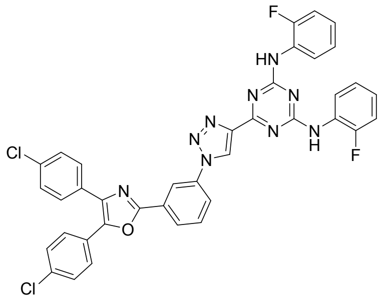

Off-white solid; yield 70%; mp = 252-254°C; R_f = 0.42 (hexane/ethyl acetate, 1:1); FT-IR: 3451, 3249, 1621, 1570, 1509, 1453, 1249, 1090, 751 cm^{-1}; ^1H NMR (400 MHz, $CDCl_3$): δ 8.79 (s, 1H), 8.54 (s, 1H), 8.35 (s br, 2H), 8.23 (d, *J* = 8.0 Hz, 1H), 7.98 (d, *J* = 8.0 Hz, 1H), 7.72-7.60 (m, 5H), 7.52 (s, 2H), 7.40 (d, *J* = 8.4 Hz, 4H), 7.17-7.06 (m, 6H) ppm; ^{13}C NMR (125 MHz, $CDCl_3$): δ 165.6, 164.5, 158.8, 153.2 (d, *J* = 242.1 Hz), 146.8, 145.5, 137.2, 136.4, 135.1, 134.6, 130.6, 130.3, 129.3, 129.29, 129.23, 129.0, 128.8, 128.0, 126.8, 126.7, 126.3, 124.1, 122.8,

122.5, 118.2, 115.1 (d, J = 19.1 Hz) ppm; HRMS (+ESI) m/z calcd for [$C_{38}H_{24}Cl_2F_2N_9O$] 730.1443, found 730.1452.

6-(1-(4-(4,5-bis(4-chlorophenyl)oxazol-2-yl)phenyl)-1H-1,2,3-triazol-4-yl)-N2,N4-bis(2-fluorophenyl)-1,3,5-triazine-2,4-diamine ***(105):***

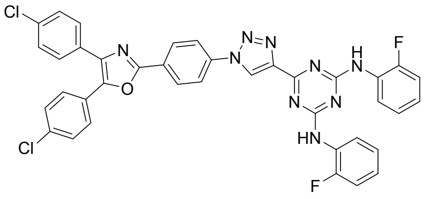

Off-white solid; yield 69%; mp = 232-234°C; R_f = 0.48 (hexane/ethyl acetate, 1:1); FT-IR: 3421, 1625, 1576, 1509, 1455, 1248, 1090, 745 cm^{-1}; ^{1}H NMR (400 MHz, $CDCl_3$): δ 8.76 (s, 1H), 8.34 (d, J = 8.4 Hz, 3H), 7.99 (d, J = 8.0 Hz, 2H), 7.66 (d, J = 8.0 Hz, 2H), 7.61 (d, J = 8.0 Hz, 2H), 7.50 (s, 2 H), 7.41 (d, J = 8.4 Hz, 4H), 7.18-7.09 (m, 6H) ppm; ^{13}C NMR (125 MHz, $CDCl_3$): δ 165.4, 164.4, 158.9, 153.2 (d, J = 242.2 Hz), 146.7, 145.3, 137.7, 136.4, 135.0, 134.5, 130.4, 129.3, 129.1 (d, J = 22.0 Hz), 127.9, 127.6, 126.8, 126.4, 124.1, 123.7, 122.9, 120.6, 115.1 (d, J = 19.1 Hz) ppm; HRMS (+ESI) m/z calcd for [$C_{38}H_{24}Cl_2F_2N_9O$] 730.1443, found 730.1453.

6-(1-(3-(4,5-bis(4-methoxyphenyl)oxazol-2-yl)phenyl)-1H-1,2,3-triazol-4-yl)-N2,N4-bis(2-fluorophenyl)-1,3,5-triazine-2,4-diamine ***(106)*****:**

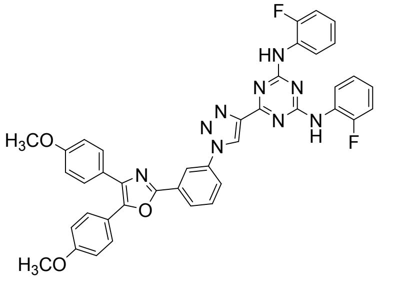

Pale yellow solid; yield 66%; mp = 140-142°C; R_f = 0.33 (hexane/ethyl acetate, 1:1); FT-IR: 3429, 3260, 3061, 1602, 1569, 1507, 1451, 1250, 1182 cm^{-1}; ^{1}H NMR (400 MHz, $CDCl_3$): δ

8.79 (s, 1H), 8.50 (s, 1H), 8.33 (s br, 2H), 8.20 (d, *J* = 7.2 Hz, 1H), 7.94 (d, *J* = 7.2 Hz, 1H), 7.66-7.56 (m, 6H), 7.15-7.04 (m, 6H), 6.93 (dd, *J* = 3.0 Hz, 8.0 Hz, 4H), 3.85 (s, 6H) ppm; ^{13}C NMR (125 MHz, $CDCl_3$): δ 165.7, 164.5, 160.0, 159.6, 157.9, 153.3 (d, *J* = 243.2 Hz), 146.8, 145.8, 137.1, 135.7, 130.4, 129.3, 128.2, 126.6, 126.5, 124.7, 124.2, 122.8, 122.0, 121.3, 118.0, 115.1 (d, *J* = 19.12 Hz), 114.24, 114.10, 55.3 ppm; HRMS (+ESI) m/z for calcd [$C_{40}H_{30}F_2N_9O_3$] 722.2434, found 722.2447.

6-(1-(4-(4,5-bis(4-methoxyphenyl)oxazol-2-yl)phenyl)-1H-1,2,3-triazol-4-yl)-N2,N4-bis(2-fluorophenyl)-1,3,5-triazine-2,4-diamine ***(107)*****:**

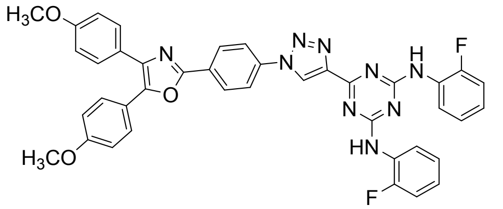

Light-yellow solid: yield 75%; mp = 144-146°C; R_f = 0.36 (hexane/ethyl acetate, 1:1); FT-IR: 3421, 2962, 1599, 1567, 1506, 1451, 1247, 1175, 1030, 745 cm^{-1}; ^1H NMR (400 MHz, $CDCl_3$): δ 8.70 (s, 1H), 8.28 (d, *J* = 8.0 Hz, 3H), 7.92 (d, *J* = 8.0 Hz, 2H), 7.61 (dd, *J* = 8.8 Hz, 15.2 Hz, 4H), 7.50 (d, *J* = 3.2 Hz, 2H), 7.17-7.06 (m, 6H), 6.93 (dd, *J* = 4.0 Hz, 8.4 Hz, 4H), 3.84 (s, 6H) ppm; ^{13}C NMR (125 MHz, $CDCl_3$)**:** δ 165.6, 164.5, 159.9, 159.6, 158.0, 153.2 (d, *J* = 247.0 Hz), 146.8, 145.7, 137.4, 135.8, 129.3, 128.2, 128.1, 127.7, 126.4, 124.8, 124.1, 123.8, 122.8, 121.4, 120.7, 115.1 (d, *J* = 19.1 Hz), 114.2, 114.1, 55.3 ppm; HRMS (+ESI) m/z calcd for [$C_{40}H_{30}F_2N_9O_3$] 722.2434, found 722.2442.

6-(1-(3-(4,5-di(furan-2-yl)oxazol-2-yl)phenyl)-1H-1,2,3-triazol-4-yl)-N2,N4-bis(2-fluorophenyl)-1,3,5-triazine-2,4-diamine ***(108)*****:**

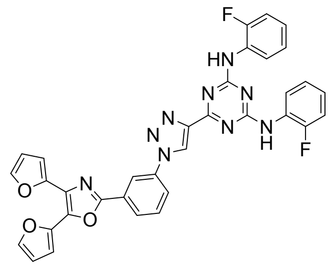

Off-white solid; yield 69%; mp = 216-218°C; R_f = 0.35 (hexane/ethyl acetate, 1:1); FT-IR: 3423, 1597, 1566, 1537, 1510, 1450, 1366, 1182, 808 cm^{-1}; ^{1}H NMR (400 MHz, $CDCl_3$): δ 8.79 (s, 1H), 8.52 (s, 1H), 8.35 (s br, 2H), 8.24 (d, *J* = 7.2 Hz, 1H), 7.98 (d, *J* = 6.8 Hz, 1H), 7.67 (t, *J* = 8.0 Hz, 1H), 7.60 (d, *J* = 13.2 Hz, 2H), 7.52 (s br, 2H), 7.16-7.06 (m, 8H), 6.58 (ddd, *J* = 1.6 Hz, 3.2 Hz, 13.6 Hz, 2H) ppm; ^{13}C NMR (125 MHz, $CDCl_3$): δ 165.7, 164.5, 158.4, 153.2 (d, *J* = 243.1 Hz), 146.8, 146.3, 143.4, 143.1, 142.8, 137.9, 137.2, 130.5, 128.5, 128.4, 127.1, 126.5, 126.4, 124.2, 122.7, 118.4, 115.1 (d, *J* = 19.0 Hz), 111.9, 111.7, 110.4, 109.7 ppm; HRMS (+ESI) m/z calcd for [$C_{34}H_{22}F_2N_9O_3$] 642.1808, found 642.1814.

6-(1-(4-(4,5-di(furan-2-yl)oxazol-2-yl)phenyl)-1H-1,2,3-triazol-4-yl)-N2,N4-bis(2-fluorophenyl)-1,3,5-triazine-2,4-diamine ***(109)*****:**

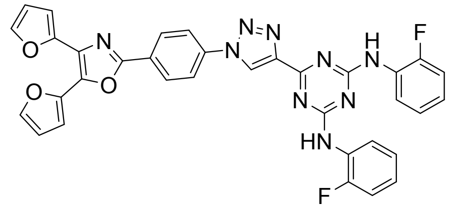

Light-yellow solid; yield 83%; mp = 128-130°C; R_f = 0.33 (hexane/ethyl acetate, 1:1); FT-IR: 3417, 3117, 1624, 1567, 1507, 1451, 1252, 1184, 988, 736 cm^{-1}; ^{1}H NMR (400 MHz, $CDCl_3$): δ 8.78 (s, 1H), 8.33 (d, *J* = 8.0 Hz, 3H), 7.96 (d, *J* = 8.4 Hz, 2H), 7.60 (dd, *J* = 1.2 Hz, 11.6 Hz, 2H), 7.53 (s br, 2H), 7.19-7.06 (m, 8H), 6.58 (ddd, *J* = 2.0 Hz, 3.6 Hz, 14.4 Hz, 2H) ppm; ^{13}C NMR (125 MHz, $CDCl_3$): δ 164.5, 158.6, 153.2 (d, *J* = 244.1 Hz), 146.3, 143.4, 143.2, 142.8,

137.8, 128.5, 128.2, 127.4, 126.3, 124.2, 122.9, 120.8, 115.1 (d, J = 19.1 Hz), 111.9, 111.7, 110.3, 109.7 ppm; HRMS (+ESI) m/z calcd for [$C_{34}H_{22}F_2N_9O_3$] 642.1808, found 642.1814.

6-(1-(2-(4,5-diphenyloxazol-2-yl)phenyl)-1H-1,2,3-triazol-4-yl)-N2,N4-bis(4-fluorophenyl)-1,3,5-triazine-2,4-diamine ***(110)*****:**

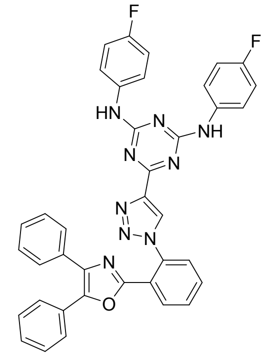

Off-white solid; yield 30%; mp = 145-146°C; R_f = 0.77 (methanol/chloroform, 1:9); FT-IR: 3284, 3070, 1697, 1615, 1558, 1495, 1211, 1041 cm^{-1}; ^{1}H NMR (400 MHz, $CDCl_3$): δ 8.61 (s, 1H), 8.40 (d, J = 7.6 Hz, 1H), 7.96 (d, J = 7.6 Hz, 1H), 7.66-7.49 (m, 10H), 7.28 (m, 2H), 7.03-6.98 (m, 4H), 7.03-6.98 (m, 4H) ppm; ^{13}C NMR (125 MHz, THF, 45°C): δ 172.2, 164.9, 164.7, 158.7 (d, J = 239.4 Hz), 146.4, 135.9, 133.4, 132.9, 132.6, 132.2, 130.8, 130.2, 129.6, 129.4, 129.0, 128.6, 128.3, 128.2, 128.1, 127.9, 127.7, 126.6, 126.2, 122.1, 114.6 (d, J = 21.8 Hz) ppm; HRMS (+ESI) m/z calcd for [$C_{38}H_{25}F_2N_9O$+H] 662.2223, found 662.2227.

6-(1-(3-(4,5-diphenyloxazol-2-yl)phenyl)-1H-1,2,3-triazol-4-yl)-N2,N4-bis(4-fluorophenyl)-1,3,5-triazine-2,4-diamine ***(111)*****:**

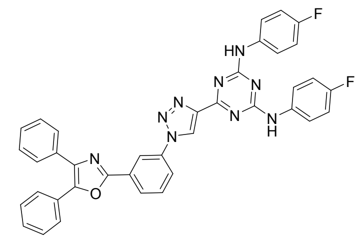

Off-white solid; yield 82%; mp = 240-242°C; R_f = 0.25 (hexane/ethyl acetate, 3:1); FT-IR: 3274, 1326, 1574, 1529, 1497, 1411, 1213, 1155, 832 cm^{-1}; ^{1}H NMR (400 MHz, $CDCl_3$): δ 8.76 (s, 1H), 8.53 (s, 1H), 8.26 (d, *J* = 7.6 Hz, 1H), 7.98 (d, *J* = 8.0 Hz, 1H), 7.75-7.67 (m, 5H), 7.55 (s, 4H), 7.43-7.34 (m, 6H), 7.06 (t, *J* = 8.0 Hz, 4H) ppm; ^{13}C NMR (125 MHz, $CDCl_3$): δ 165.4, 146.5, 159.4 (d, *J* = 242.2 Hz), 146.9, 146.4, 137.2, 137.1, 133.9, 132.1, 130.5, 129.1, 129.0, 128.7 (d, *J* = 9.5 Hz), 128.5, 128.1, 126.8, 126.7, 123.9, 122.8, 122.3, 118.1, 115.5 (d, *J* = 22.0 Hz) ppm; HRMS (+ESI) m/z calcd for [$C_{38}H_{25}F_2N_9O$+H] 662.2223, found 662.2226.

6-(1-(4-(4,5-diphenyloxazol-2-yl)phenyl)-1H-1,2,3-triazol-4-yl)-N2,N4-bis(4-fluorophenyl)-1,3,5-triazine-2,4-diamine ***(112)*****:**

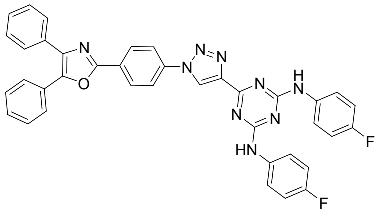

Off-white solid; yield 47%; mp = 237-239°C; R_f = 0.78 (methanol/chloroform, 1:9); FT-IR: 3386, 3080, 1694, 1565, 1495, 1434, 1411, 1212, 989, 831cm^{-1}; ^{1}H NMR (400 MHz, $CDCl_3$): δ 8.68 (s, 1H), 8.34 (d, *J* = 8.0 Hz, 2H), 7.95 (d, *J* = 8.8 Hz, 2H), 7.70 (dd, *J* = 6.4 Hz, 15.6 Hz, 4H), 7.54 (s, 4H), 7.43-7.40 (m, 6H), 7.07-7.04 (m, 4H) ppm; ^{13}C NMR (125 MHz, $CDCl_3$): δ 165.7, 164.9, 158.84, 158.82 (d, *J* = 239.3 Hz), 146.2, 138.3, 137.0, 135.8, 133.4 (d, *J* = 22.0 Hz), 132.6, 130.8, 129.2, 129.0, 128.8, 128.7, 128.5, 128.2, 128.0, 127.8, 127.6, 127.5, 126.7, 122.3 (d, *J* = 6.6 Hz), 120.5, 114.7 (d, *J* = 22.0 Hz) ppm; HRMS (+ESI) m/z calcd for [$C_{38}H_{25}F_2N_9O$+H] 662.2223, found 662.2227.

6-(1-(2-(4,5-bis(4-fluorophenyl)oxazol-2-yl)phenyl)-1H-1,2,3-triazol-4-yl)-N2,N4-bis(4-fluorophenyl)-1,3,5-triazine-2,4-diamine ***(113)*****:**

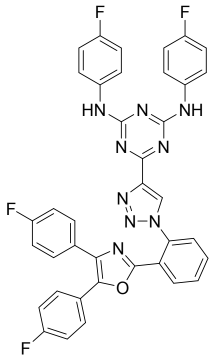

Pale green solid; yield 77%; mp = 163-165°C; R_f = 0.14 (hexane/ethyl acetate, 3:1); FT-IR: 3421, 1612, 1584, 1499, 1444, 1414, 1225, 1154 cm^{-1}; ^{1}H NMR (400 MHz, CDCl$_3$): δ 8.57 (s, 1H), 8.37 (d, J = 7.6 Hz, 1H), 7.73-7.65 (m, 2H), 7.62 (d, J = 8.0 Hz, 1H), 7.58-7.53 (m, 6H), 7.35-7.31 (m, 3H), 7.02-6.93 (m, 7H) ppm; ^{13}C NMR (125 MHz, THF, 45°C): δ 165.9, 164.8, 162.8 (d, J = 124.8 Hz), 162.6 (d, J = 246.0 Hz), 157.7, 156.9, 146.4, 145.2, 135.9, 135.2, 134.9, 131.0, 130.4, 129.6 (d, J = 7.6 Hz), 129.5, 128.8 (d, J = 7.6 Hz), 128.2, 124.5, 123.9, 122.1, 115.5 (d, J = 21.8 Hz), 115.2 (d, J = 21.0 Hz), 114.7 (d, J = 22.8 Hz) ppm; HRMS (+ESI) m/z calcd for [$C_{38}H_{23}F_4N_9O$+H] 698.2034, found 698.2035.

6-(1-(3-(4,5-bis(4-fluorophenyl)oxazol-2-yl)phenyl)-1H-1,2,3-triazol-4-yl)-N2,N4-bis(4-fluorophenyl)-1,3,5-triazine-2,4-diamine ***(114)*****:**

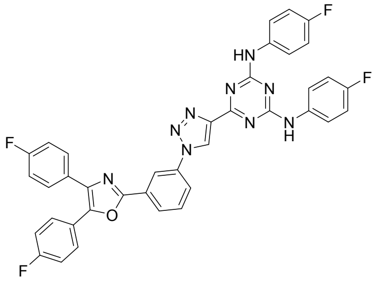

Off-white solid; yield 87%; mp = 262-264°C; R_f = 0.17 (methanol/chloroform, 1:9); FT-IR: 3419, 1616, 1568, 1494, 1409, 1211, 1156, 829 cm^{-1}; ^{1}H NMR (400 MHz, CDCl$_3$): δ 8.73 (s, 1H), 8.51 (s, 1H), 8.22 (d, J = 8.0 Hz, 1H), 7.95 (d, J = 8.4 Hz, 1H), 7.70-7.63 (m, 5H), 7.54 (m, 4H), 7.35 (s, 1H), 7.12 (t, J = 8.4 Hz, 4H), 7.04 (t, J = 8.4 Hz, 4H) ppm; ^{13}C NMR (125 MHz,

$CDCl_3$): δ 164.1, 159.5 (d, J = 235 Hz), 145.5, 136.1, 133.9, 130.5, 129.8 (d, J = 7.6 Hz), 129.1, 128.8 (d, J = 7.6 Hz), 128.0, 126.8, 124.7, 122.9, 122.4, 118.3, 116.0 (d, J = 22.0 Hz), 115.8 (d, J = 21.8 Hz), 115.5 (d, J = 23.0 Hz) ppm; HRMS (+ESI) m/z calcd for [$C_{38}H_{23}F_4N_9O$+H] 698.2034, found 698.2030.

6-(1-(4-(4,5-bis(4-fluorophenyl)oxazol-2-yl)phenyl)-1H-1,2,3-triazol-4-yl)-N2,N4-bis(4-fluorophenyl)-1,3,5-triazine-2,4-diamine ***(115)*****:**

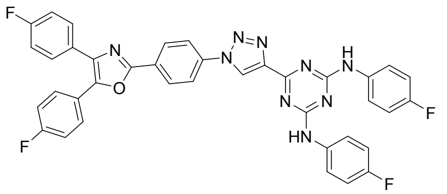

Off-white solid; yield 26%; mp = 287-289°C; R_f = 0.12 (hexane/ethyl acetate, 3:1); FT-IR: 3415, 3061, 2920, 2853, 1565, 1499, 1405, 1208, 1157 cm^{-1}; ^1H NMR (400 MHz, $CDCl_3$): δ 9.98 (s, 1H), 9.27 (s, 1H), 8.29 (d, J = 8.4 Hz, 2H), 8.21 (d, J = 8.4 Hz, 2H), 7.81 (s br, 2H), 7.73-7.65 (m, 5H), 7.35-7.26 (m, 4H), 7.15 (t, J = 8.4 Hz, 4H) ppm; ^{13}C NMR (125 MHz, THF, 45°C): δ 164.9, 163.1 (d, J = 247.0 Hz), 162.8 (d, J = 246.0 Hz), 159.8, 158.9, 157.8, 145.2, 138.5, 136.0, 135.8, 129.6 (d, J = 8.6 Hz), 129.0 (d, J = 8.6 Hz), 128.6, 127.5, 127.4, 125.2, 123.8, 122.3, 120.5, 115.7 (d, J = 21.8 Hz), 115.2 (d, J = 21.0 Hz), 114.7 (d, J = 22.8 Hz) ppm; HRMS (+ESI) m/z calcd for [$C_{38}H_{23}F_4N_9O$+H] 698.2034, found 698.2037.

6-(1-(2-(4,5-bis(4-chlorophenyl)oxazol-2-yl)phenyl)-1H-1,2,3-triazol-4-yl)-N2,N4-bis(4-fluorophenyl)-1,3,5-triazine-2,4-diamine ***(116)*****:**

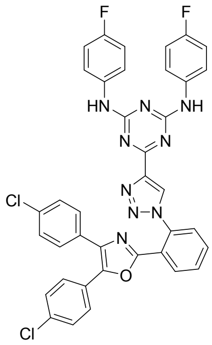

Off-white solid; yield 83%; mp = 272-274°C; R_f = 0.12 (hexane/ethyl acetate, 3:1); FT-IR: 3273, 3132, 1621, 1588, 1495, 1410, 1212, 828 cm^{-1}; ^1H NMR (400 MHz, $CDCl_3$): δ 8.75 (s, 1H), 8.54 (s, 1H), 8.25 (d, J = 7.6 Hz, 1H), 7.99 (d, J = 8.4 Hz, 1H), 7.73-7.61 (m, 5H), 7.55 (s, 4H), 7.43-7.40 (m, 4H), 7.07 (d, J = 7.4 Hz, 4H) ppm; ^{13}C NMR (125 MHz, THF, 45°C): δ 165.6, 164.8, 158.7 (d, J = 240.0 Hz), 159.1, 146.4, (d, J = 243.0 Hz), 137.8, 136.3, 135.8, 134.8, 134.1, 130.8, 130.3, 129.1 (d, J = 21.0 Hz), 128.7, 128.6, 128.3, 127.2, 126.0, 123.9, 122.2, 122.19, 122.0, 117.8, 114.7 (d, J = 22.8 Hz) ppm; HRMS (+ESI)m/z calcd for [$C_{38}H_{23}ClF_{22}N_9O$+H] 730.1443, found 730.1448.

6-(1-(3-(4,5-bis(4-chlorophenyl)oxazol-2-yl)phenyl)-1H-1,2,3-triazol-4-yl)-N2,N4-bis(4-fluorophenyl)-1,3,5-triazine-2,4-diamine ***(117)*****:**

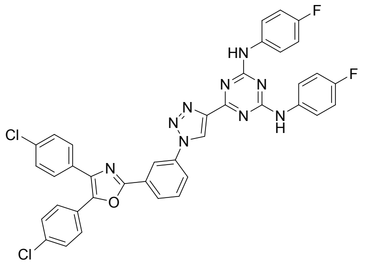

Off-white solid; yield 55%; mp = 255-257°C; R_f = 0.15 (hexane/ethyl acetate, 3:1); FT-IR: 3263, 3127, 1622, 1589, 1496, 1411, 1214, 1091, 829 cm^{-1}; ^1H NMR (400 MHz, $CDCl_3$): δ 8.74 (s, 1H), 8.52 (s, 1H), 8.23 (d, J = 8.0 Hz, 1H), 7.97 (d, J = 8.4 Hz, 1H), 7.72-7.60 (m, 5H), 7.54 (s, 4H), 7.42-7.39 (m, 4H), 7.08-7.04 (m, 6H) ppm; ^{13}C NMR (125 MHz, THF, 45°C): δ 165.6,

164.8, 158.7 (d, J = 239.0 Hz), 159.2, 147.4, 145.5, 137.8, 136.3, 135.8, 134.8, 134.1, 130.8, 130.3, 129.1 (d, J = 21.0 Hz), 128.7, 128.6, 128.3, 127.2, 126.0, 123.9, 122.26, 122.20, 122.0, 117.8, 114.7 (d, J = 22.8 Hz) ppm.; HRMS (+ESI) m/z calcd for [$C_{38}H_{23}ClF_{22}N_9O$+H] 730.1443, found 730.1445.

6-(1-(4-(4,5-bis(4-chlorophenyl)oxazol-2-yl)phenyl)-1H-1,2,3-triazol-4-yl)-N2,N4-bis(4-fluorophenyl)-1,3,5-triazine-2,4-diamine ***(118)*****:**

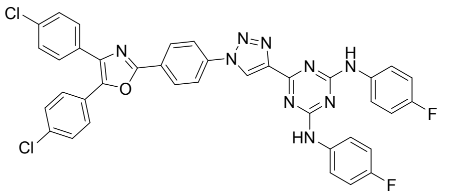

Off-white solid; yield 51%; mp = > 290°C; R_f = 0.74 (methanol/chloroform, 1:9); FT-IR: 3216, 3127, 1588, 1497, 1412, 1211, 1090 cm^{-1}; ^1H NMR (400 MHz, $CDCl_3$): δ 9.99 (s br, 2H), 9.29 (s br, 1H), 8.31 (d, J = 8.4 Hz, 2H), 8.23 (d, J = 8.4 Hz, 2H), 7.81 (s br, 2H), 7.71-7.66 (m, 5H), 7.57-7.52 (m, 4H), 7.16 (t, J = 8.8 Hz, 4H) ppm; ^{13}C NMR (125 MHz, THF, 45°C): δ 165.6, 164.9, 159.3, 158.8 (d, J = 240.2 Hz), 147.4, 145.3, 138.6, 136.4, 135.8, 134.8, 134.1, 130.8 (d, J = 21.8 Hz), 129.2, 129.0, 128.6, 128.3, 127.6, 127.3, 127.2, 123.7, 122.3, 120.5, 114.7 (d, J = 21.8 Hz) ppm; HRMS (+ESI) m/z calcd for [$C_{38}H_{23}ClF_{22}N_9O$+H] 730.1443, found 730.1450.

6-(1-(2-(4,5-bis(4-methoxyphenyl)oxazol-2-yl)phenyl)-1H-1,2,3-triazol-4-yl)-N2,N4-bis(4-fluorophenyl)-1,3,5-triazine-2,4-diamine ***(119)*****:**

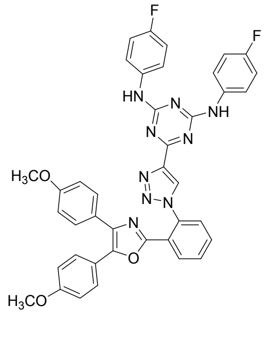

Pale yellow solid; yield 43%; mp = 157-159°C; R_f = 0.20 (hexane/ethyl acetate, 3:1); FT-IR: 3336, 3136, 1692, 1602, 1574, 1495, 1410, 1161 cm^{-1}; ^1H NMR (400 MHz, $CDCl_3$): δ 8.59 (s, 1H), 8.39 (d, J = 7.2 Hz, 1H), 7.72-7.69 (m, 1H), 7.64-7.62 (m, 2H), 7.53-7.50 (m, 6H), 7.31 (d, J = 8.8 Hz, 2H), 7.03 (m, 4H), 6.82 (d, J = 8.0 Hz, 2H), 6.76 (d, J = 8.4 Hz, 2H), 3.76 (s, 3H), 3.70 (s, 3H) ppm; ^{13}C NMR (125 MHz, THF, 45°C): δ 166.1, 164.9, 160.2, 159.8, 158.7 (d, J = 239.4 Hz), 156.0, 146.3, 145.6, 135.9, 135.1, 134.7, 130.4, 130.2, 129.3, 128.7, 128.1, 128.0, 124.6, 124.3, 122.1, 120.9, 114.7 (d, J = 21.8 Hz), 114.3, 113.8, 113.6, 54.4 ppm; HRMS (+ESI) m/z calcd for [$C_{40}H_{29}F_2N_9O_3$+H] 722.2434, found 722.2438.

6-(1-(3-(4,5-bis(4-methoxyphenyl)oxazol-2-yl)phenyl)-1H-1,2,3-triazol-4-yl)-N2,N4-bis(4-fluorophenyl)-1,3,5-triazine-2,4-diamine ***(120)***:

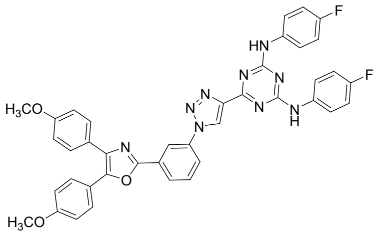

Off-white solid; yield 77 %; mp = 168-170°C; R_f = 0.16 (hexane/ethyl acetate, 3:1); FT-IR: 3353, 3125, 1692, 1573, 1495, 1409, 1164, 1025 cm^{-1}; ^1H NMR (400 MHz, $CDCl_3$): δ 8.68 (s, 1H), 8.41 (s, 1H), 8.13 (d, J = 7.6 Hz, 1H), 7.86 (dd, J = 8.4 Hz, 24.4 Hz 2H), 7.62-7.56 (m, 6H), 7.48 (s, 4H), 6.98 (d, J = 8.0 Hz, 4H), 6.90 (d, J = 8.0 Hz, 4H), 3.83 (s, 6H) ppm; ^{13}C NMR (125 MHz, THF, 45°C): δ 165.6, 164.8, 160.5, 159.9, 158.8 (d, J = 241.3 Hz), 158.0, 147.3, 145.7, 137.8, 135.8, 130.2, 129.3, 128.8, 128.3, 125.8, 124.9, 123.9, 122.3, 121.5, 117.6, 114.6 (d, J = 22.0 Hz), 114.5, 113.7, 54.6, 54.5 ppm; HRMS (+ESI) m/z calcd for [$C_{40}H_{29}F_2N_9O_3$+H] 722.2434, found 722.2435.

6-(1-(4-(4,5-bis(4-methoxyphenyl)oxazol-2-yl)phenyl)-1H-1,2,3-triazol-4-yl)-N2,N4-bis(4-fluorophenyl)-1,3,5-triazine-2,4-diamine ***(121)***:

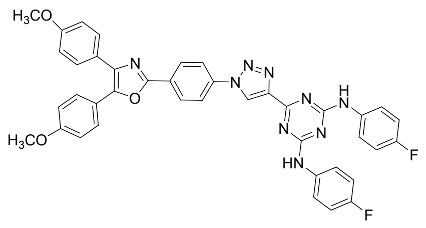

Off-white solid; yield 67%; mp = 160-162°C; R_f = 0.13 (hexane/ethyl acetate, 3:1); FT-IR: 3379, 2845, 1689, 1600, 1495, 1409, 1242, 1163, 1023 cm^{-1}; ^{1}H NMR (400 MHz, $CDCl_3$): δ 8.73 (s, 1H), 8.48 (s, 1H), 8.25-8.20 (m, 1H), 8.05 (t, *J* = 7.6 Hz, 1H), 7.93-7.87 (m, 2H), 7.65-7.59 (m, 4H), 7.52 (s, 4H), 7.03 (s, 4H), 6.94 (d, *J* = 8.4 Hz, 4H) ppm; ^{13}C NMR (125 MHz, THF, 45°C): δ 165.6, 164.8, 160.4, 159.9, 158.8 (d, *J* = 239.4 Hz), 158.1, 146.0, 145.5, 138.1, 135.8, 128.8, 128.2, 127.9, 127.3, 124.9, 123.7, 122.2, 121.5, 120.4, 114.7 (d, *J* = 21.8 Hz), 114.0, 113.7, 54.6, 54.4 ppm; HRMS (+ESI) m/z calcd for [$C_{40}H_{29}F_2N_9O_3$+H] 722.2434, found 722.2437.

6-(1-(2-(4,5-di(furan-2-yl)oxazol-2-yl)phenyl)-1H-1,2,3-triazol-4-yl)-N2,N4-bis(4-fluorophenyl)-1,3,5-triazine-2,4-diamine ***(122)*****:**

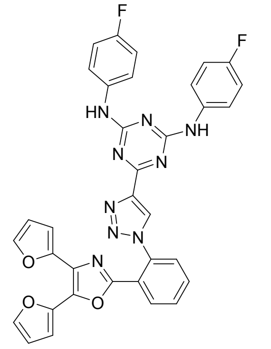

Light-yellow solid; yield 49%; mp = 147-148°C; R_f = 0.11 (hexane/ethyl acetate, 3:1); FT-IR: 3270, 3128, 1627, 1572, 1487, 1411, 1215, 824 cm^{-1}; ^{1}H NMR (400 MHz, $CDCl_3$): δ 8.55 (s, 1H), 8.39 (d, *J* = 7.6 Hz, 1H), 7.73-7.65 (m, 2H), 7.60 (d, *J* = 7.6 Hz, 1H), 7.54 (s br, 4H), 7.49 (s, 1H), 7.43 (s, 1H), 7.02-7.00 (m, 5H), 6.84 (dd, *J* = 3.2 Hz, 10.0 Hz, 2H), 6.44 (s br, 2H) ppm; ^{13}C NMR (125 MHz, $CDCl_3$, 45°C): δ 165.9, 164.6, 159.3 (d, *J* = 243.1 Hz), 156.1, 146.3, 145.8 143.3, 142.8, 142.6, 137.9, 134.4, 134.0, 131.2, 130.7, 130.0, 129.1, 128.2, 127.9, 123.3, 122.7,

115.4 (d, *J* = 22.8 Hz), 111.8, 111.5, 110.5, 109.8 ppm; HRMS (+ESI) m/z calcd for $[C_{34}H_{21}F_2N_9O_3+H]$ 642.1808, found 642.1807.

6-(1-(3-(4,5-di(furan-2-yl)oxazol-2-yl)phenyl)-1H-1,2,3-triazol-4-yl)-N2,N4-bis(4-fluorophenyl)-1,3,5-triazine-2,4-diamine ***(123)*****:**

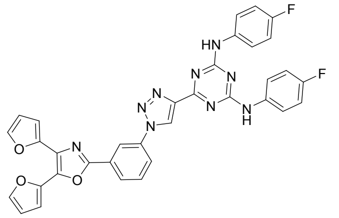

Grey solid; yield 69%; mp = 210-212°C (decomposed); R_f = 0.11 (hexane/ethyl acetate, 3:1); FT-IR: 3265, 3130, 1634, 1581, 1483, 1417, 1219, cm^{-1}; ^{1}H NMR (400 MHz, $CDCl_3$): δ 8.75 (s, 1H), 8.50 (s, 1H), 8.24 (d, *J* = 7.6 Hz, 1H), 7.98 (d, *J* = 6.4 Hz,1H), 7.67 (t, *J* = 8.0 Hz, 1H), 7.61-7.55 (m, 6H), 7.35 (s br, 2H), 7.11 (d, *J* = 2.8 Hz, 1H), 7.06-7.05 (m, 5H), 6.59 (d, *J* = 12.8 Hz, 2H) ppm; ^{13}C NMR (125 MHz, $CDCl_3$, 45°C): δ 164.5, 159.4 (d, *J* = 248.0 Hz), 146.3, 143.4, 143.1, 142.7, 137.9, 137.3, 133.9, 130.5, 128.5, 127.1, 122.8, 122.6, 118.4, 115.5 (d, *J* = 21.8 Hz), 111.9, 111.6, 110.5, 109.7 ppm; HRMS (+ESI) m/z calcd for $[C_{34}H_{21}F_2N_9O_3+H]$ 642.1808, found 642.1809.

6-(1-(4-(4,5-di(furan-2-yl)oxazol-2-yl)phenyl)-1H-1,2,3-triazol-4-yl)-N2,N4-bis(4-fluorophenyl)-1,3,5-triazine-2,4-diamine ***(124)*****:**

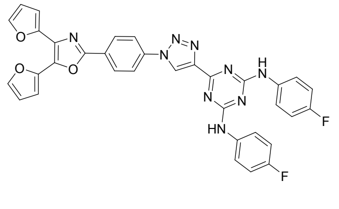

Light green solid; yield 68%; mp = 210-212°C; R_f = 0.15 (hexane/ethyl acetate, 3:1); FT-IR: 3268, 3126, 1621, 1569, 1495, 1408, 1211, 830 cm^{-1}; ^{1}H NMR (400 MHz, $CDCl_3$): δ 8.66 (s, 1H), 8.32 (d, *J* = 8.4 Hz, 2H), 7.93 (d, *J* = 8.4 Hz, 2H), 7.59 (d, *J* = 11.2 Hz, 2H), 7.53 (s, 4H),

7.33 (s, 2H), 7.09-7.02 (m, 6H), 6.58 (d, *J* = 12.8 Hz, 2H) ppm; ^{13}C NMR (125 MHz, $CDCl_3$, 45°C): δ 165.4, 164.5, 160.4, 159.4 (d, *J* = 243.2 Hz), 158.5, 146.4, 143.3, 143.2, 142.7, 137.9, 133.9, 128.6, 128.2, 127.5, 123.5, 122.8, 120.7, 115.5 (d, *J* = 22.8 Hz), 111.8, 111.6, 110.3, 109.7 ppm; HRMS (+ESI) m/z calcd for $[C_{34}H_{21}F_2N_9O_3+H]$ 642.1808, found 642.18010.

^{1}H **NMR: 2-(chloromethyl)-4,5-bis(4-fluorophenyl)oxazole**

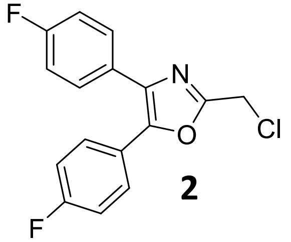

2

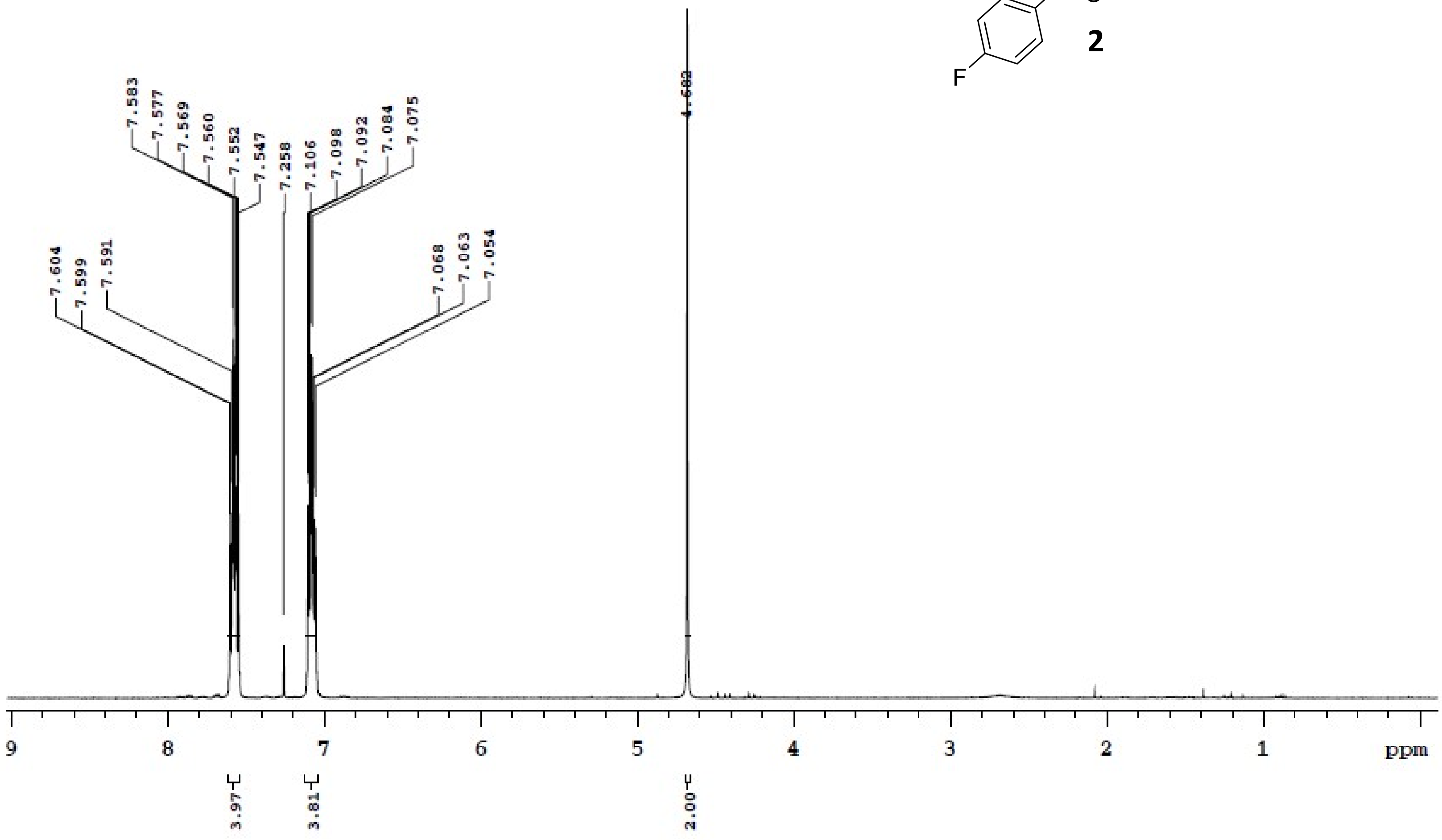

^{13}C NMR: 2-(chloromethyl)-4,5-bis(4-fluorophenyl)oxazole

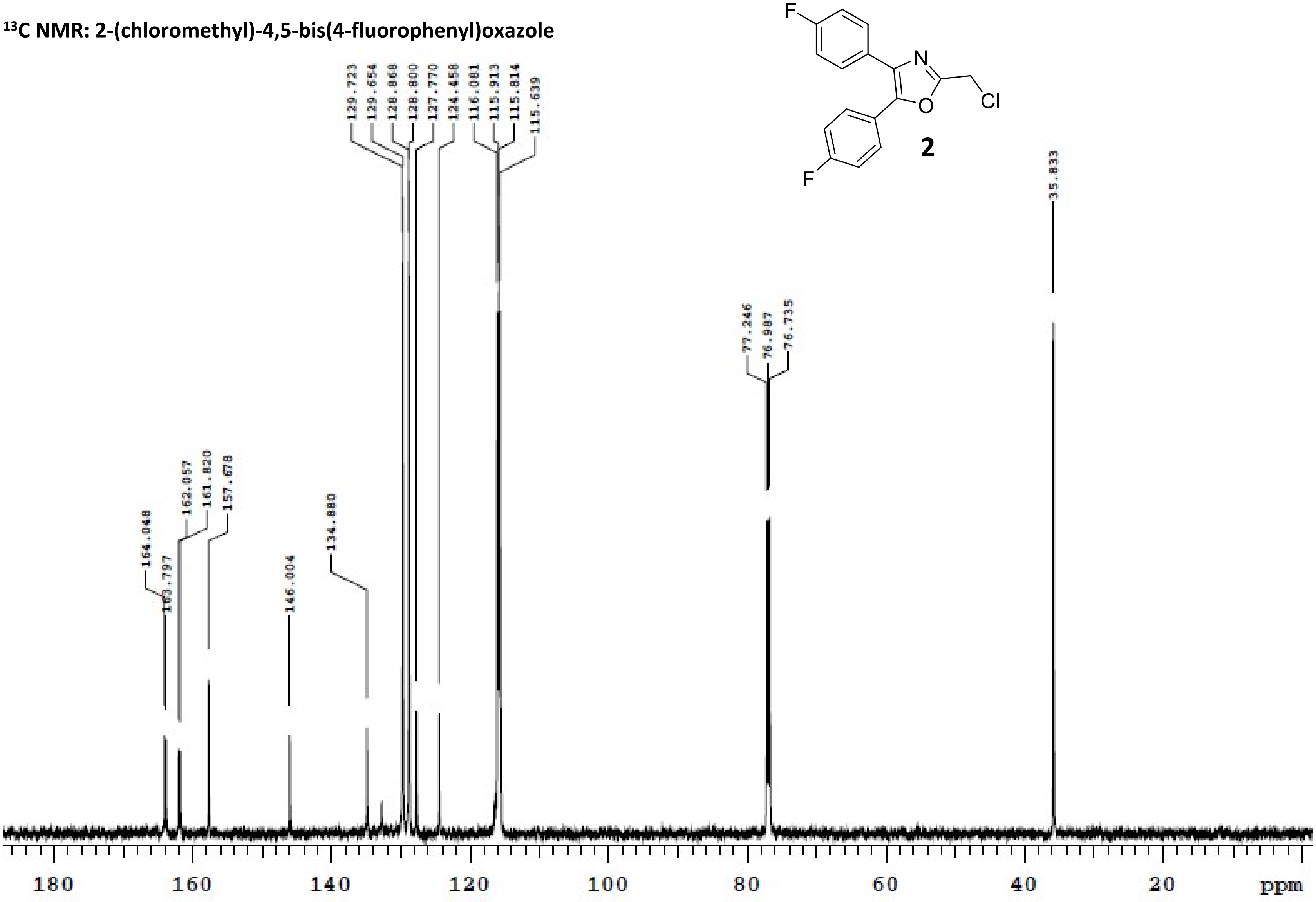

^{1}H NMR: 2-(chloromethyl)-4,5-bis(4-chlorophenyl)oxazole

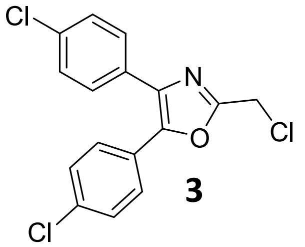

3

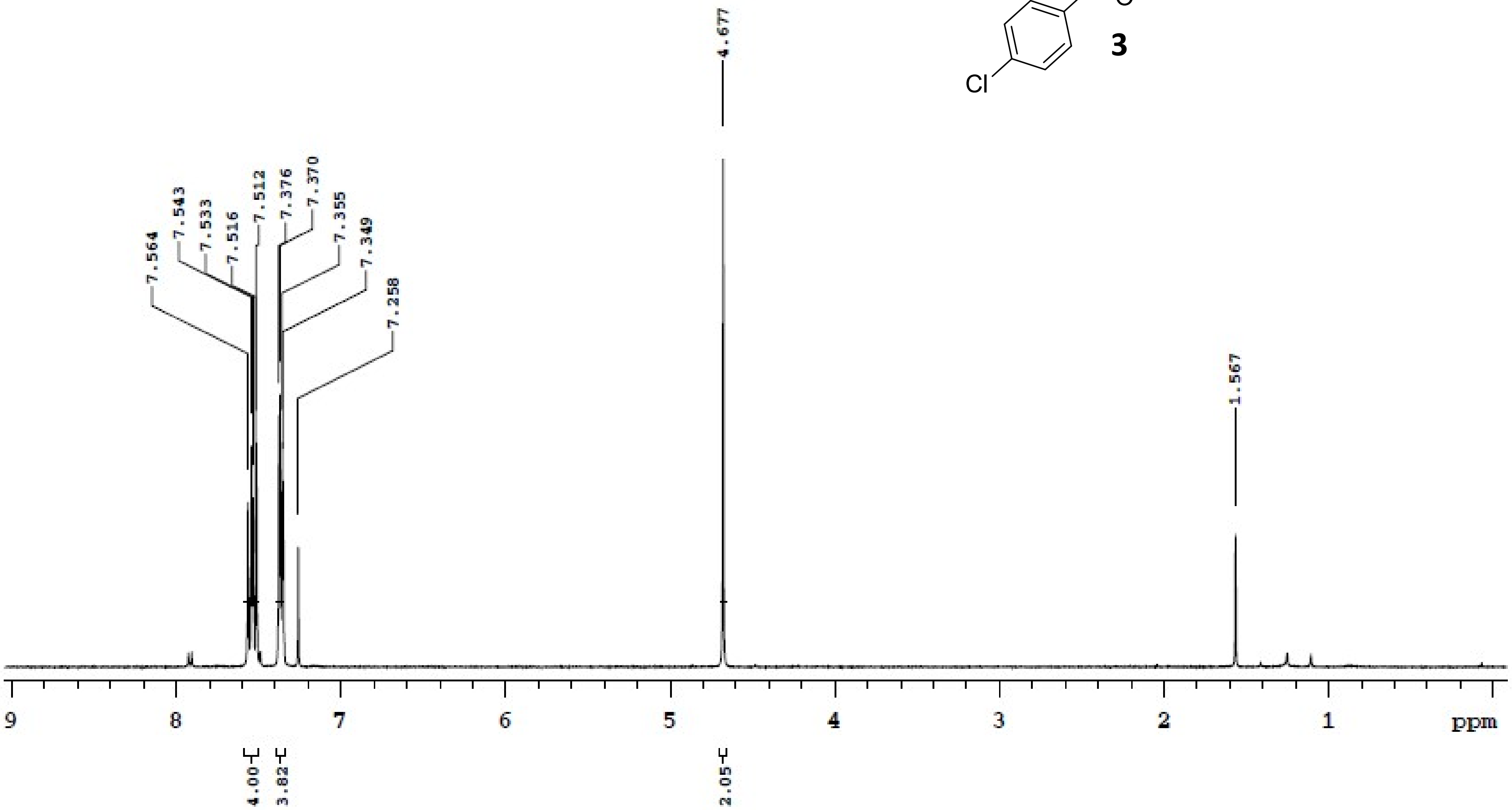

^{13}C NMR: 2-(chloromethyl)-4,5-bis(4-chlorophenyl)oxazole

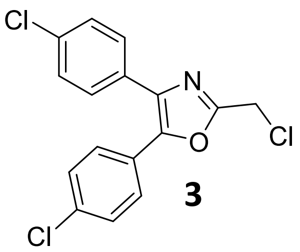

3

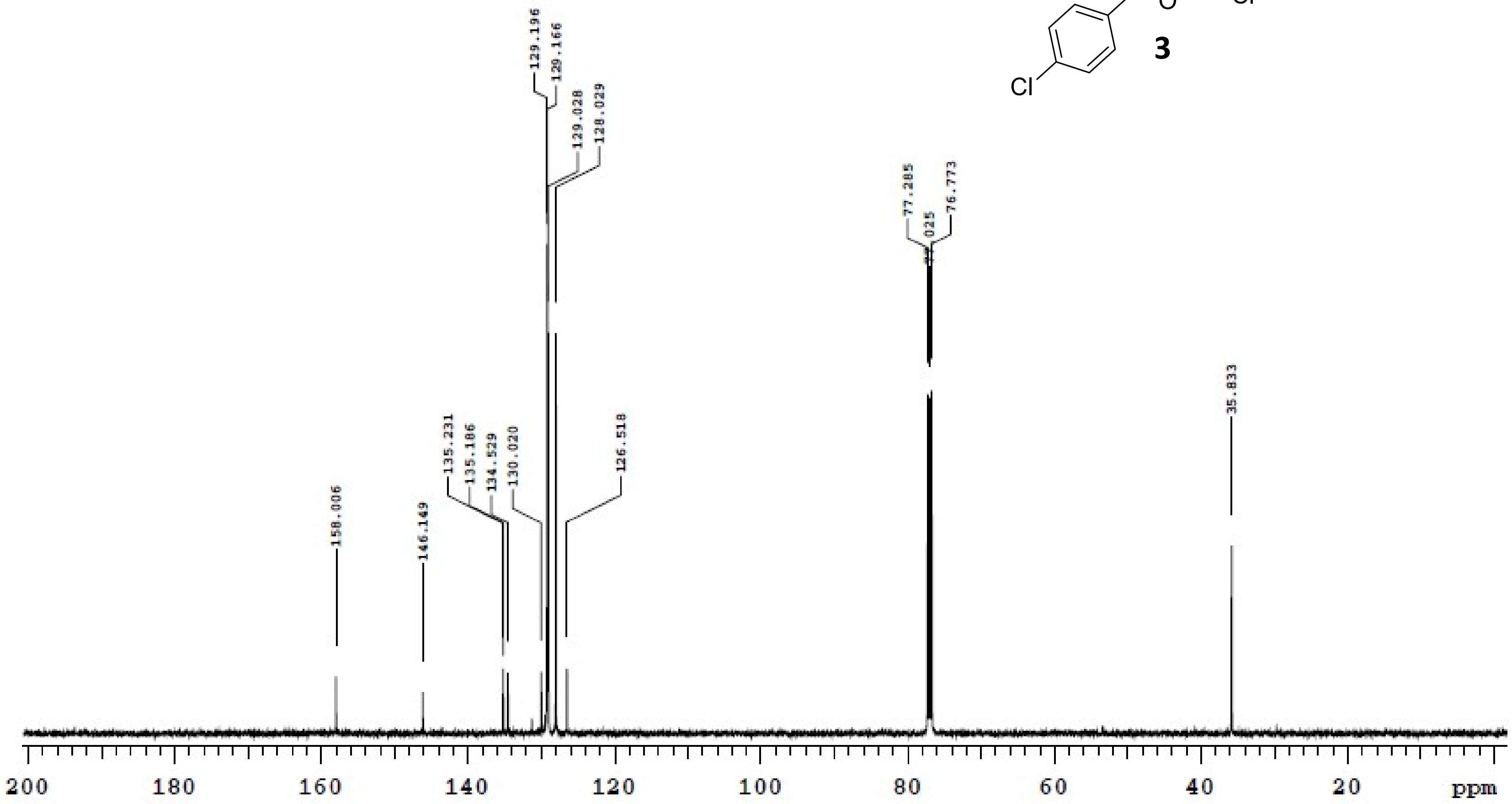

^{1}H NMR: 4-(((4,5-diphenyloxazol-2-yl)methyl)thio)aniline

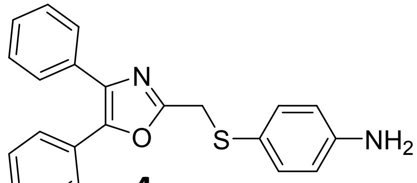

4

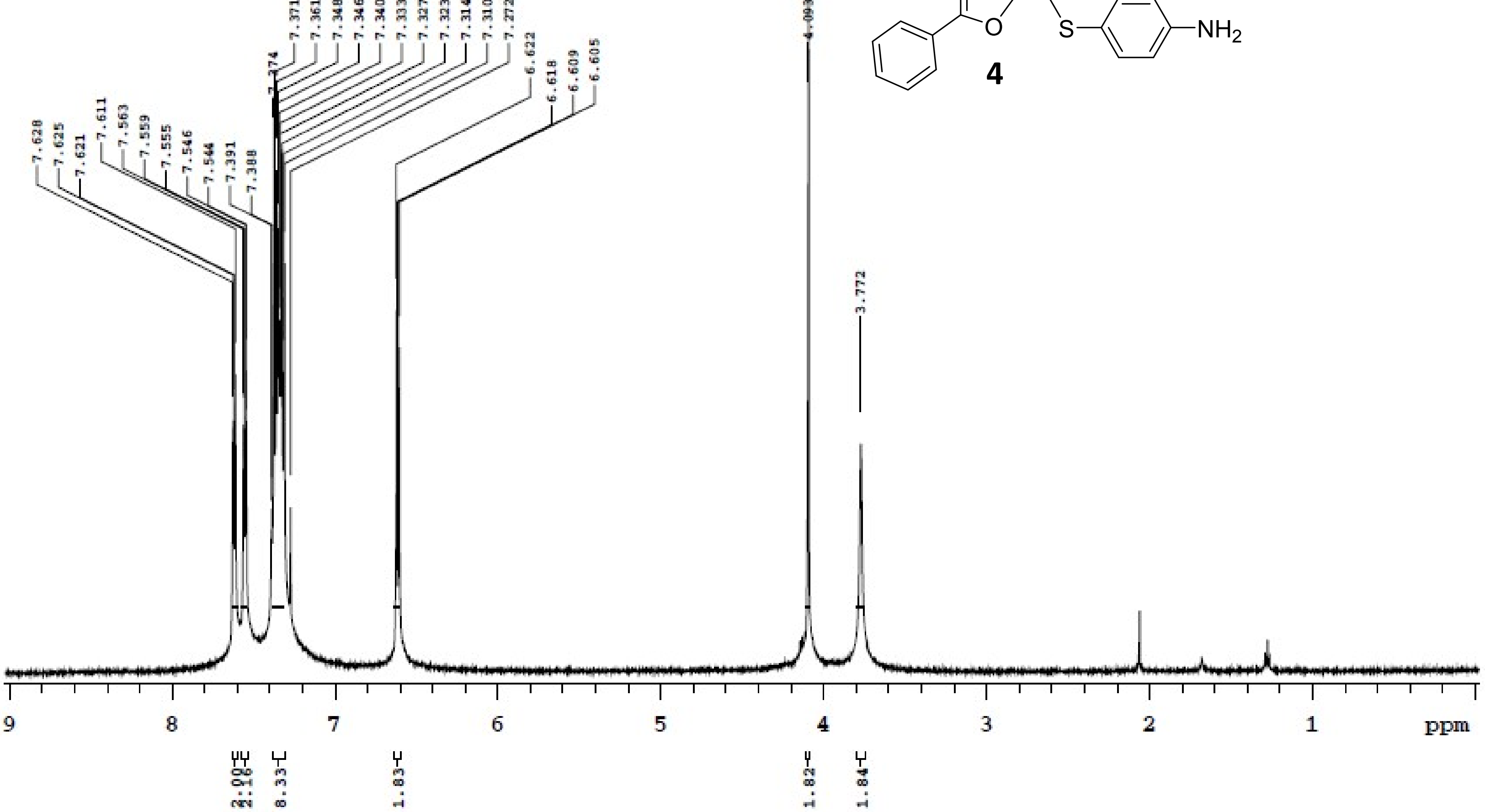

^{13}C NMR: 4-(((4,5-diphenyloxazol-2-yl)methyl)thio)aniline

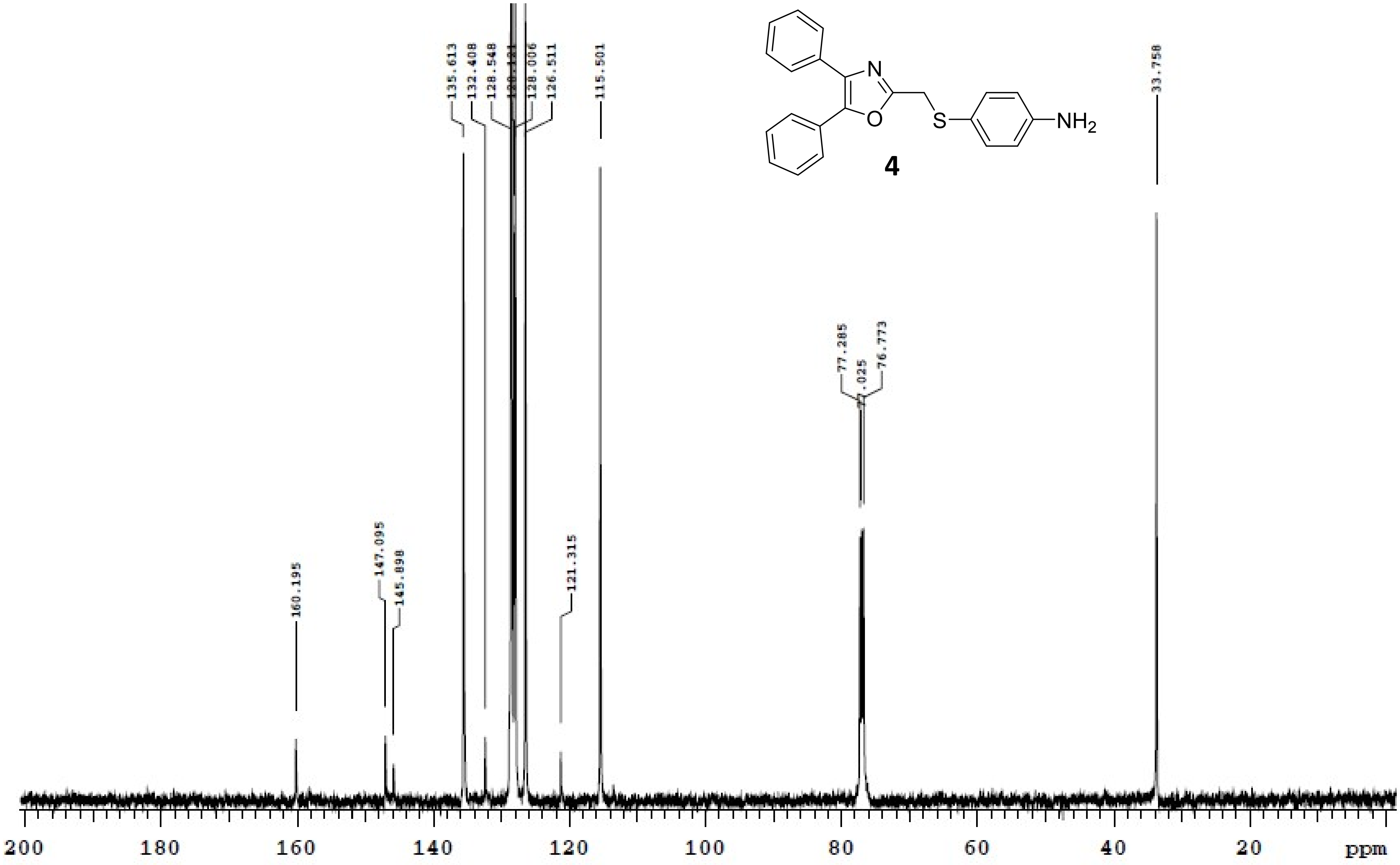

^{1}H NMR: 4-(((4,5-bis(4-fluorophenyl)oxazol-2-yl)methyl)thio)aniline

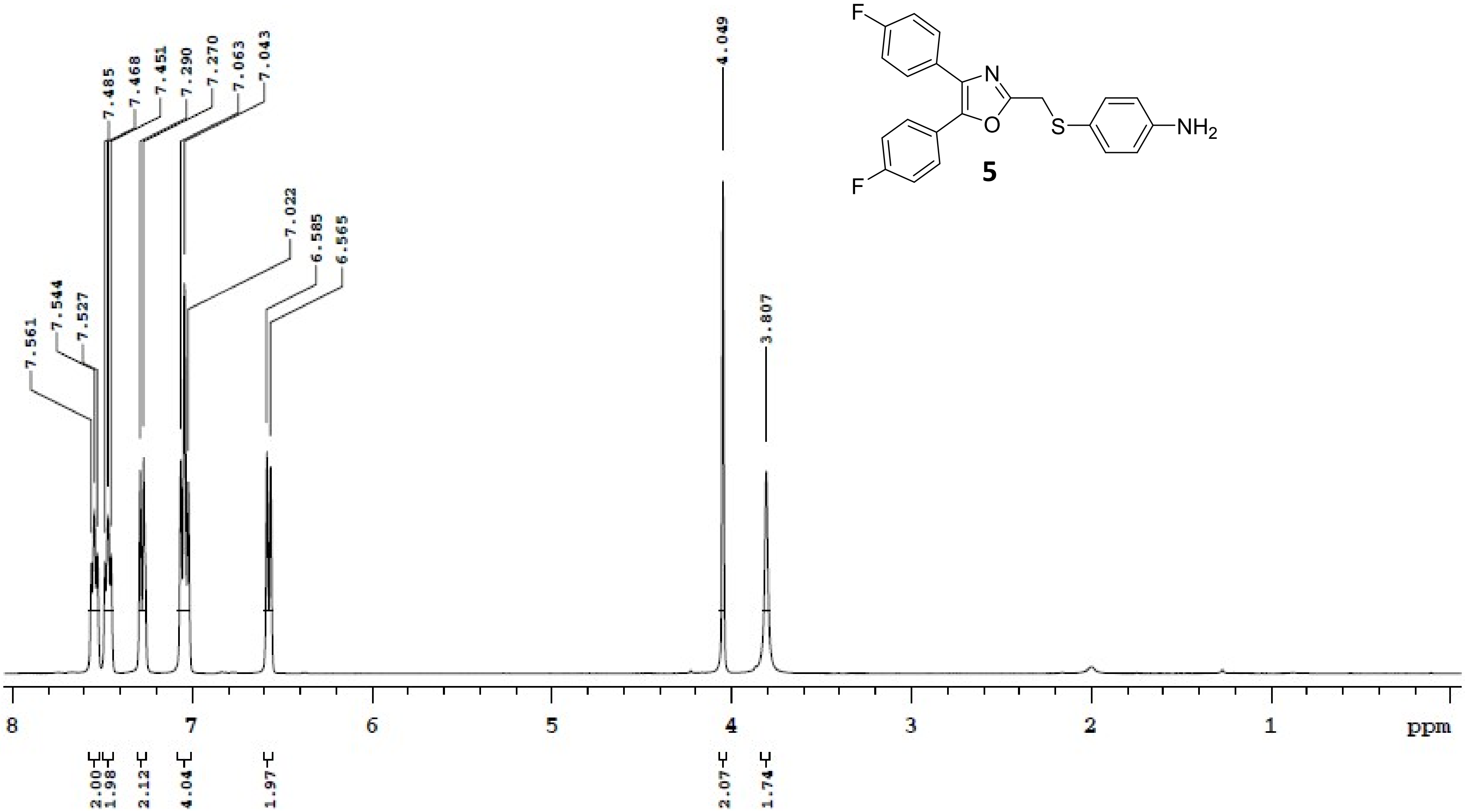

^{13}C NMR: 4-(((4,5-bis(4-fluorophenyl)oxazol-2-yl)methyl)thio)aniline

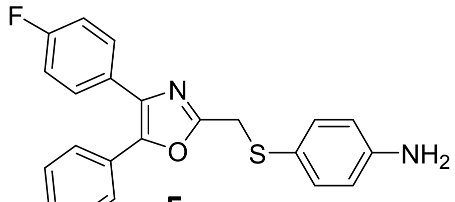

5

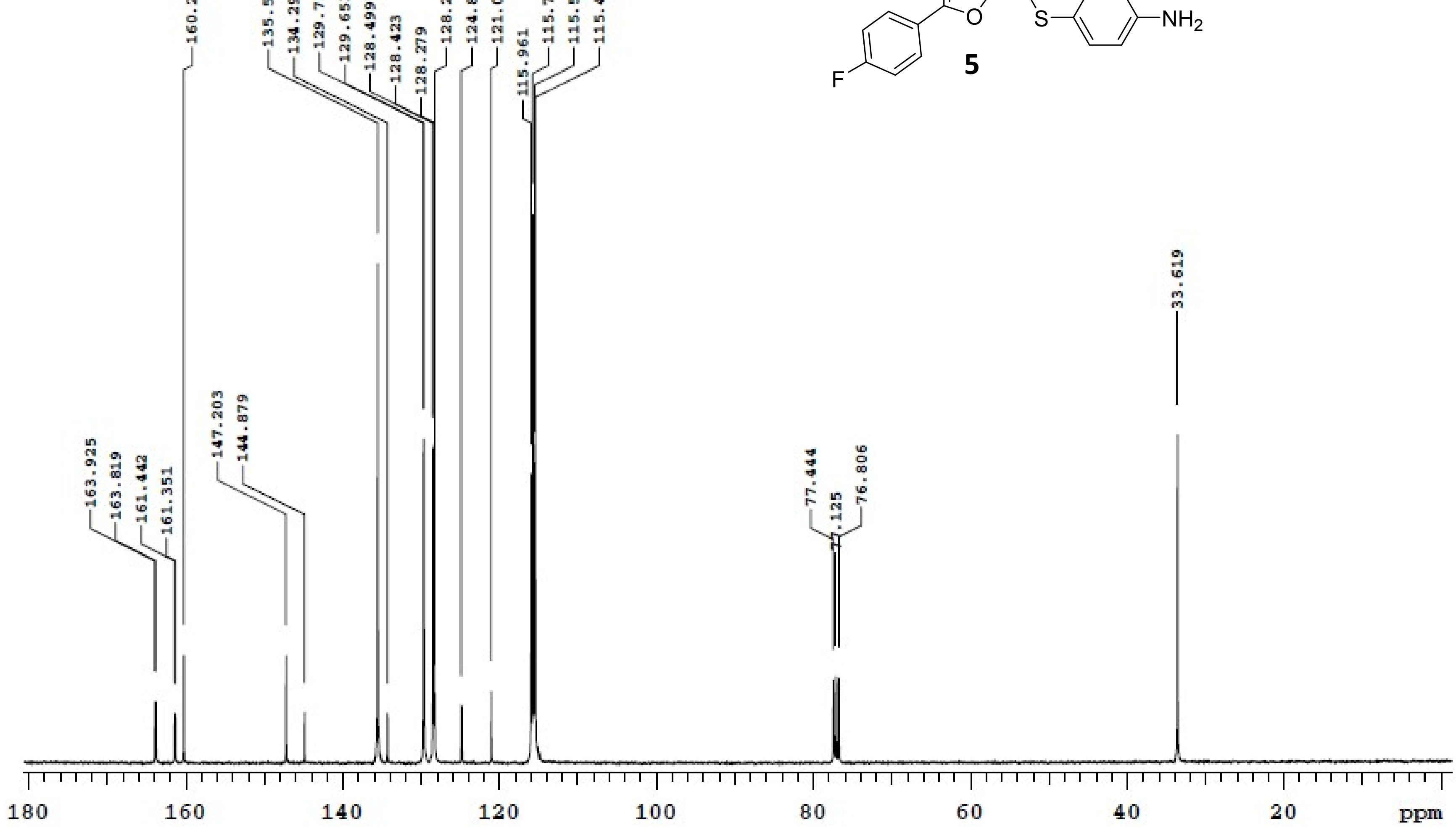

^{1}H NMR:4-(((4,5-bis(4-chlorophenyl)oxazol-2-yl)methyl)thio)aniline

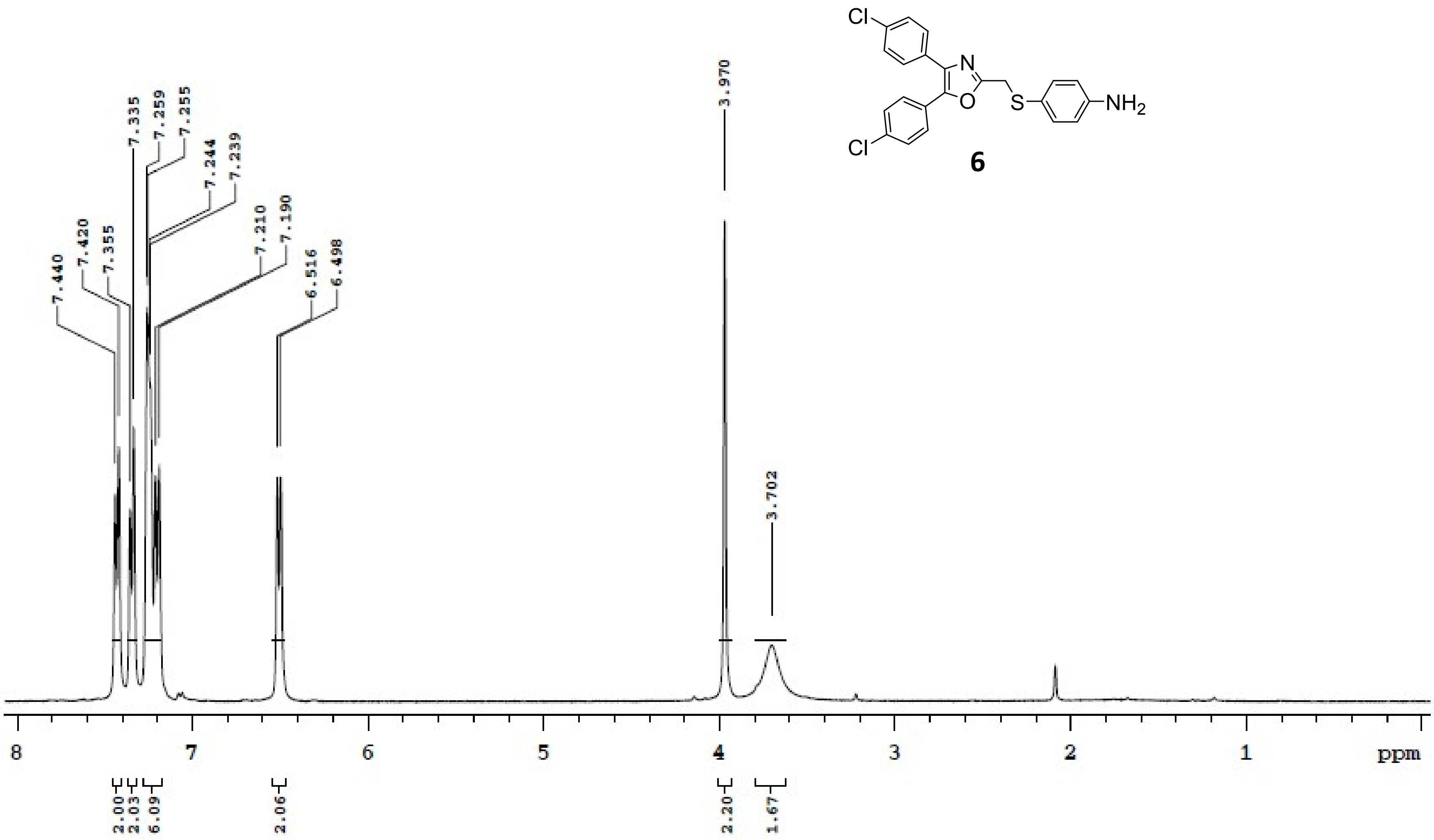

^{13}C NMR: 4-(((4,5-bis(4-chlorophenyl)oxazol-2-yl)methyl)thio)aniline

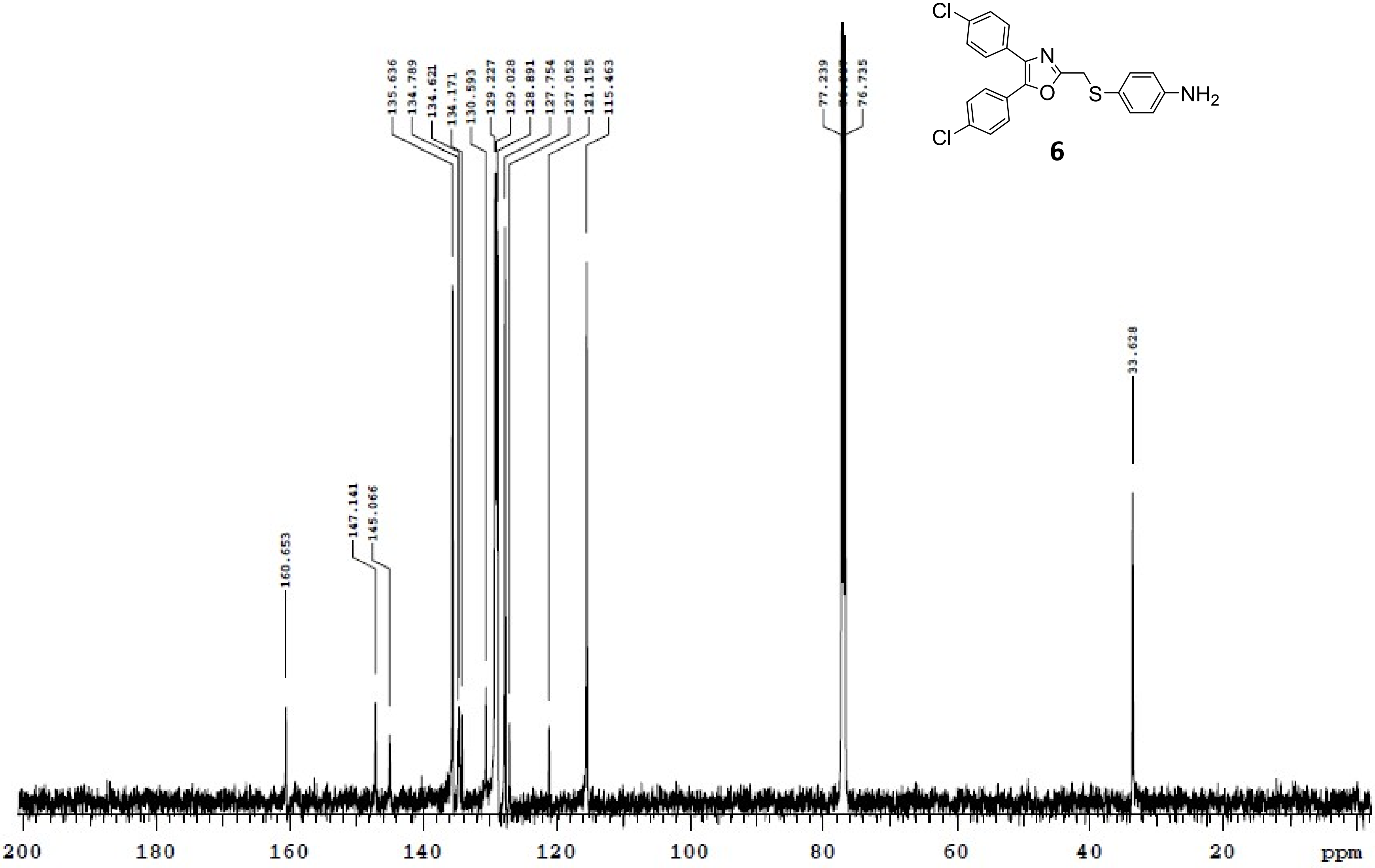

^{1}H NMR: 2-(((4-azidophenyl)thio)methyl)-4,5-diphenyloxazole

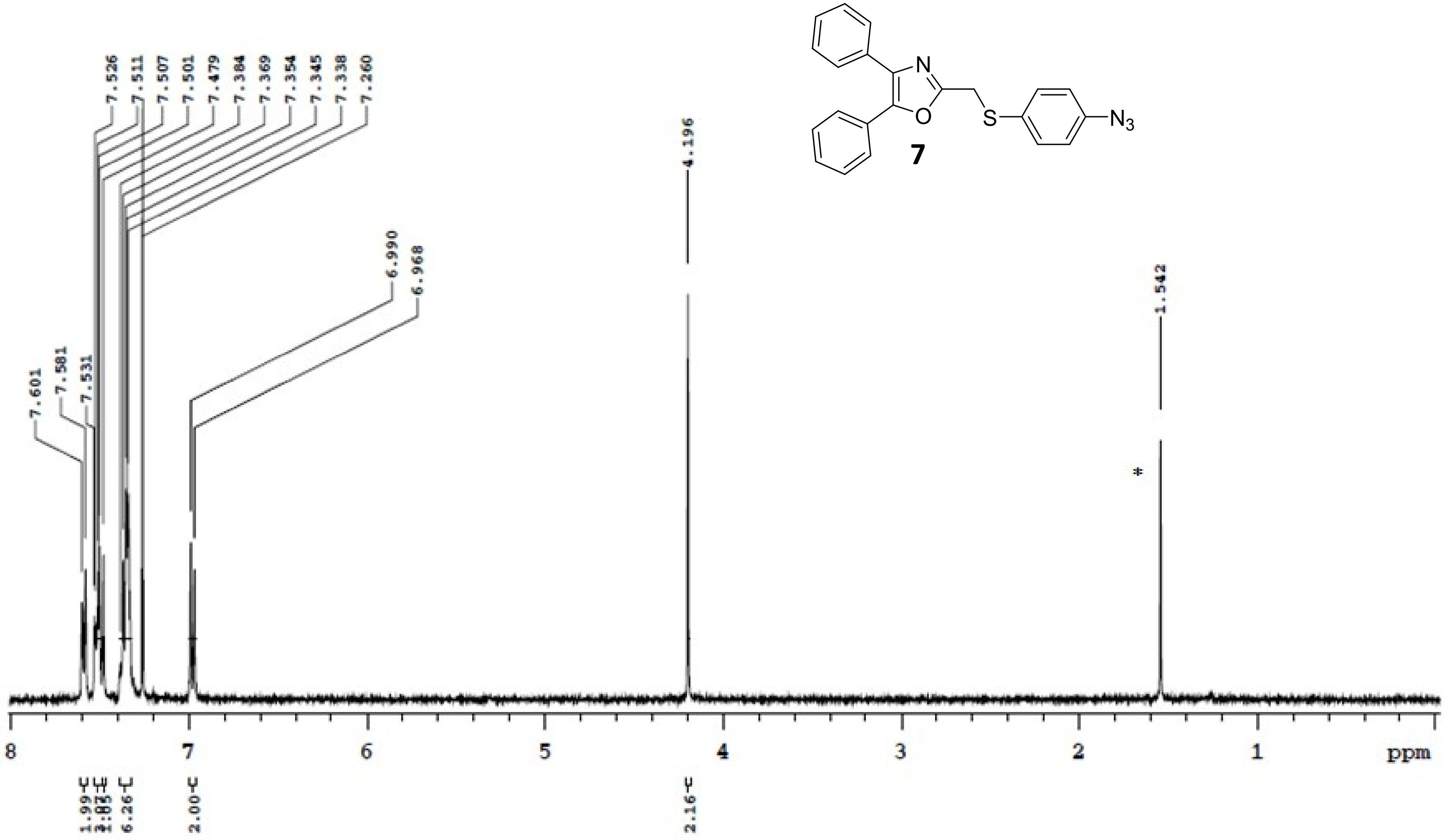

* water traces

^{13}C NMR: 2-(((4-azidophenyl)thio)methyl)-4,5-diphenyloxazole

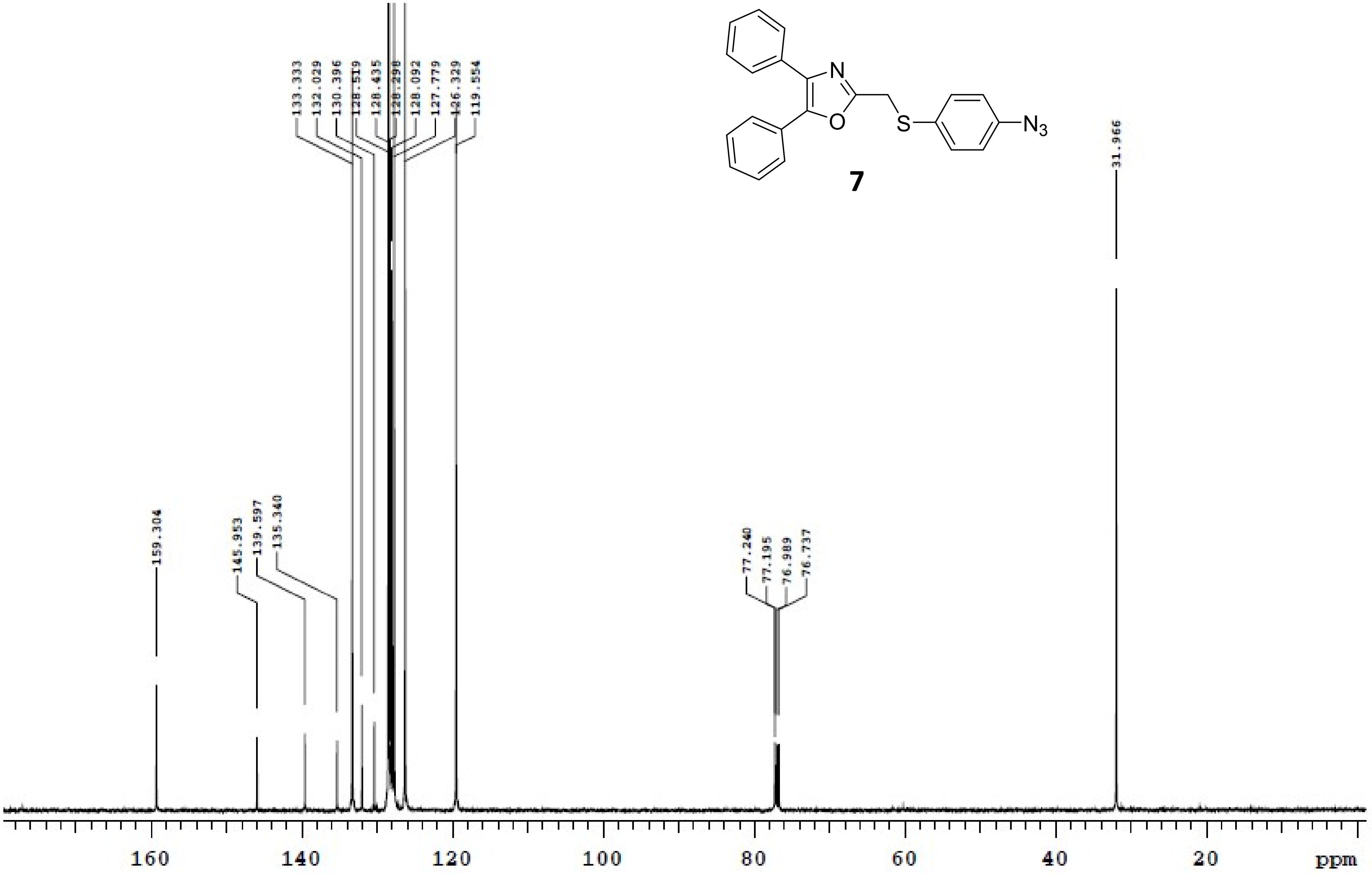

^{1}H NMR: 2-(((4-azidophenyl)thio)methyl)-4,5-bis(4-fluorophenyl)oxazole

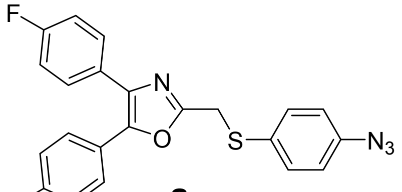

8

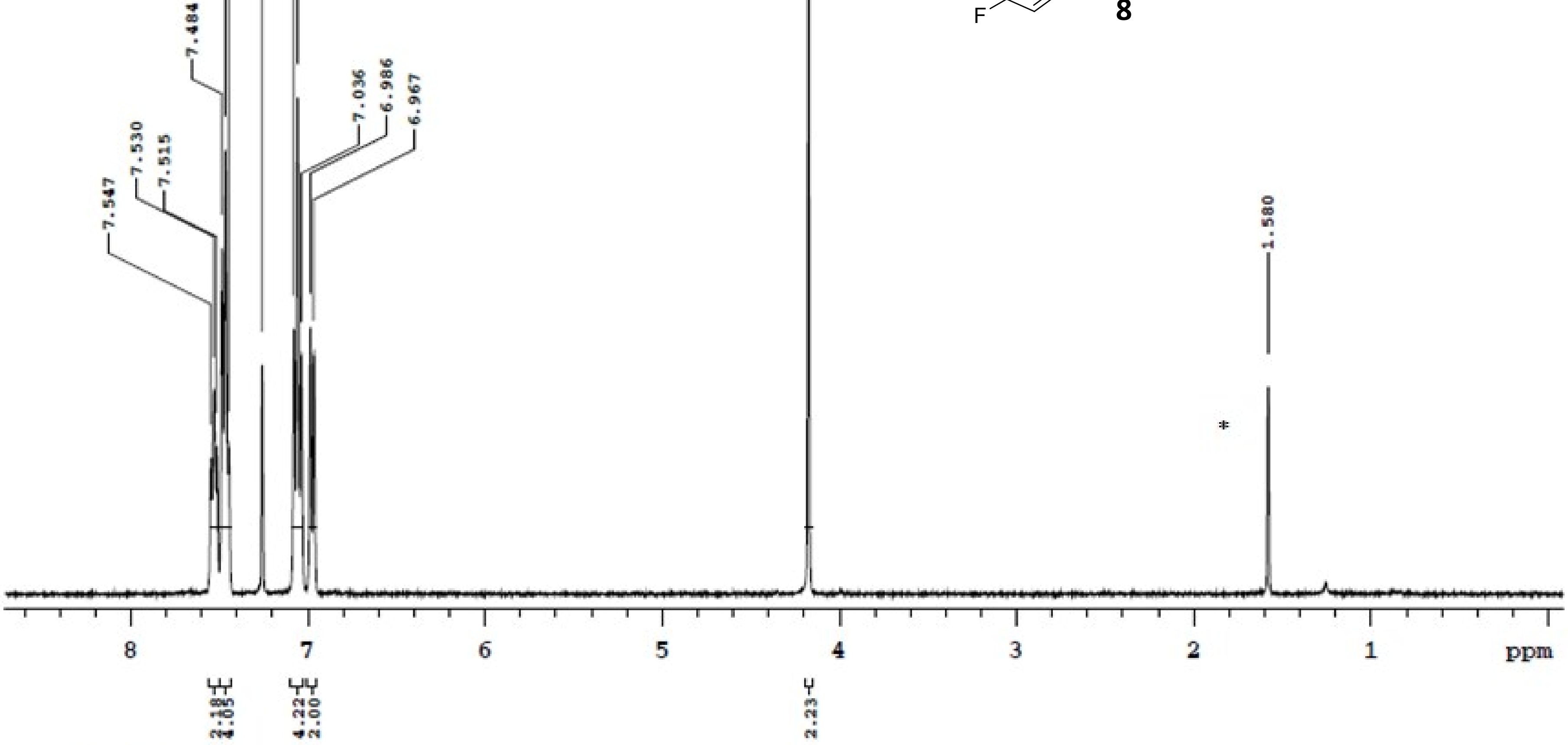

* water traces

^{13}C NMR: 2-(((4-azidophenyl)thio)methyl)-4,5-bis(4-fluorophenyl)oxazole

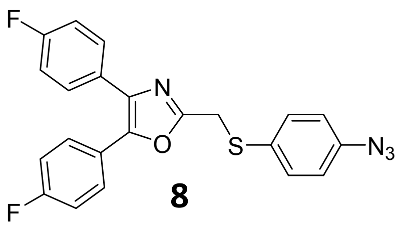

8

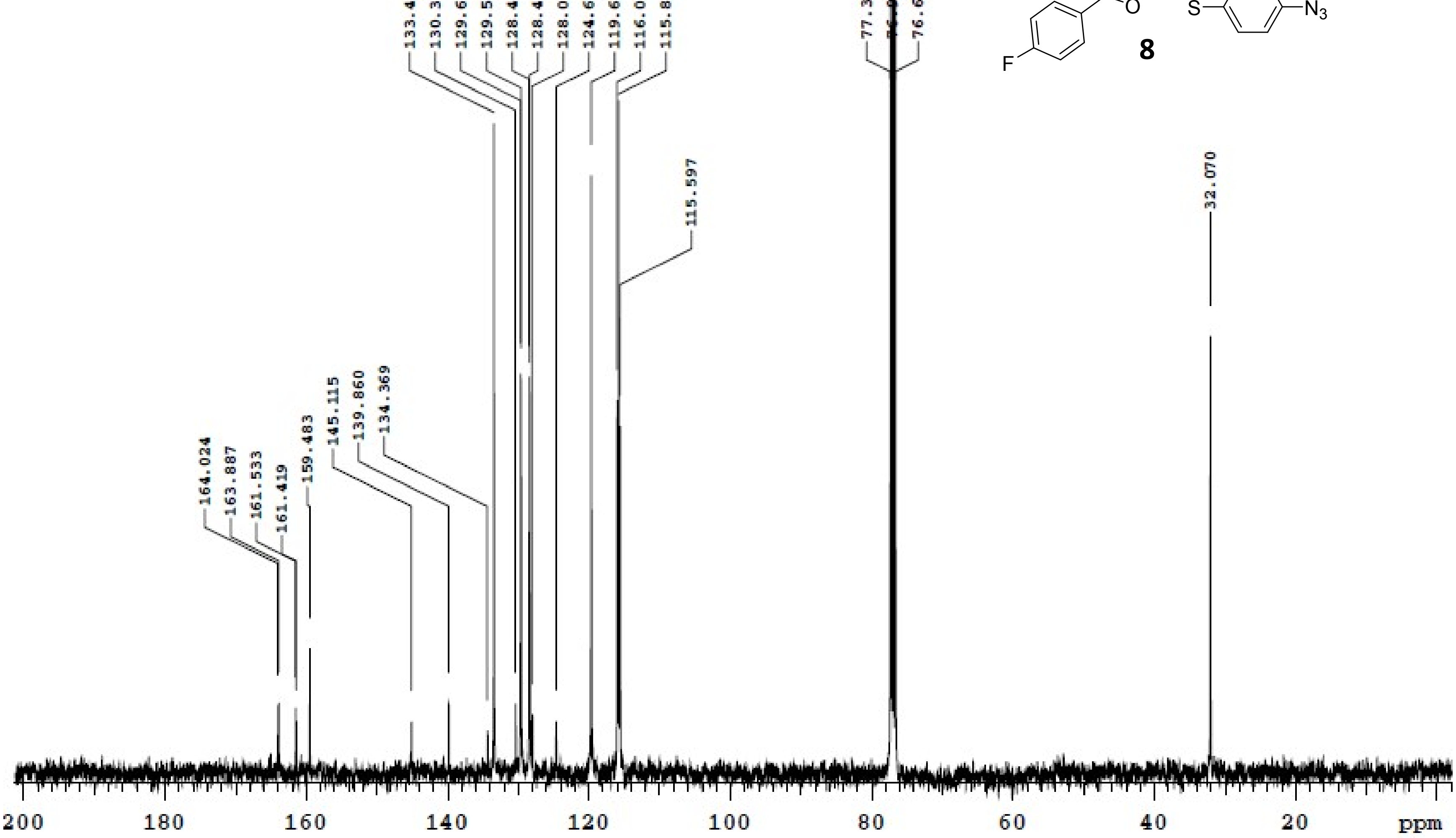

^{1}H NMR: 2-(((4-azidophenyl)thio)methyl)-4,5-bis(4-chlorophenyl)oxazole

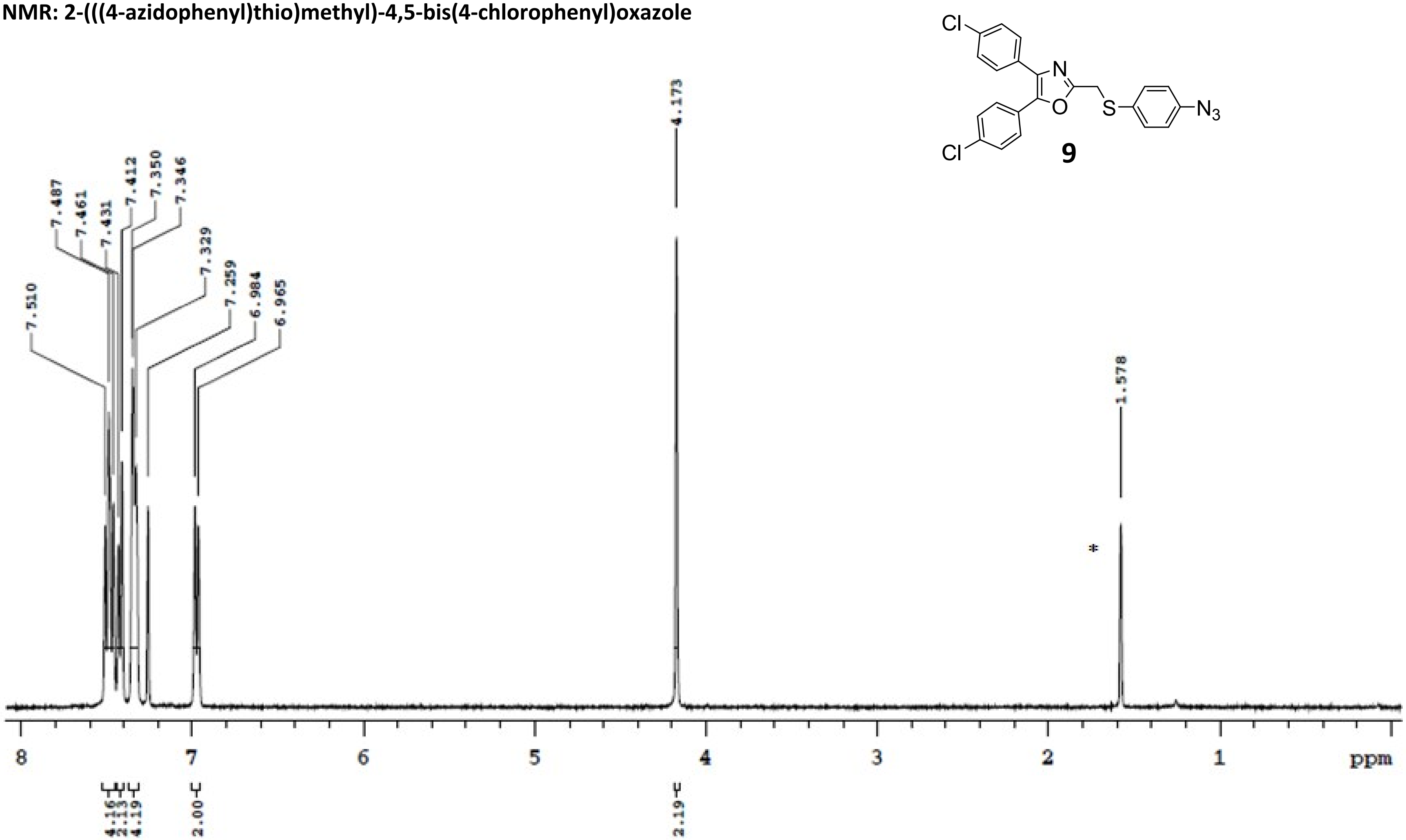

* water traces

^{13}C NMR: 2-(((4-azidophenyl)thio)methyl)-4,5-bis(4-chlorophenyl)oxazole

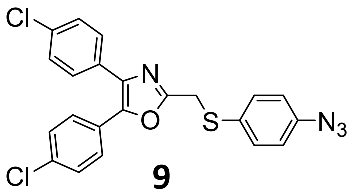

9

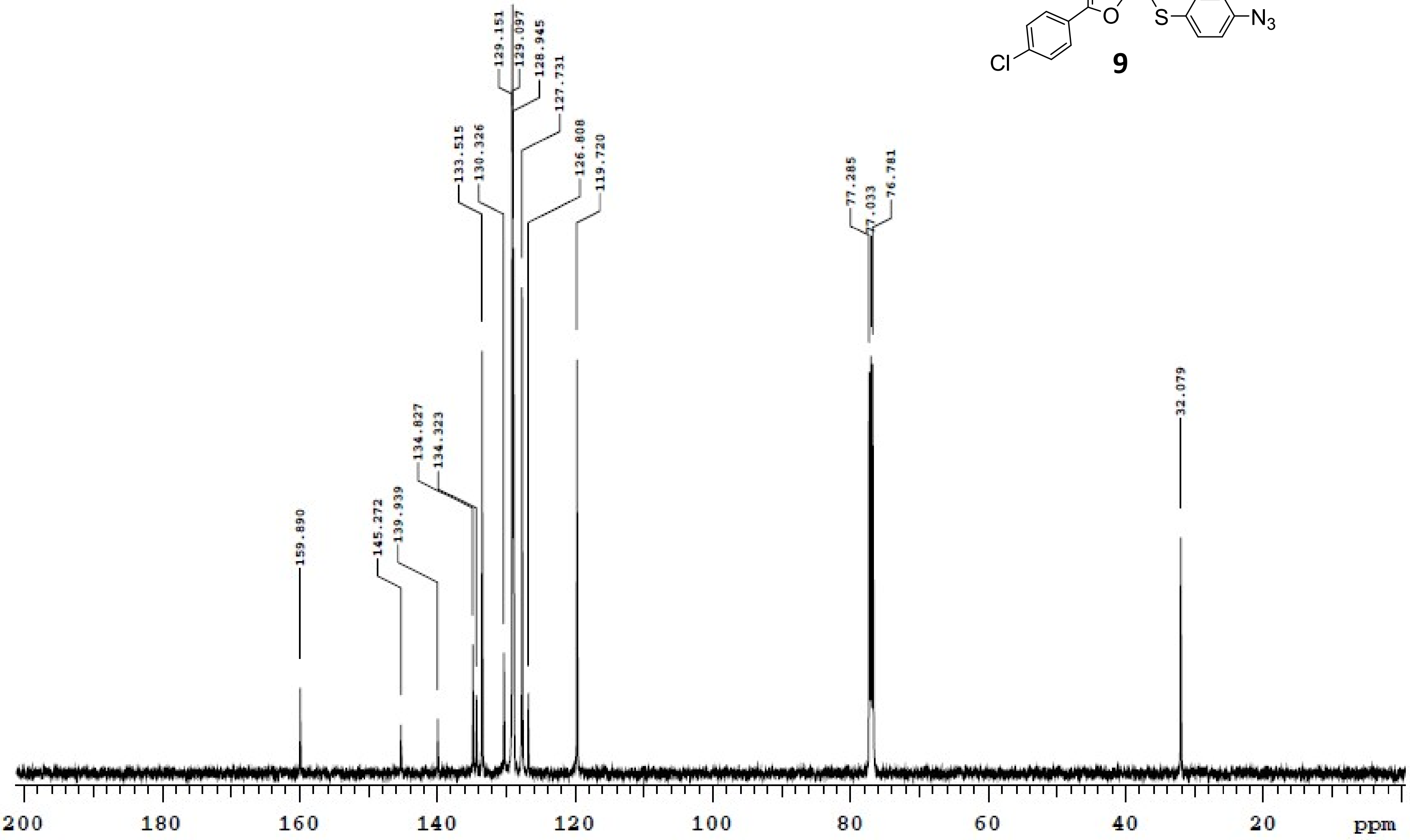

^{1}H NMR: 2-(((4-azidophenyl)sulfinyl)methyl)-4,5-diphenyloxazole

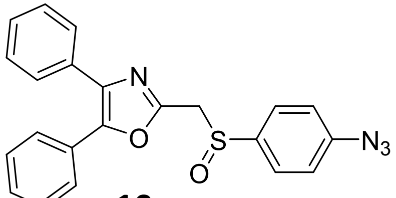

10

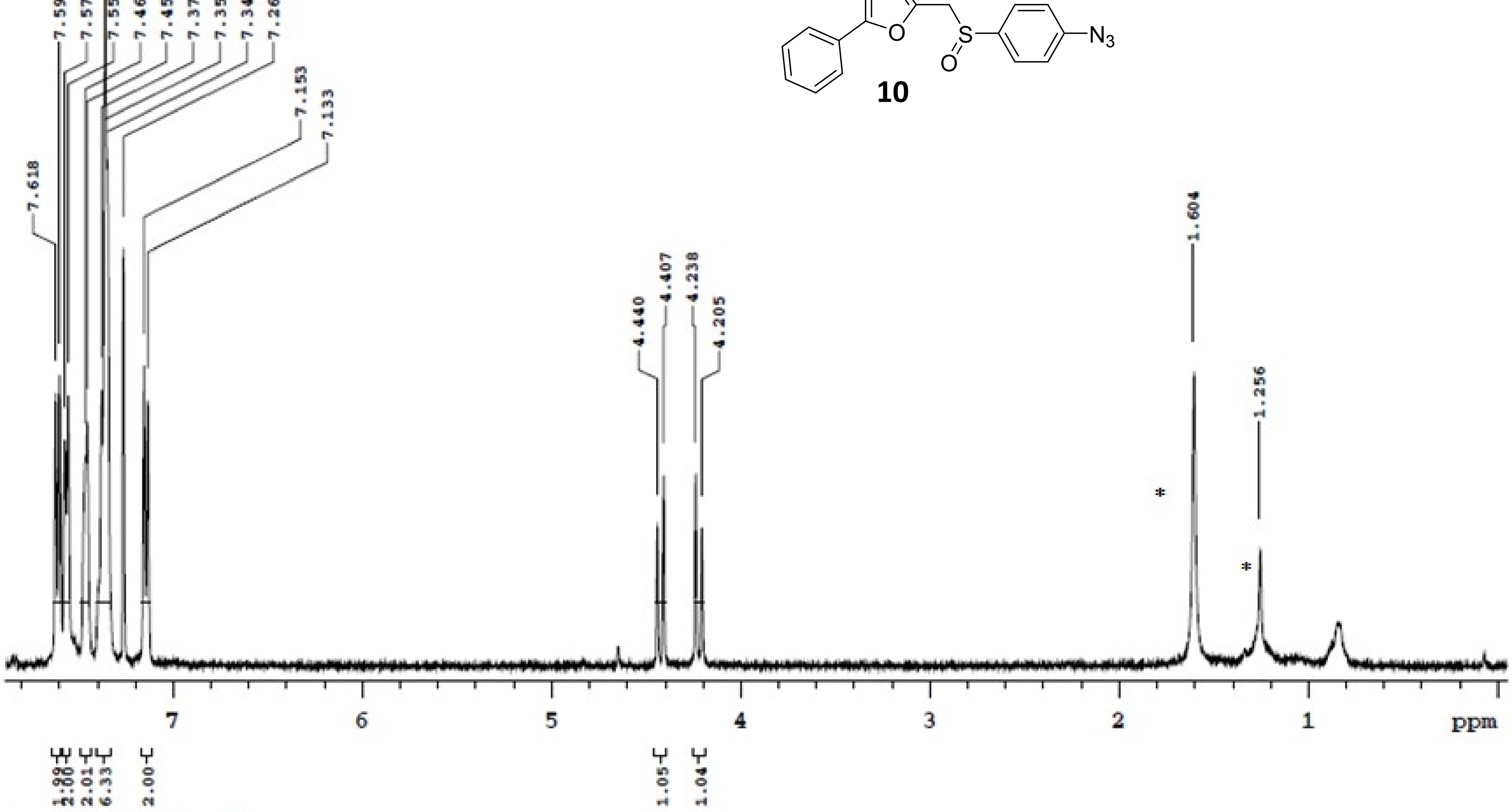

* water and solvent traces

^{13}C NMR: 2-(((4-azidophenyl)sulfinyl)methyl)-4,5-diphenyloxazole

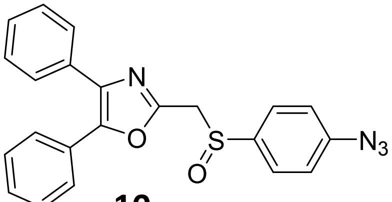

10

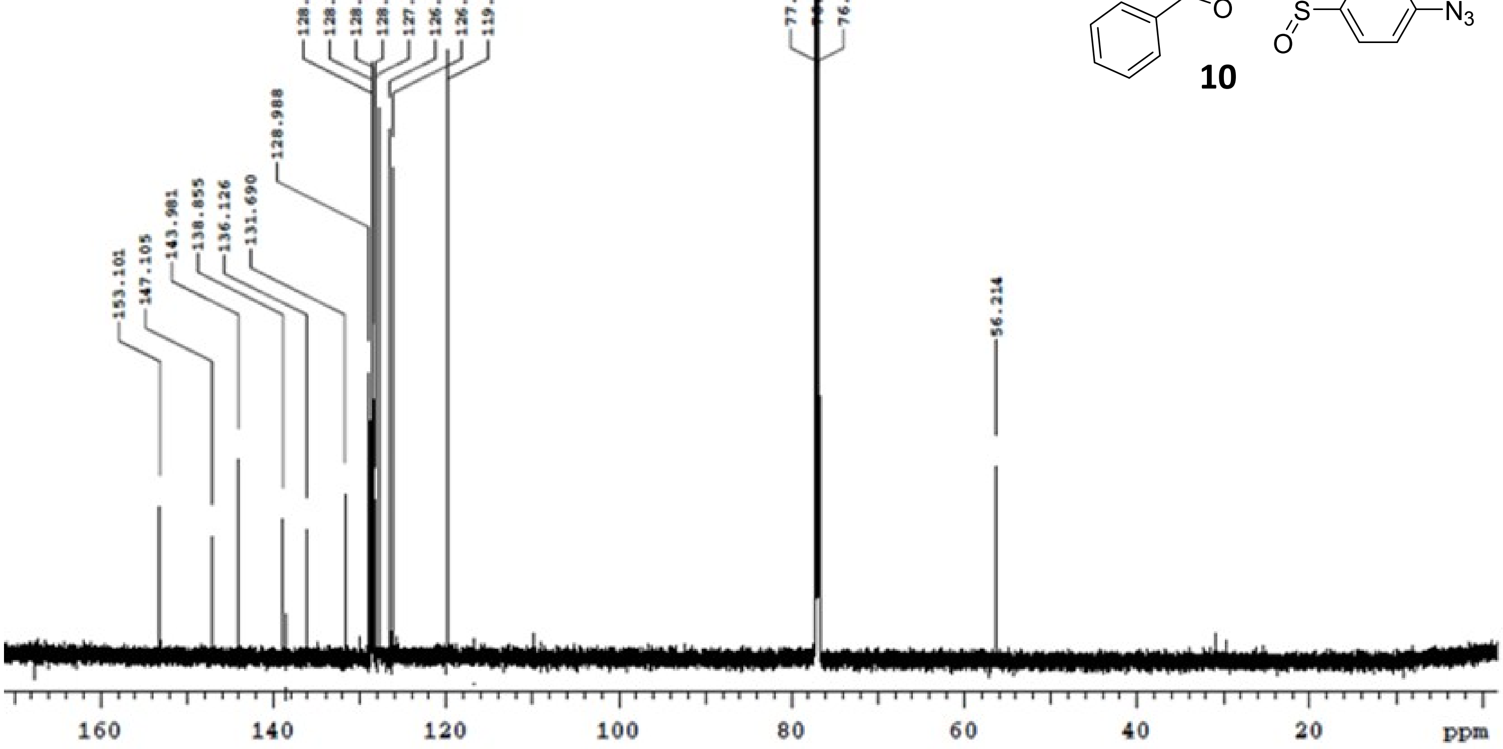

^{1}H NMR: 2-(((4-azidophenyl)sulfinyl)methyl)-4,5-bis(4-fluorophenyl)oxazole

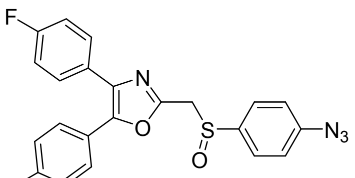

11

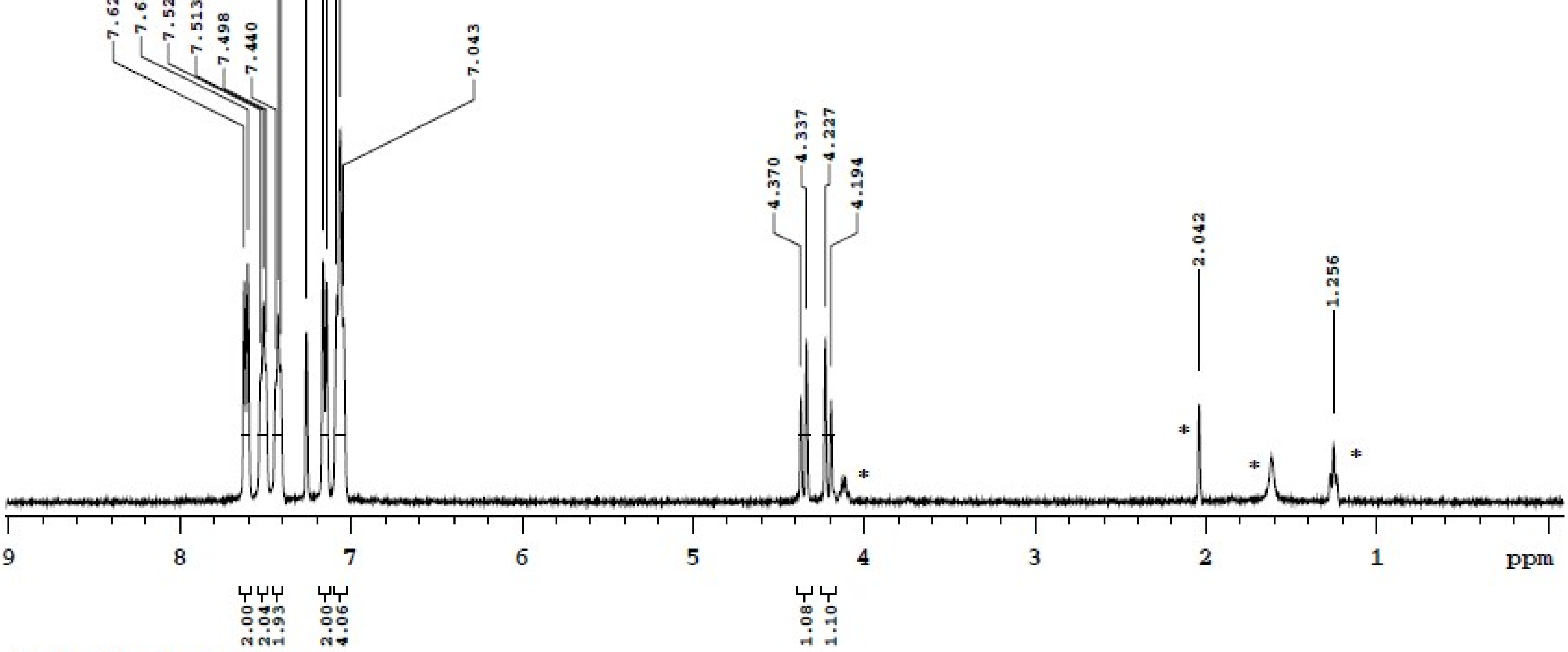

* solvent and water traces

^{13}C NMR: 2-(((4-azidophenyl)sulfinyl)methyl)-4,5-bis(4-fluorophenyl)oxazole

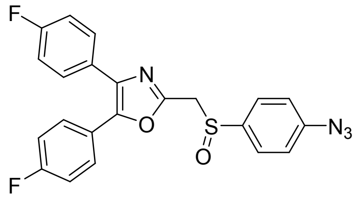

11

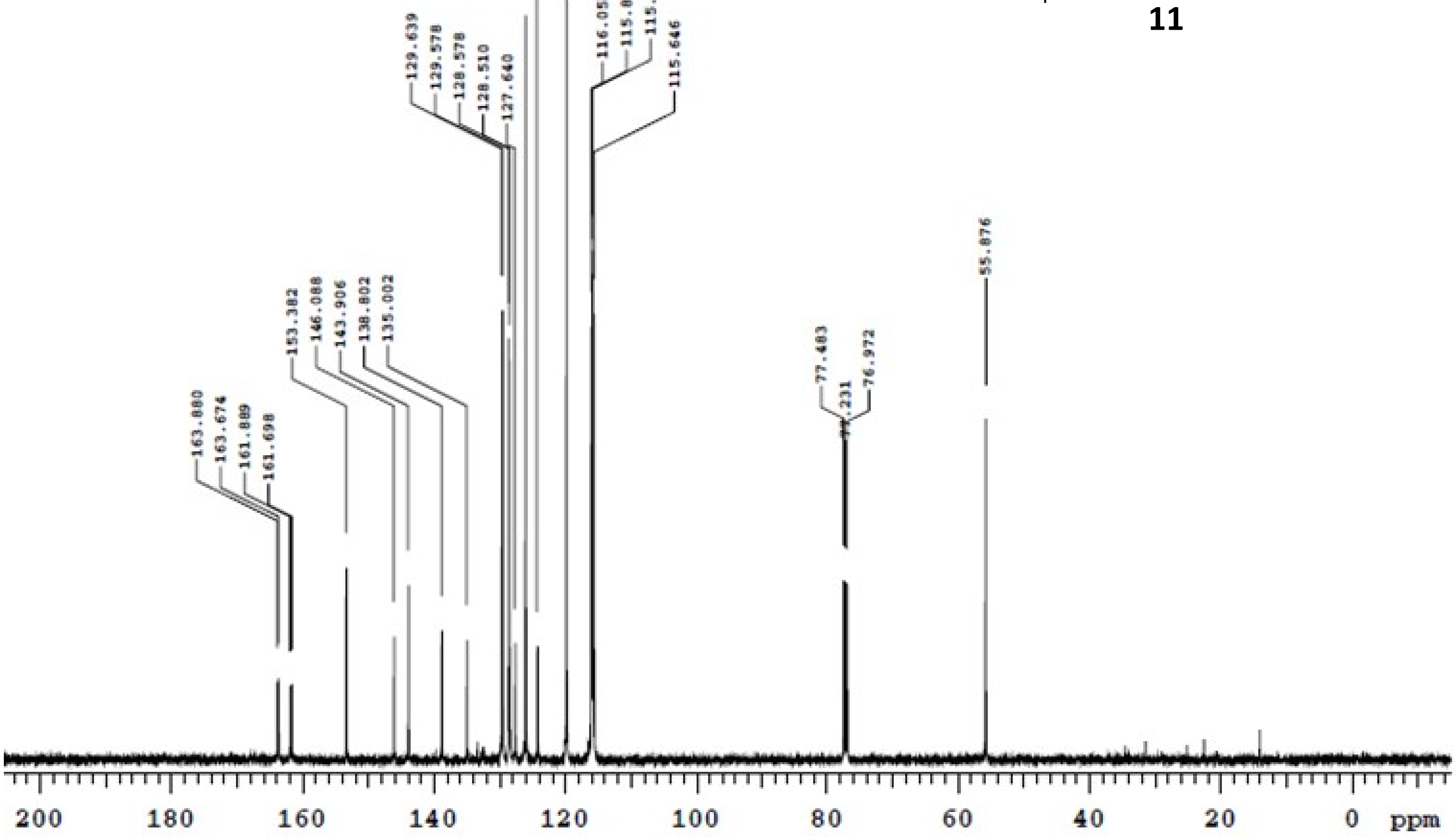

^{1}H NMR: 2-(((4-azidophenyl)sulfinyl)methyl)-4,5-bis(4-chlorophenyl)oxazole

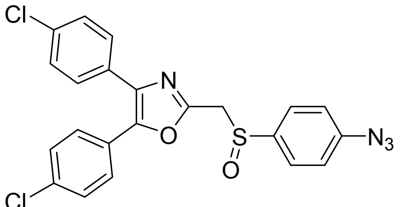

12

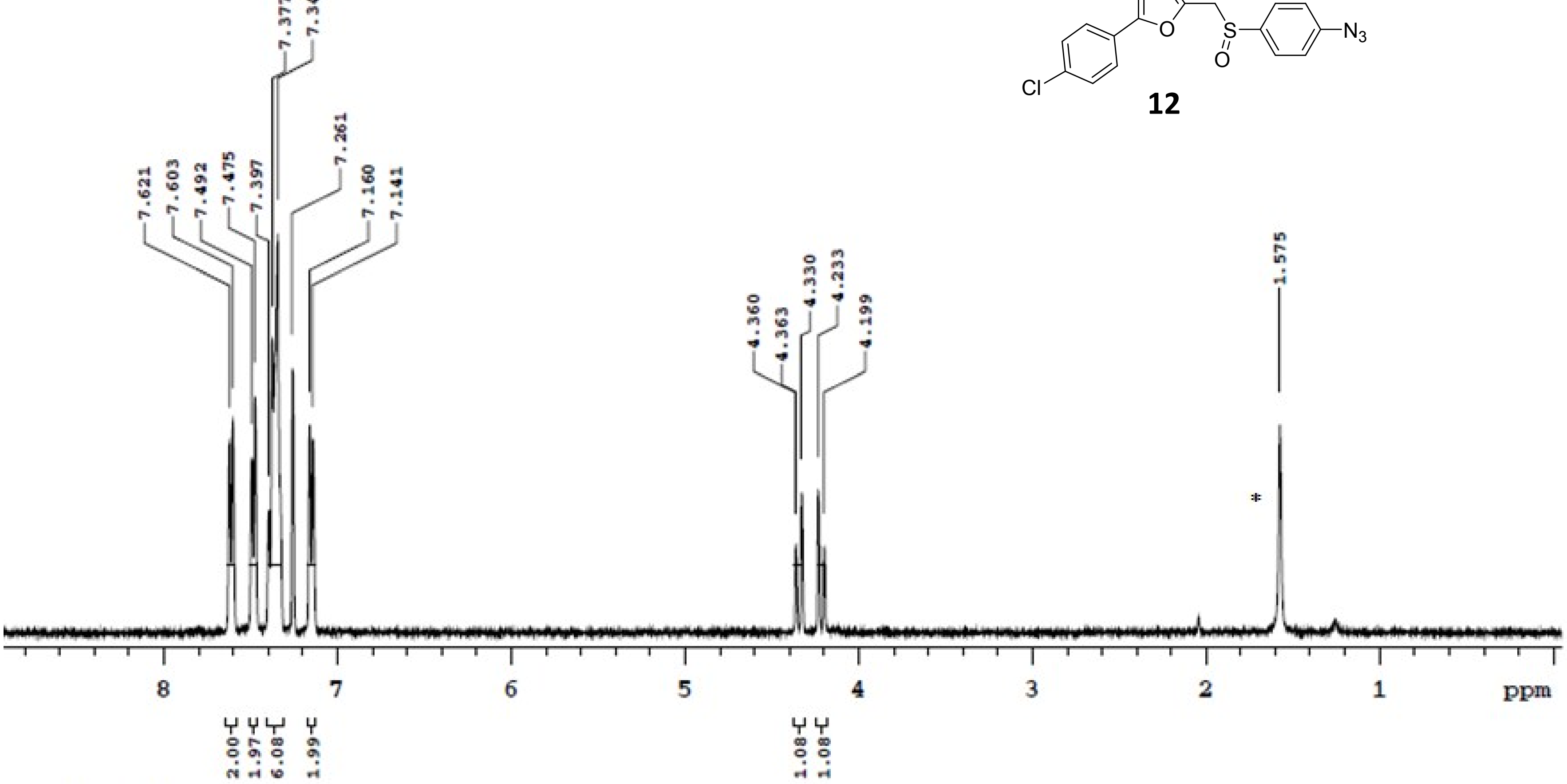

* water traces

^{13}C NMR: 2-(((4-azidophenyl)sulfinyl)methyl)-4,5-bis(4-chlorophenyl)oxazole

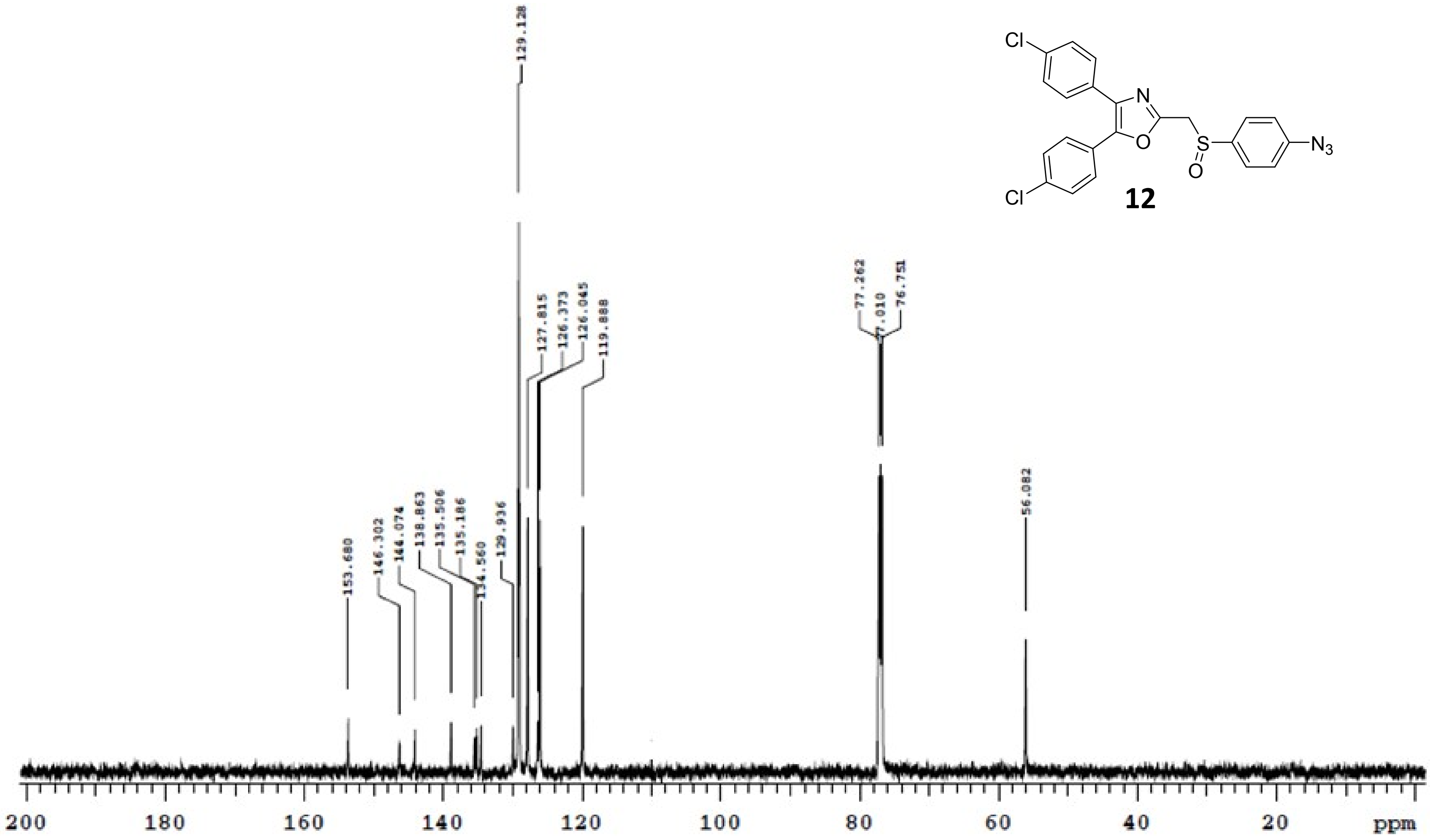

12

^{1}H NMR: 2-(((4-azidophenyl)sulfonyl)methyl)-4,5-diphenyloxazole

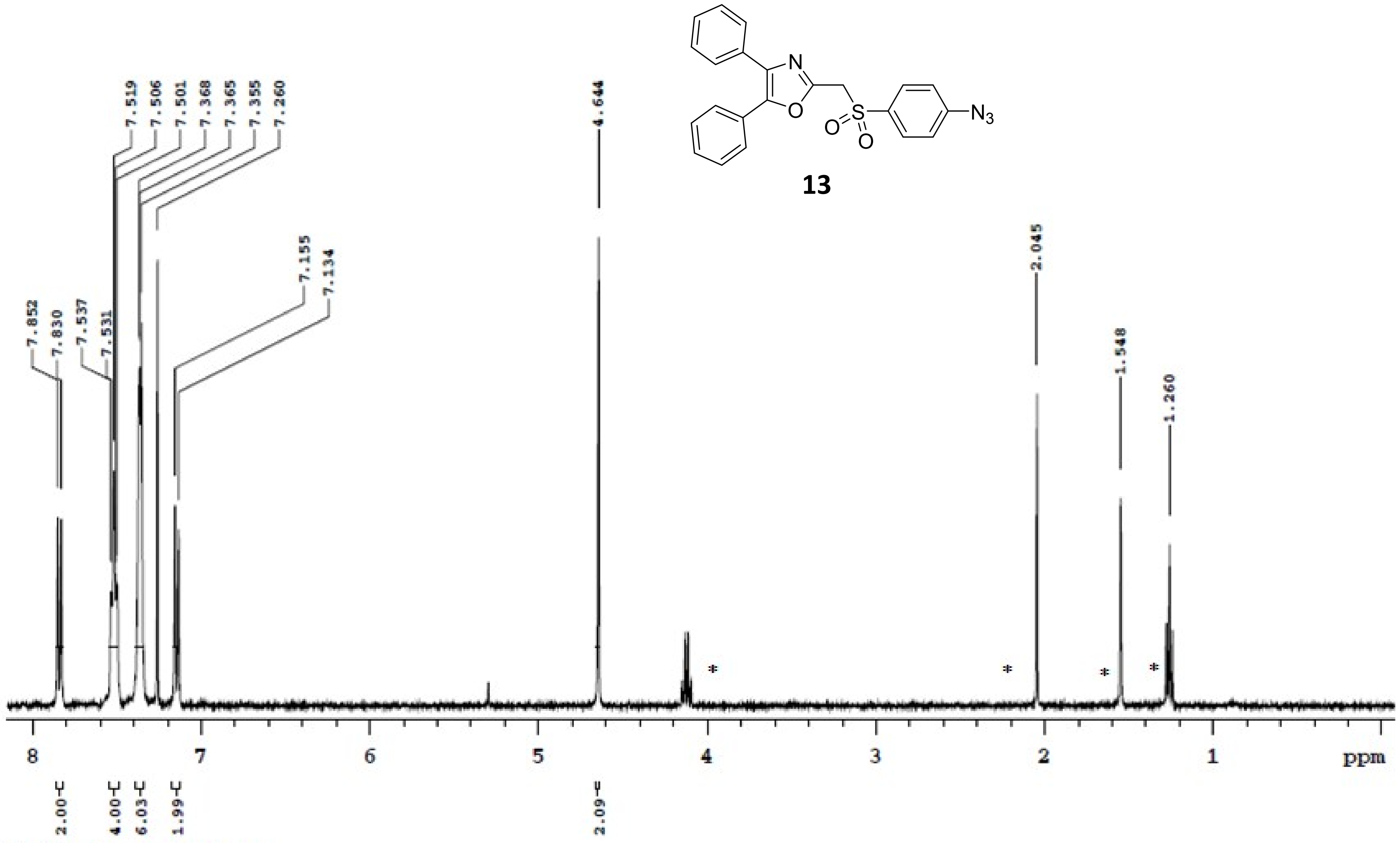

13

* solvent and water traces

^{13}C NMR: 2-(((4-azidophenyl)sulfonyl)methyl)-4,5-diphenyloxazole

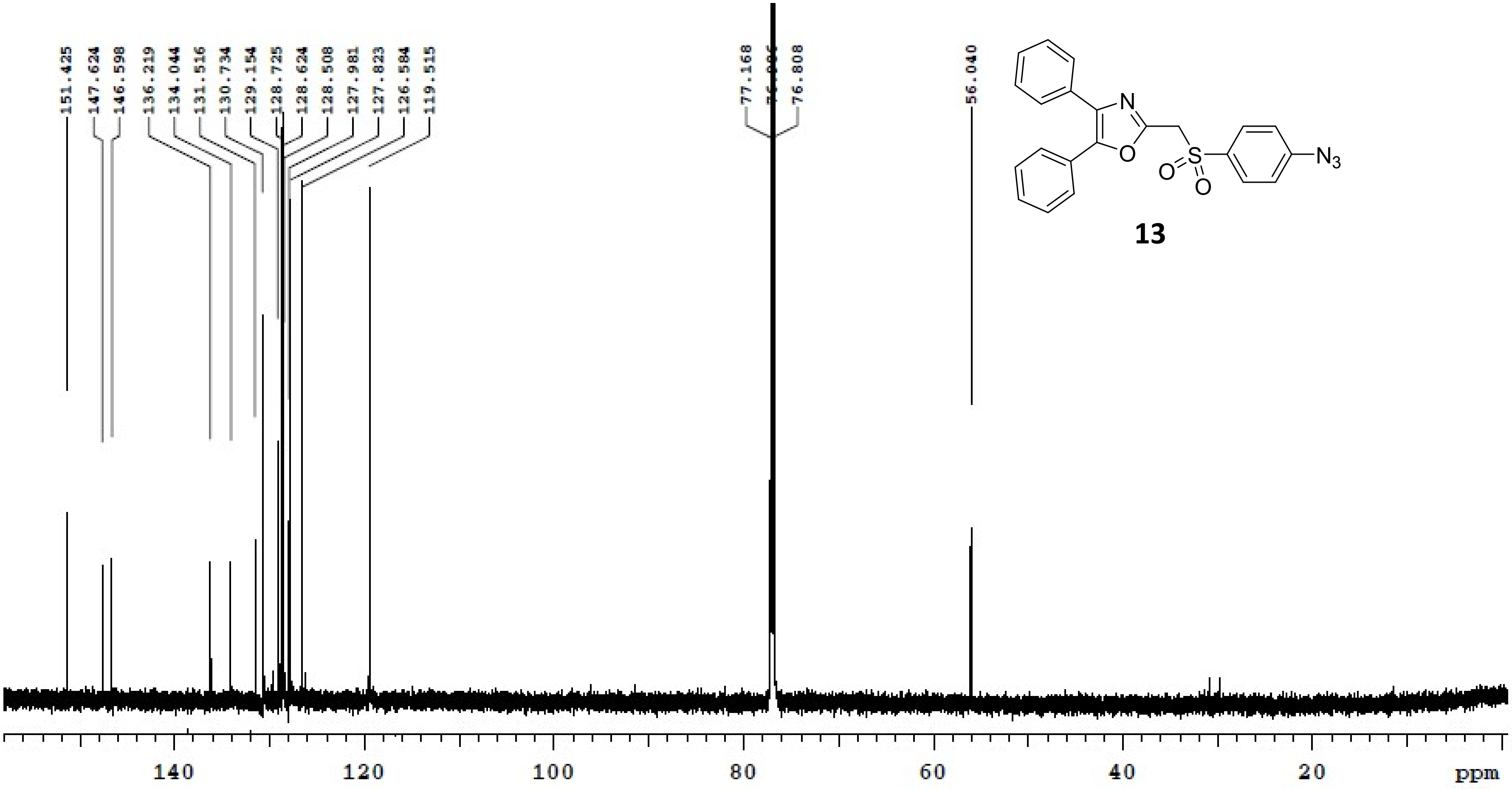

^{1}H NMR:2-(((4-azidophenyl)sulfonyl)methyl)-4,5-bis(4-fluorophenyl)oxazole

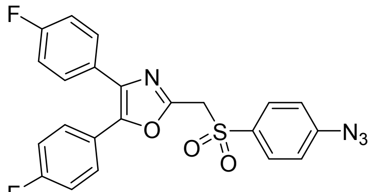

14

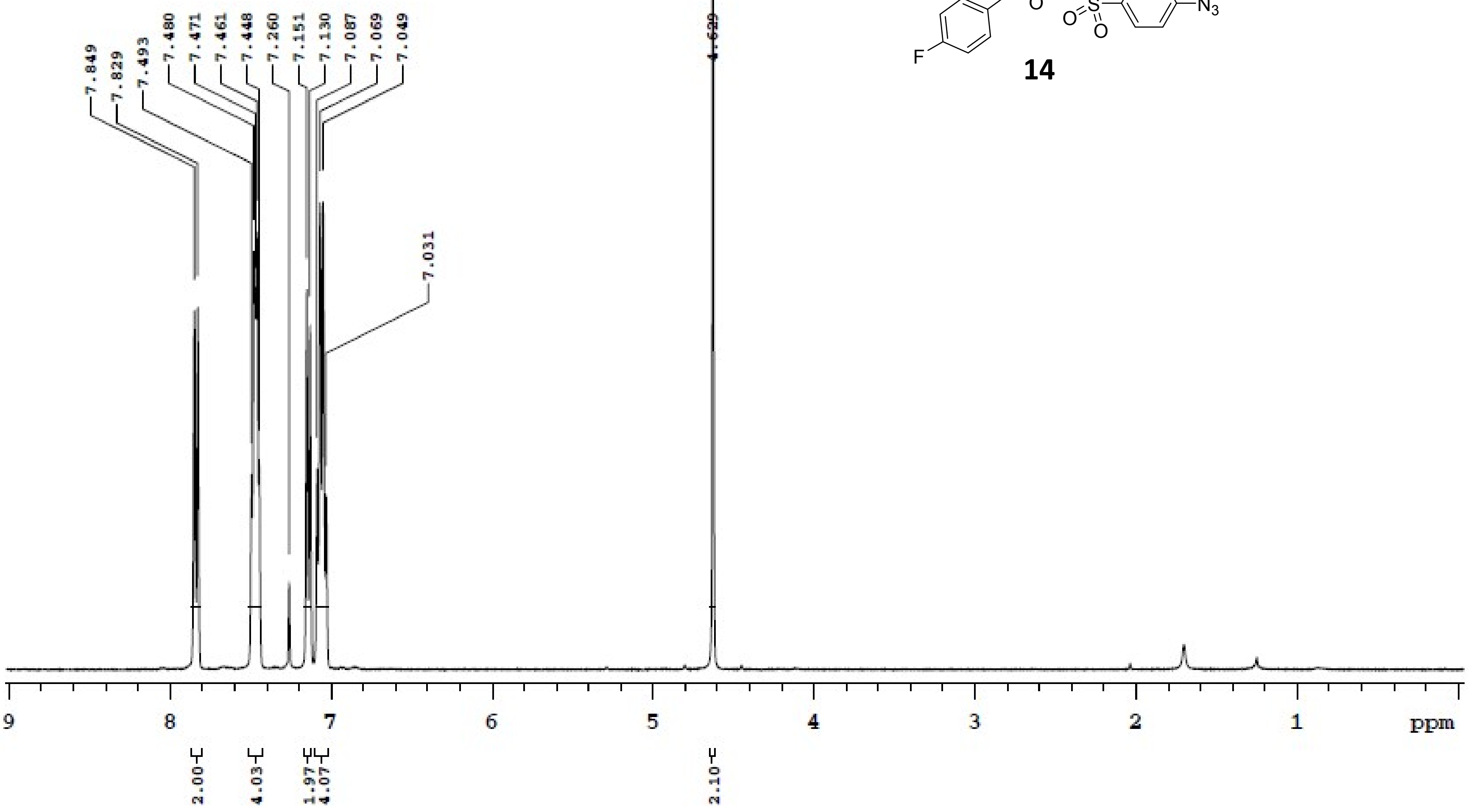

^{13}C NMR: 2-(((4-azidophenyl)sulfonyl)methyl)-4,5-bis(4-fluorophenyl)oxazole

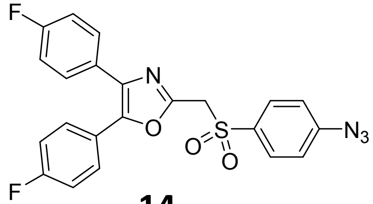

14

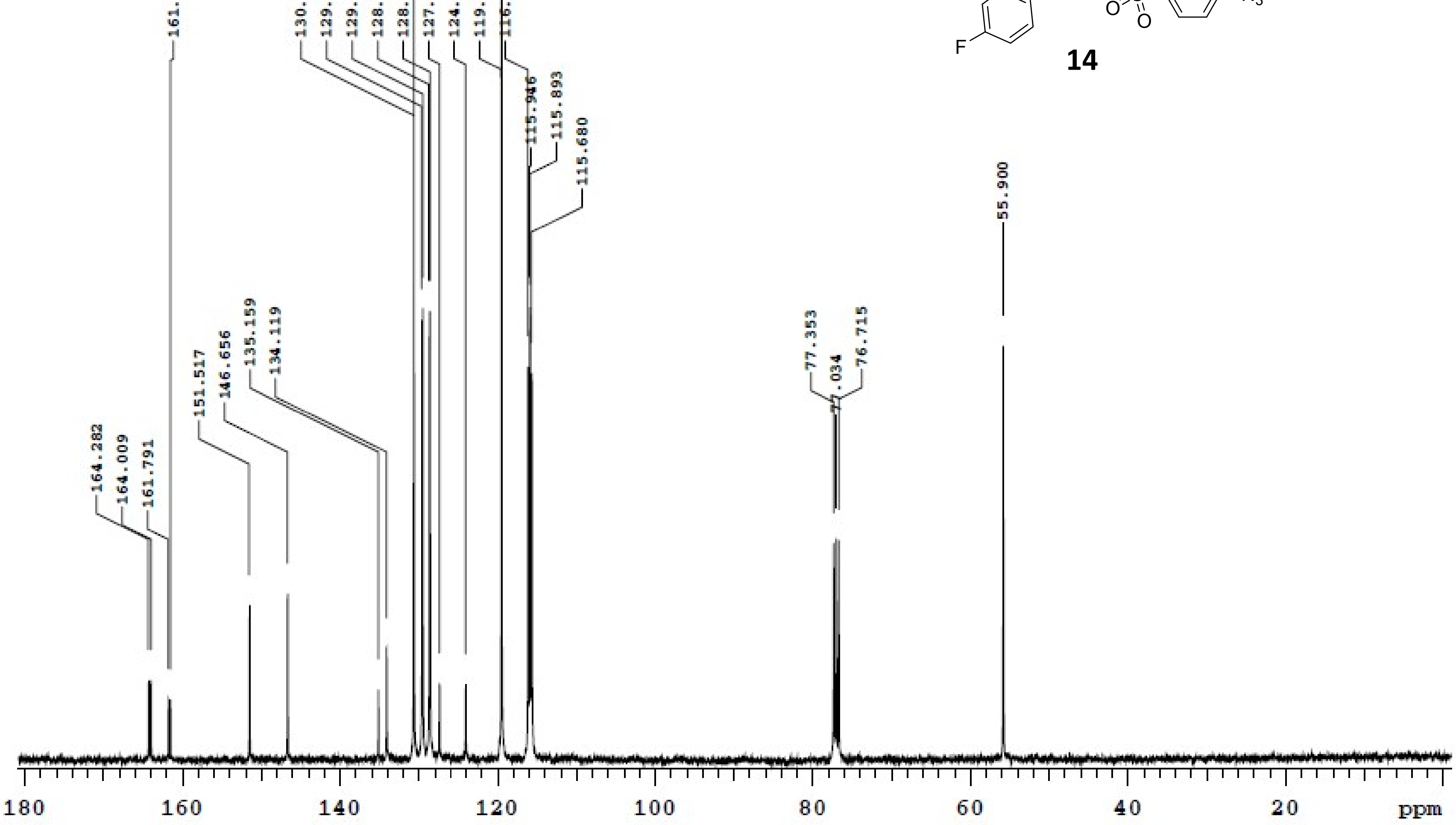

^{1}H NMR: 2-(((4-azidophenyl)sulfonyl)methyl)-4,5-bis(4-chlorophenyl)oxazole

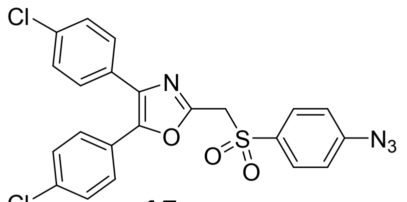

15

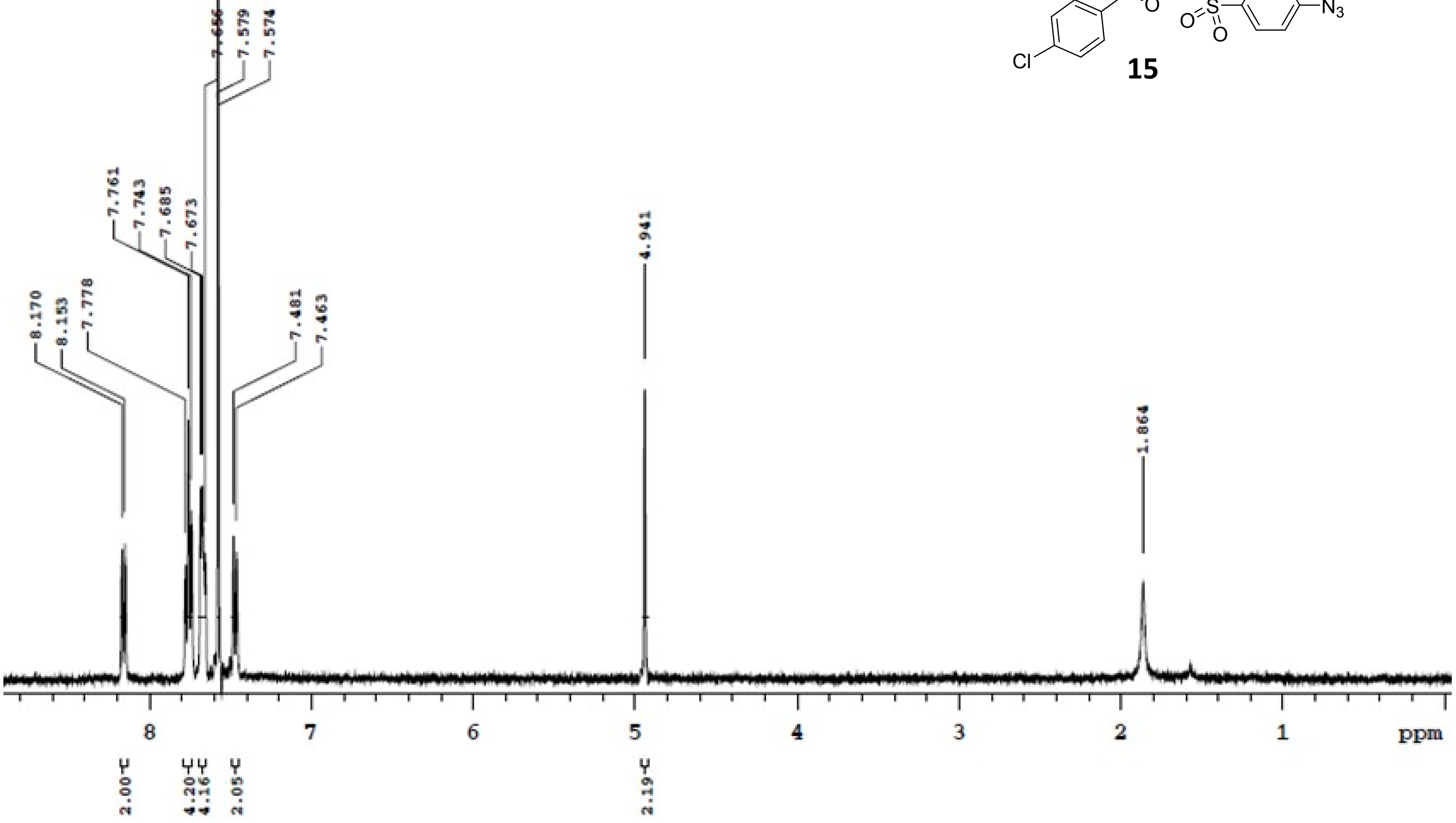

^{13}C NMR: 2-(((4-azidophenyl)sulfonyl)methyl)-4,5-bis(4-chlorophenyl)oxazole

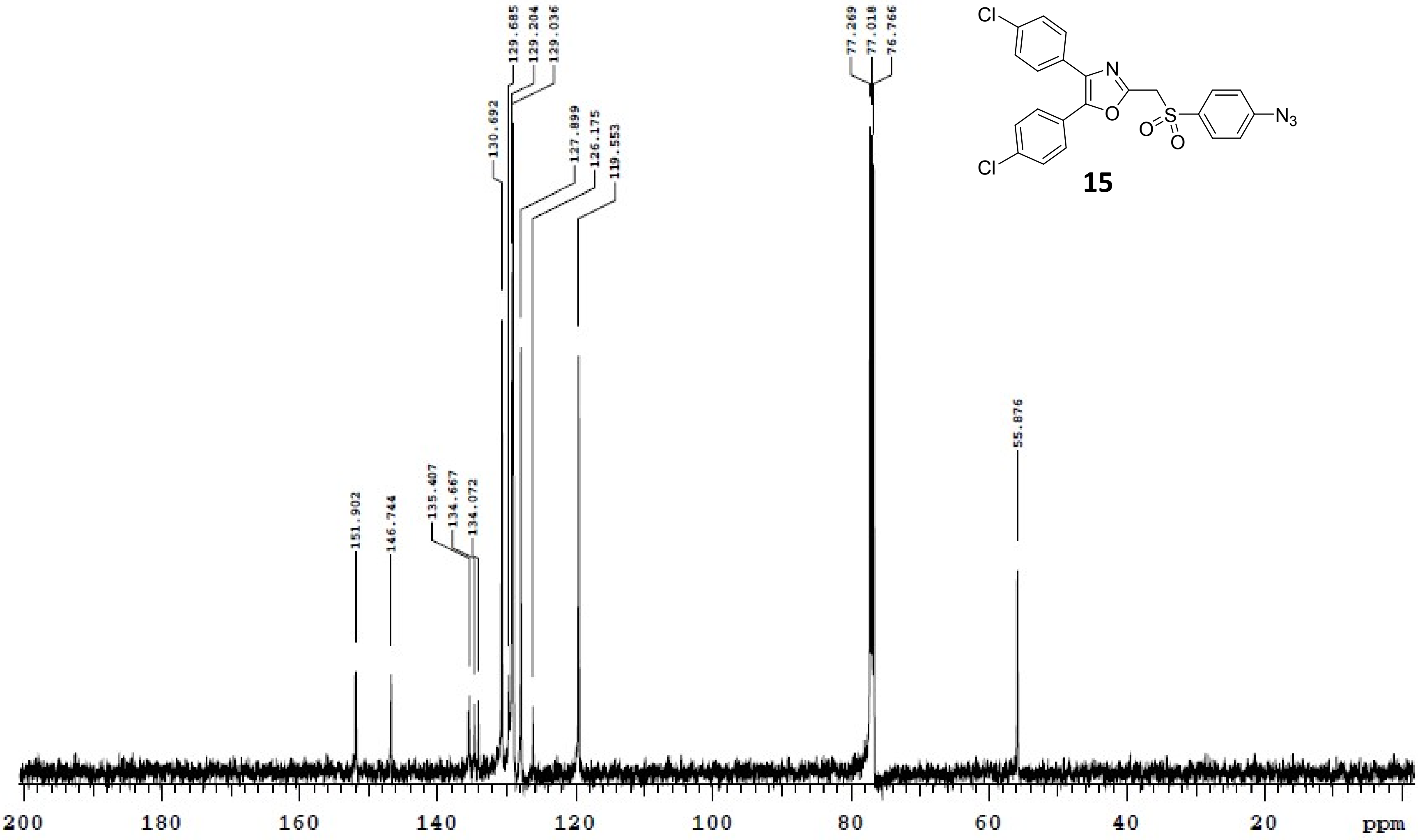

^{1}H NMR : N2,N2,N4,N4-tetraethyl-6-ethynyl-1,3,5-triazine-2,4-diamine

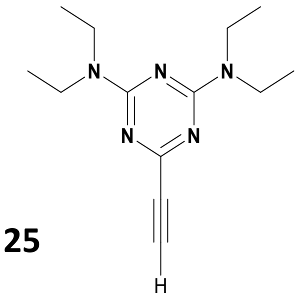

25

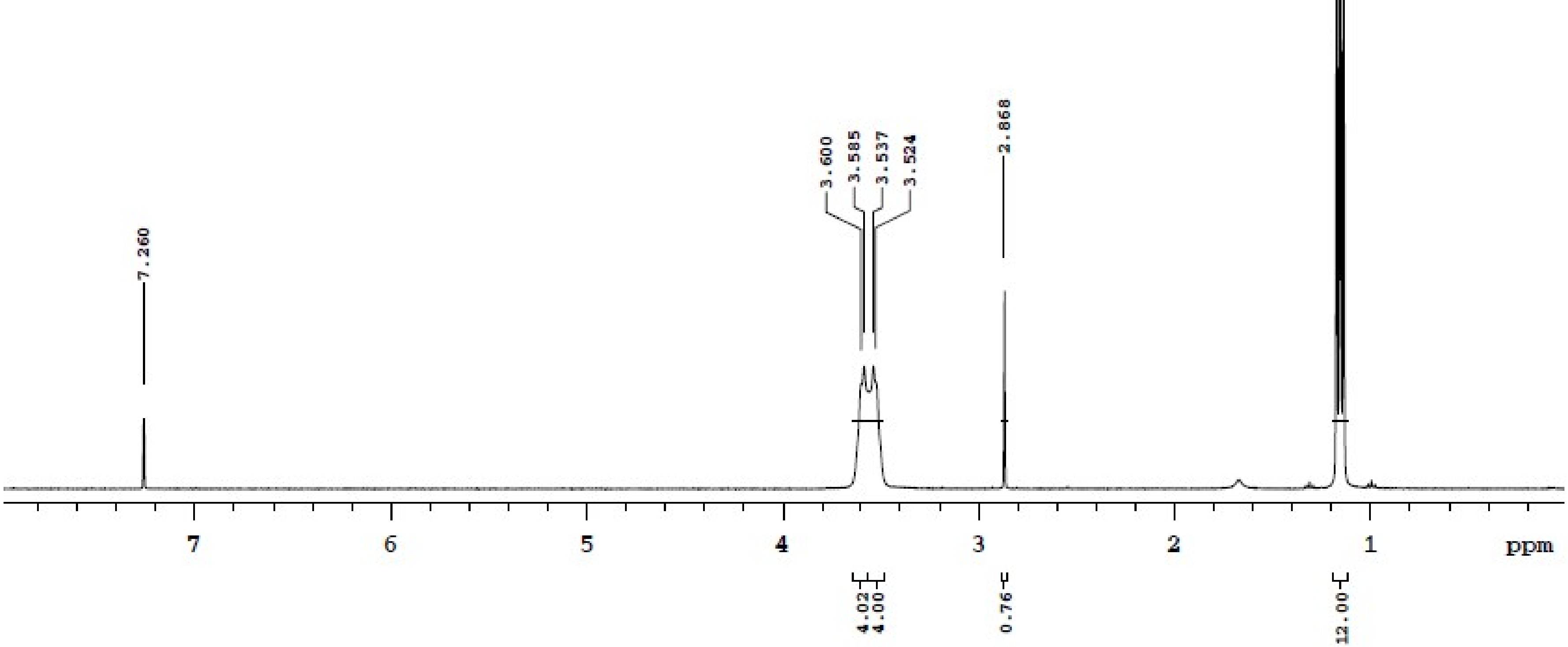

^{13}C NMR: N2,N2,N4,N4-tetraethyl-6-ethynyl-1,3,5-triazine-2,4-diamine

25

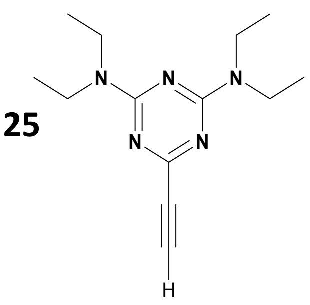

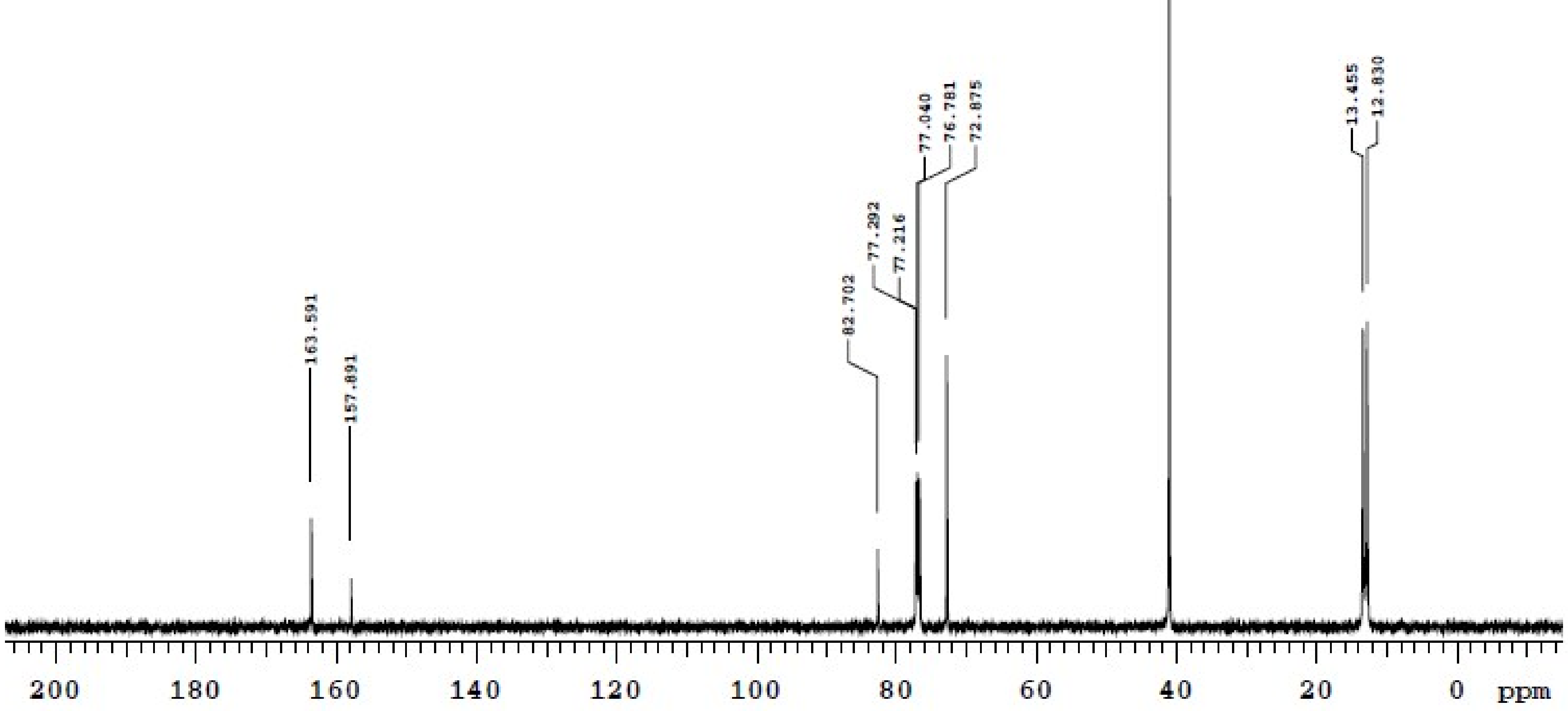

^{1}H NMR : 6-ethynyl-N2,N4-bis(2-fluorophenyl)-1,3,5-triazine-2,4-diamine

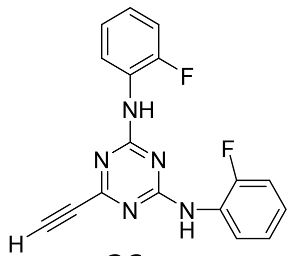

26

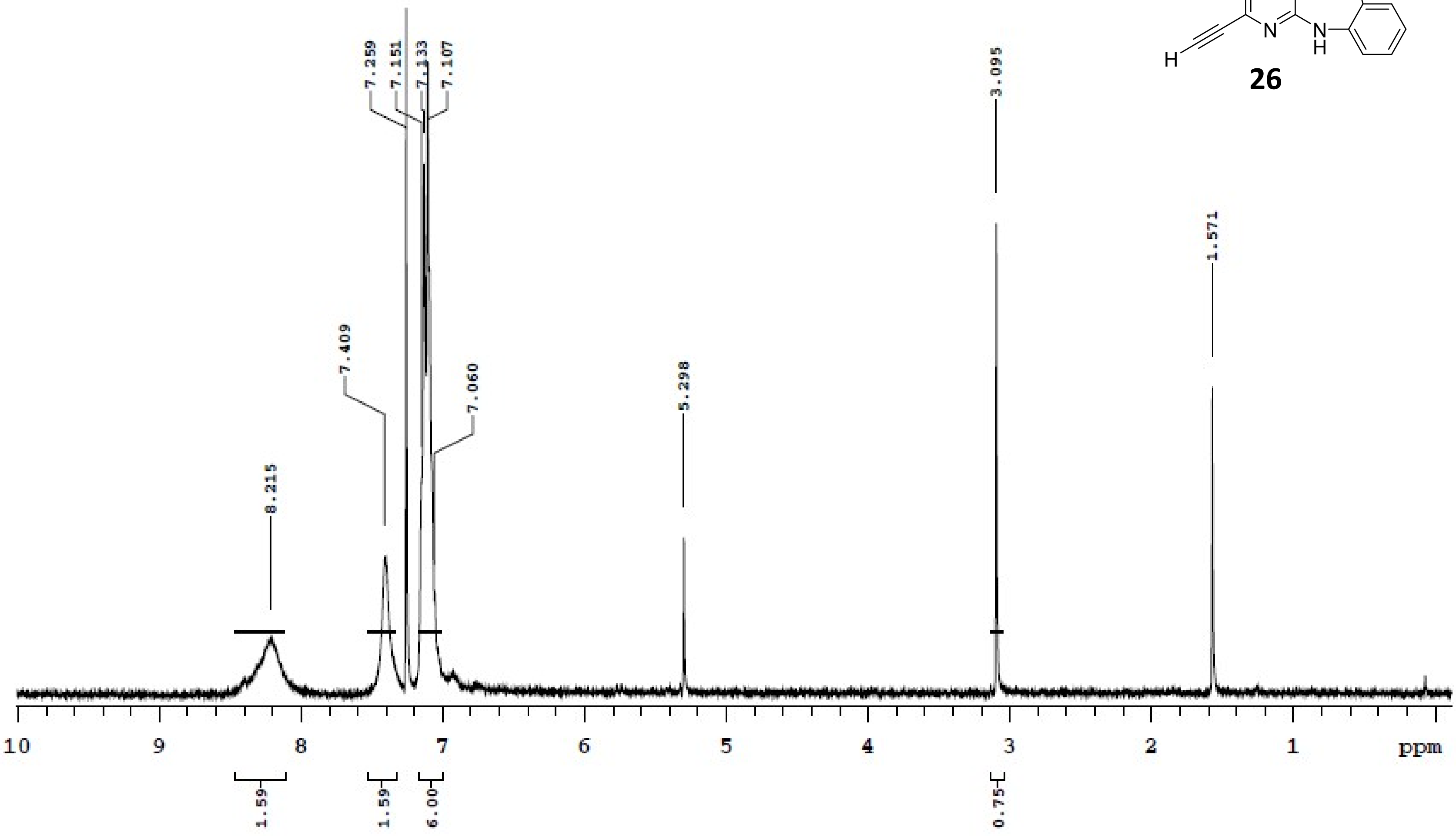

^{13}C NMR : 6-ethynyl-N2,N4-bis(2-fluorophenyl)-1,3,5-triazine-2,4-diamine

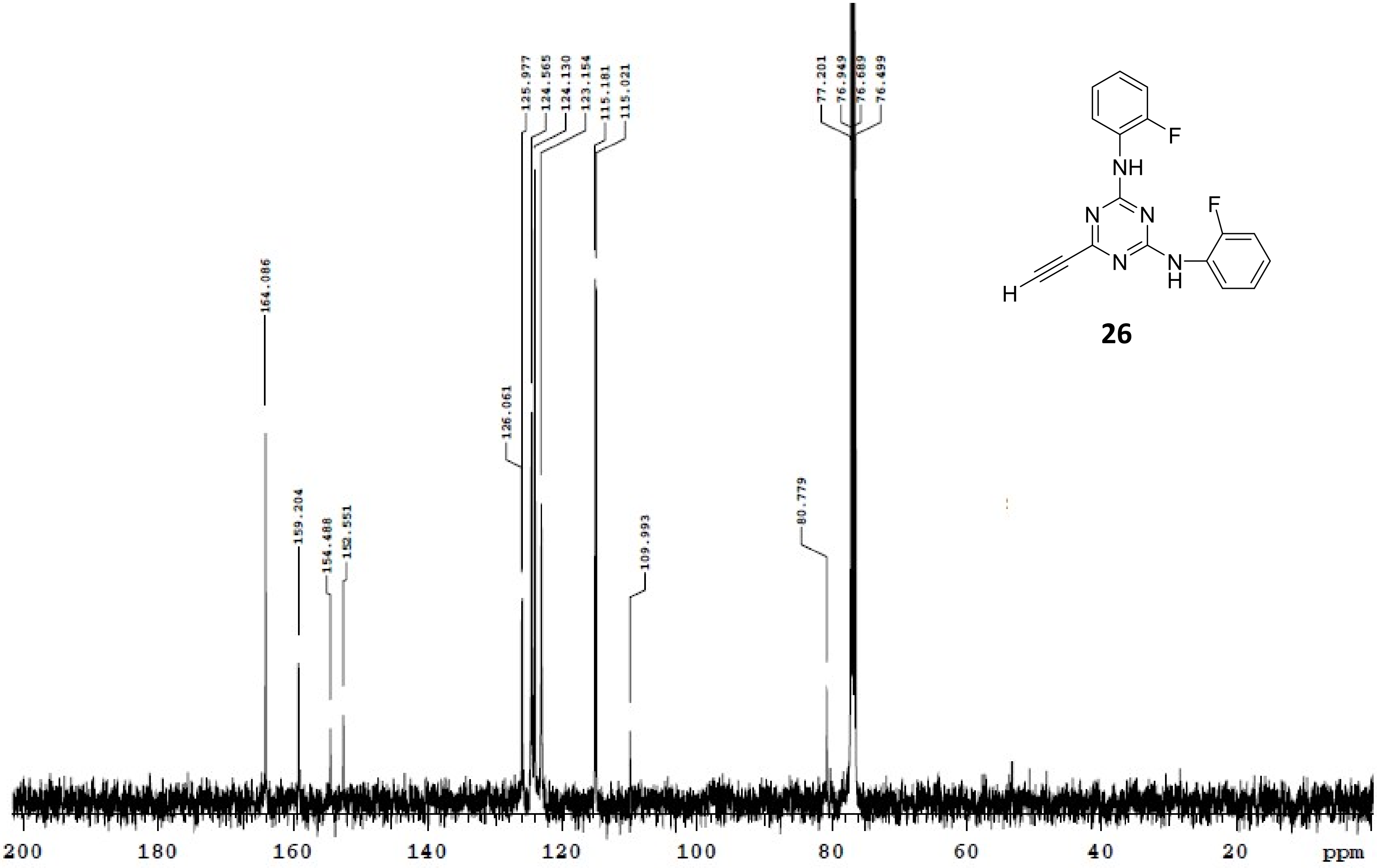

^{1}H NMR: 6-ethynyl-N2,N4-bis(4-fluorophenyl)-1,3,5-triazine-2,4-diamine

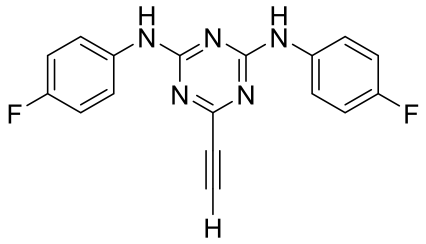

27

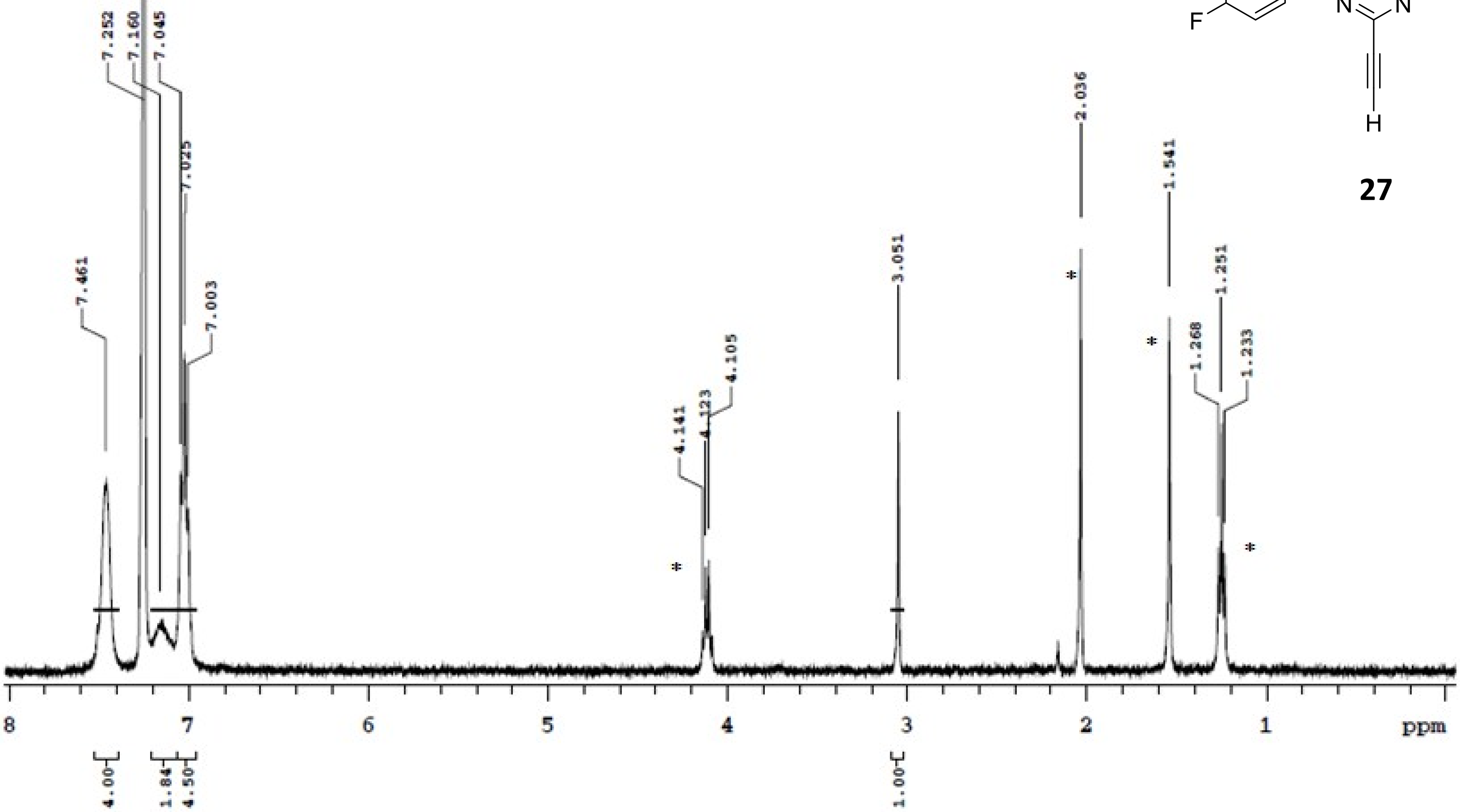

* solvent traces

^{13}C NMR: 6-ethynyl-N2,N4-bis(4-fluorophenyl)-1,3,5-triazine-2,4-diamine

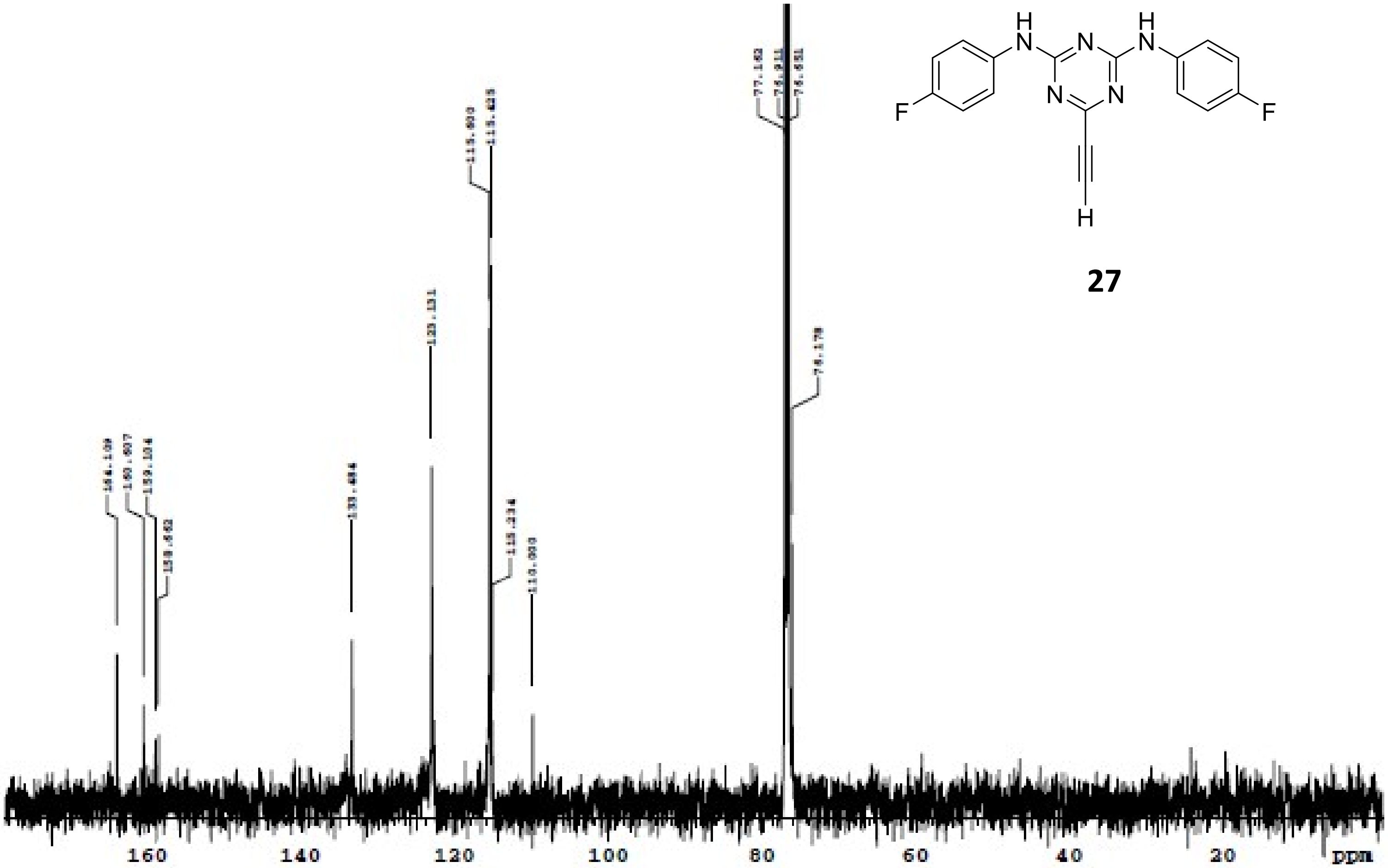

27

^{1}H NMR: 4,5-diphenyl-2-(((4-(4-(pyridin-3-yl)-1H-1,2,3-triazol-1-yl)phenyl)sulfinyl)methyl)oxazole

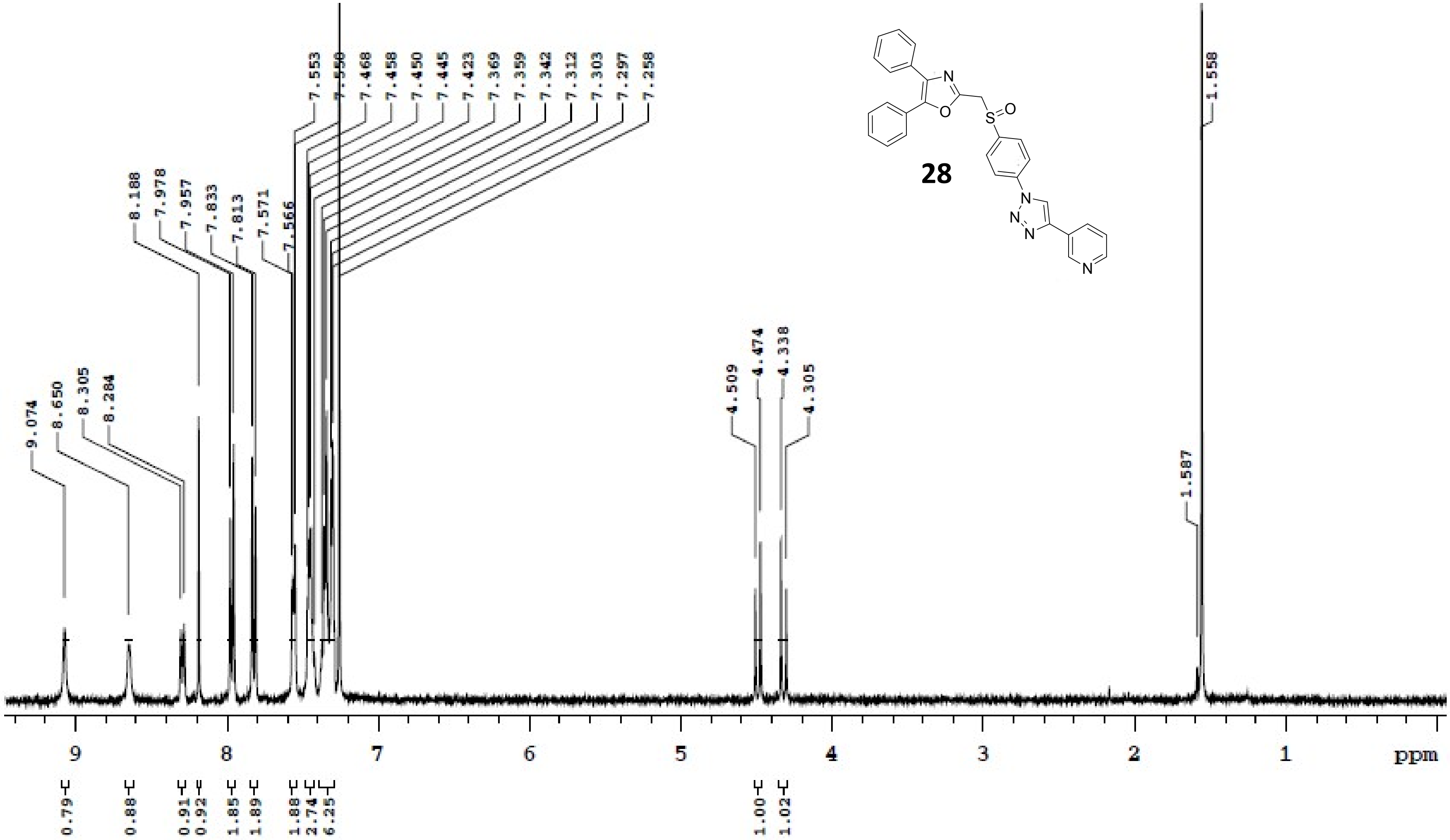

^{13}C NMR: 4,5-diphenyl-2-(((4-(4-(pyridin-3-yl)-1H-1,2,3-triazol-1-yl)phenyl)sulfinyl)methyl)oxazole

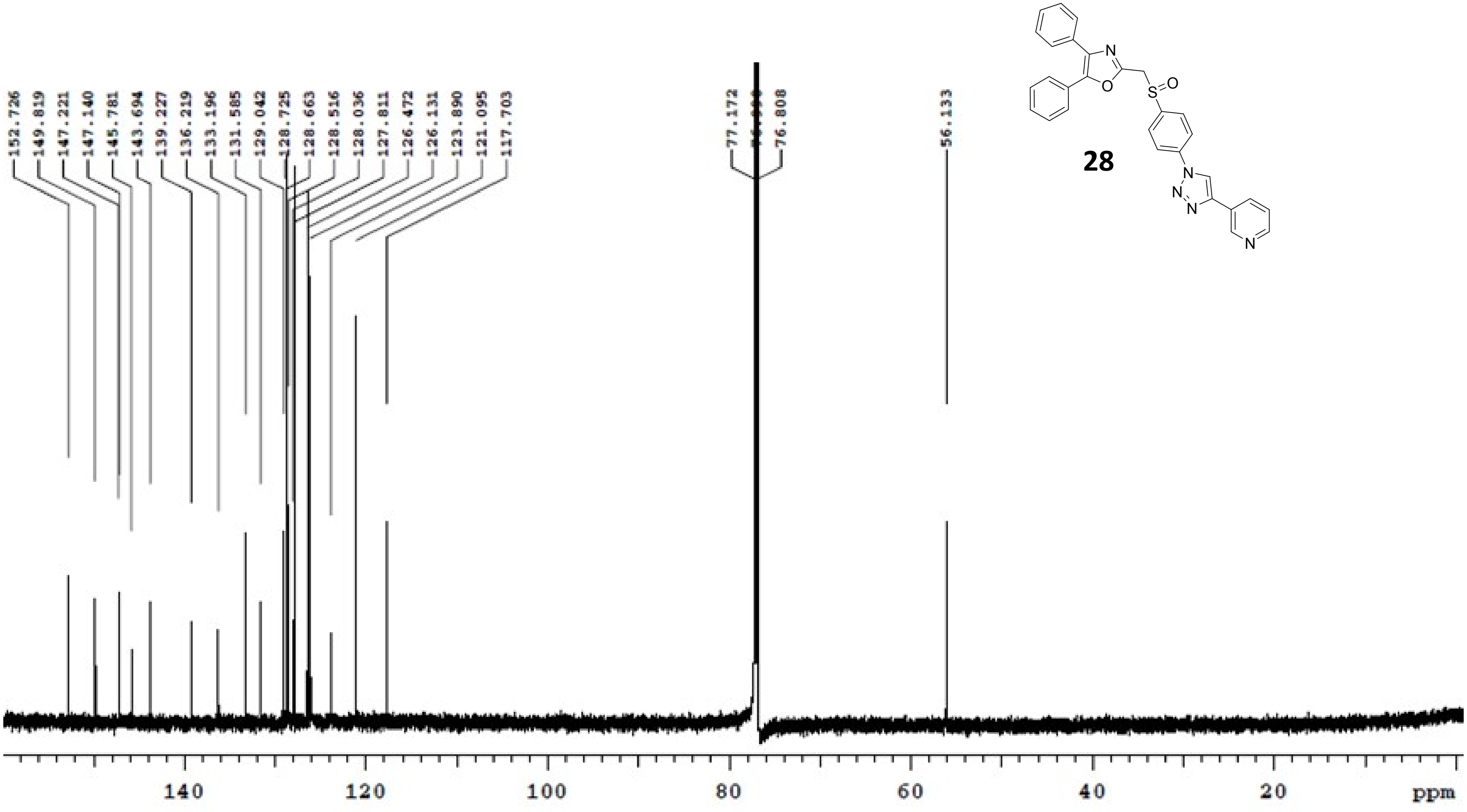

28

^{1}H NMR: 2-(((4-(4-(4-fluorophenyl)-1H-1,2,3-triazol-1-yl)phenyl)sulfinyl)methyl)-4,5-diphenyloxazole

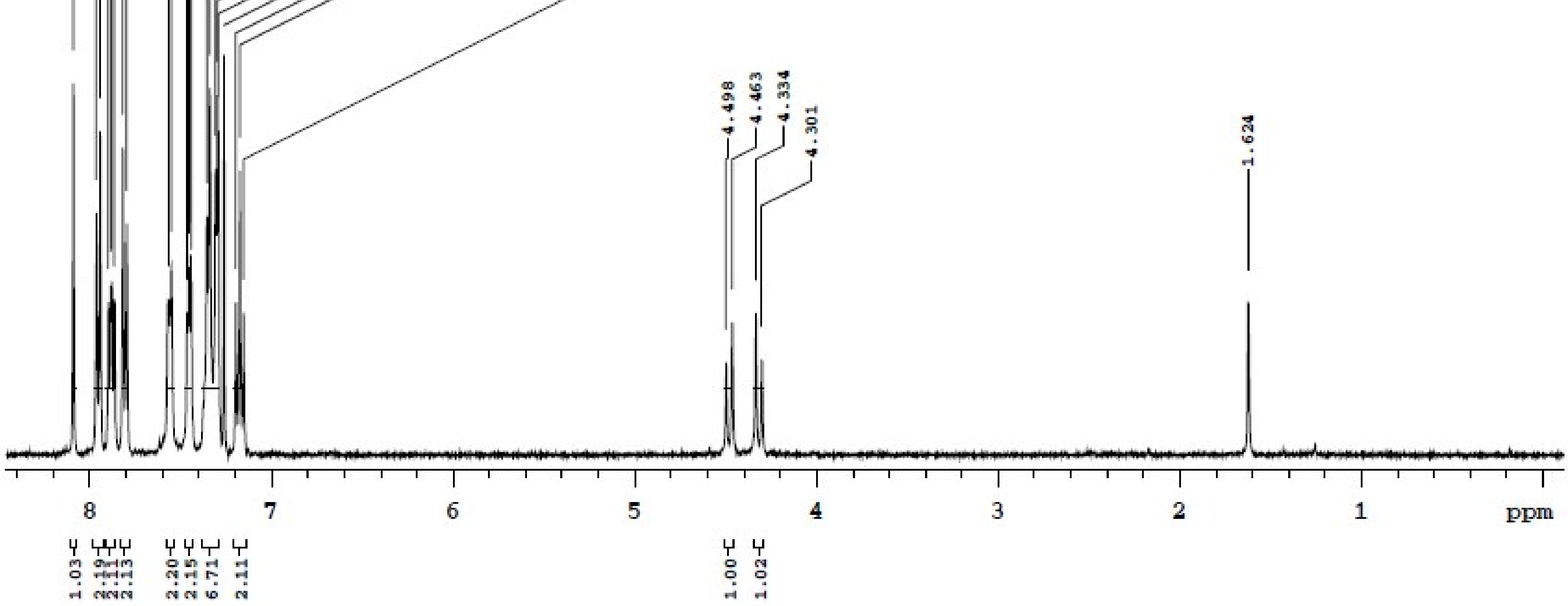

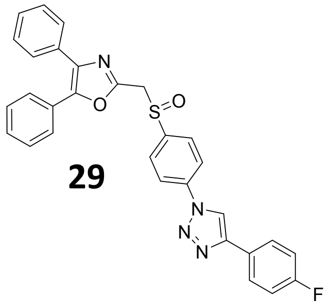

29

^{13}C NMR: 2-(((4-(4-(4-fluorophenyl)-1H-1,2,3-triazol-1-yl)phenyl)sulfinyl)methyl)-4,5-diphenyloxazole

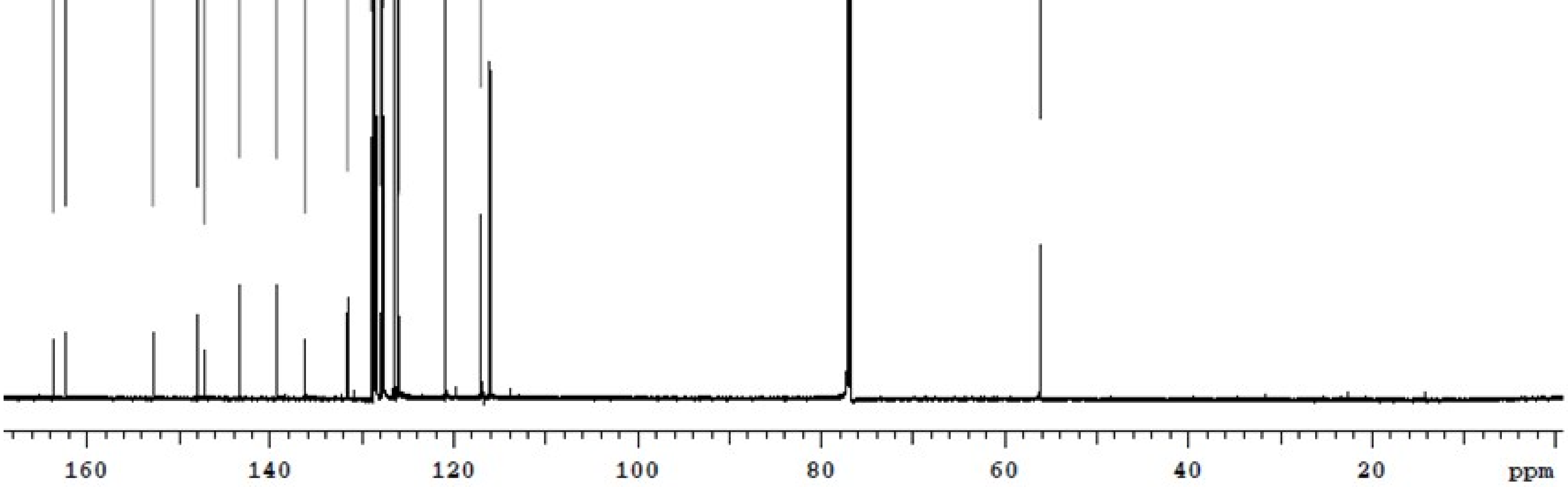

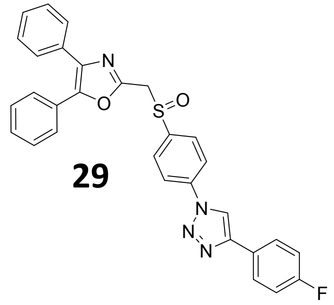

29

^{1}H NMR: 2-(((4-(4-(3-fluorophenyl)-1H-1,2,3-triazol-1-yl)phenyl)sulfinyl)methyl)-4,5-diphenyloxazole

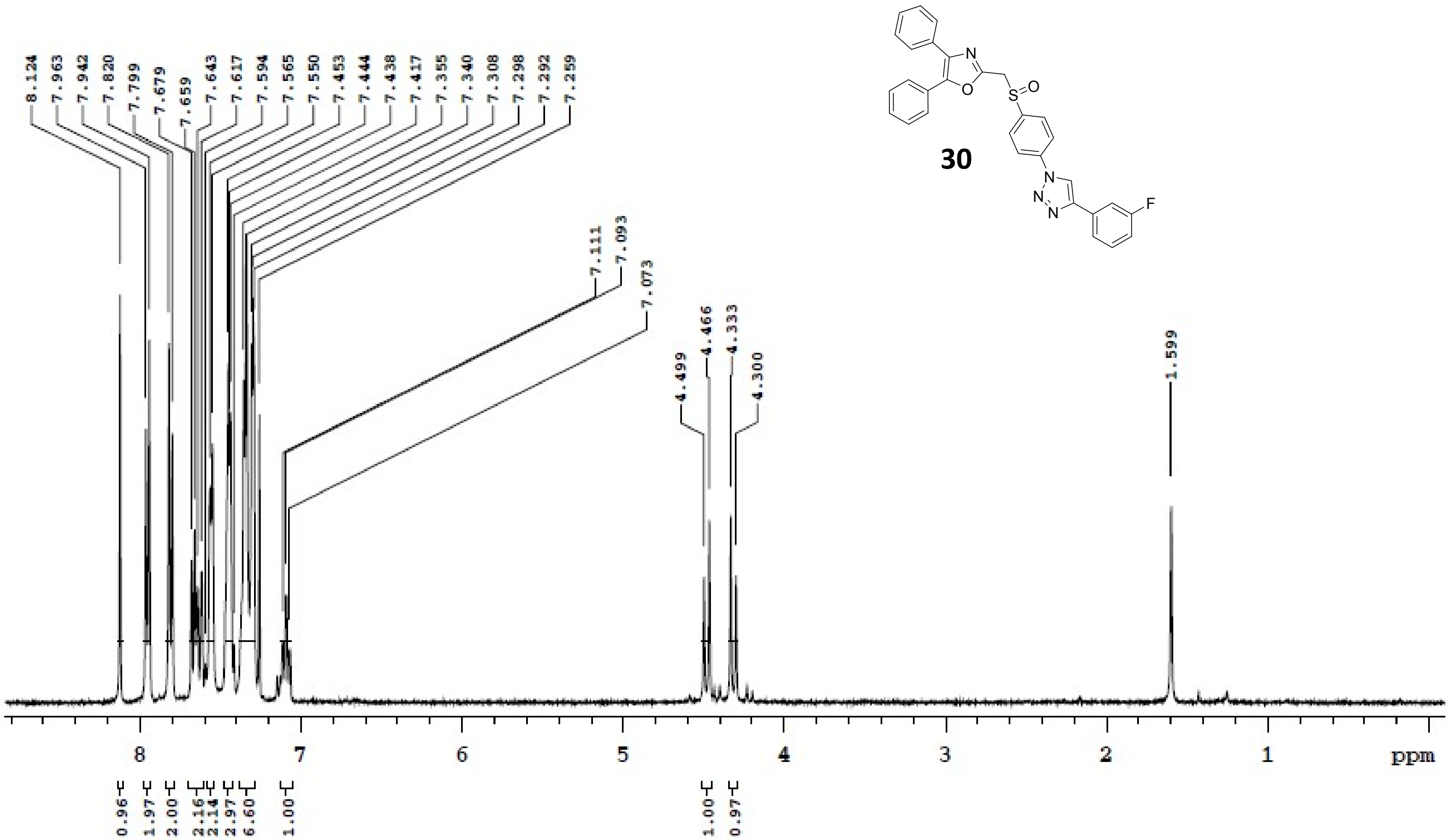

^{13}C NMR: 2-(((4-(4-(3-fluorophenyl)-1H-1,2,3-triazol-1-yl)phenyl)sulfinyl)methyl)-4,5-diphenyloxazole

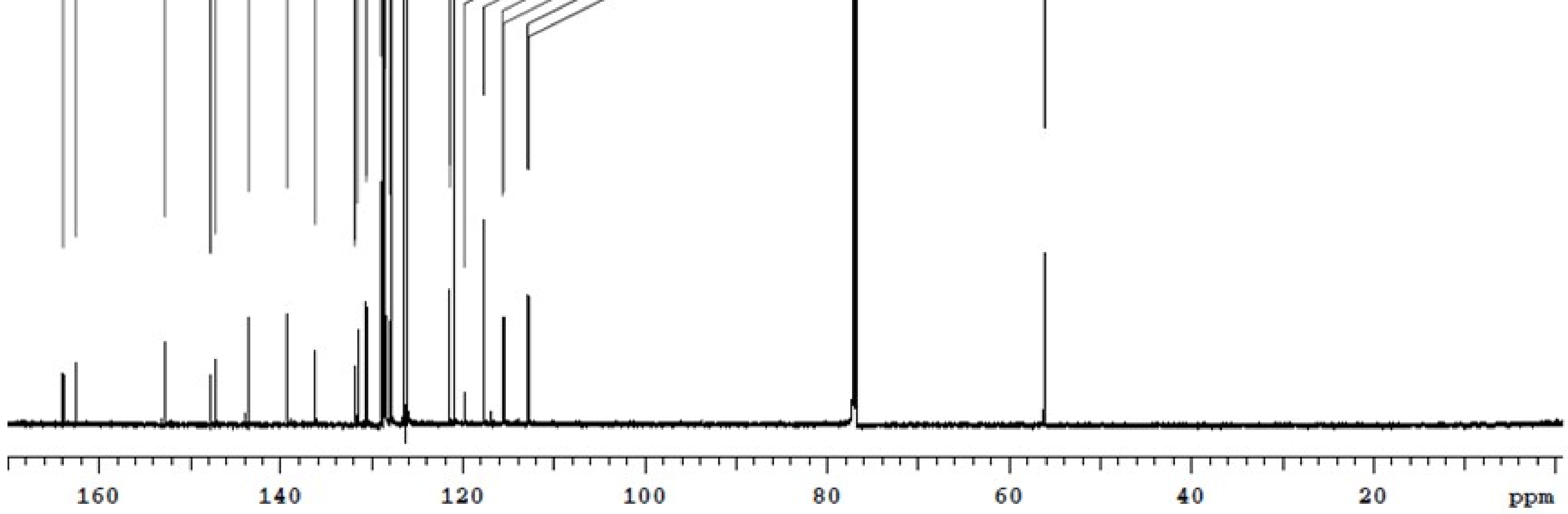

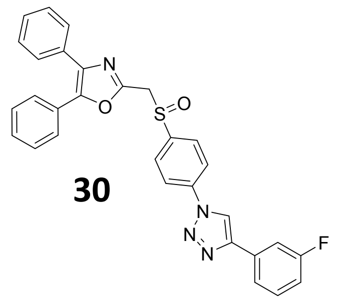

30

^{1}H NMR: 2-(((4-(4-(2-methoxyphenyl)-1H-1,2,3-triazol-1-yl)phenyl)sulfinyl)methyl)-4,5-diphenyloxazole

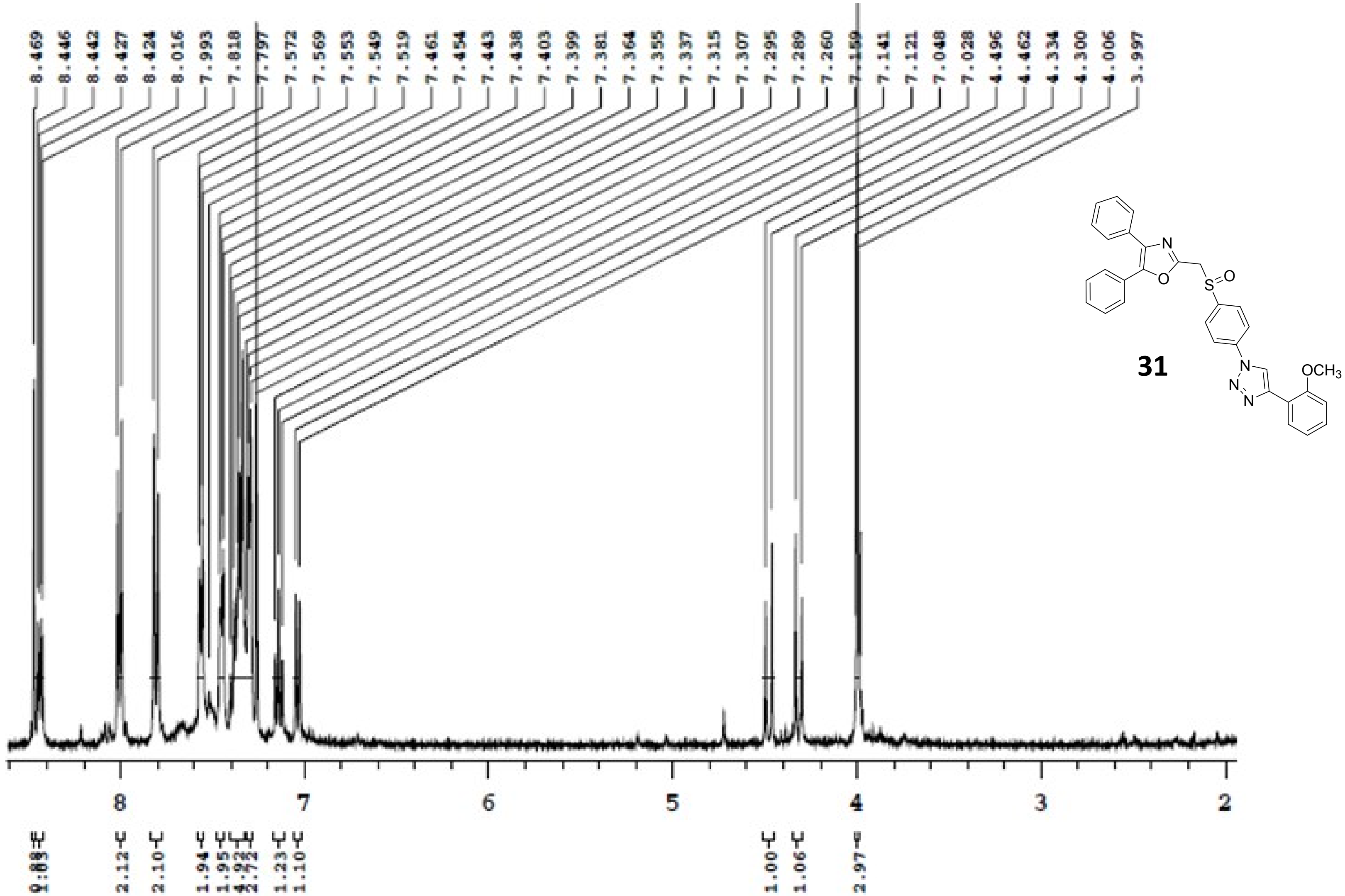

31

^{13}C NMR: 2-(((4-(4-(2-methoxyphenyl)-1H-1,2,3-triazol-1-yl)phenyl)sulfinyl)methyl)-4,5-diphenyloxazole

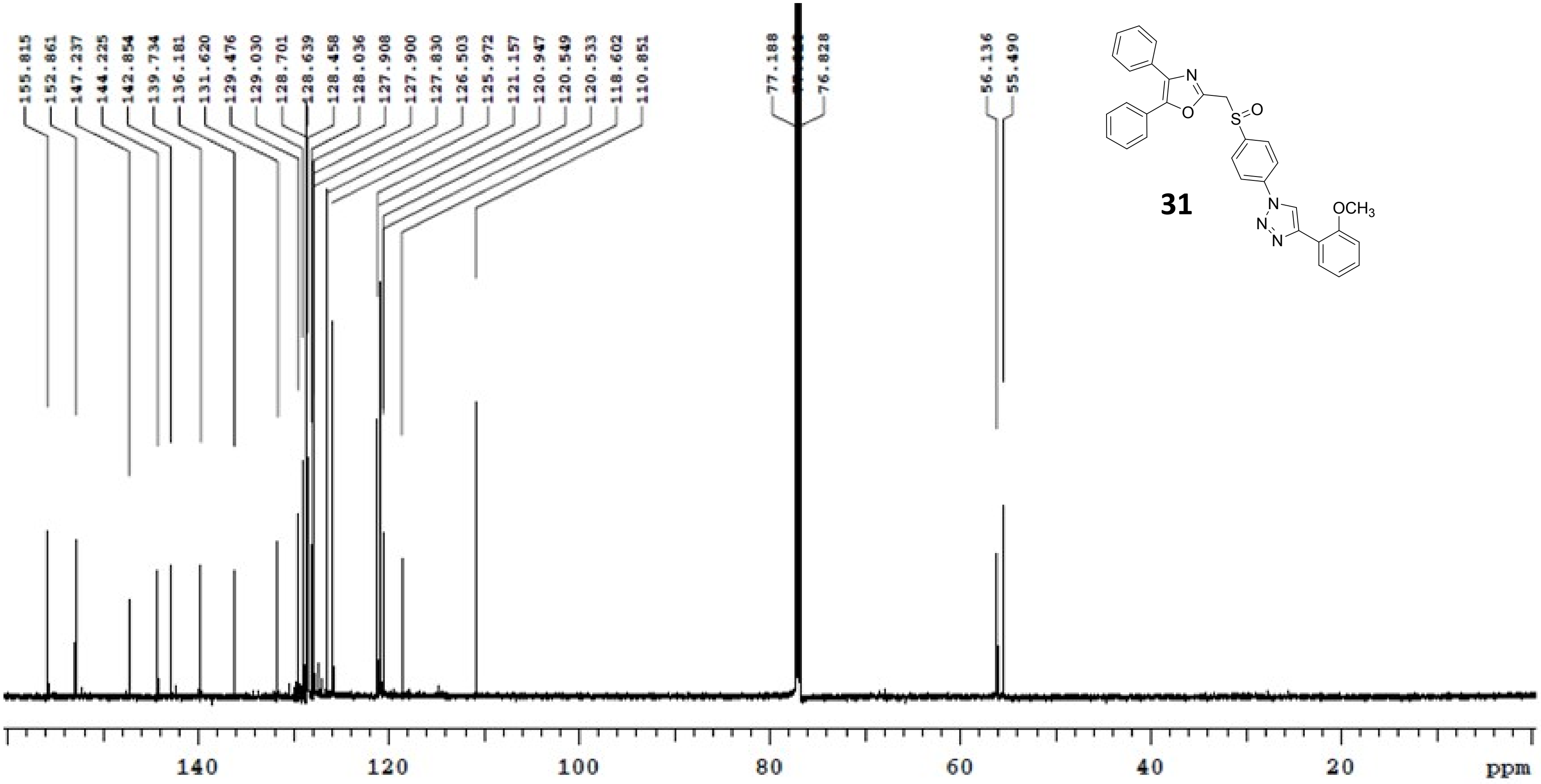

31

^{1}H NMR: 4,5-diphenyl-2-(((4-(4-(2-(trifluoromethyl)phenyl)-1H-1,2,3-triazol-1-yl)phenyl)sulfinyl)methyl)oxazole

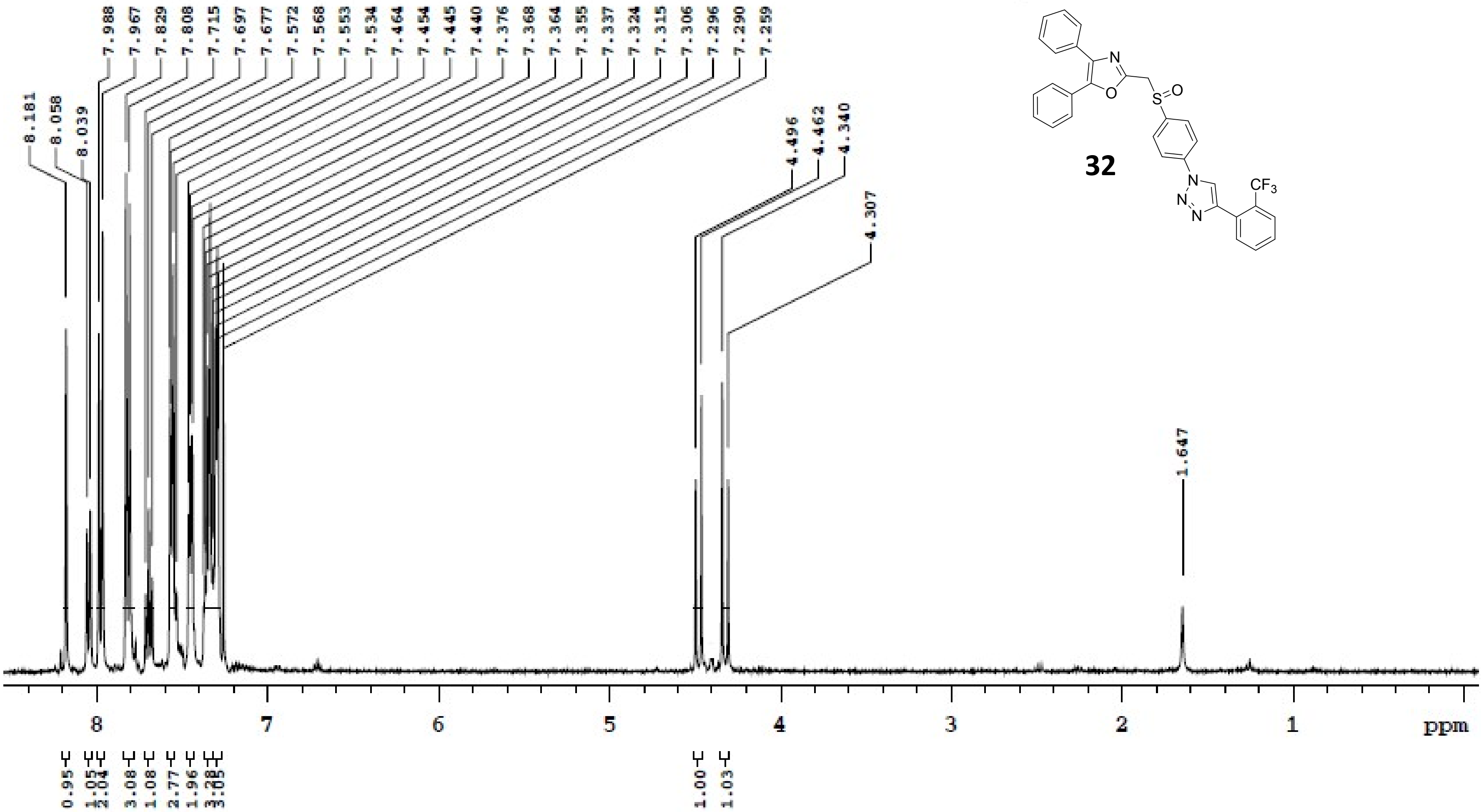

32

^{13}C NMR: 4,5-diphenyl-2-(((4-(4-(2-(trifluoromethyl)phenyl)-1H-1,2,3-triazol-1-yl)phenyl)sulfinyl)methyl)oxazole

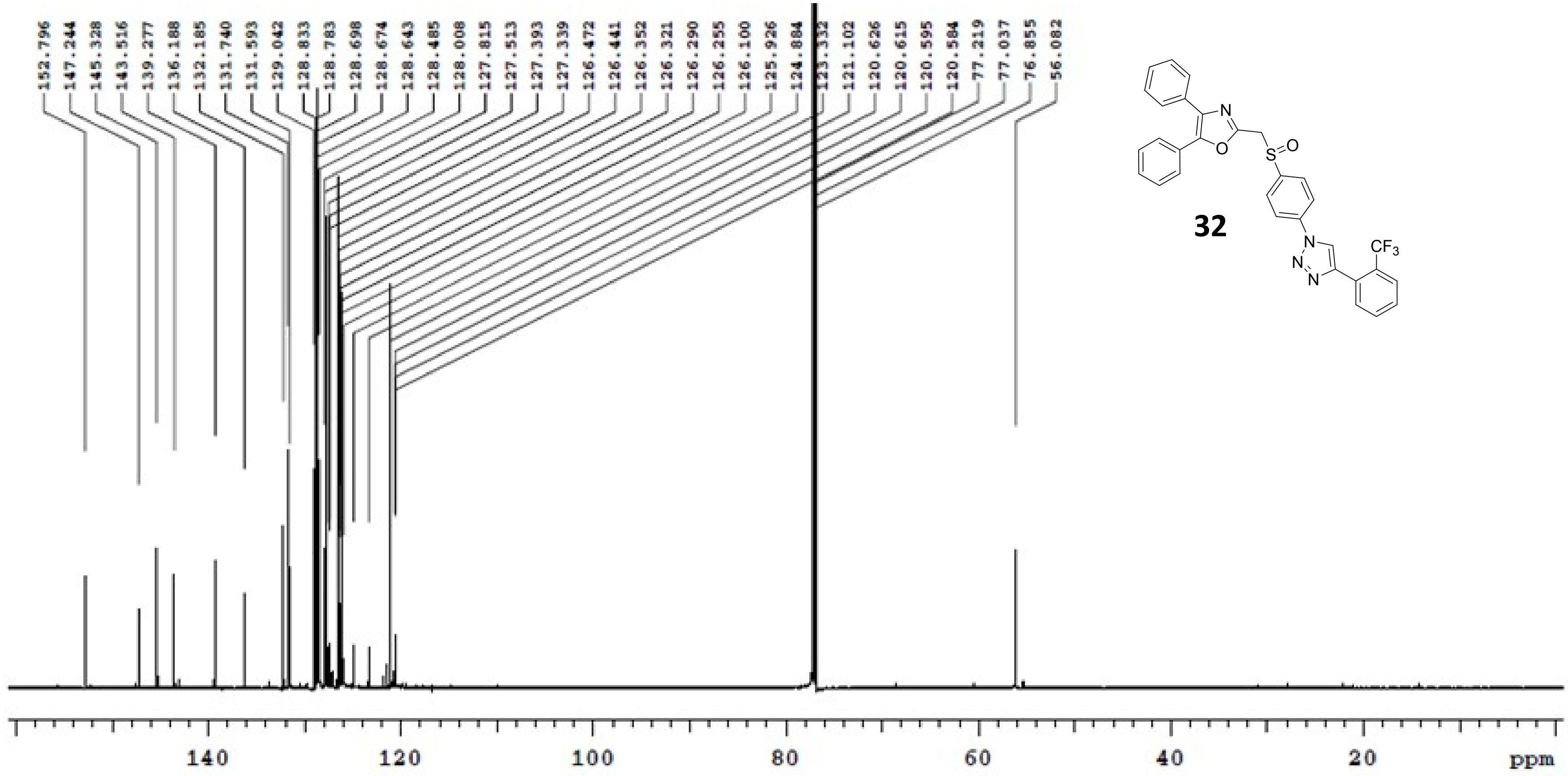

^{1}H NMR: 2-(((4-(4-(6-methoxynaphthalen-2-yl)-1H-1,2,3-triazol-1-yl)phenyl)sulfinyl)methyl)-4,5-diphenyloxazole

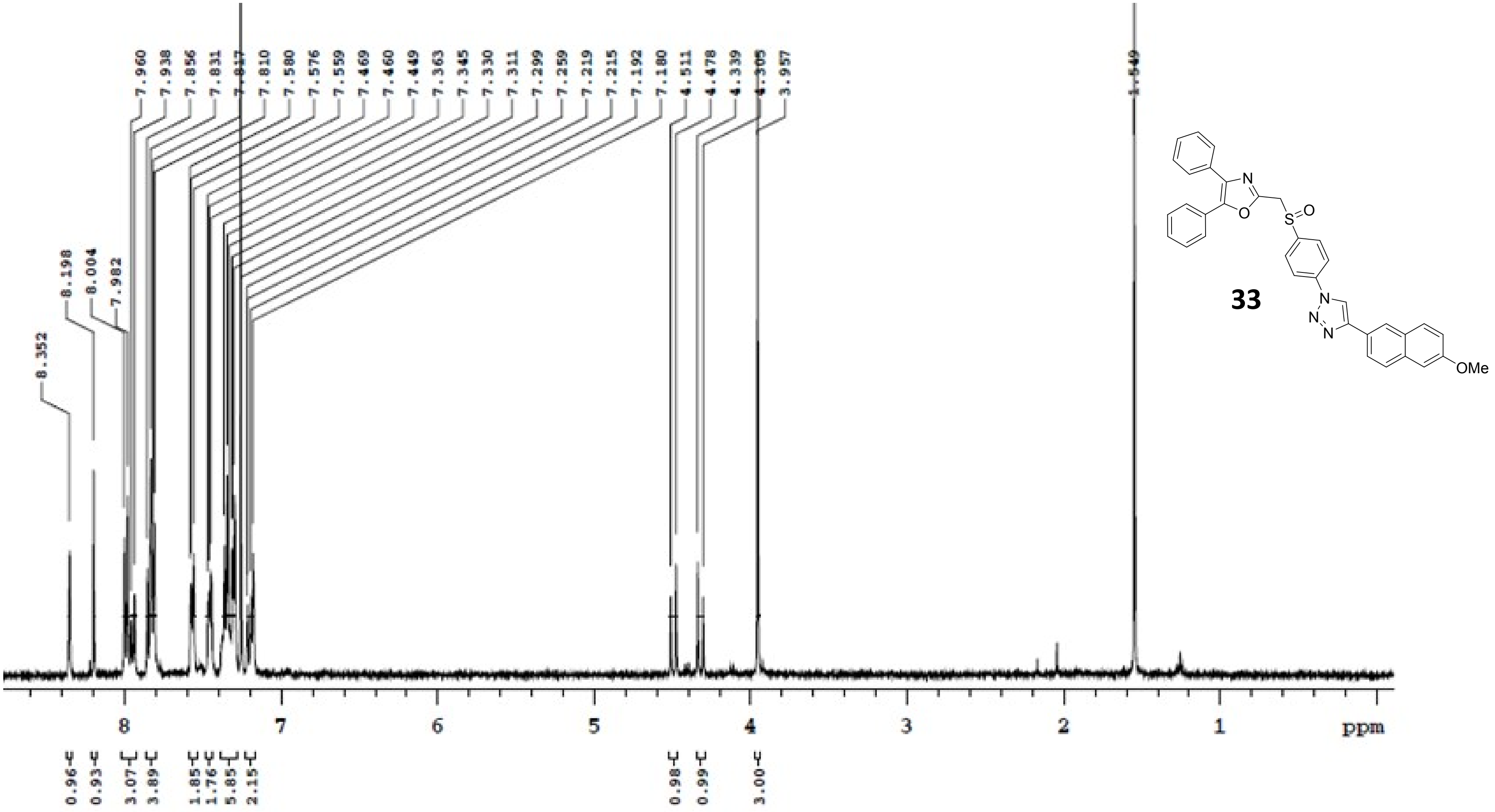

^{13}C NMR: 2-(((4-(4-(6-methoxynaphthalen-2-yl)-1H-1,2,3-triazol-1-yl)phenyl)sulfinyl)methyl)-4,5-diphenyloxazole

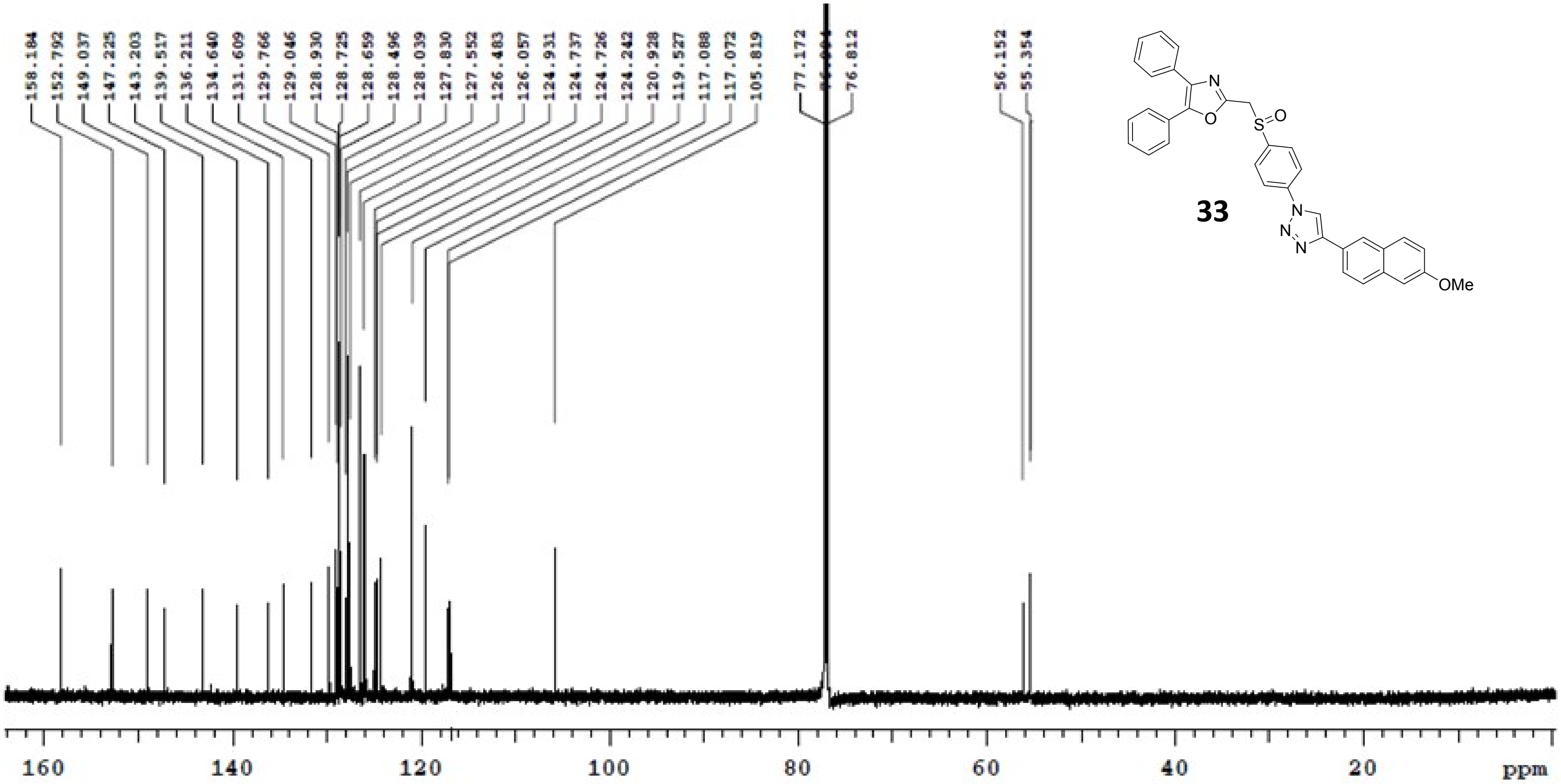

¹H NMR: 2-(((4-(4-(4-pentylphenyl)-1H-1,2,3-triazol-1-yl)phenyl)sulfinyl)methyl)-4,5-diphenyloxazole

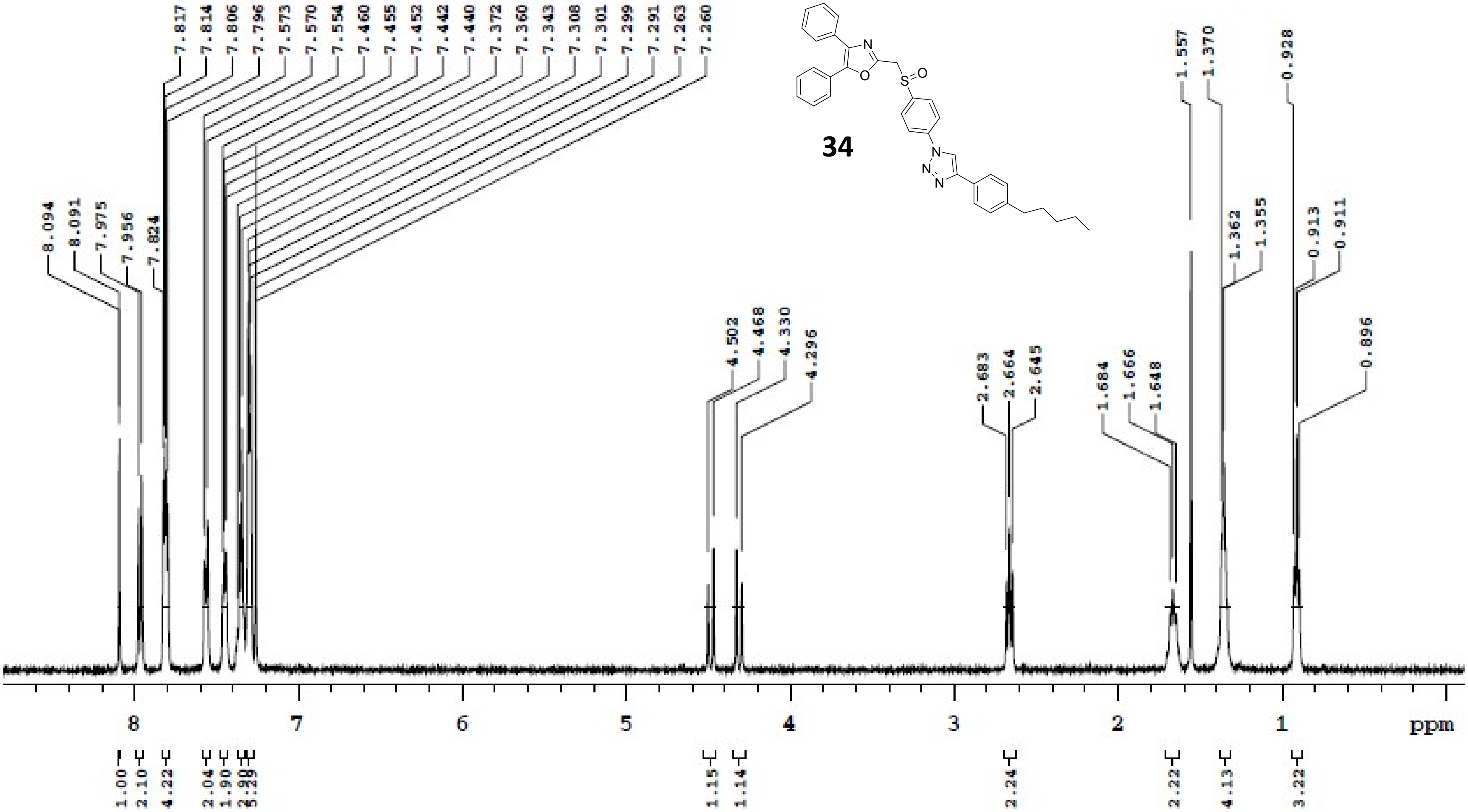

^{13}C NMR: 2-(((4-(4-(4-pentylphenyl)-1H-1,2,3-triazol-1-yl)phenyl)sulfinyl)methyl)-4,5-diphenyloxazole

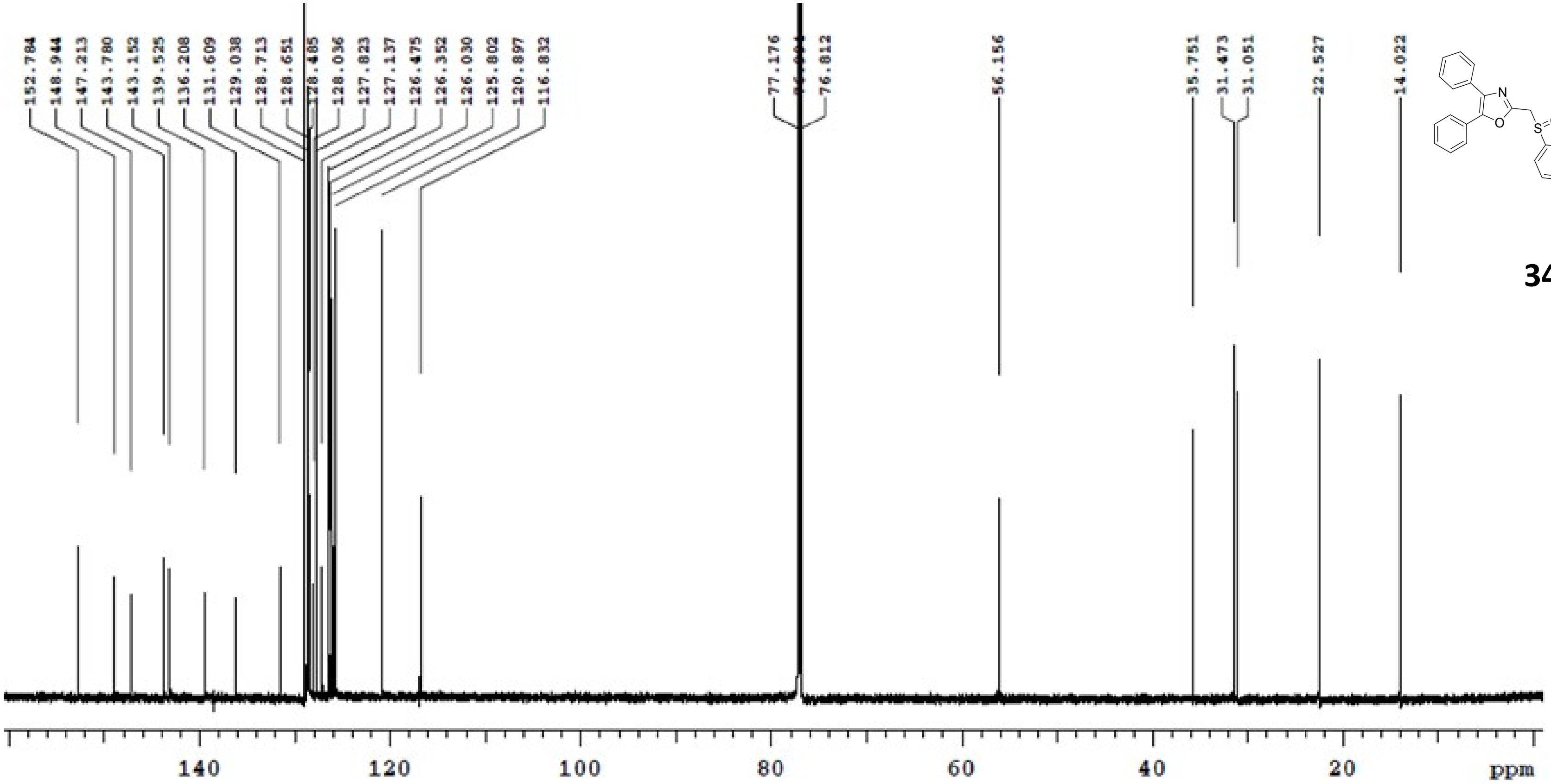

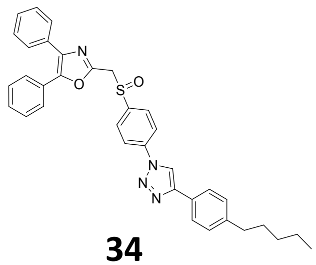

34

^{1}H NMR: 2-(((4-(4-(4-phenoxyphenyl)-1H-1,2,3-triazol-1-yl)phenyl)sulfinyl)methyl)-4,5-diphenyloxazole

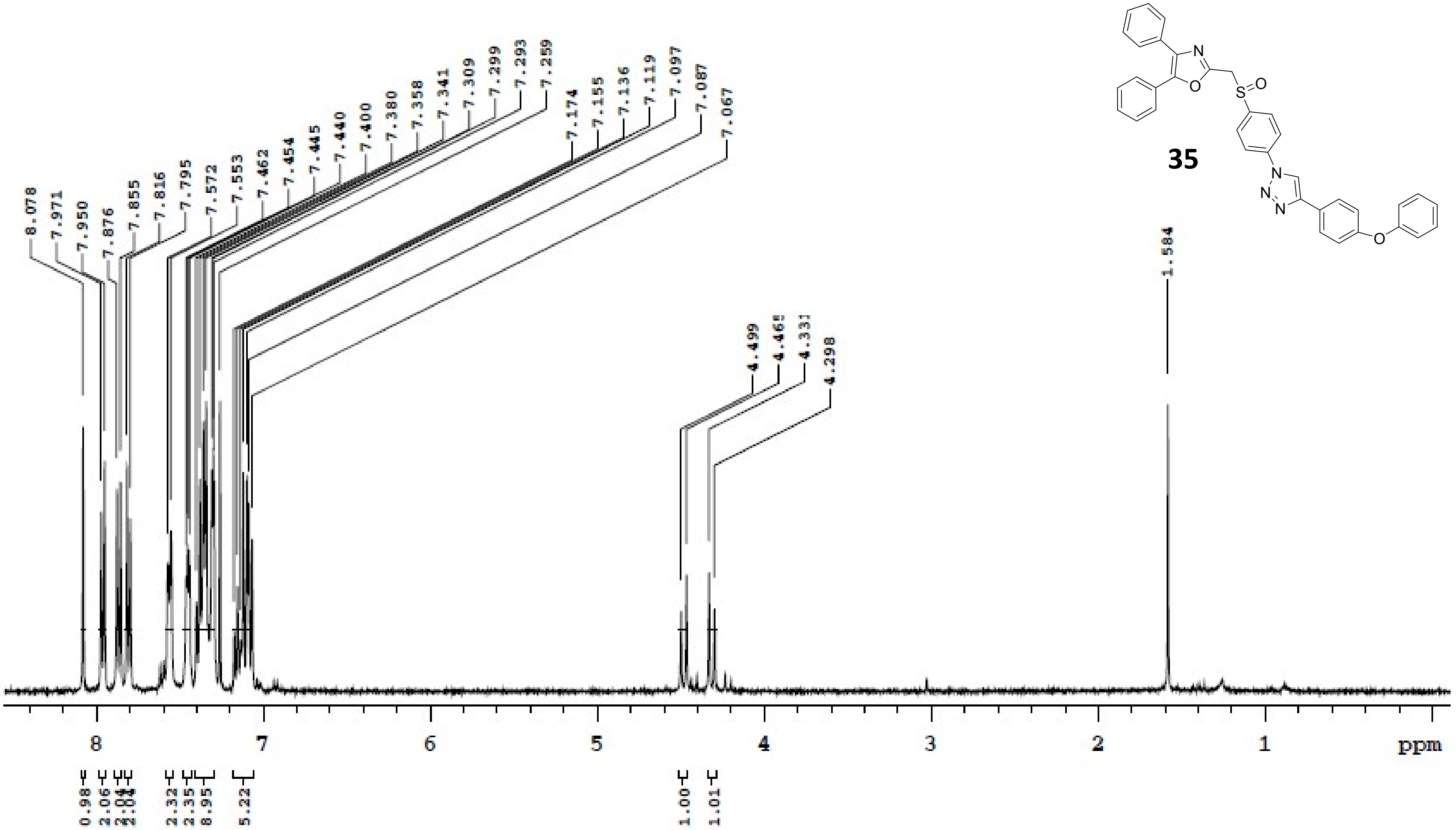

35

^{13}C NMR: 2-(((4-(4-(4-phenoxyphenyl)-1H-1,2,3-triazol-1-yl)phenyl)sulfinyl)methyl)-4,5-diphenyloxazole

35

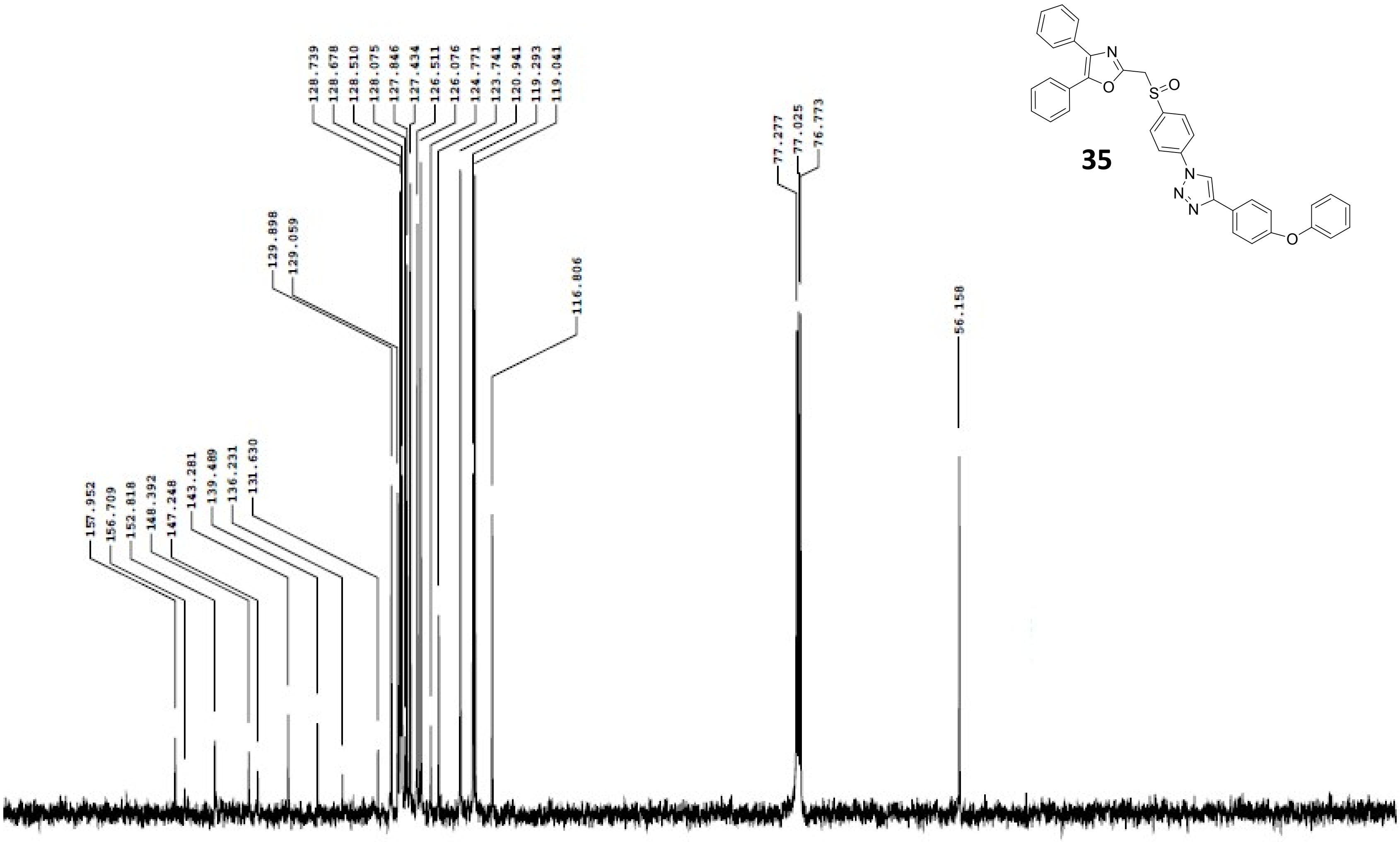

^{1}H NMR: 2-(((4-(4-(3,5-bis(trifluoromethyl)phenyl)-1H-1,2,3-triazol-1-yl)phenyl)sulfinyl)methyl)-4,5-diphenyloxazole

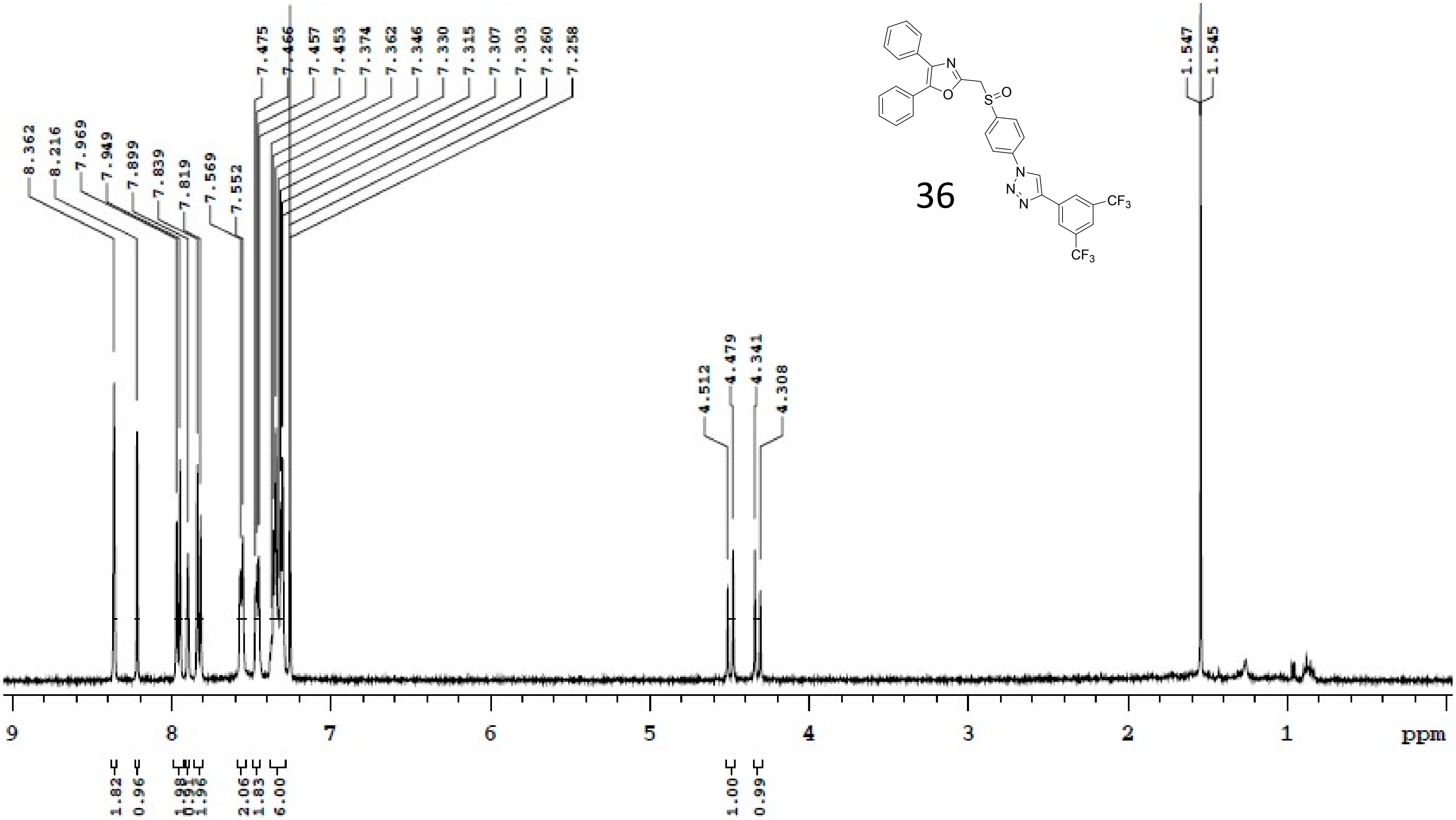

^{13}C NMR: 2-(((4-(4-(3,5-bis(trifluoromethyl)phenyl)-1H-1,2,3-triazol-1-yl)phenyl)sulfinyl)methyl)-4,5-diphenyloxazole

36

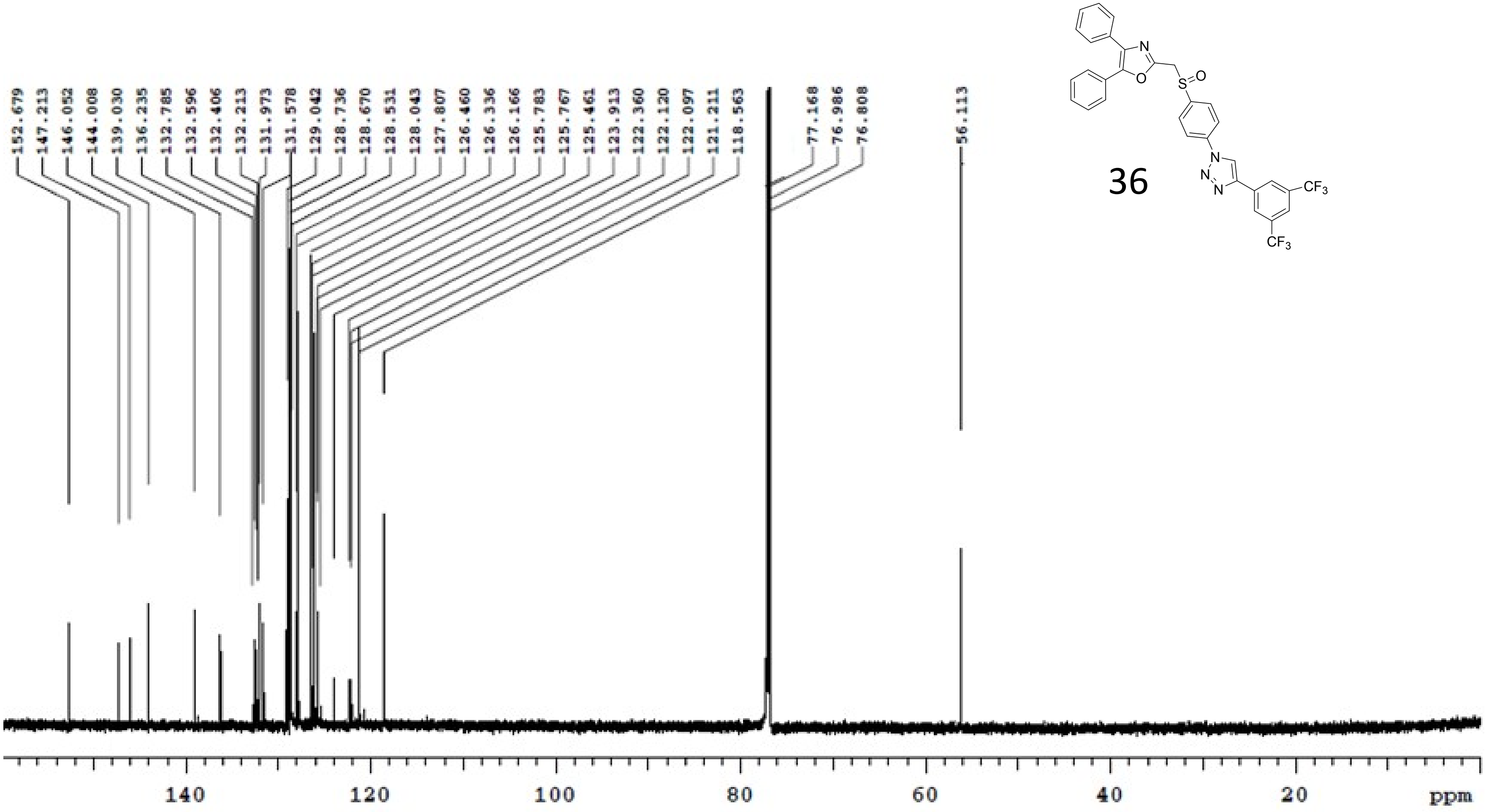

^{1}H NMR: 4,5-diphenyl-2-(((4-(4-(pyridin-3-yl)-1H-1,2,3-triazol-1-yl)phenyl)sulfonyl)methyl)oxazole

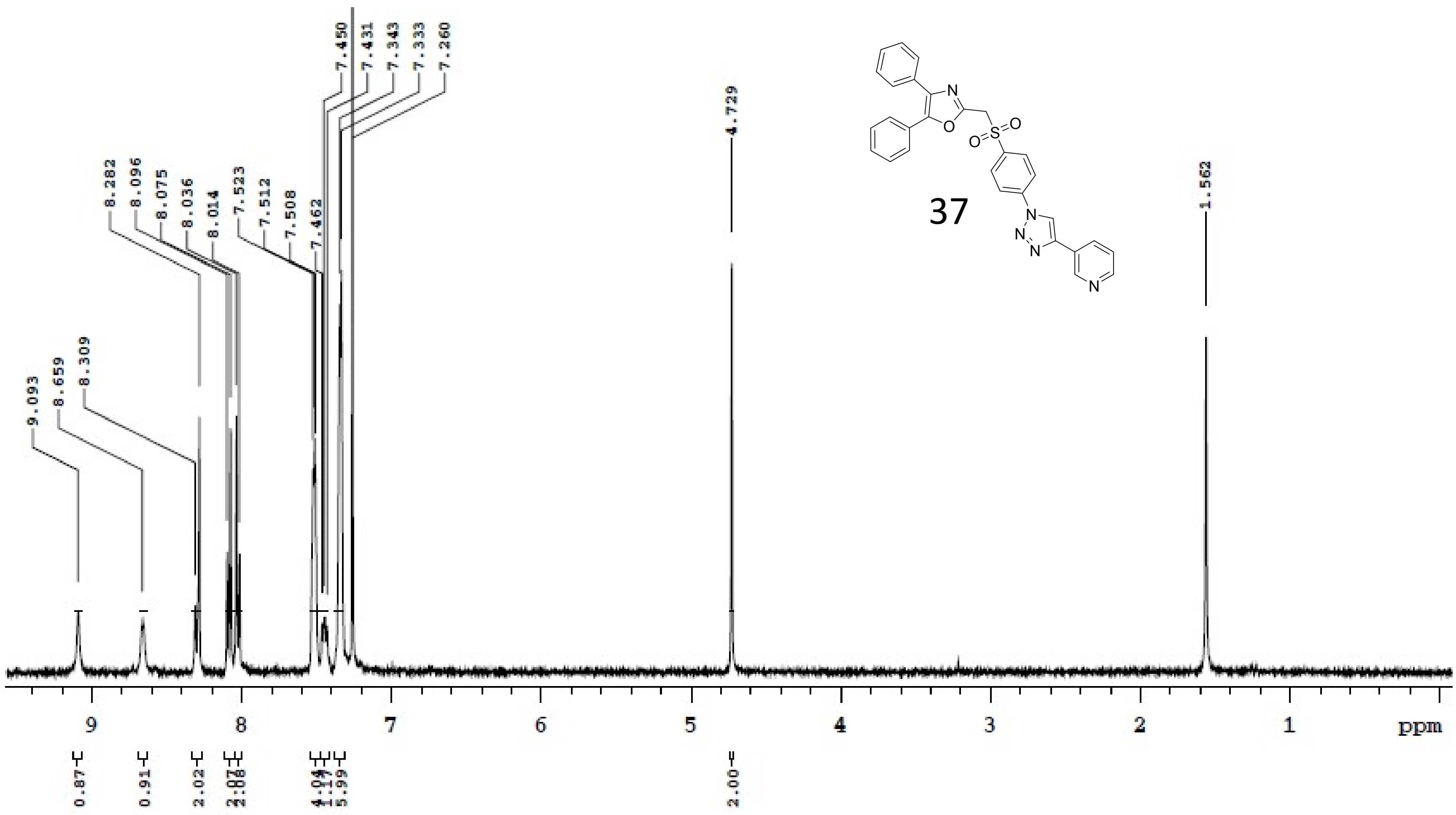

37

^{13}C NMR: 4,5-diphenyl-2-(((4-(4-(pyridin-3-yl)-1H-1,2,3-triazol-1-yl)phenyl)sulfonyl)methyl)oxazole

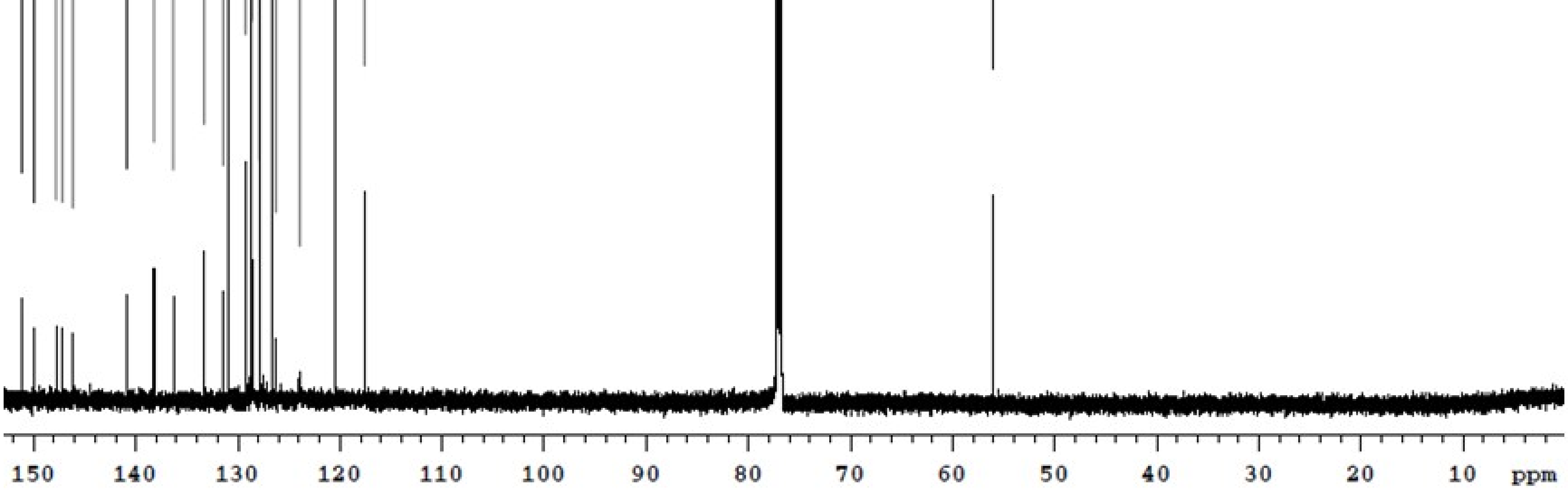

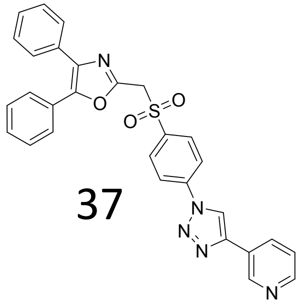

37

^{1}H NMR: 2-(((4-(4-(4-fluorophenyl)-1H-1,2,3-triazol-1-yl)phenyl)sulfonyl)methyl)-4,5-diphenyloxazole

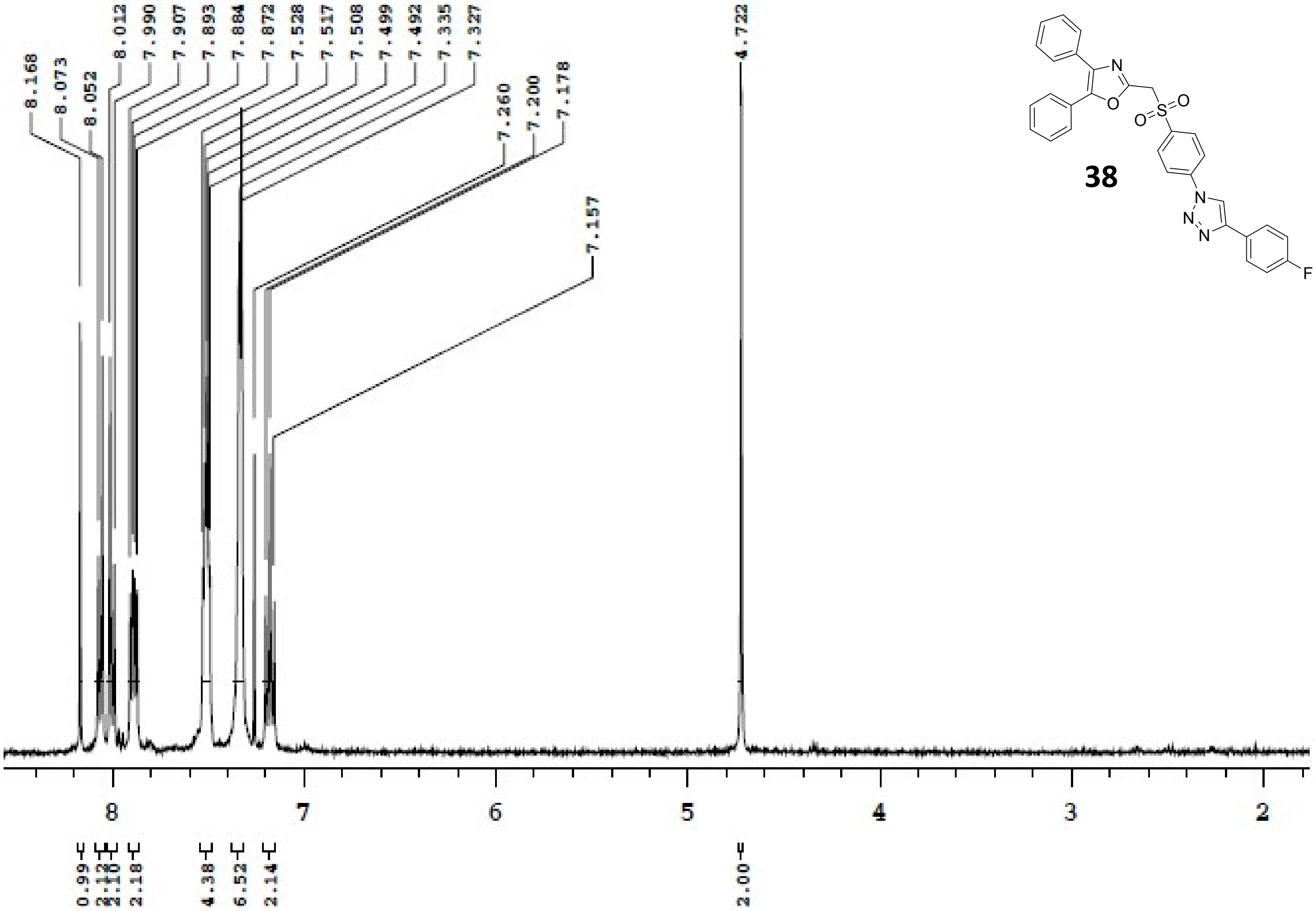

38

^{13}C NMR: 2-(((4-(4-(4-fluorophenyl)-1H-1,2,3-triazol-1-yl)phenyl)sulfonyl)methyl)-4,5-diphenyloxazole

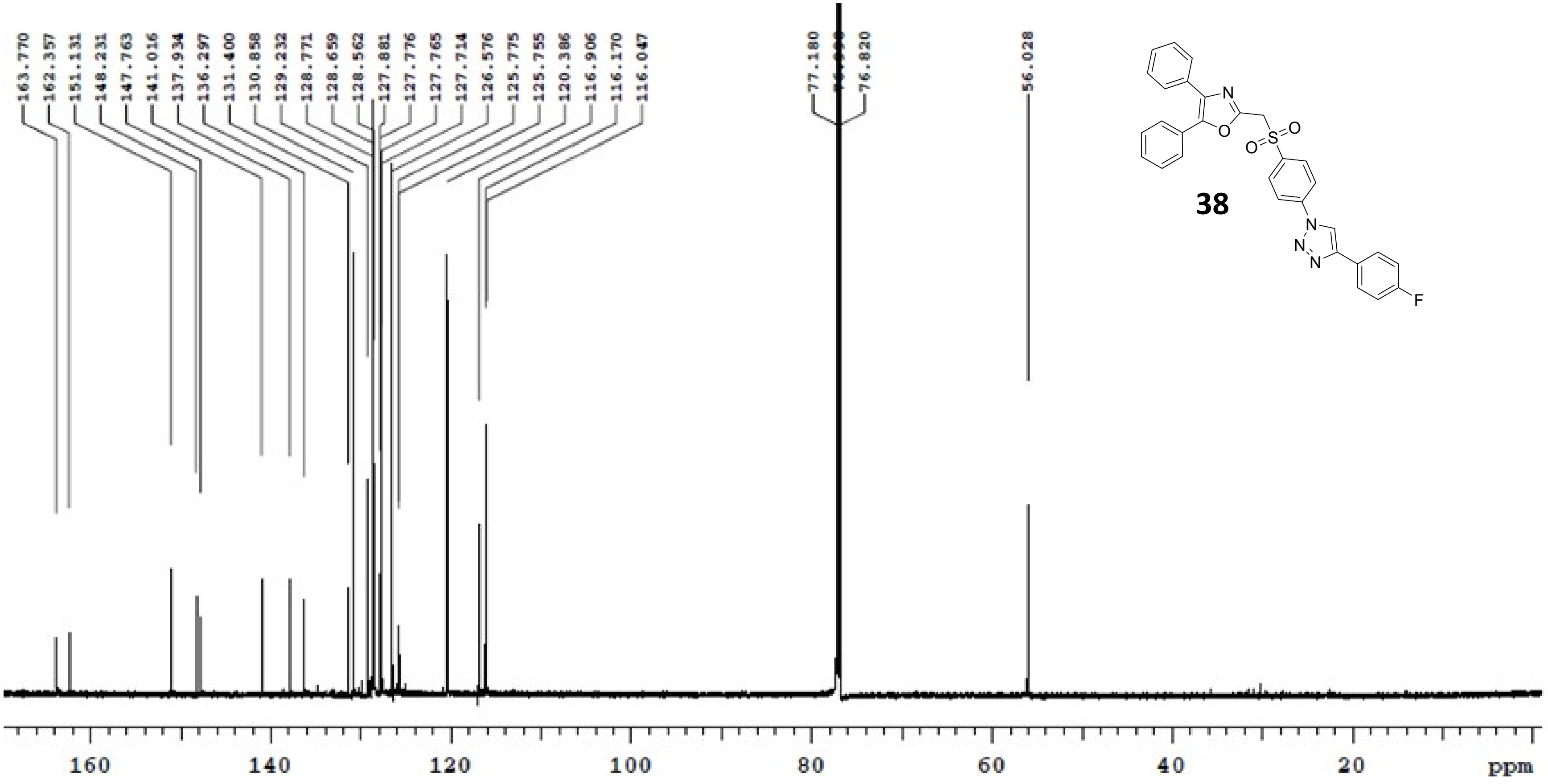

38

^{1}H NMR: 2-(((4-(4-(3-fluorophenyl)-1H-1,2,3-triazol-1-yl)phenyl)sulfonyl)methyl)-4,5-diphenyloxazole

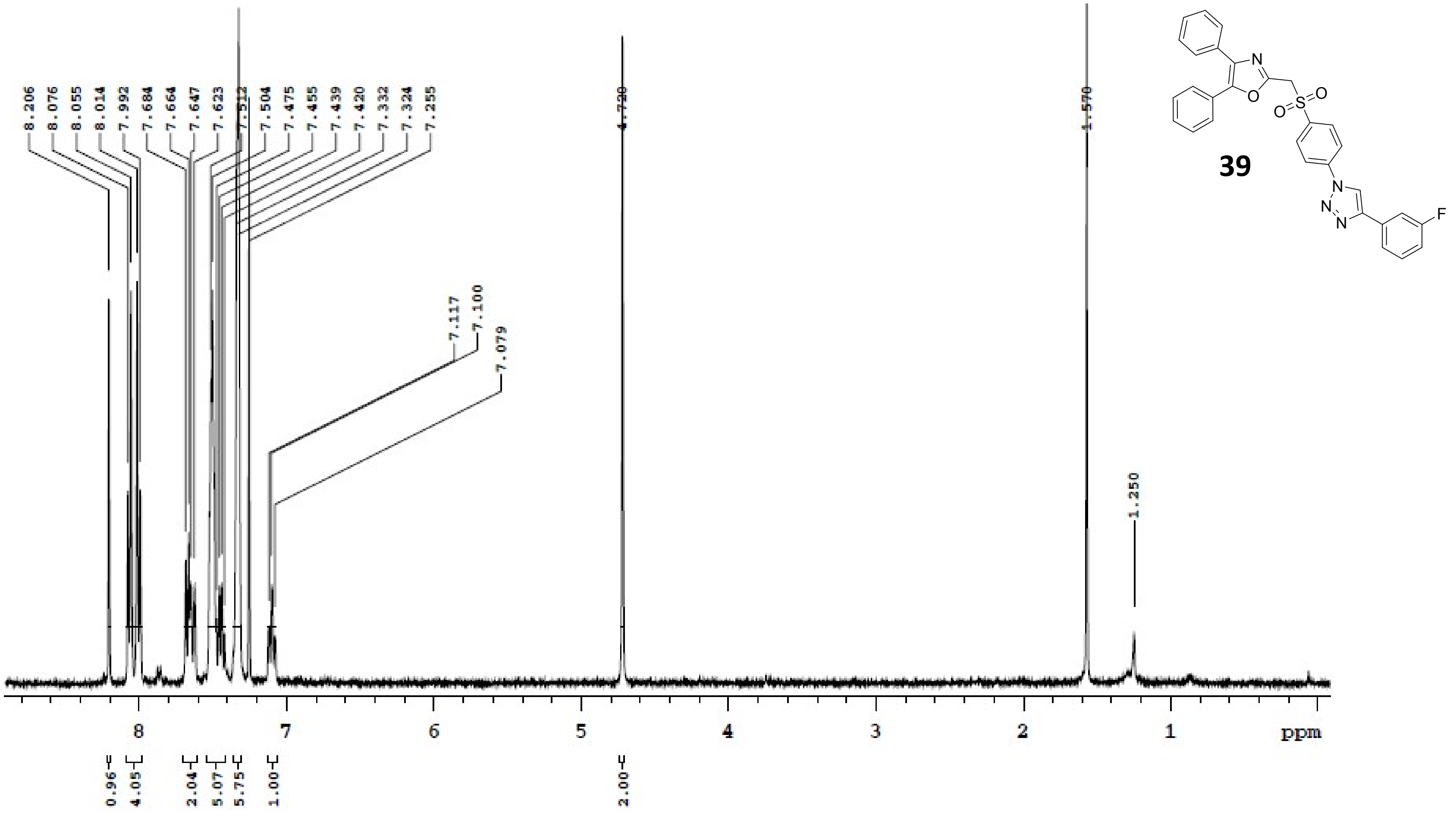

^{13}C NMR: 2-(((4-(4-(3-fluorophenyl)-1H-1,2,3-triazol-1-yl)phenyl)sulfonyl)methyl)-4,5-diphenyloxazole

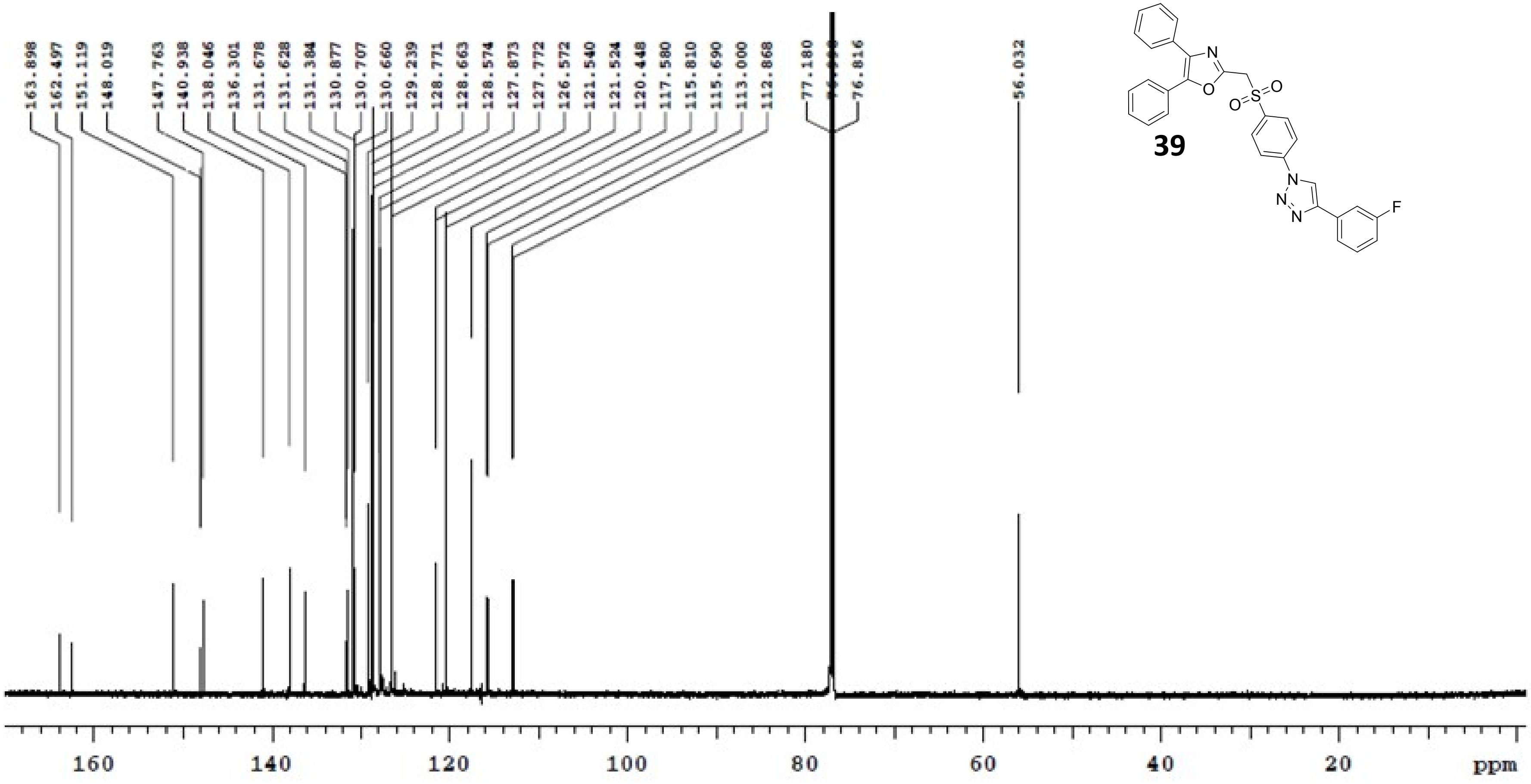

^{1}H NMR: 2-(((4-(4-(2-methoxyphenyl)-1H-1,2,3-triazol-1-yl)phenyl)sulfonyl)methyl)-4,5-diphenyloxazole

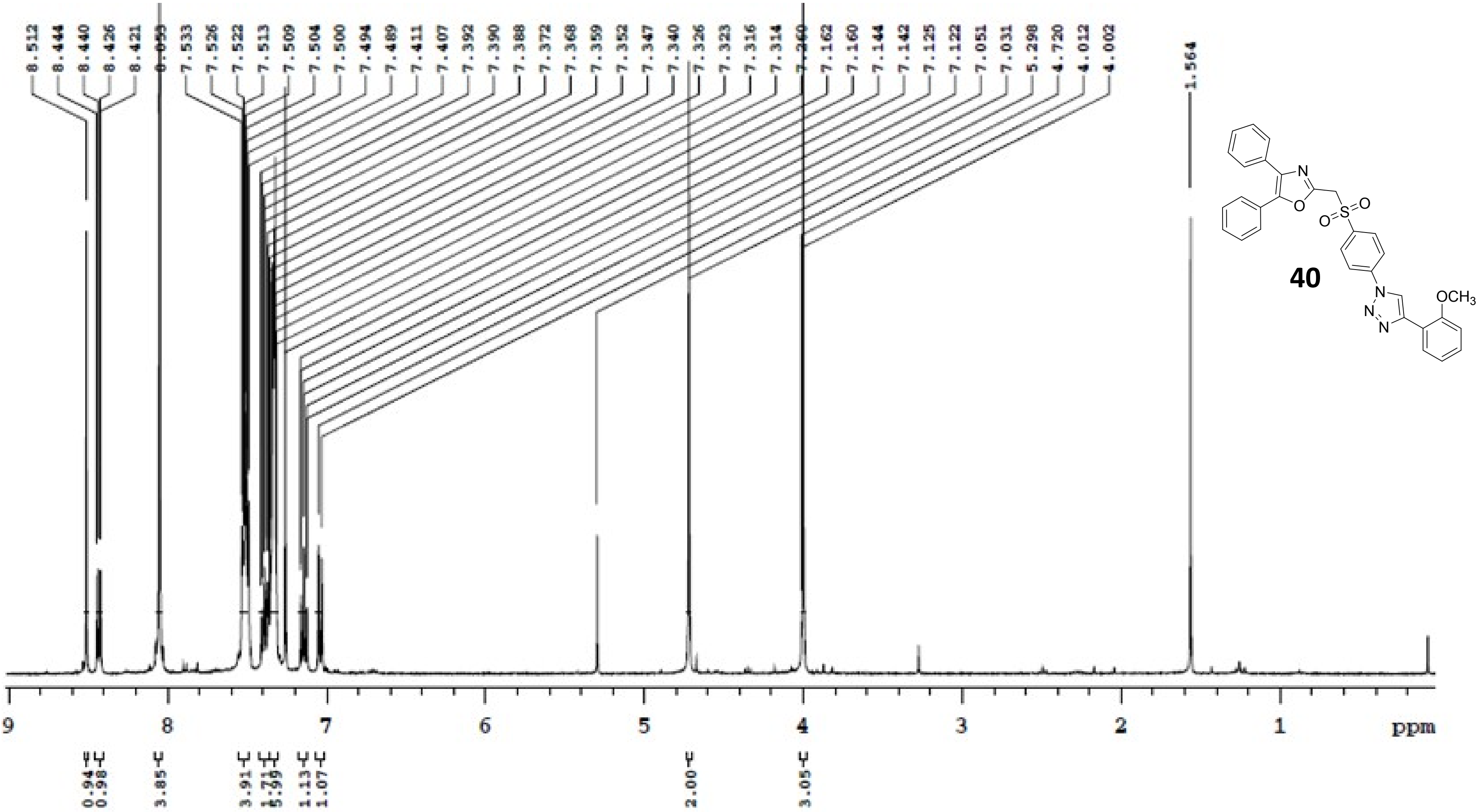

^{13}C NMR: 2-(((4-(4-(2-methoxyphenyl)-1H-1,2,3-triazol-1-yl)phenyl)sulfonyl)methyl)-4,5-diphenyloxazole

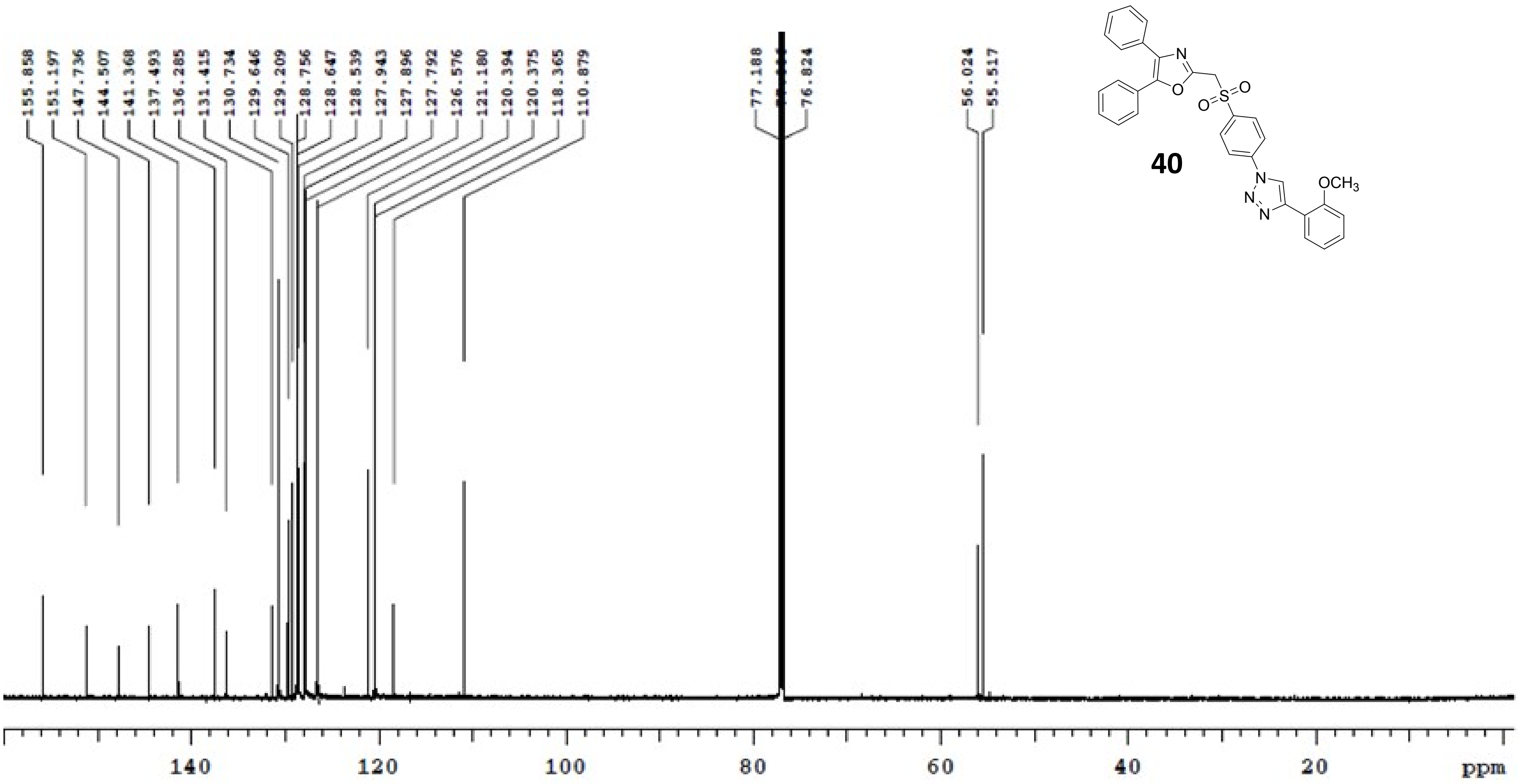

^{1}H NMR: 4,5-diphenyl-2-(((4-(4-(2-(trifluoromethyl)phenyl)-1H-1,2,3-triazol-1-yl)phenyl)sulfonyl)methyl)oxazole

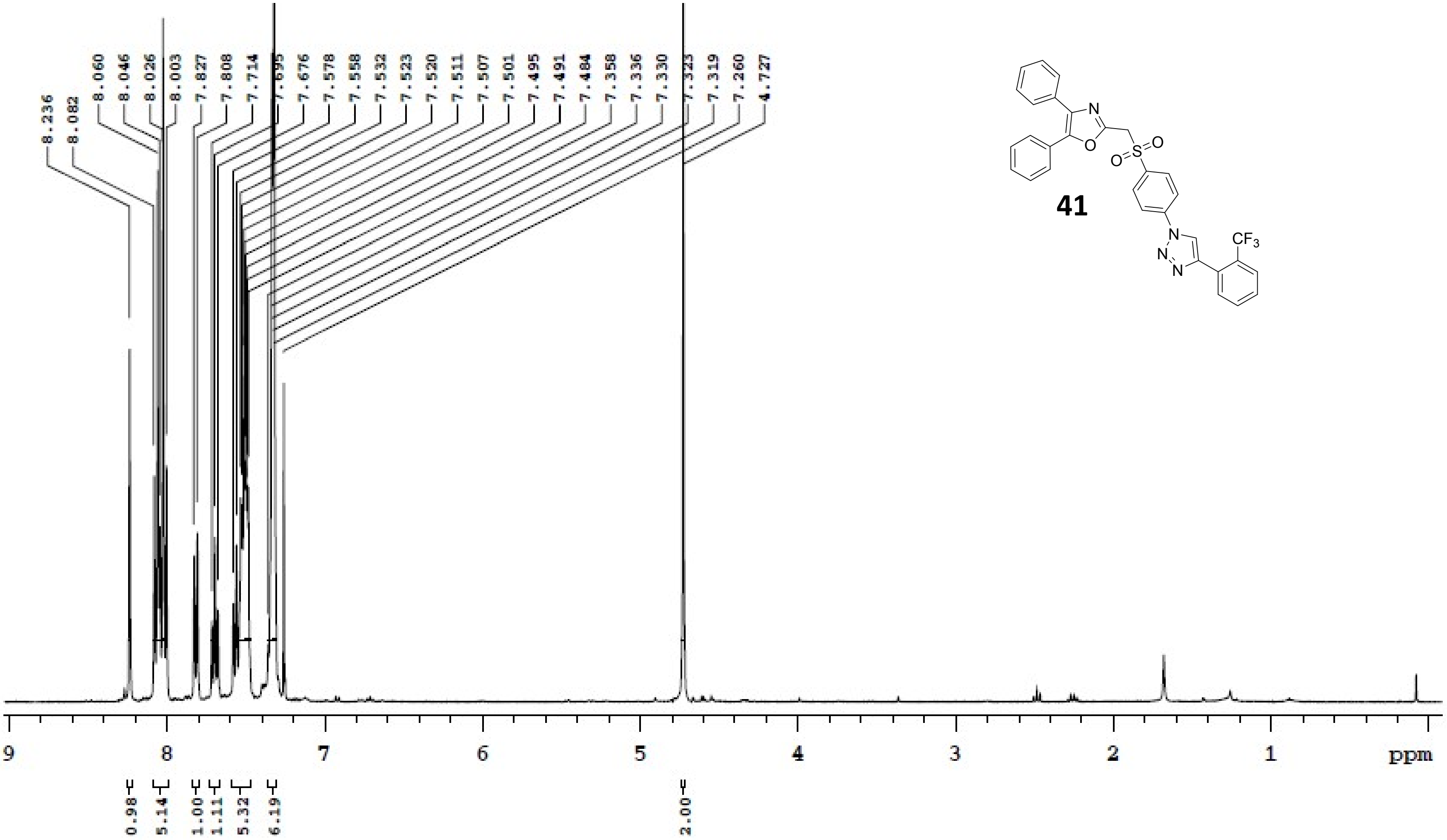

41

^{13}C NMR: 4,5-diphenyl-2-(((4-(4-(2-(trifluoromethyl)phenyl)-1H-1,2,3-triazol-1-yl)phenyl)sulfonyl)methyl)oxazole

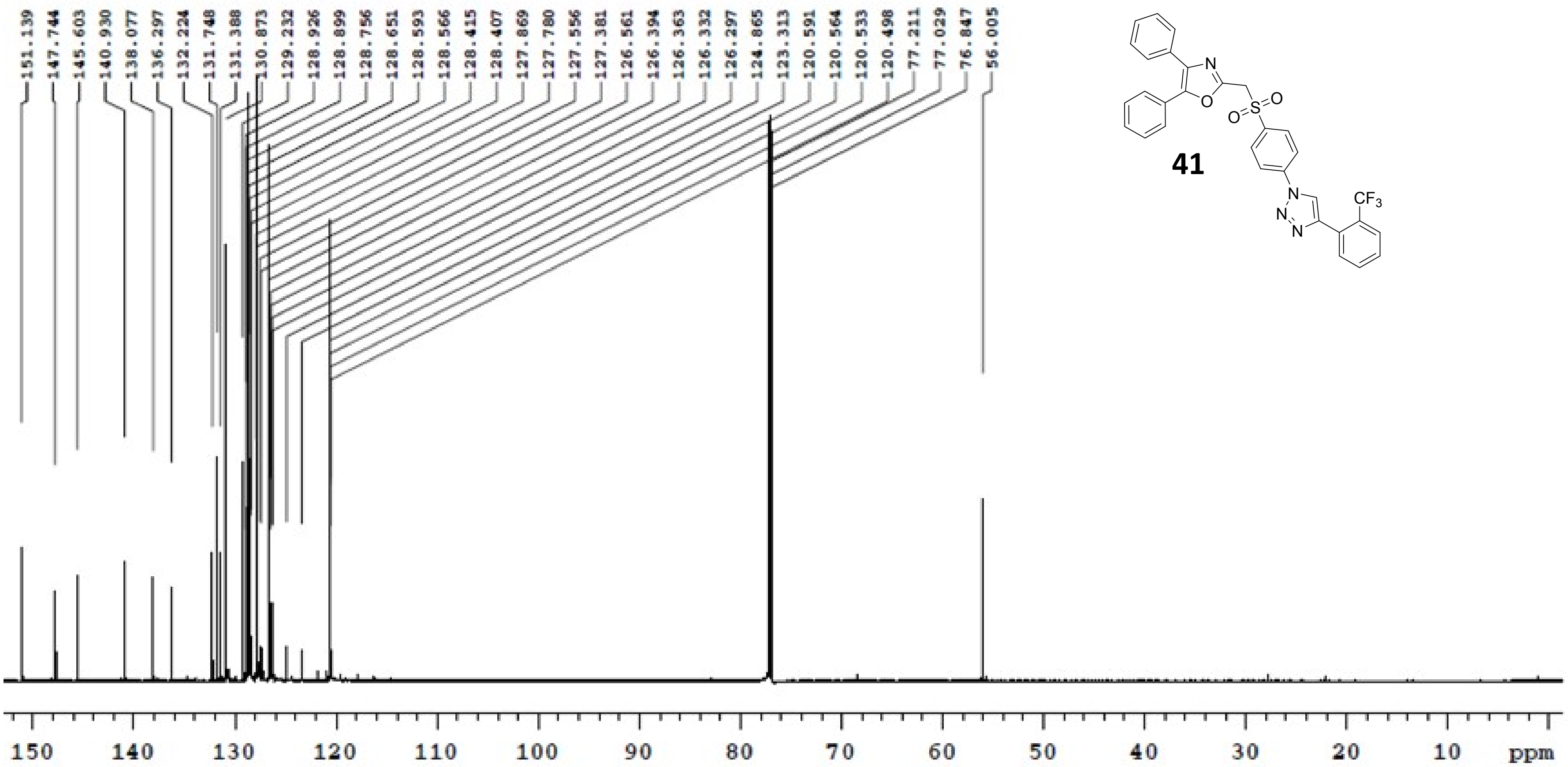

41

^{1}H NMR: 2-(((4-(4-(6-methoxynaphthalen-2-yl)-1H-1,2,3-triazol-1-yl)phenyl)sulfonyl)methyl)-4,5-diphenyloxazole

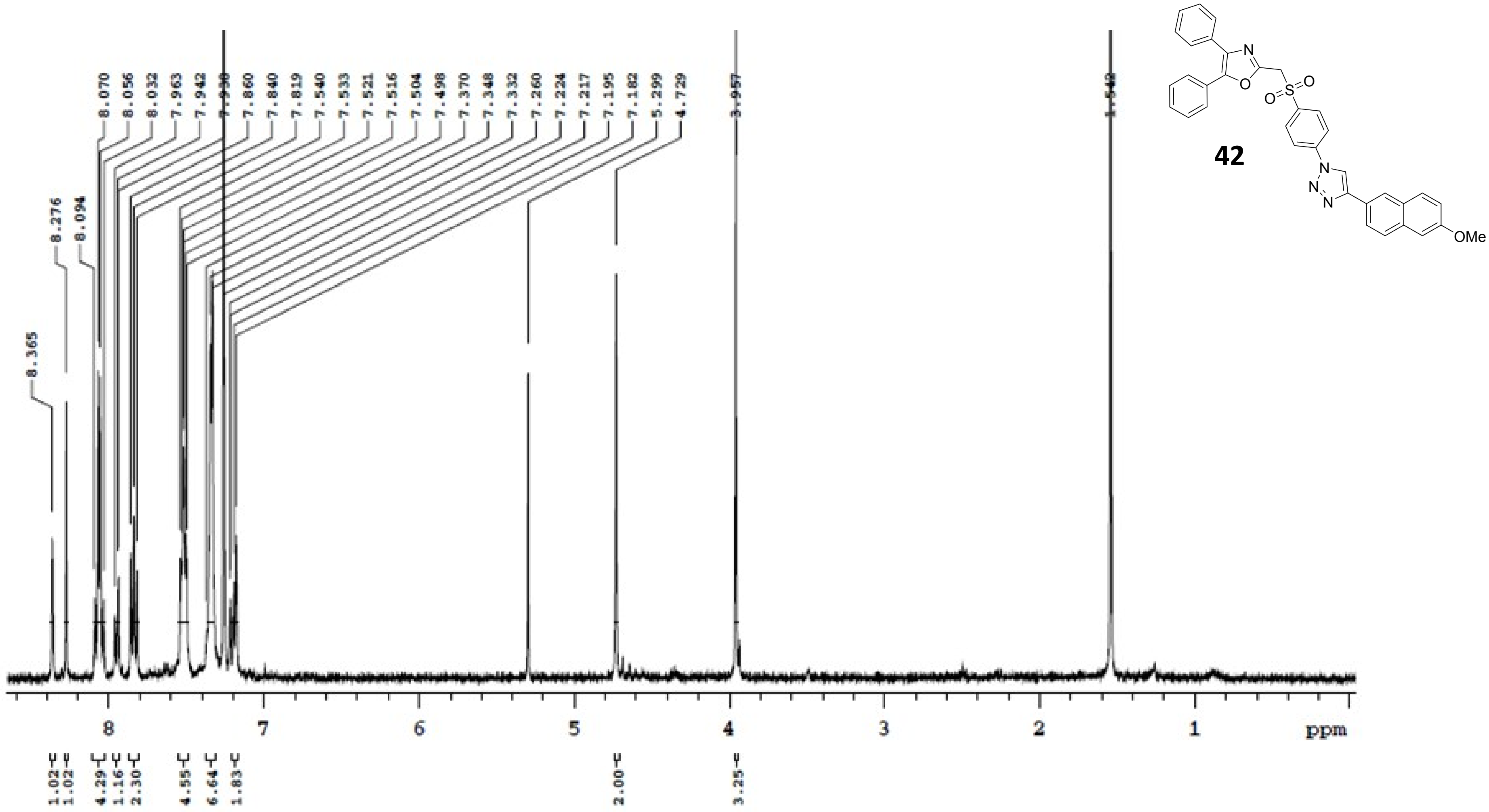

42

^{13}C NMR: 2-(((4-(4-(6-methoxynaphthalen-2-yl)-1H-1,2,3-triazol-1-yl)phenyl)sulfonyl)methyl)-4,5-diphenyloxazole

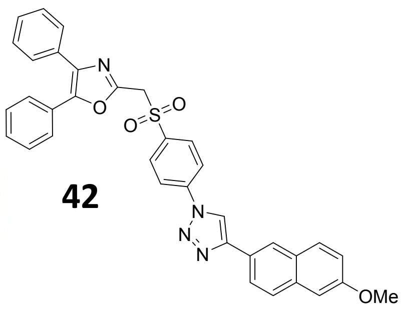

42

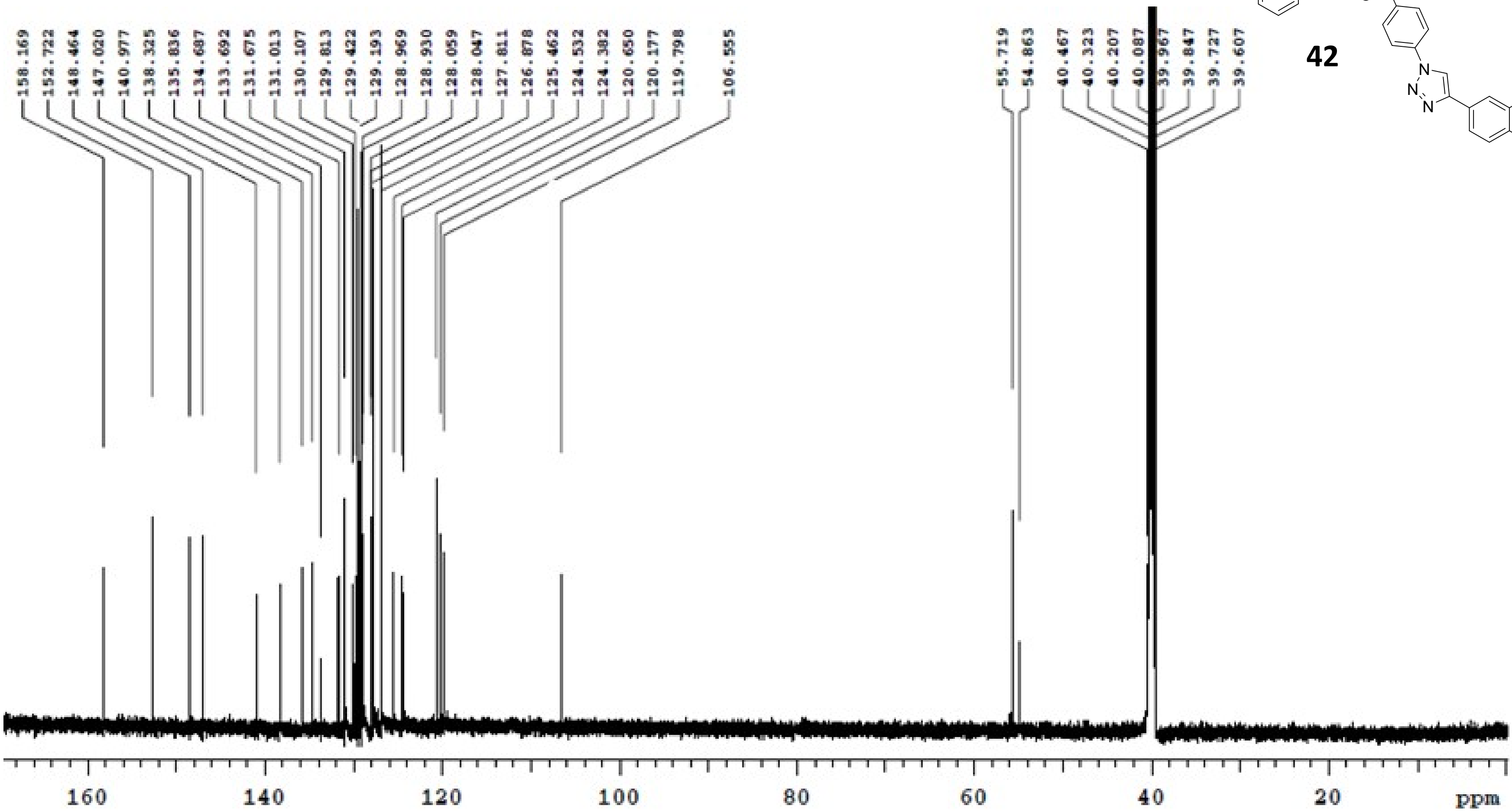

^{1}H NMR: 2-(((4-(4-(4-pentylphenyl)-1H-1,2,3-triazol-1-yl)phenyl)sulfonyl)methyl)-4,5-diphenyloxazole

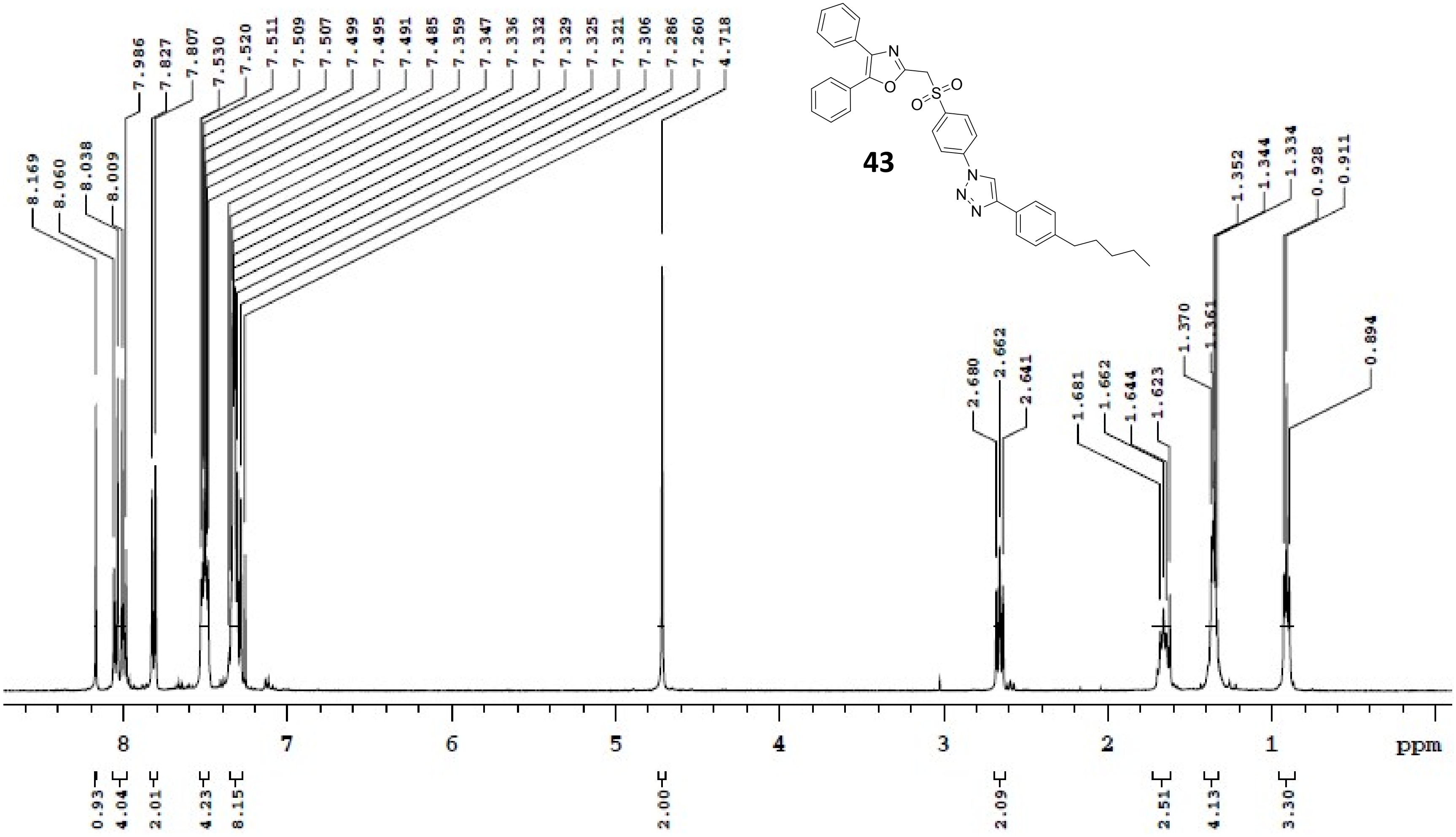

^{13}C NMR: 2-(((4-(4-(4-pentylphenyl)-1H-1,2,3-triazol-1-yl)phenyl)sulfonyl)methyl)-4,5-diphenyloxazole

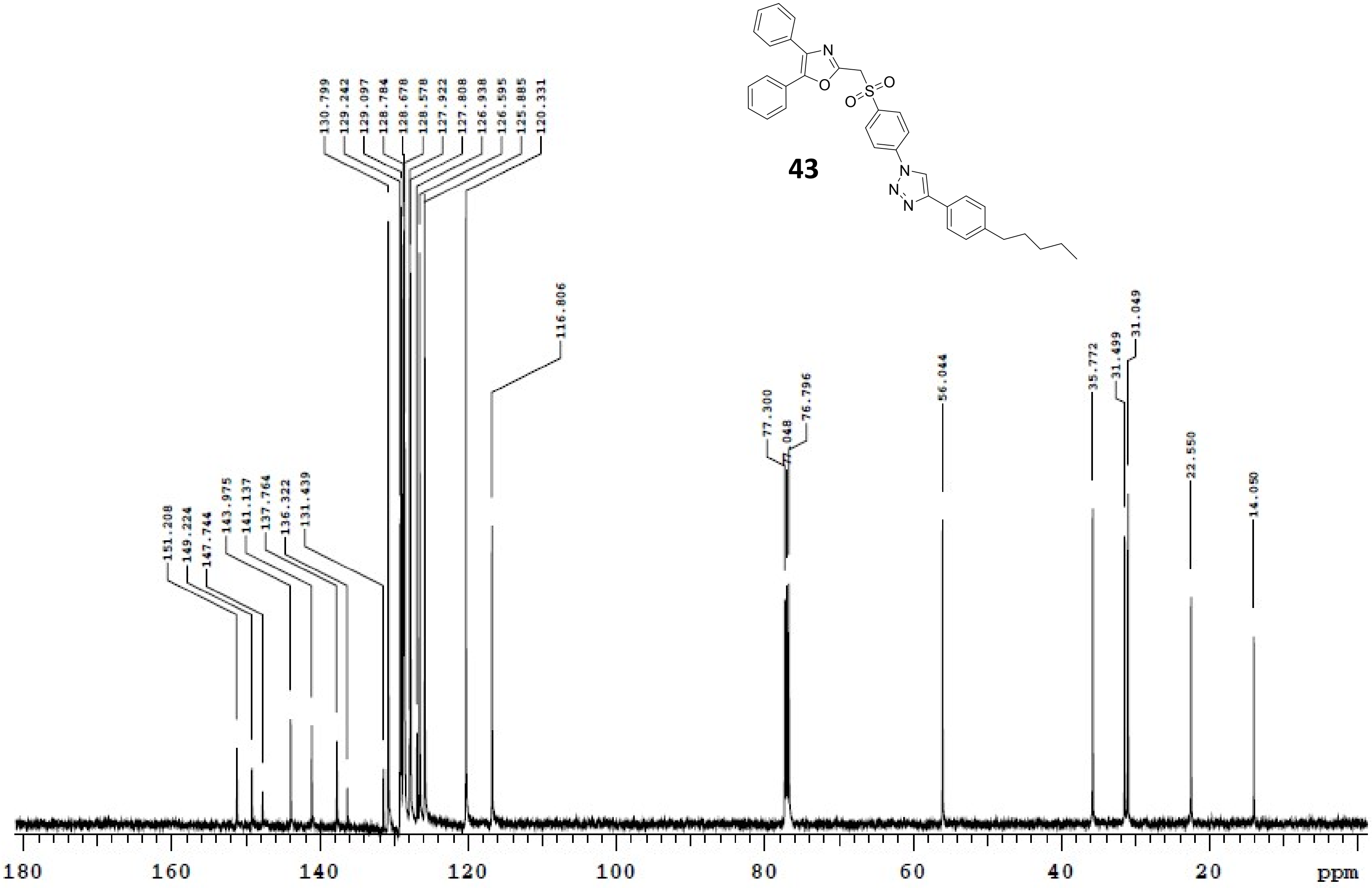

^{1}H NMR: 2-(((4-(4-(4-phenoxyphenyl)-1H-1,2,3-triazol-1-yl)phenyl)sulfonyl)methyl)-4,5-diphenyloxazole

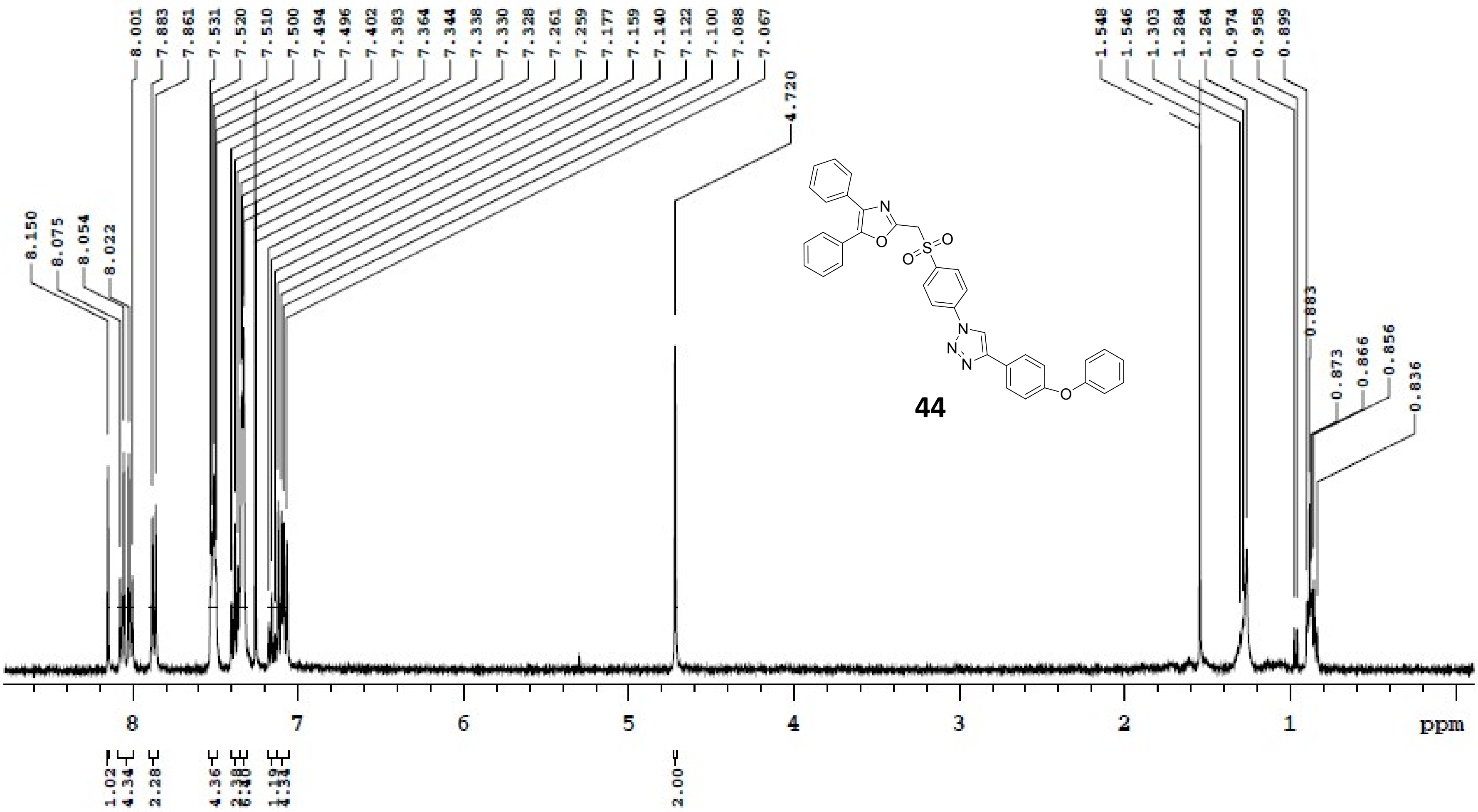

^{13}C NMR: 2-(((4-(4-(4-phenoxyphenyl)-1H-1,2,3-triazol-1-yl)phenyl)sulfonyl)methyl)-4,5-diphenyloxazole

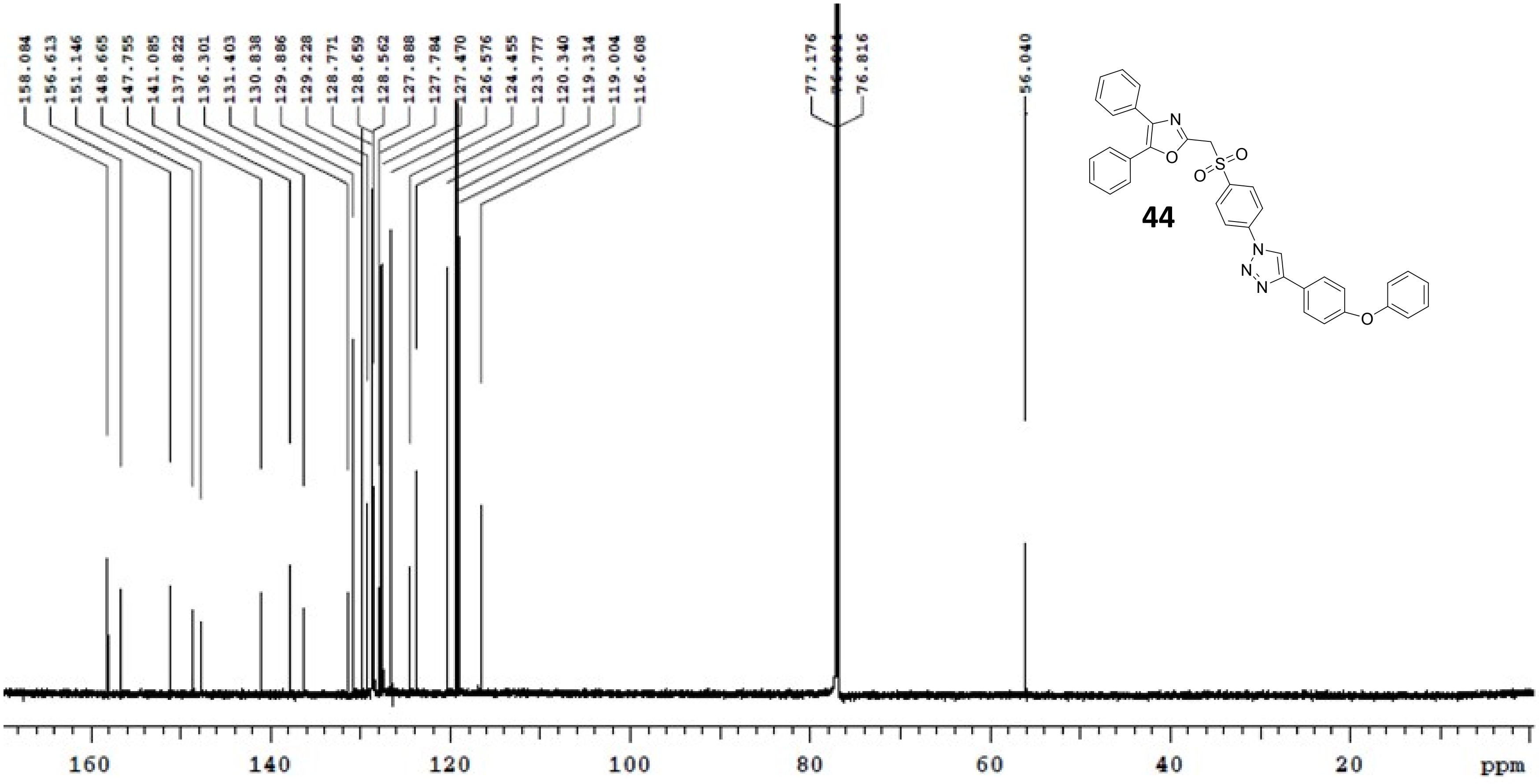

^{1}H NMR: 2-(((4-(4-(3,5-bis(trifluoromethyl)phenyl)-1H-1,2,3-triazol-1-yl)phenyl)sulfonyl)methyl)-4,5-diphenyloxazole

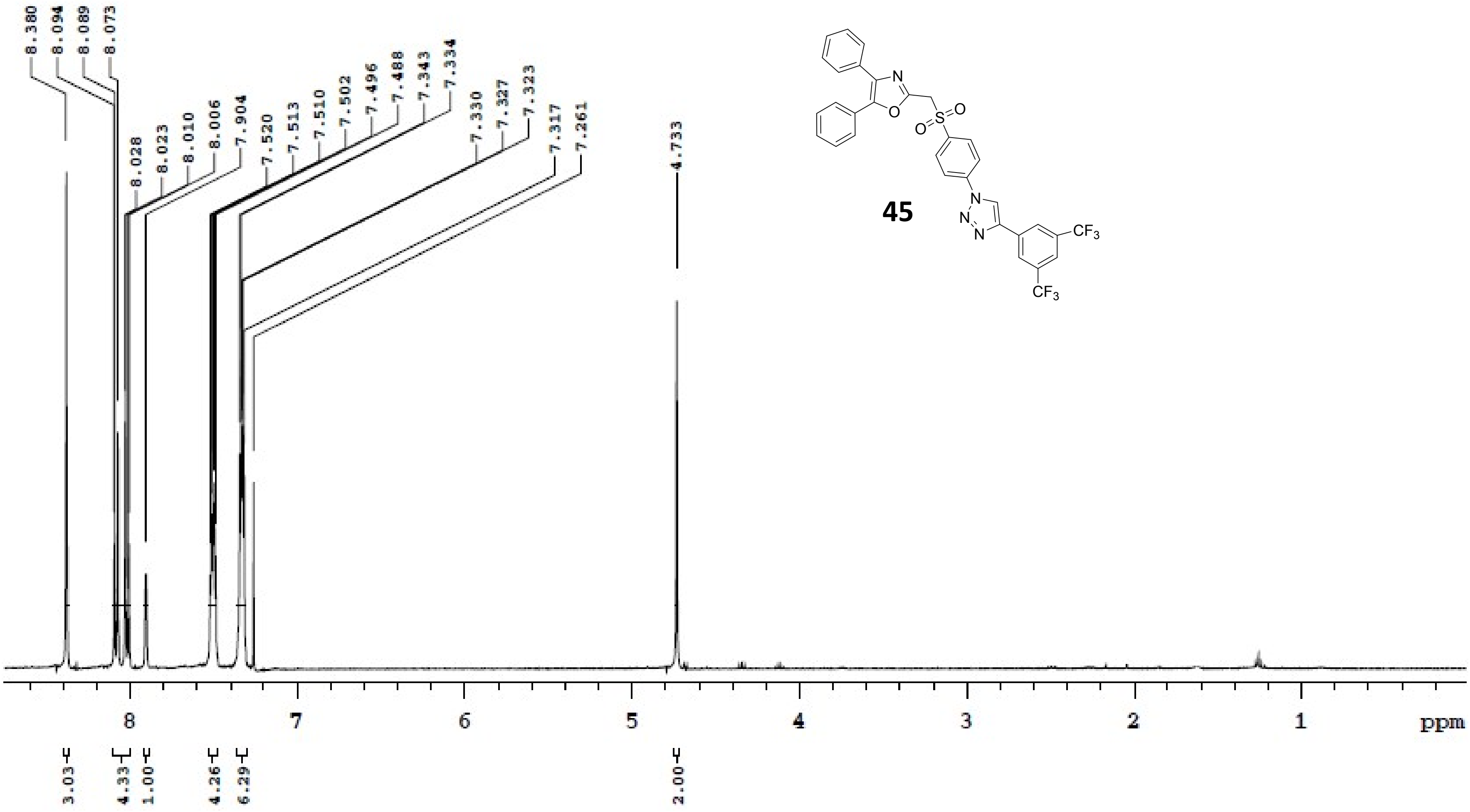

^{13}C NMR: 2-(((4-(4-(3,5-bis(trifluoromethyl)phenyl)-1H-1,2,3-triazol-1-yl)phenyl)sulfonyl)methyl)-4,5-diphenyloxazole

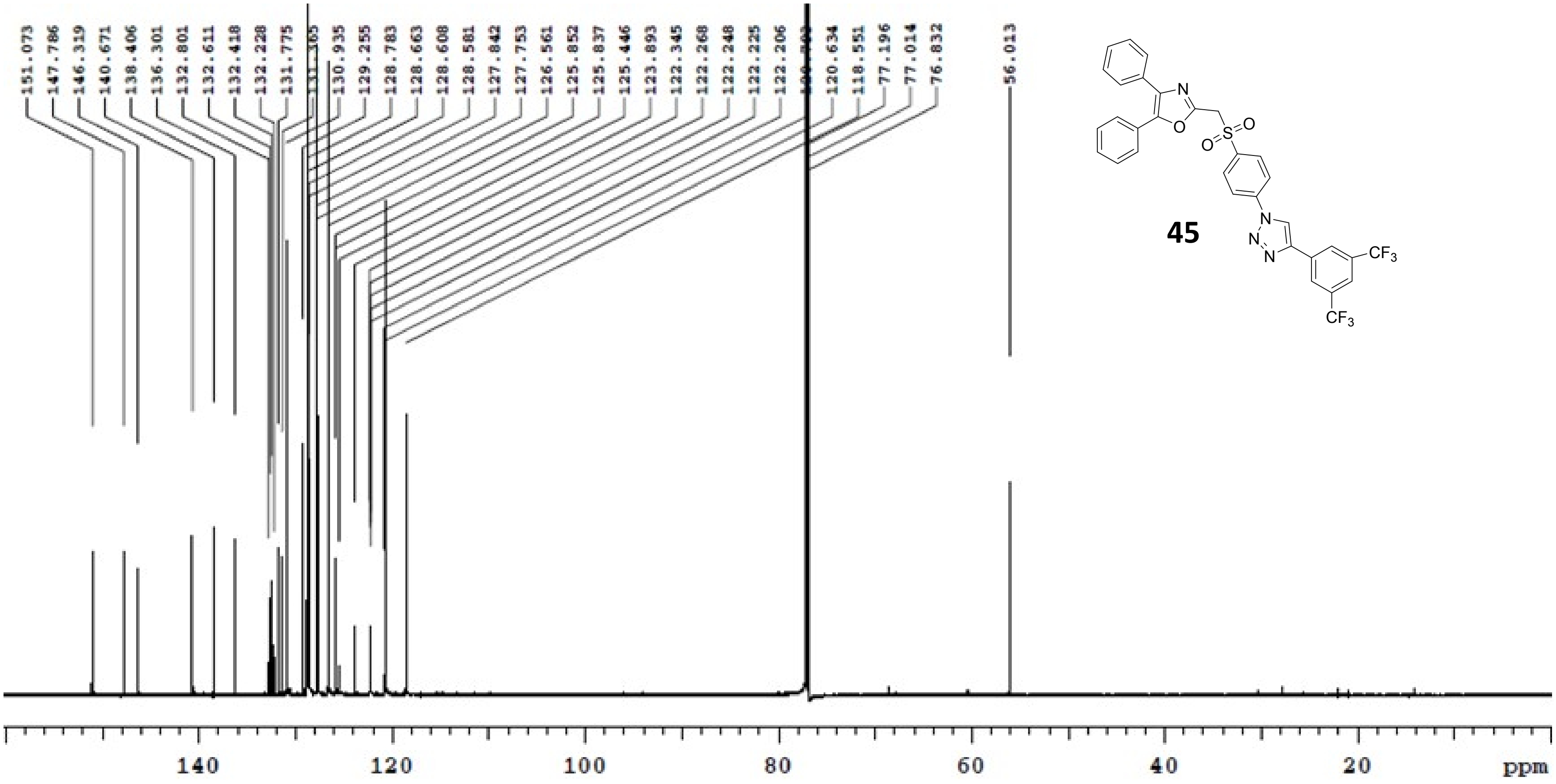

45

^{1}H NMR: 4,5-bis(4-fluorophenyl)-2-(((4-(4-(3-fluorophenyl)-1H-1,2,3-triazol-1-yl)phenyl)sulfinyl)methyl)oxazole

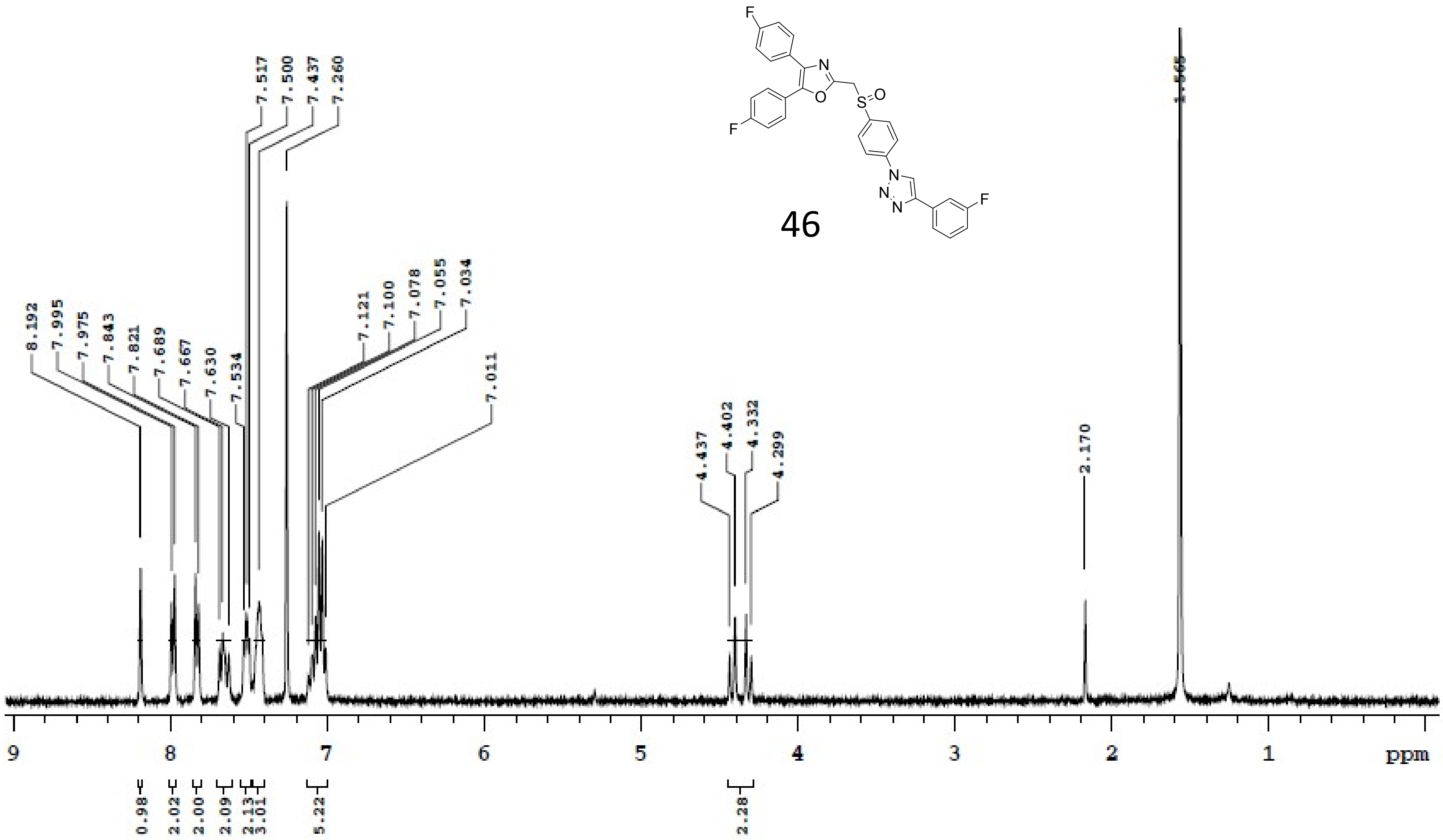

^{13}C NMR: 4,5-bis(4-fluorophenyl)-2-(((4-(4-(3-fluorophenyl)-1H-1,2,3-triazol-1-yl)phenyl)sulfinyl)methyl)oxazole

46

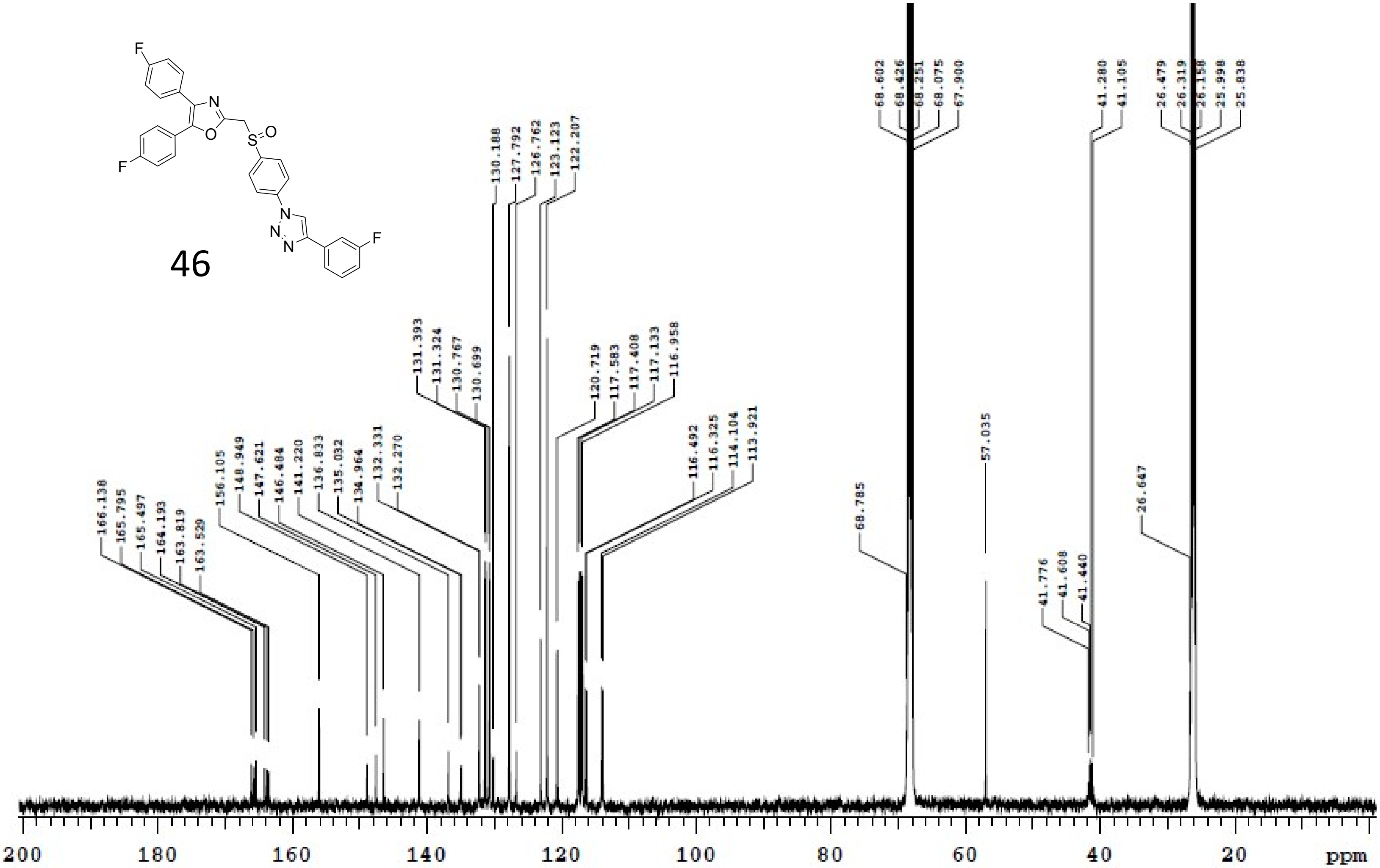

^{1}H NMR: 4,5-bis(4-fluorophenyl)-2-(((4-(4-(4-fluorophenyl)-1H-1,2,3-triazol-1-yl)phenyl)sulfinyl)methyl)oxazole

47

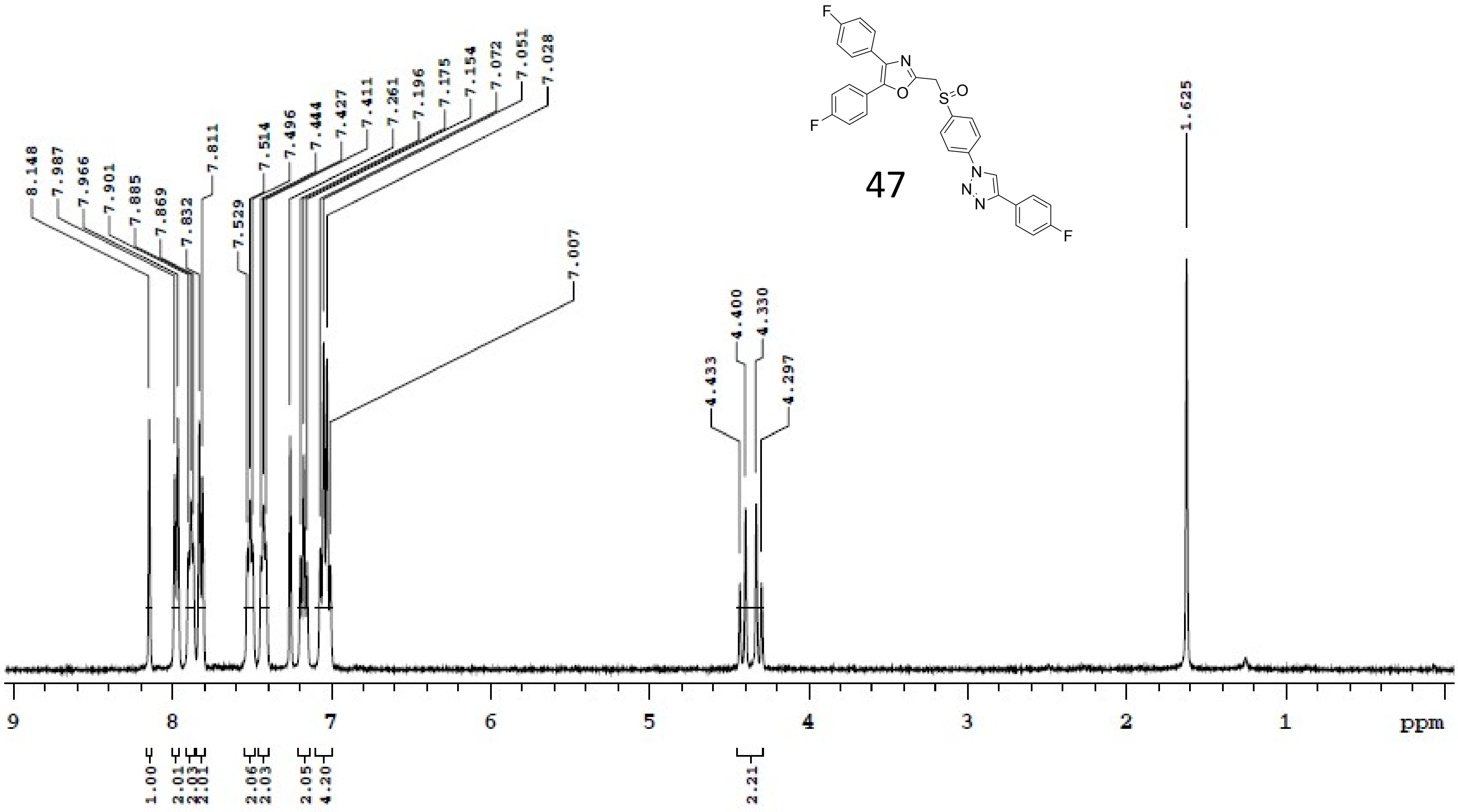

^{13}C NMR: 4,5-bis(4-fluorophenyl)-2-(((4-(4-(4-fluorophenyl)-1H-1,2,3-triazol-1-yl)phenyl)sulfinyl)methyl)oxazole

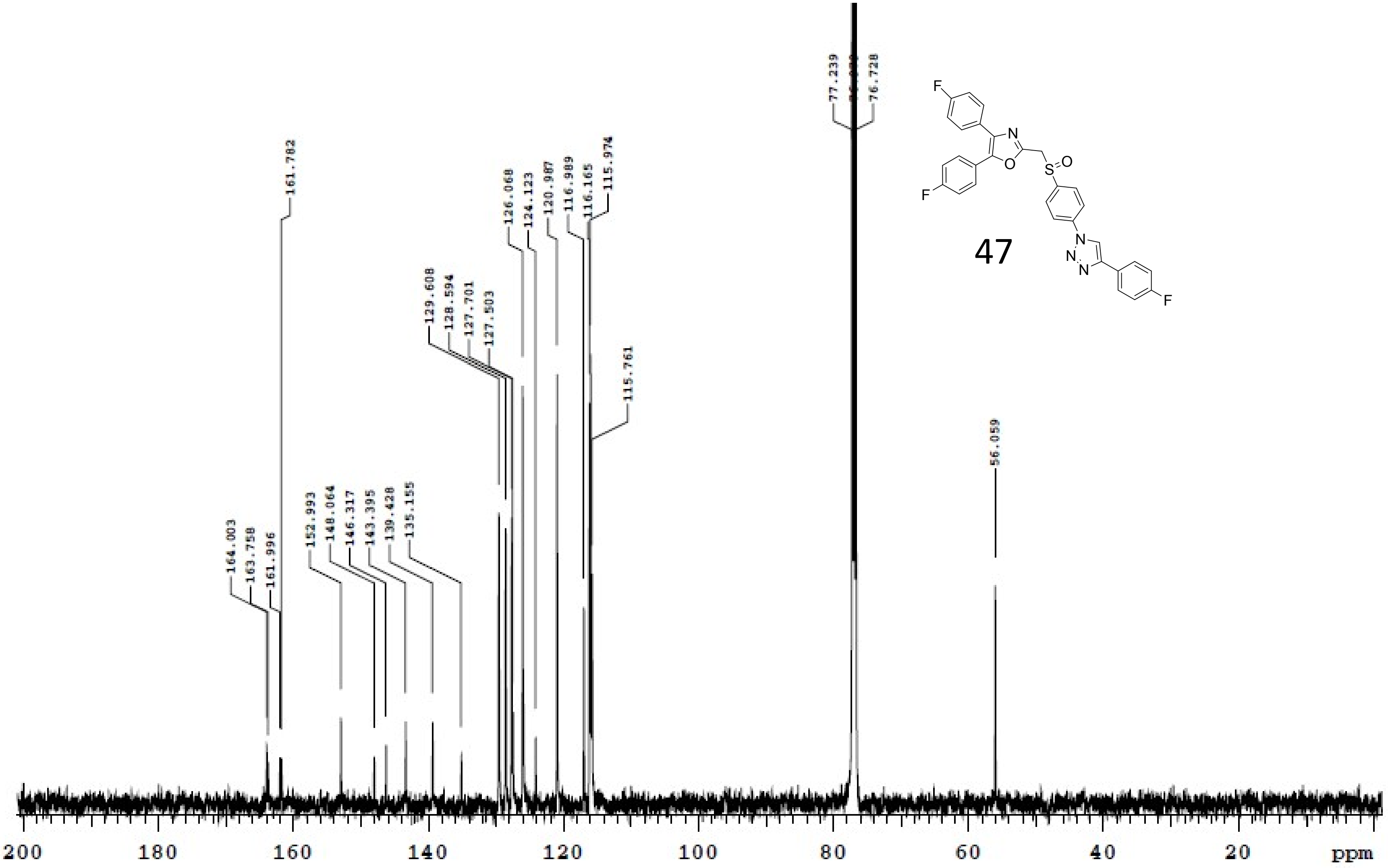

^{1}H NMR: 4,5-bis(4-fluorophenyl)-2-(((4-(4-(2-methoxyphenyl)-1H-1,2,3-triazol-1-yl)phenyl)sulfinyl)methyl)oxazole

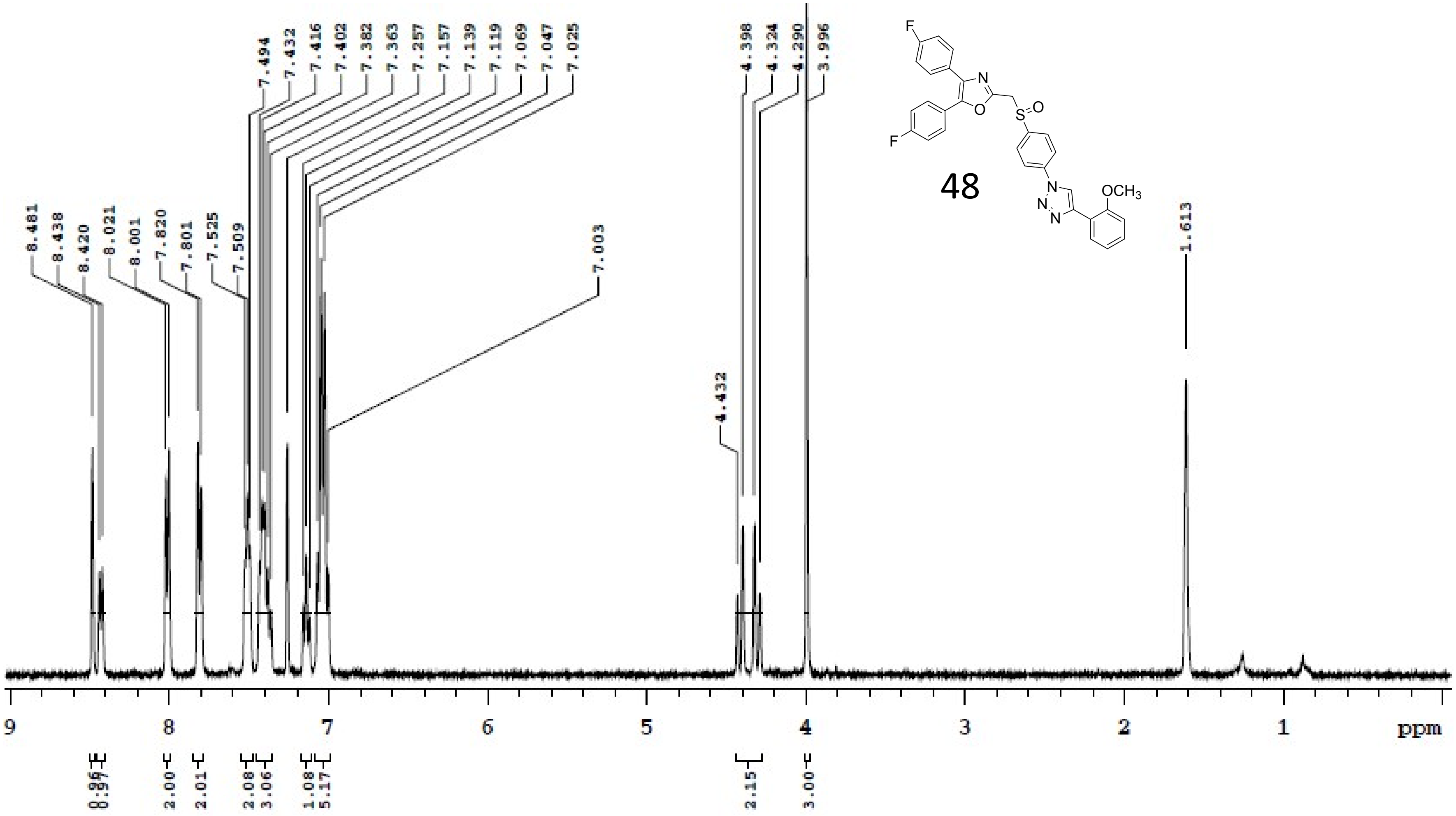

^{13}C NMR: 4,5-bis(4-fluorophenyl)-2-(((4-(4-(2-methoxyphenyl)-1H-1,2,3-triazol-1-yl)phenyl)sulfinyl)methyl)oxazole

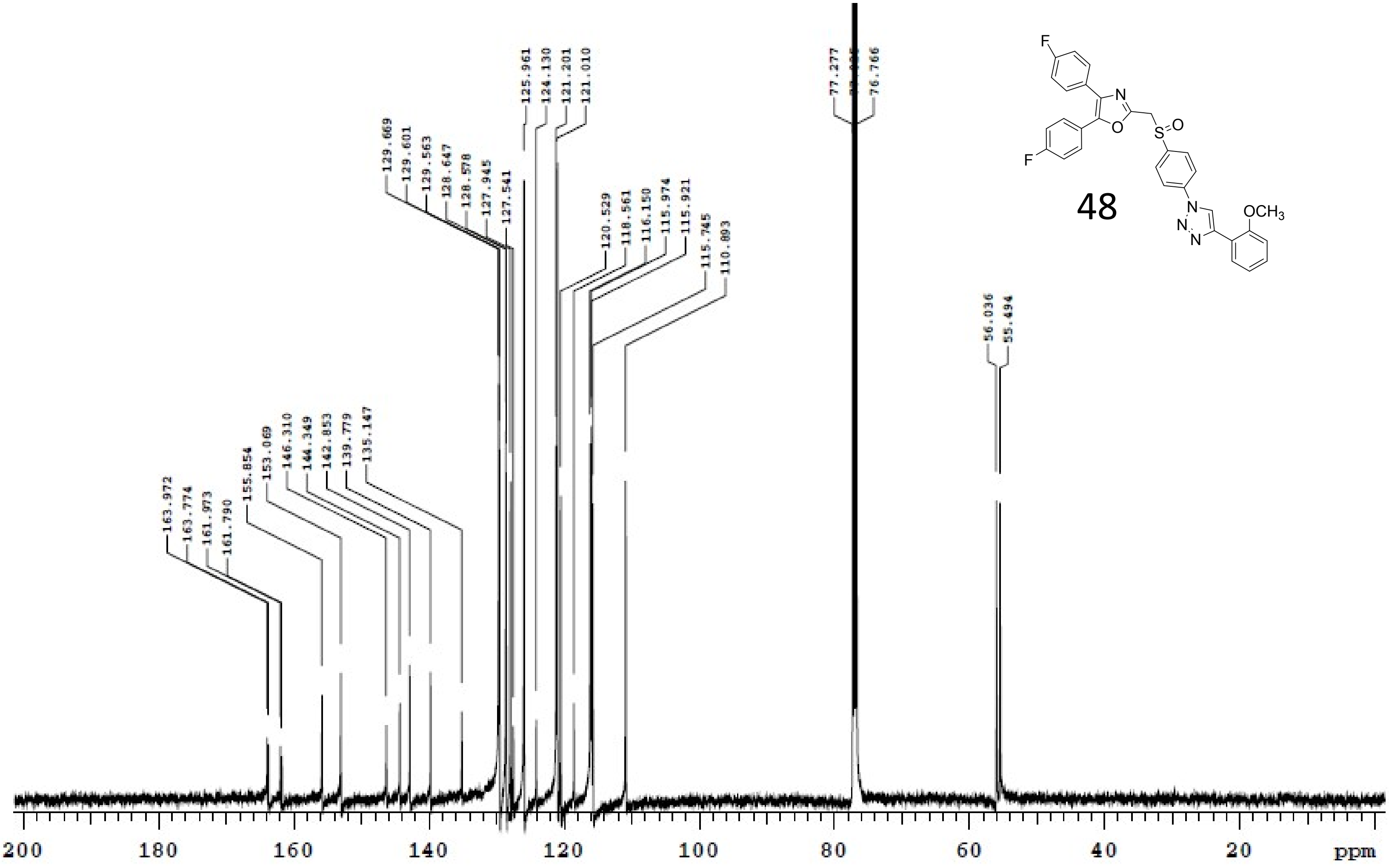

^{1}H NMR: 4,5-bis(4-chlorophenyl)-2-(((4-(4-(3-fluorophenyl)-1H-1,2,3-triazol-1-yl)phenyl)sulfinyl)methyl)oxazole

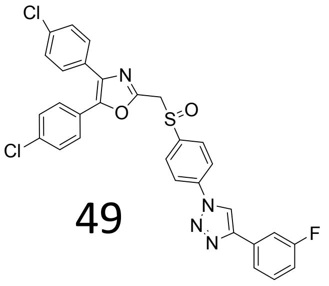

49

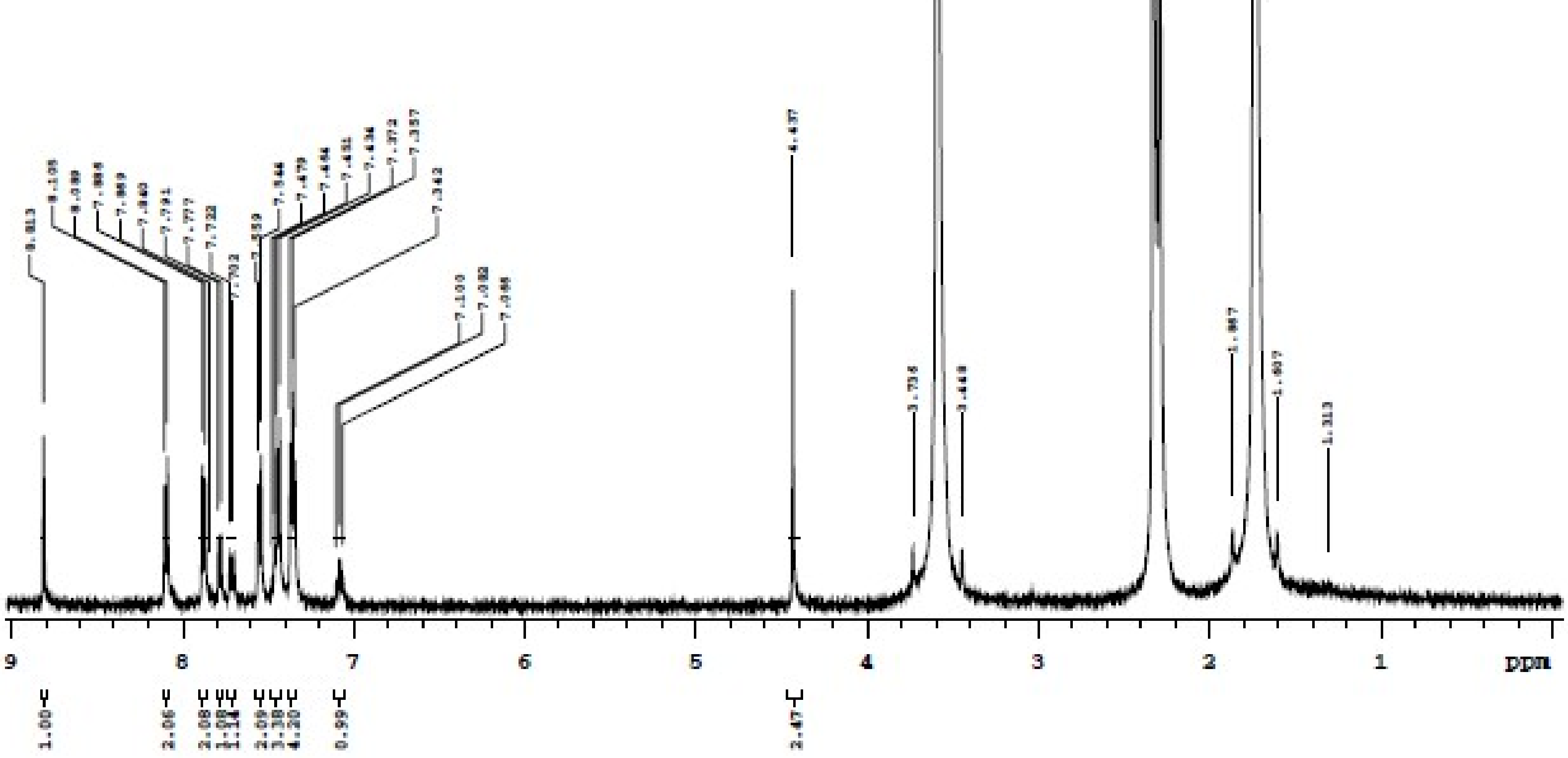

^{13}C NMR: 4,5-bis(4-chlorophenyl)-2-(((4-(4-(3-fluorophenyl)-1H-1,2,3-triazol-1-yl)phenyl)sulfinyl)methyl)oxazole

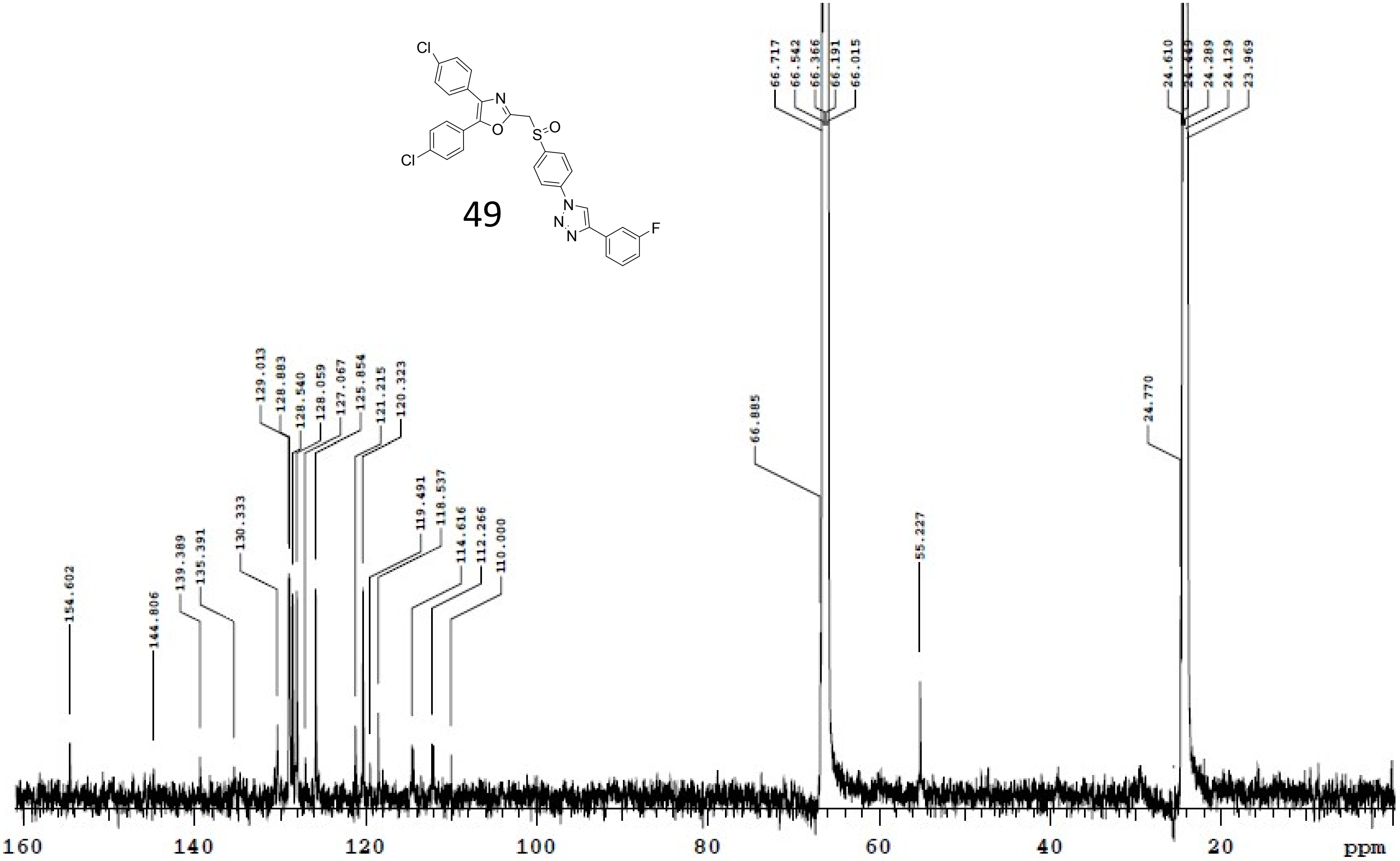

^{1}H NMR: 4,5-bis(4-chlorophenyl)-2-(((4-(4-(4-fluorophenyl)-1H-1,2,3-triazol-1-yl)phenyl)sulfinyl)methyl)oxazole

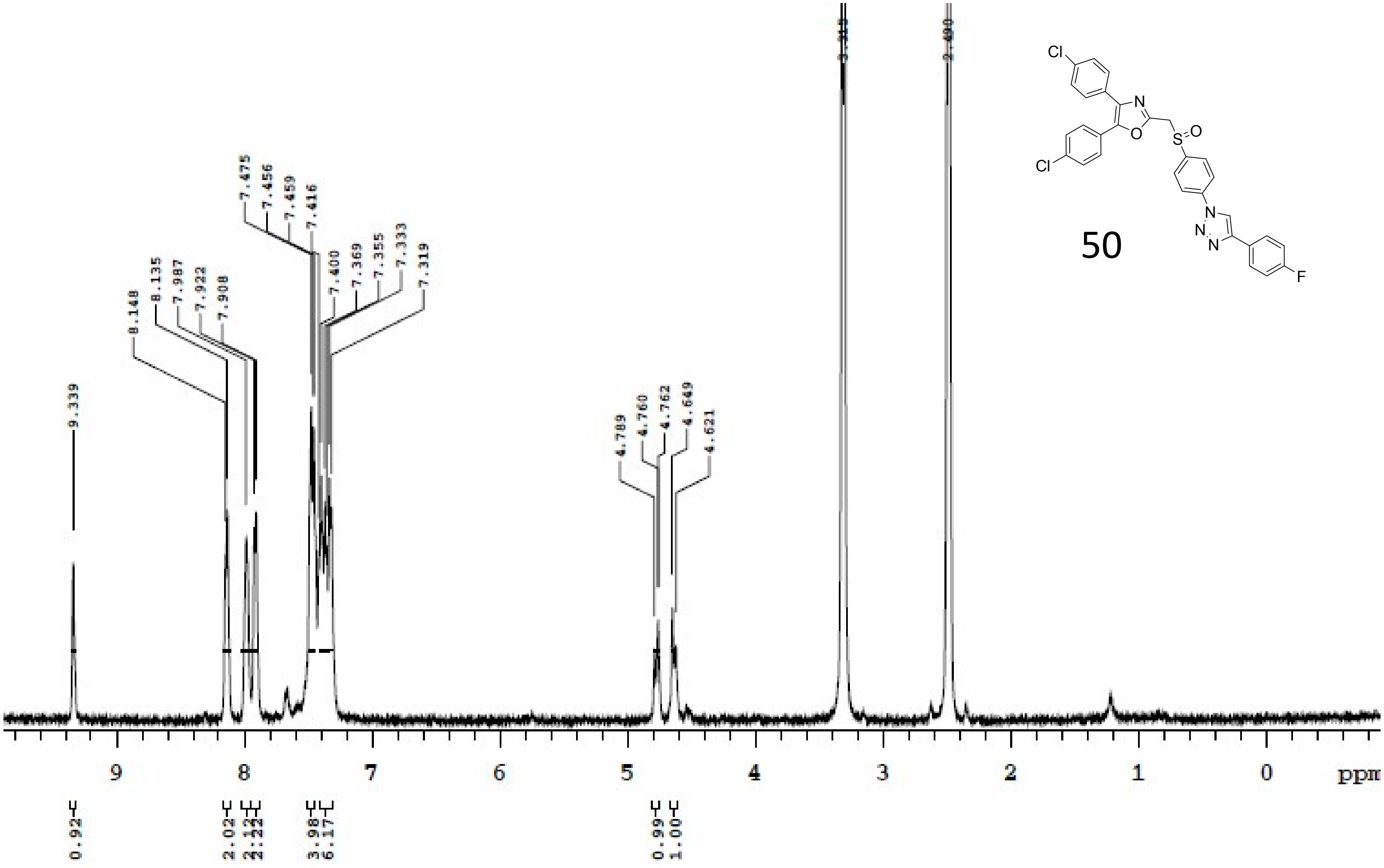

^{13}C NMR: 4,5-bis(4-chlorophenyl)-2-(((4-(4-(4-fluorophenyl)-1H-1,2,3-triazol-1-yl)phenyl)sulfinyl)methyl)oxazole

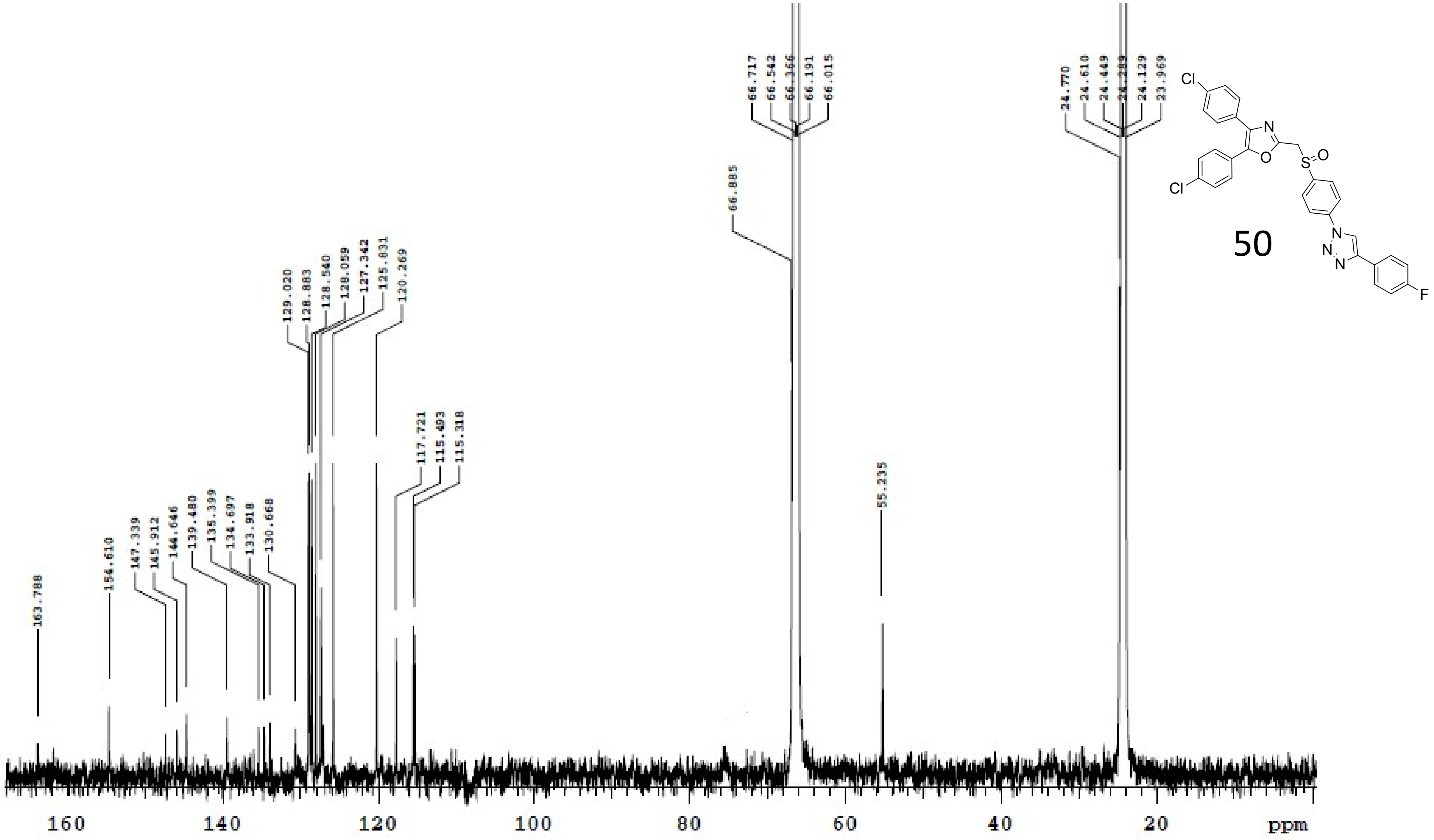

50

^{1}H NMR: 4,5-bis(4-chlorophenyl)-2-(((4-(4-(2-methoxyphenyl)-1H-1,2,3-triazol-1-yl)phenyl)sulfinyl)methyl)oxazole

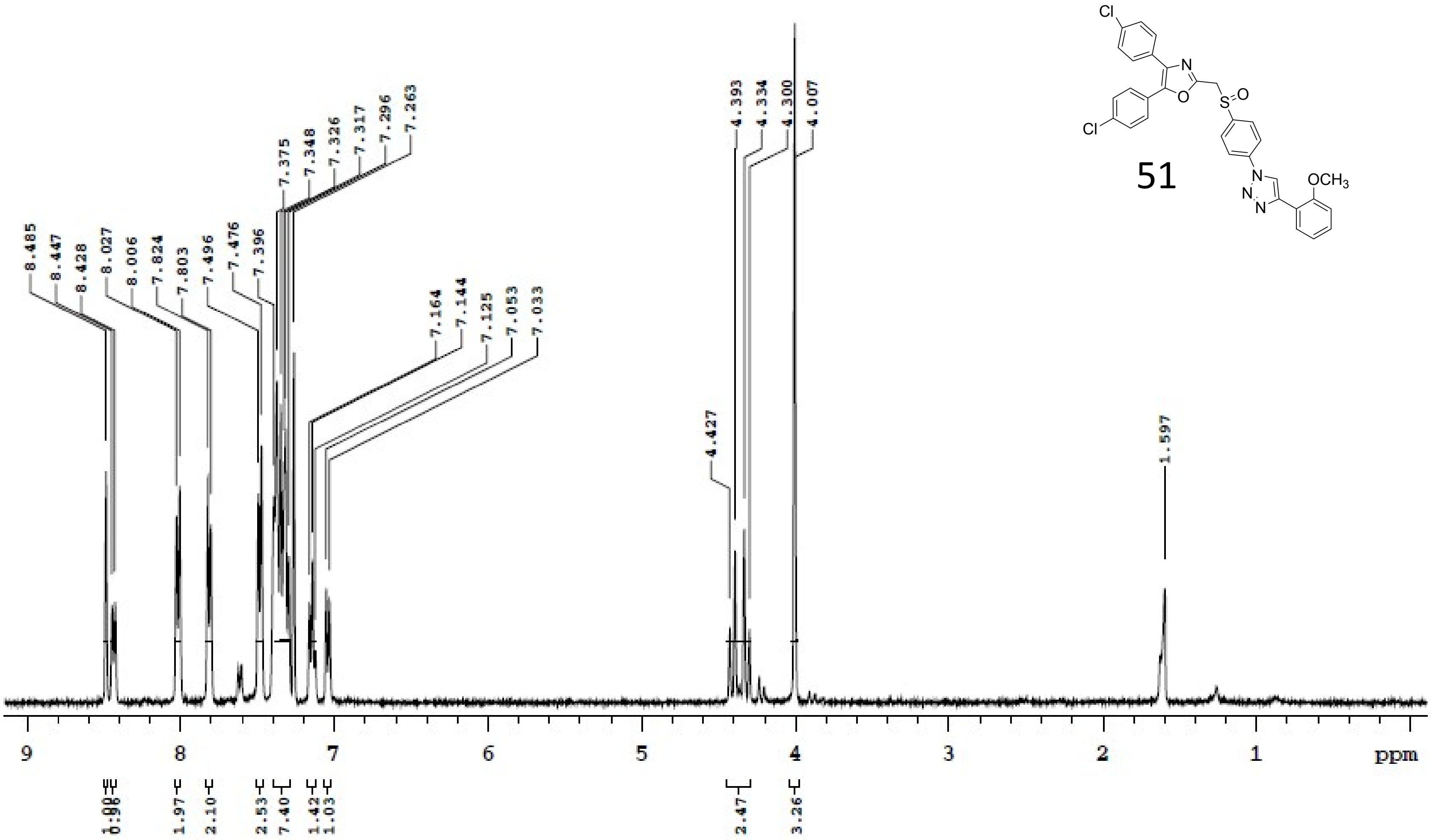

51

^{13}C NMR: 4,5-bis(4-chlorophenyl)-2-(((4-(4-(2-methoxyphenyl)-1H-1,2,3-triazol-1-yl)phenyl)sulfinyl)methyl)oxazole

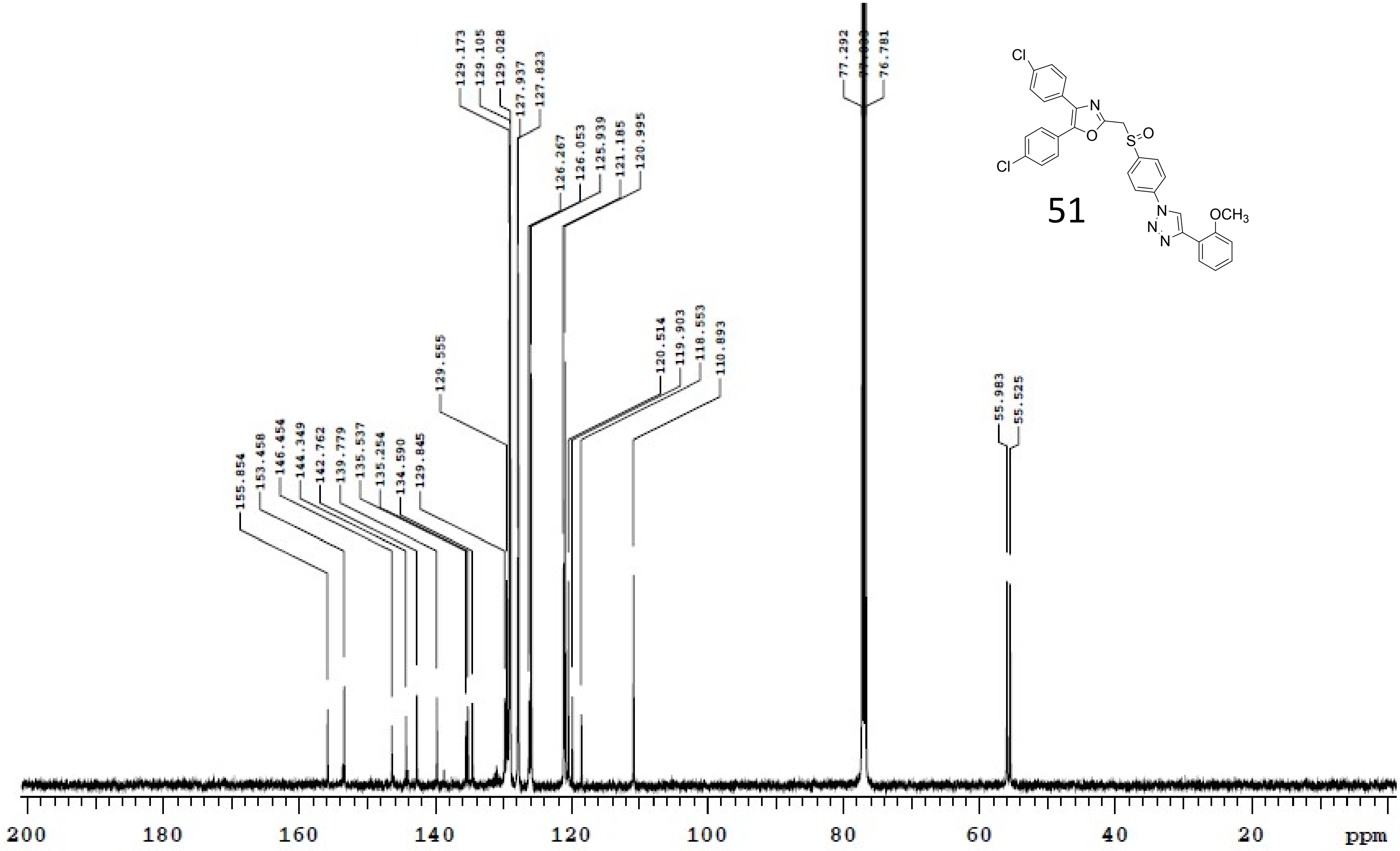

^{1}H NMR: 4,5-bis(4-fluorophenyl)-2-(((4-(4-(3-fluorophenyl)-1H-1,2,3-triazol-1-yl)phenyl)sulfonyl)methyl)oxazole

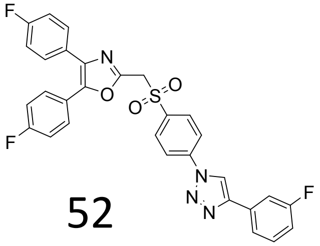

52

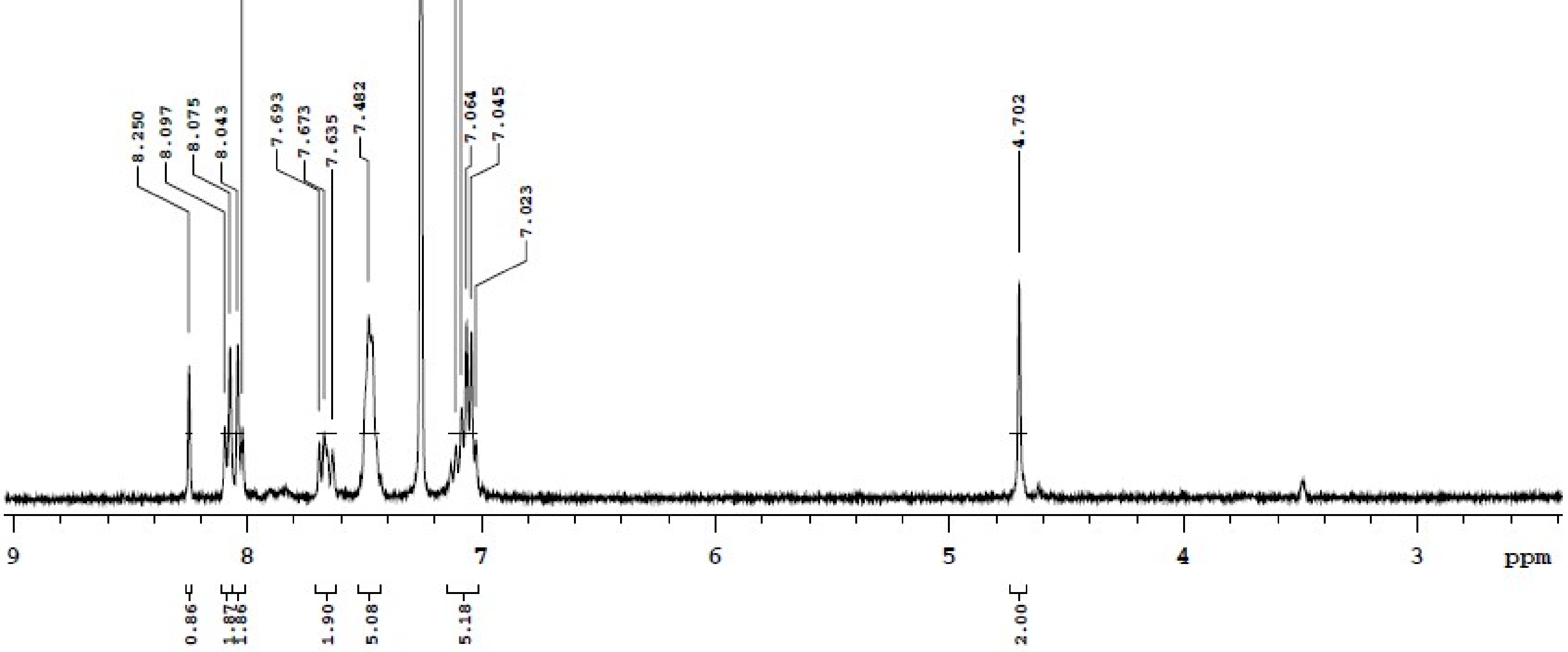

^{13}C NMR: 4,5-bis(4-fluorophenyl)-2-(((4-(4-(3-fluorophenyl)-1H-1,2,3-triazol-1-yl)phenyl)sulfonyl)methyl)oxazole

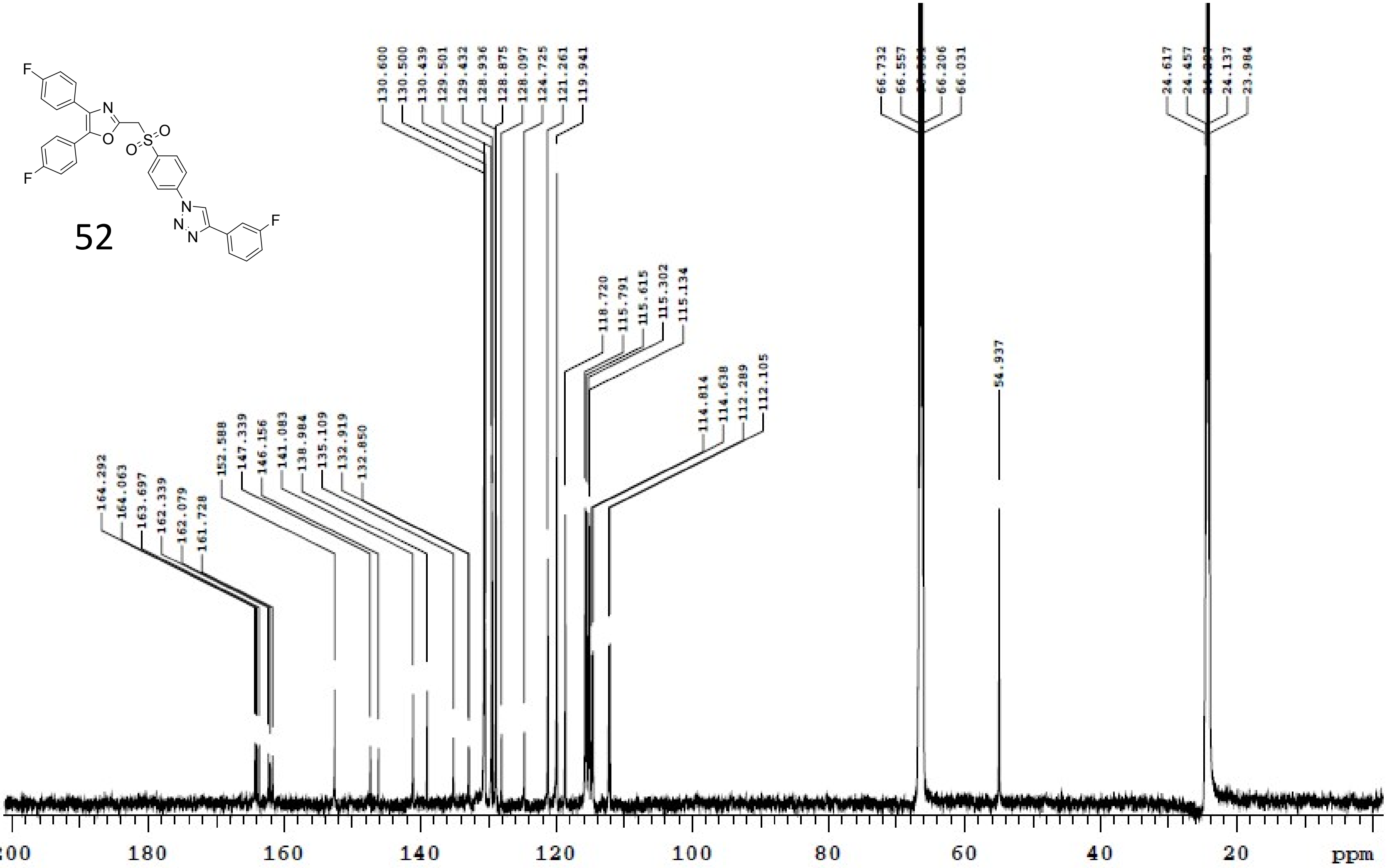

^{1}H NMR: 4,5-bis(4-fluorophenyl)-2-(((4-(4-(4-fluorophenyl)-1H-1,2,3-triazol-1-yl)phenyl)sulfonyl)methyl)oxazole

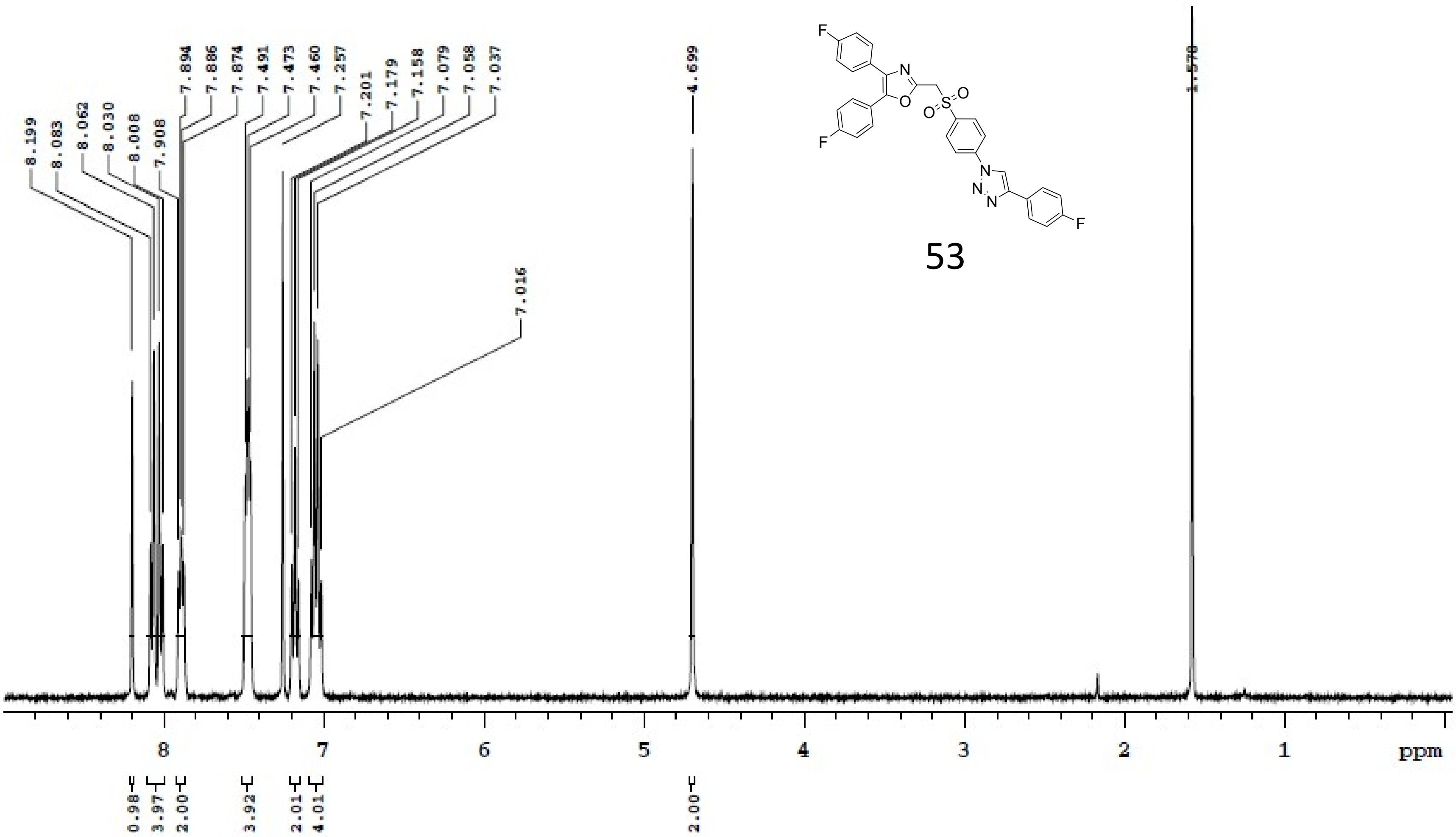

53

^{13}C NMR: 4,5-bis(4-fluorophenyl)-2-(((4-(4-(4-fluorophenyl)-1H-1,2,3-triazol-1-yl)phenyl)sulfonyl)methyl)oxazole

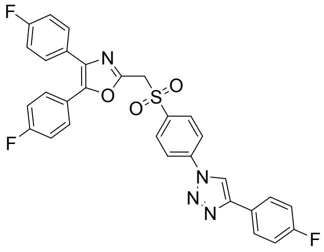

53

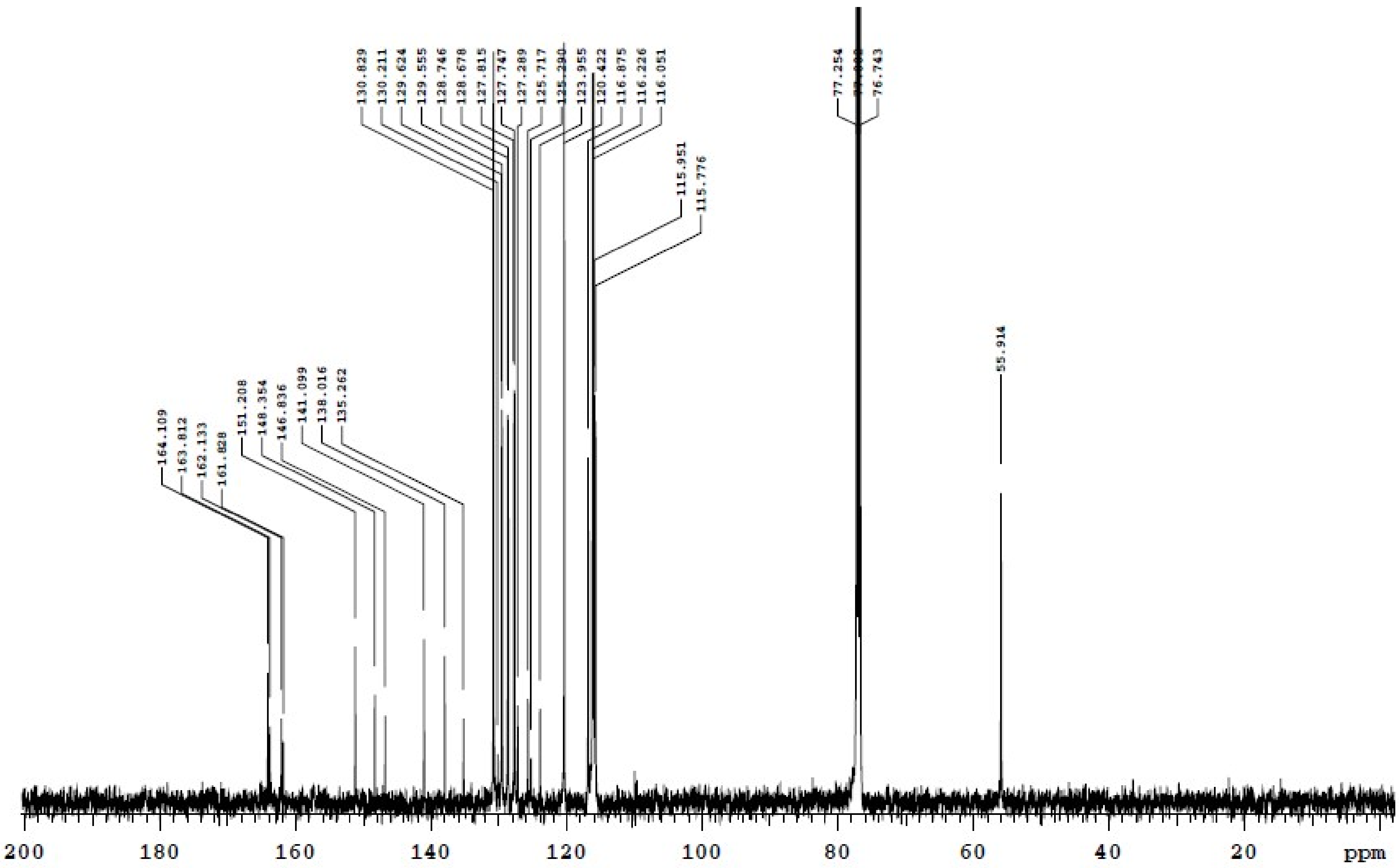

^{1}H NMR: **4,5-bis(4-fluorophenyl)-2-(((4-(4-(2-methoxyphenyl)-1H-1,2,3-triazol-1-yl)phenyl)sulfonyl)methyl)oxazole**

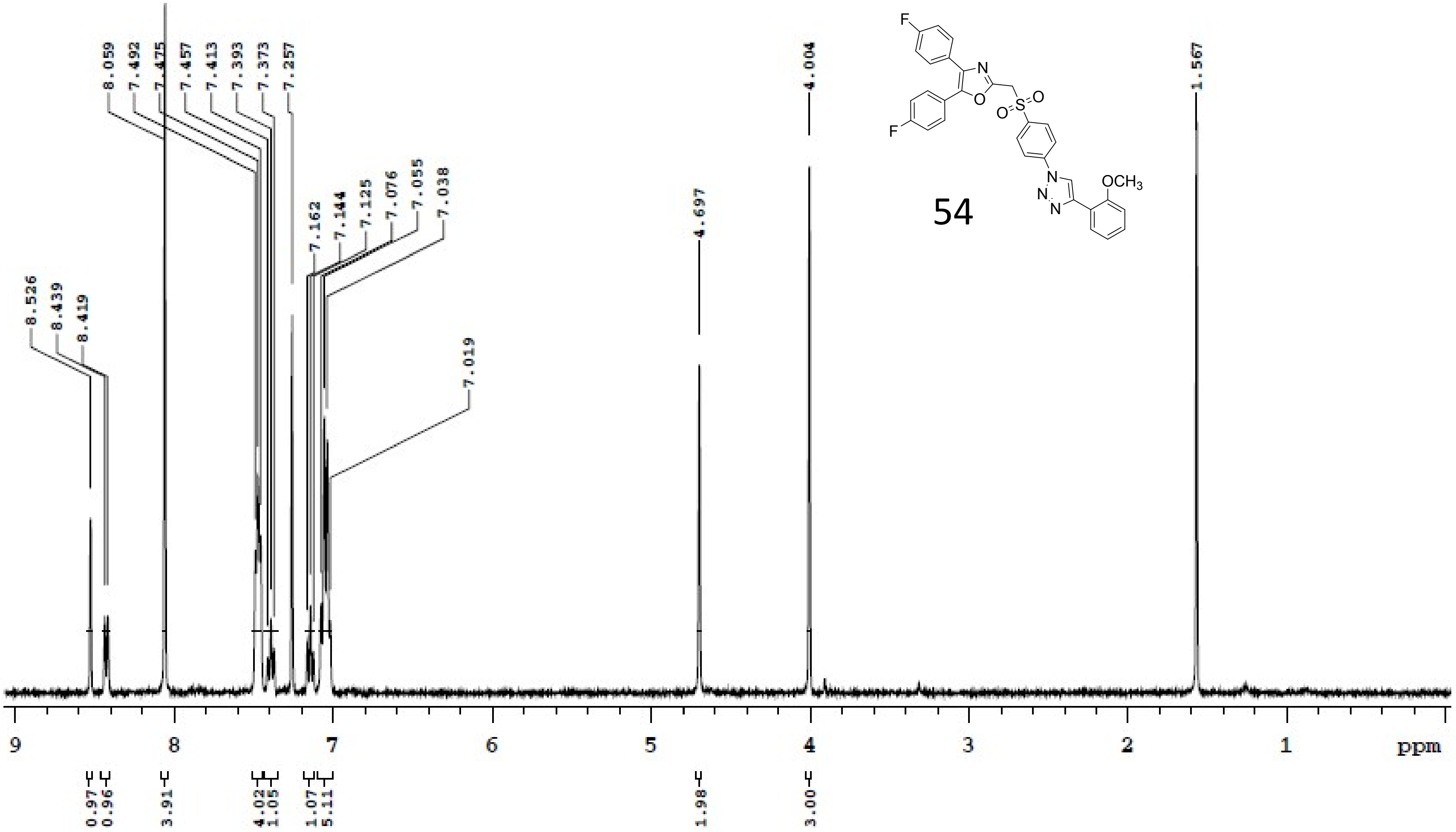

^{13}C NMR: 4,5-bis(4-fluorophenyl)-2-(((4-(4-(2-methoxyphenyl)-1H-1,2,3-triazol-1-yl)phenyl)sulfonyl)methyl)oxazole

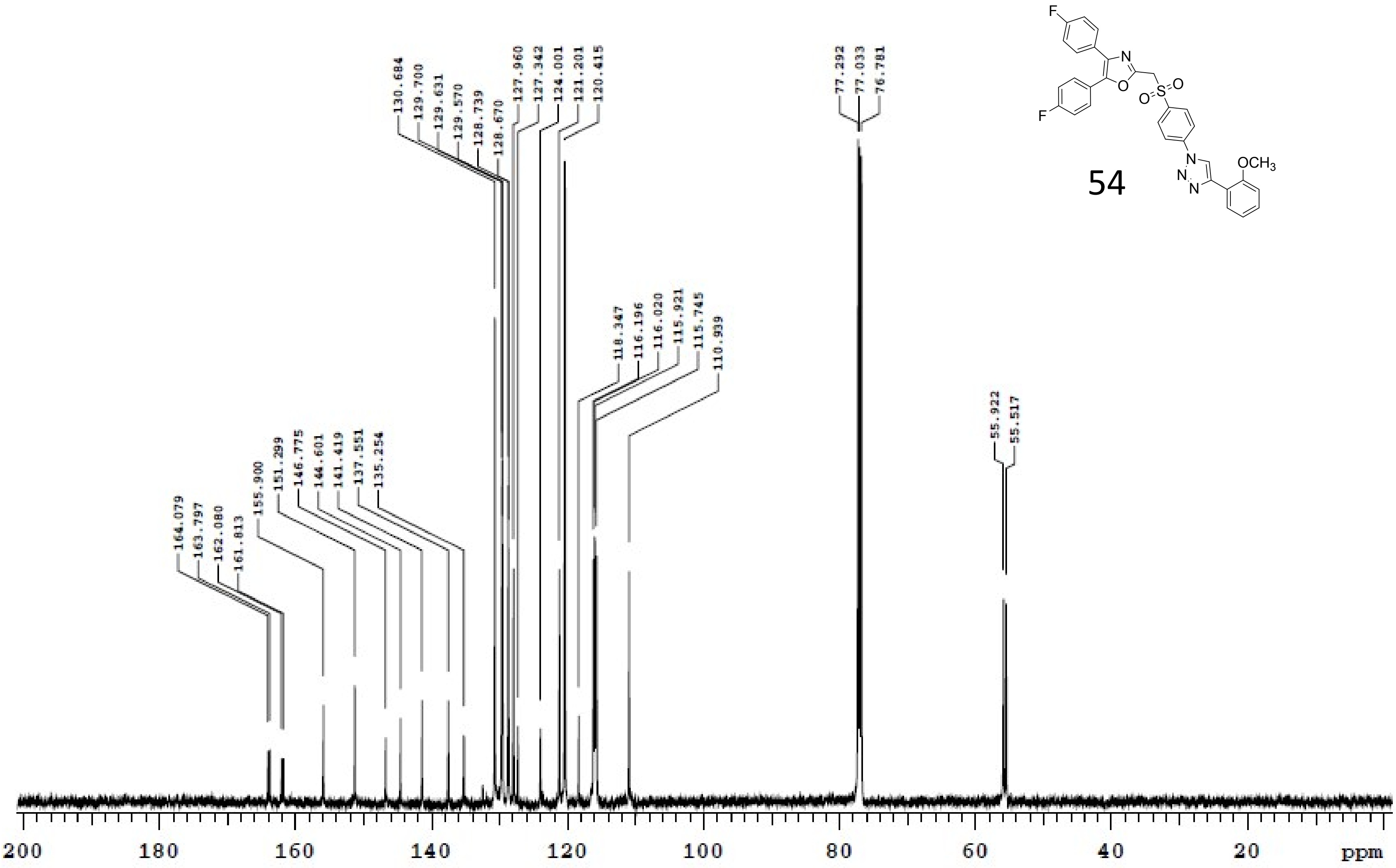

54

¹H NMR: 4,5-bis(4-chlorophenyl)-2-(((4-(4-(3-fluorophenyl)-1H-1,2,3-triazol-1-yl)phenyl)sulfonyl)methyl)oxazole

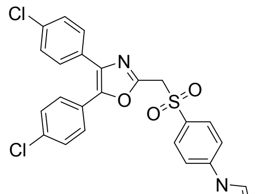

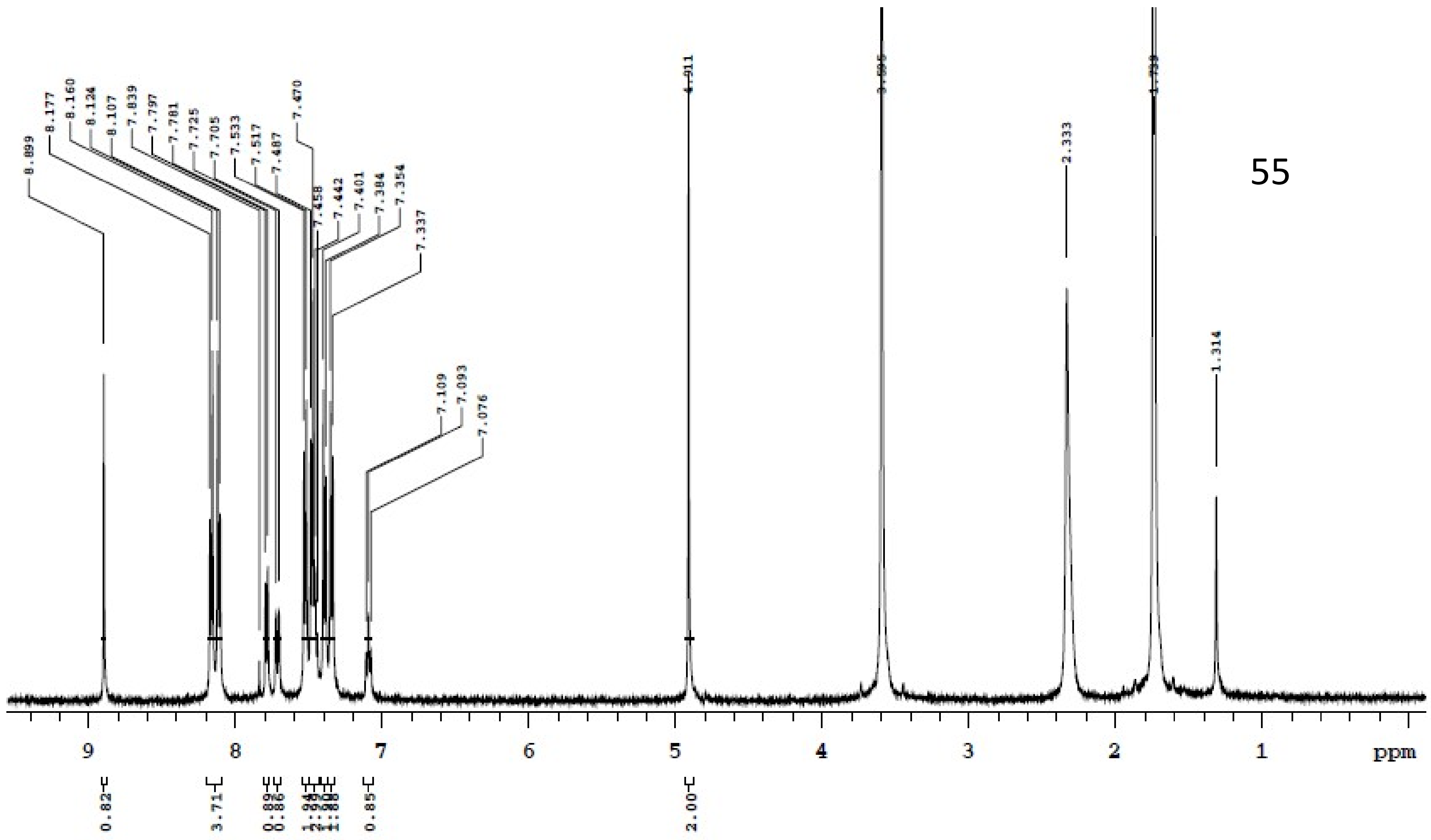

^{13}C NMR: 4,5-bis(4-chlorophenyl)-2-(((4-(4-(3-fluorophenyl)-1H-1,2,3-triazol-1-yl)phenyl)sulfonyl)methyl)oxazole

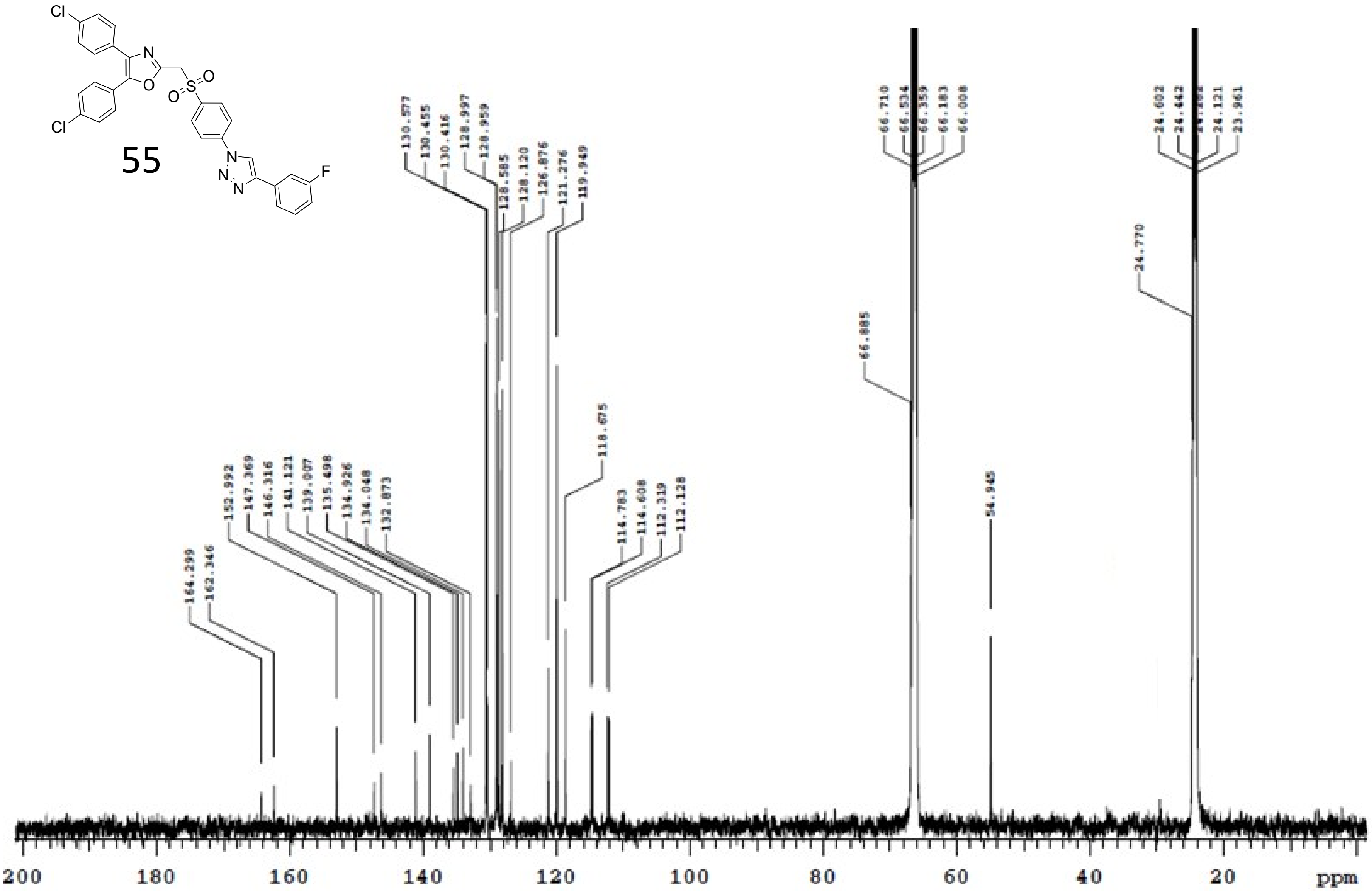

^{1}H NMR: 4,5-bis(4-chlorophenyl)-2-(((4-(4-(4-fluorophenyl)-1H-1,2,3-triazol-1-yl)phenyl)sulfonyl)methyl)oxazole

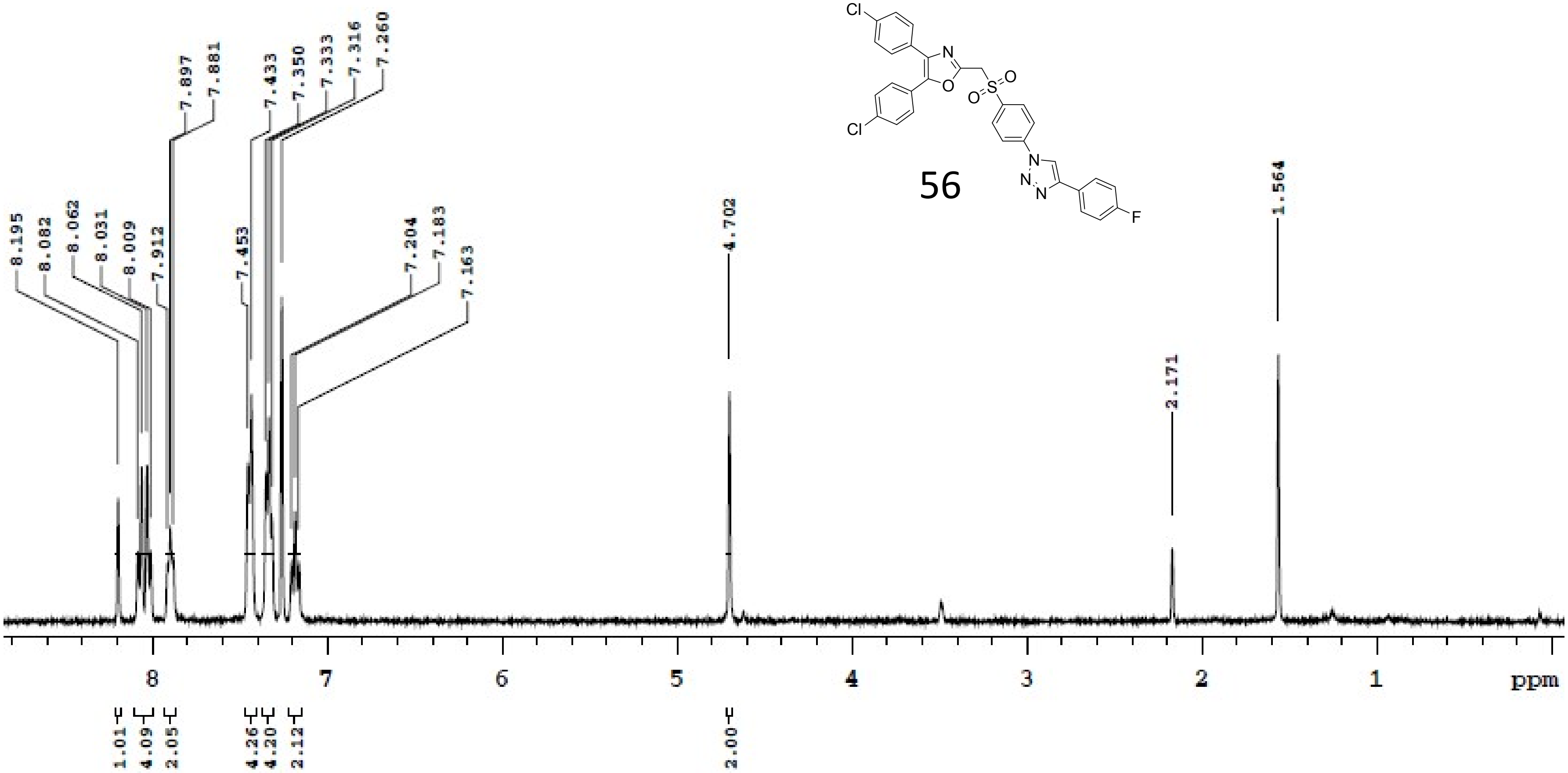

^{13}C NMR: 4,5-bis(4-chlorophenyl)-2-(((4-(4-(4-fluorophenyl)-1H-1,2,3-triazol-1-yl)phenyl)sulfonyl)methyl)oxazole

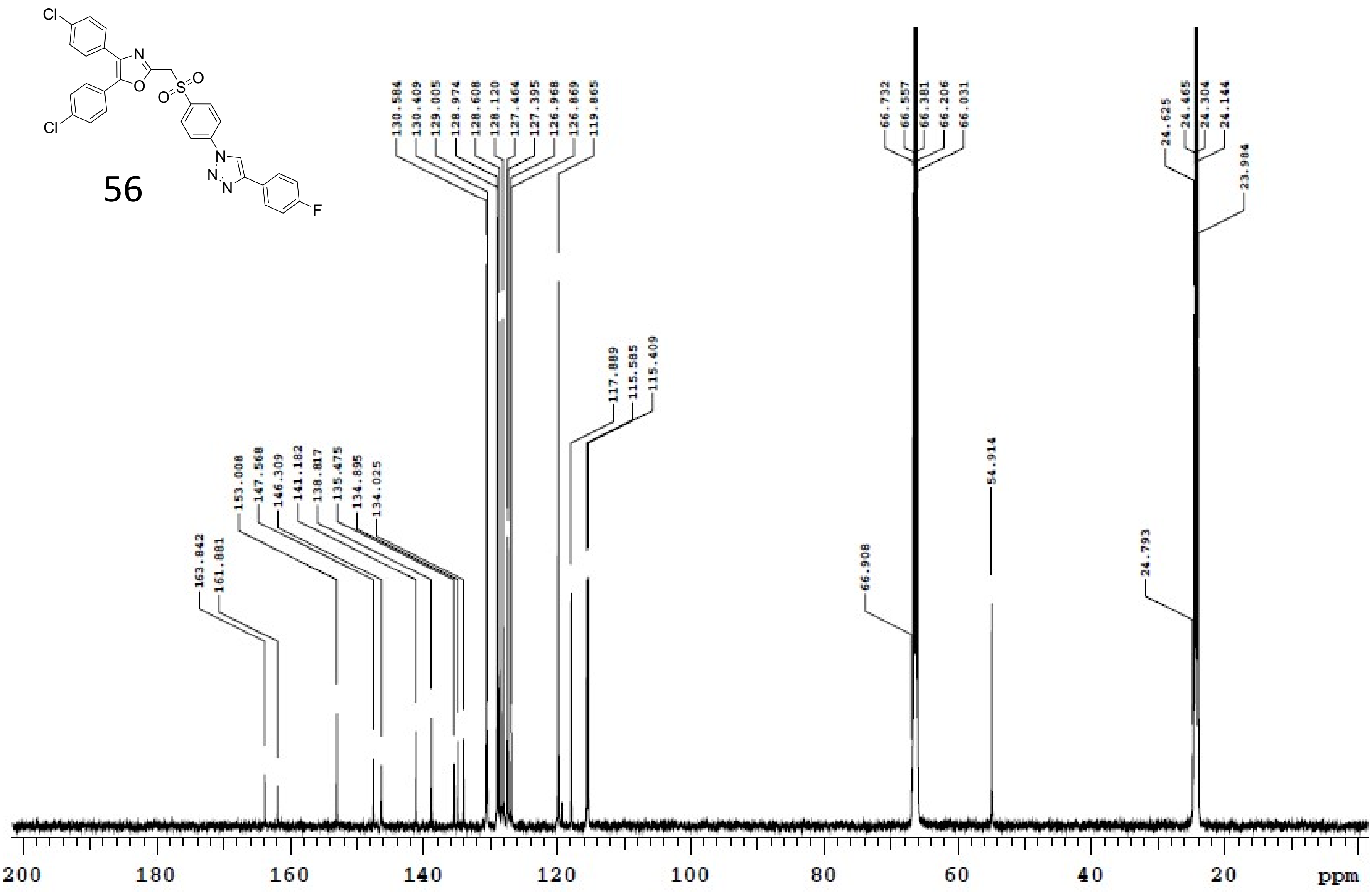

56

^{1}H NMR: 4,5-bis(4-chlorophenyl)-2-(((4-(4-(2-methoxyphenyl)-1H-1,2,3-triazol-1-yl)phenyl)sulfonyl)methyl)oxazole

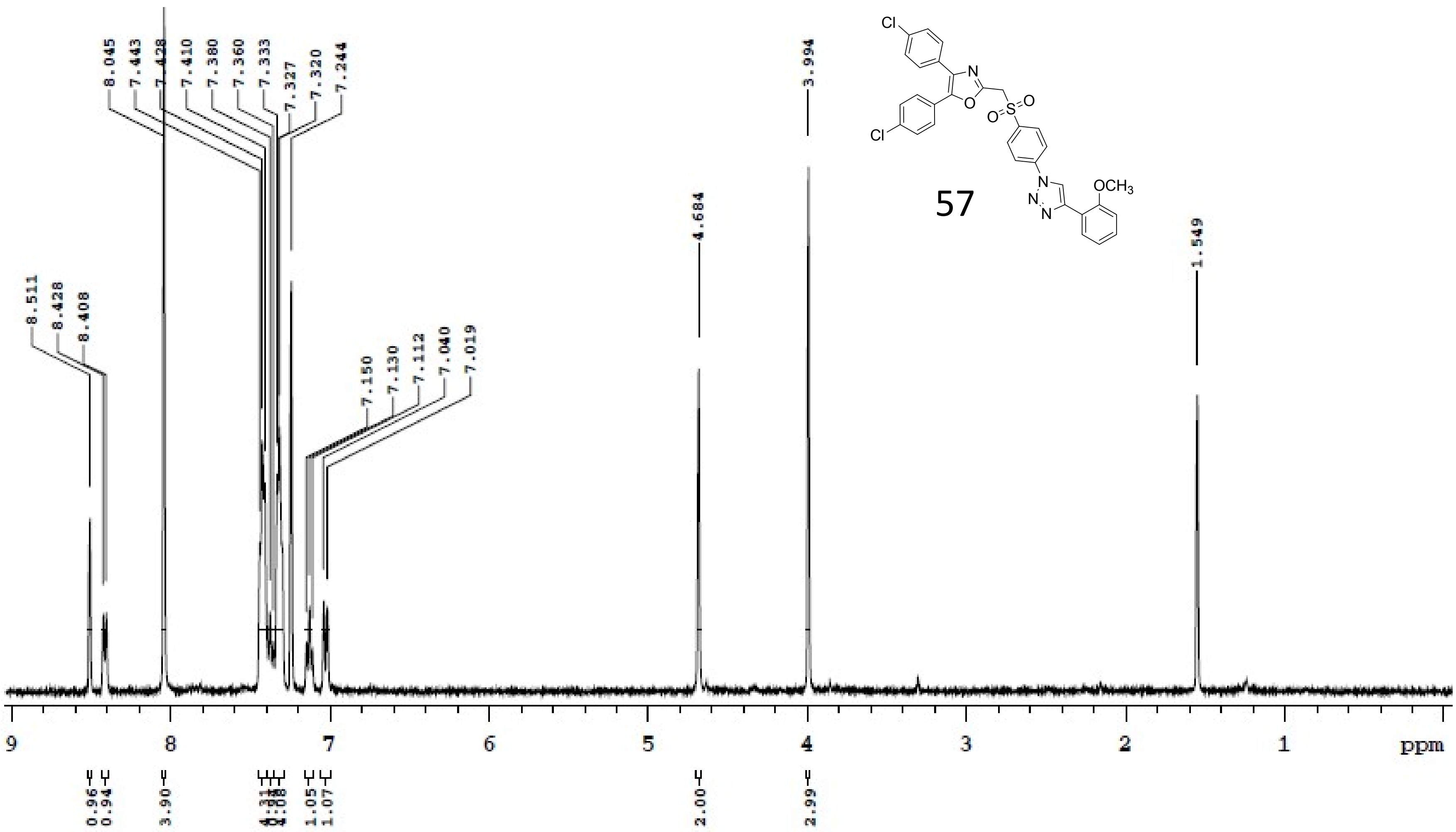

^{13}C NMR: 4,5-bis(4-chlorophenyl)-2-(((4-(4-(2-methoxyphenyl)-1H-1,2,3-triazol-1-yl)phenyl)sulfonyl)methyl)oxazole

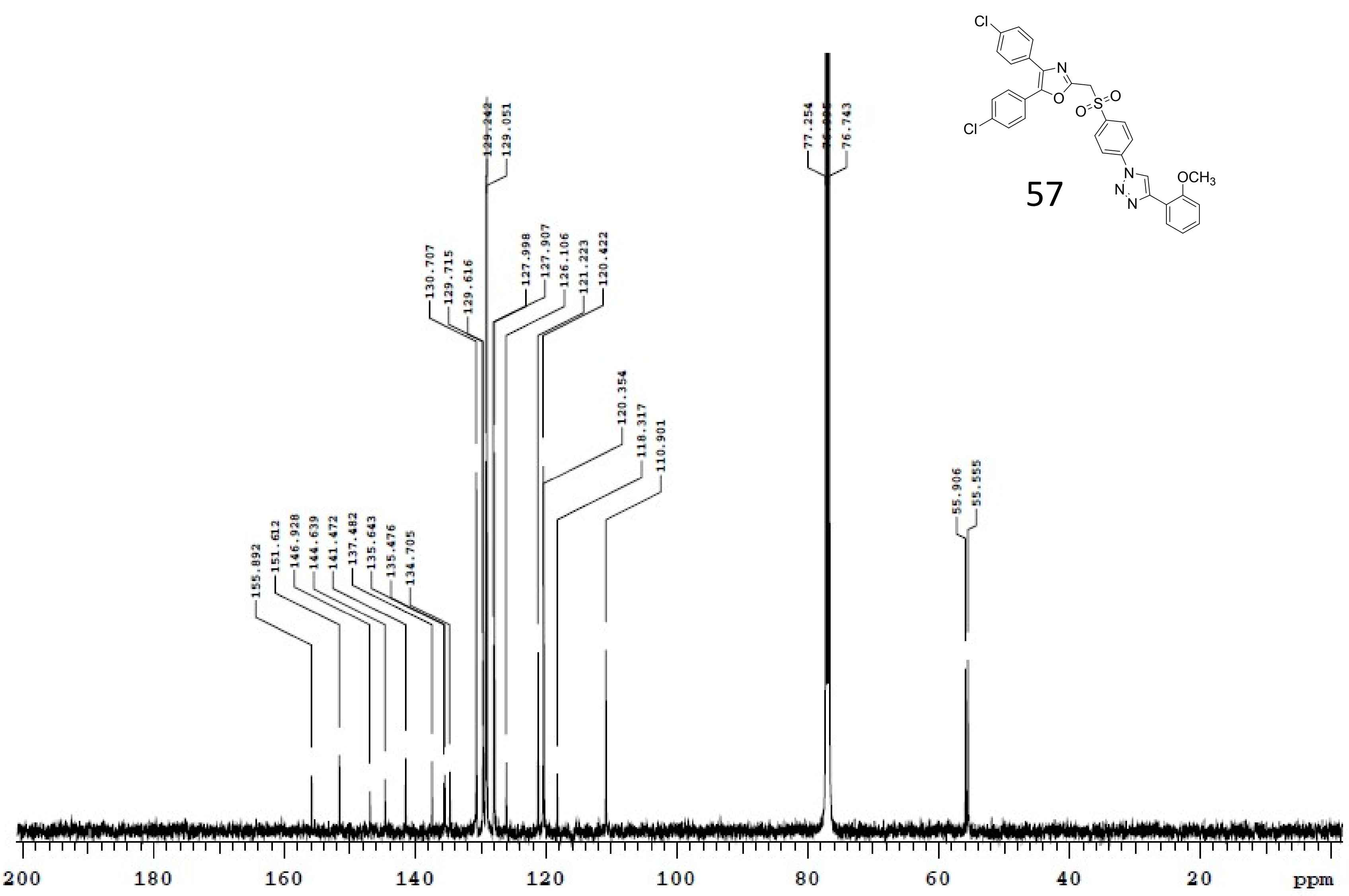

^{1}H NMR: 2-oxo-1,2-diphenylethyl 2-azidobenzoate

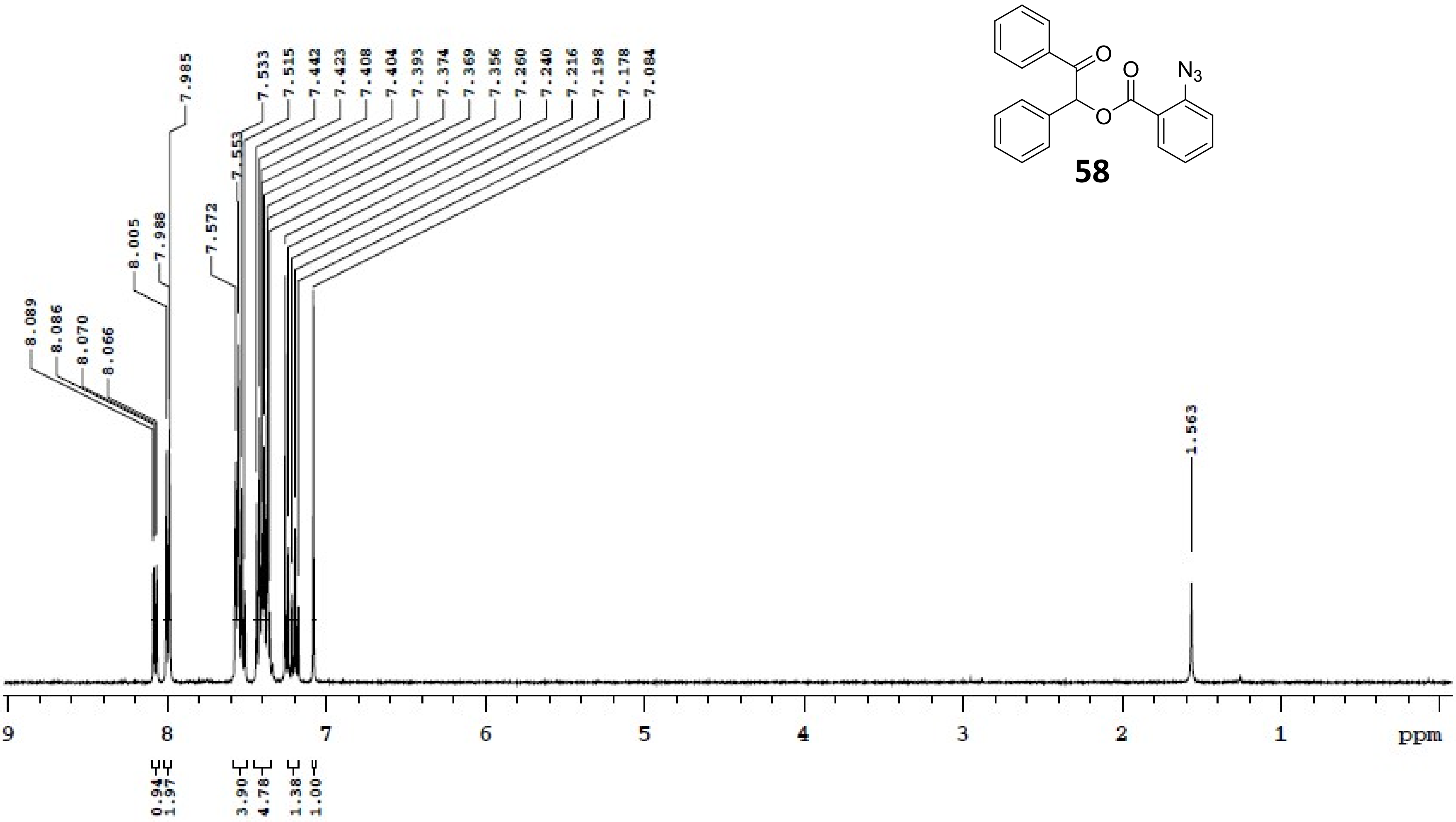

58

^{13}C NMR: 2-oxo-1,2-diphenylethyl 2-azidobenzoate

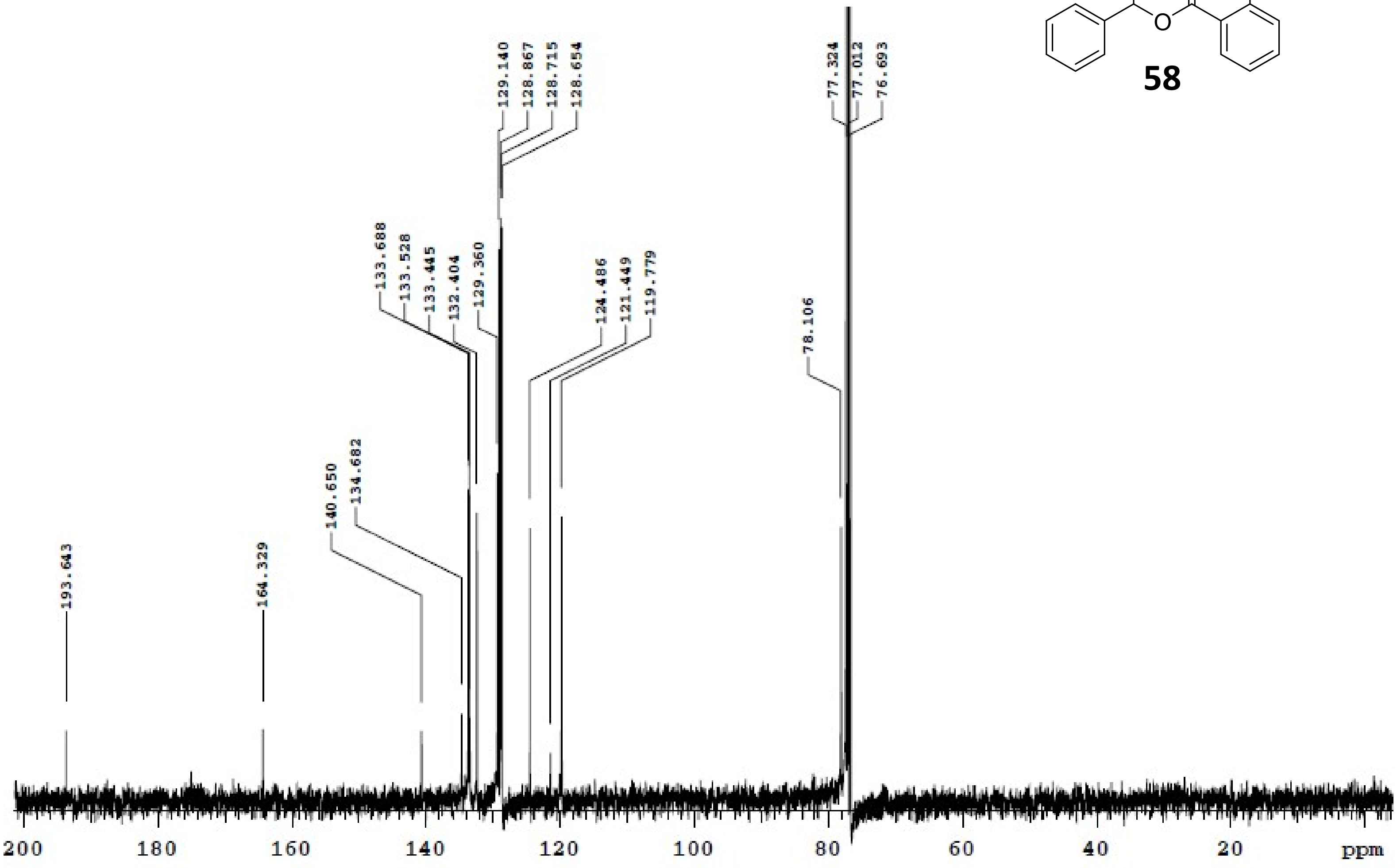

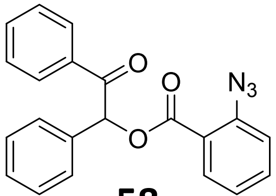

58

^{1}H NMR: 1,2-bis(4-chlorophenyl)-2-oxoethyl 2-azidobenzoate

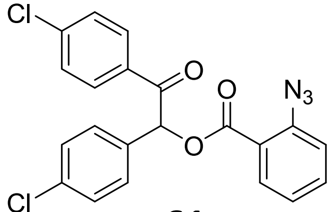

61

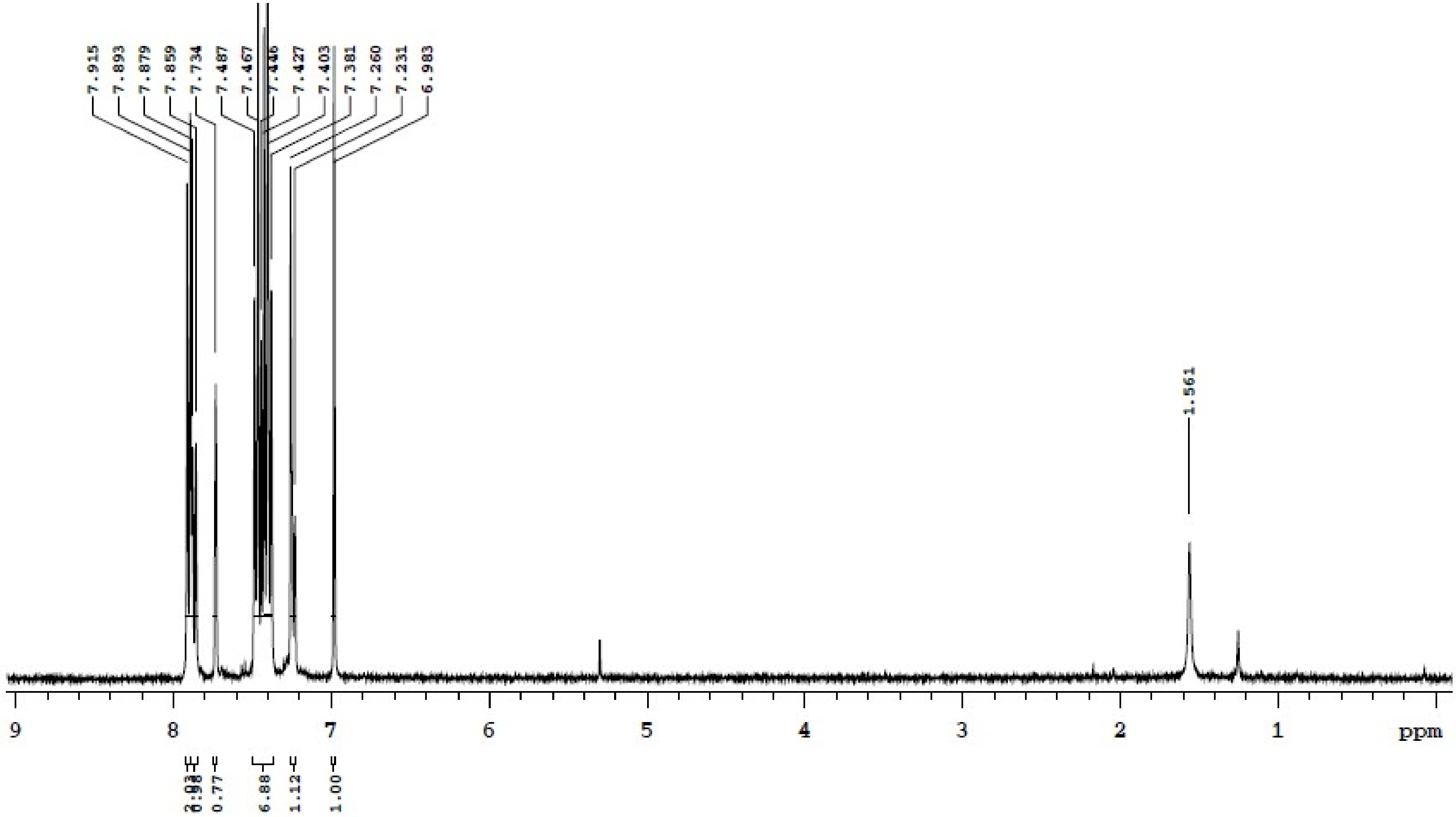

^{13}C NMR: 1,2-bis(4-chlorophenyl)-2-oxoethyl 2-azidobenzoate

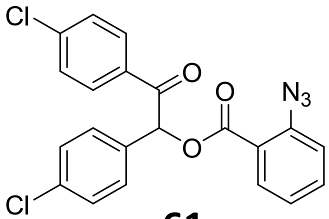

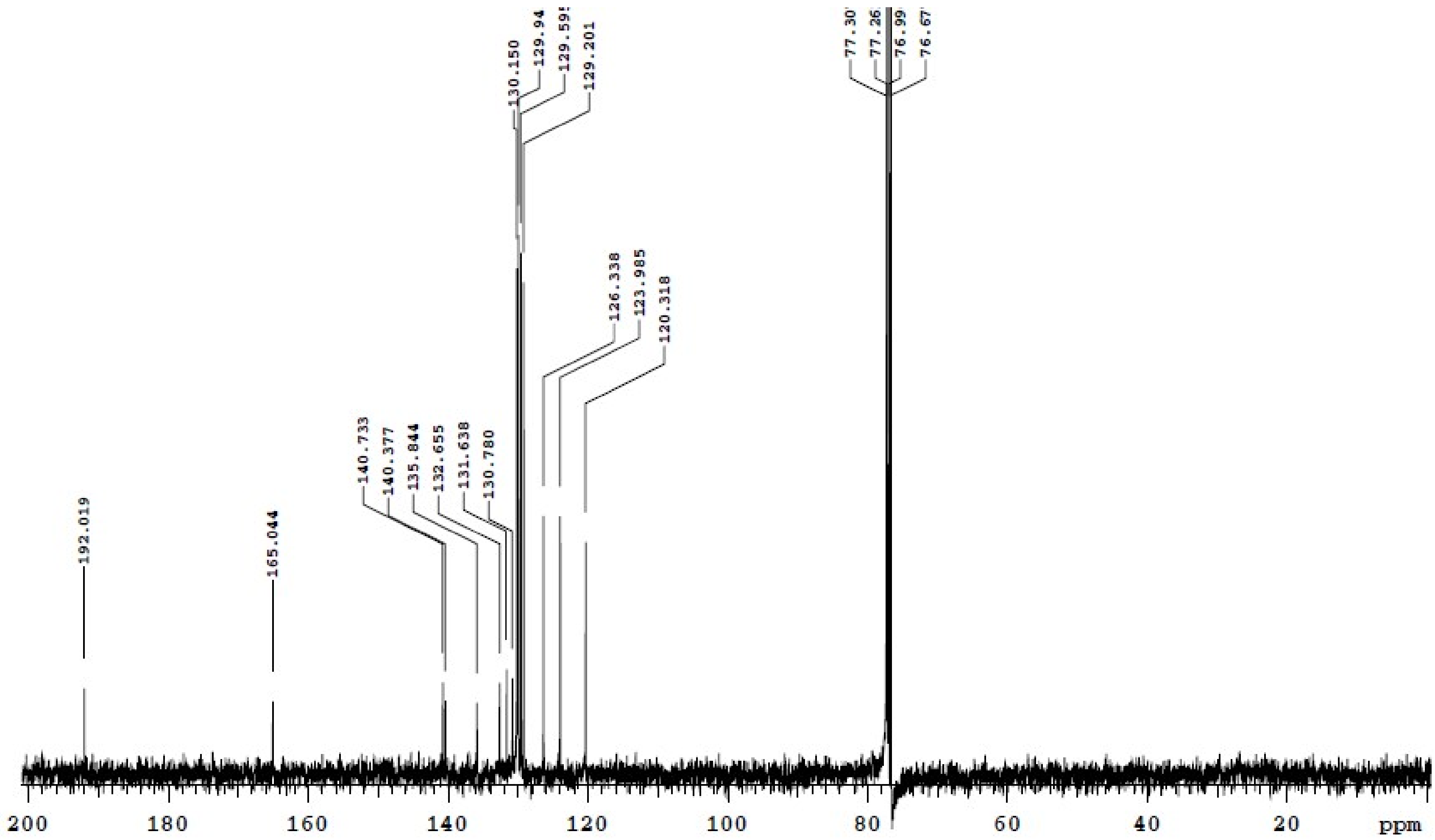

^{1}H NMR: 1,2-bis(4-fluorophenyl)-2-oxoethyl 2-azidobenzoate

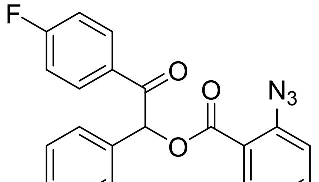

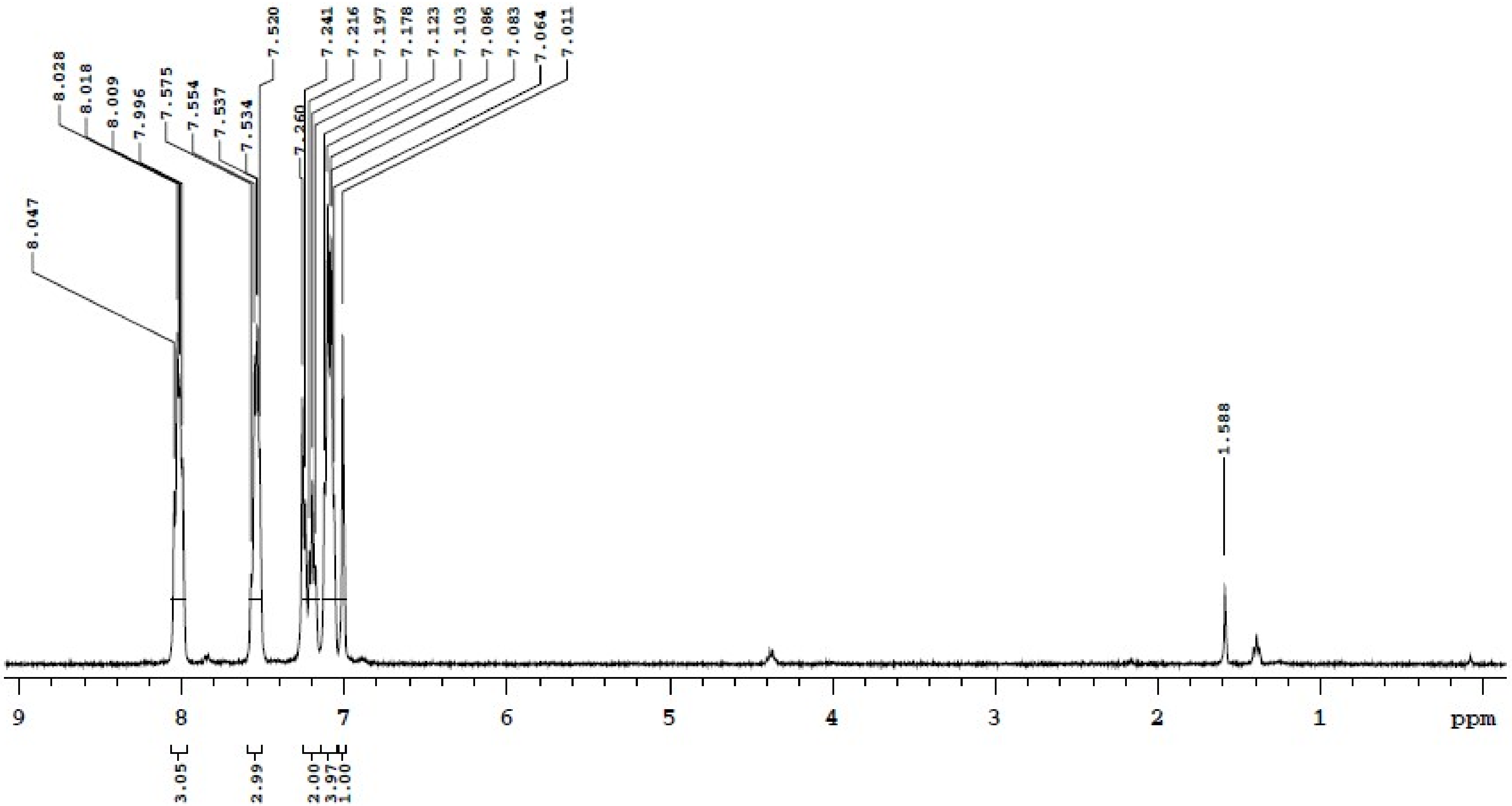

¹³C NMR: 1,2-bis(4-fluorophenyl)-2-oxoethyl 2-azidobenzoate

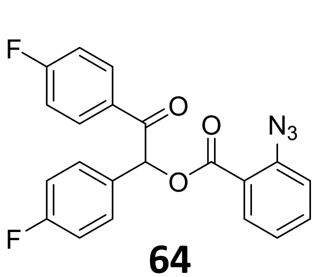

64

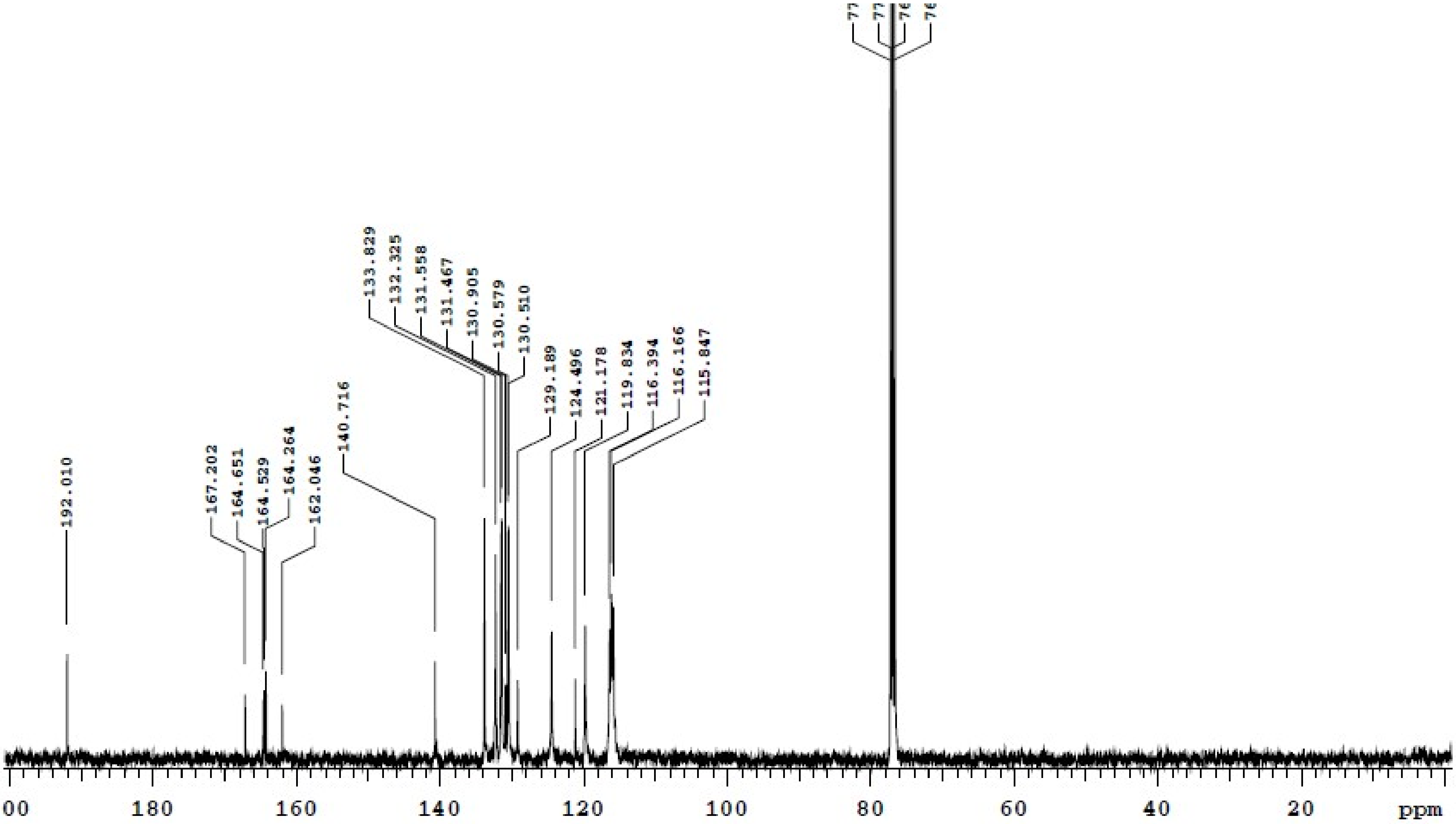

¹H NMR: 1,2-bis(4-methoxyphenyl)-2-oxoethyl 2-azidobenzoate

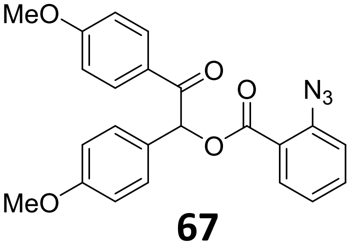

67

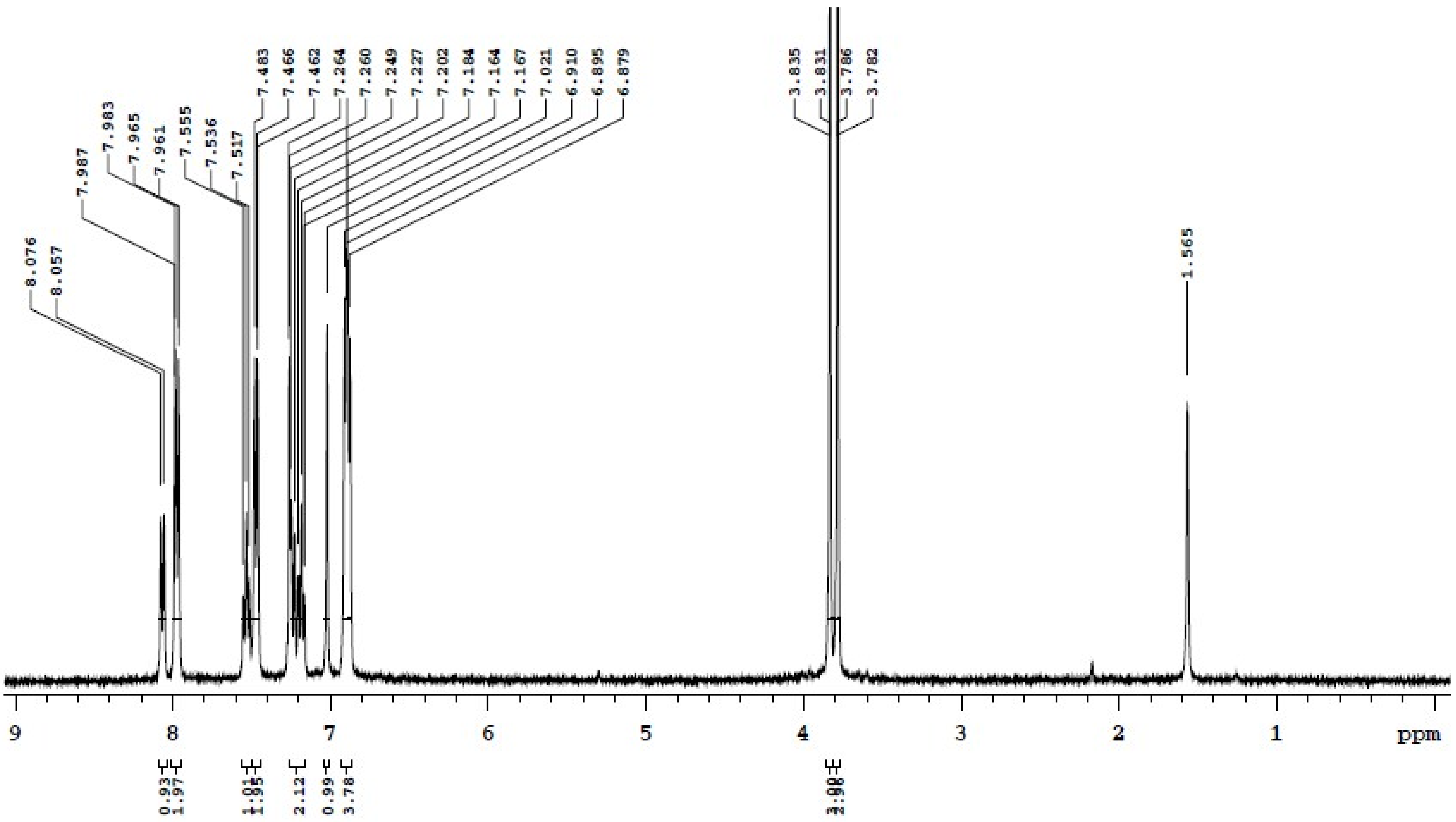

^{13}C NMR: 1,2-bis(4-methoxyphenyl)-2-oxoethyl 2-azidobenzoate

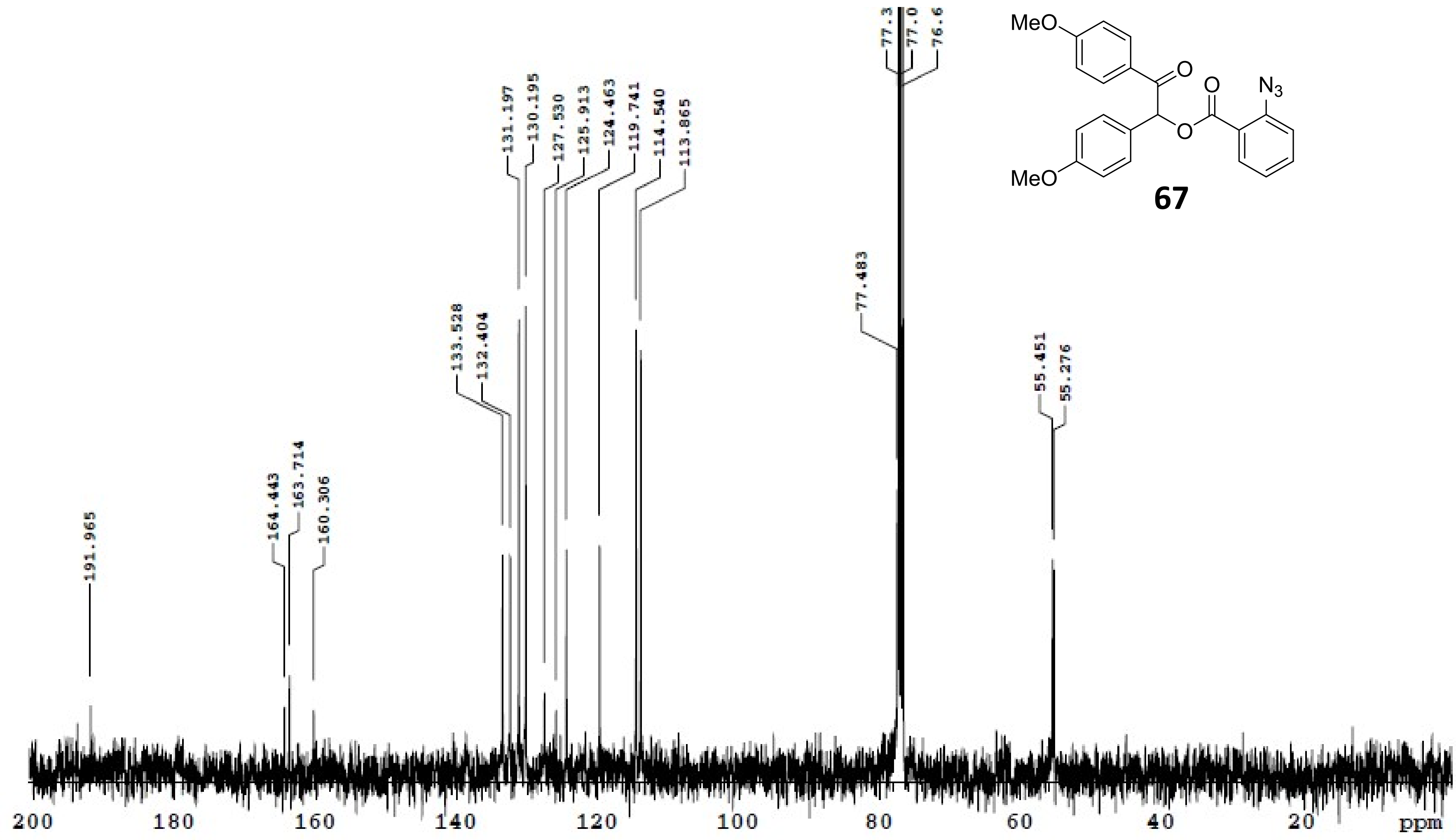

67

^{1}H NMR: 1,2-di(furan-2-yl)-2-oxoethyl 2-azidobenzoate

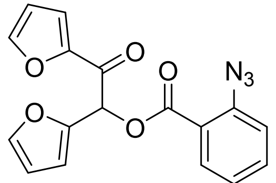

70

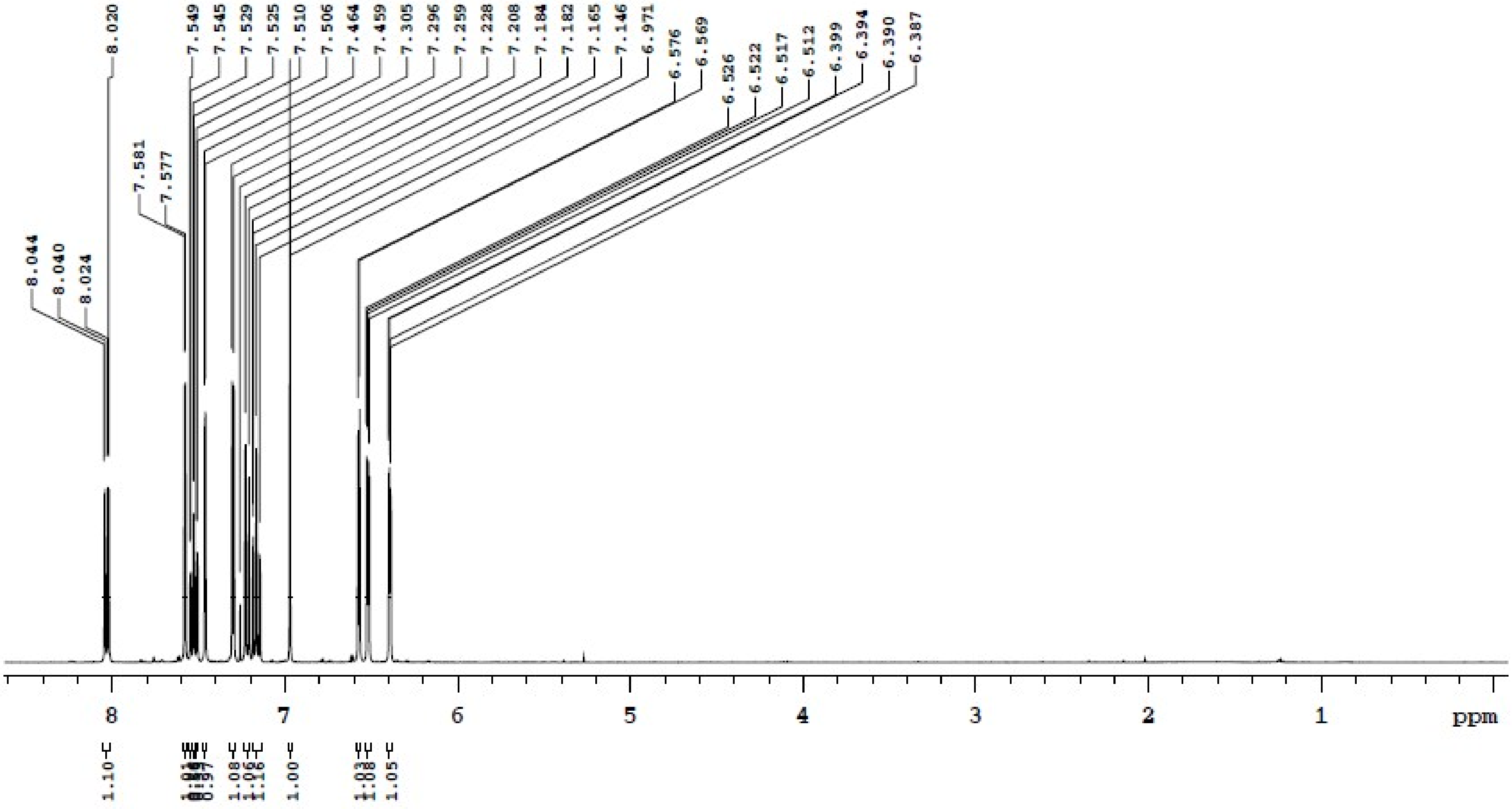

^{13}C NMR: 1,2-di(furan-2-yl)-2-oxoethyl 2-azidobenzoate

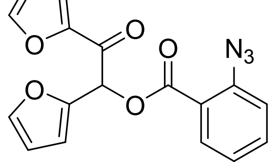

70

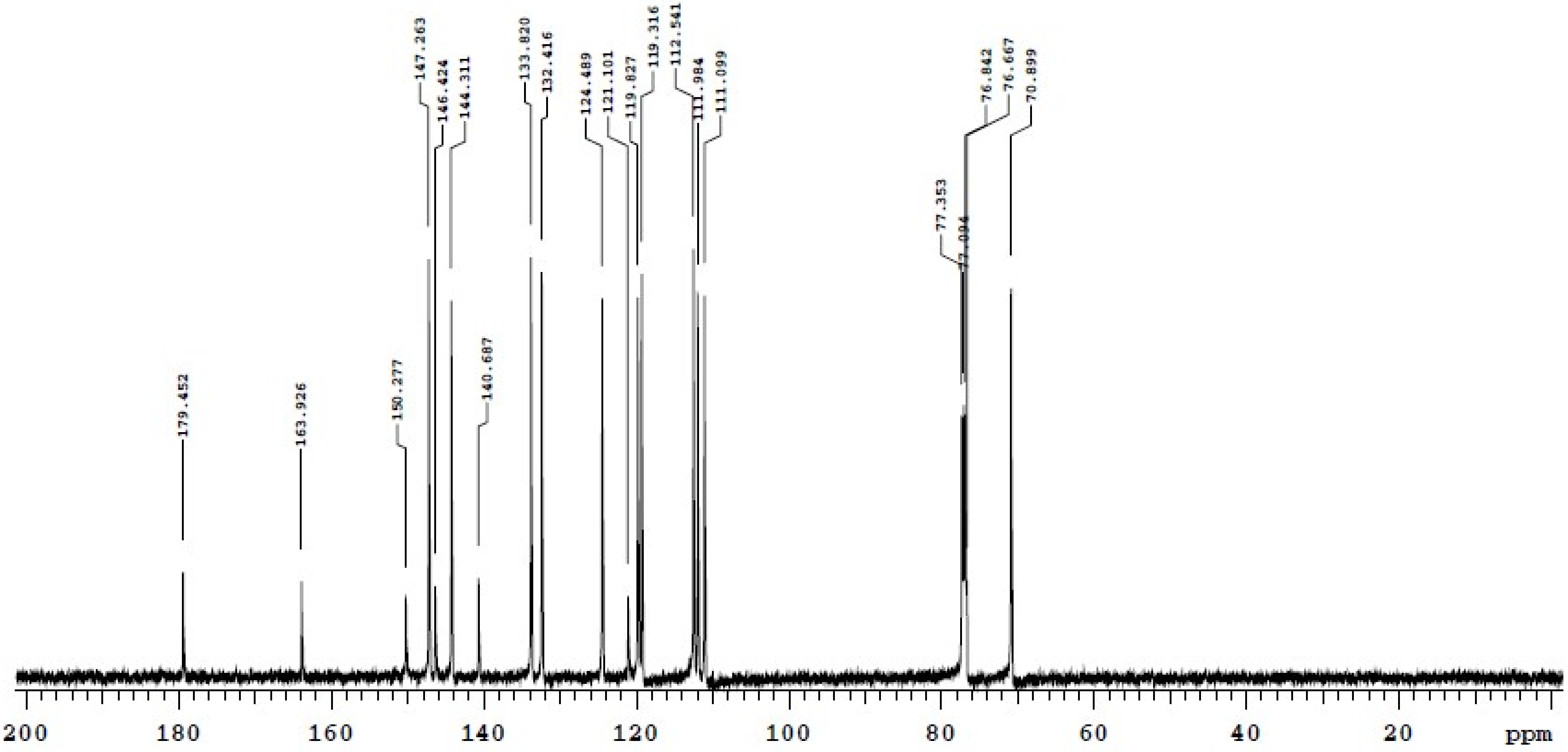

^{1}H NMR: 1,2-di(furan-2-yl)-2-oxoethyl 3-azidobenzoate

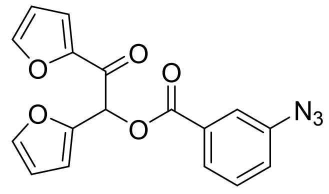

71

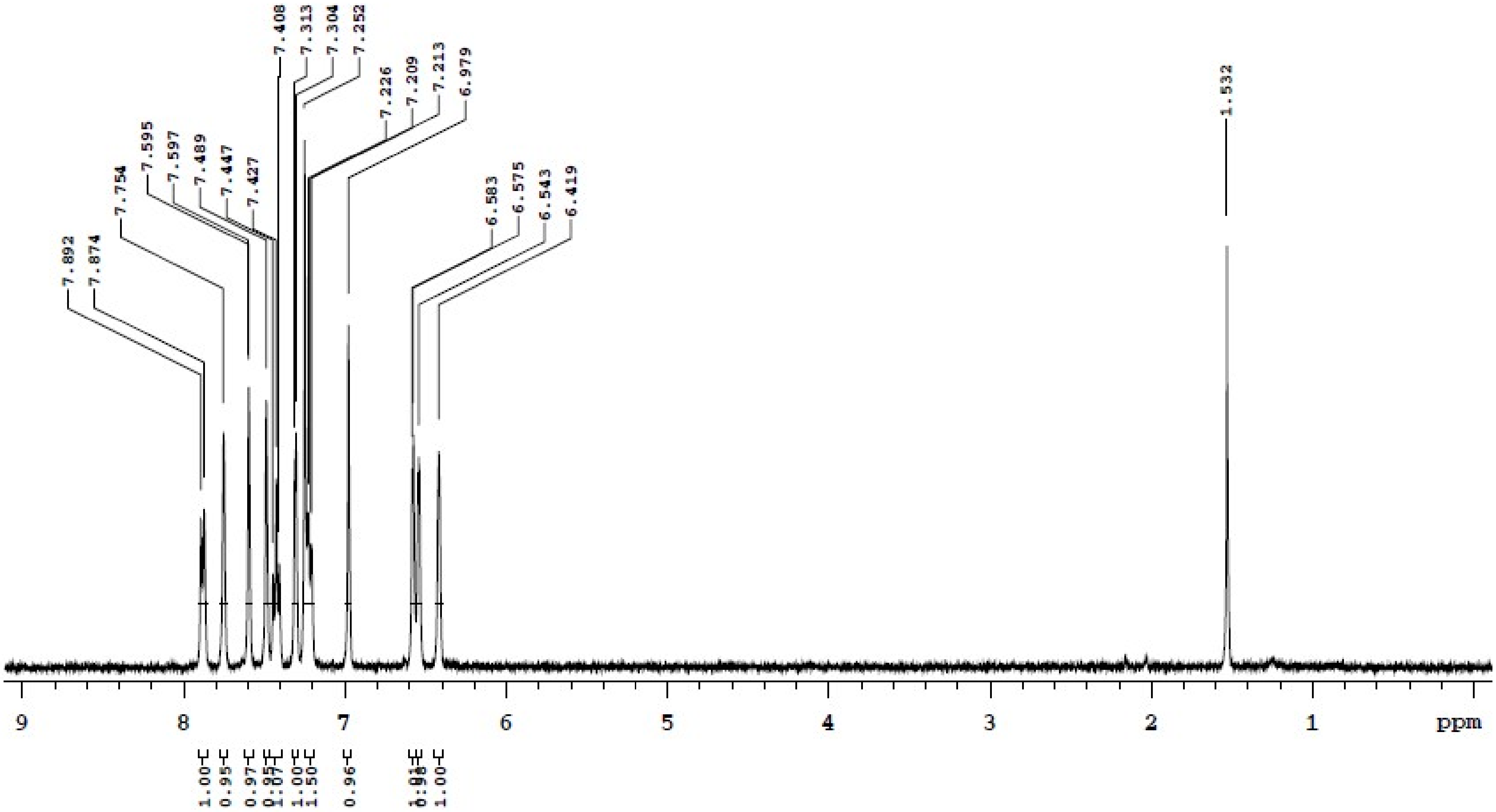

^{13}C NMR: 1,2-di(furan-2-yl)-2-oxoethyl 3-azidobenzoate

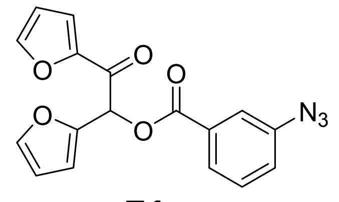

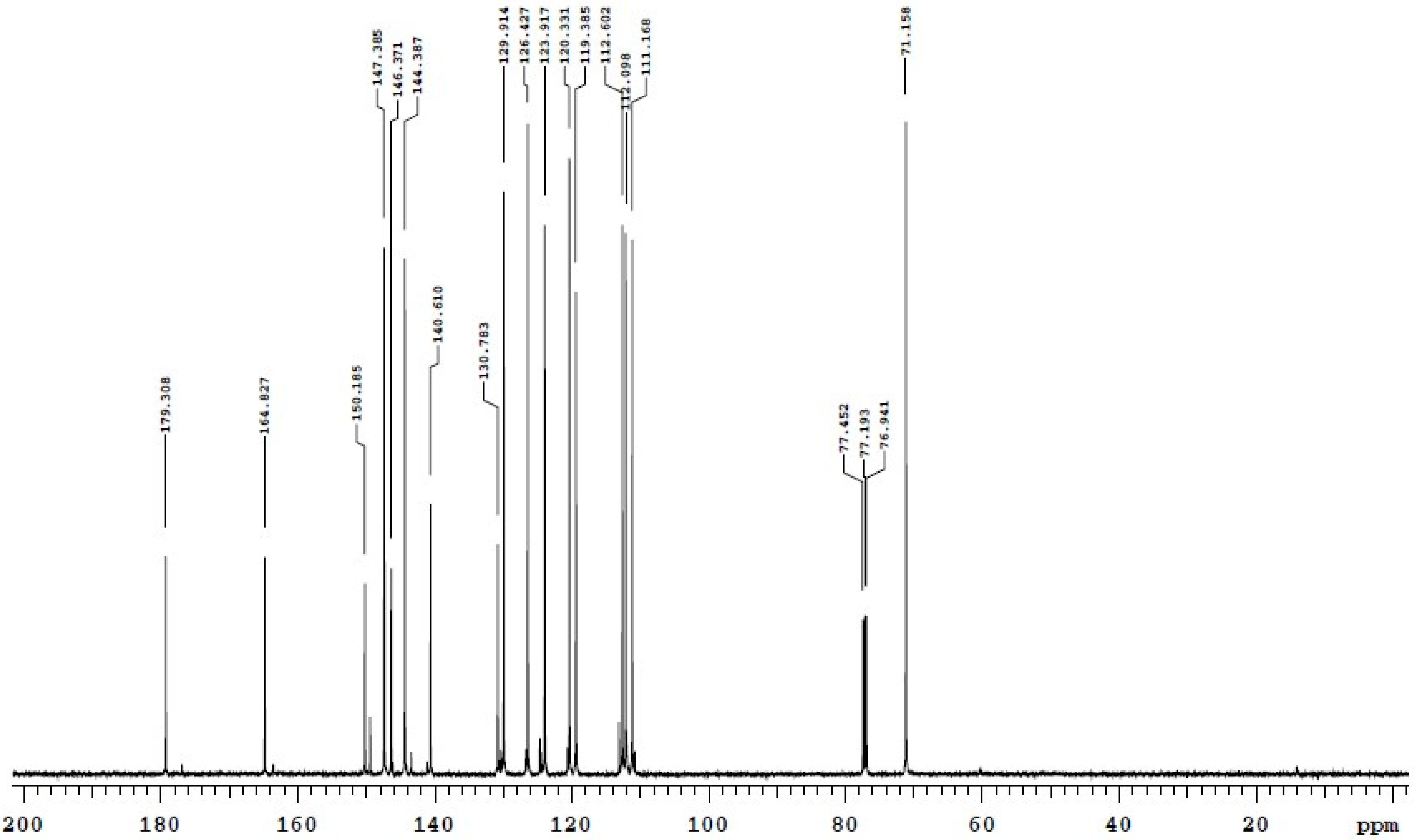

^{1}H NMR: 1,2-di(furan-2-yl)-2-oxoethyl 4-azidobenzoate

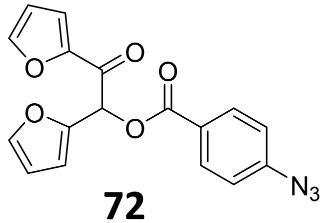

72

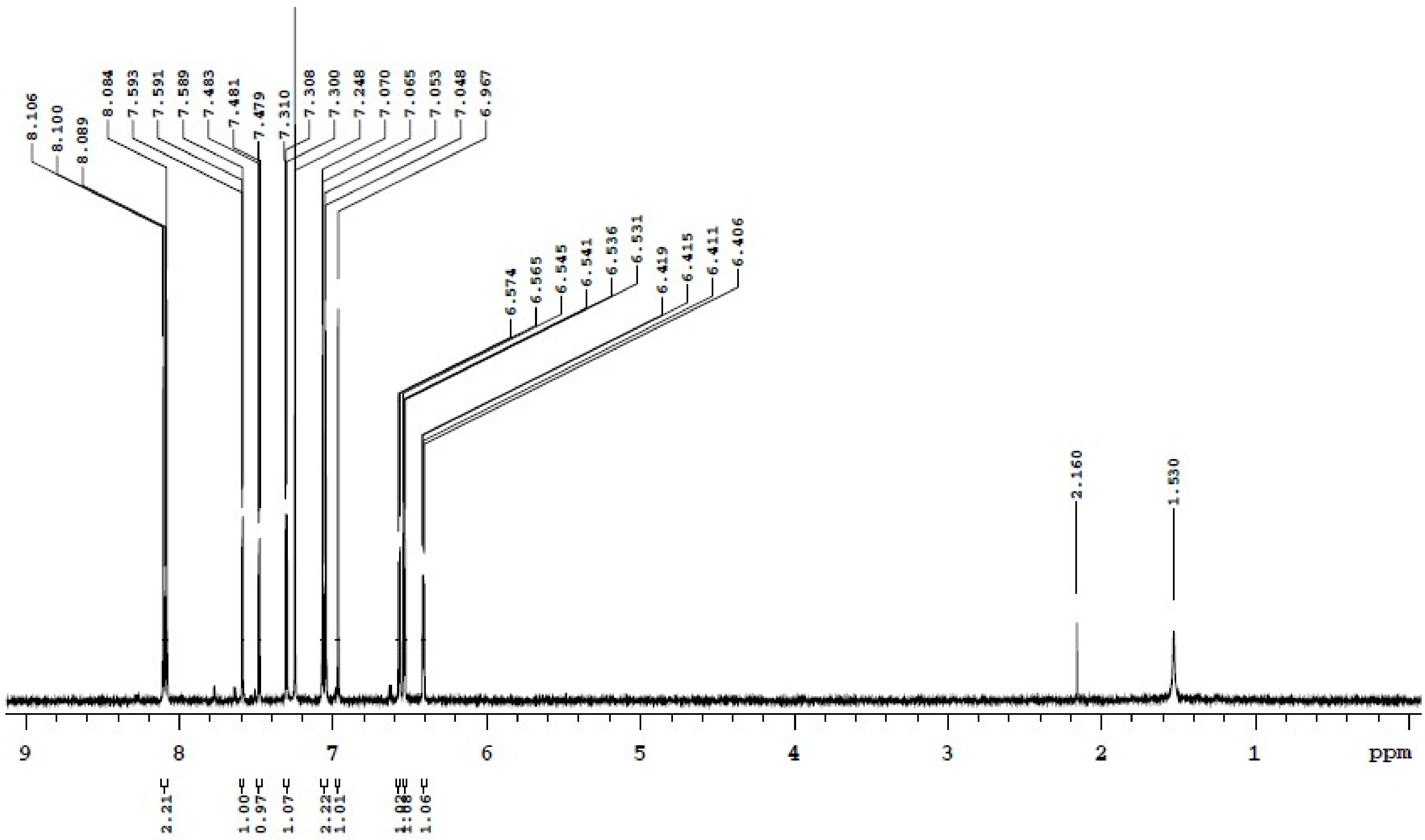

^{13}C NMR: 1,2-di(furan-2-yl)-2-oxoethyl 4-azidobenzoate

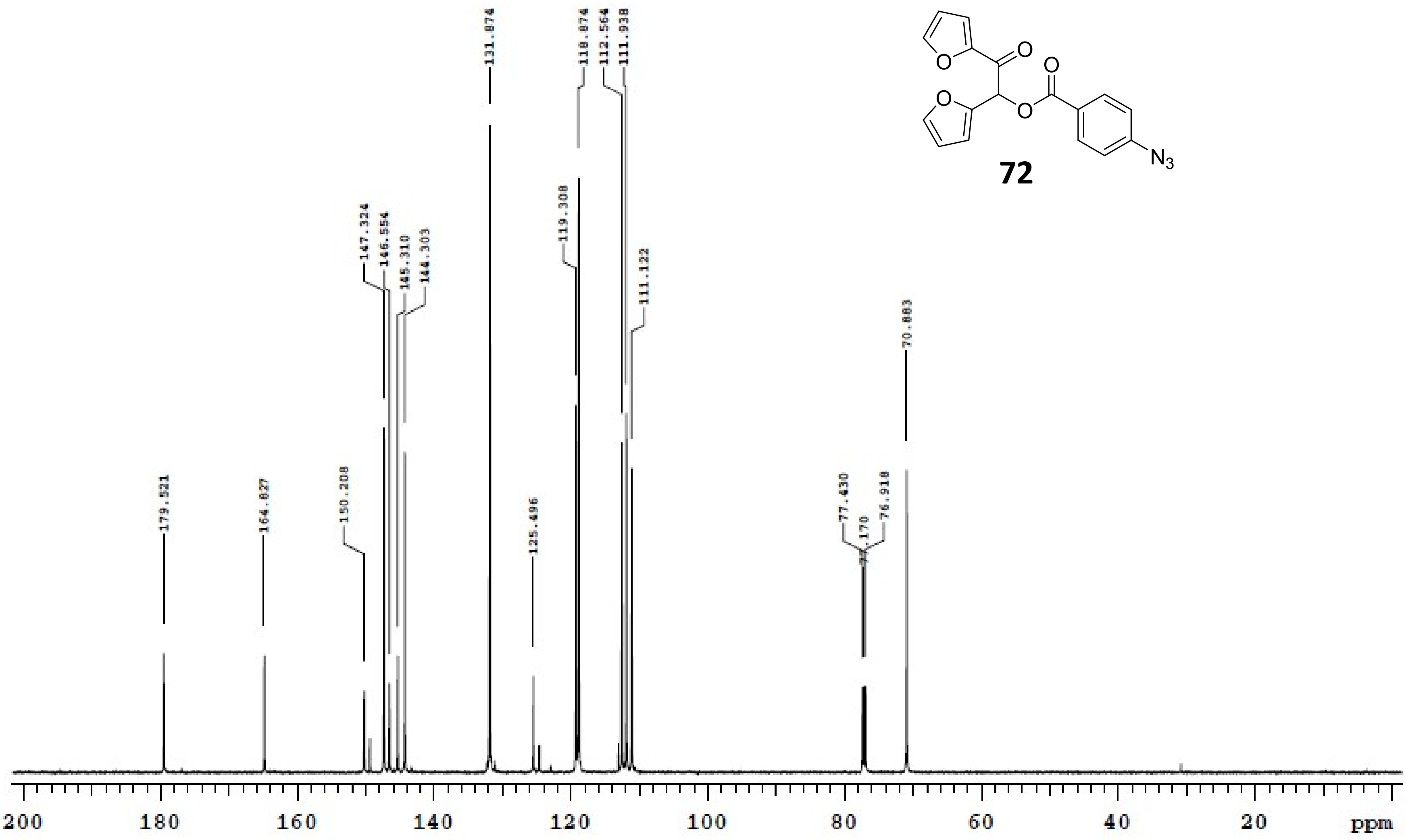

72

^{1}H NMR: 2-(2-azidophenyl)-4,5-diphenyloxazole

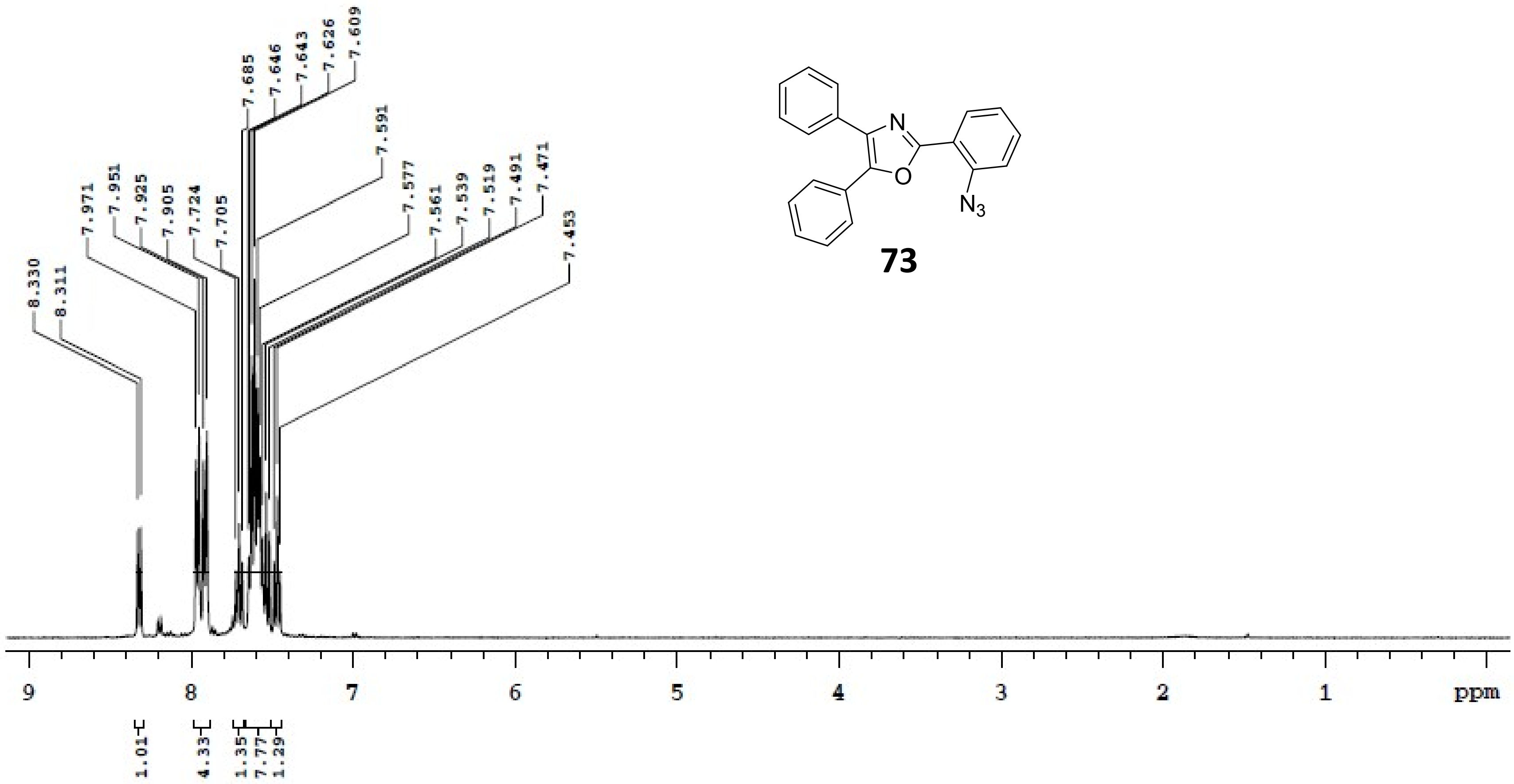

73

^{13}C NMR: 2-(2-azidophenyl)-4,5-diphenyloxazole

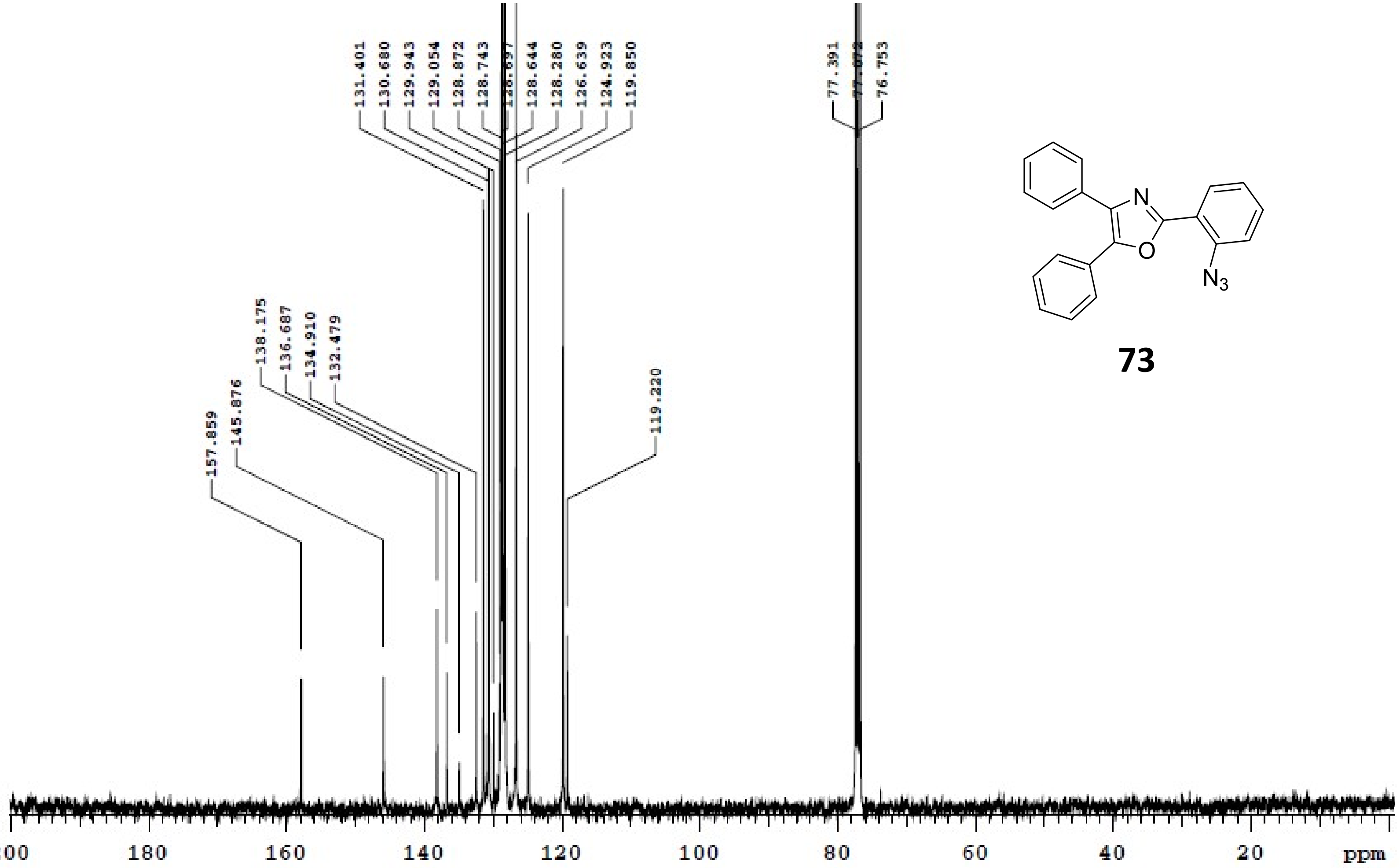

73

^{1}H NMR: 2-(2-azidophenyl)-4,5-bis(4-chlorophenyl)oxazole

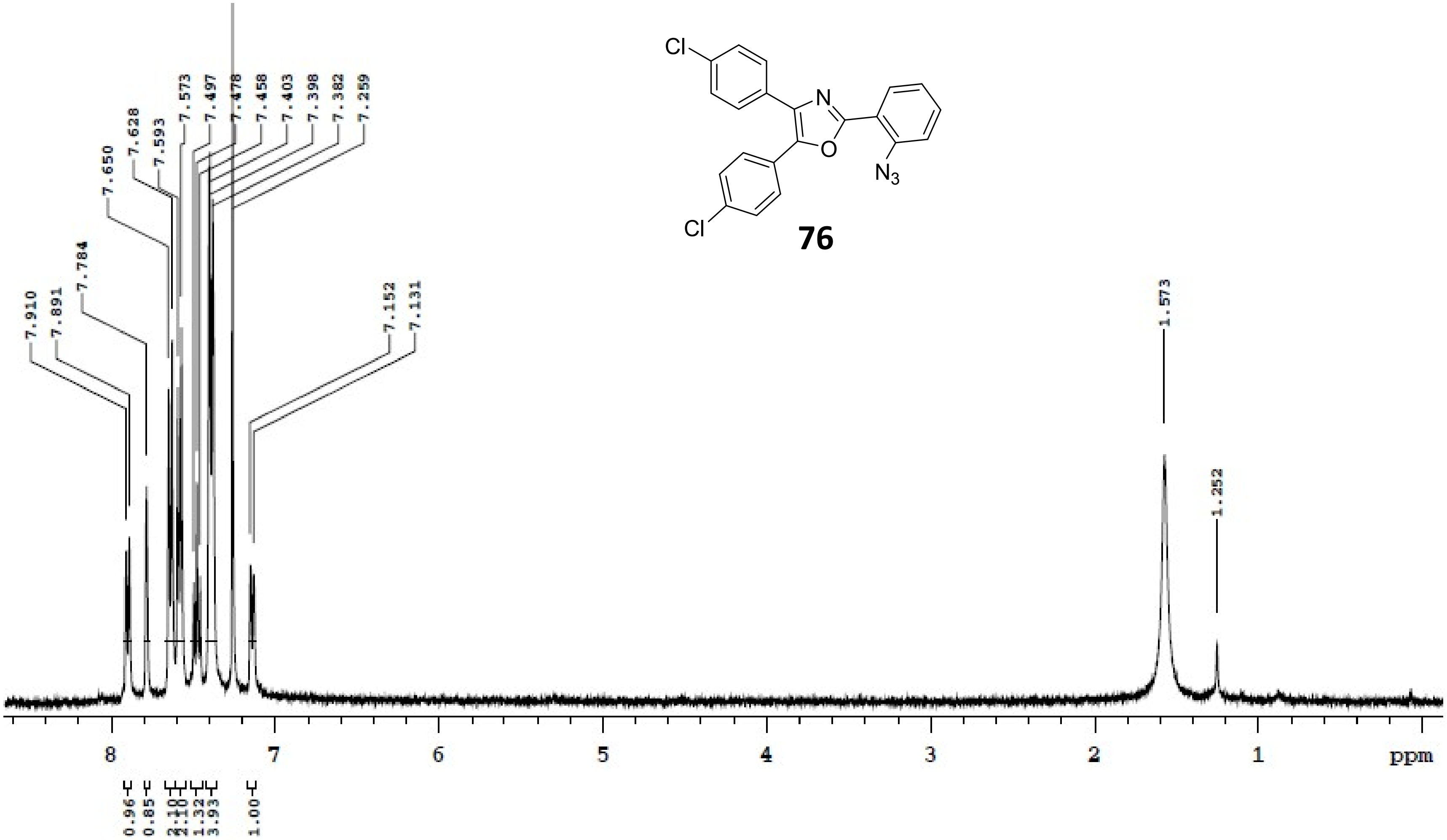

^{13}C NMR: 2-(2-azidophenyl)-4,5-bis(4-chlorophenyl)oxazole

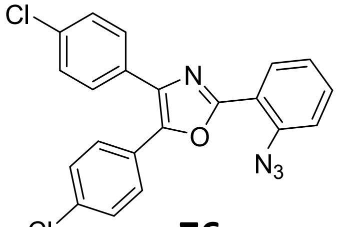

76

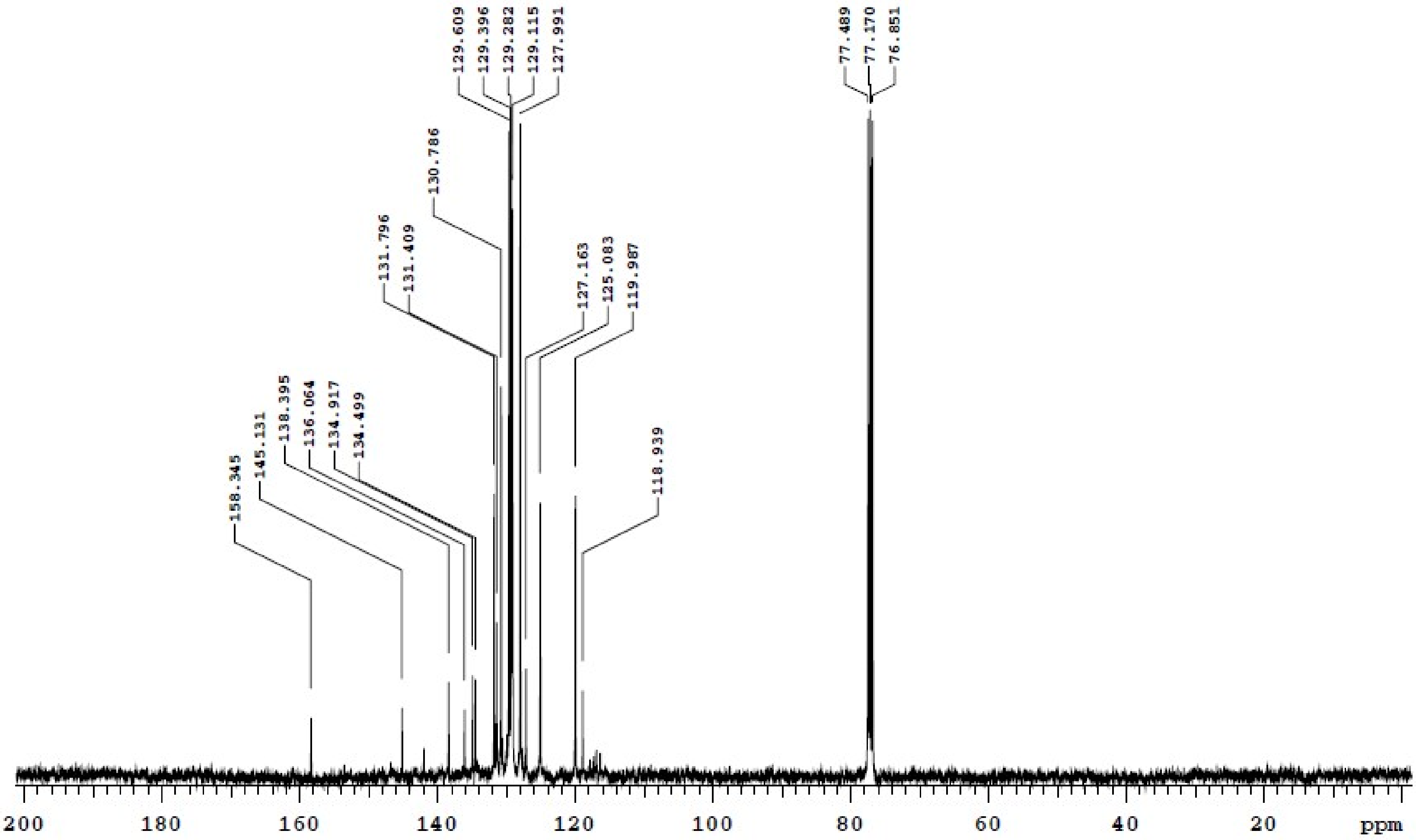

^{1}H NMR: 2-(2-azidophenyl)-4,5-bis(4-fluorophenyl)oxazole

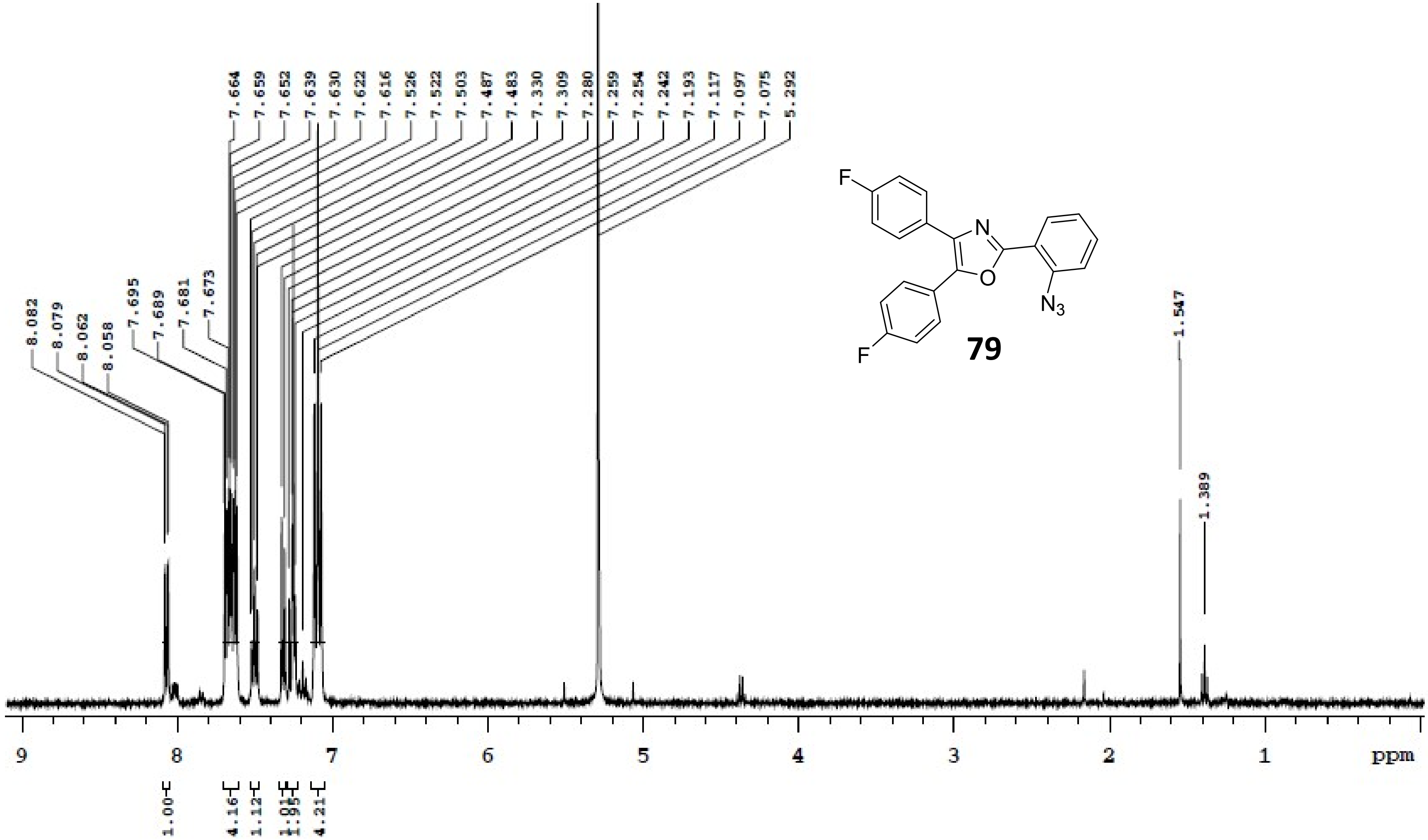

^{13}C NMR: 2-(2-azidophenyl)-4,5-bis(4-fluorophenyl)oxazole

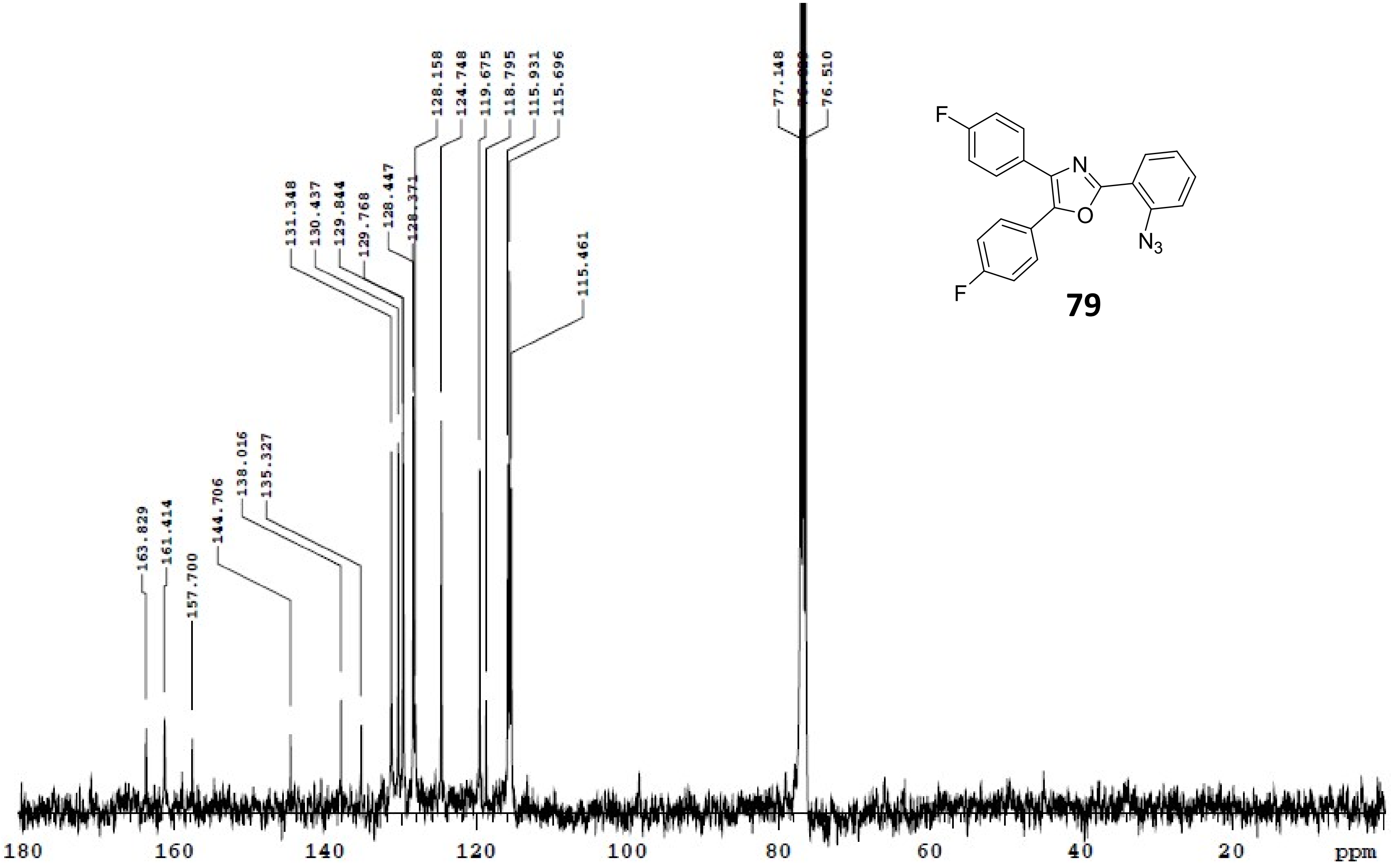

79

^{1}H NMR: 2-(2-azidophenyl)-4,5-bis(4-methoxyphenyl)oxazole

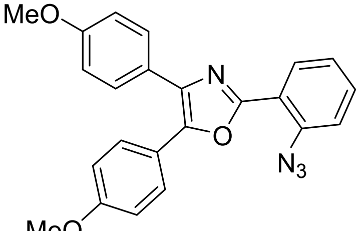

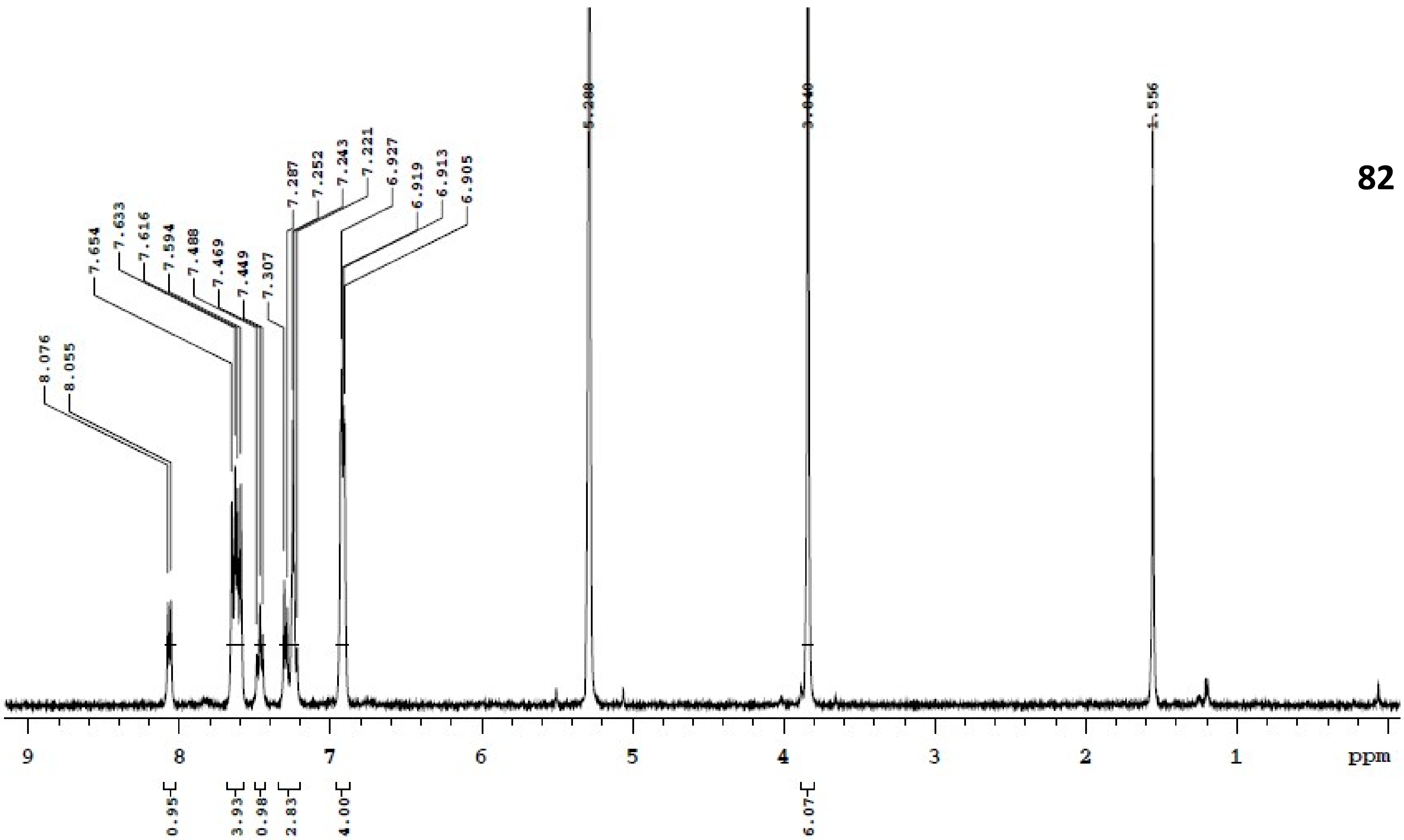

^{13}C NMR: 2-(2-azidophenyl)-4,5-bis(4-methoxyphenyl)oxazole

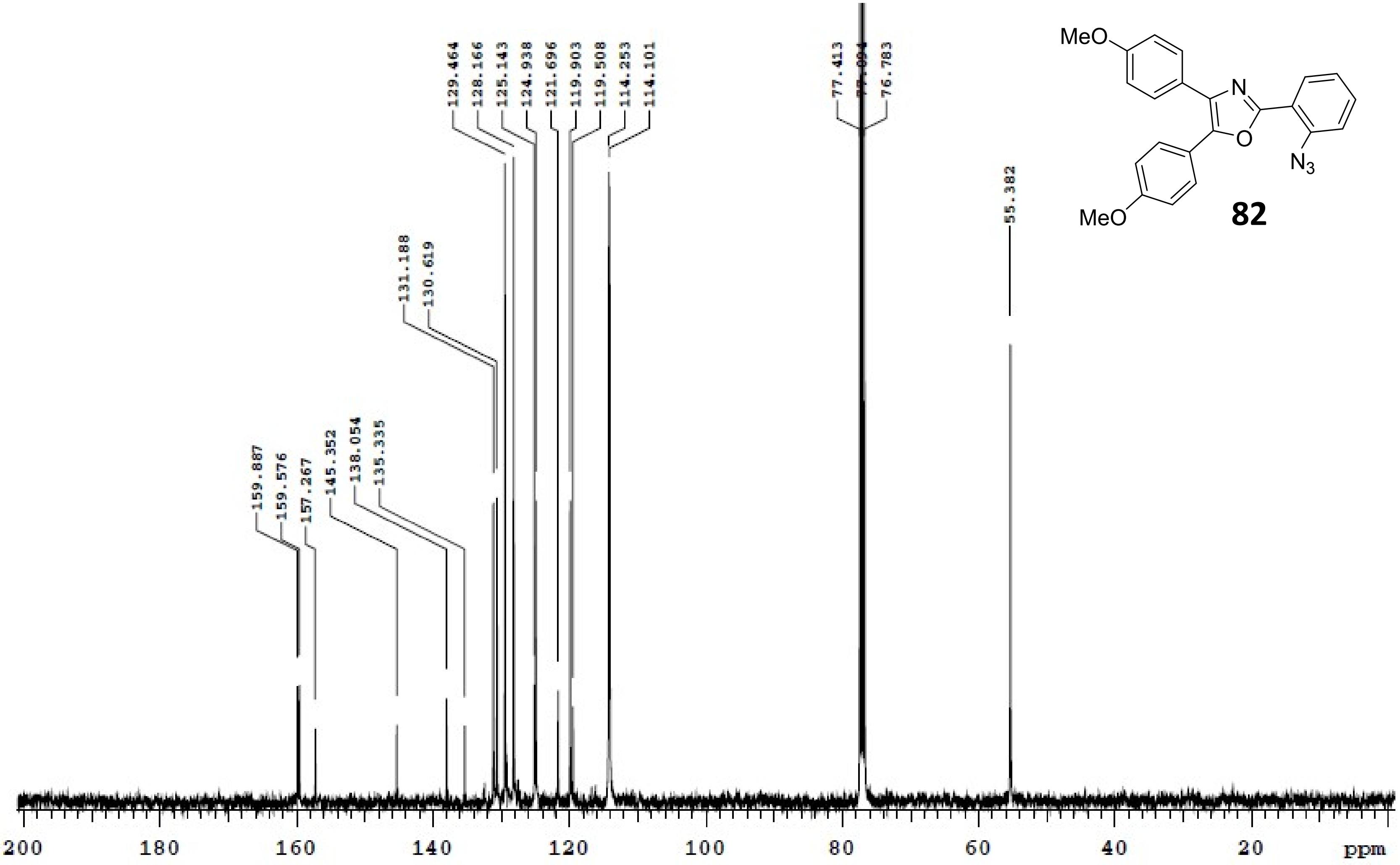

82

^{1}H NMR: 2-(2-azidophenyl)-4,5-di(furan-2-yl)oxazole

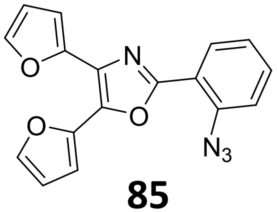

85

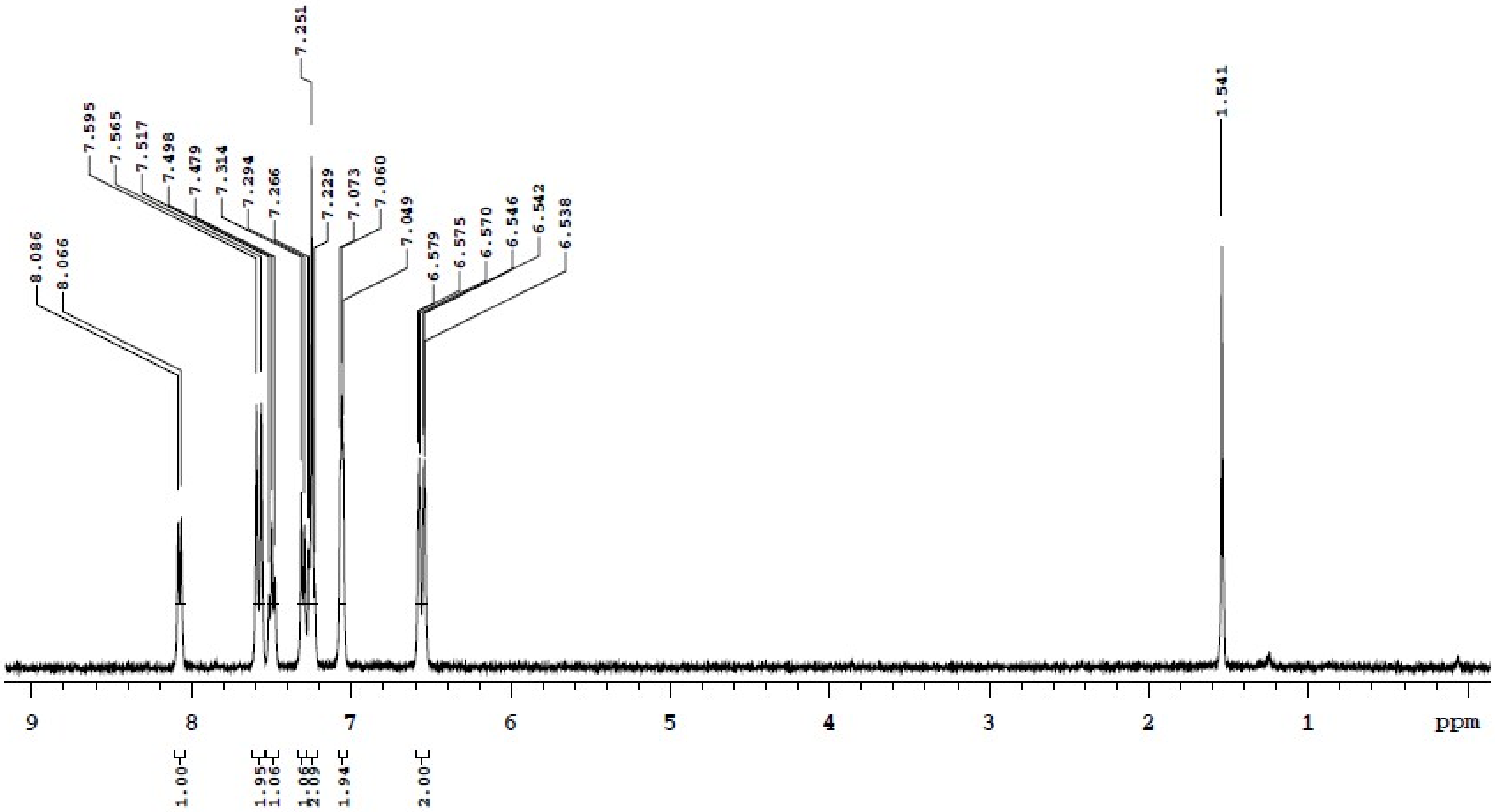

8.086
8.066
7.595
7.565
7.517
7.498
7.479
7.314
7.294
7.266
7.251
7.229
7.073
7.060
7.049
6.579
6.575
6.570
6.546
6.542
6.538
1.541
9
8
7
6
5
4
3
2
1
ppm
1.00
1.95
1.06
1.08
2.09
1.94
2.00

^{1}H NMR: 2-(2-azidophenyl)-4,5-di(furan-2-yl)oxazole

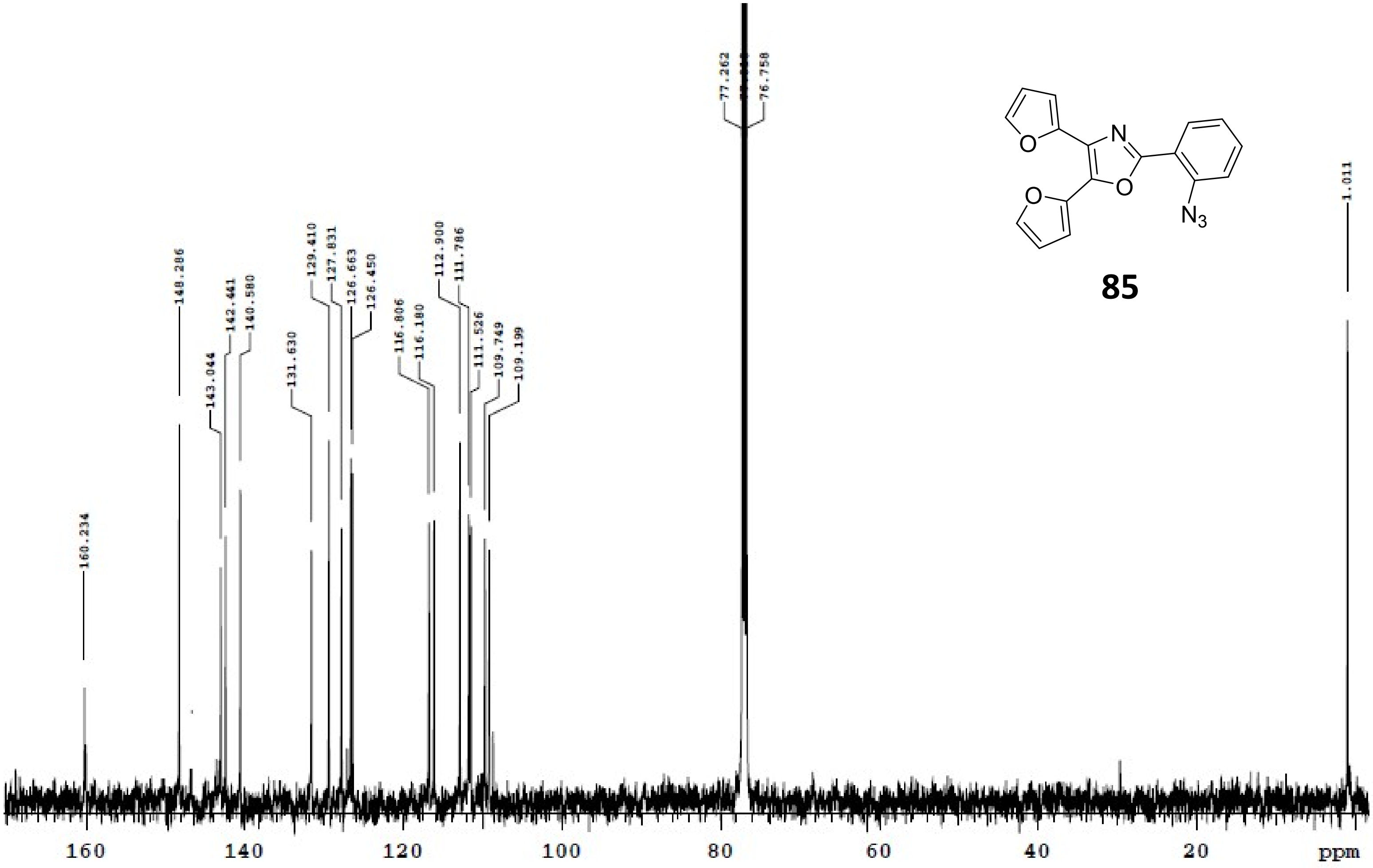

85

^{1}H NMR: 2-(3-azidophenyl)-4,5-di(furan-2-yl)oxazole

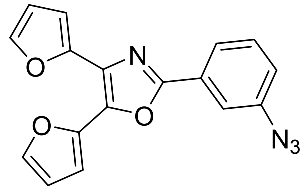

86

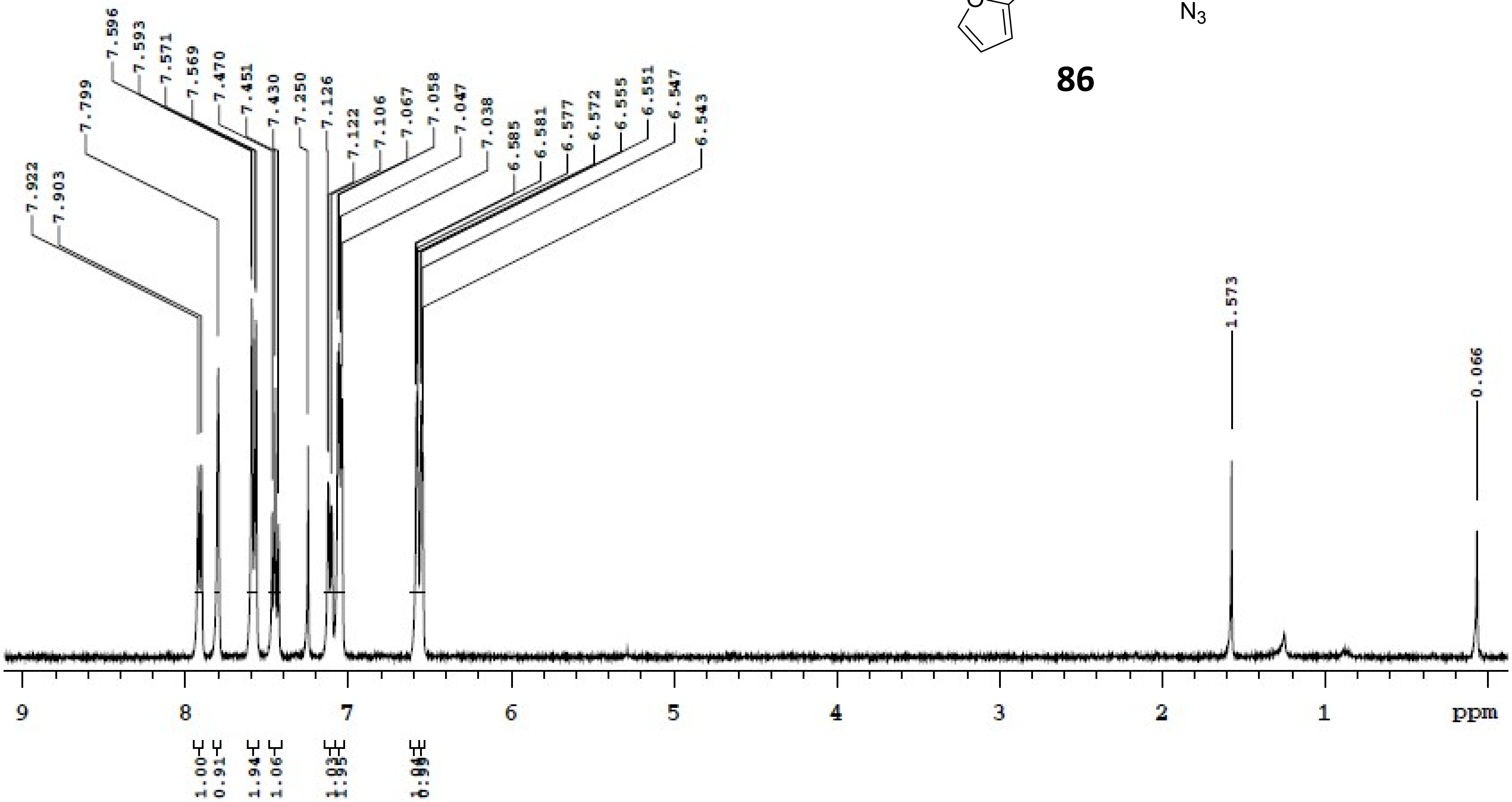

^{13}C NMR: 2-(3-azidophenyl)-4,5-di(furan-2-yl)oxazole

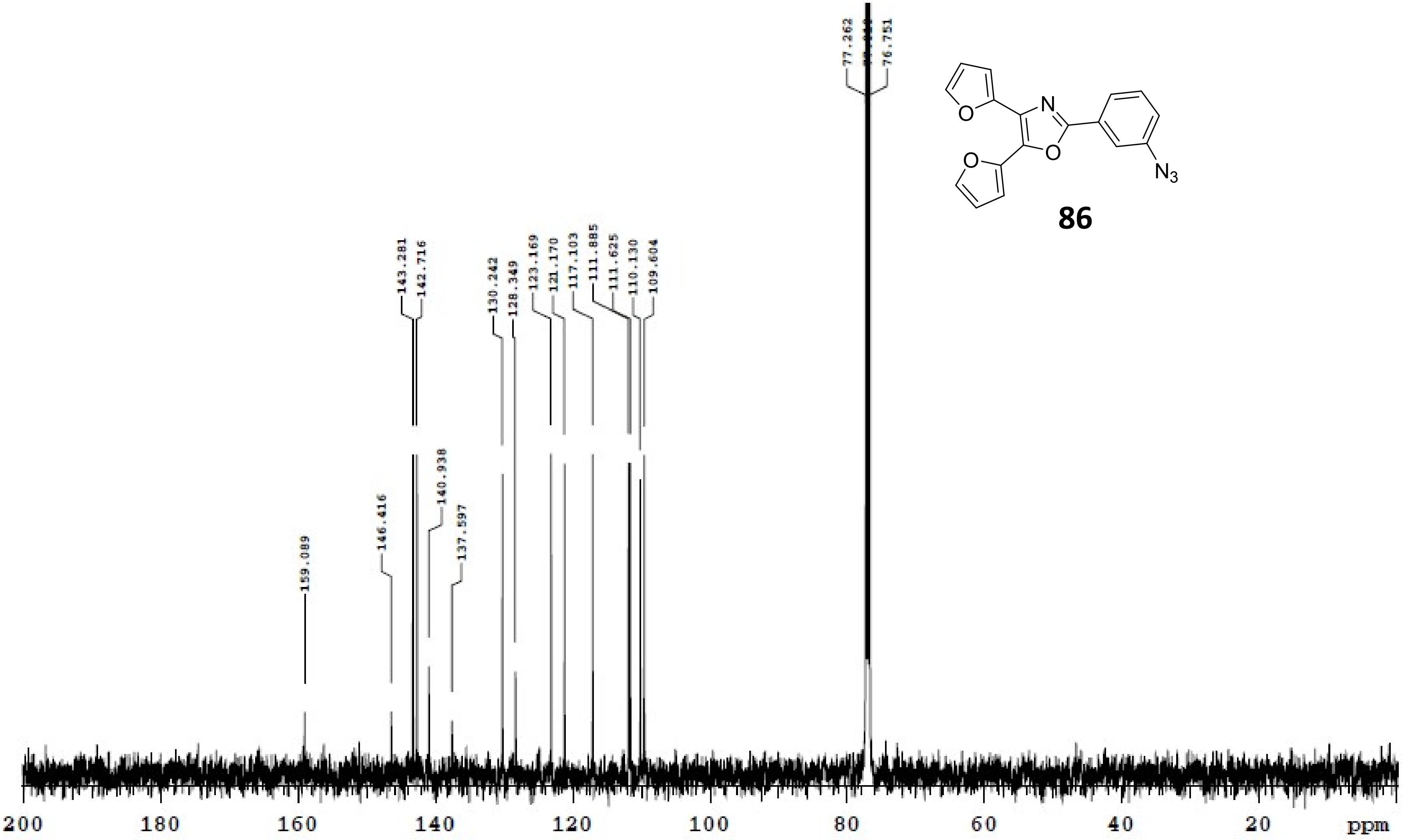

^{1}H NMR: 2-(4-azidophenyl)-4,5-di(furan-2-yl)oxazole

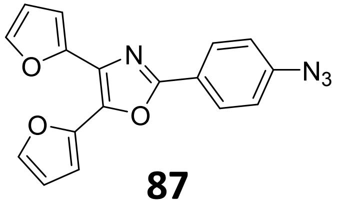

87

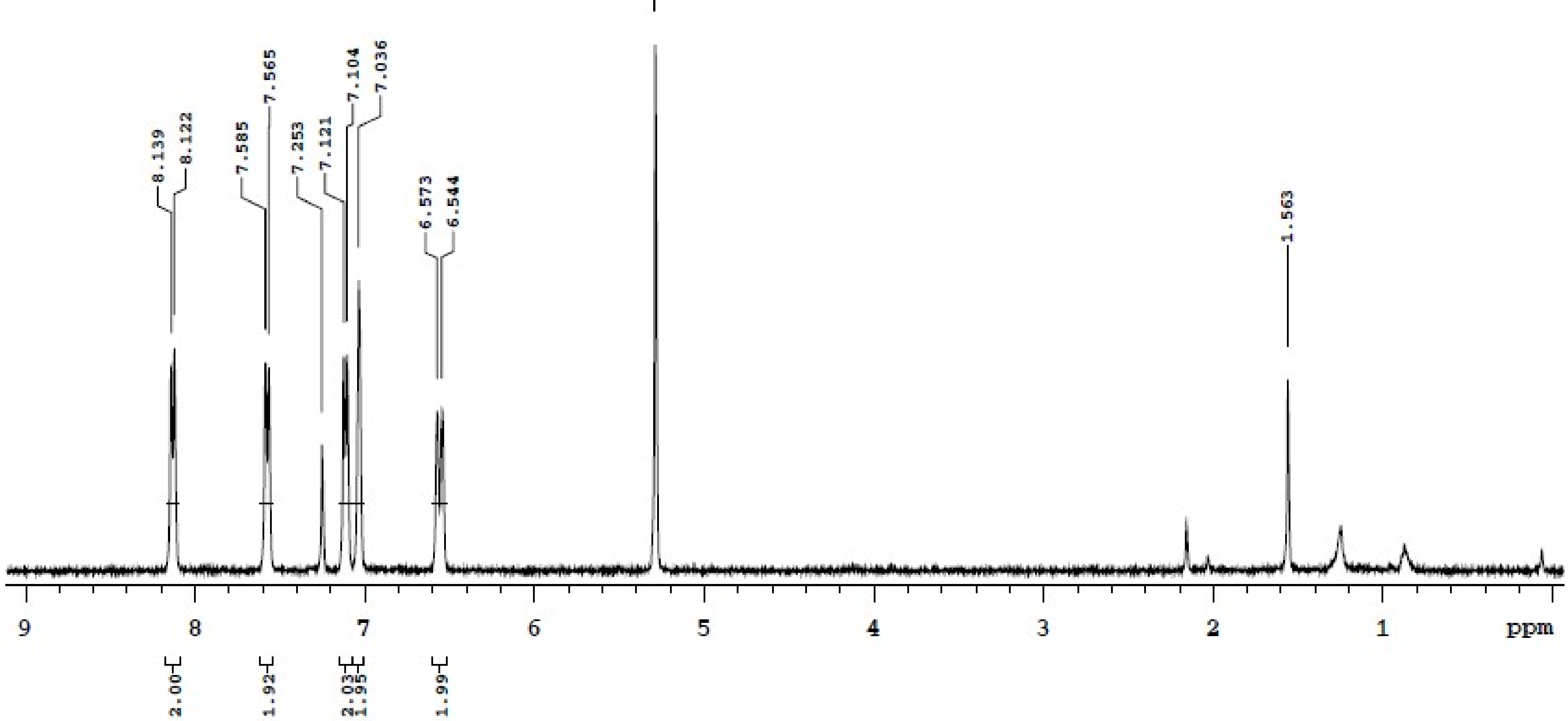

^{13}C NMR: 2-(4-azidophenyl)-4,5-di(furan-2-yl)oxazole

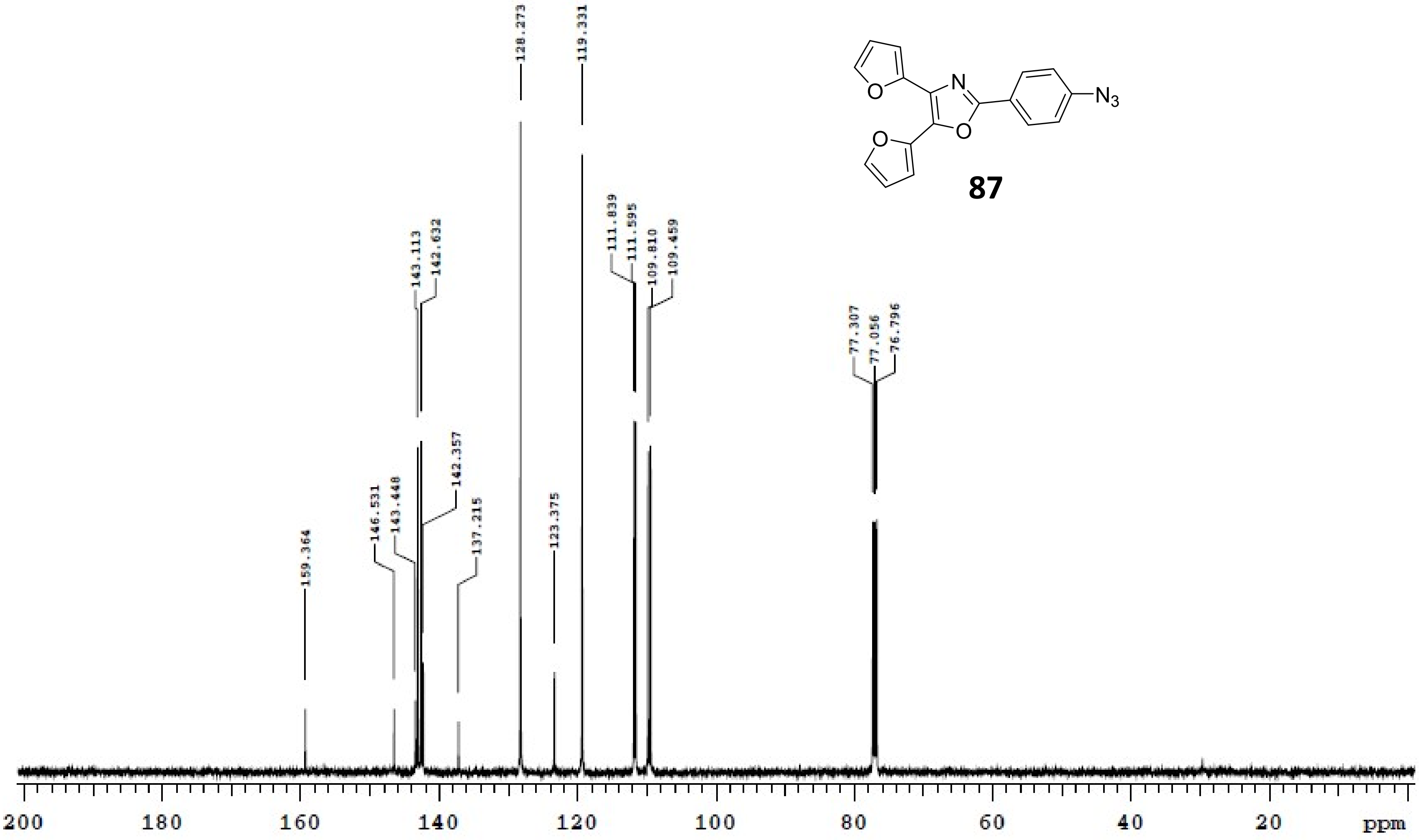

87

^{1}H NMR : N2, N2, N4, N4-tetraethyl-6-((trimethylsilyl)ethynyl)-1,3,5-triazine-2,4-diamine

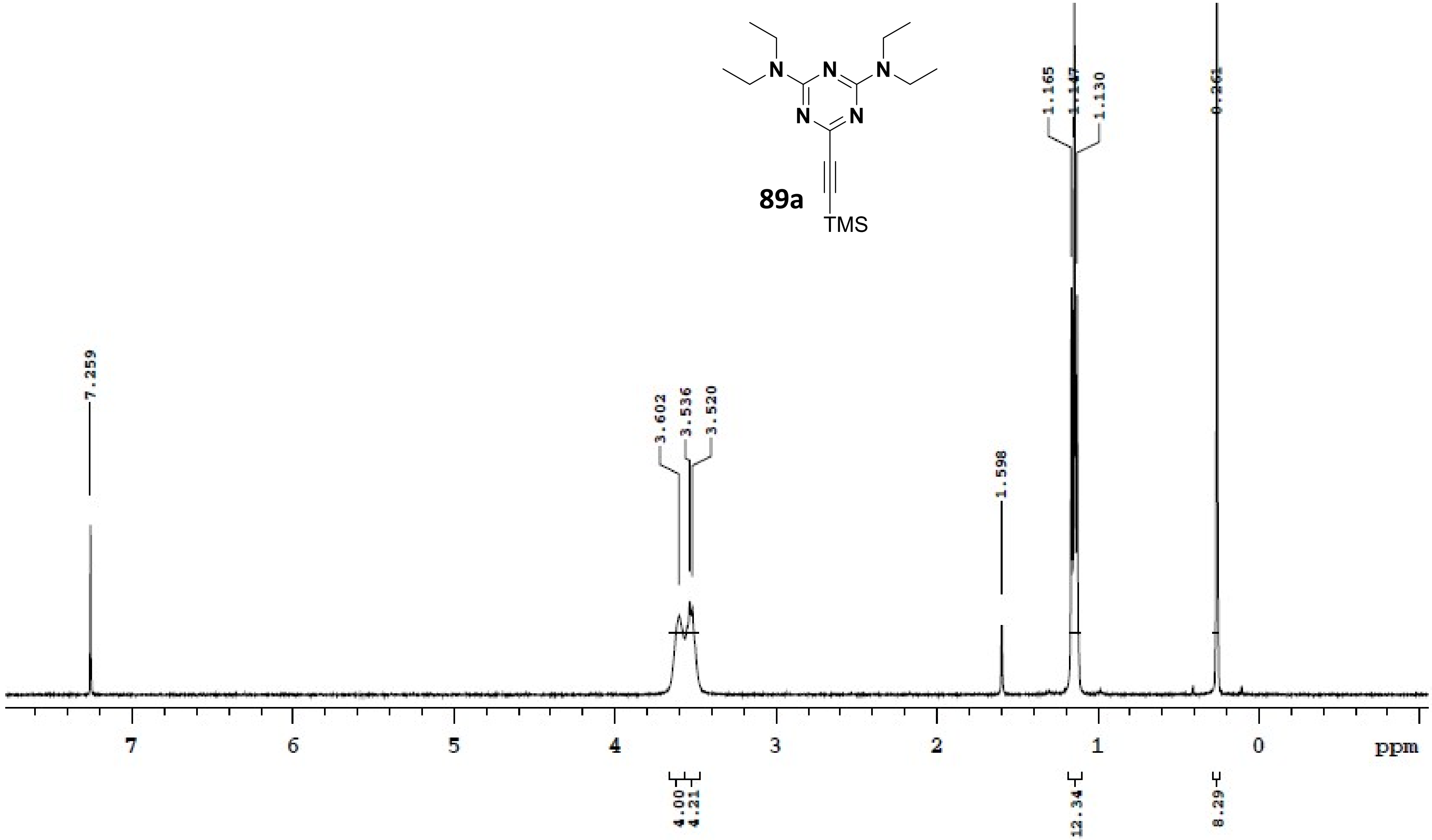

^{13}C NMR: N2, N2, N4, N4-tetraethyl-6-((trimethylsilyl)ethynyl)-1,3,5-triazine-2,4-diamine

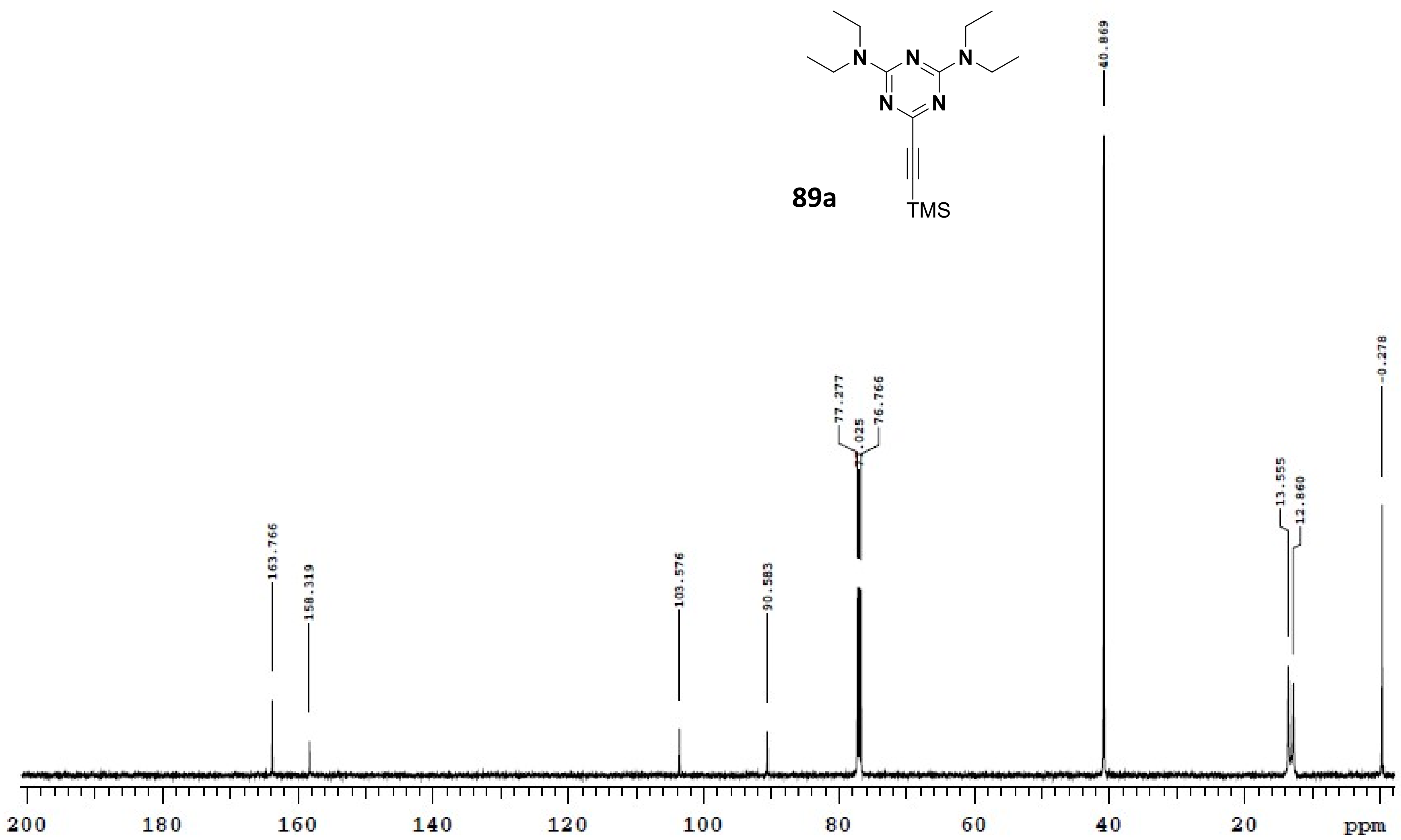

^{1}H NMR: N^2,N^4-bis(2-fluorophenyl)-6-((trimethylsilyl)ethynyl)-1,3,5-triazine-2,4-diamine

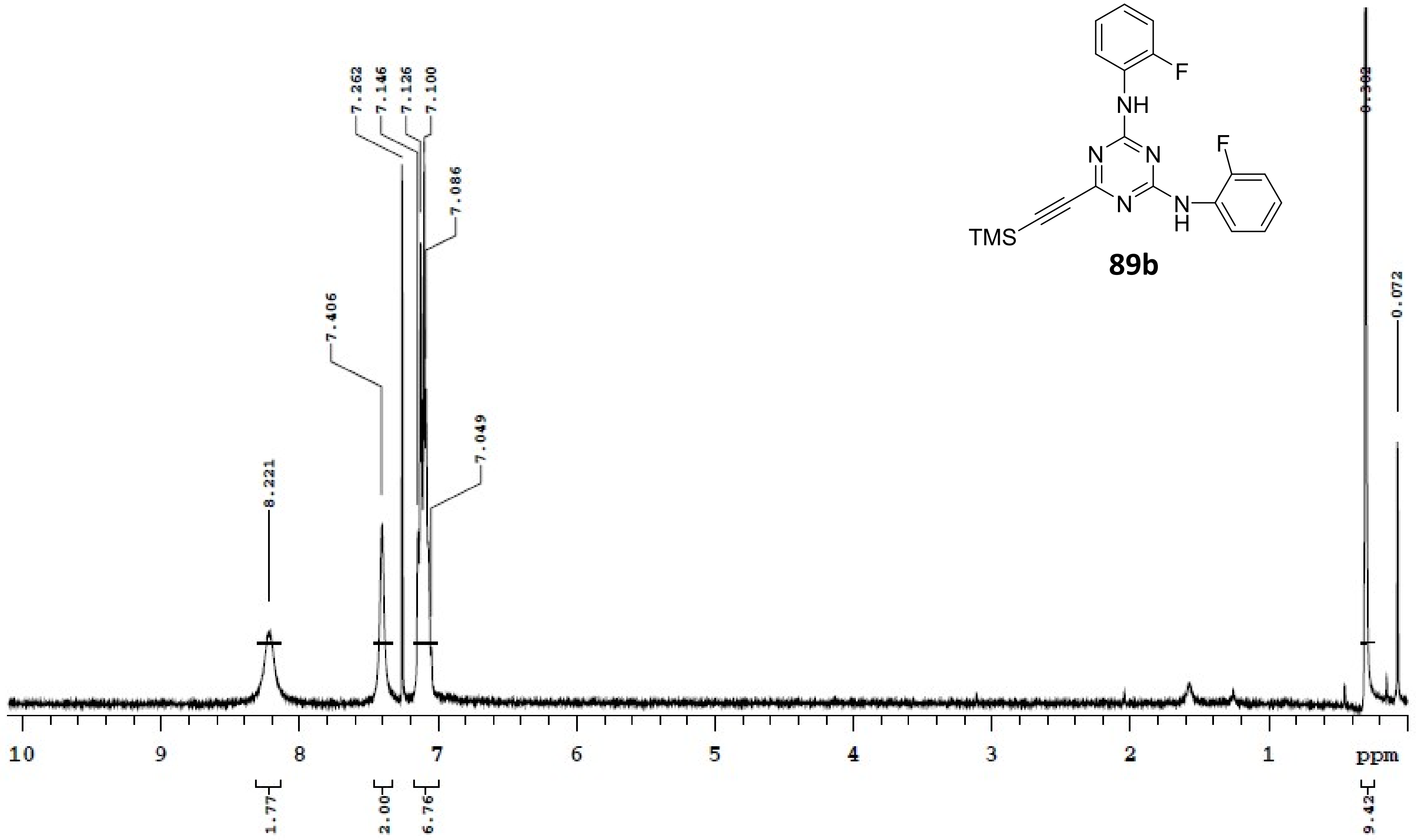

^{13}C NMR: N^{2},N^{4}-bis(2-fluorophenyl)-6-((trimethylsilyl)ethynyl)-1,3,5-triazine-2,4-diamine

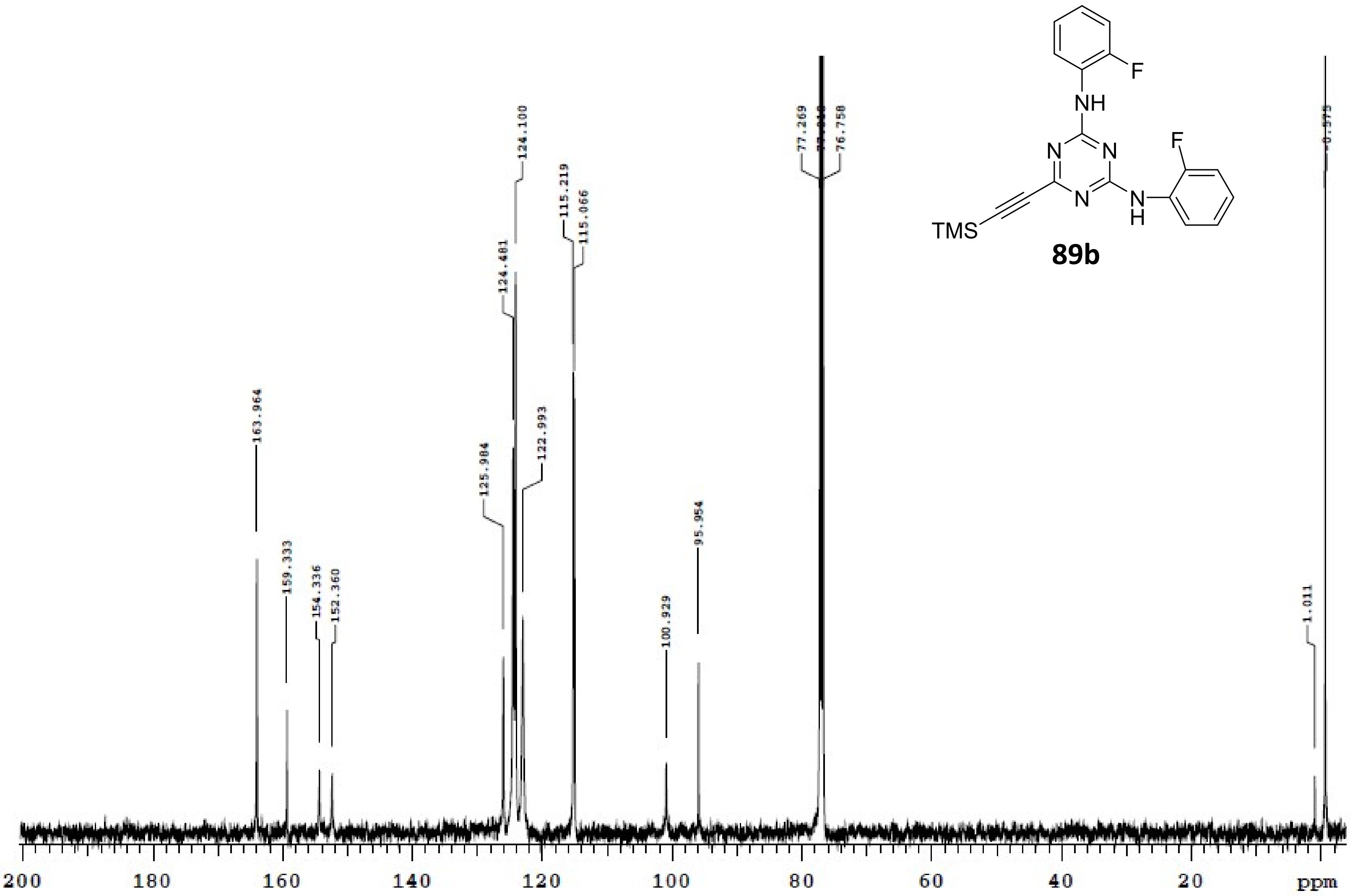

^{1}H NMR: N2,N4-bis(4-fluorophenyl)-6-((trimethylsilyl)ethynyl)-1,3,5-triazine-2,4-diamine

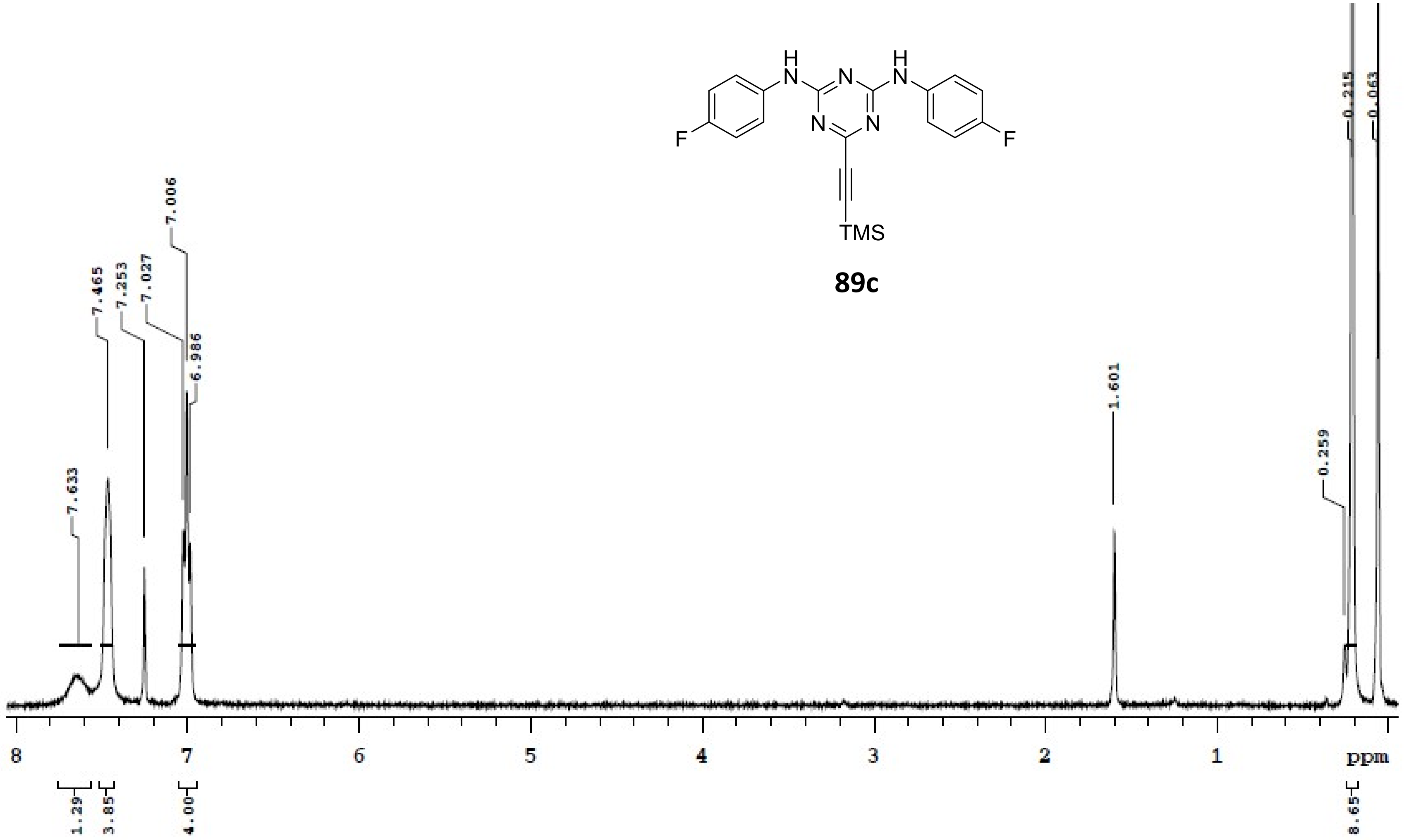

^{13}C NMR: N2,N4-bis(4-fluorophenyl)-6-((trimethylsilyl)ethynyl)-1,3,5-triazine-2,4-diamine

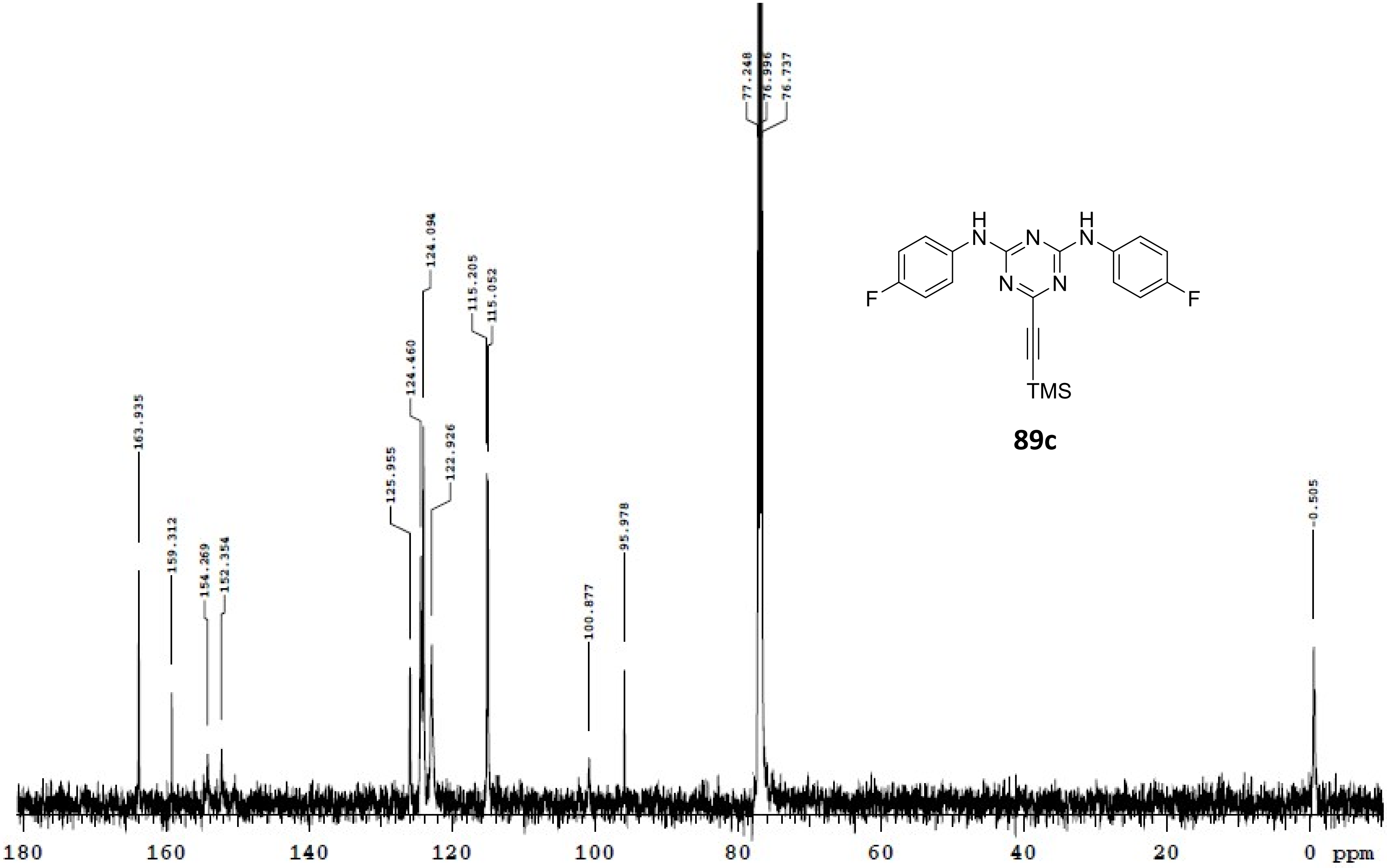

89c

^{1}H NMR : 6-(1-(3-(4,5-diphenyloxazol-2-yl)phenyl)-1H-1,2,3-triazol-4-yl)-N2,N2,N4,N4-tetraethyl-1,3,5-triazine-2,4-diamine

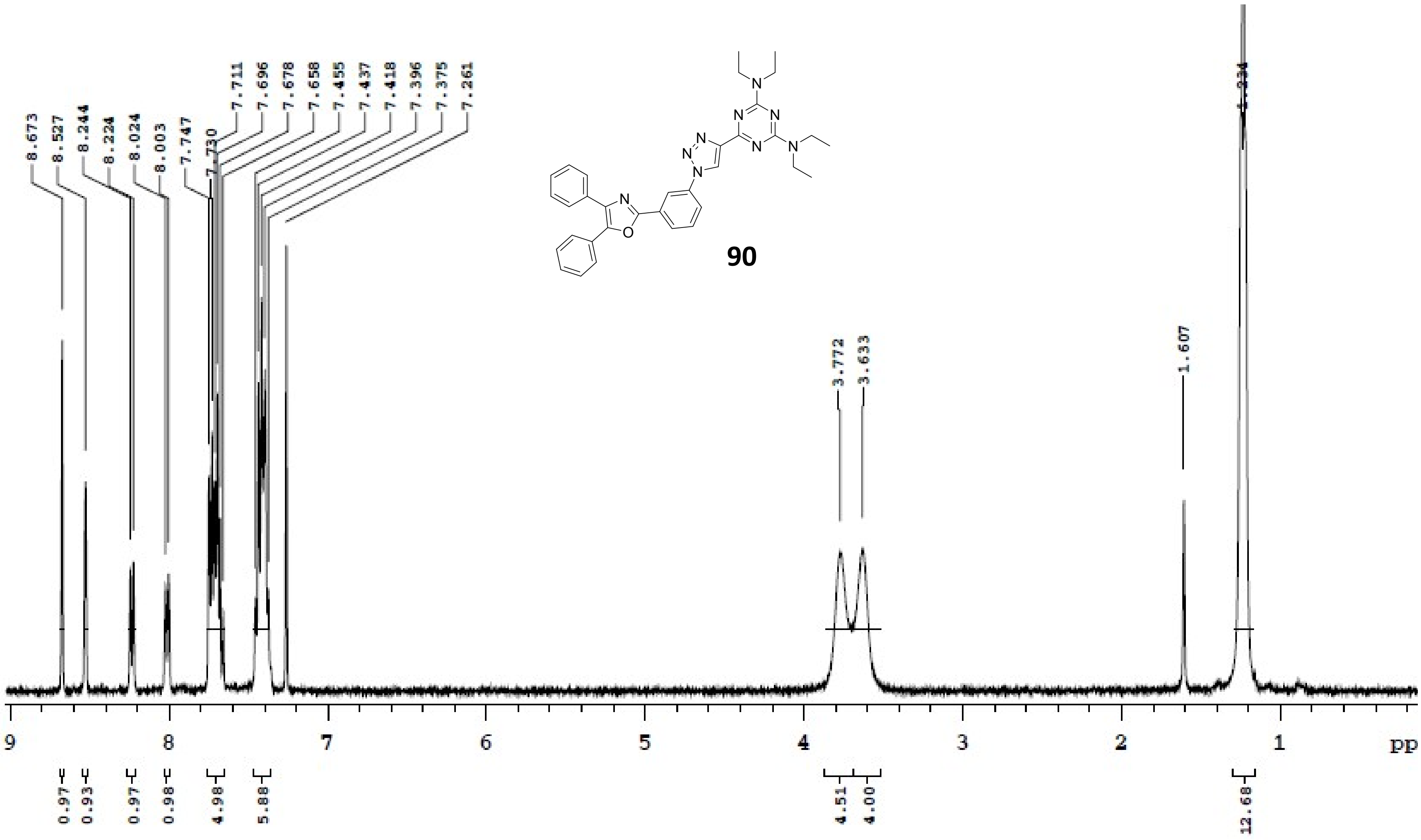

^{13}C NMR: 6-(1-(3-(4,5-diphenyloxazol-2-yl)phenyl)-1H-1,2,3-triazol-4-yl)-N2,N2,N4,N4-tetraethyl-1,3,5-triazine-2,4-diamine

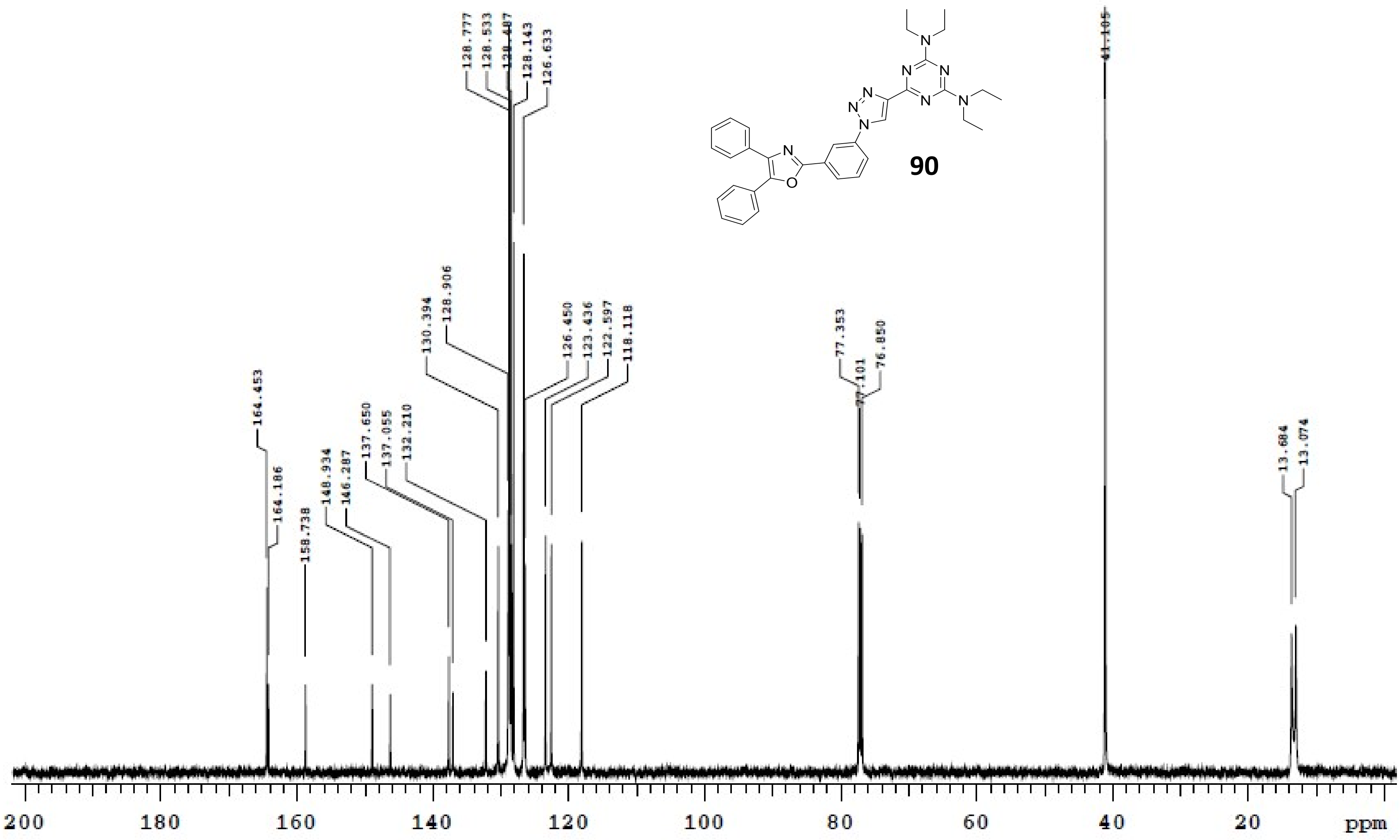

90

^{1}H NMR : 6-(1-(4-(4,5-diphenyloxazol-2-yl)phenyl)-1H-1,2,3-triazol-4-yl)-N2,N2,N4,N4-tetraethyl-1,3,5-triazine-2,4-diamine

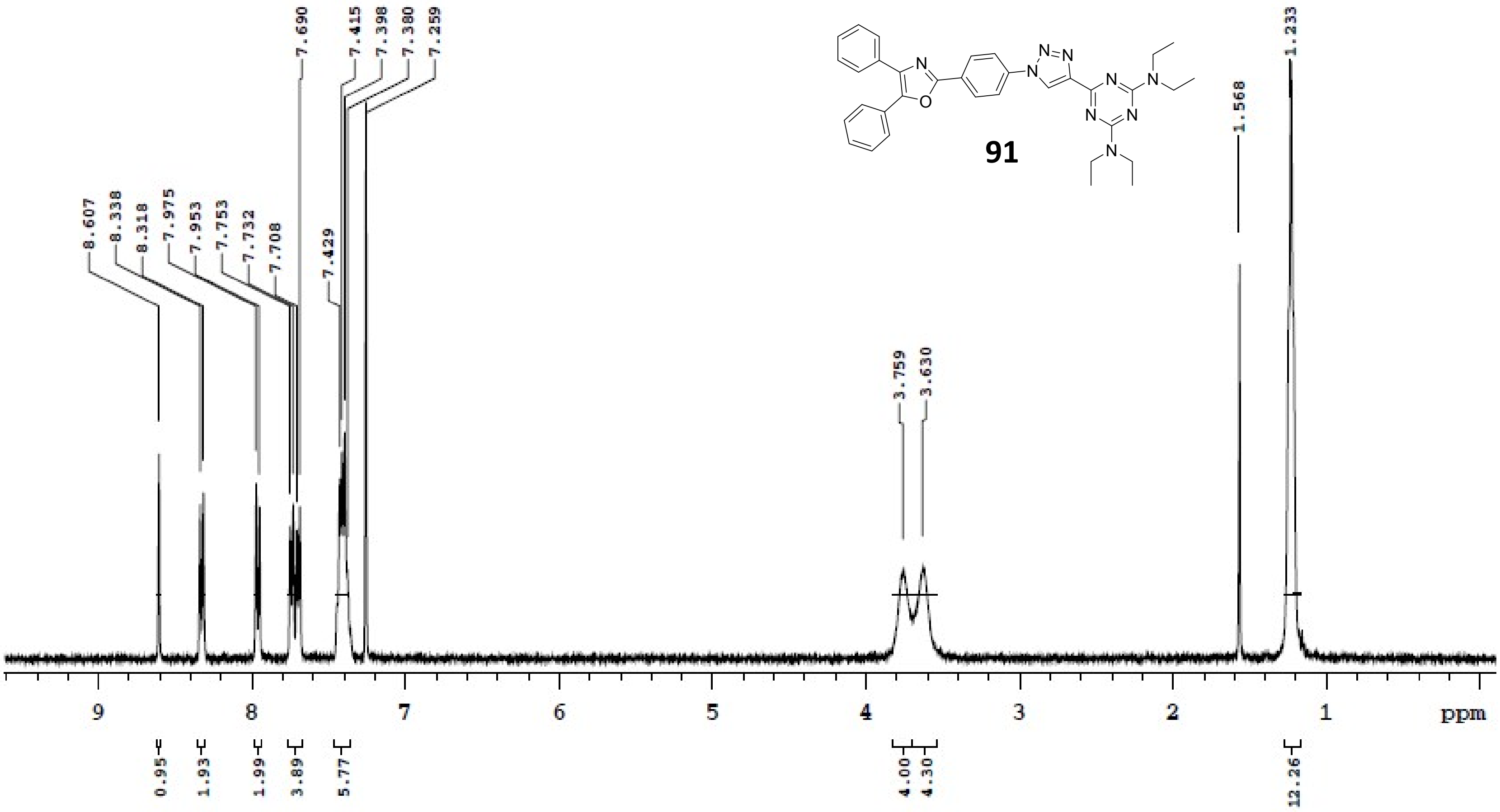

^{13}C NMR: 6-(1-(4-(4,5-diphenyloxazol-2-yl)phenyl)-1H-1,2,3-triazol-4-yl)-N2,N2,N4,N4-tetraethyl-1,3,5-triazine-2,4-diamine

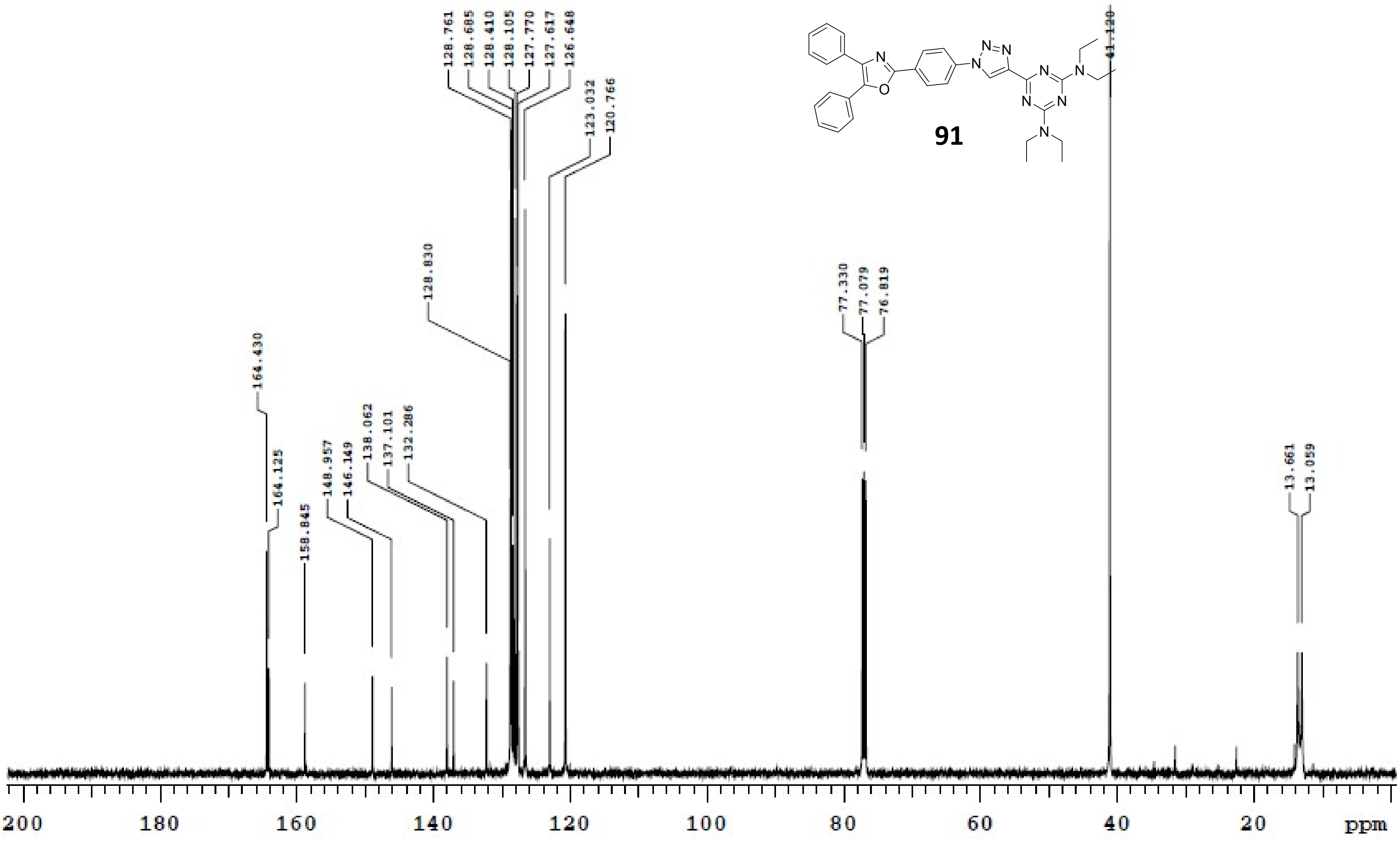

^{1}H NMR : 6-(1-(3-(4,5-bis(4-fluorophenyl)oxazol-2-yl)phenyl)-1H-1,2,3-triazol-4-yl)-N2,N2,N4,N4-tetraethyl-1,3,5-triazine-2,4-diamine

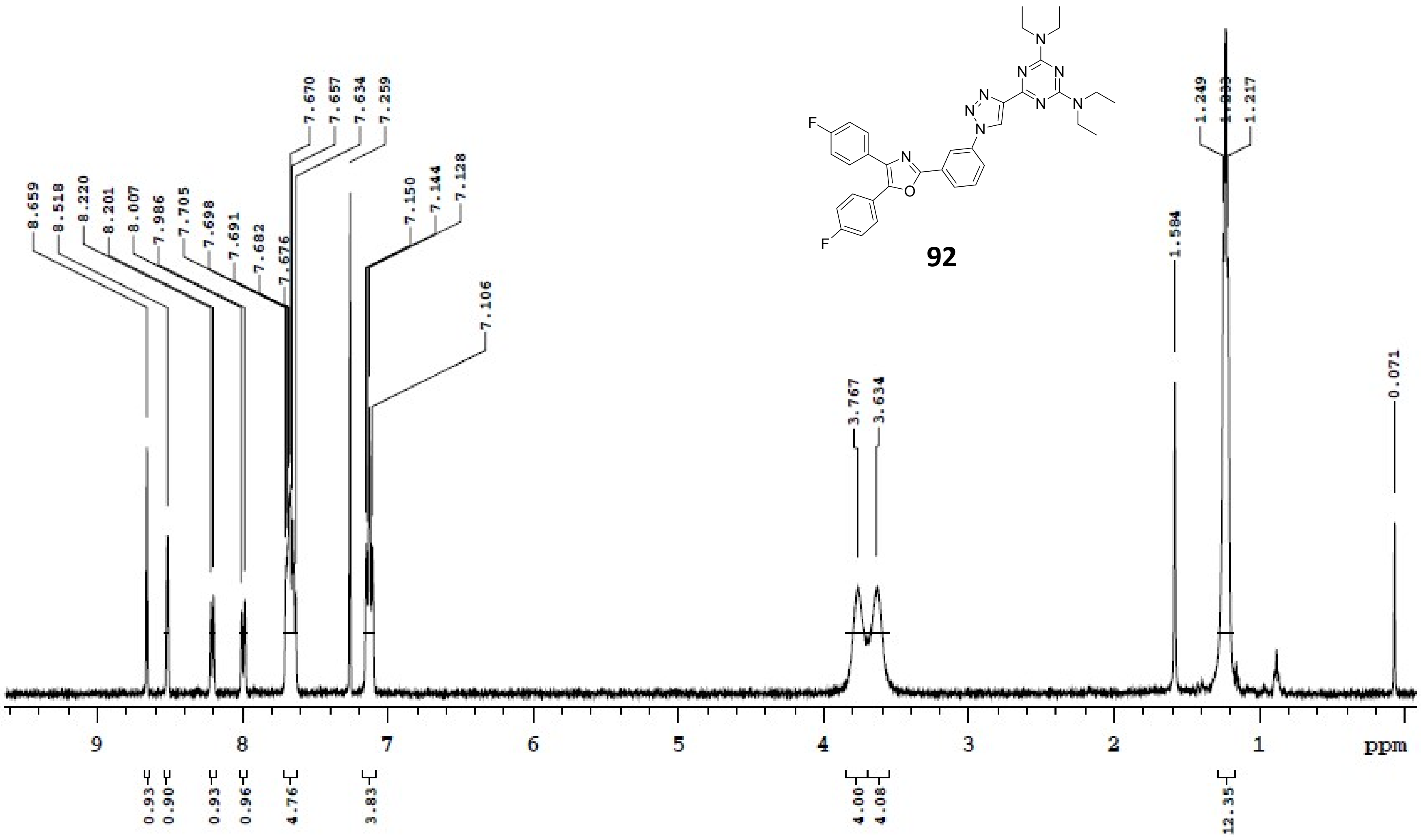

^{13}C NMR: 6-(1-(3-(4,5-bis(4-fluorophenyl)oxazol-2-yl)phenyl)-1H-1,2,3-triazol-4-yl)-N2,N2,N4,N4-tetraethyl-1,3,5-triazine-2,4-diamine

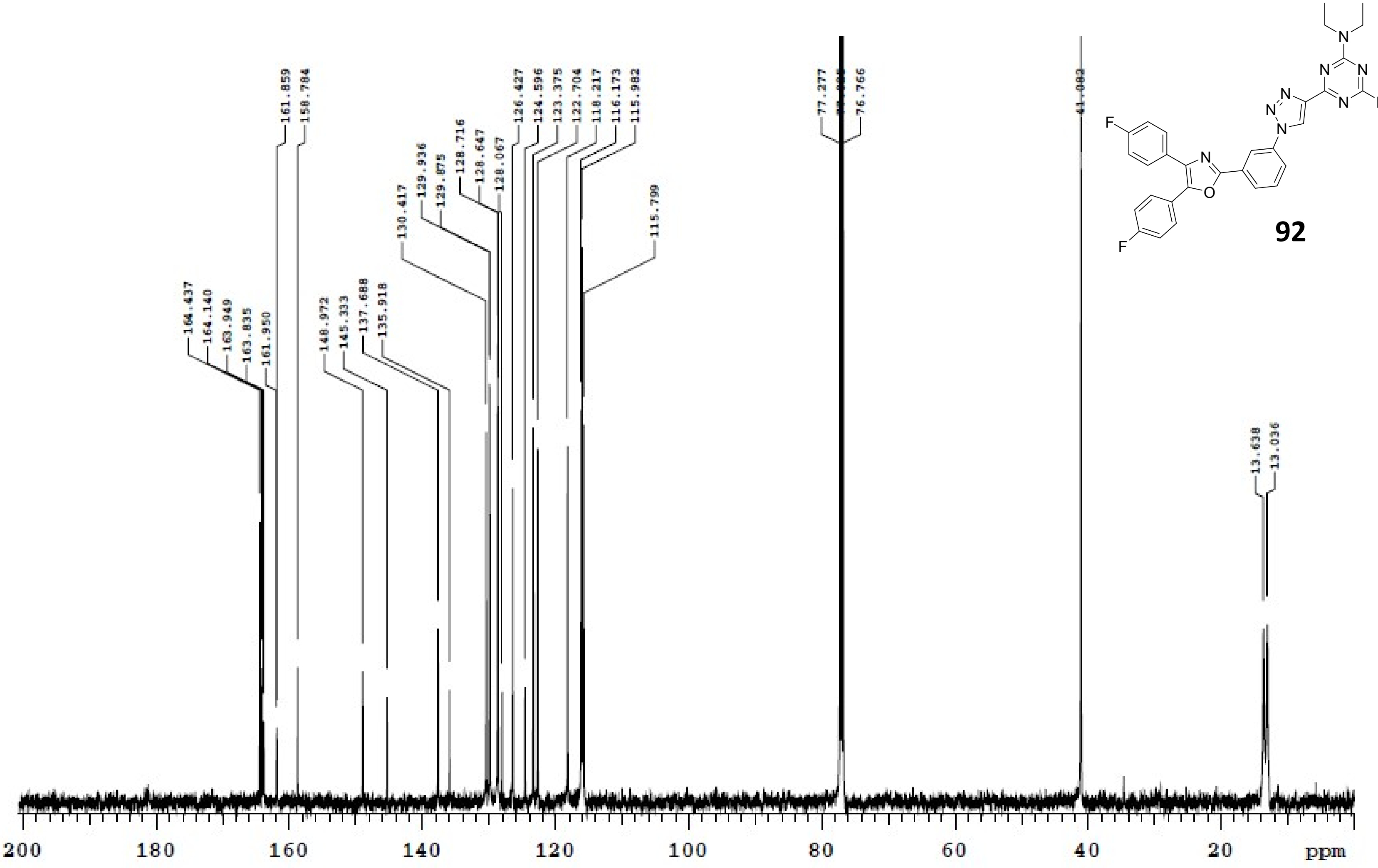

^{1}H NMR : 6-(1-(4-(4,5-bis(4-fluorophenyl)oxazol-2-yl)phenyl)-1H-1,2,3-triazol-4-yl)-N2,N2,N4,N4-tetraethyl-1,3,5-triazine-2,4-diamine

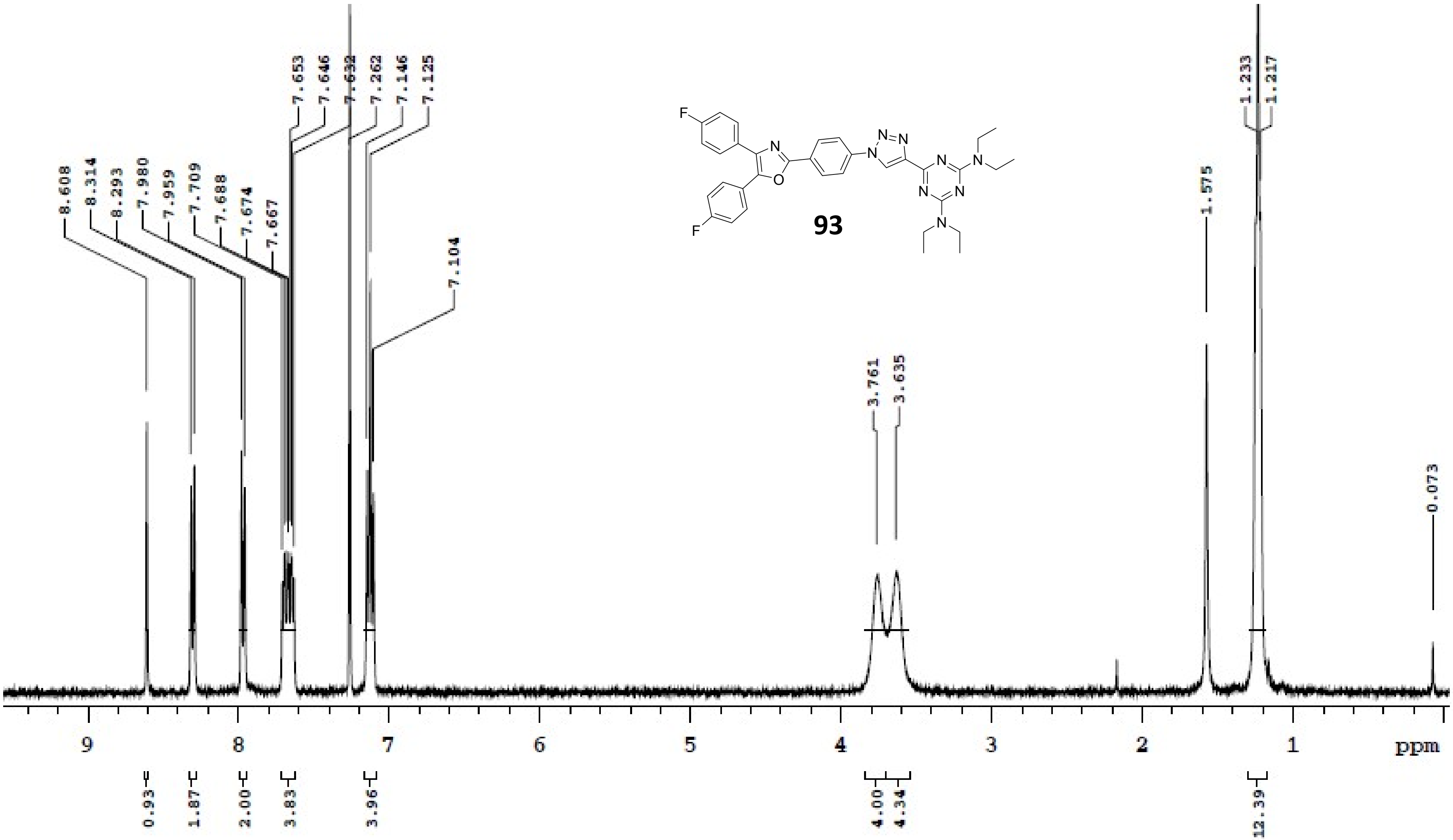

^{13}C NMR: 6-(1-(4-(4,5-bis(4-fluorophenyl)oxazol-2-yl)phenyl)-1H-1,2,3-triazol-4-yl)-N2,N2,N4,N4-tetraethyl-1,3,5-triazine-2,4-diamine

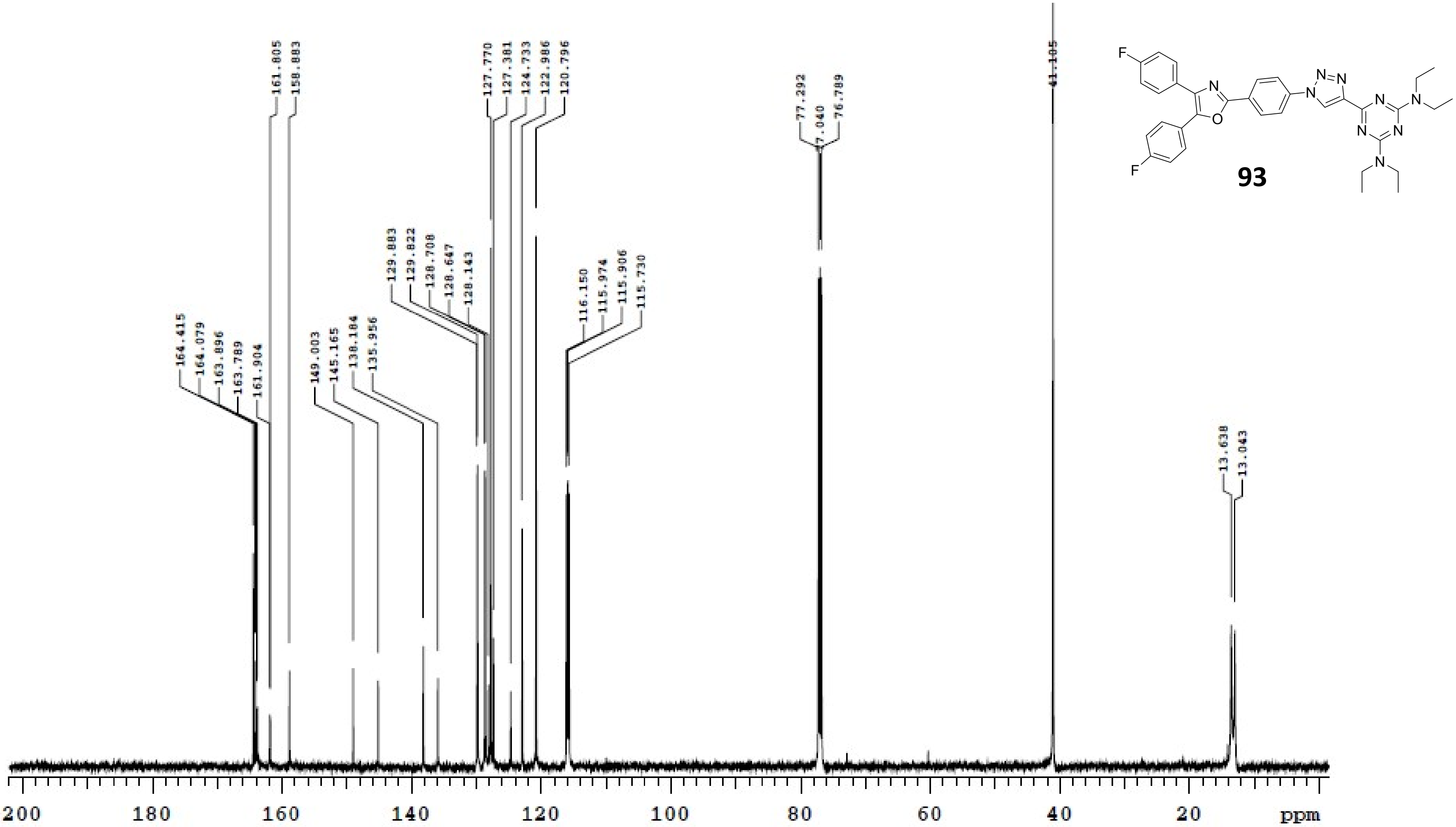

93

^{1}H NMR : 6-(1-(3-(4,5-bis(4-chlorophenyl)oxazol-2-yl)phenyl)-1H-1,2,3-triazol-4-yl)-N2,N2,N4,N4-tetraethyl-1,3,5-triazine-2,4-diamine

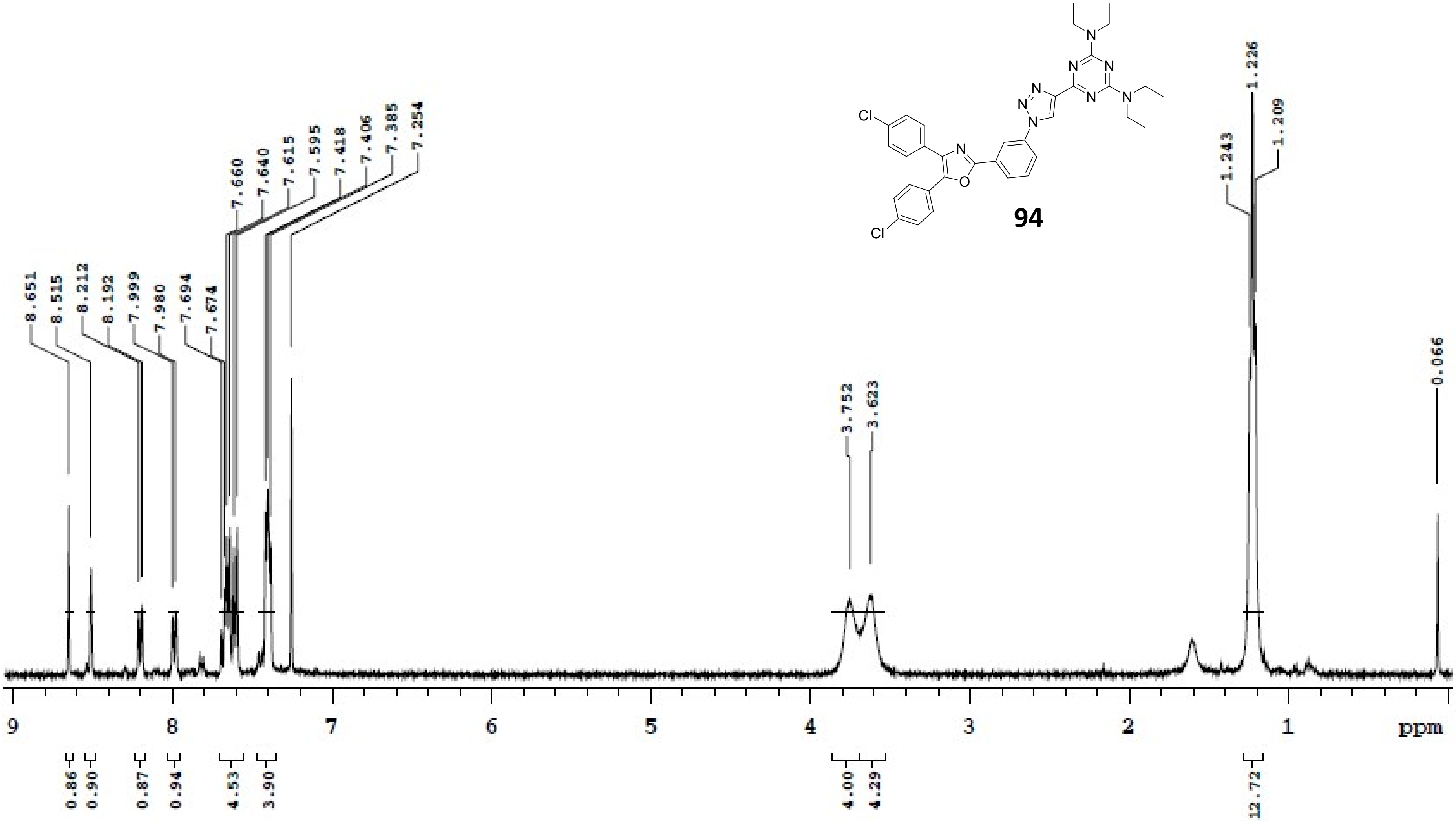

^{13}C NMR: 6-(1-(3-(4,5-bis(4-chlorophenyl)oxazol-2-yl)phenyl)-1H-1,2,3-triazol-4-yl)-N2,N2,N4,N4-tetraethyl-1,3,5-triazine-2,4-diamine

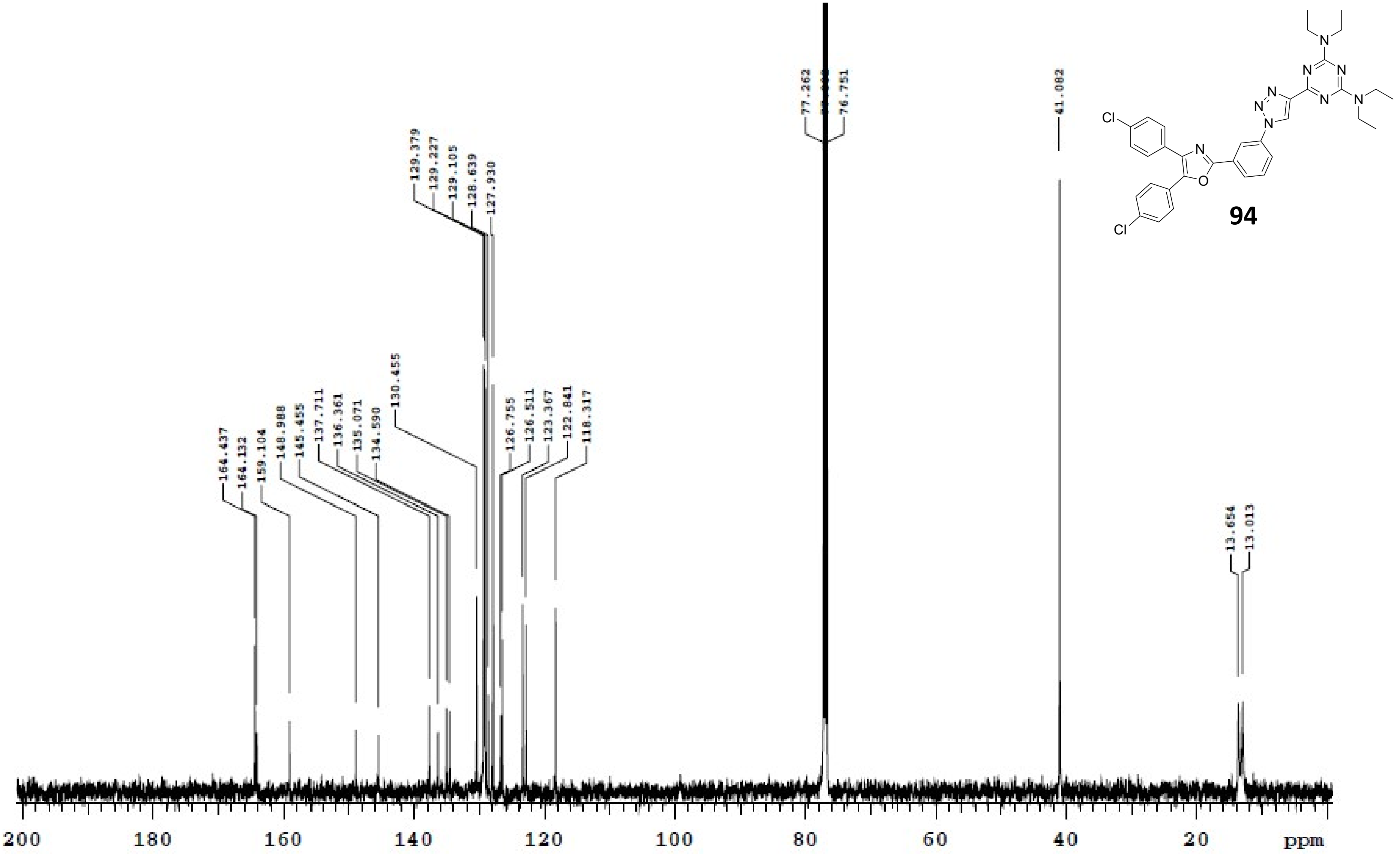

94

^{1}H NMR : 6-(1-(4-(4,5-bis(4-chlorophenyl)oxazol-2-yl)phenyl)-1H-1,2,3-triazol-4-yl)-N2,N2,N4,N4-tetraethyl-1,3,5-triazine-2,4-diamine

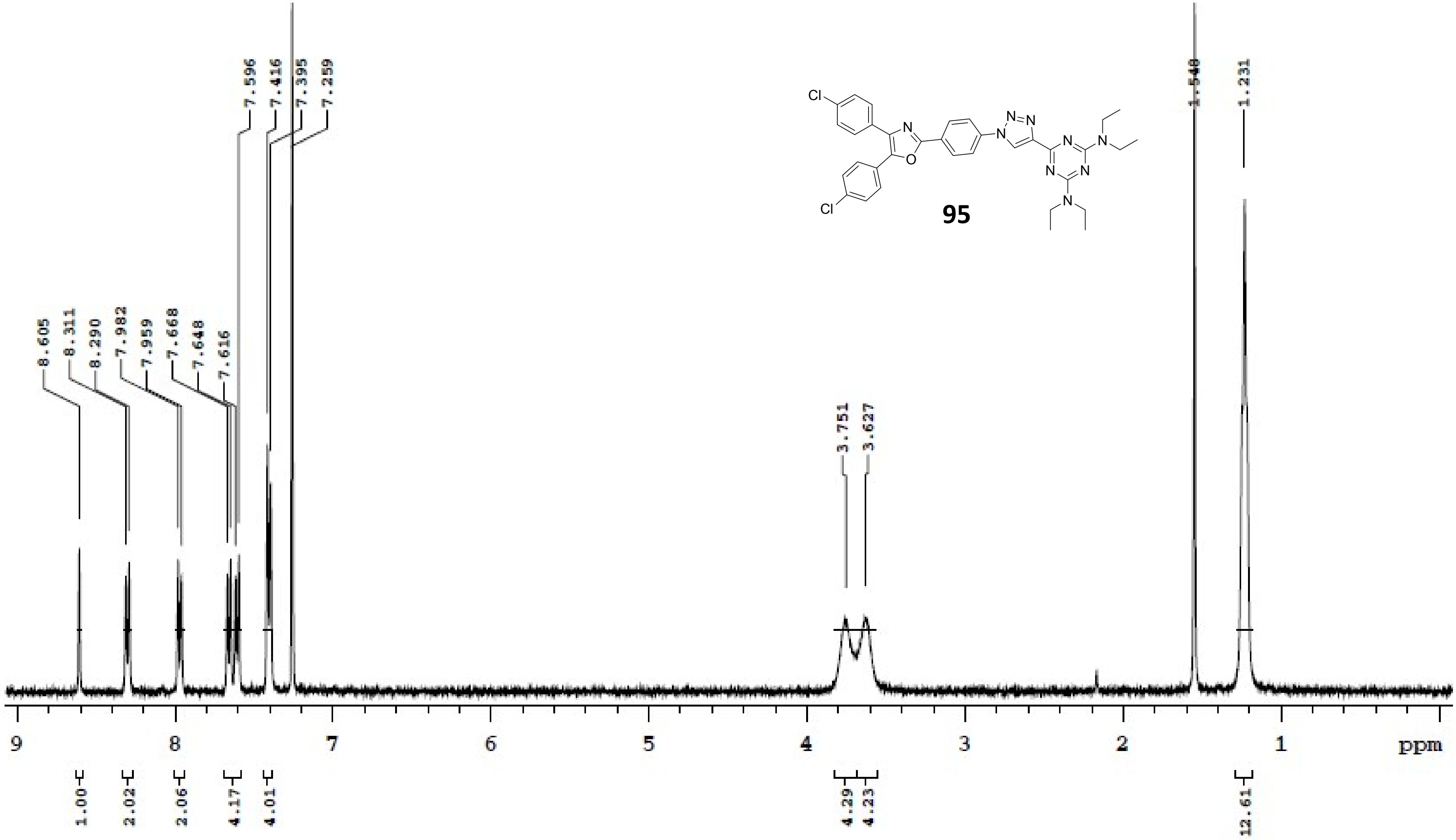

^{13}C NMR: 6-(1-(4-(4,5-bis(4-chlorophenyl)oxazol-2-yl)phenyl)-1H-1,2,3-triazol-4-yl)-N2,N2,N4,N4-tetraethyl-1,3,5-triazine-2,4-diamine

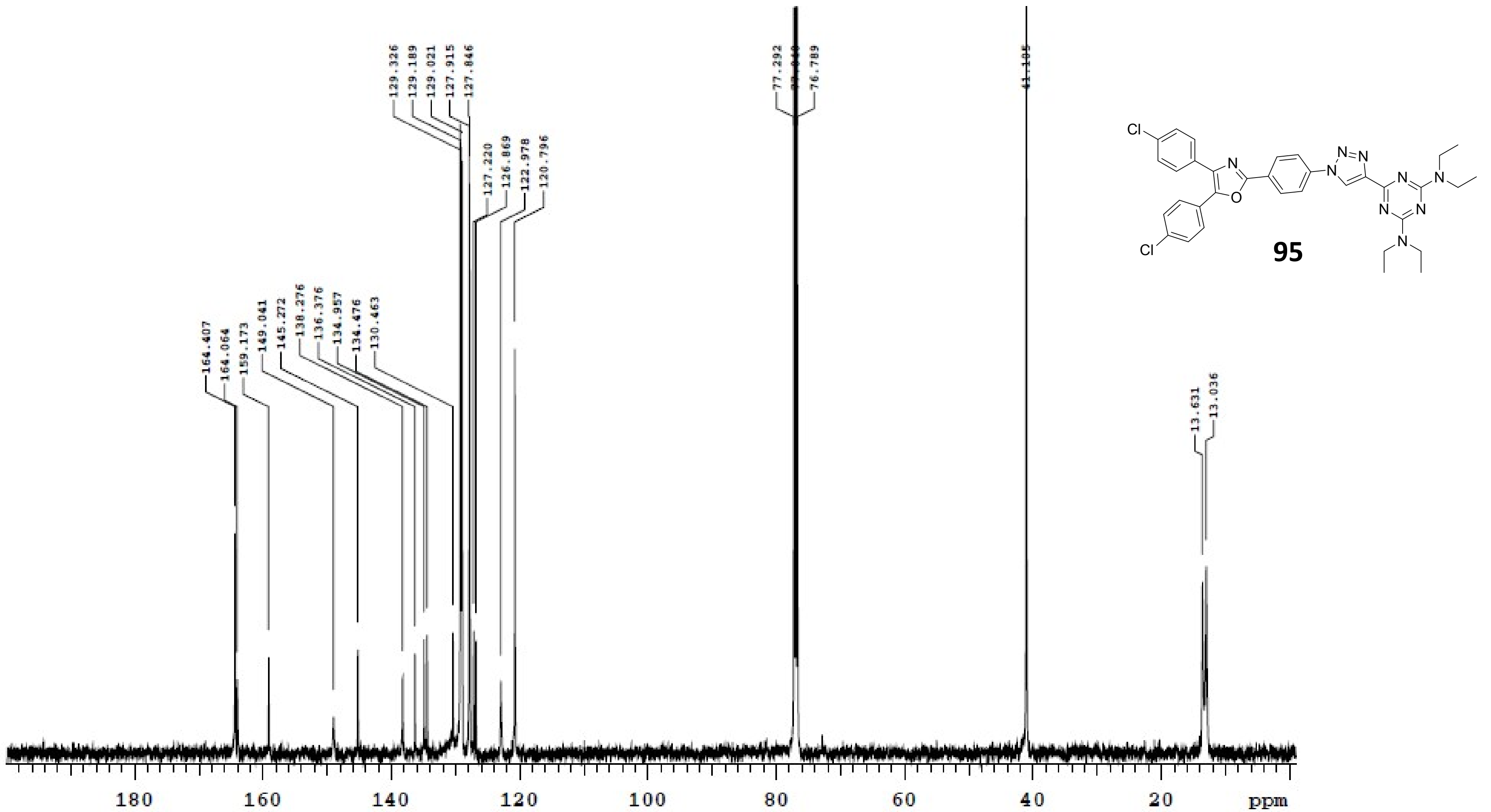

^{1}H NMR : 6-(1-(3-(4,5-bis(4-methoxyphenyl)oxazol-2-yl)phenyl)-1H-1,2,3-triazol-4-yl)-N2,N2,N4,N4-tetraethyl-1,3,5-triazine-2,4-diamine

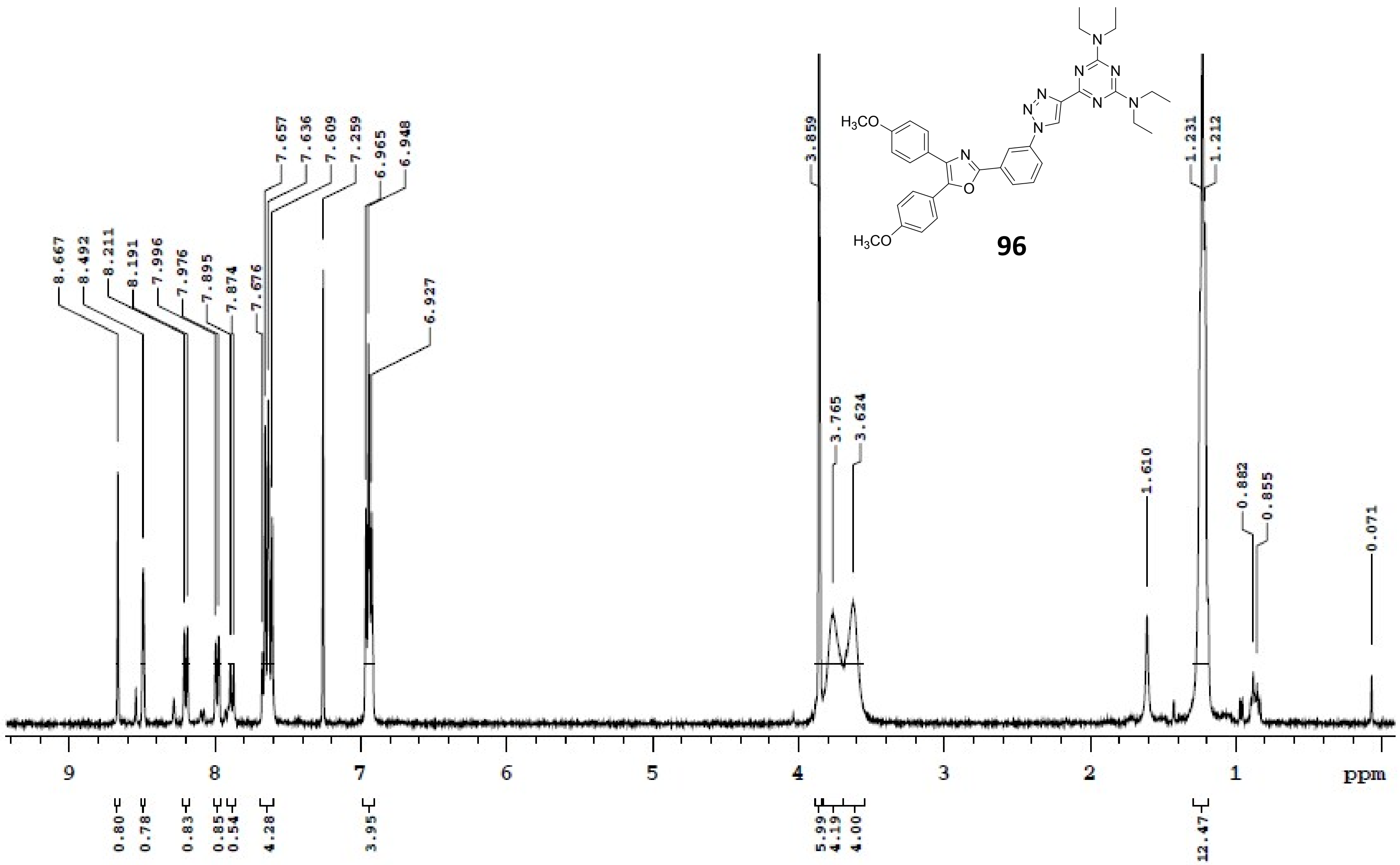

^{13}C NMR: 6-(1-(3-(4,5-bis(4-methoxyphenyl)oxazol-2-yl)phenyl)-1H-1,2,3-triazol-4-yl)-N2,N2,N4,N4-tetraethyl-1,3,5-triazine-2,4-diamine

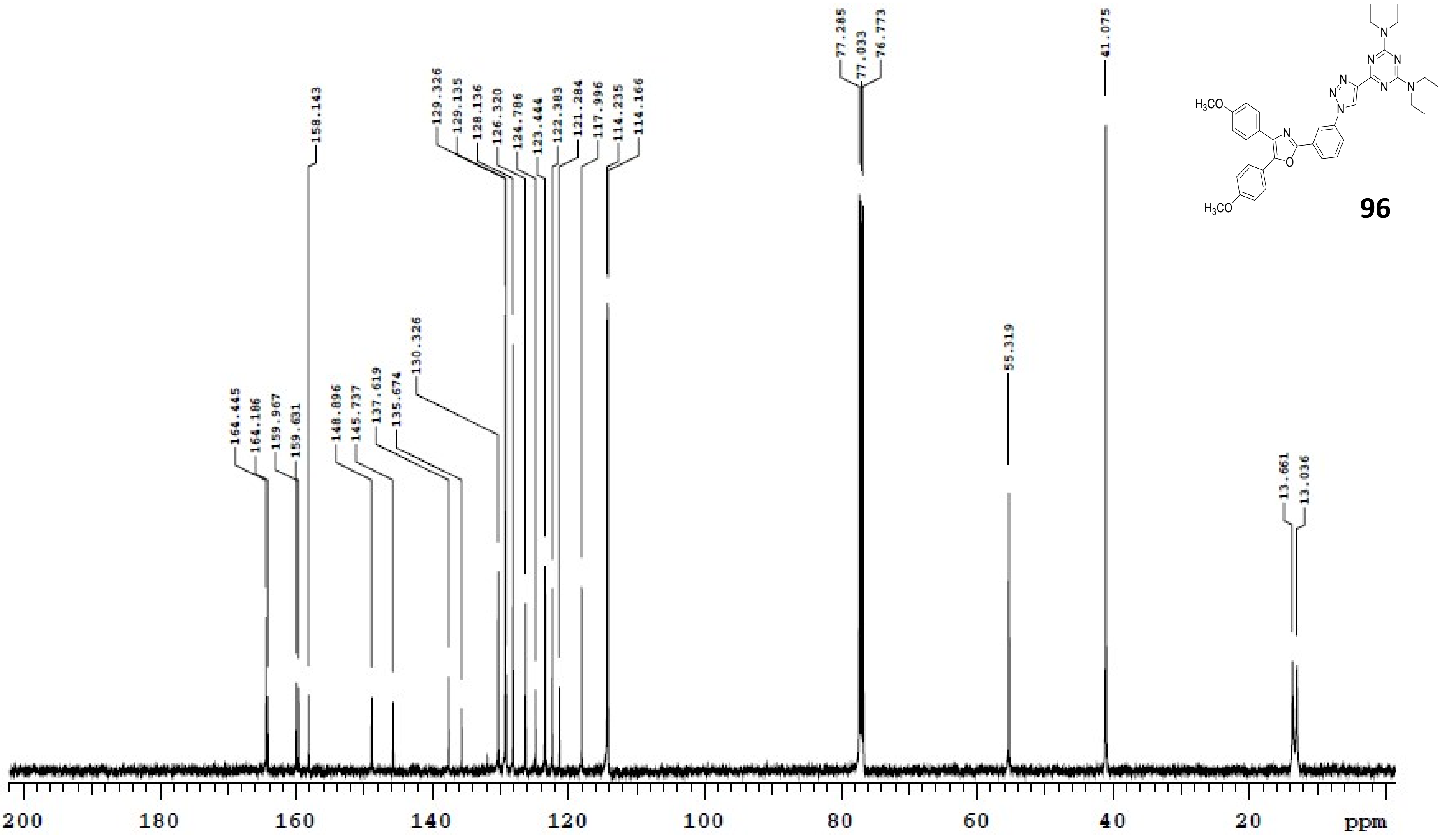

96

^{1}H NMR : 6-(1-(4-(4,5-bis(4-methoxyphenyl)oxazol-2-yl)phenyl)-1H-1,2,3-triazol-4-yl)-N2,N2,N4,N4-tetraethyl-1,3,5-triazine-2,4-diamine

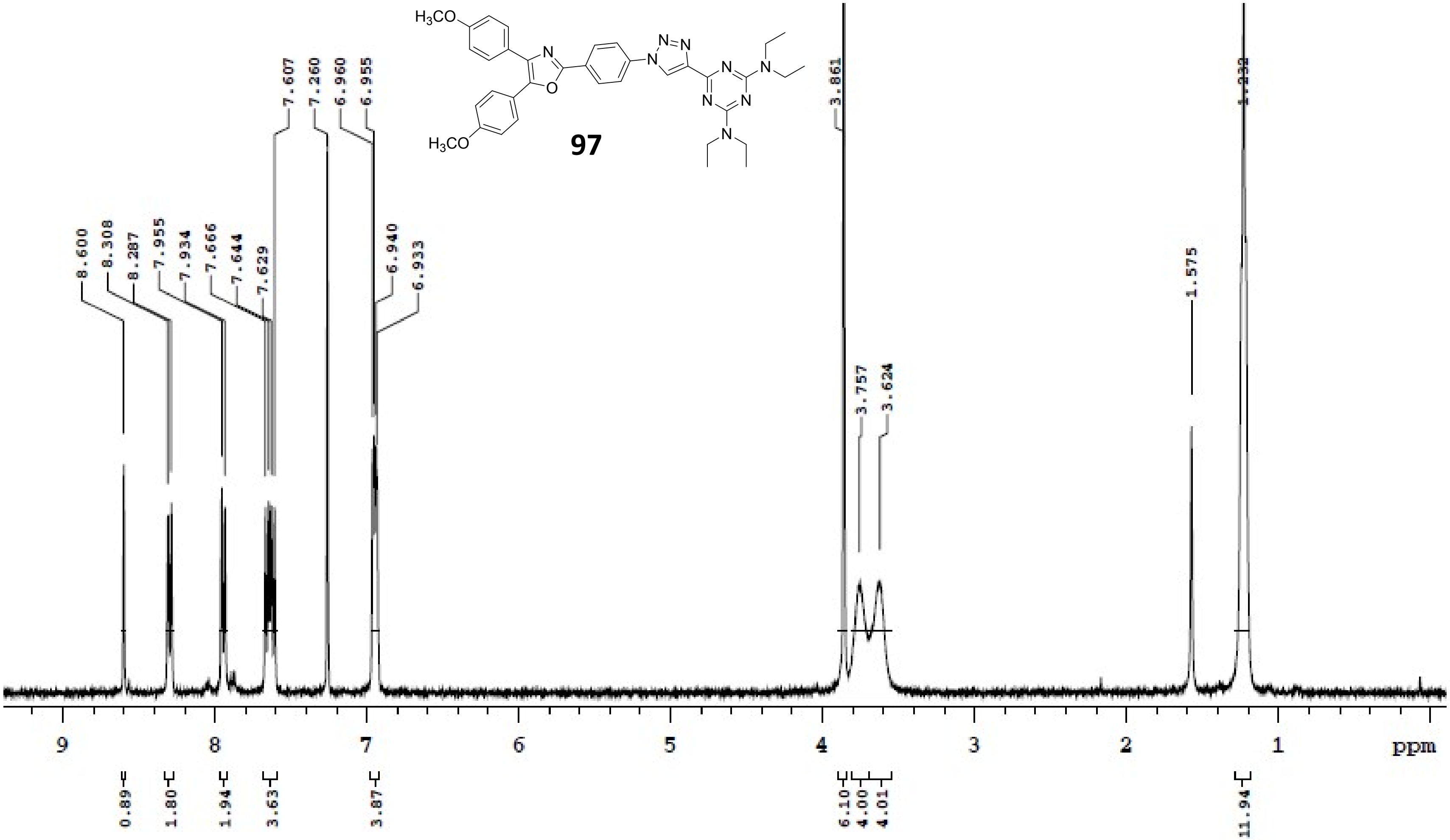

^{13}C NMR: 6-(1-(4-(4,5-bis(4-methoxyphenyl)oxazol-2-yl)phenyl)-1H-1,2,3-triazol-4-yl)-N2,N2,N4,N4-tetraethyl-1,3,5-triazine-2,4-diamine

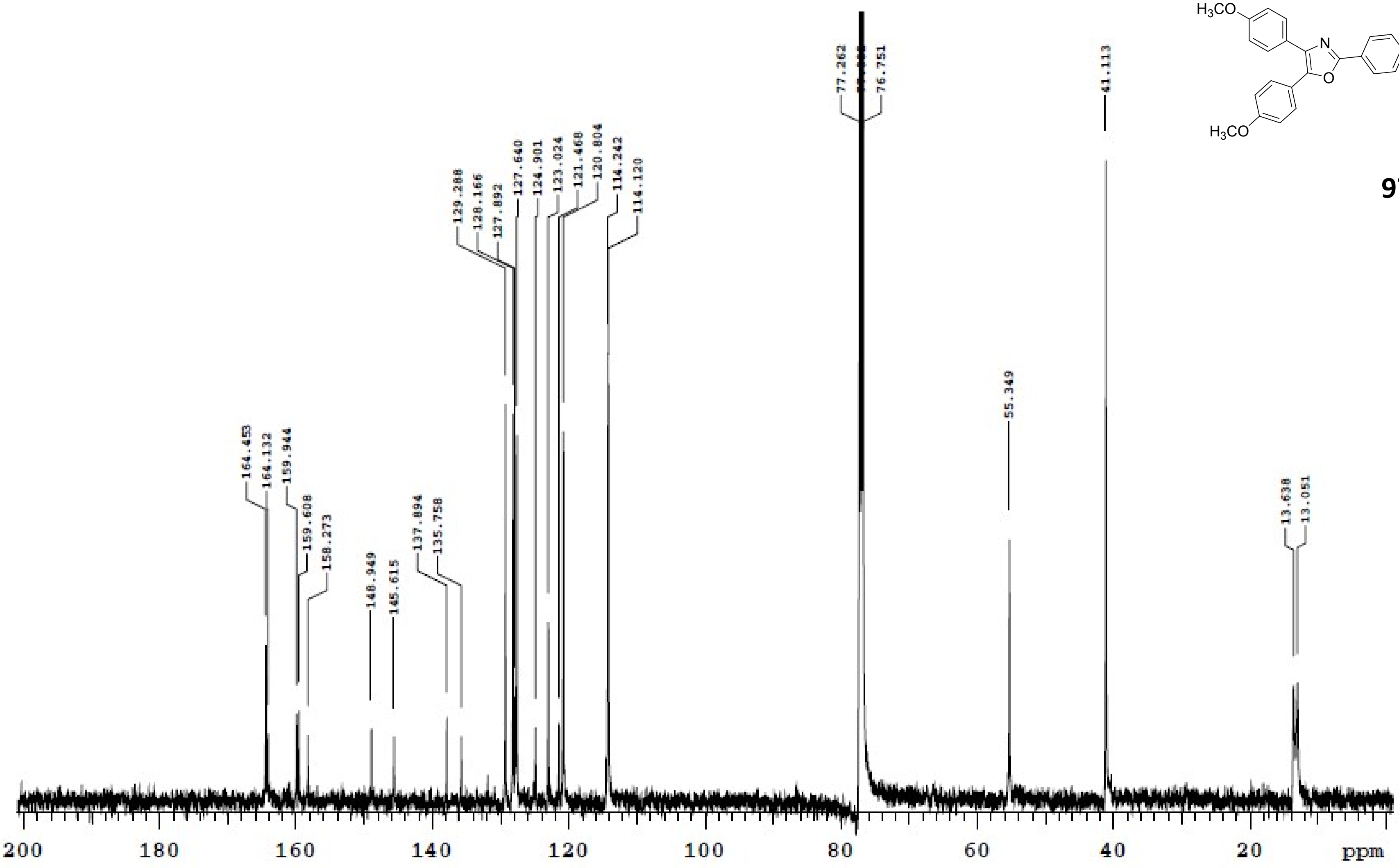

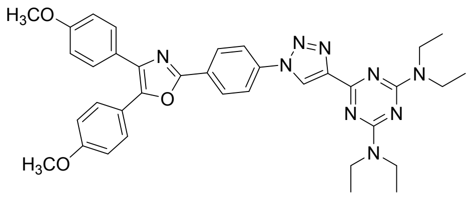

97

^{1}H NMR : 6-(1-(3-(4,5-di(furan-2-yl)oxazol-2-yl)phenyl)-1H-1,2,3-triazol-4-yl)-N2,N2,N4,N4-tetraethyl-1,3,5-triazine-2,4-diamine

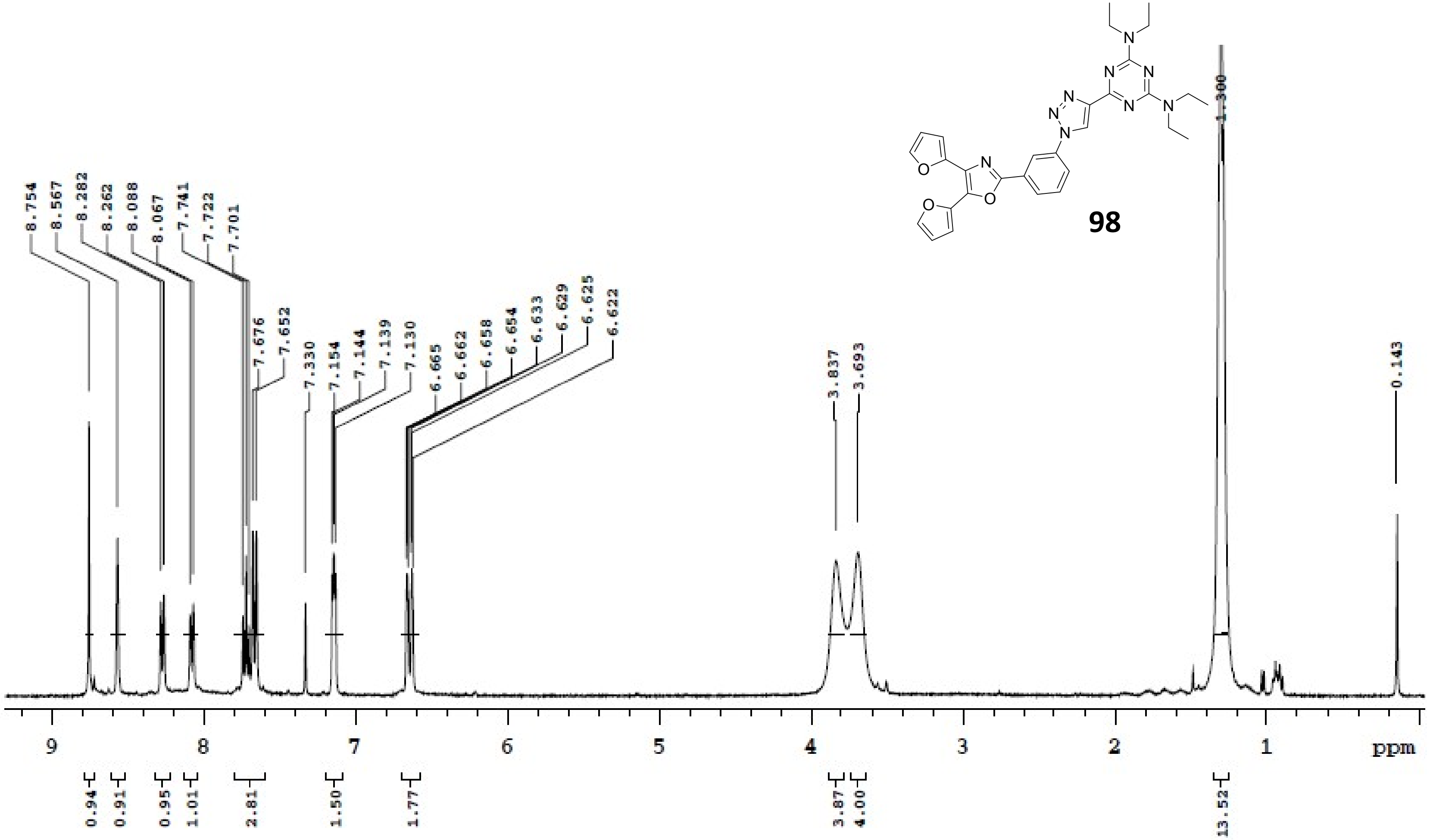

^{13}C NMR: 6-(1-(3-(4,5-di(furan-2-yl)oxazol-2-yl)phenyl)-1H-1,2,3-triazol-4-yl)-N2,N2,N4,N4-tetraethyl-1,3,5-triazine-2,4-diamine

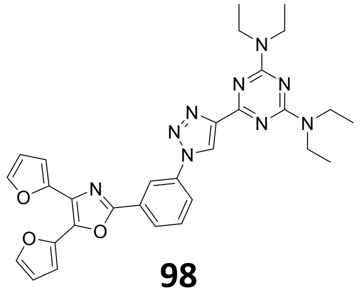

98

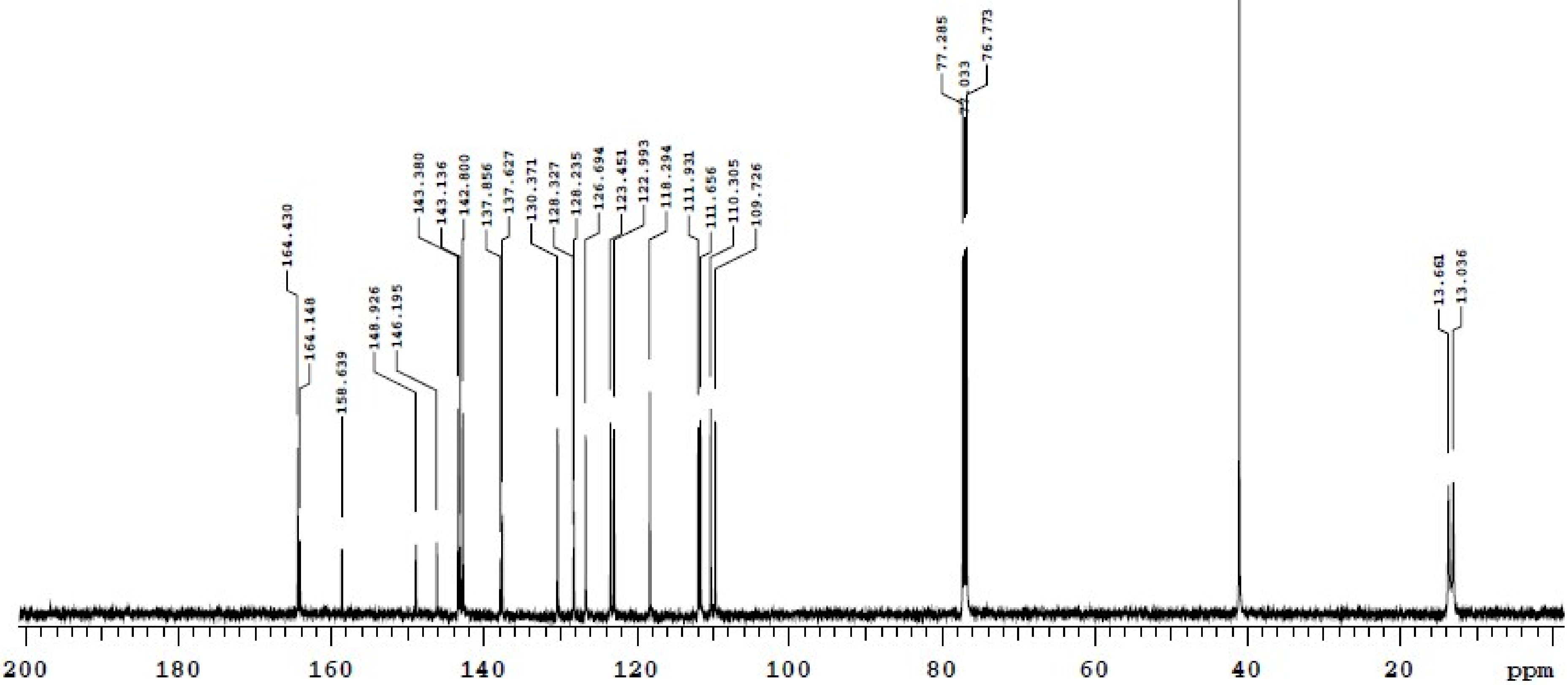

^{1}H NMR : 6-(1-(4-(4,5-di(furan-2-yl)oxazol-2-yl)phenyl)-1H-1,2,3-triazol-4-yl)-N2,N2,N4,N4-tetraethyl-1,3,5-triazine-2,4-diamine

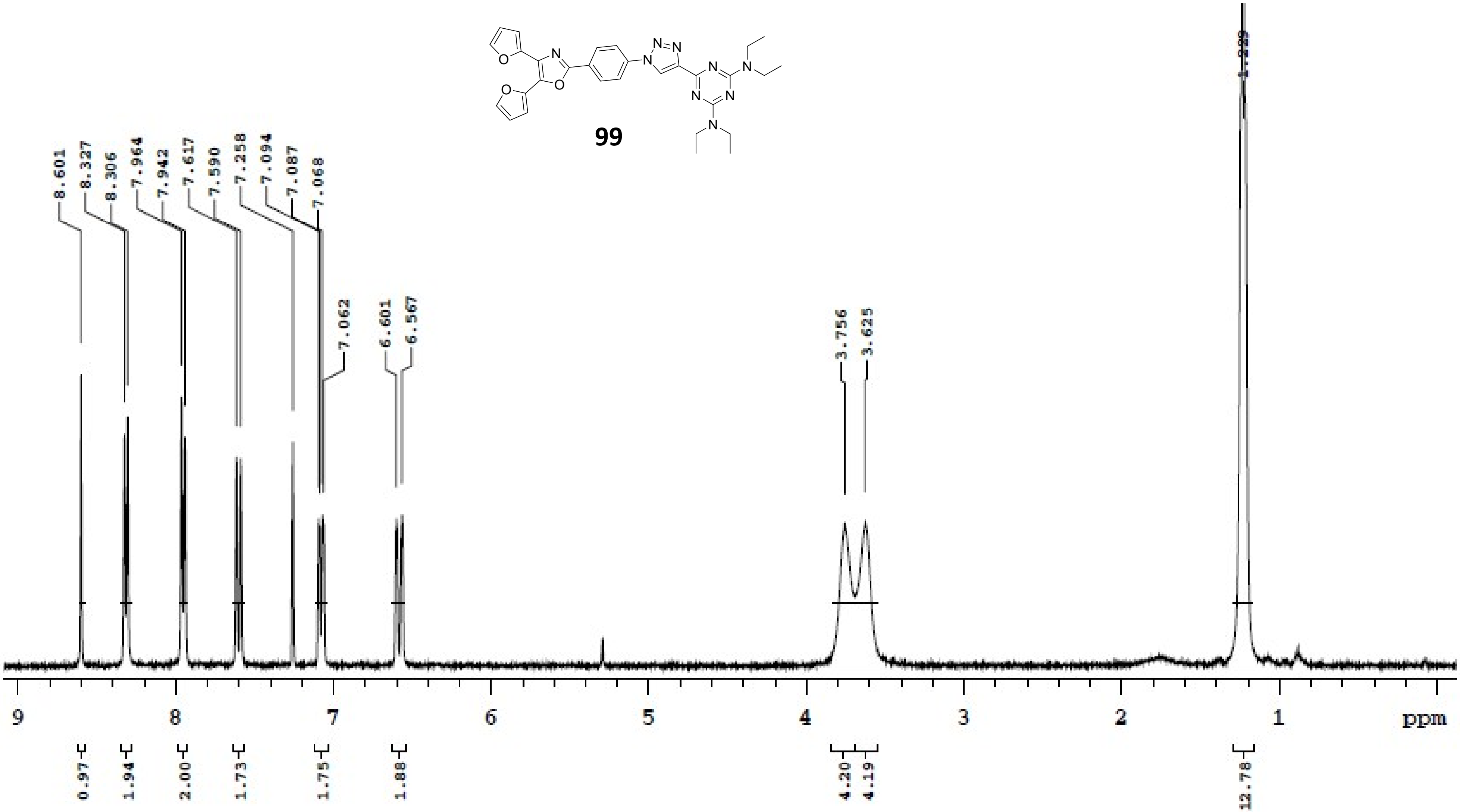

^{13}C NMR: 6-(1-(4-(4,5-di(furan-2-yl)oxazol-2-yl)phenyl)-1H-1,2,3-triazol-4-yl)-N2,N2,N4,N4-tetraethyl-1,3,5-triazine-2,4-diamine

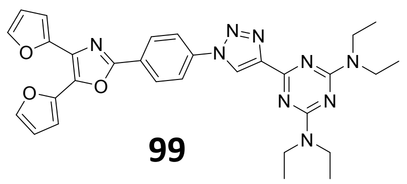

99

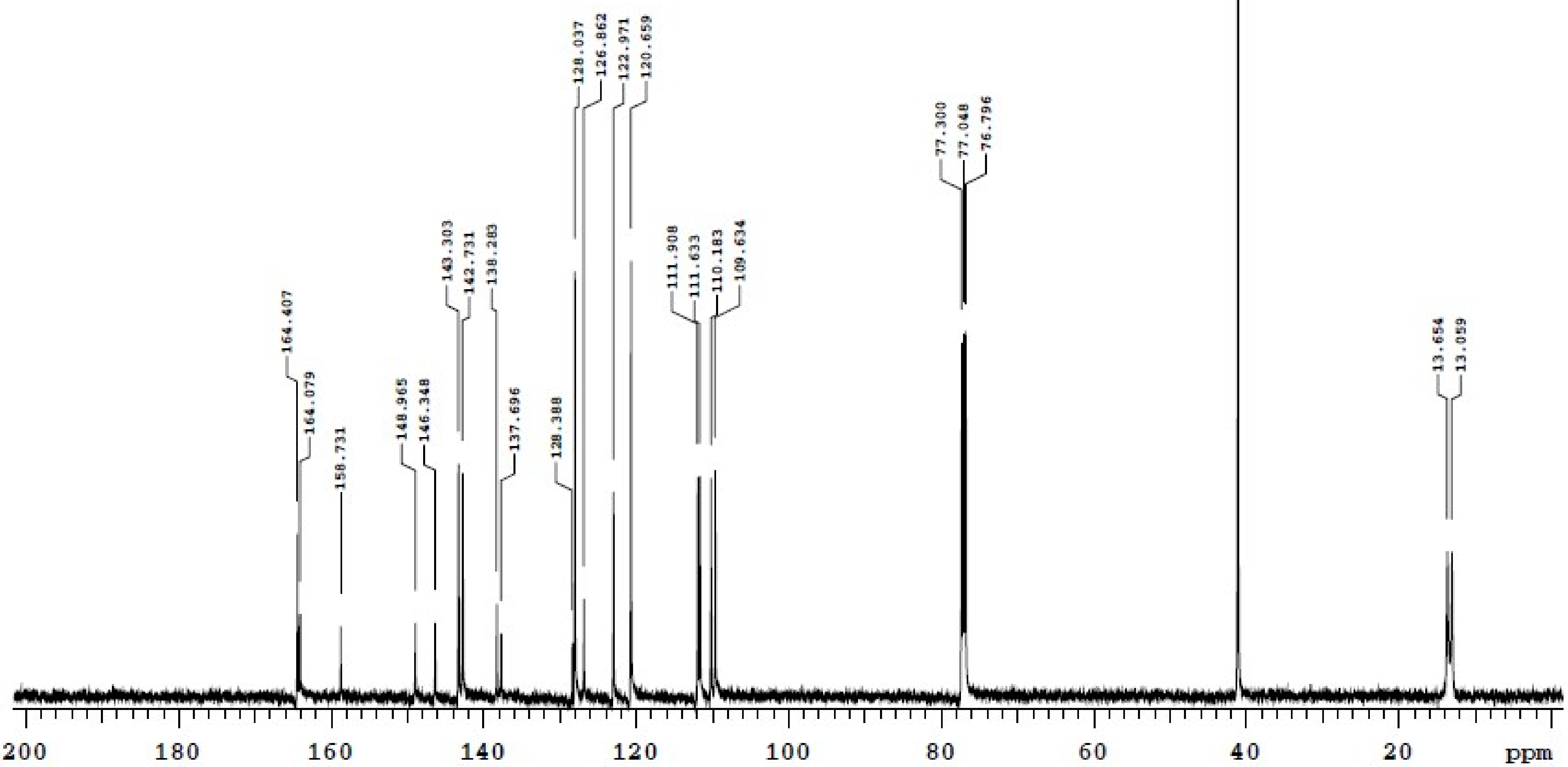

^{1}H NMR: 6-(1-(3-(4,5-diphenyloxazol-2-yl)phenyl)-1H-1,2,3-triazol-4-yl)-N2,N4-bis(2-fluorophenyl)-1,3,5-triazine-2,4-diamine (PCP-VI-166)

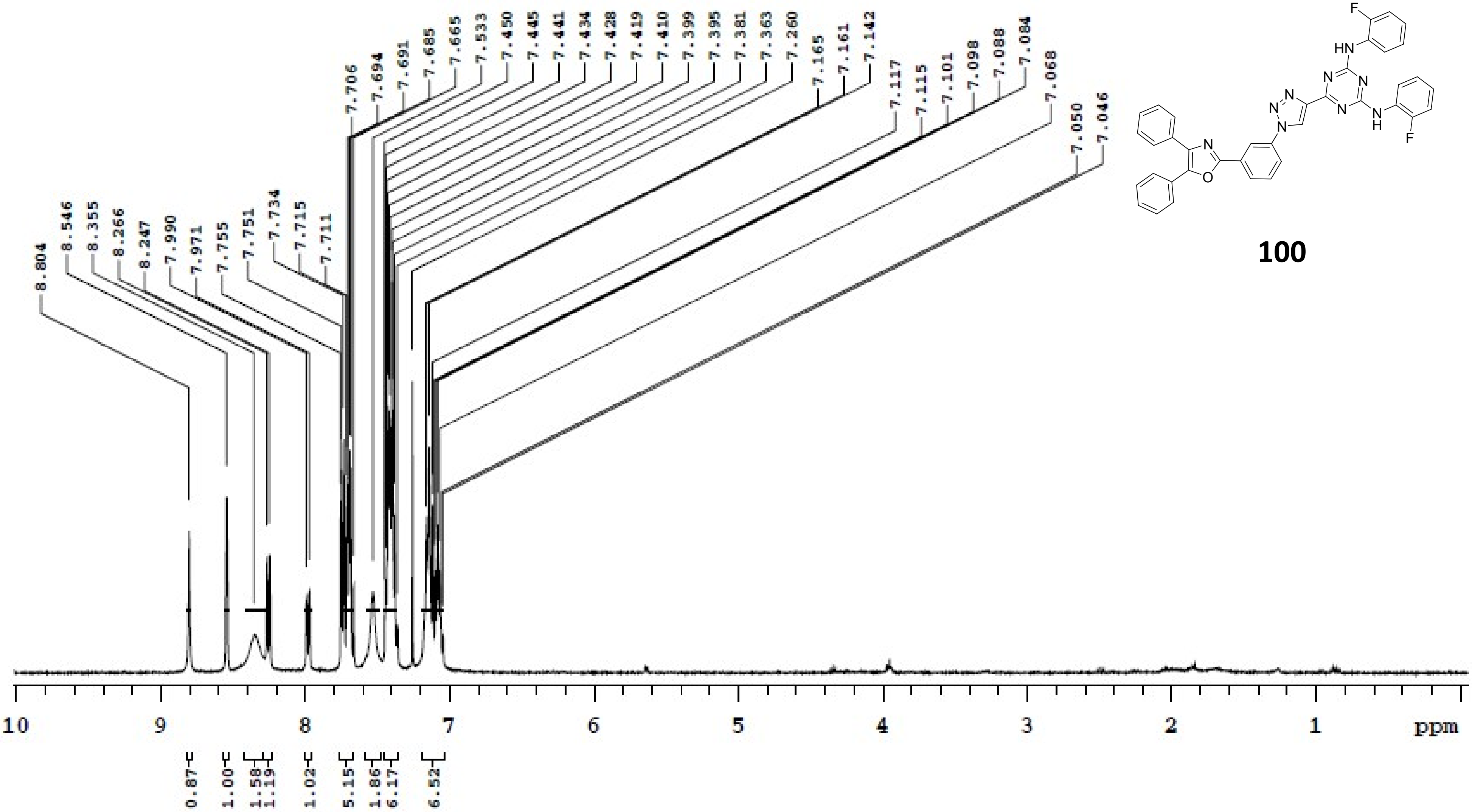

100

^{13}C NMR: 6-(1-(3-(4,5-diphenyloxazol-2-yl)phenyl)-1H-1,2,3-triazol-4-yl)-N2,N4-bis(2-fluorophenyl)-1,3,5-triazine-2,4-diamine

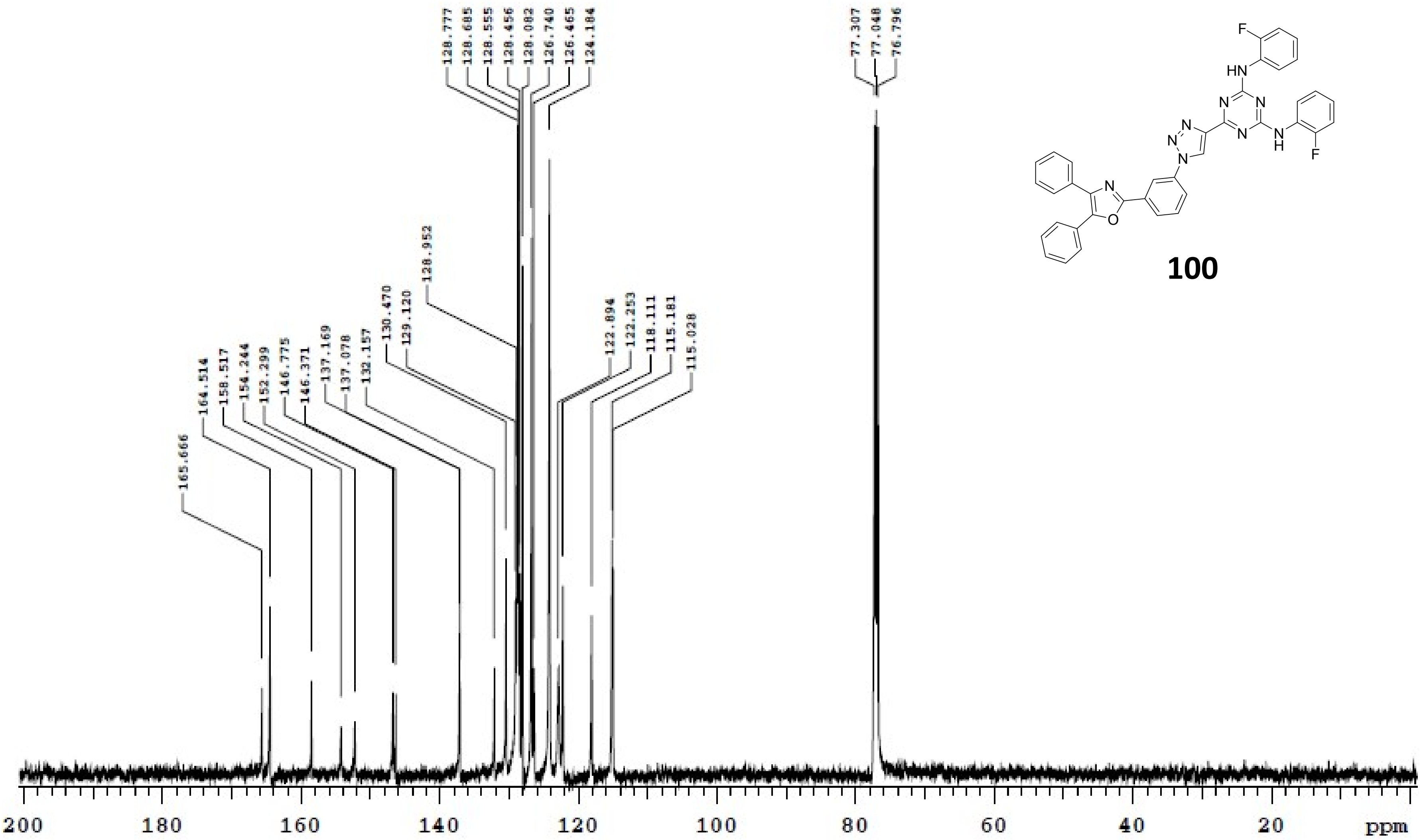

^{1}H NMR: 6-(1-(4-(4,5-diphenyloxazol-2-yl)phenyl)-1H-1,2,3-triazol-4-yl)-N2,N4-bis(2-fluorophenyl)-1,3,5-triazine-2,4-diamine

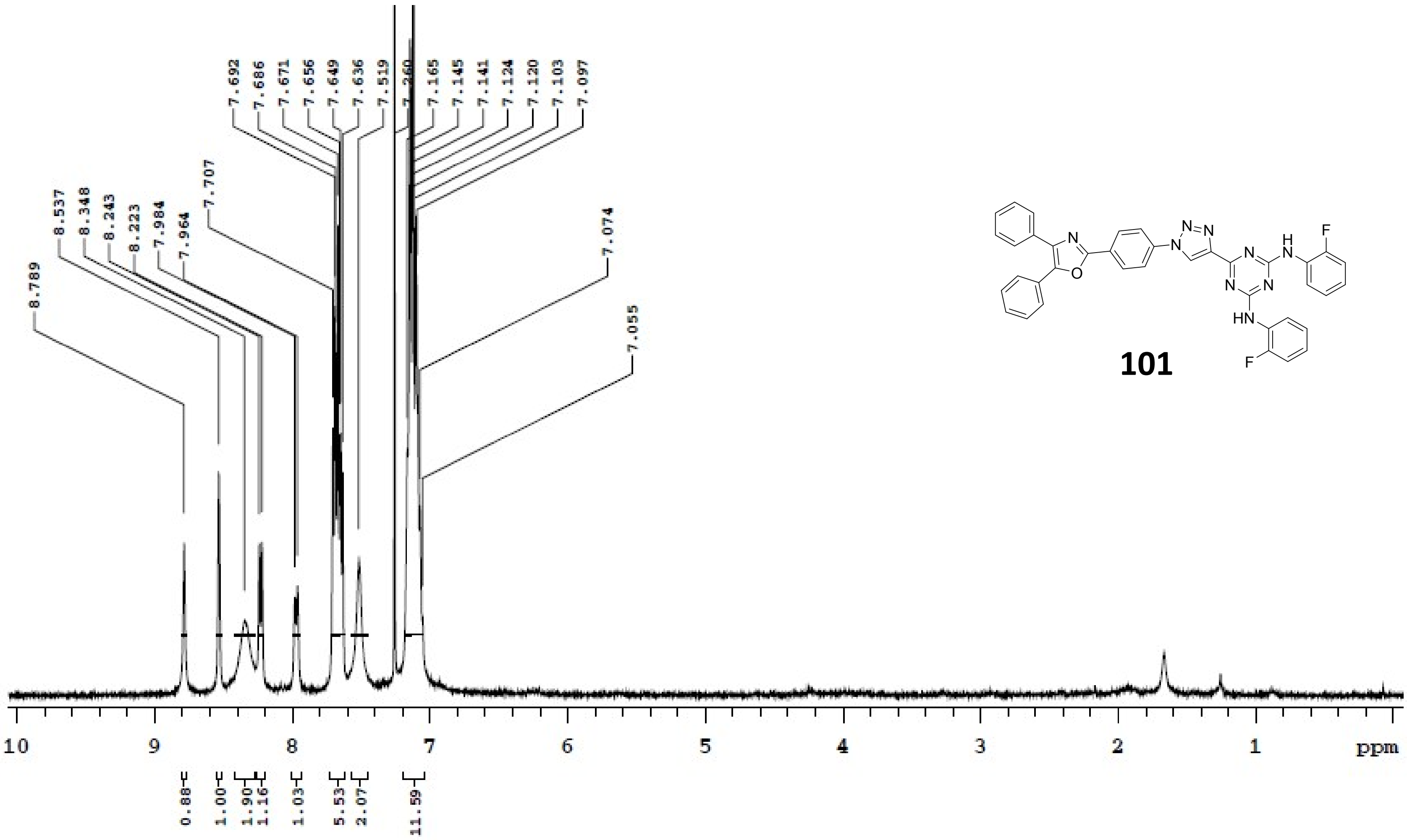

101

^{13}C NMR: 6-(1-(4-(4,5-diphenyloxazol-2-yl)phenyl)-1H-1,2,3-triazol-4-yl)-N2,N4-bis(2-fluorophenyl)-1,3,5-triazine-2,4-diamine

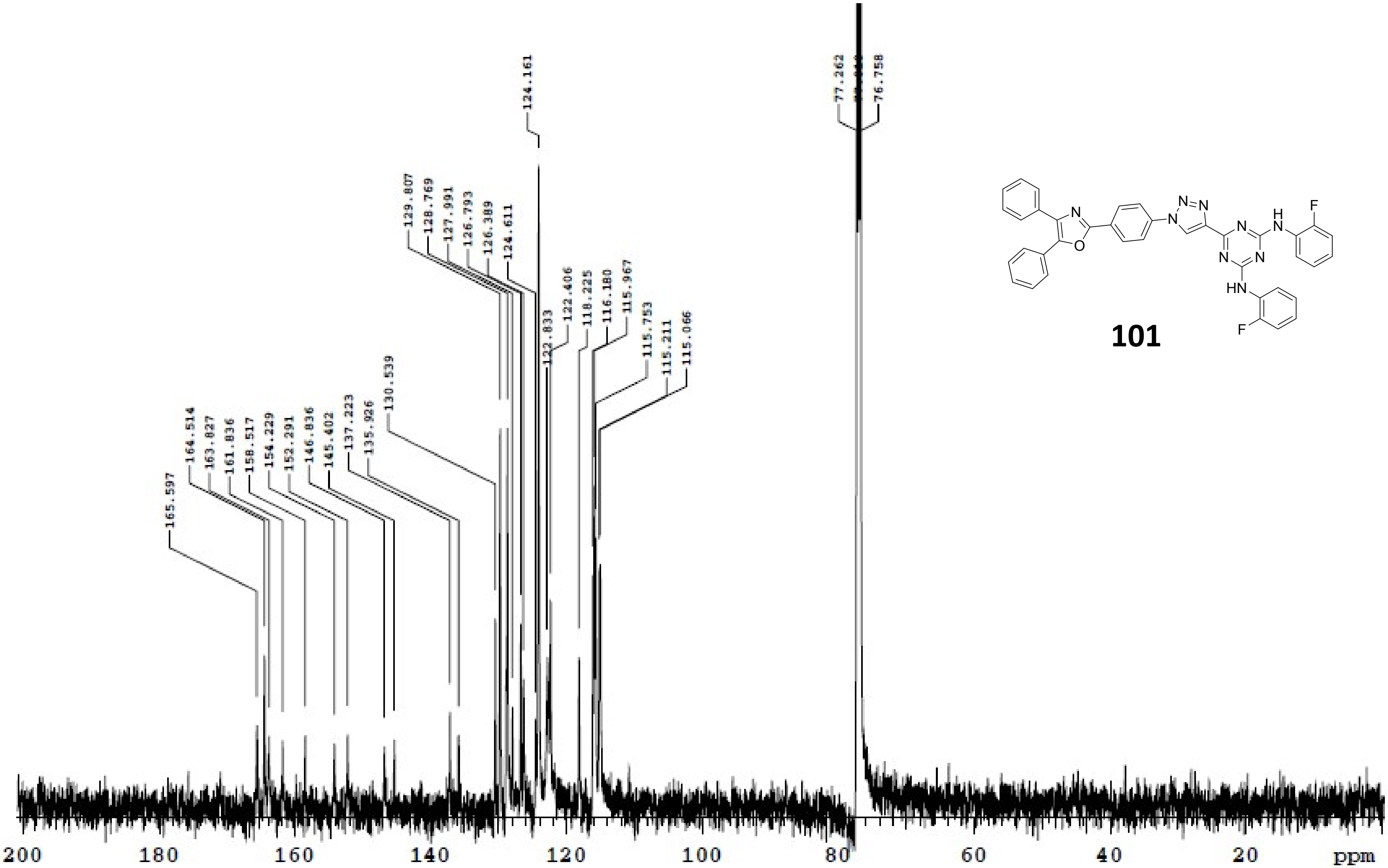

101

^{1}H NMR: 6-(1-(3-(4,5-bis(4-fluorophenyl)oxazol-2-yl)phenyl)-1H-1,2,3-triazol-4-yl)-N2,N4-bis(2-fluorophenyl)-1,3,5-triazine-2,4-diamine

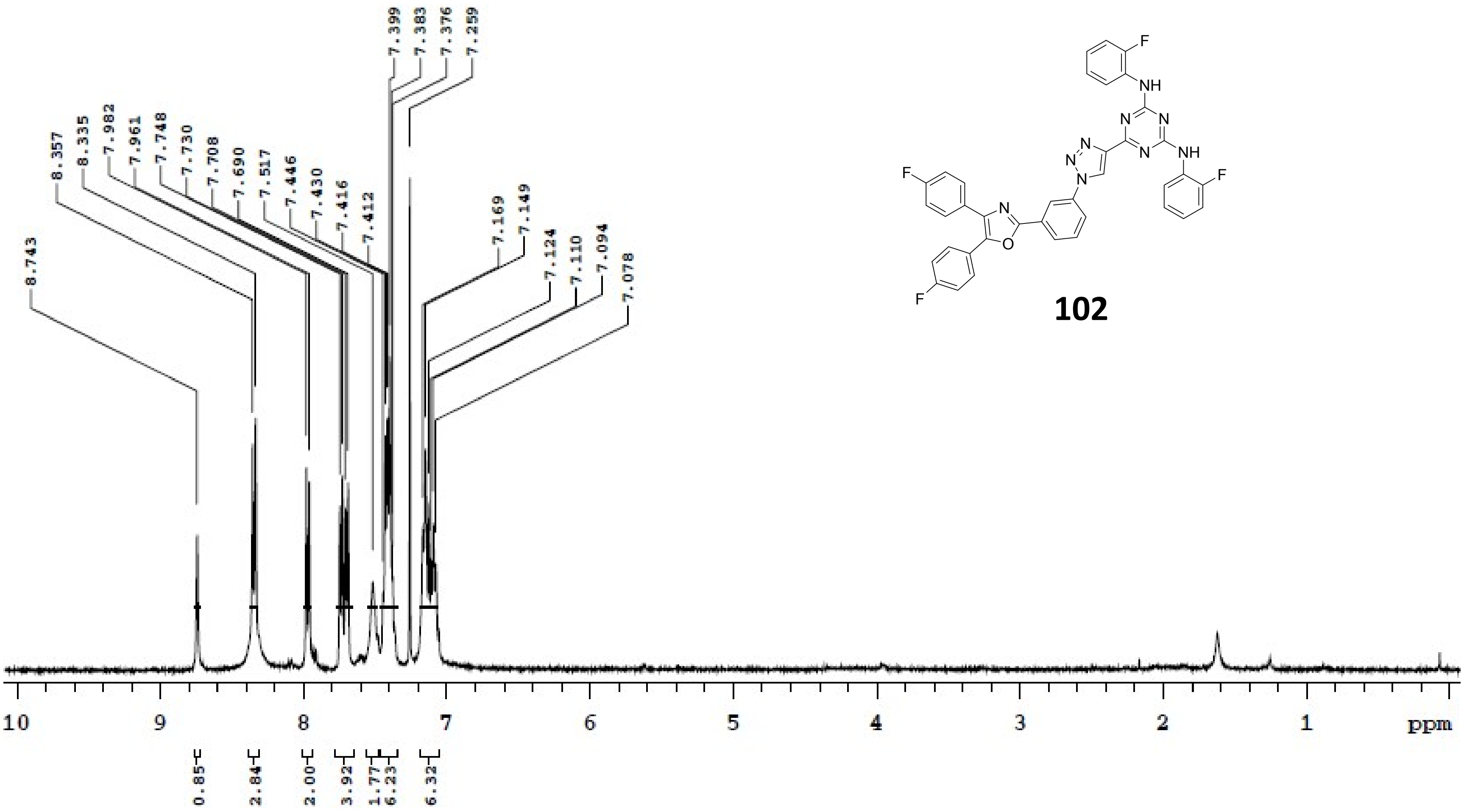

^{13}C NMR: 6-(1-(3-(4,5-bis(4-fluorophenyl)oxazol-2-yl)phenyl)-1H-1,2,3-triazol-4-yl)-N2,N4-bis(2-fluorophenyl)-1,3,5-triazine-2,4-diamine

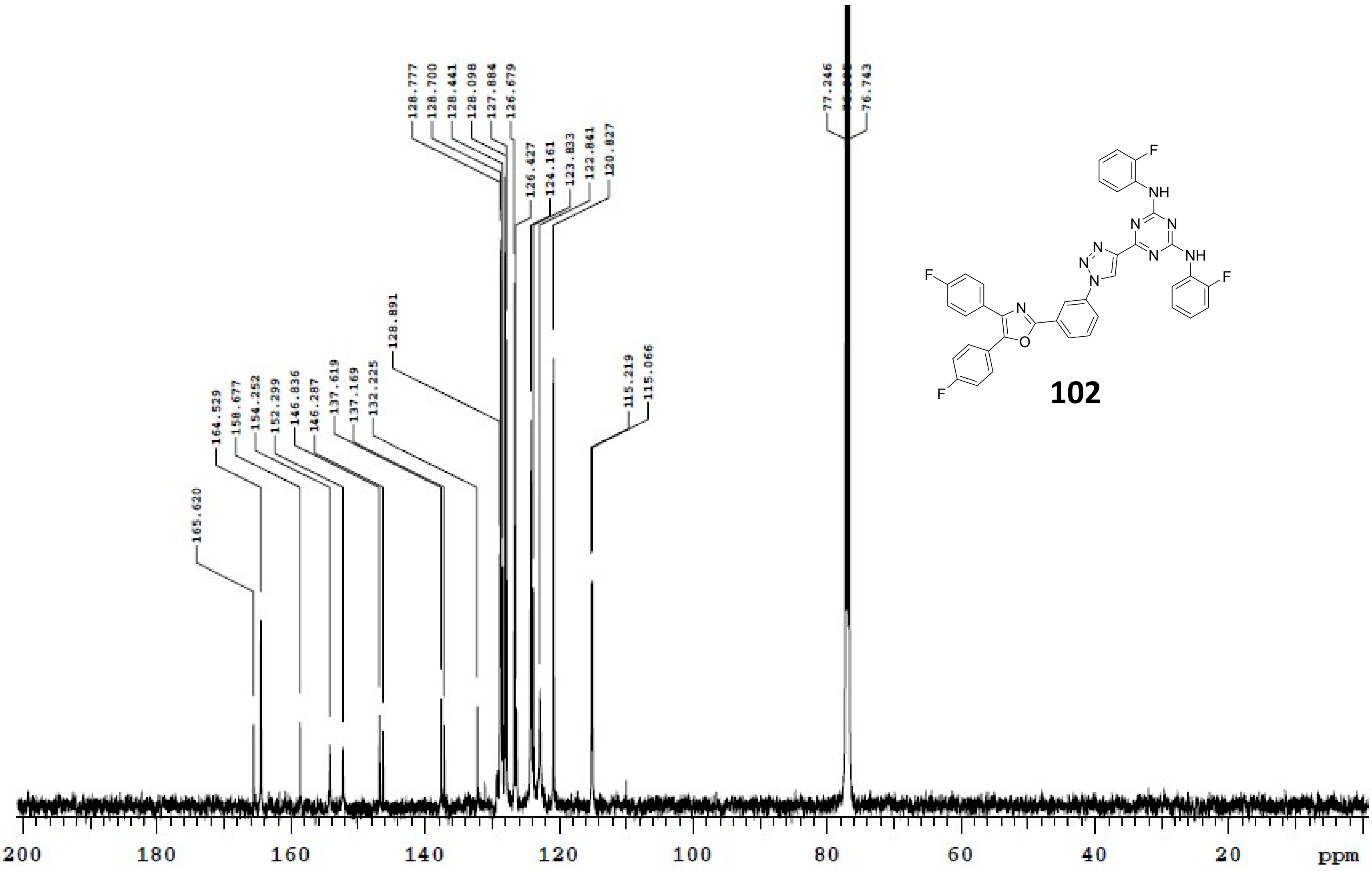

102

^{1}H NMR: 6-(1-(4-(4,5-bis(4-fluorophenyl)oxazol-2-yl)phenyl)-1H-1,2,3-triazol-4-yl)-N2,N4-bis(2-fluorophenyl)-1,3,5-triazine-2,4-diamine

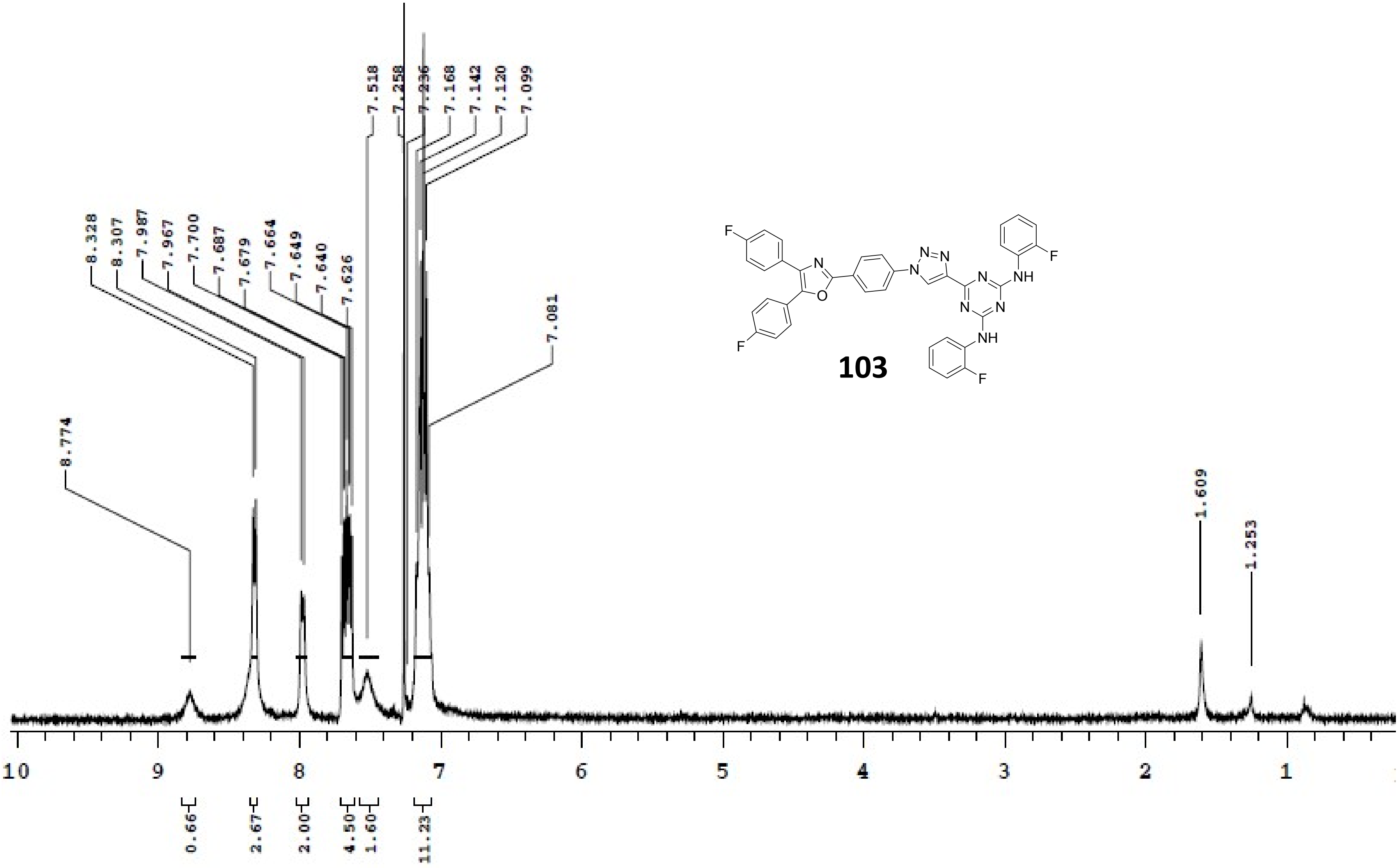

^{13}C NMR: 6-(1-(4-(4,5-bis(4-fluorophenyl)oxazol-2-yl)phenyl)-1H-1,2,3-triazol-4-yl)-N2,N4-bis(2-fluorophenyl)-1,3,5-triazine-2,4-diamine

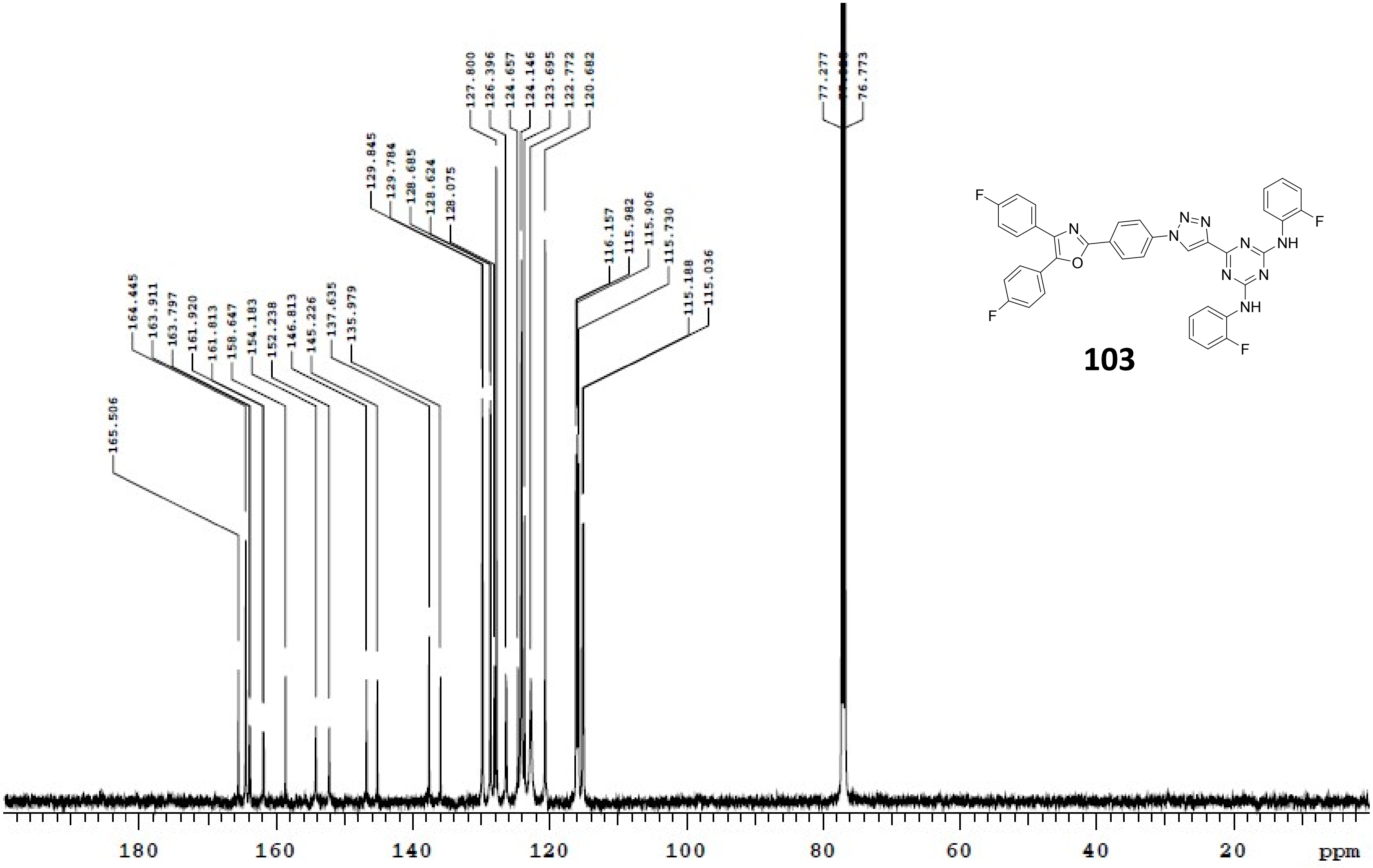

103

^{1}H NMR: 6-(1-(3-(4,5-bis(4-chlorophenyl)oxazol-2-yl)phenyl)-1H-1,2,3-triazol-4-yl)-N2,N4-bis(2-fluorophenyl)-1,3,5-triazine-2,4-diamine

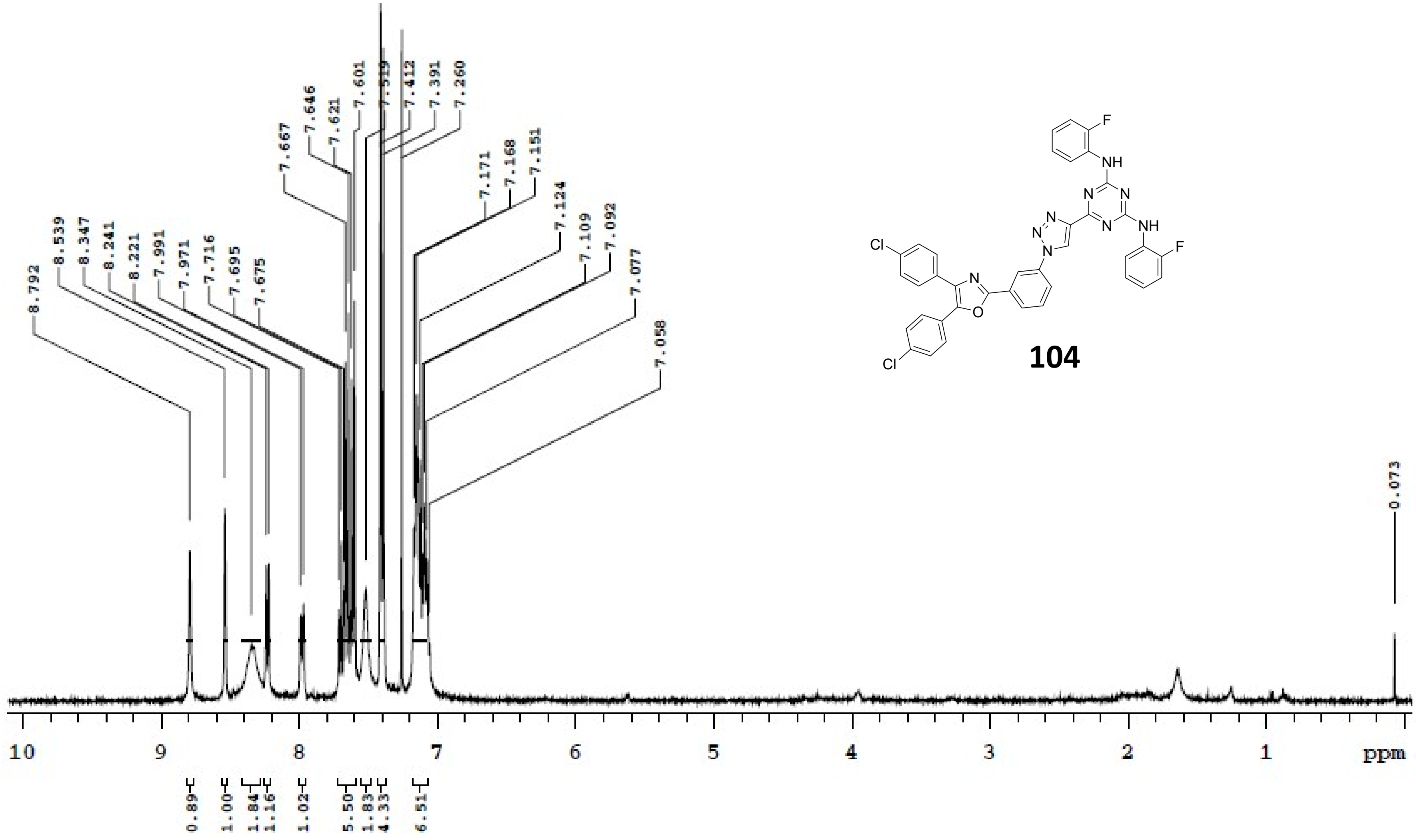

^{13}C NMR: 6-(1-(3-(4,5-bis(4-chlorophenyl)oxazol-2-yl)phenyl)-1H-1,2,3-triazol-4-yl)-N2,N4-bis(2-fluorophenyl)-1,3,5-triazine-2,4-diamine

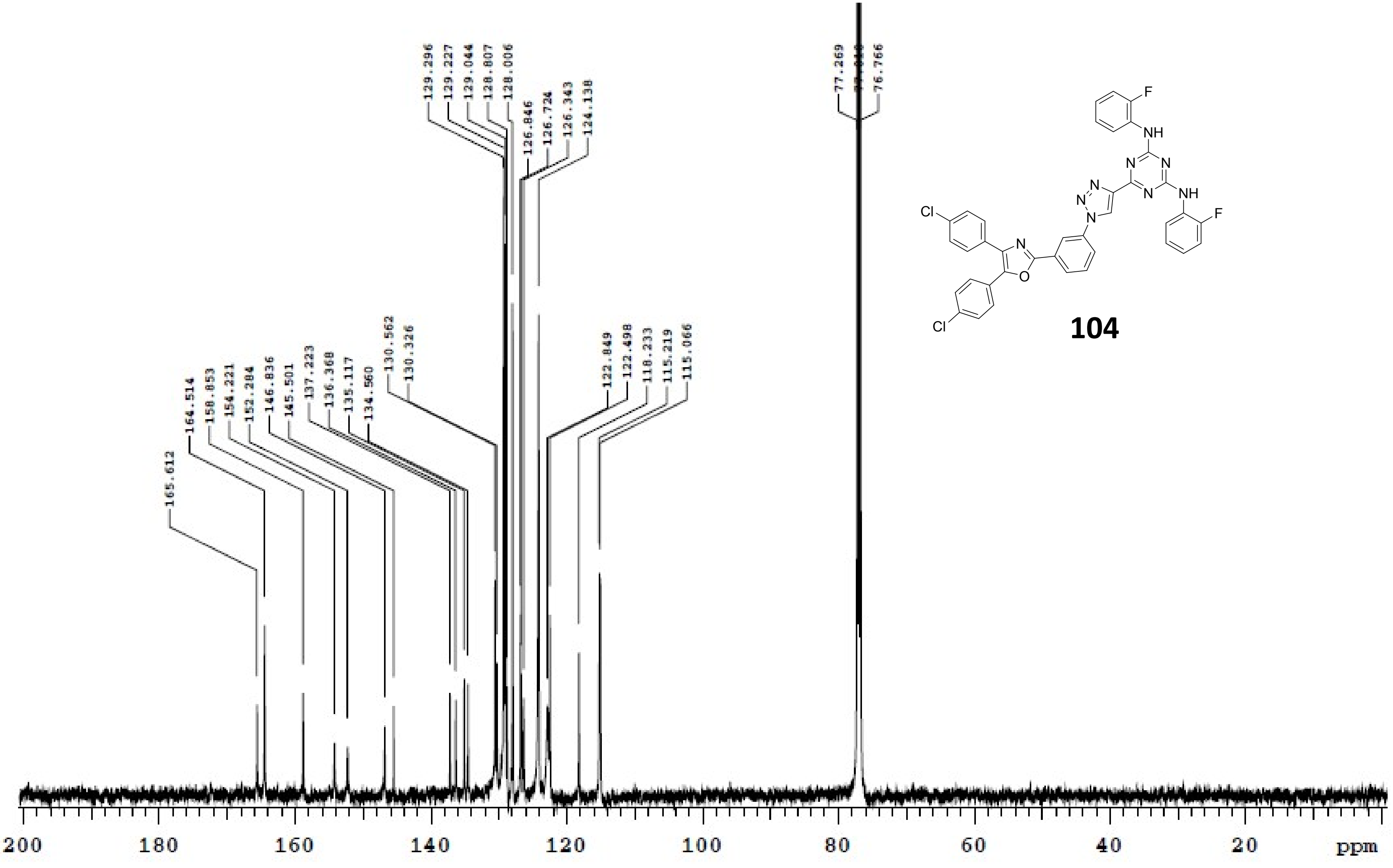

104

^{1}H NMR: 6-(1-(4-(4,5-bis(4-chlorophenyl)oxazol-2-yl)phenyl)-1H-1,2,3-triazol-4-yl)-N2,N4-bis(2-fluorophenyl)-1,3,5-triazine-2,4-diamine

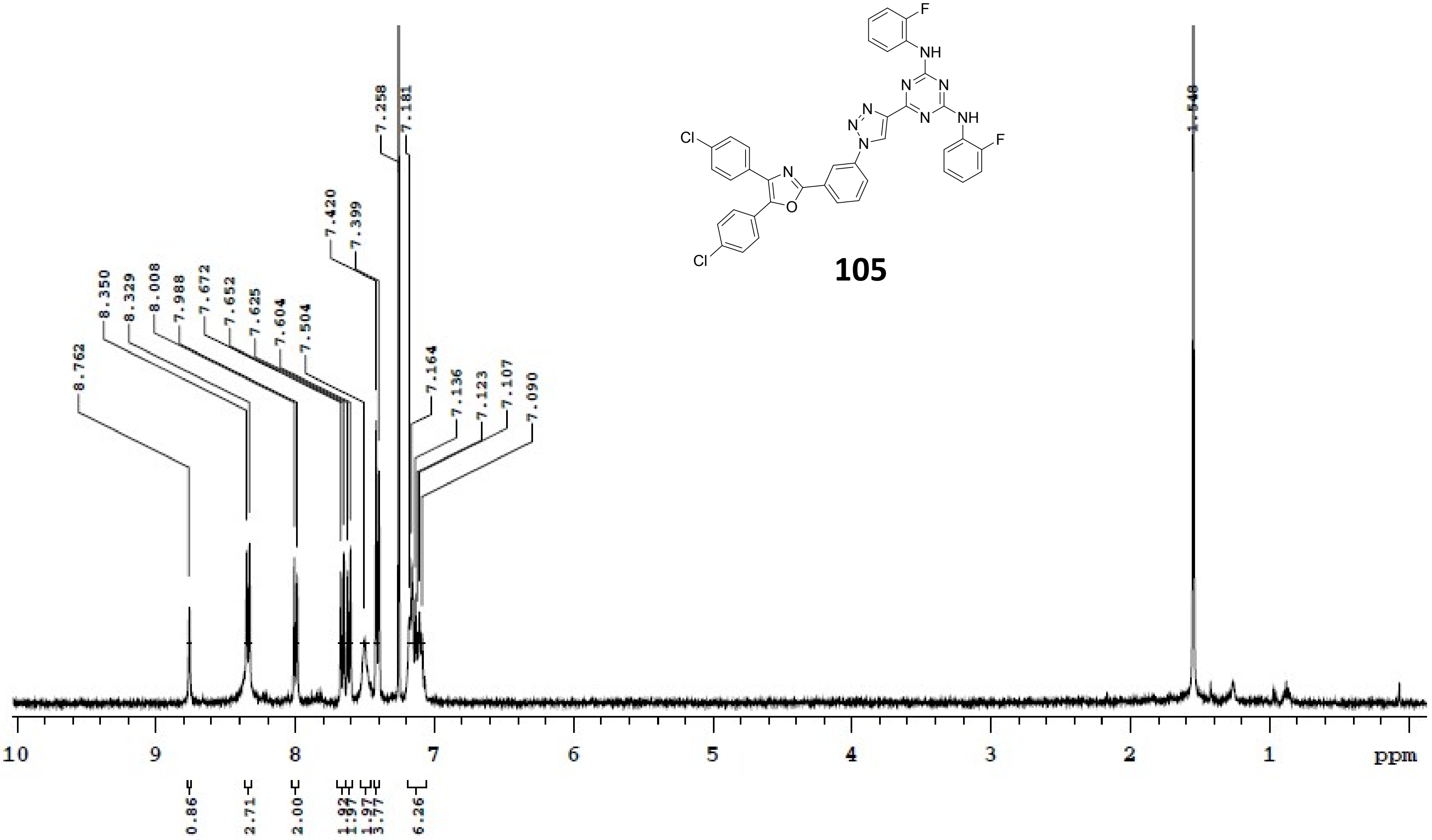

105

^{13}C NMR: 6-(1-(4-(4,5-bis(4-chlorophenyl)oxazol-2-yl)phenyl)-1H-1,2,3-triazol-4-yl)-N2,N4-bis(2-fluorophenyl)-1,3,5-triazine-2,4-diamine

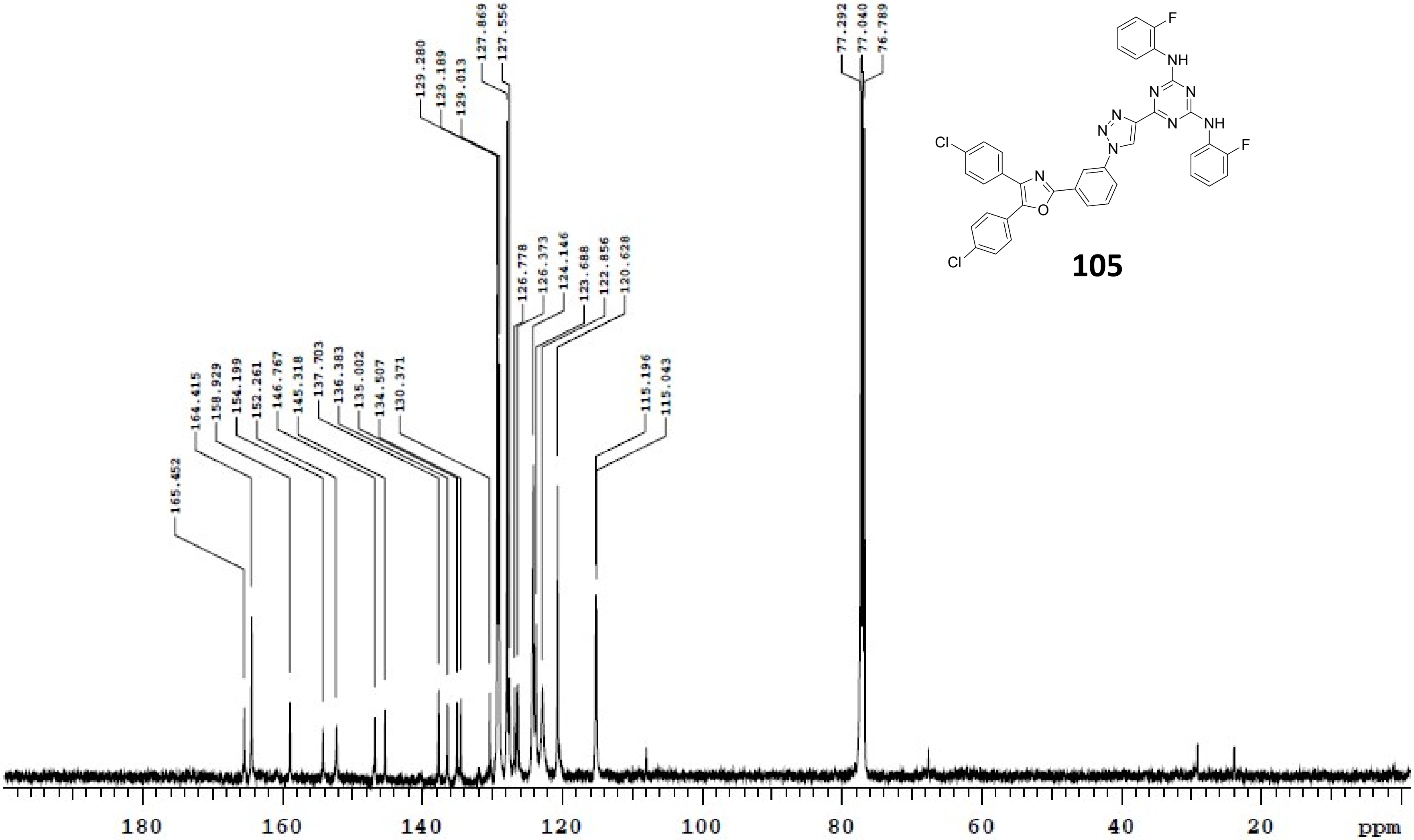

105

^{1}H NMR: 6-(1-(3-(4,5-bis(4-methoxyphenyl)oxazol-2-yl)phenyl)-1H-1,2,3-triazol-4-yl)-N2,N4-bis(2-fluorophenyl)-1,3,5-triazine-2,4-diamine

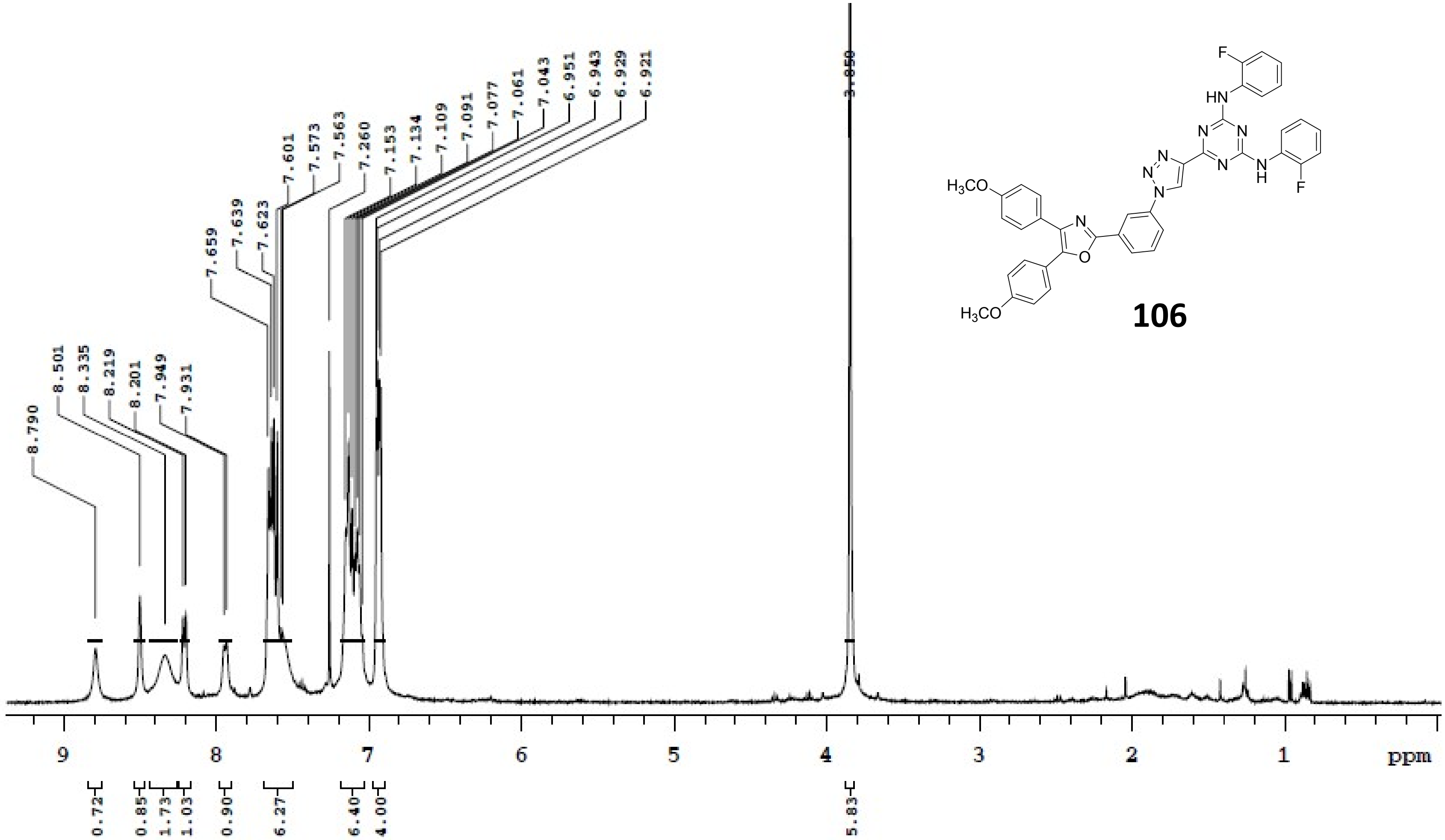

^{13}C NMR: 6-(1-(3-(4,5-bis(4-methoxyphenyl)oxazol-2-yl)phenyl)-1H-1,2,3-triazol-4-yl)-N2,N4-bis(2-fluorophenyl)-1,3,5-triazine-2,4-diamine

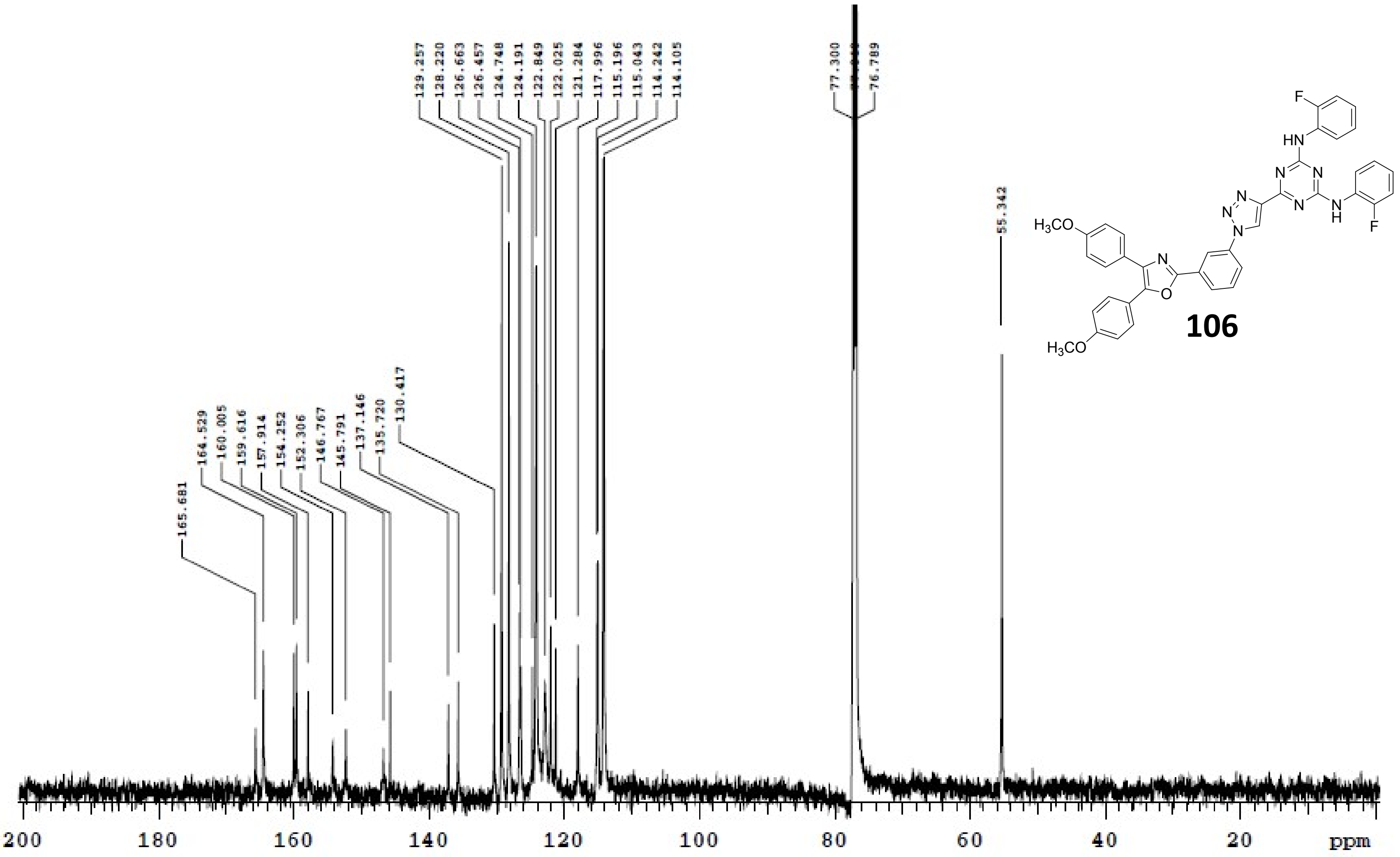

^{1}H NMR: 6-(1-(4-(4,5-bis(4-methoxyphenyl)oxazol-2-yl)phenyl)-1H-1,2,3-triazol-4-yl)-N2,N4-bis(2-fluorophenyl)-1,3,5-triazine-2,4-diamine

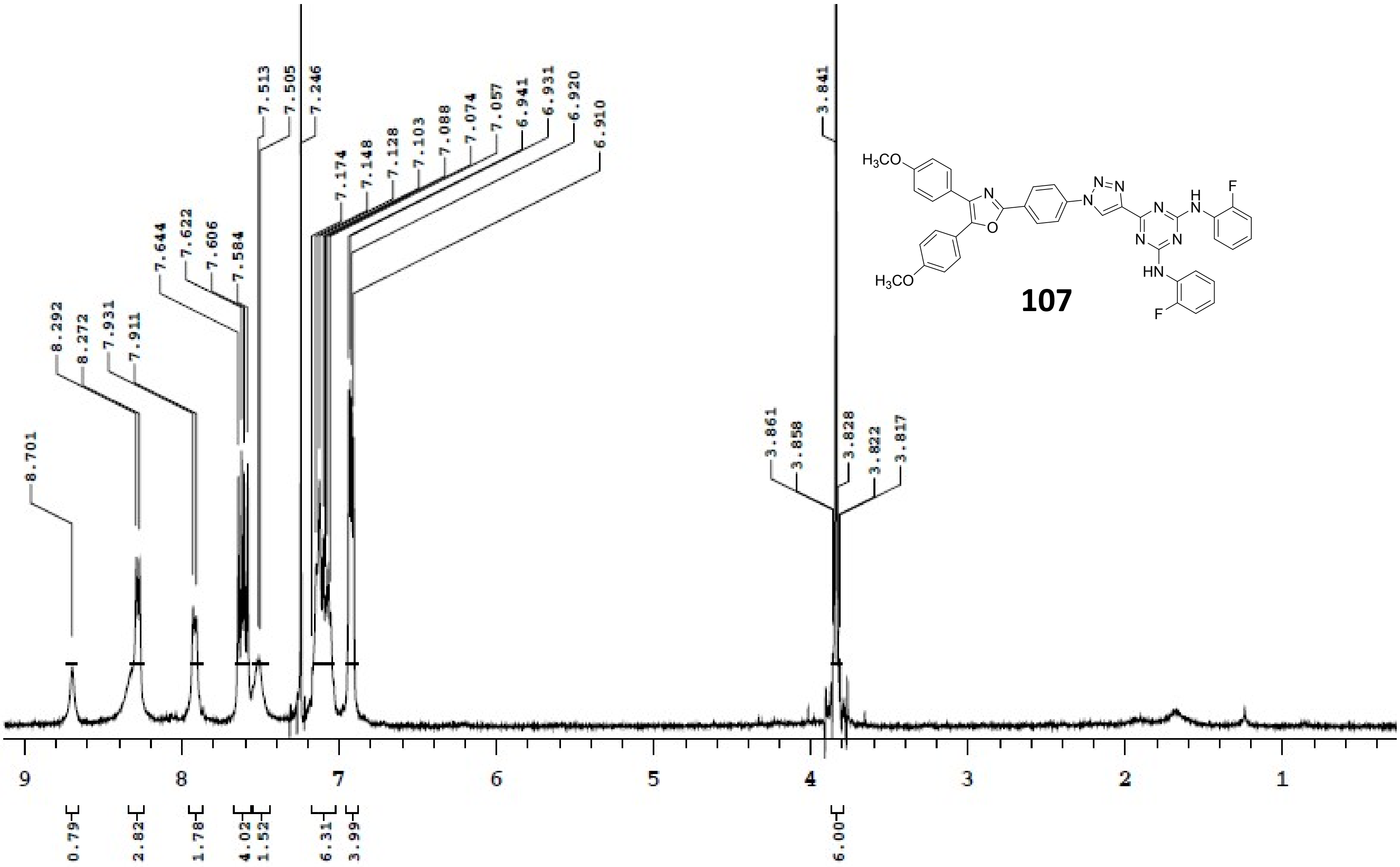

^{13}C NMR: 6-(1-(4-(4,5-bis(4-methoxyphenyl)oxazol-2-yl)phenyl)-1H-1,2,3-triazol-4-yl)-N2,N4-bis(2-fluorophenyl)-1,3,5-triazine-2,4-diamine

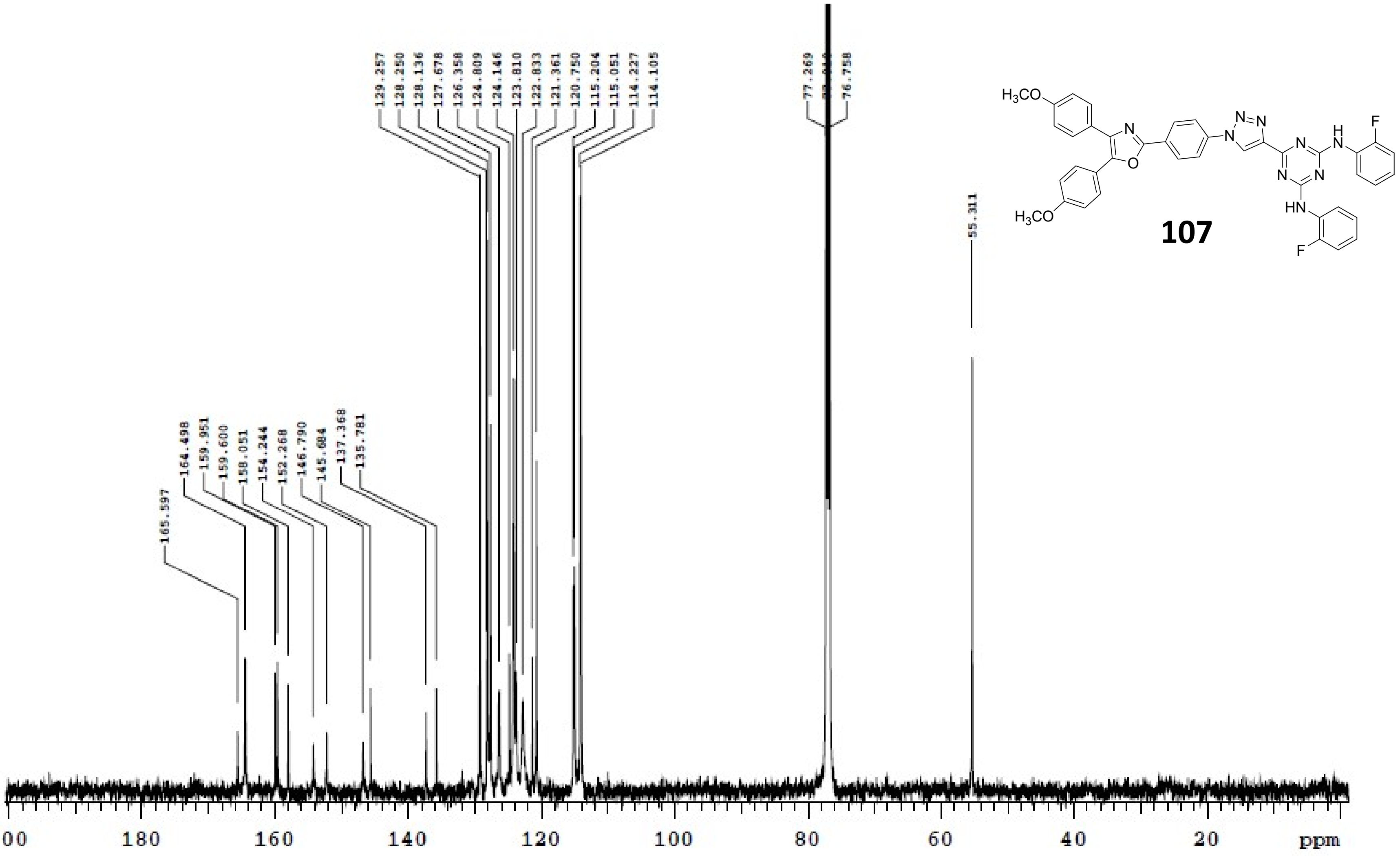

107

^{1}H NMR: 6-(1-(3-(4,5-di(furan-2-yl)oxazol-2-yl)phenyl)-1H-1,2,3-triazol-4-yl)-N2,N4-bis(2-fluorophenyl)-1,3,5-triazine-2,4-diamine

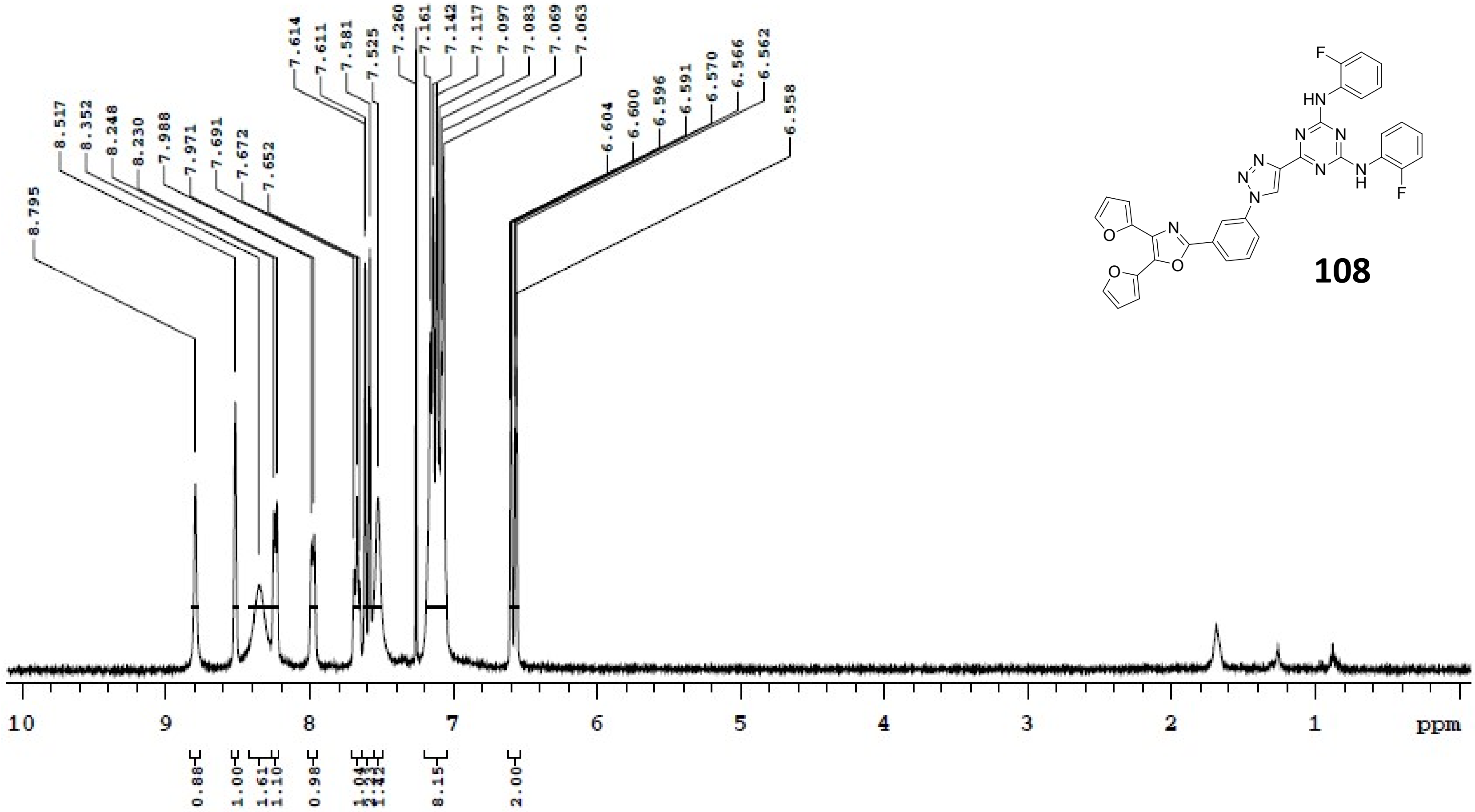

108

^{13}C NMR: 6-(1-(3-(4,5-di(furan-2-yl)oxazol-2-yl)phenyl)-1H-1,2,3-triazol-4-yl)-N2,N4-bis(2-fluorophenyl)-1,3,5-triazine-2,4-diamine

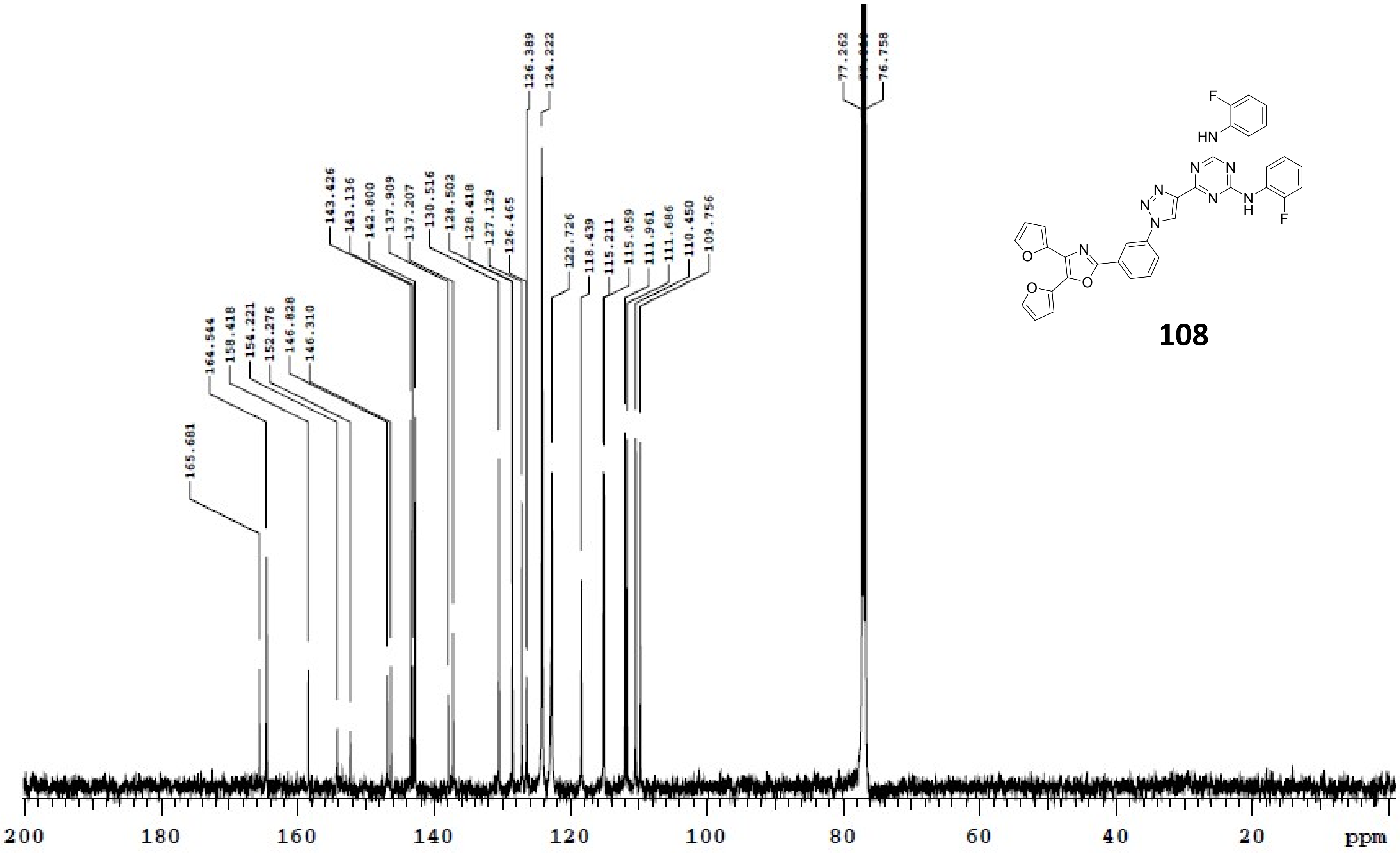

108

^{1}H NMR: 6-(1-(4-(4,5-di(furan-2-yl)oxazol-2-yl)phenyl)-1H-1,2,3-triazol-4-yl)-N2,N4-bis(2-fluorophenyl)-1,3,5-triazine-2,4-diamine

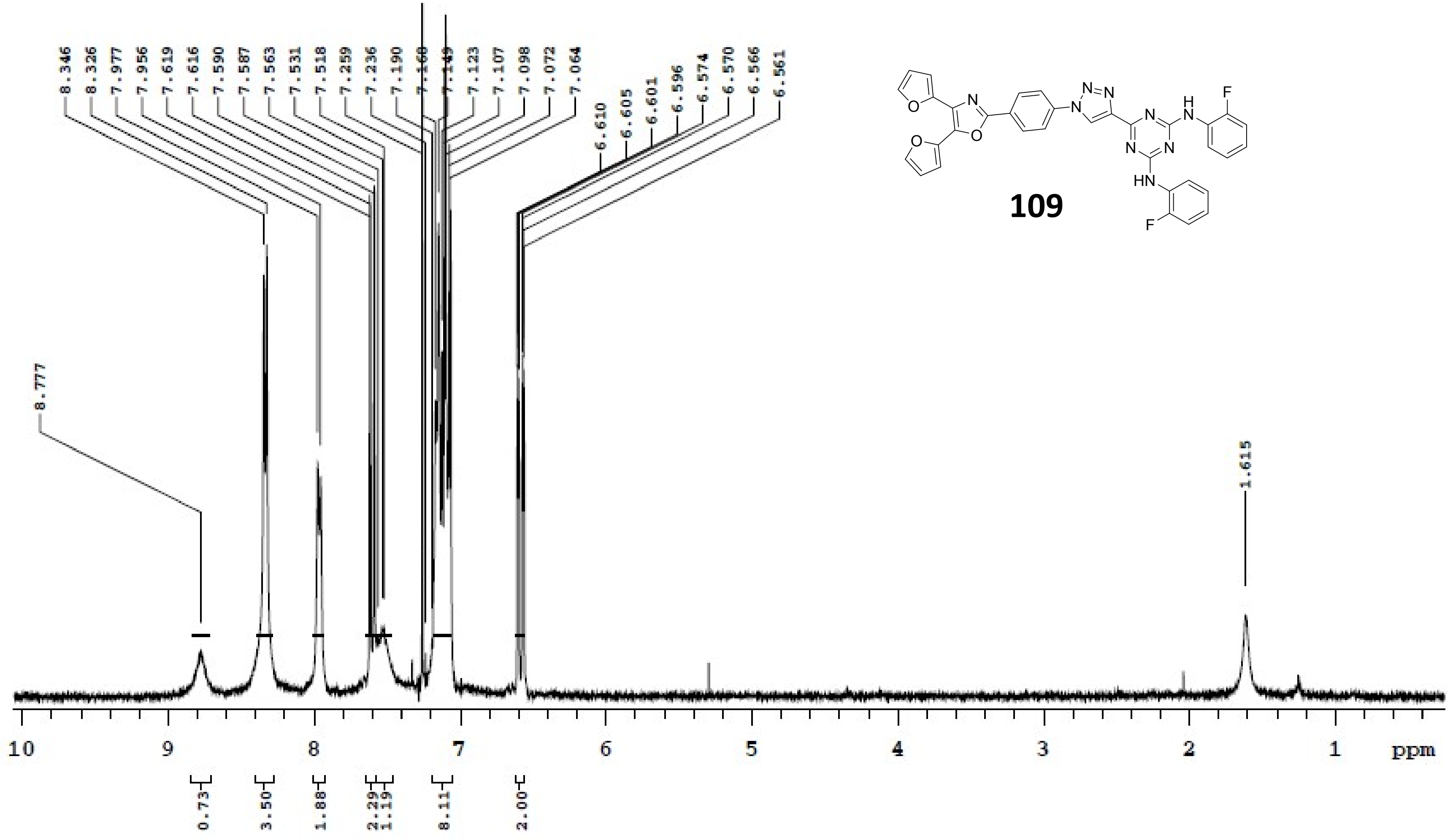

109

^{13}C NMR: 6-(1-(4-(4,5-di(furan-2-yl)oxazol-2-yl)phenyl)-1H-1,2,3-triazol-4-yl)-N2,N4-bis(2-fluorophenyl)-1,3,5-triazine-2,4-diamine

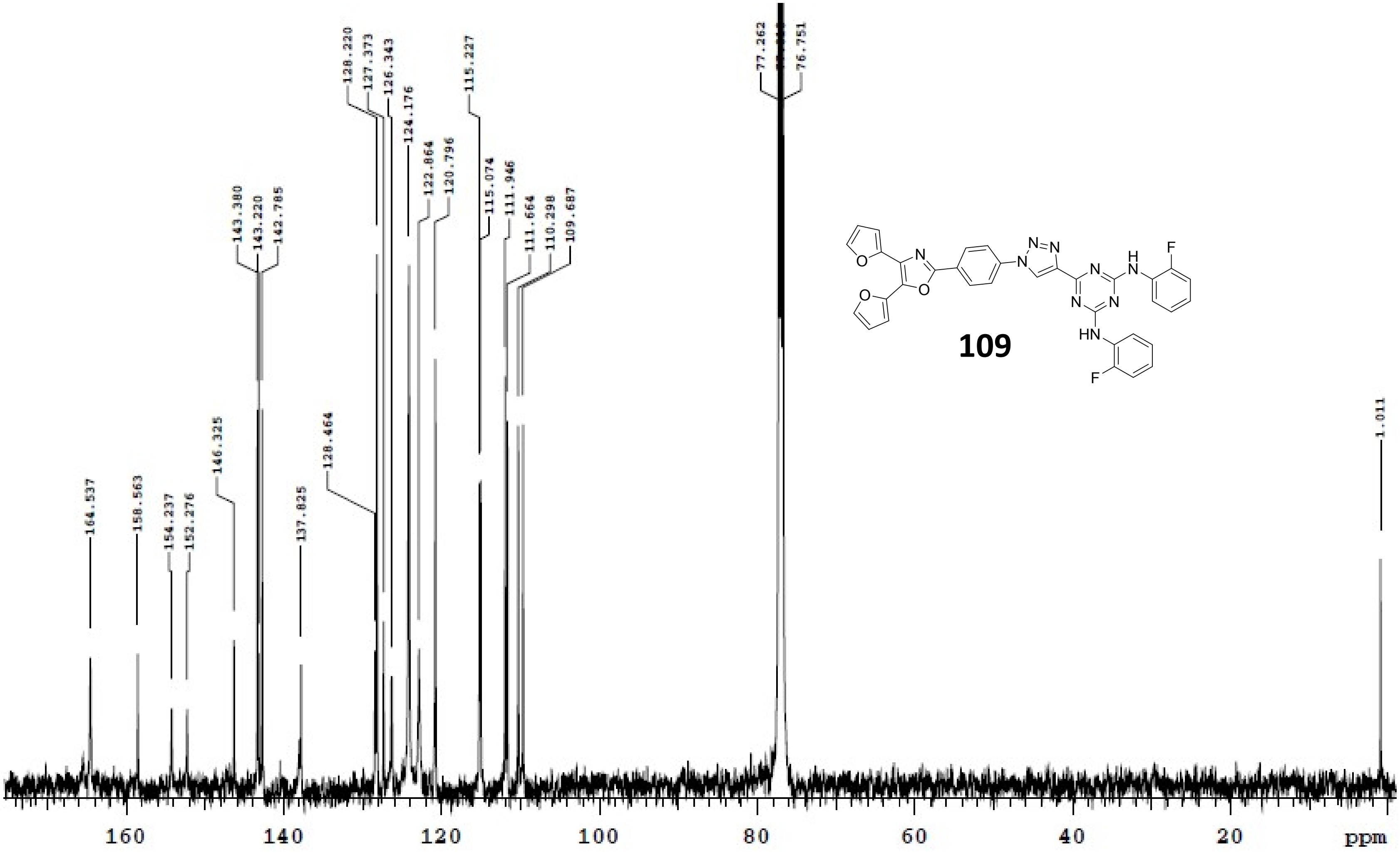

^{1}H NMR: 6-(1-(2-(4,5-diphenyloxazol-2-yl)phenyl)-1H-1,2,3-triazol-4-yl)-N2,N4-bis(4-fluorophenyl)-1,3,5-triazine-2, 4-diamine

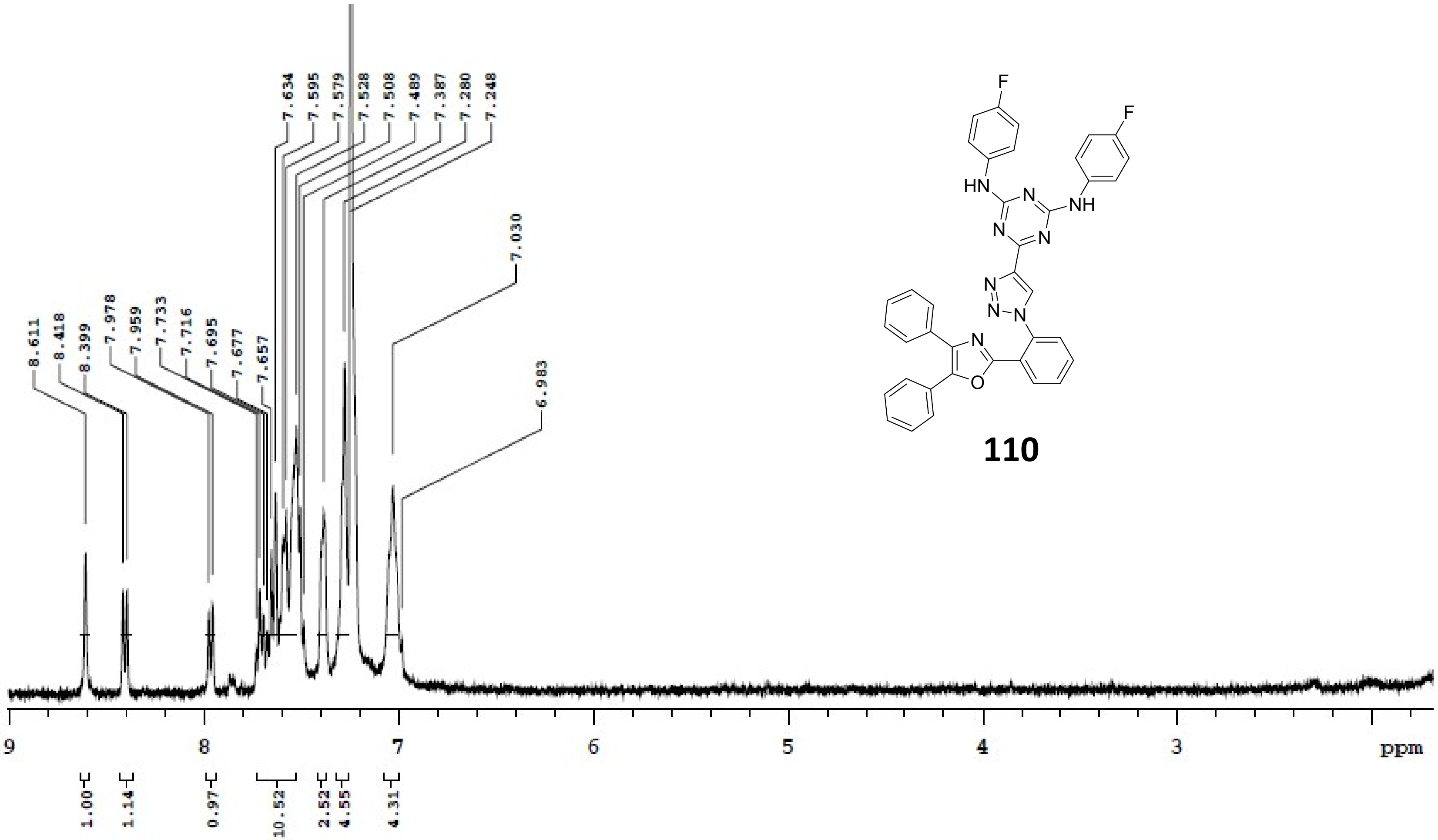

110

^{13}C NMR: 6-(1-(2-(4,5-diphenyloxazol-2-yl)phenyl)-1H-1,2,3-triazol-4-yl)-N2,N4-bis(4-fluorophenyl)-1,3,5-triazine-2,4-diamine

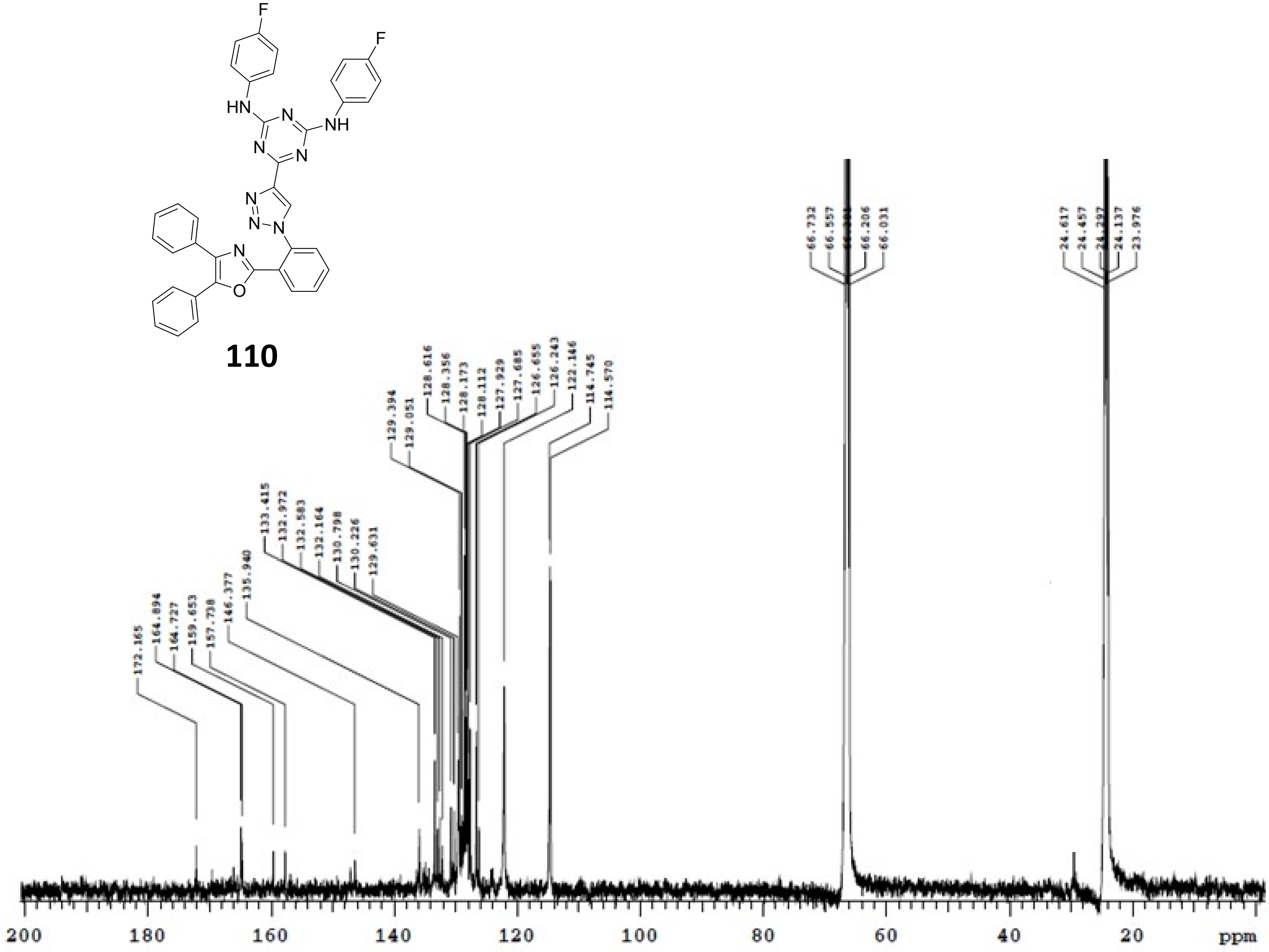

110

^{1}H NMR: 6-(1-(3-(4,5-diphenyloxazol-2-yl)phenyl)-1H-1,2,3-triazol-4-yl)-N2,N4-bis(4-fluorophenyl)-1,3,5-triazine-2,4-diamine

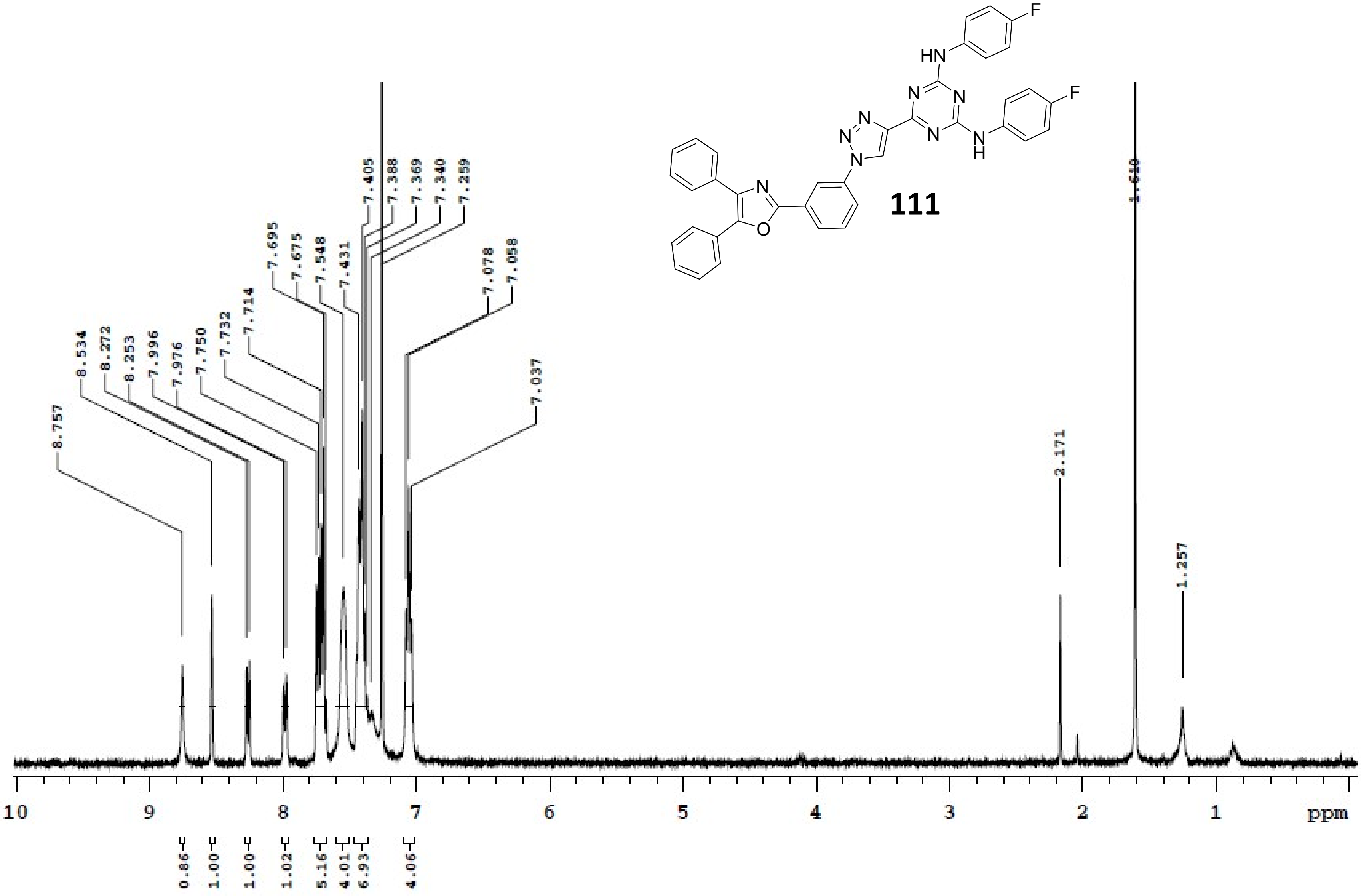

^{13}C NMR: 6-(1-(3-(4,5-diphenyloxazol-2-yl)phenyl)-1H-1,2,3-triazol-4-yl)-N2,N4-bis(4-fluorophenyl)-1,3,5-triazine-2,4-diamine

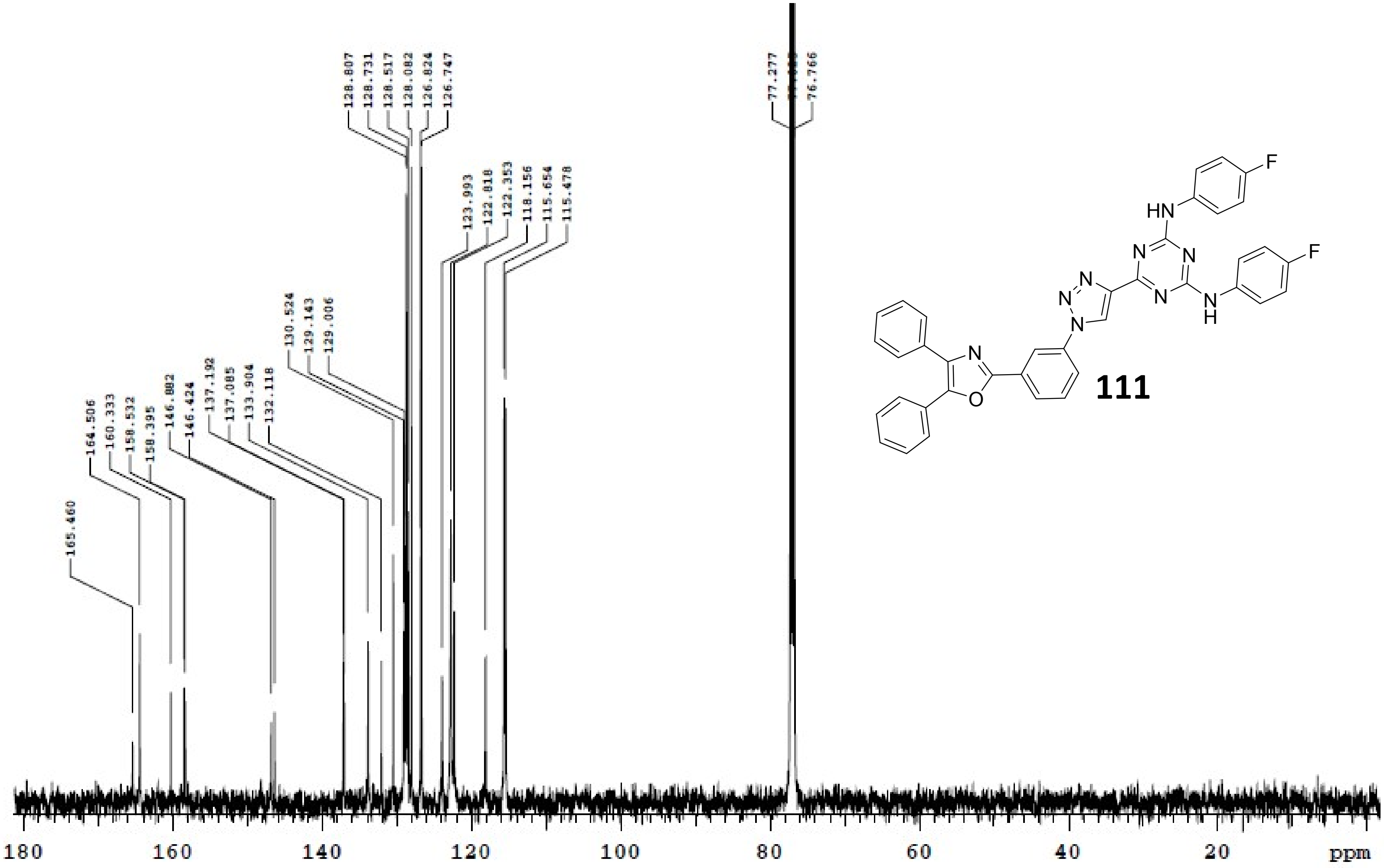

^{1}H NMR: 6-(1-(4-(4,5-diphenyloxazol-2-yl)phenyl)-1H-1,2,3-triazol-4-yl)-N2,N4-bis(4-fluorophenyl)-1,3,5-triazine-2,4-diamine

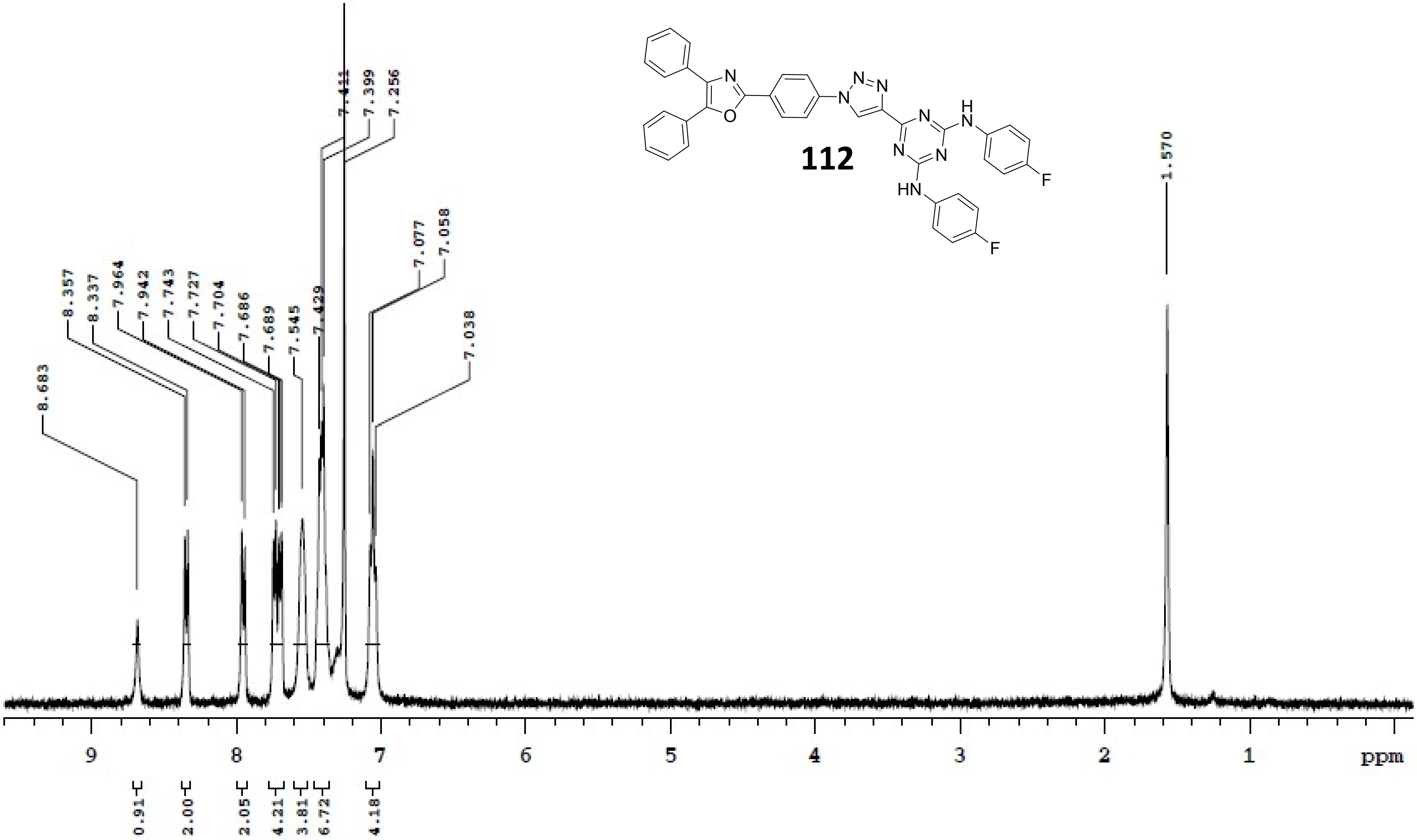

^{13}C NMR: 6-(1-(4-(4,5-diphenyloxazol-2-yl)phenyl)-1H-1,2,3-triazol-4-yl)-N2,N4-bis(4-fluorophenyl)-1,3,5-triazine-2,4-diamine

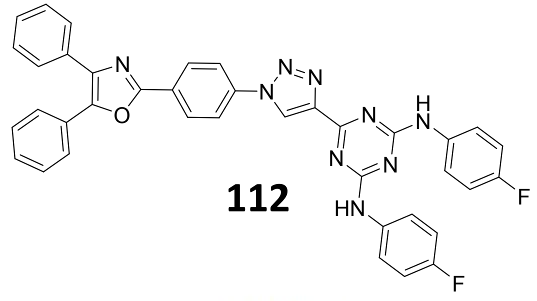

112

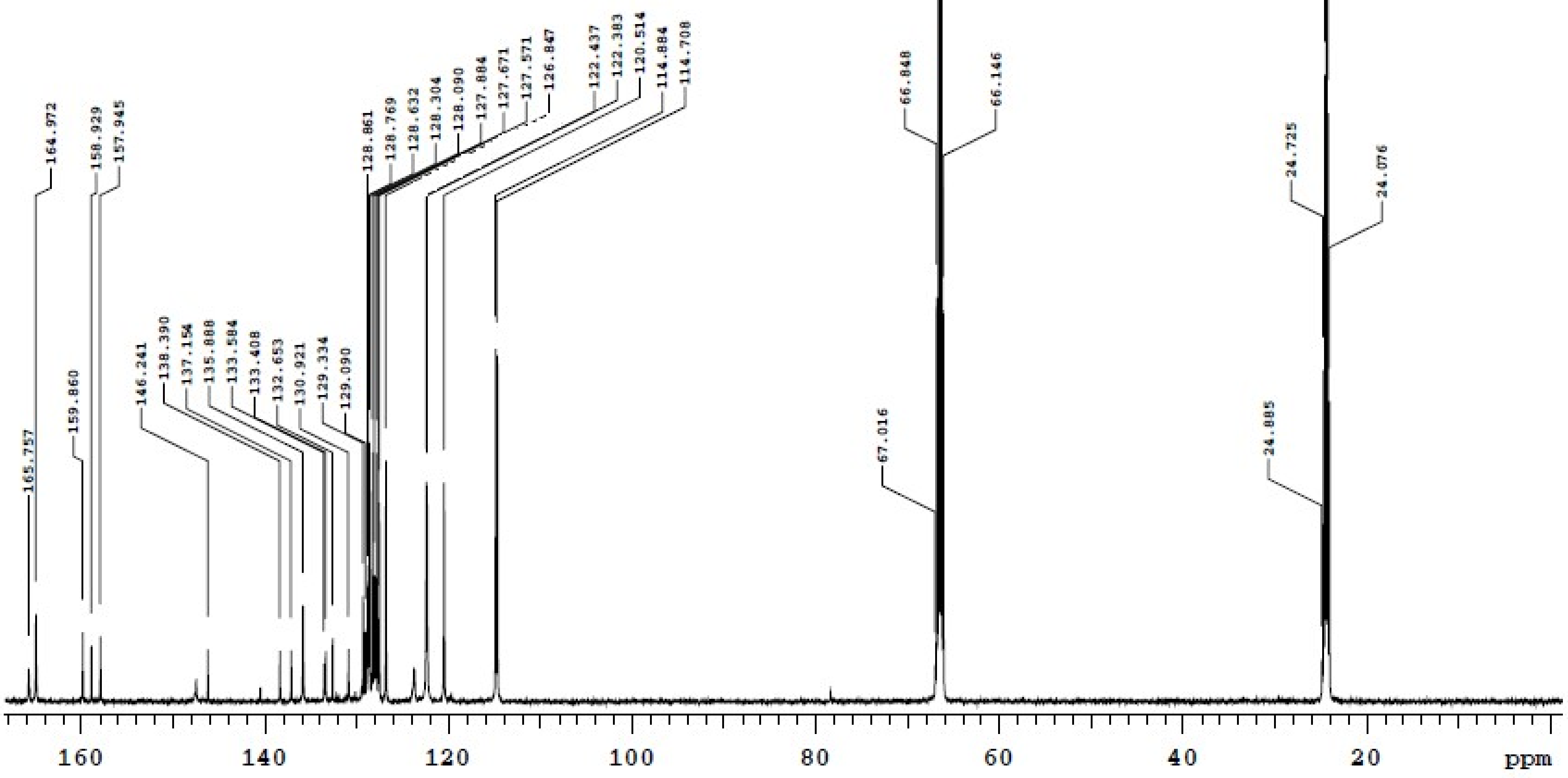

^{1}H NMR: 6-(1-(2-(4,5-bis(4-fluorophenyl)oxazol-2-yl)phenyl)-1H-1,2,3-triazol-4-yl)-N2,N4-bis(4-fluorophenyl)-1,3,5-triazine-2,4-diamine

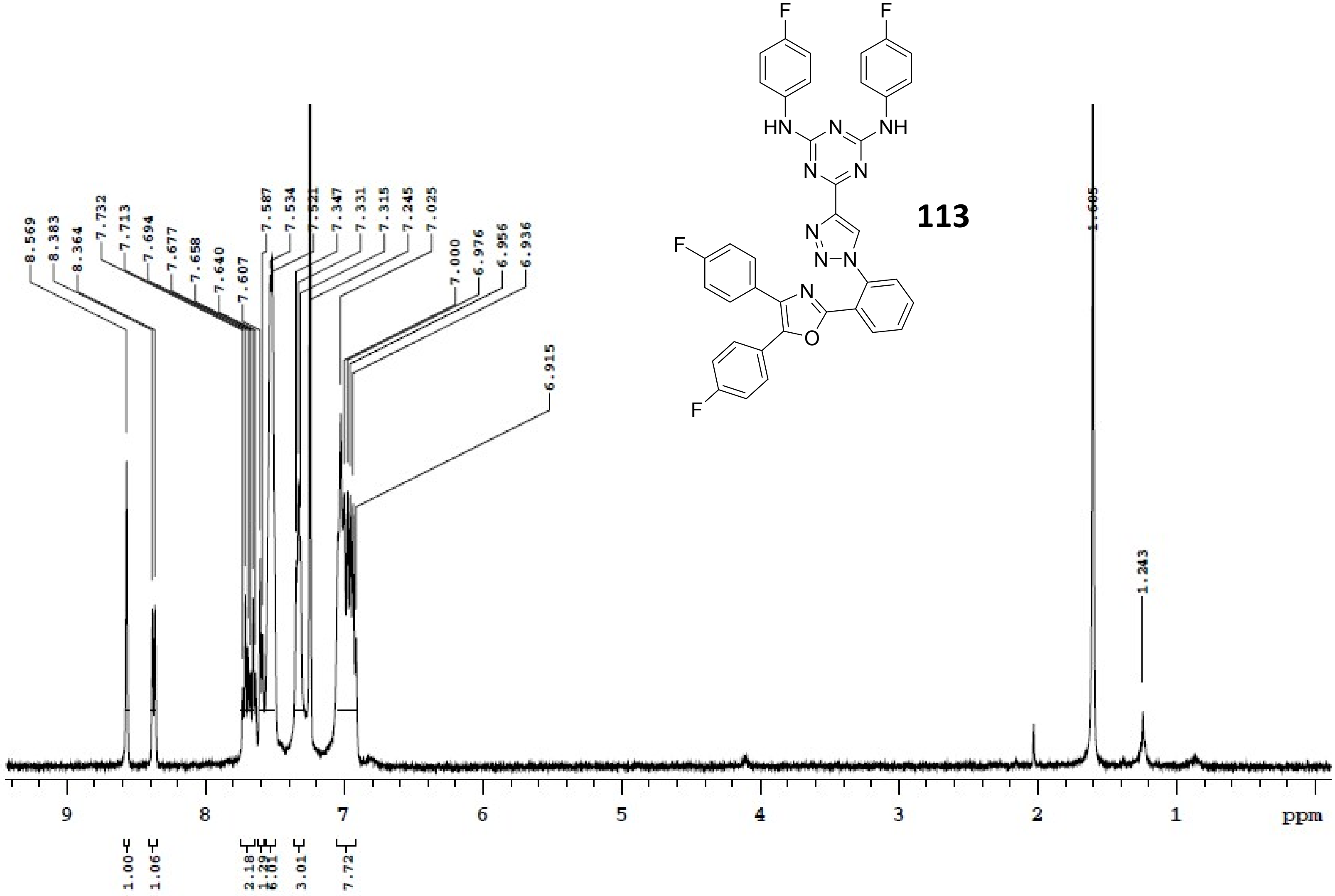

^{13}C NMR: 6-(1-(2-(4,5-bis(4-fluorophenyl)oxazol-2-yl)phenyl)-1H-1,2,3-triazol-4-yl)-N2,N4-bis(4-fluorophenyl)-1,3,5-triazine-2,4-diamine

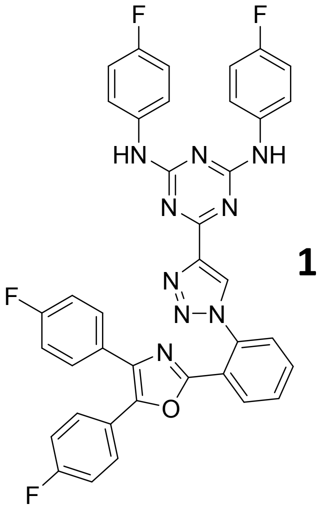

113

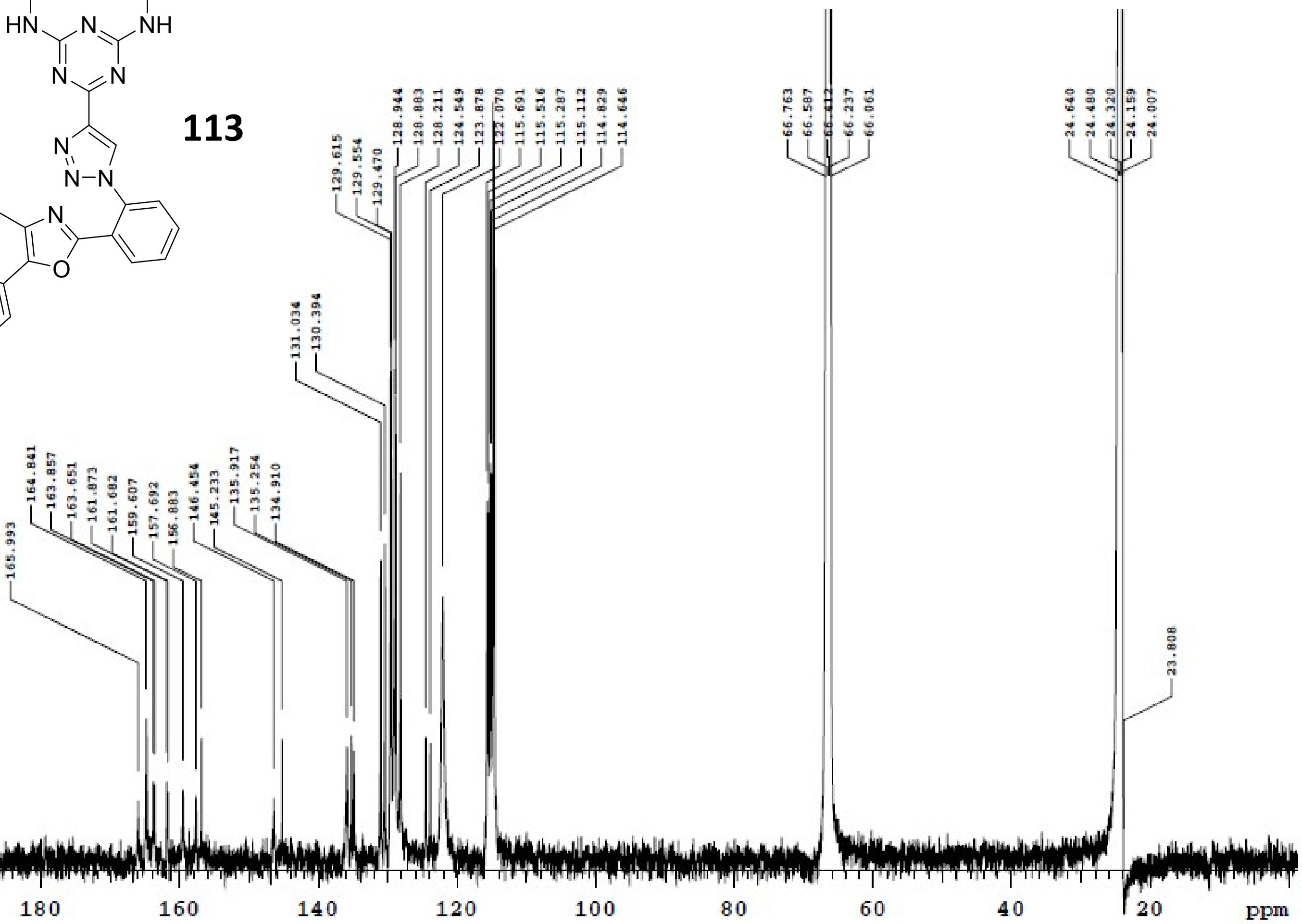

^{1}H NMR: 6-(1-(3-(4,5-bis(4-fluorophenyl)oxazol-2-yl)phenyl)-1H-1,2,3-triazol-4-yl)-N2,N4-bis(4-fluorophenyl)-1,3,5-triazine-2,4-diamine

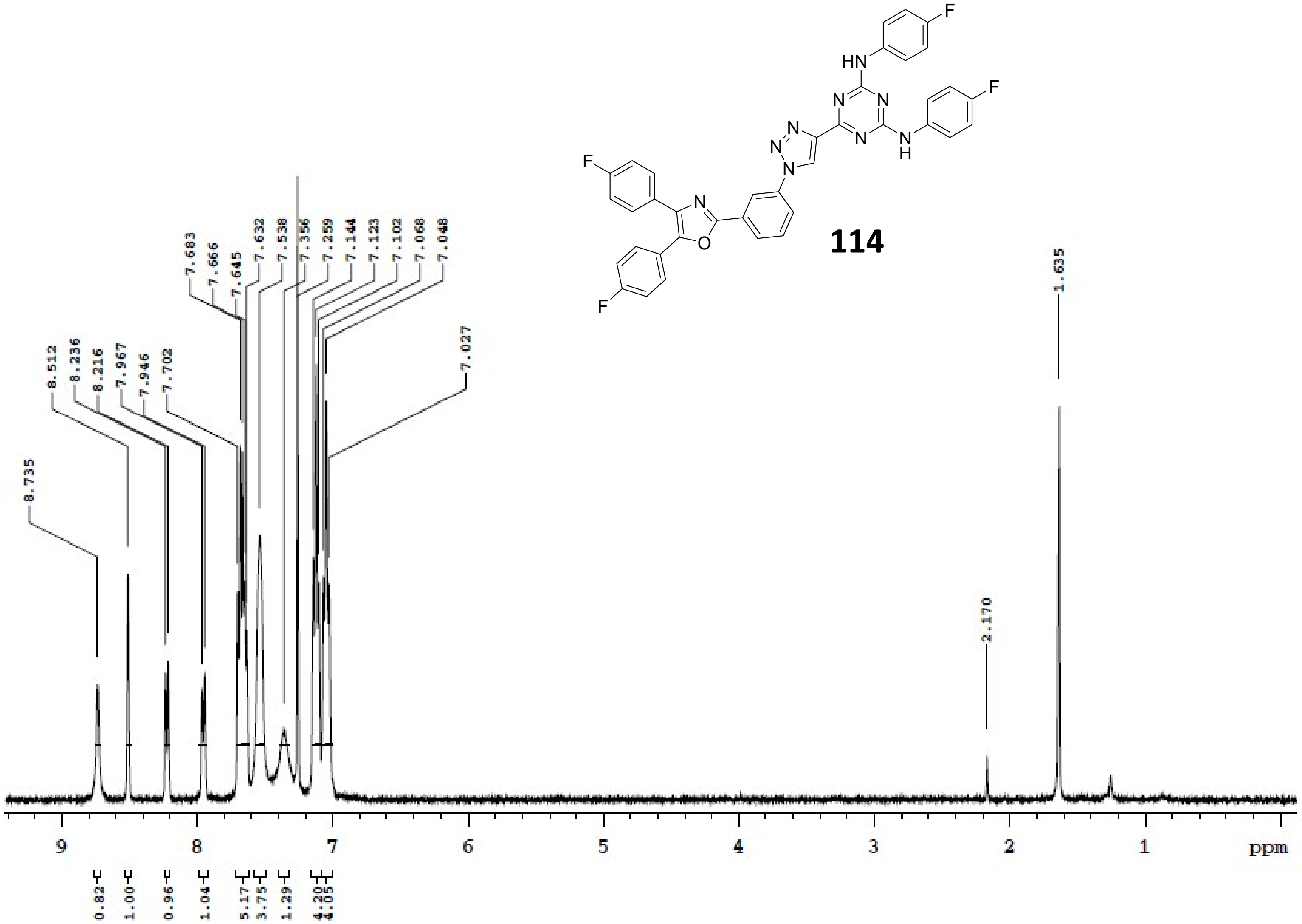

^{13}C NMR: 6-(1-(3-(4,5-bis(4-fluorophenyl)oxazol-2-yl)phenyl)-1H-1,2,3-triazol-4-yl)-N2,N4-bis(4-fluorophenyl)-1,3,5-triazine-2,4-diamine

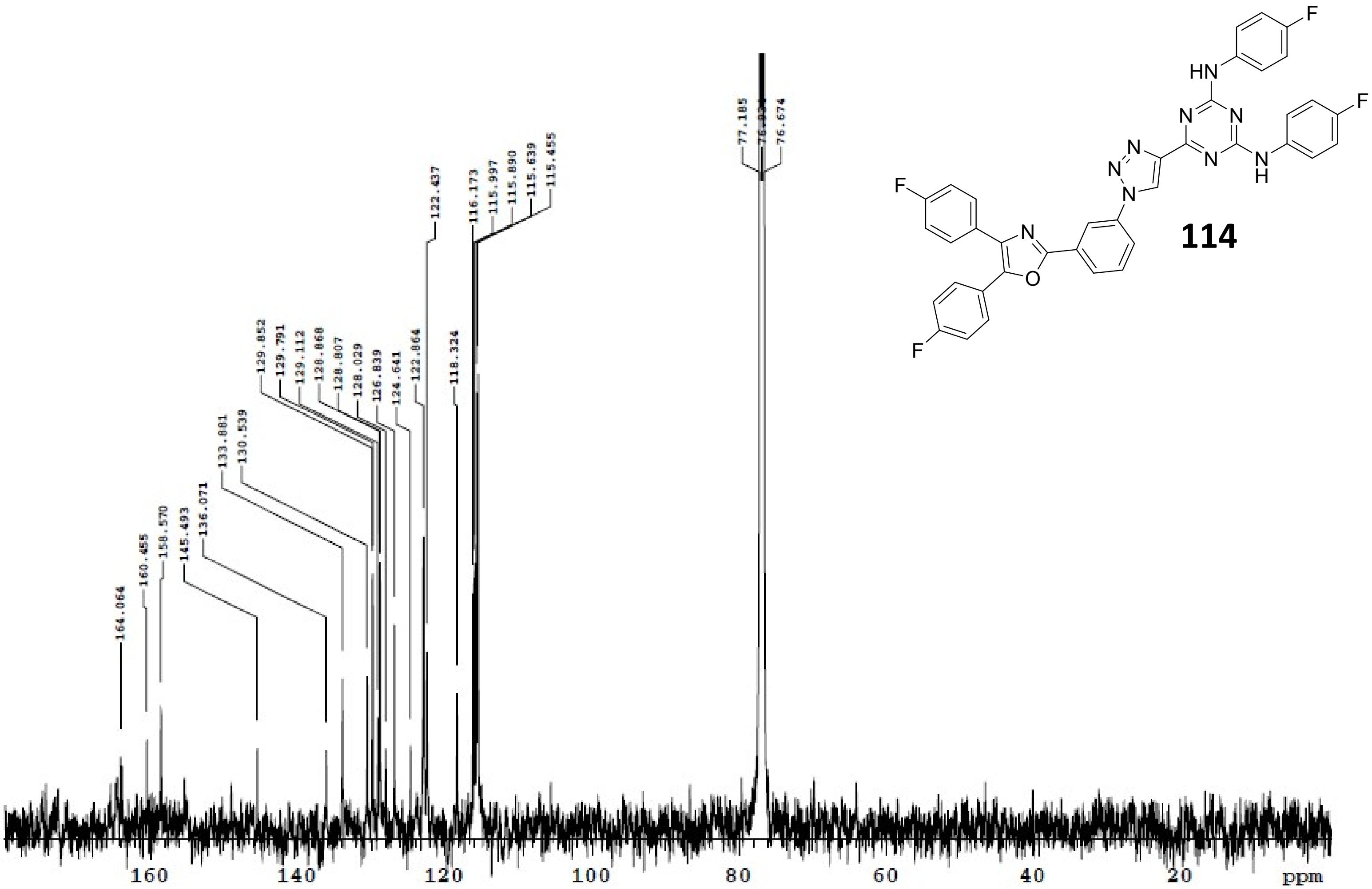

114

^{1}H NMR: 6-(1-(4-(4,5-bis(4-fluorophenyl)oxazol-2-yl)phenyl)-1H-1,2,3-triazol-4-yl)-N2,N4-bis(4-fluorophenyl)-1,3,5-triazine-2,4-diamine

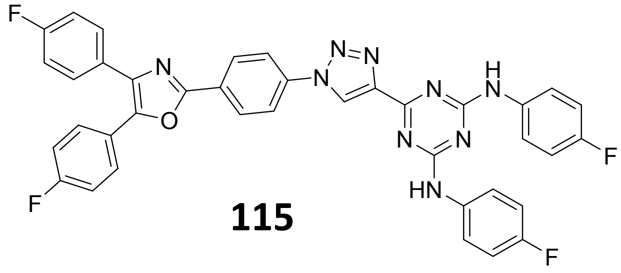

115

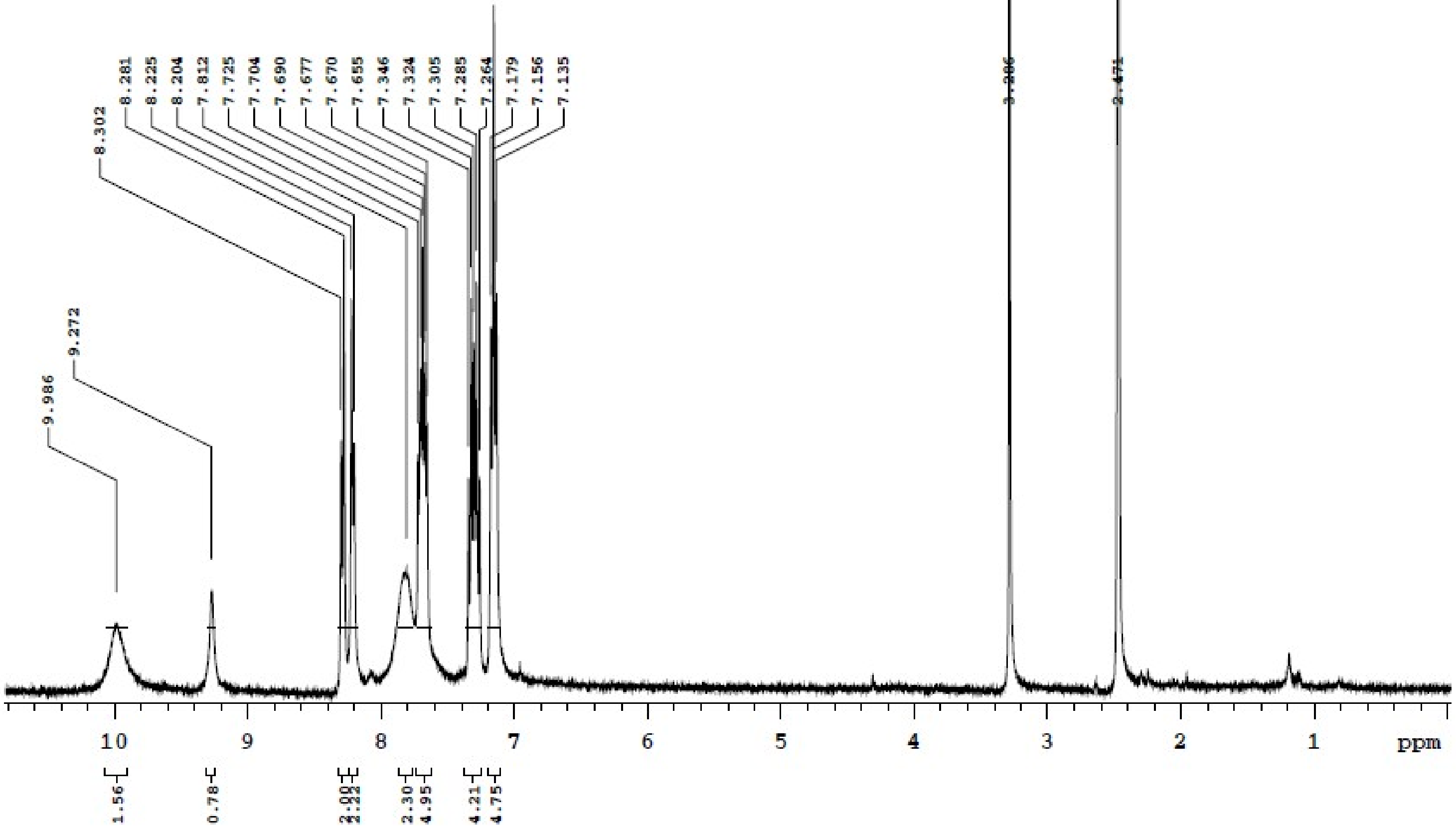

^{13}C NMR: 6-(1-(4-(4,5-bis(4-fluorophenyl)oxazol-2-yl)phenyl)-1H-1,2,3-triazol-4-yl)-N2,N4-bis(4-fluorophenyl)-1,3,5-triazine-2,4-diamine

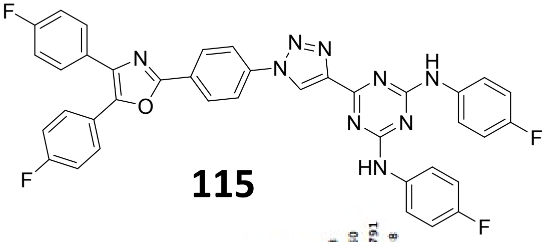

115

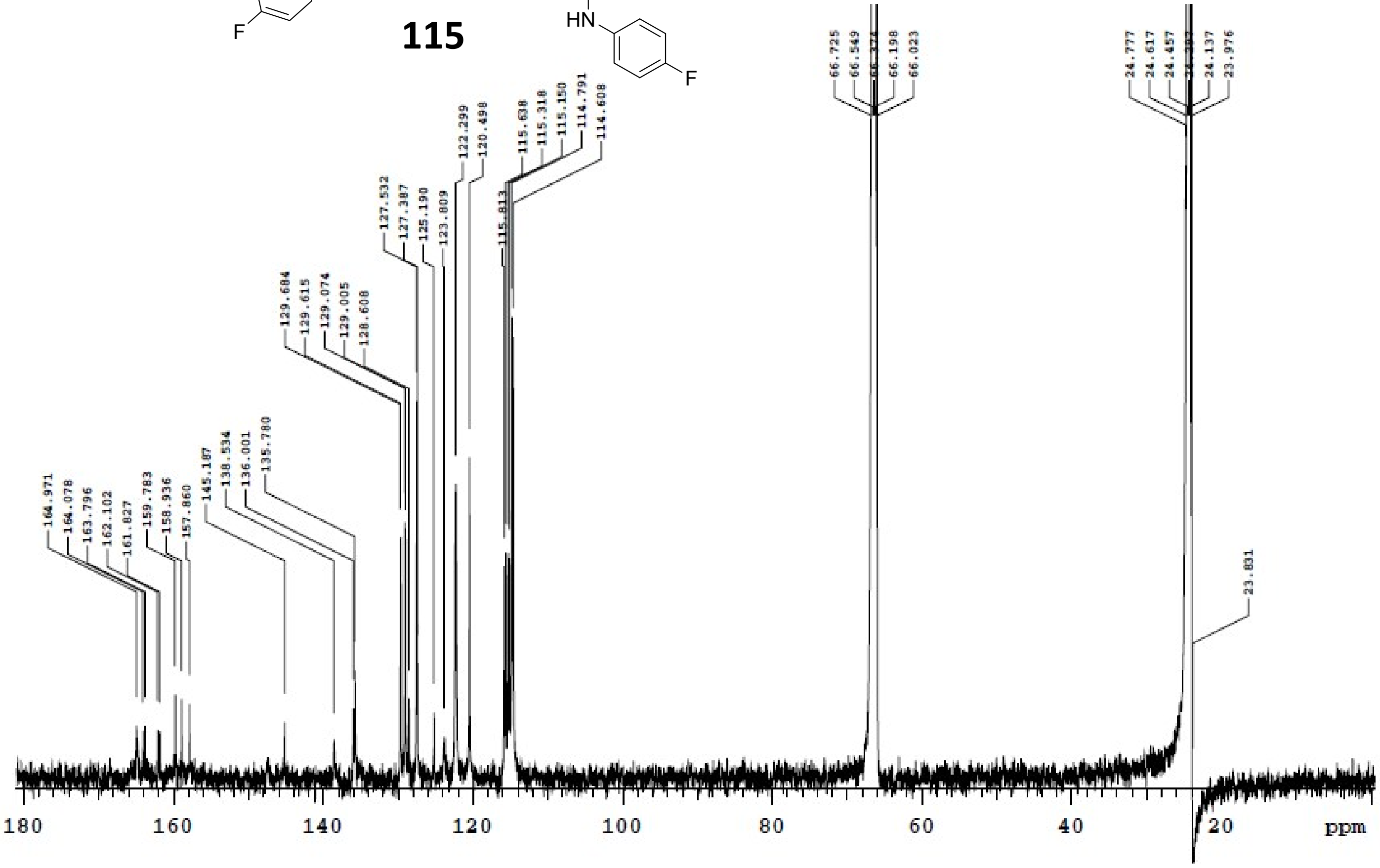

^{1}H NMR: 6-(1-(2-(4,5-bis(4-chlorophenyl)oxazol-2-yl)phenyl)-1H-1,2,3-triazol-4-yl)-N2,N4-bis(4-fluorophenyl)-1,3,5-triazine-2,4-diamine

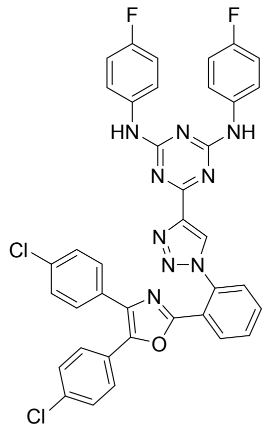

116

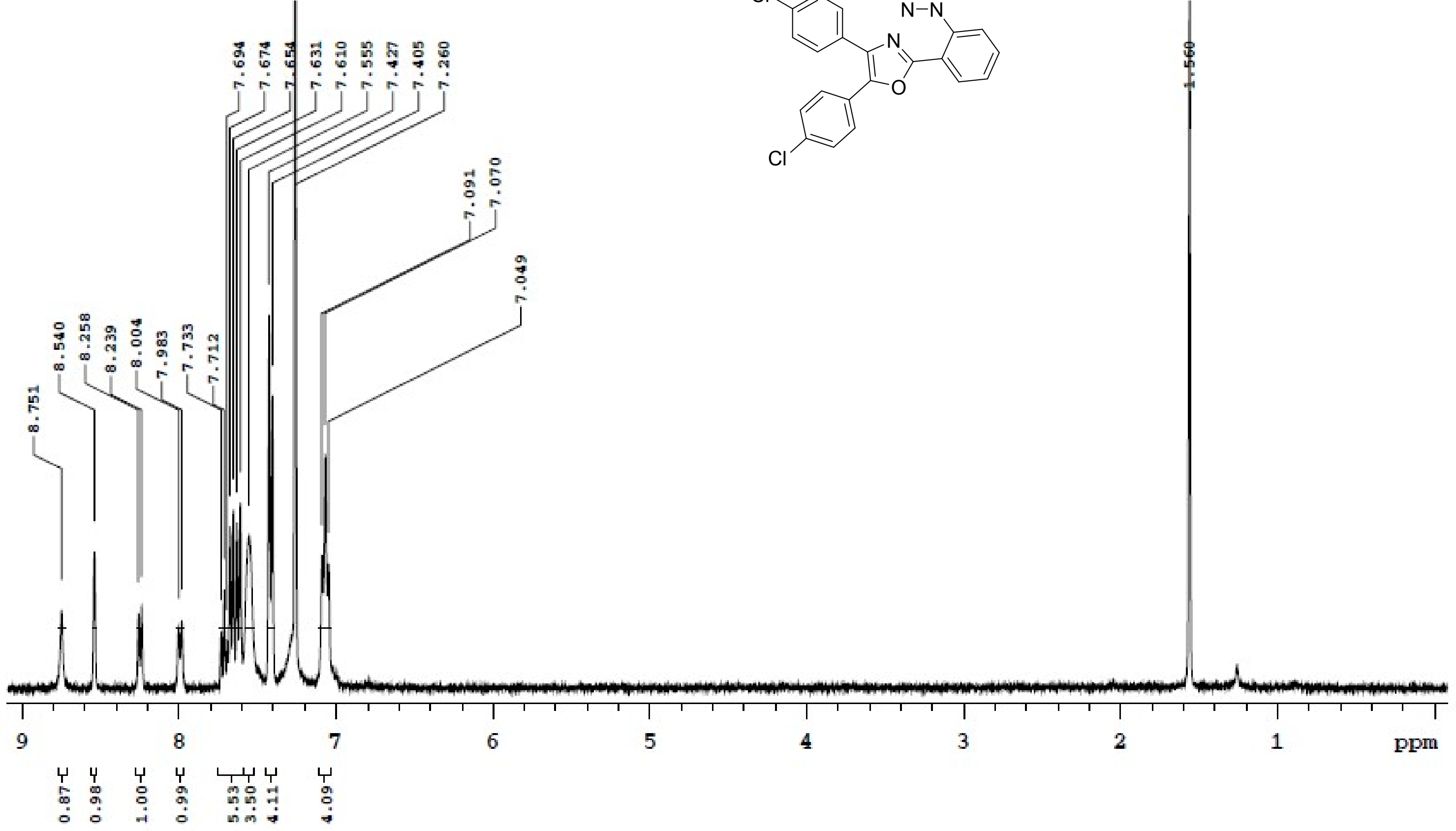

^{13}C NMR: 6-(1-(2-(4,5-bis(4-chlorophenyl)oxazol-2-yl)phenyl)-1H-1,2,3-triazol-4-yl)-N2,N4-bis(4-fluorophenyl)-1,3,5-triazine-2,4-diamine

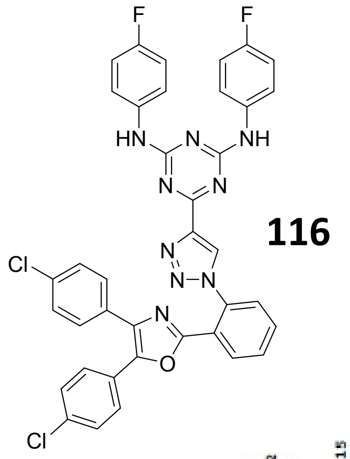

116

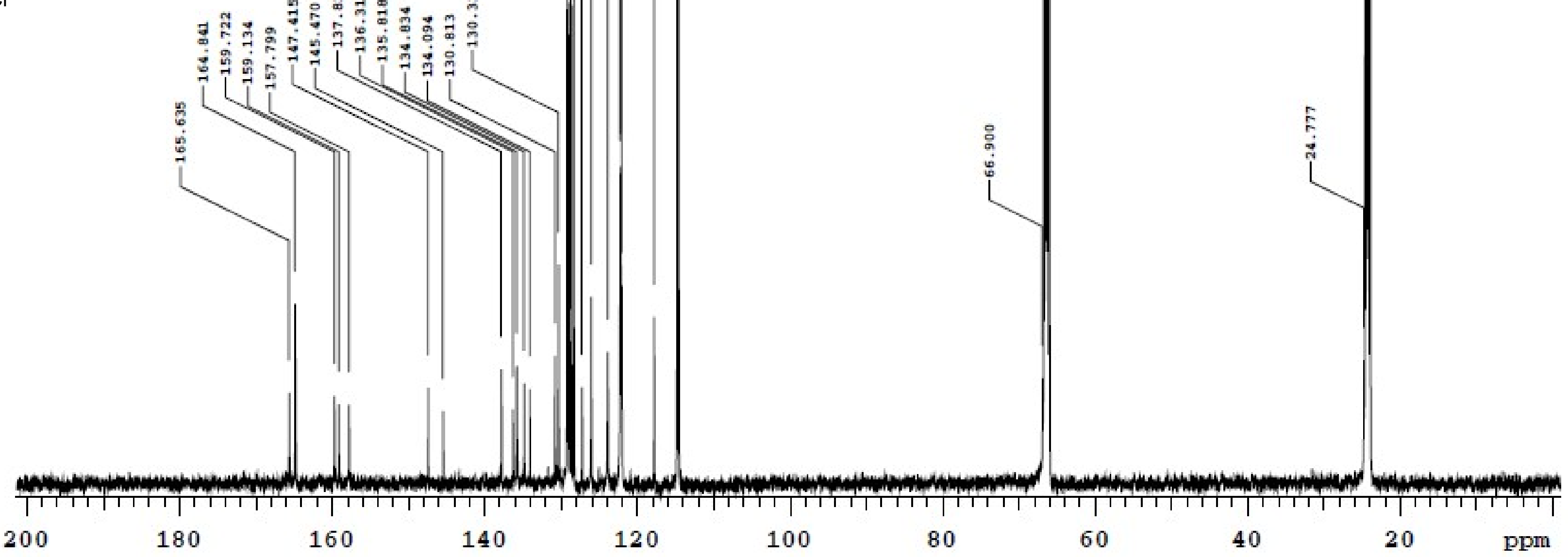

^{1}H NMR: 6-(1-(3-(4,5-bis(4-chlorophenyl)oxazol-2-yl)phenyl)-1H-1,2,3-triazol-4-yl)-N2,N4-bis(4-fluorophenyl)-1,3,5-triazine-2,4-diamine

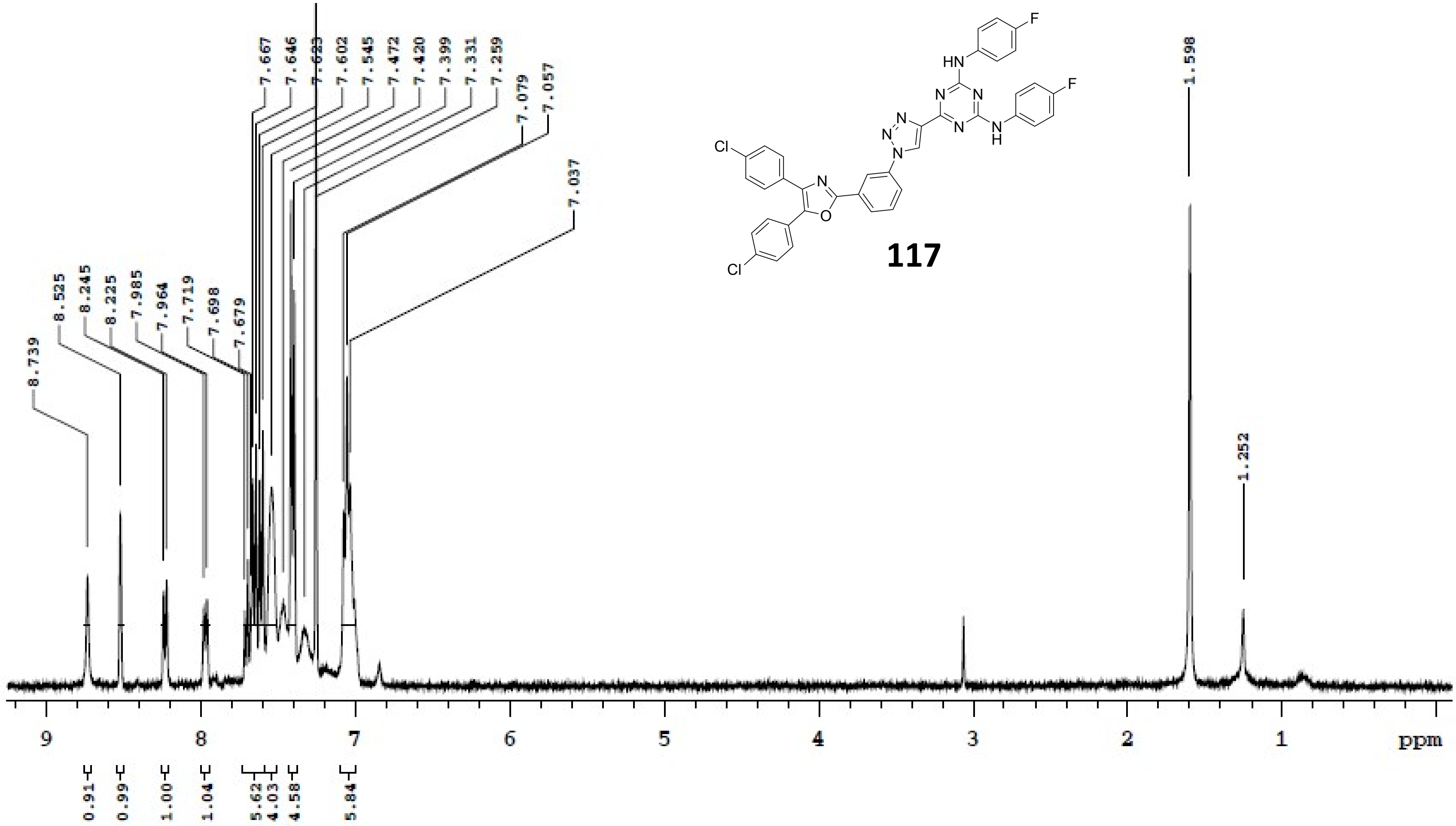

^{13}C NMR: 6-(1-(3-(4,5-bis(4-chlorophenyl)oxazol-2-yl)phenyl)-1H-1,2,3-triazol-4-yl)-N2,N4-bis(4-fluorophenyl)-1,3,5-triazine-2,4-diamine

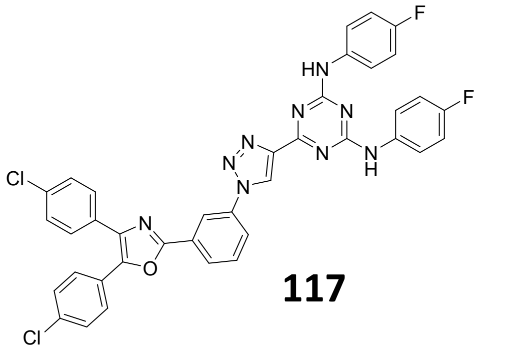

117

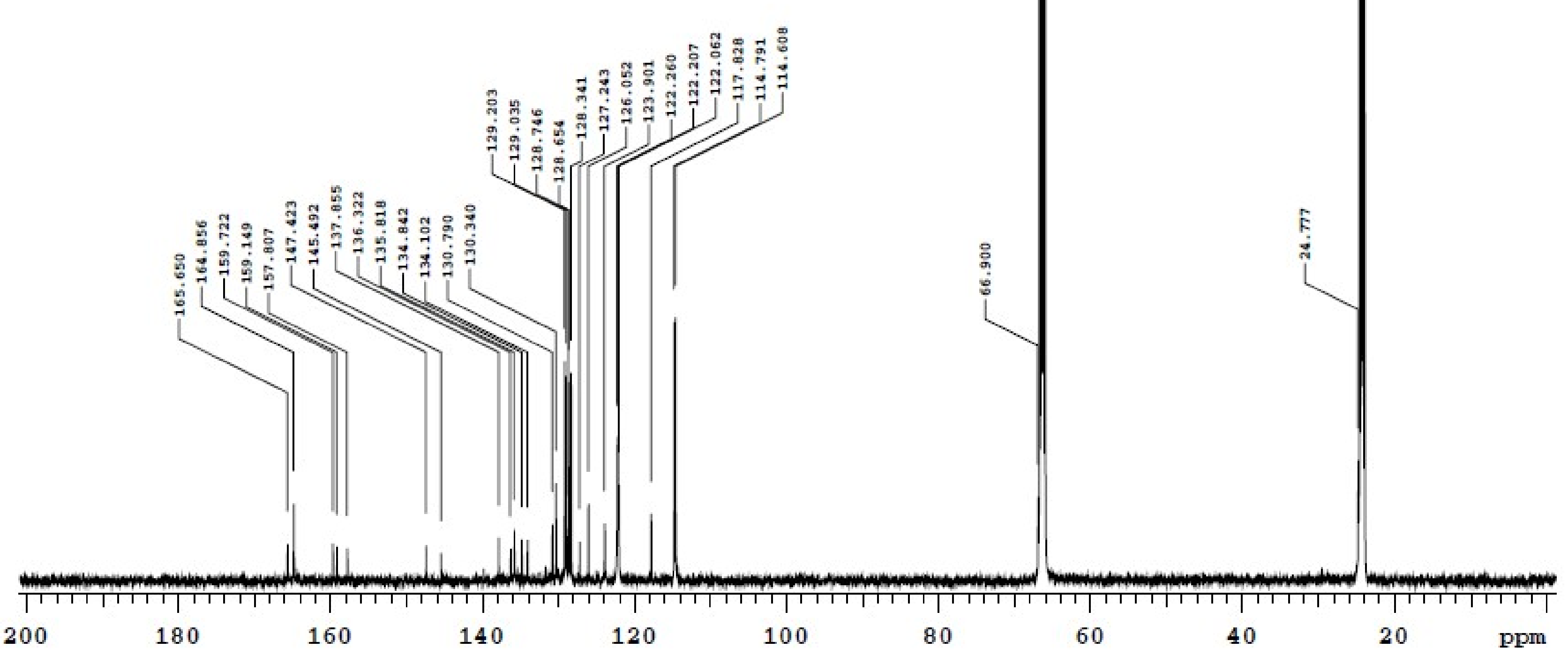

^{1}H NMR: 6-(1-(4-(4,5-bis(4-chlorophenyl)oxazol-2-yl)phenyl)-1H-1,2,3-triazol-4-yl)-N2,N4-bis(4-fluorophenyl)-1,3,5-triazine-2,4-diamine

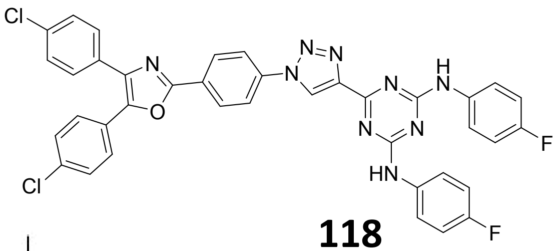

118

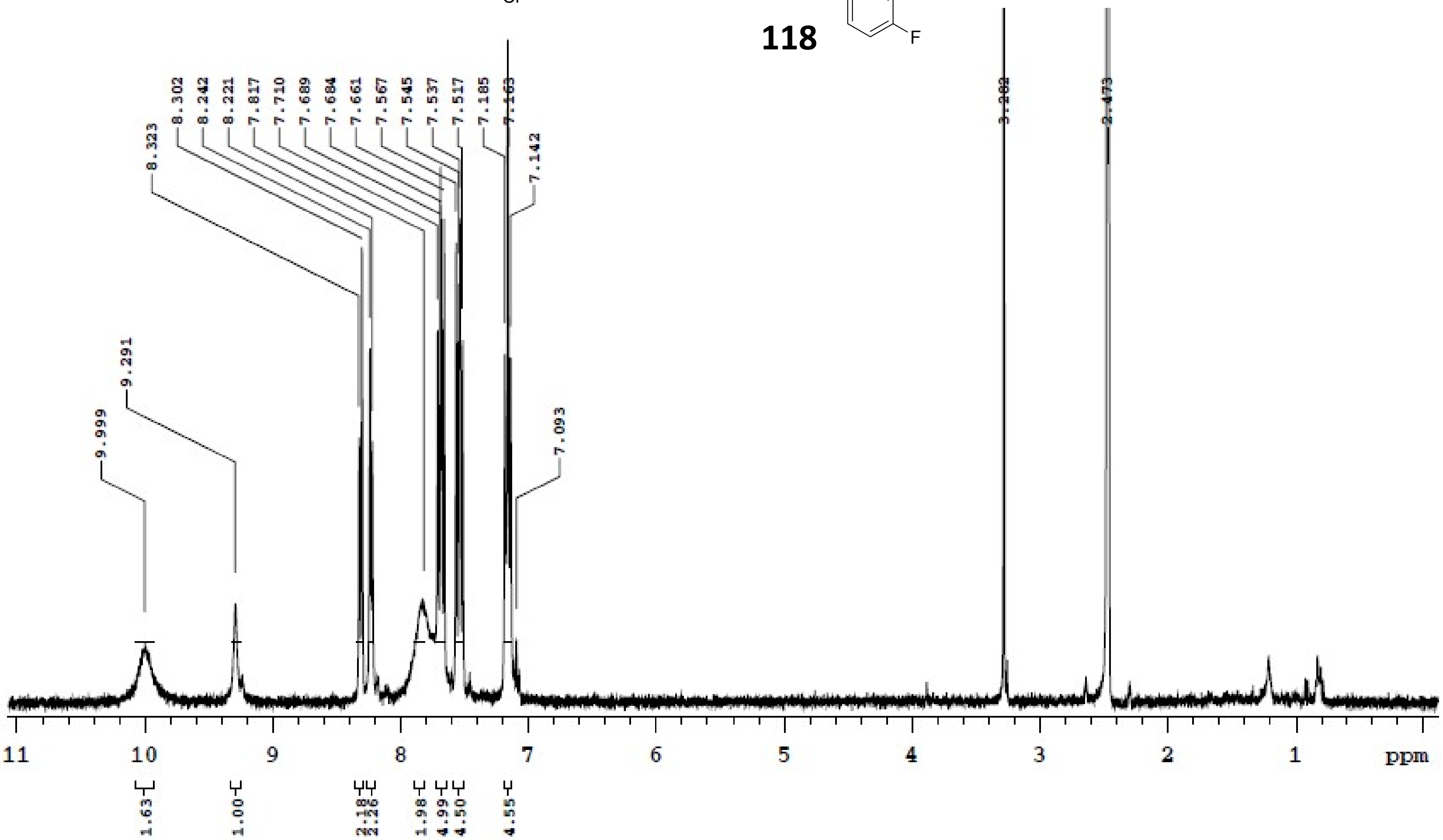

^{13}C NMR: 6-(1-(4-(4,5-bis(4-chlorophenyl)oxazol-2-yl)phenyl)-1H-1,2,3-triazol-4-yl)-N2,N4-bis(4-fluorophenyl)-1,3,5-triazine-2,4-diamine

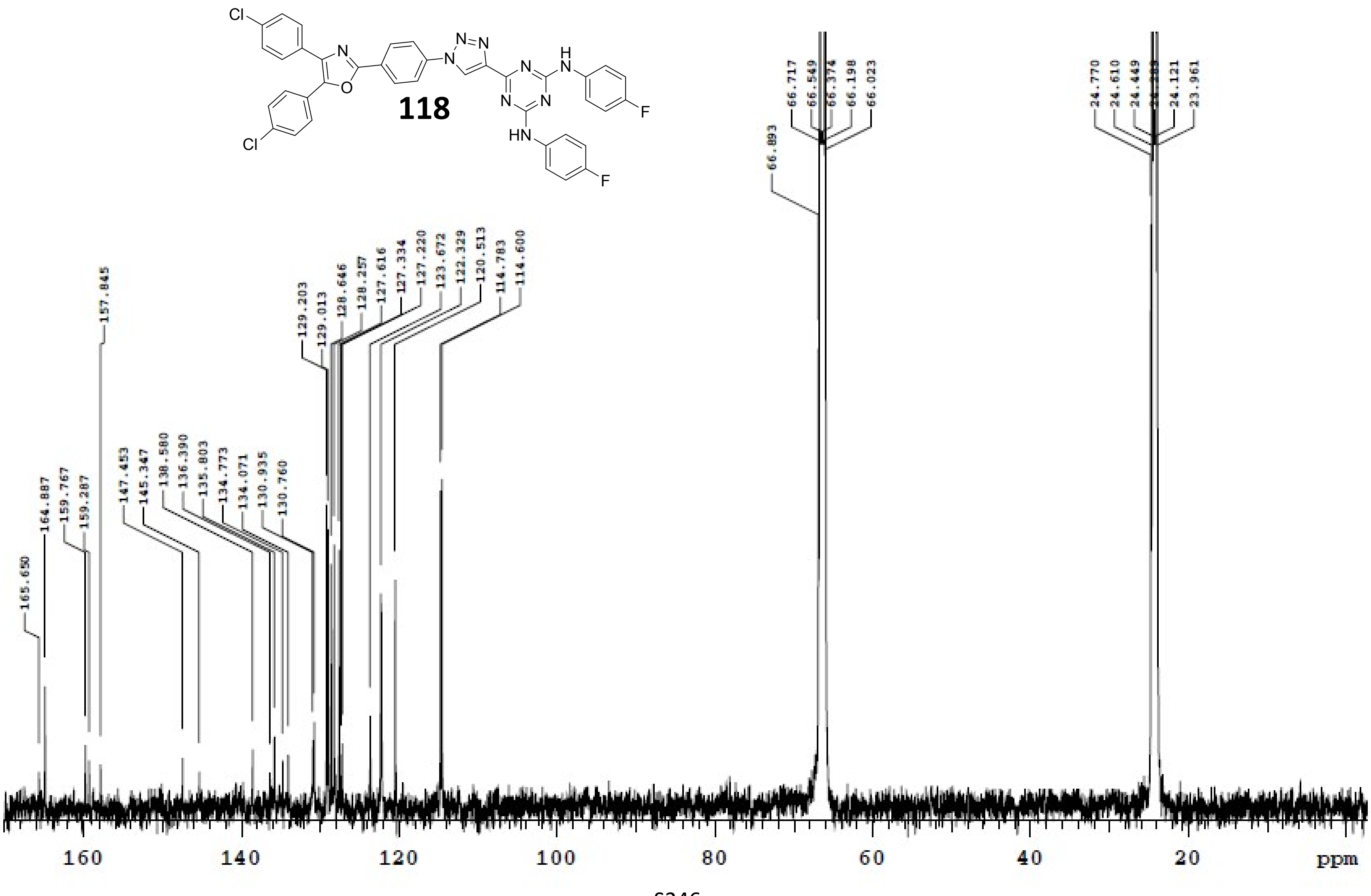

^{1}H NMR: 6-(1-(2-(4,5-bis(4-methoxyphenyl)oxazol-2-yl)phenyl)-1H-1,2,3-triazol-4-yl)-N2,N4-bis(4-fluorophenyl)-1,3,5-triazine-2,4-diamine

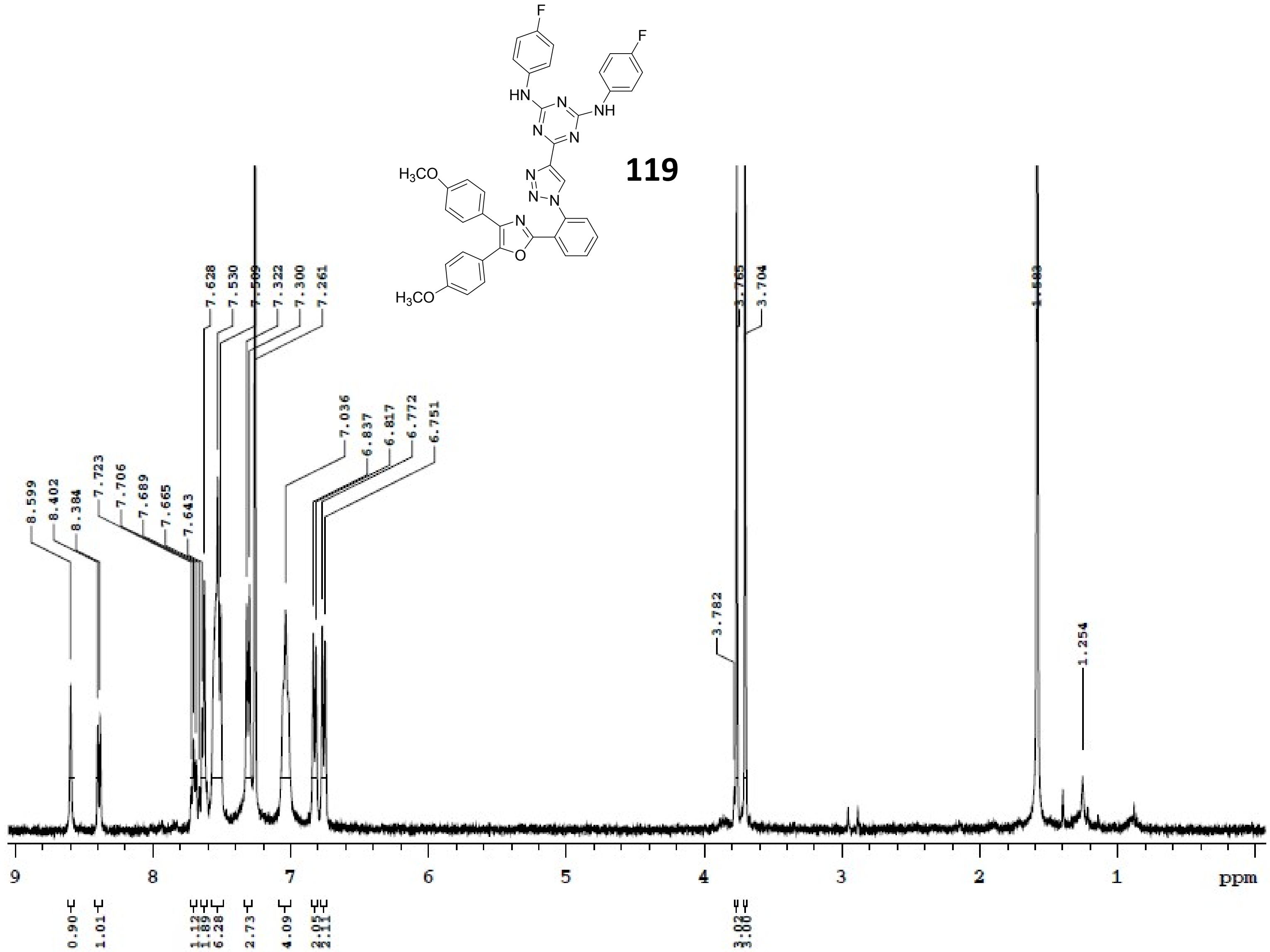

^{13}C NMR: 6-(1-(2-(4,5-bis(4-methoxyphenyl)oxazol-2-yl)phenyl)-1H-1,2,3-triazol-4-yl)-N2,N4-bis(4-fluorophenyl)-1,3,5-triazine-2,4-diamine

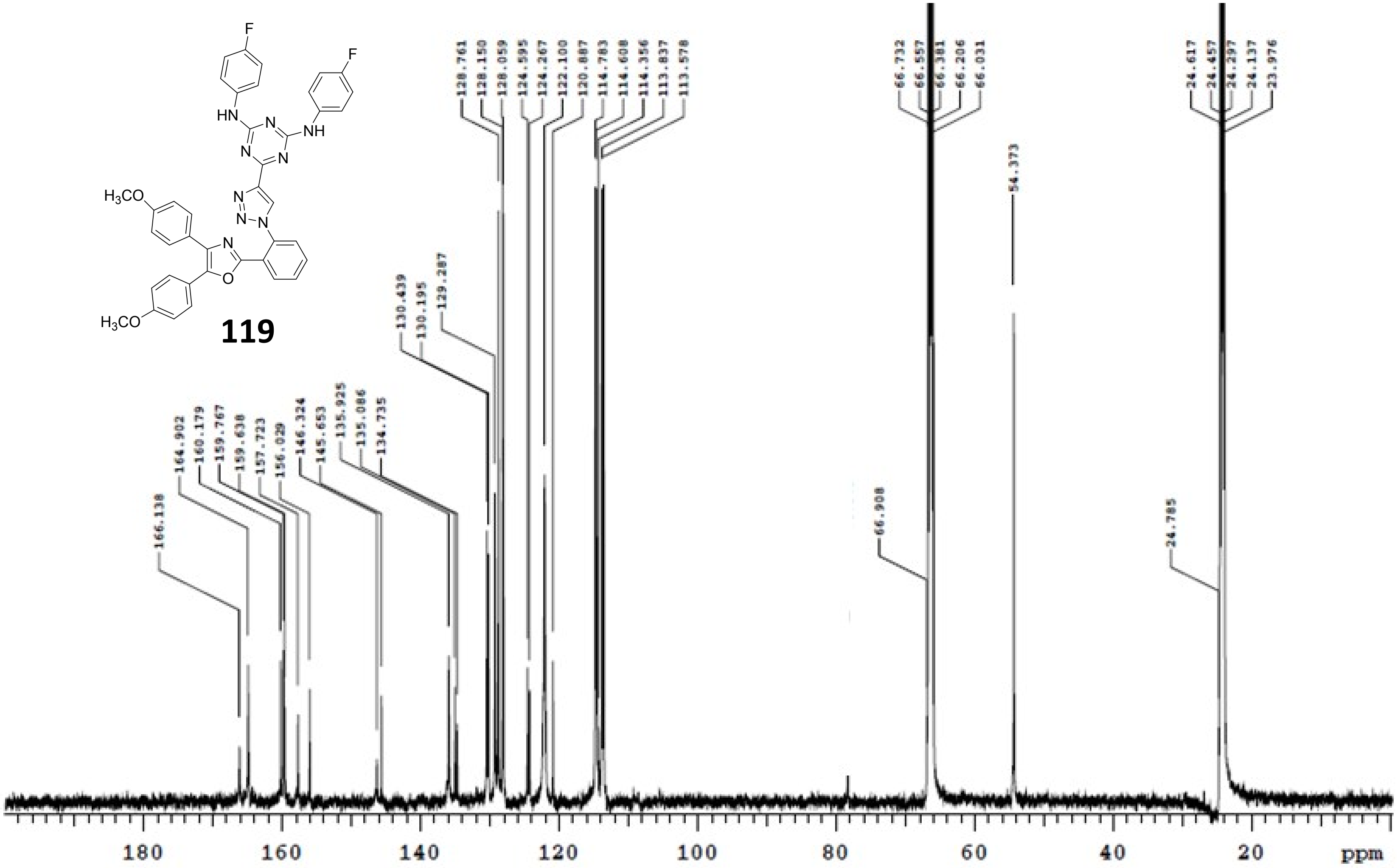

119

^{1}H NMR: 6-(1-(3-(4,5-bis(4-methoxyphenyl)oxazol-2-yl)phenyl)-1H-1,2,3-triazol-4-yl)-N2,N4-bis(4-fluorophenyl)-1,3,5-triazine-2,4-diamine

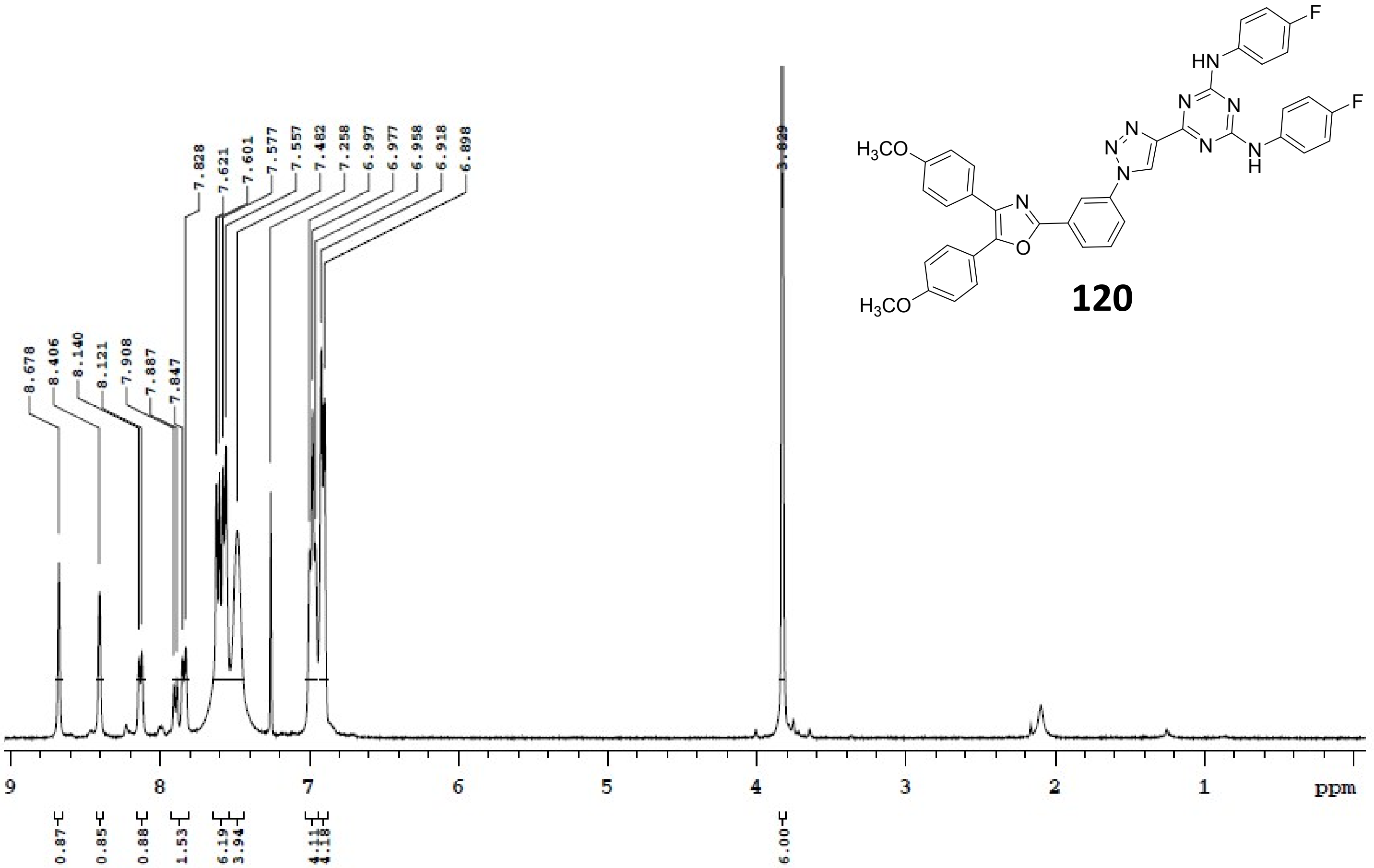

^{13}C NMR: 6-(1-(3-(4,5-bis(4-methoxyphenyl)oxazol-2-yl)phenyl)-1H-1,2,3-triazol-4-yl)-N2,N4-bis(4-fluorophenyl)-1,3,5-triazine-2,4-diamine

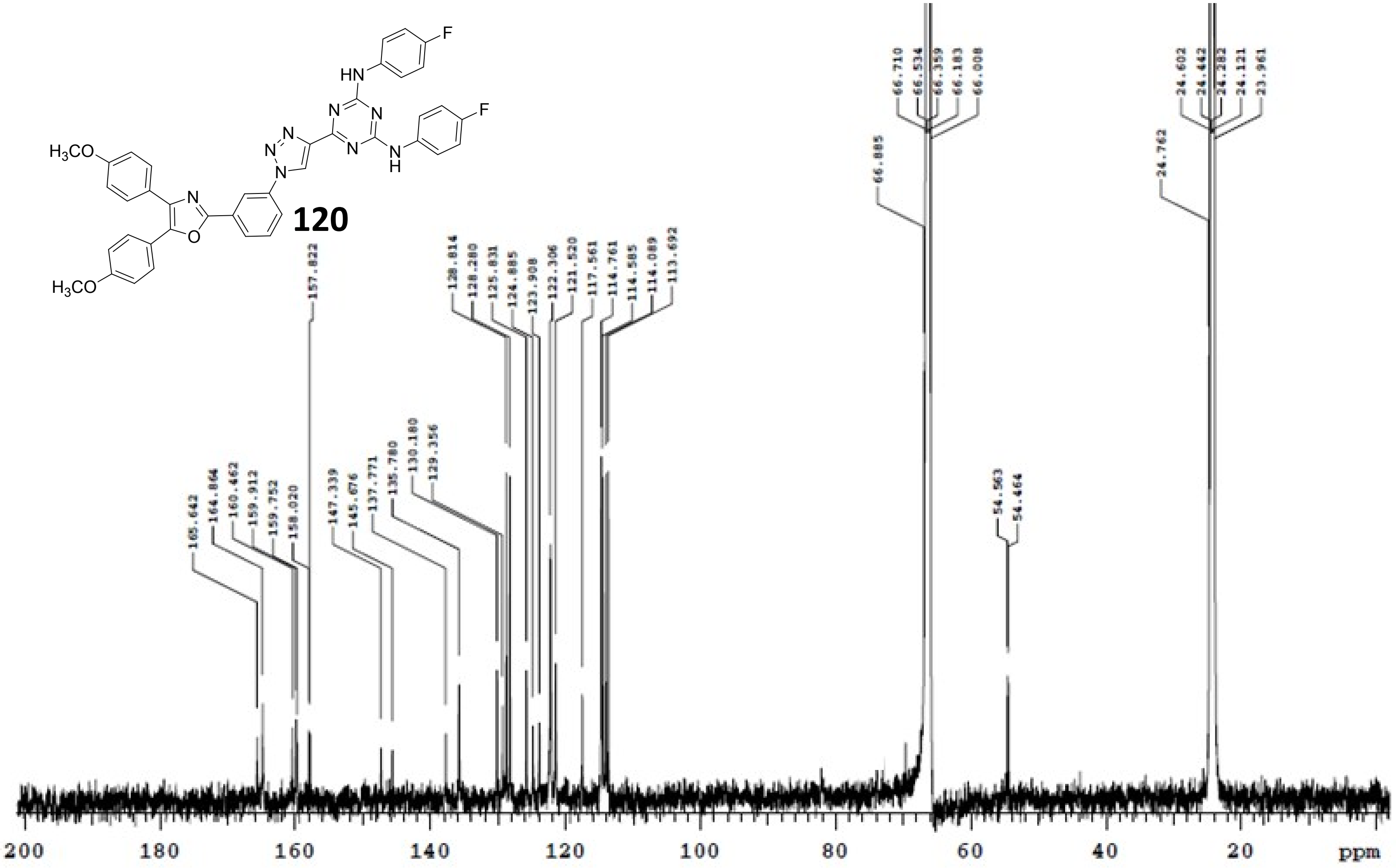

^{1}H NMR: 6-(1-(4-(4,5-bis(4-methoxyphenyl)oxazol-2-yl)phenyl)-1H-1,2,3-triazol-4-yl)-N2,N4-bis(4-fluorophenyl)-1,3,5-triazine-2,4-diamine

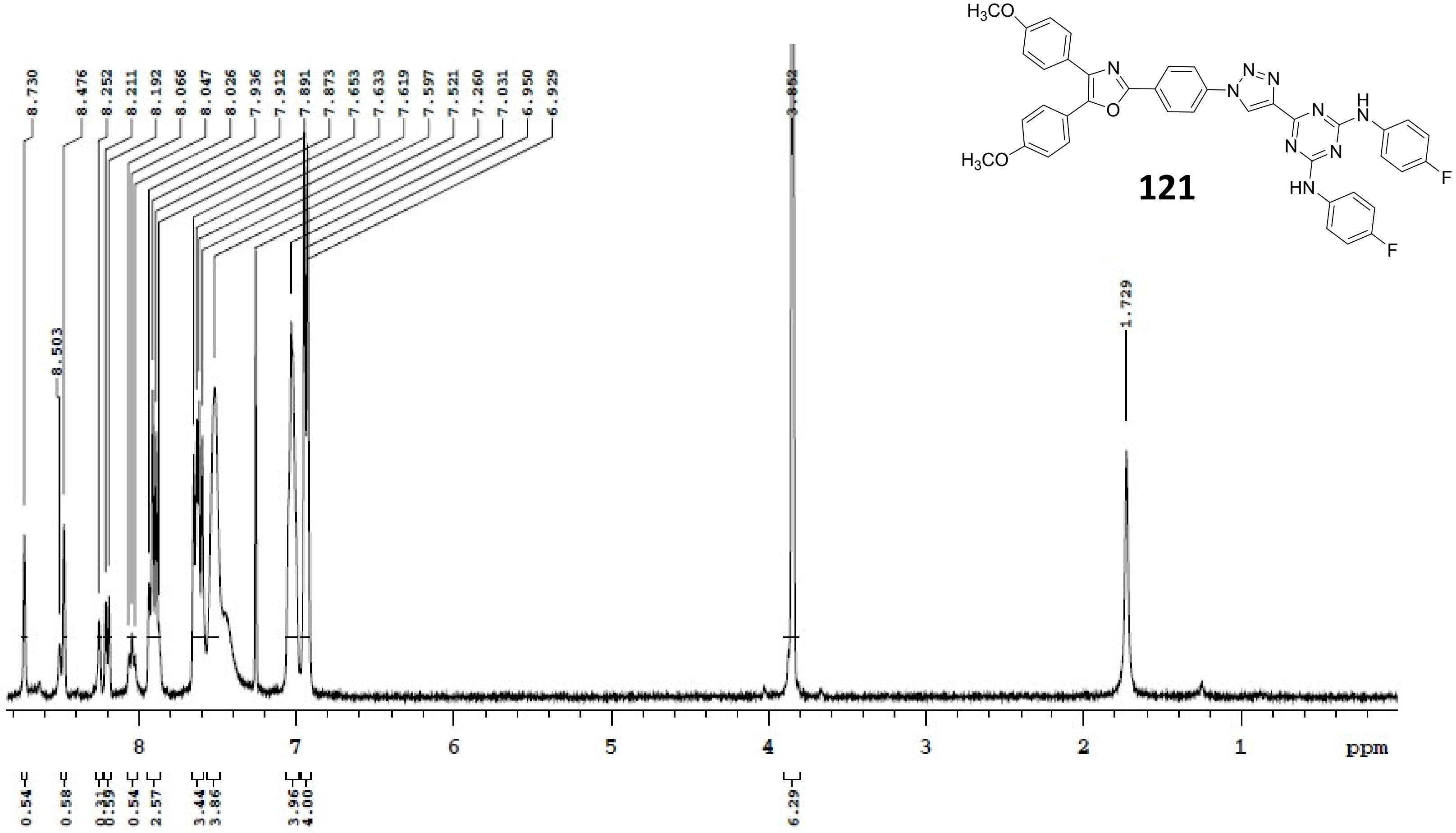

121

^{13}C NMR: 6-(1-(4-(4,5-bis(4-methoxyphenyl)oxazol-2-yl)phenyl)-1H-1,2,3-triazol-4-yl)-N2,N4-bis(4-fluorophenyl)-1,3,5-triazine-2,4-diamine

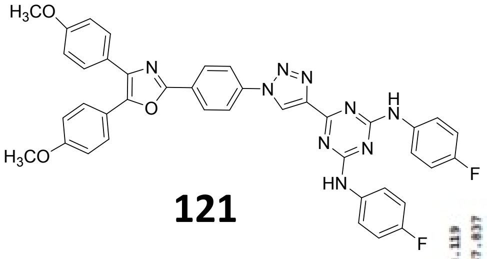

121

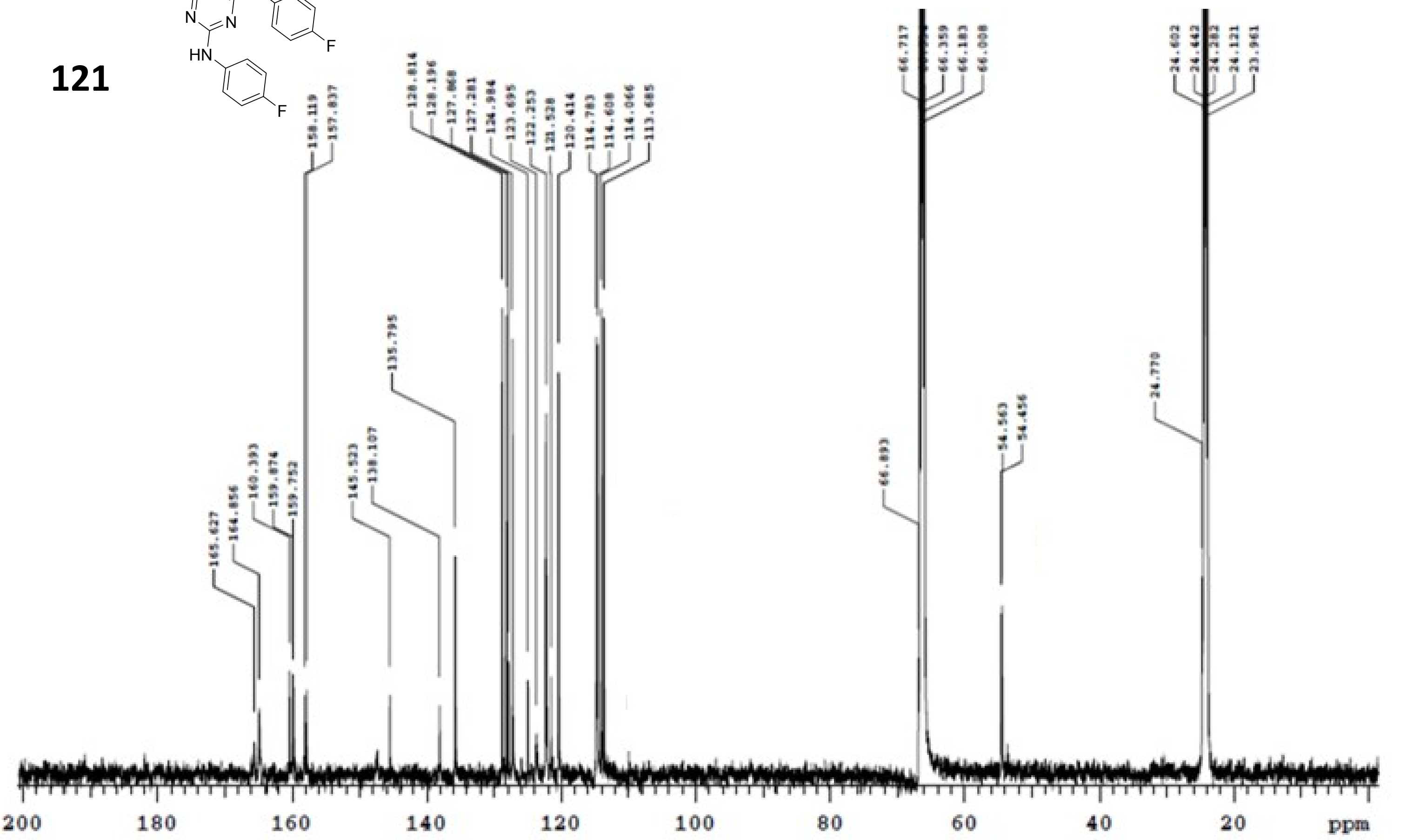

^{1}H NMR: 6-(1-(2-(4,5-di(furan-2-yl)oxazol-2-yl)phenyl)-1H-1,2,3-triazol-4-yl)-N2,N4-bis(4-fluorophenyl)-1,3,5-triazine-2,4-diamine

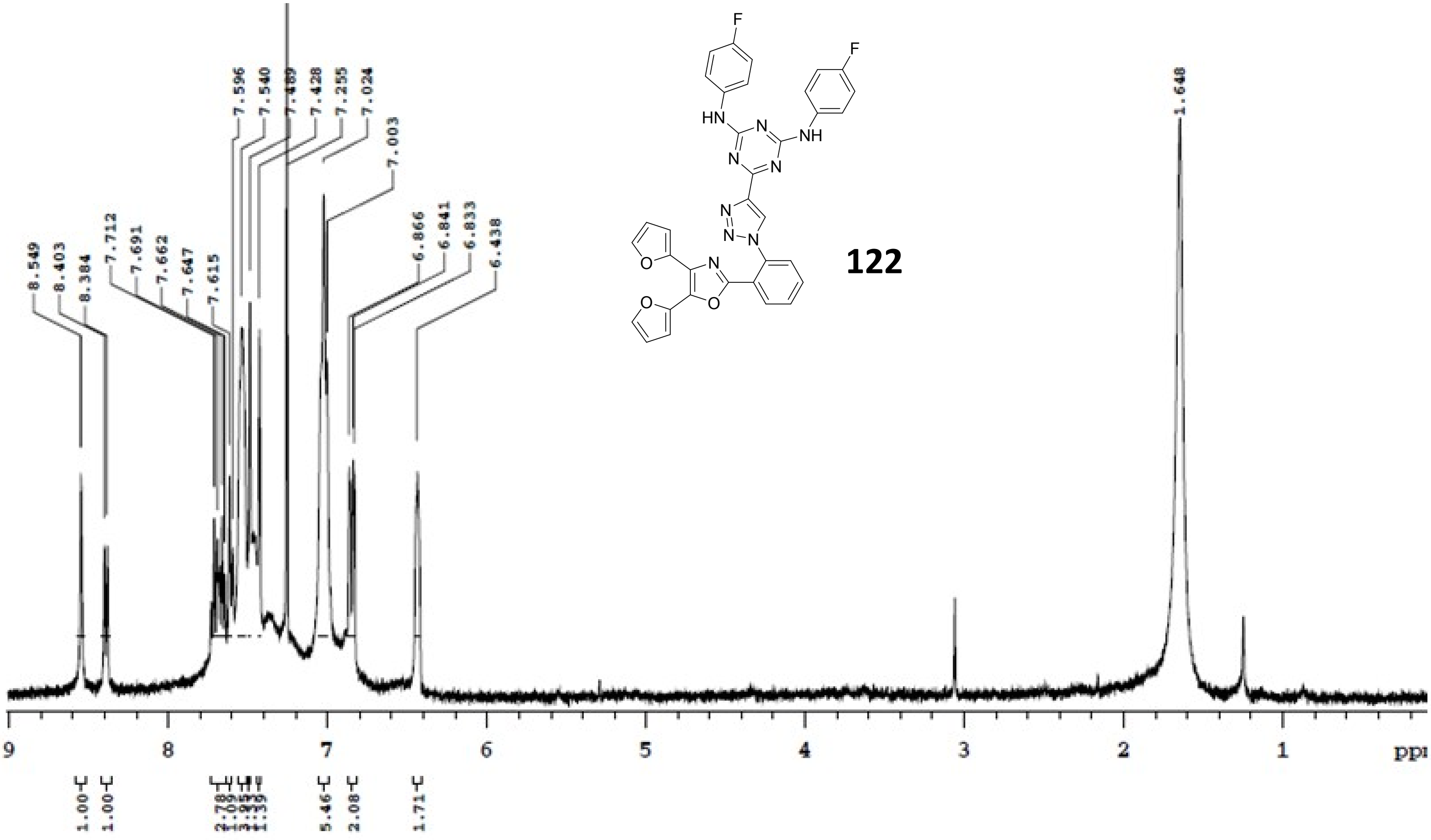

^{13}C NMR: 6-(1-(2-(4,5-di(furan-2-yl)oxazol-2-yl)phenyl)-1H-1,2,3-triazol-4-yl)-N2,N4-bis(4-fluorophenyl)-1,3,5-triazine-2,4-diamine

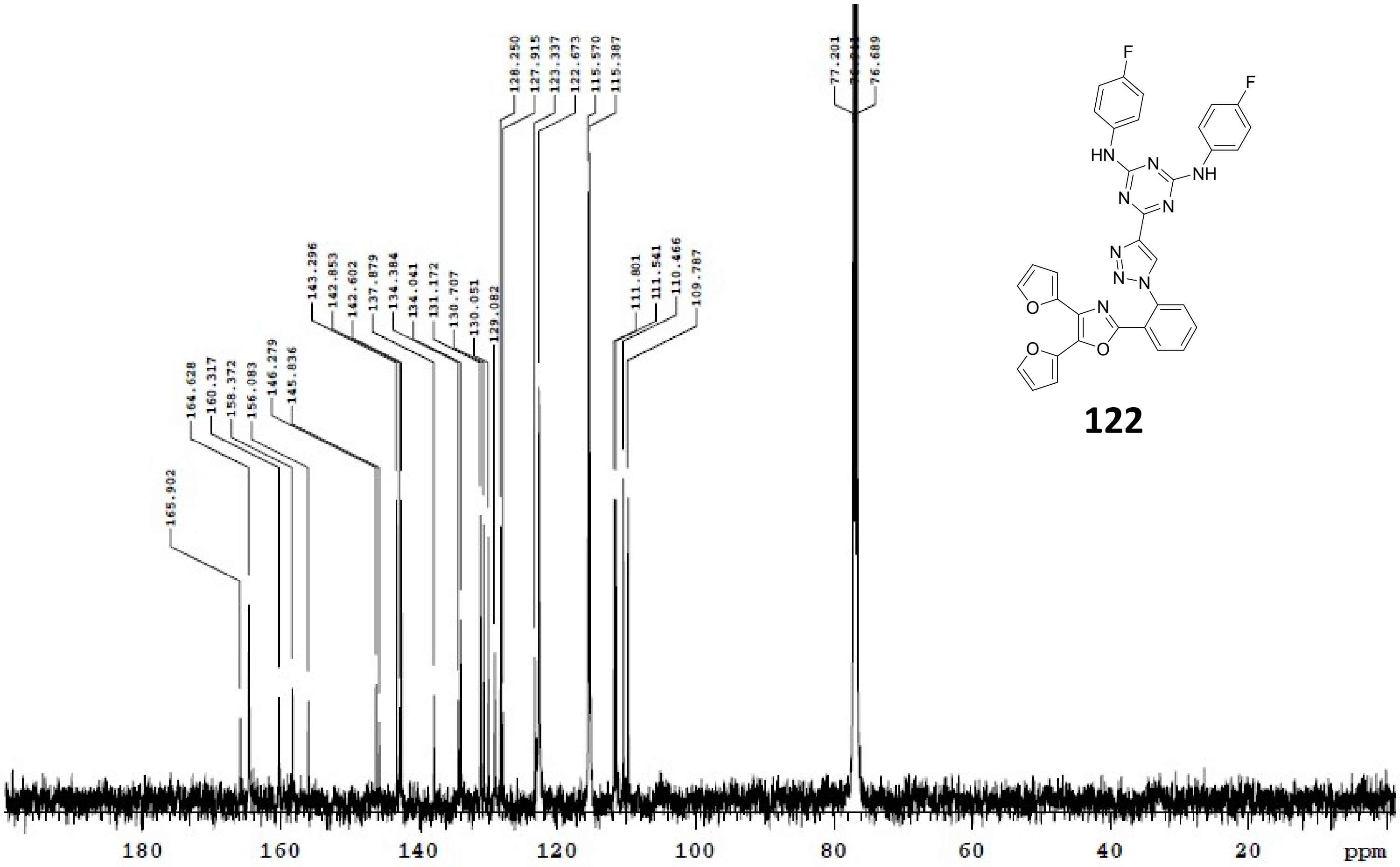

122

^{1}H NMR: 6-(1-(3-(4,5-di(furan-2-yl)oxazol-2-yl)phenyl)-1H-1,2,3-triazol-4-yl)-N2,N4-bis(4-fluorophenyl)-1,3,5-triazine-2,4-diamine

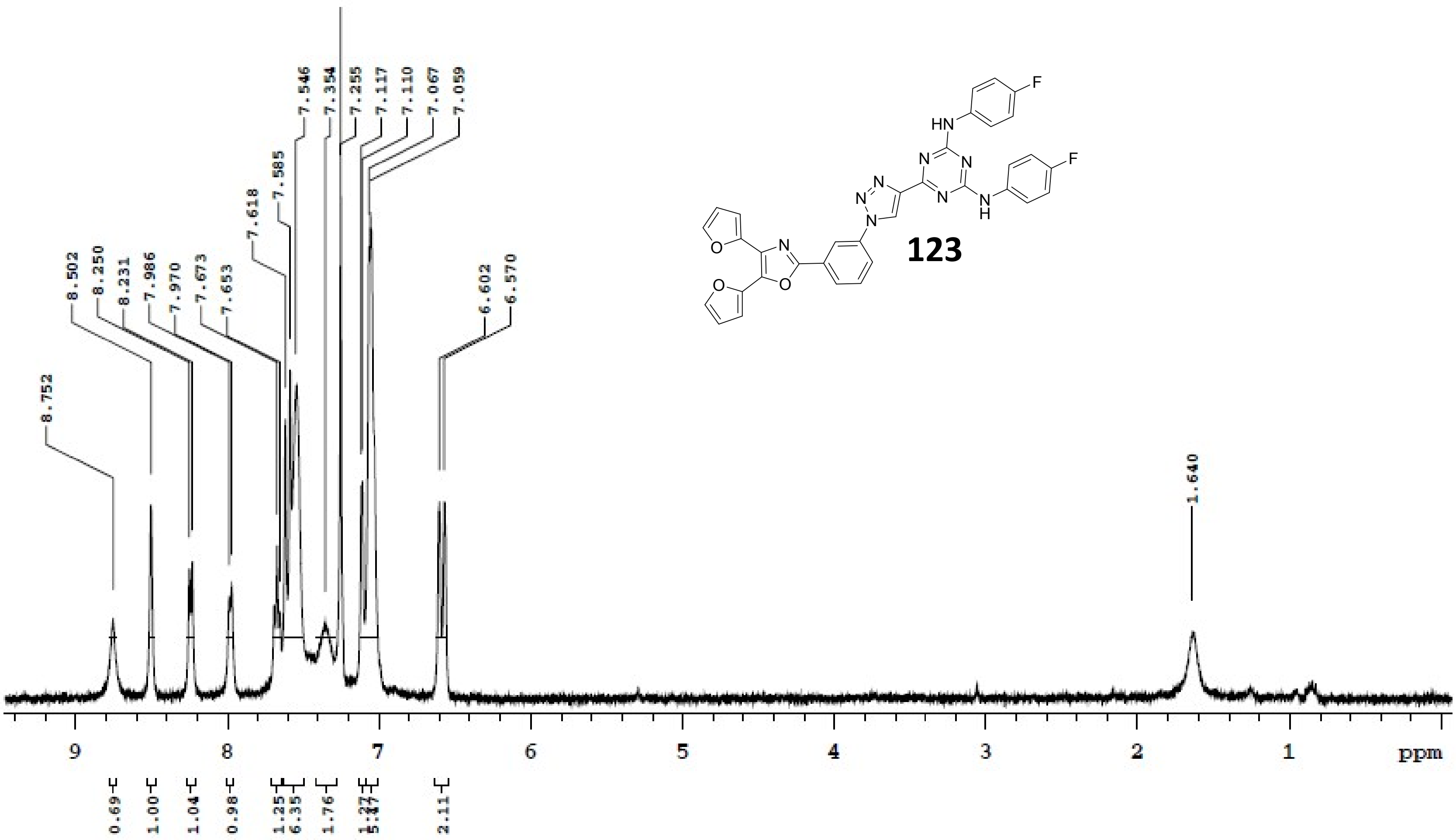

^{13}C NMR: 6-(1-(3-(4,5-di(furan-2-yl)oxazol-2-yl)phenyl)-1H-1,2,3-triazol-4-yl)-N2,N4-bis(4-fluorophenyl)-1,3,5-triazine-2,4-diamine

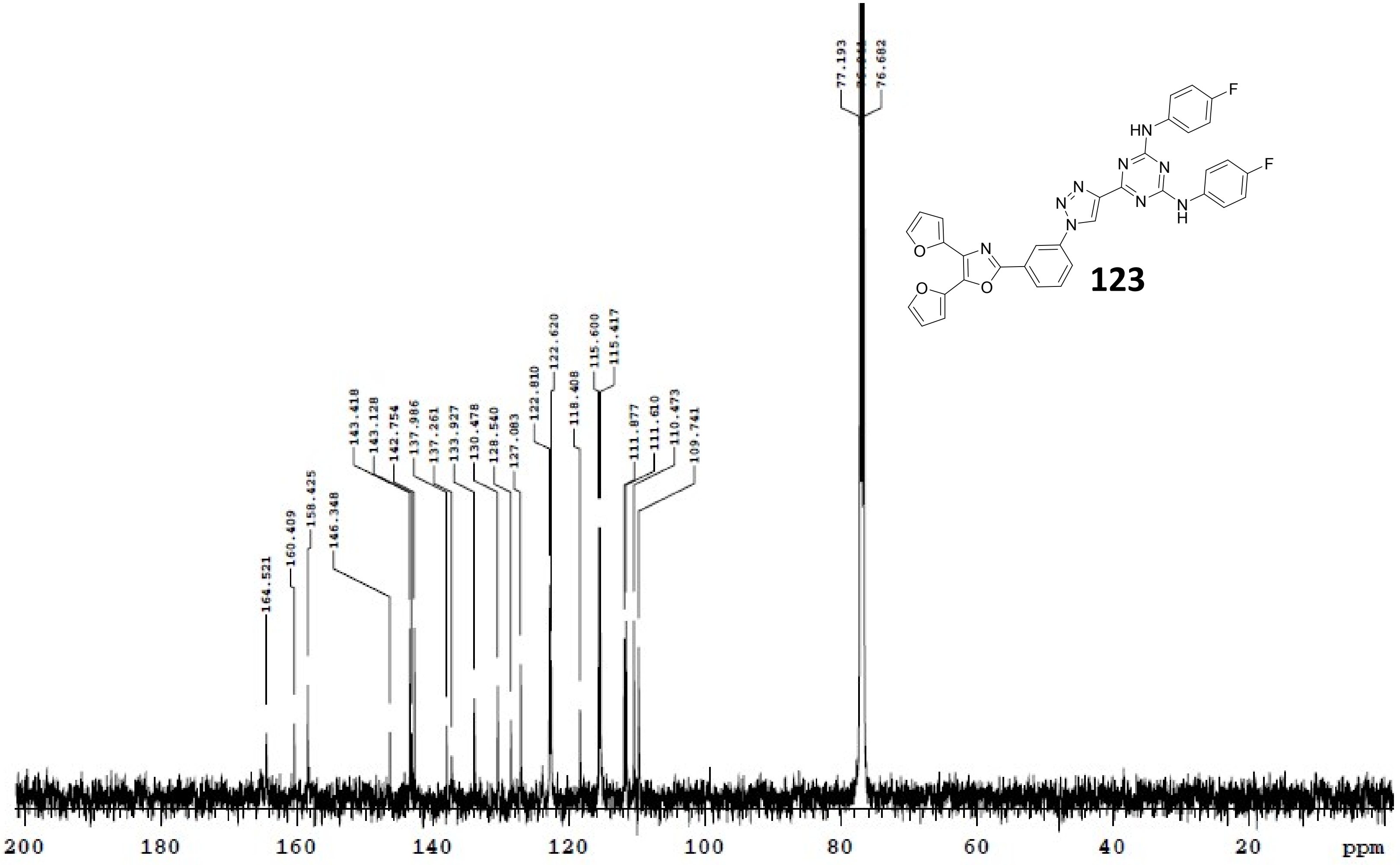

^{1}H NMR: 6-(1-(4-(4,5-di(furan-2-yl)oxazol-2-yl)phenyl)-1H-1,2,3-triazol-4-yl)-N2,N4-bis(4-fluorophenyl)-1,3,5-triazine-2,4-diamine

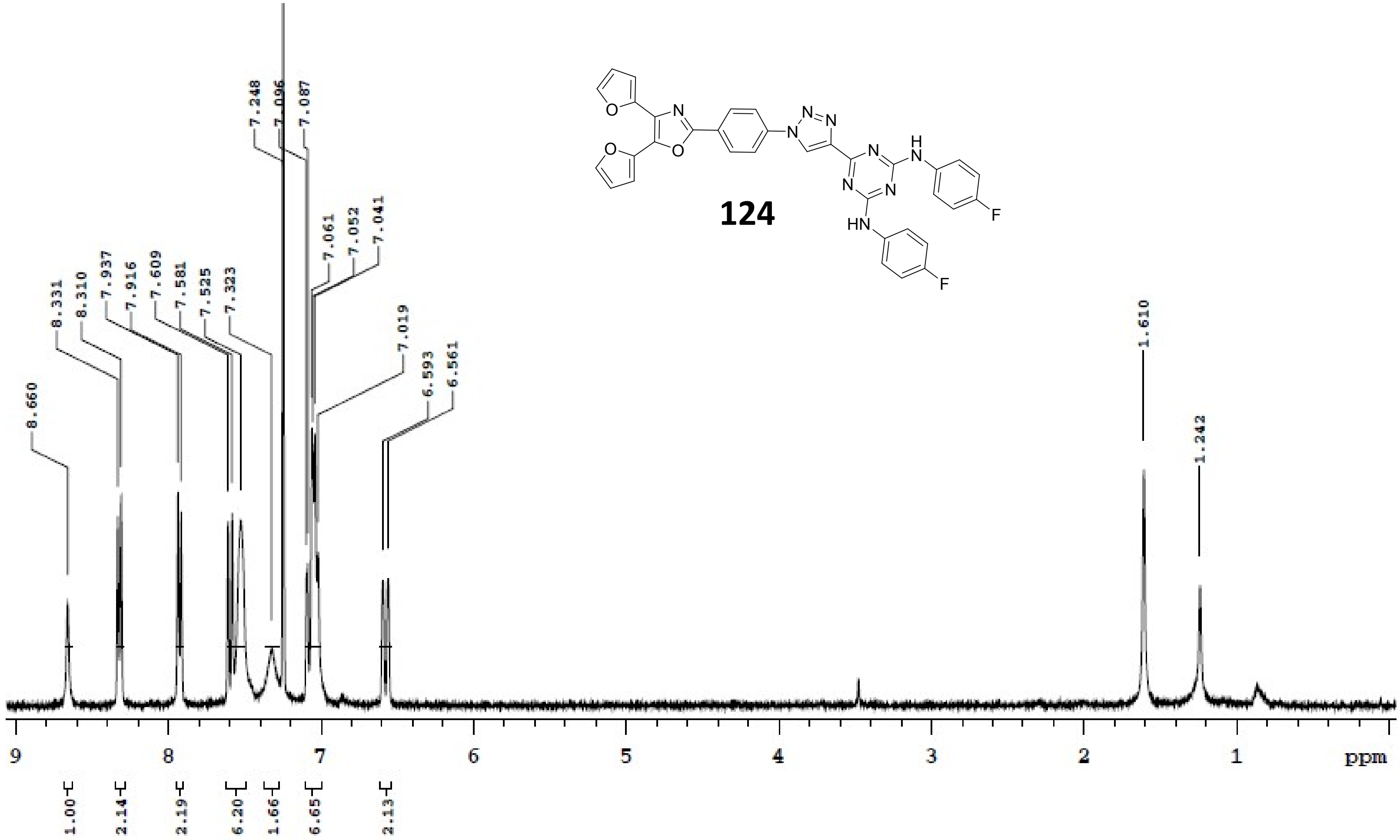

^{13}C NMR: 6-(1-(4-(4,5-di(furan-2-yl)oxazol-2-yl)phenyl)-1H-1,2,3-triazol-4-yl)-N2,N4-bis(4-fluorophenyl)-1,3,5-triazine-2,4-diamine

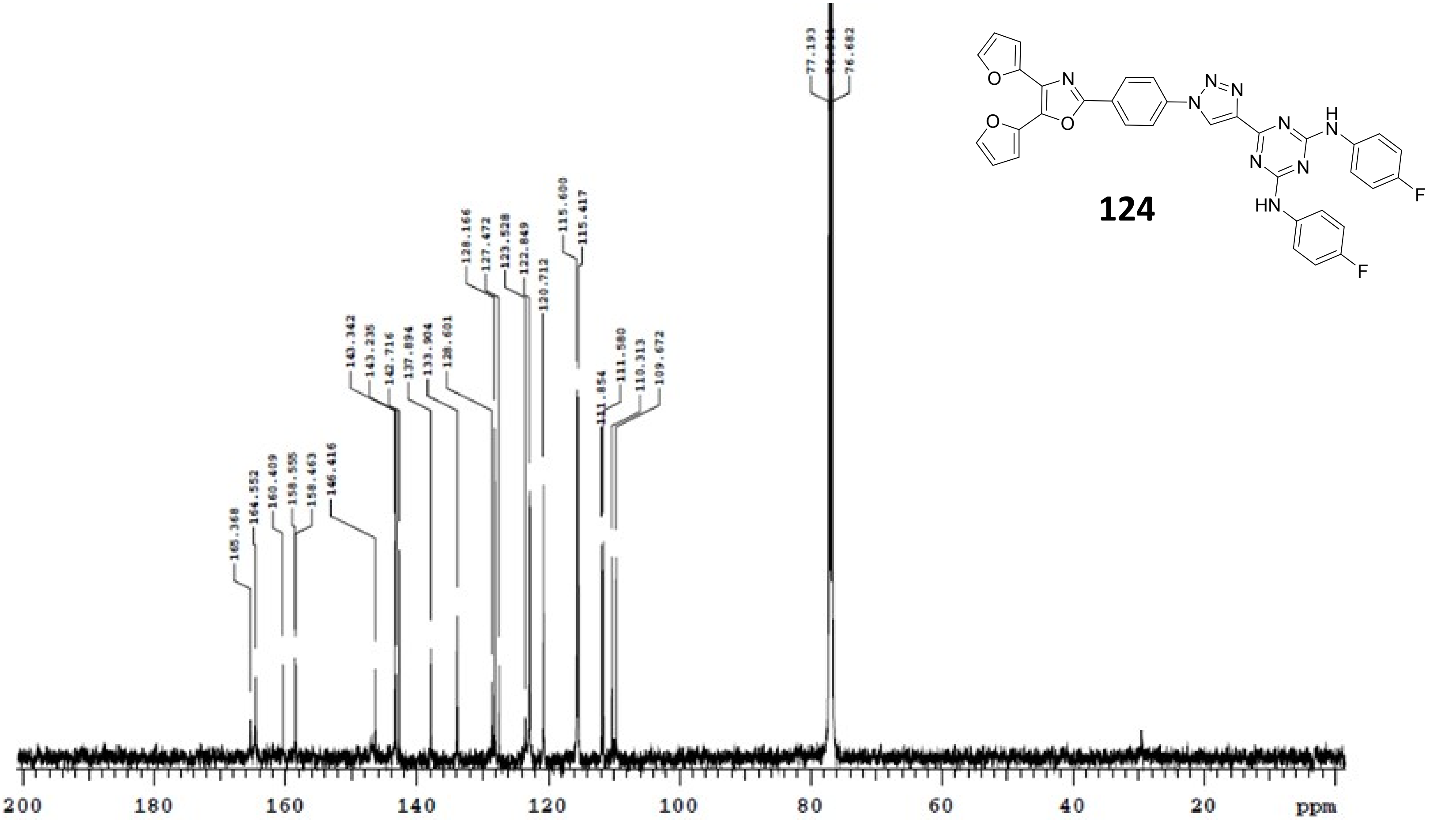

124

References:

1. P.C. Patil, F.A. Luzzio, D.R. Demuth. *Tetrahedron Lett*. 2015, **56**, 3039-3041.
2. P.C. Patil, J. Tan, D.R. Demuth, F.A. Luzzio *Bioorg. Med. Chem.* 2016, **24**, 5410-5417.
